# Radical Group Transfer of Vinyl and Alkynyl Silanes Driven by Photoredox Catalysis

**DOI:** 10.1021/acs.joc.3c01213

**Published:** 2023-08-15

**Authors:** Floriane Baussière, Marius M. Haugland

**Affiliations:** Department of Chemistry, UiT The Arctic University of Norway, 9037 Tromsø, Norway

## Abstract

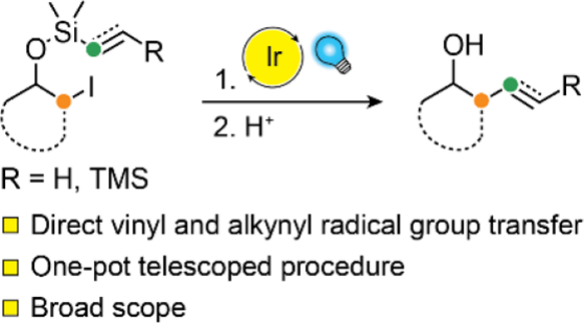

Radical group transfer is a powerful tool for the formation of C–C bonds. These processes typically involve radical addition to C–C π bonds, followed by fragmentation of the resulting cyclic intermediate. Despite the advantageous lability of organosilanes in this context, silicon-tethered radical acceptor groups have remained underexplored in radical group transfer reactions. We report a general photoredox-catalyzed protocol for the radical group transfer of vinyl and alkynyl silanes onto sp^3^ carbons, using activated and unactivated iodides as radical precursors. Our method displays high diastereoselectivity and excellent functional group tolerance, and enables direct formation of group transfer products by in situ ring opening. Mechanistic investigations revealed that the reaction proceeds via an unusual dual catalytic cycle, resulting in an overall redox-neutral process.

## Introduction

**Scheme 1:**
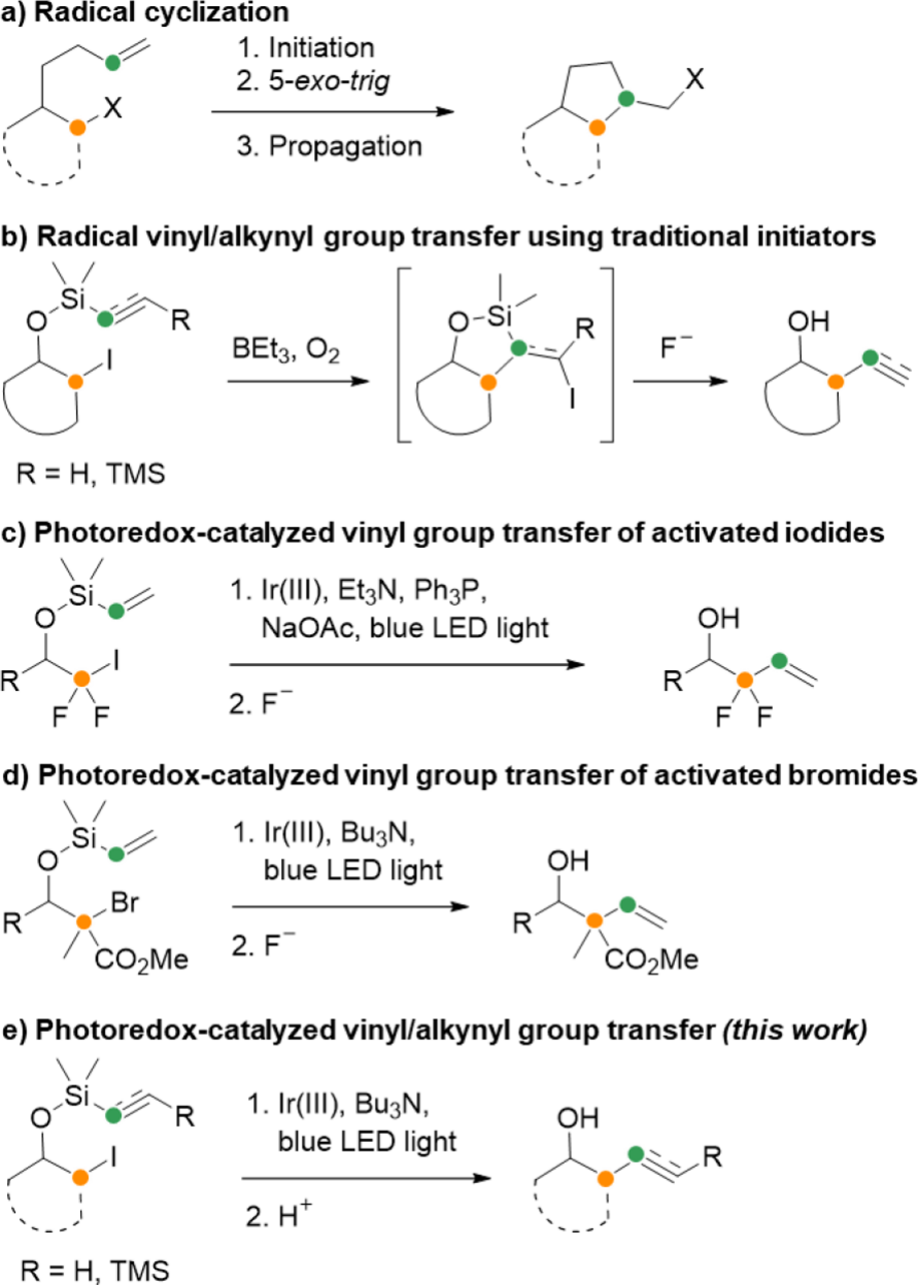
Radical Cyclizations and Group Transfers

Radical cyclizations are a powerful strategy to forge C–C bonds. These intramolecular processes benefit from very rapid kinetics, can generate multiple bonds in one transformation, and enable unique reactivity pathways for the synthesis of complex scaffolds.^1−3^ Although radical processes have traditionally relied on harmful reagents and harsh conditions, photoredox catalysis has emerged as a method of choice for performing radical chemistry under mild, non-toxic conditions.^4,5^ Radical cyclizations typically involve the addition of a carbon-centered radical to a C–C π bond, leading to cyclic products (Scheme 1a).^2,6^ This strategy has been widely employed to produce both saturated and unsaturated all-carbon rings and heterocycles.^1,3,7^ Stereoselective radical cyclizations, moreover, have been used to access enantioenriched cyclic frameworks, which feature, e.g., in many pharmaceuticals and natural products.^8−13^

The use of silicon-substituted alkenes and alkynes in radical cyclizations has, however, remained relatively underexplored. Due to the controllable lability of organosilanes, the cyclic products of these reactions can undergo ring-opening, which leads to an overall group transfer reaction of the vinyl or alkynyl substituent from silicon to carbon. Radical group transfer reactions of vinyl and alkynyl silanes have previously been performed using traditional radical initiators, e.g., on cyclic alkyl iodide substrates (Scheme 1b).^14−16^ Following the radical cyclization reaction, addition of a fluoride source results in elimination and desilylation. A similar strategy has also been applied to activated, tertiary bromides^17^ and in a radical cyclization cascade to set multiple contiguous stereocenters.^18^

More recently, two examples of radical cyclizations with vinyl silanes initiated by photoredox catalysis have been shown. Alkene group transfer was achieved with electronically activated alkyl iodides using an Ir(iii) photocatalyst and irradiation with blue light (Scheme 1c).^19^ Similarly, irradiation of an Ir(iii) photocatalyst resulted in alkene group transfer of tertiary, activated alkyl bromides (Scheme 1d).^20^ Beyond these successful initial demonstrations, a general method for group transfer reactions of silicon-tethered alkenes and alkynes has not yet been reported.

In this work, we present a general photoredox-catalyzed method for the radical group transfer of vinyl and alkynyl silanes. Using activated as well as unactivated alkyl iodides as radical precursors, we demonstrate the installation of alkenes and alkynes on sp^3^ carbon atoms across a broad range of substrates. Our protocol exhibits excellent functional group tolerance and high diastereoselectivity, and enables the removal of the siloxane tether without the use of a fluoride source. Mechanistic investigations, moreover, reveal an unusual dual catalytic mechanism that enables an overall redox-neutral process. We expect the in-depth understanding of this reactivity to facilitate future use of vinyl and alkynyl silanes in radical chemistry.

## Results and Discussion

**Scheme 2:**
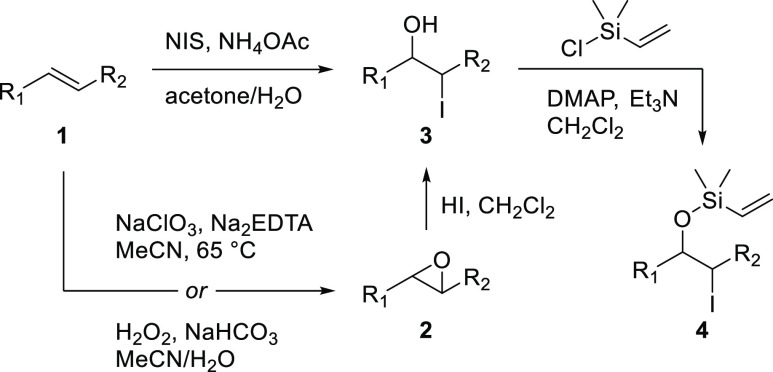
Main Routes for Substrate Synthesis A few substrates required modification of these standard protocols (see the Supporting Information for details).

Substrates for the envisaged radical group transfer were prepared according to the general route shown in Scheme 2. Alkenes (**1**) were transformed, either directly or via an epoxide intermediate (**2**), into iodohydrins (**3**), which were further derivatized into vinyl siloxanes (**4**).

We began our investigation with a screening of different potential metal-based and organic photocatalysts for the photoredox-catalyzed group transfer (Table 1, entries 1–10). Inspired by a previously reported method for heterolytic cleavage of unactivated C–I bonds,^21^ we subjected iodide **4a** to a range of photocatalysts in the presence of an amine electron donor under irradiation by blue LED lights. To our surprise, we observed the direct formation of the alkene group transfer product **5a**. Previous studies have reported the formation of a cyclized intermediate that requires the addition of a fluoride source to facilitate ring-opening/elimination.^20^ Under our conditions, however, no fluoride appears to be required.

**Table 1:**
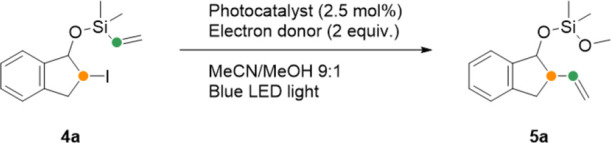
Optimization of Reaction Conditions^a^

entry	photocatalyst	electron donor	time	yield (%)^b^
1	*fac*-Ir(ppy)_3_	Bu_3_N	4 h	75
2	7-(diethylamino)-3-(thiophen-2-yl)-2*H*-chromen-2-one	Bu_3_N	22 h	70
3	perylene	Bu_3_N	4 h	74
4	eosin Y	Bu_3_N	4 h	66
5	bengal rose	Bu_3_N	4 h	70
6	rhodamine B	Bu_3_N	4 h	61
7	4CzIPN	Bu_3_N	2 h	88
8	Ru(bpy)_3_Cl_2_	Bu_3_N	23 h	79
9	[Ir(dtbbpy)(ppy)_2_]PF_6_	Bu_3_N	0.5 h	81
10	[Ir(dF(CF_3_)ppy)_2_ (dtbbpy)]PF_6_	Bu_3_N	1.5 h	72
11	perylene	Et_3_N	18 h	79
12	perylene	DIPEA	1 h	74
13	4CzIPN	Et_3_N	4 h	73
14	4CzIPN	DIPEA	4 h	85
15	[Ir(dtbbpy)(ppy)_2_]PF_6_	Et_3_N	0.5 h	74
16	[Ir(dtbbpy)(ppy)_2_]PF_6_	DIPEA	0.5 h	78

a Reaction scale: 90 μmol.

b Yield determined by quantitative ^1^H NMR with ethylene carbonate as an internal standard.

Unexpectedly, all the photocatalysts tested proved capable of catalyzing the reaction. No clear distinction in the yield was seen between photocatalysts typically going through oxidative (Table 1, entries 1–6) and reductive (Table 1, entries 7–10) quenching pathways, nor between metal-based (Table 1, entries 1, 8–10) and organic (Table 1, entries 2–7) photocatalysts. In line with previous reports of atom transfer radical addition of activated halides with alkenes and alkynes, complete conversion from **4a** to **5a** generally proceeded faster with photocatalysts that undergo reductive quenching.^22^ 4CzIPN (Table 1, entry 7) was selected as the most promising photocatalyst due to its fast reaction time and high yield.

The effect of the tertiary amine used as an electron donor was studied next, with perylene, 4CzIPN, and [Ir(dtbbpy)(ppy)_2_]PF_6_ (Table 1, entries 3, 7, 9, and 11–16). While all three electron donors gave comparable reaction times and yields with [Ir(dtbbpy)(ppy)_2_]PF_6_, the choice of tertiary amine had an impact on the conversion rate with perylene and 4CzIPN. Tributylamine was chosen as the best electron donor as it had a faster conversion rate than triethylamine and DIPEA (Table 1, entries 7, 13, and 14) and provided higher yields.

Based on these results, we applied our selected reaction conditions (Table 1, entry 7) to an open-chain substrate **4f** in a telescoped procedure where the siloxane is cleaved upon treatment with HCl. Unexpectedly, this open-chain analogue of **4a** required 6 h to reach complete conversion (Table 2, entry 1). To improve the reaction rate, 4CzIPN was replaced by the more efficient photocatalyst [Ir(dtbbpy)(ppy)_2_]PF_6_ for further development. A solvent screening performed with **4f** confirmed acetonitrile/methanol 9:1 (Table 2, entry 2) as the best solvent system.

**Table 2:**
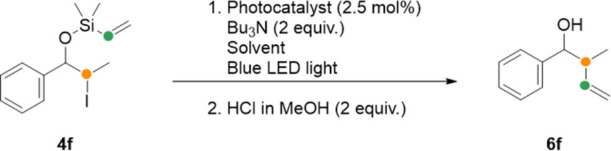
Solvent Screening^a^

entry	photocatalyst	solvent	time^b^	yield (%)^c^
1	4CzIPN	MeCN/MeOH 9:1	6 h	54
2	[Ir(dtbbpy)(ppy)_2_]PF_6_	MeCN/MeOH 9:1	2 h	57
3	[Ir(dtbbpy)(ppy)_2_]PF_6_	MeCN	3 h	55
4	[Ir(dtbbpy)(ppy)_2_]PF_6_	CH_2_Cl_2_	24 h	36
5	[Ir(dtbbpy)(ppy)_2_]PF_6_	EtOAc	24 h	n.d.^d^
6	[Ir(dtbbpy)(ppy)_2_]PF_6_	DMF	24 h	57
7	[Ir(dtbbpy)(ppy)_2_]PF_6_	*t*-BuCN	1 h	42
8	[Ir(dtbbpy)(ppy)_2_]PF_6_	THF	22.5 h	n.d.^d^
9	[Ir(dtbbpy)(ppy)_2_]PF_6_	THF/H_2_O 99:1	22.5 h	n.d.^d^

a Reaction scale: 70 μmol.

b Photoredox catalysis reaction time; acidic desilylation reaction time: 10 min.

c Yield determined by quantitative ^1^H NMR with ethylene carbonate as an internal standard.

d Not determined.

**Scheme 3:**
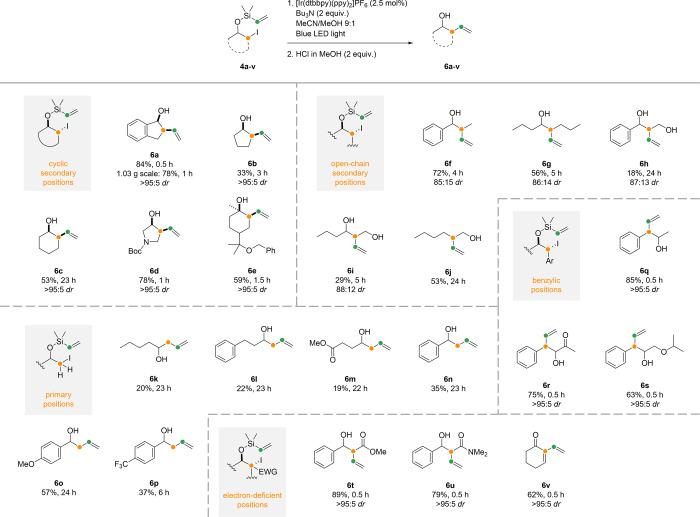
Radical Group Transfer of Vinyl Silanes The products are organized in five categories, depending on the position of the iodide before the radical reaction. Reaction scale: 0.30 mmol.

Next, the optimized photoredox reaction/acidic desilylation procedure was successfully applied to a wide range of substrates (Scheme 3). Although most radical group transfers produced secondary homoallylic alcohols, the protocol also proved applicable to primary (**6j**) and tertiary (**6e**) alcohols. Moreover, the method showed good functional group tolerance toward ketones (**6r**, **6v**), esters (**6m**, **6t**), amides (**6u**), carbamates (**6d**), ethers (**6e**, **6s**), and electron-rich as well as deficient aryl groups (**6o**–**p**). The reaction could also be performed at gram scale with a similar isolated yield (**6a**, Scheme 3). In contrast, no conversion was observed when attempting the reaction with bromide analogues of substrates **4a** and **4g**. This is not surprising, as halogen abstraction of alkyl bromides is known to be more challenging than alkyl iodides due to the higher bond strength of the C–Br bond.^23^

Group transfer reactions of alkyl iodides at secondary positions generally occurred notably faster in cyclic systems (**6a**–**e**) than in open-chain counterparts (**6f**–**j**). The slower reaction rates of open-chain systems could be attributed to their higher entropic degrees of freedom. We were happy to observe that the radical group transfer reaction occurred with high diastereoselectivity in cyclic systems **6a**–**6e**, while open-chain systems **6f**–**6i** produced 12–15% of a minor diastereomer. The reaction at primary positions (**6k**–**p**), however, was slow and gave modest to moderate yields despite complete consumption of the substrates. Primary alkyl radicals are known to be unstable due to the lack of hyperconjugative donation of electron density from adjacent alkyl groups.^24^ This can result in a higher activation energy for the formation of such radicals, thereby explaining the longer reaction times. Benzylic radicals, on the other hand, are known to be stabilized by delocalization.^25,26^ Indeed, we were pleased to observe rapid reactions and high isolated yields at benzylic positions (**6q**–**s**). Similarly, reactions at electron-deficient positions also proceeded rapidly and gave high yields (**6t**–**v**). This has been attributed to the ease of activation of the carbon-iodine bonds in substrates **4q–v**.^27^ The resulting radical intermediates, moreover, are electrophilic and thus polarity-matched for reaction with the electron-rich vinyl silane moiety.^24^ Interestingly, the radical alkene group transfer in open-chain benzylic and electron-deficient systems **6q**–**6v** also proceeded with high diastereoselectivity. A similar effect has been reported previously in group transfer reactions of other vinyl silanes with electron-deficient radicals.^20^ We were pleased to obtain the β,γ-unsaturated carboxylic acid derivatives **6t**–**u** with no migration of the double bond.^28^ Only in ketone **6v** did we observe elimination of the intermediate homoallylic alcohol to produce a conjugated system.

In general, longer reaction times correlated with lower isolated yields, which suggests that the radical group transfer is competing with a slower side reaction. To investigate this hypothesis, the reaction with **4k** was performed in MeCN-*d*_3_/CD_3_OD 9:1, and, upon full consumption of the starting material, directly subjected to analysis by ^1^H NMR with an internal standard. The yield at this stage was 40%, with no sign of elimination or other decomposition products despite the reaction being run in a closed system. ^1^H NMR analysis was repeated after acidic desilylation and after aqueous workup. The yield did not change during desilylation, while a further 25% of the material was lost during the workup. Additional control experiments with **4n** verify that the starting material and product only undergo minor background degradation under the reaction conditions, but that 60% of the material is lost during the photoredox-catalyzed reaction (see the Supporting Information). Replacing Bu_3_N with the more sterically hindered amine 1,2,2,6,6-pentamethylpiperidine results in similar isolated yields, which suggests little or no degradation through S_N_2 substitution of the alkyl iodide. Although no side products could be observed by ^1^H NMR or isolated, we hypothesize that degradation can occur through polymerization when the rate of the group transfer reaction is slow.

**Scheme 4:**
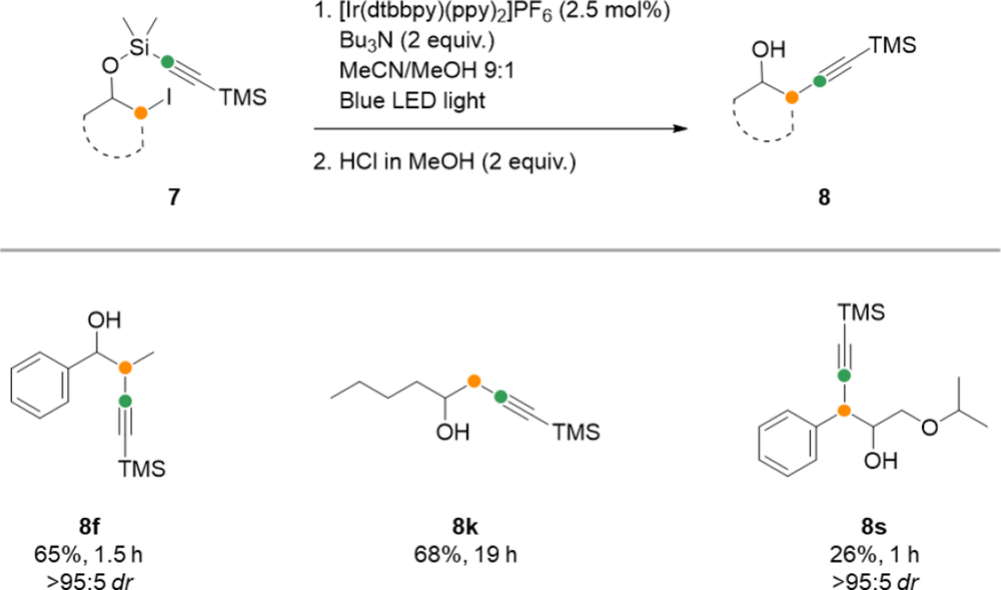
Radical Group Transfer of Alkynyl Silanes Reaction scale: 0.30 mmol.

To our delight, the protocol could also be extended to radical group transfer of alkynes (Scheme 4). Analogous alkynyl silane substrates **7f**, **7k**, and **7s** were prepared from iodohydrins **3** by treatment with an alkynyl aminosilane (see the Supporting Information). After subjecting this material to radical group transfer and desilylation, we obtained products alkynylated at secondary (**8f**), primary (**8k**), and benzylic (**8s**) positions. Under the acidic desilylation conditions, the trimethylsilyl substituent on the alkyne remains intact.

### Mechanistic Investigations

Intrigued by the direct formation of the alkene group transfer product, in contrast to previous studies, we turned our attention to investigating the mechanism of the reaction. No conversion was observed when the reaction was performed in the absence of either the photocatalyst, Bu_3_N, or visible light irradiation (see the Supporting Information) which strongly suggests that the reaction goes through a radical mechanism. In an attempt to trap any radical intermediates with a sufficiently long lifetime, we performed the radical alkene transfer reaction in the presence of 2,2,6,6-tetramethylpiperidine 1-oxyl (TEMPO) as a radical trap. However, no adducts between TEMPO and radical intermediates could be observed.

**Scheme 5:**
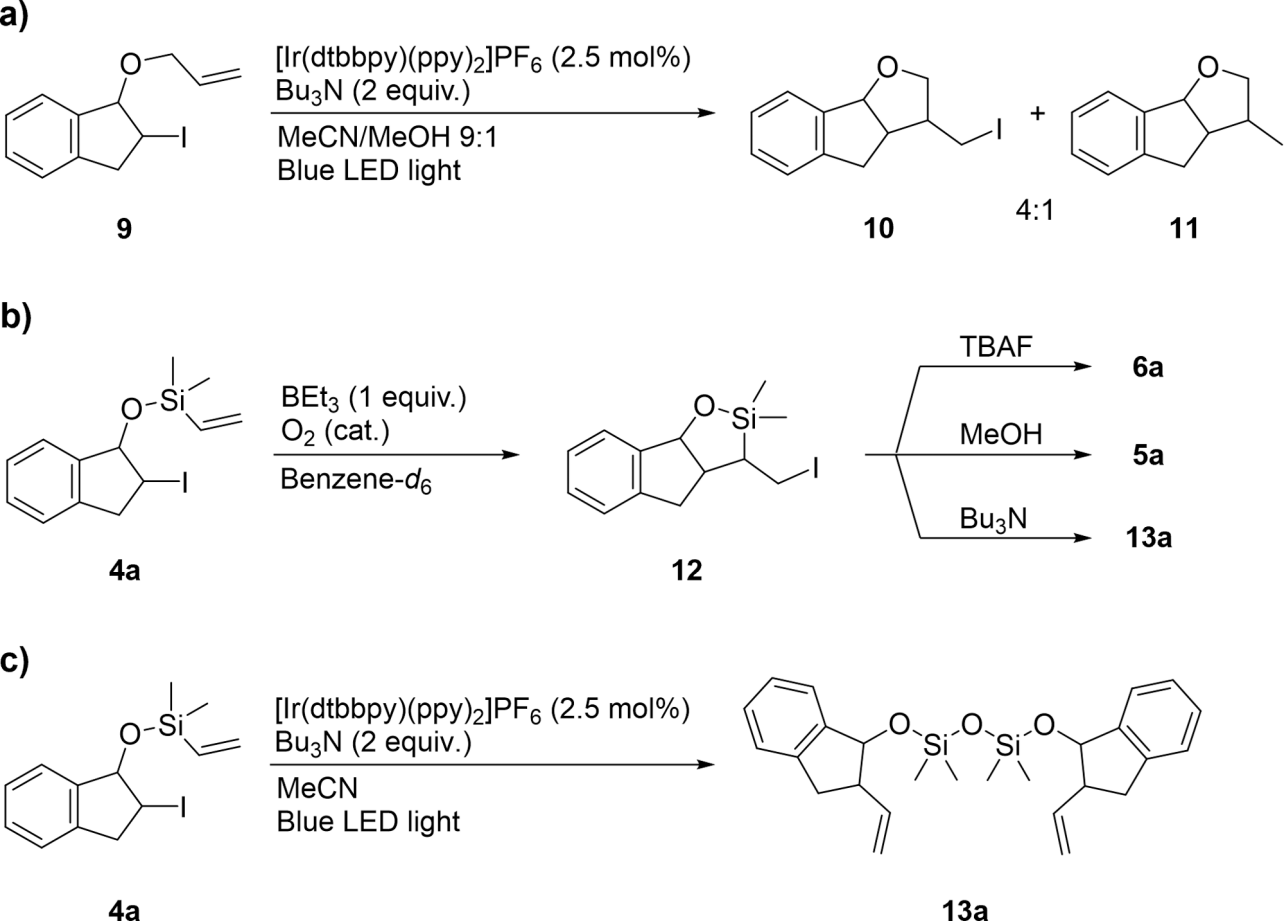
(a–c) Mechanistic Investigations

Next, we set out to investigate the importance of the silicon atom for the group transfer reaction. We subjected the allylic ether **9**, which can be considered an all-carbon analogue of vinyl silane **4a**, to the alkene transfer reaction conditions (Scheme 5a). This resulted in the formation of a 4:1 mixture of halogenated and reduced cyclic products **10** and **11**, respectively, through a 5-*exo-trig* radical cyclization. If vinyl silanes **4** undergo the same cyclization, this implies that the silicon atom can facilitate a subsequent ring-opening process under the reaction conditions. To verify this, iodide **4a** was subjected to BEt_3_ and trace levels of oxygen in benzene-*d*_6_ with ^1^H NMR monitoring. Under these conditions, the cyclized iodide **12** is formed via a radical chain mechanism (Scheme 5b). Addition of tetrabutylammonium fluoride facilitated the formation of the ring-opened and desilylated product **6a**. Addition of methanol or Bu_3_N was also found to promote rapid ring opening, to **5a** and the dimeric disiloxane **13a**, respectively, with methanol reacting faster (see the Supporting Information for details). We therefore propose that under the photoredox-catalyzed reaction conditions, the intermediate siloxane **5** (see Table 1) is formed by nucleophilic attack on the silicon atom of **12**. Notably, as nucleophilic/Lewis basic species coordinate to BEt_3_ and prevent radical generation with O_2_, group transfer reactions with in situ ring opening cannot be performed with this initiator.

**Figure 1:**
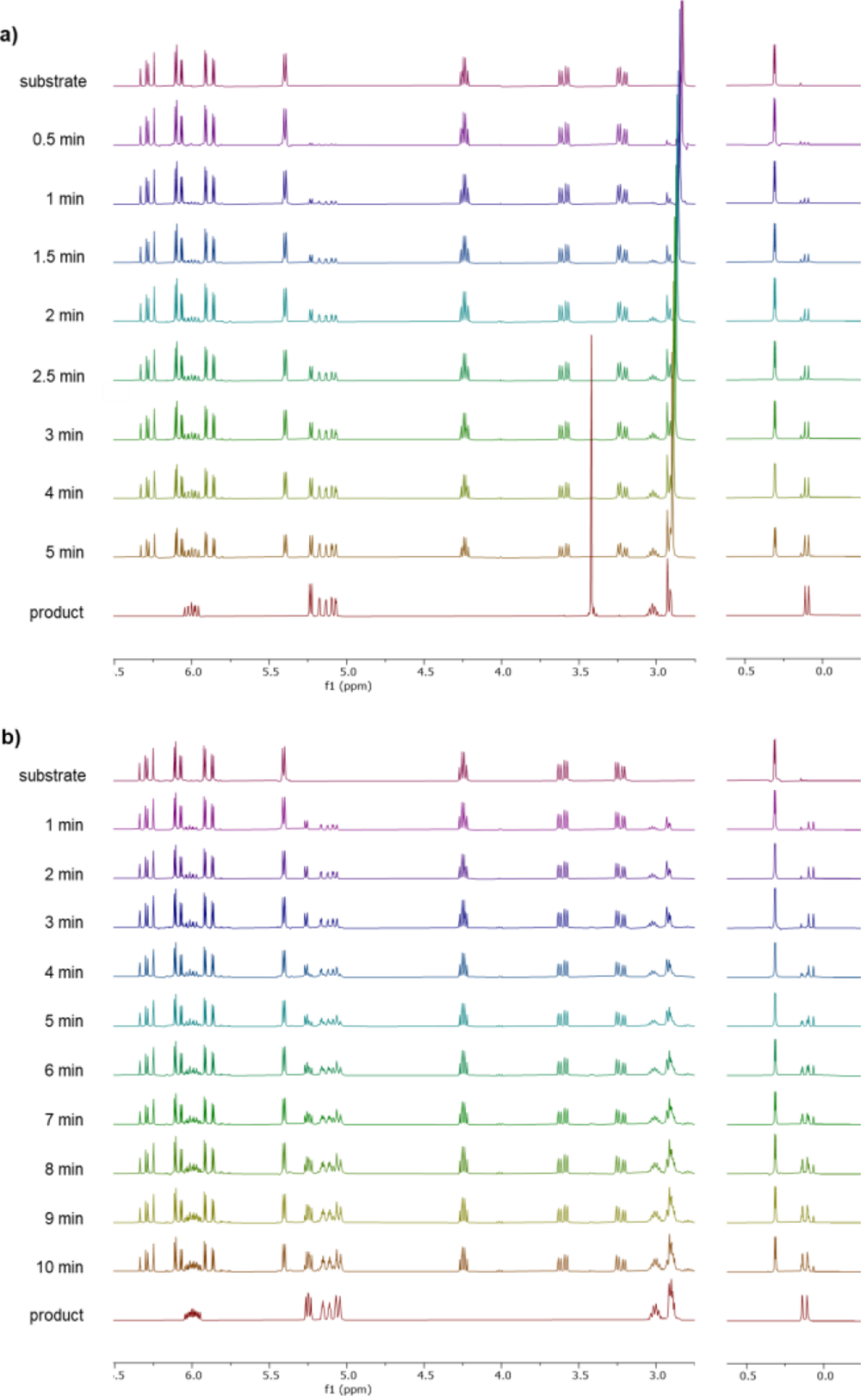
NMR monitoring of the reaction in deuterated solvents (see the Supporting Information for full spectra). Reactions were irradiated with blue LED light for the indicated time, interrupted for ^1^H NMR analysis, and resubmitted to further light irradiation. (a) MeCN-*d*_3_/CD_3_OD 9:1; (b) MeCN-*d*_3_.

When the photoredox-catalyzed reaction was performed in the absence of methanol, the only observed product was a disiloxane-bridged dimer **13a** (Scheme 5c). This suggests that under these conditions, the reaction proceeds through a silylium ion stabilized by a Lewis base (e.g., MeCN or Bu_3_N).^29^ Silylium ions are known to be extremely reactive toward nucleophiles, and even traces of water are known to result in disiloxane formation.^29^ We therefore performed the radical group transfer reaction in a gas-tight NMR tube with direct monitoring by ^1^H NMR spectroscopy, in the presence or absence of methanol-*d*_4_ (Figure 1a,b, respectively). As expected, when methanol was present in the reaction mixture, we observed rapid formation of product **5a** with no detectable intermediates (Figure 1a). In pure acetonitrile, however, the reaction proceeded via an intermediate that could be clearly observed at the start of the reaction. This intermediate was gradually converted into the dimeric product **13a** as the reaction progressed. Thus, we postulate that the observed intermediate is the solvent-stabilized silylium ion, which is subsequently captured by trace amounts of water to produce the dimeric product **13a**.

**Scheme 6:**
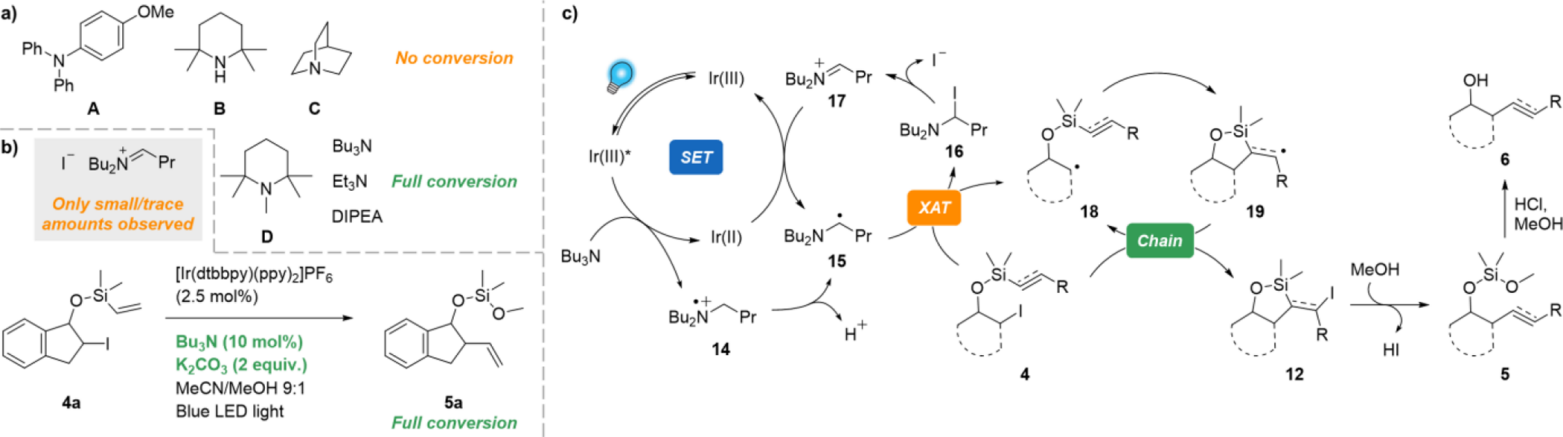
(a–c) Mechanistic Observations and Proposed Mechanism

We were surprised to find that all the photocatalysts tested proved capable of catalyzing the reaction (Table 1). Unactivated halides are known to have more negative reduction potentials (e.g., ethyl iodide, *E*_red_ = −1.67 V vs SCE^30^) than activated halides (α-carbonyl, α-heteroatom, benzylic or allylic) and therefore normally require strongly reducing photocatalysts for efficient radical formation by single-electron transfer (SET).^21,26,31^ This suggests that the reaction rather proceeds via a halogen-atom transfer (XAT) mechanism, where facile SET between photocatalysts and amines with subsequent deprotonation generates α-aminoalkyl radicals. Such radicals are highly potent halogen abstractors that can rapidly generate alkyl radicals from alkyl halides. At the same time, α-haloamines are formed, which in turn dissociate into iminium halide salts.^32,33^ Control experiments substituting Bu_3_N with 4-methoxytriphenylamine (A), 2,2,6,6-tetramethylpiperidine (B), or quinuclidine (C), which cannot participate in XAT, led to no conversion, thereby confirming that the reaction is initiated by an XAT process (Scheme 6a). The reaction could, however, be promoted by 1,2,2,5,5-pentamethylpiperidine (D) and other amines containing the α-hydrogens required for XAT. At the same time, we were surprised to only observe very small or even trace amounts of the iminium halide or related hydrolysis products, even though these side products are expected in stoichiometric quantities in XAT reactions. Moreover, full conversion could also be achieved using 10 mol % of Bu_3_N in the presence of stoichiometric K_2_CO_3_ (Scheme 6b). Given the well-matched reduction potentials of iminium ions (e.g., 1-(4-fluorophenyl)ethan-1-iminium: *E*_red_ = −1.10 V vs SCE^34^) and [Ir(dtbbpy)(ppy)_2_]PF_6_ (*E*_red_ = −1.51 V vs SCE), we propose that the iminium ion can be reduced back to the α-aminoalkyl radical by the reduced photocatalyst in our system, thereby turning over the photocatalytic cycle. Although similar reductions have been reported previously,^34^ we believe this to be the first example of a coupled XAT/iminium SET reduction, leading to an overall process that is catalytic with respect to the amine.

Based on these results, we propose the following mechanism for the photoredox-catalyzed radical group transfer of vinyl and alkynyl groups from silanes (Scheme 6c). Following irradiation with visible light, the excited photocatalyst Ir(III)* effects the single-electron oxidation of Bu_3_N. Subsequent deprotonation of the resulting aminium radical **14** generates the key α-aminoalkyl radical **15**, which partakes in XAT with iodide **4**. The resulting α-iodoamine **16** rapidly collapses into iminium ion **17**, which is then reduced by Ir(II) to regenerate the ground-state photocatalyst and the α-aminoalkyl radical **15**. Alkyl radical **18**, meanwhile, undergoes fast 5-*exo* cyclization to radical **19**, which propagates the reaction through a radical chain mechanism. Nucleophilic attack on the cyclized iodide intermediate **12** by methanol leads to the ring-opened siloxane **5**. Once the group transfer reaction is complete, addition of acid triggers solvolysis into alcohol **6**.

## Conclusions

We have successfully developed a general protocol for the photoredox-catalyzed radical group transfer of vinyl and alkynyl silanes. Our method enables the incorporation of alkenes and alkynes on sp^3^ positions, using either activated or unactivated alkyl iodides as radical precursors. The protocol exhibits high diastereoselectivity and broad functional group tolerance and does not require the use of a fluoride source to drive the ring-opening of the cyclic siloxane intermediate. Instead, we have found that the use of a nucleophilic solvent, which is not compatible with established conventional radical initiators, can promote ring opening to directly yield the group transfer product. Having investigated the mechanism of the reaction, we have identified an XAT reaction coupled with reductive regeneration of the α-aminoalkyl radical halogen abstractor. This unusual dual catalytic cycle results in an overall redox-neutral radical group transfer reaction that can be performed with catalytic amounts of amine. We expect that this process can also be applied in the initiation of other radical transformations, and that the improved understanding of the reactivity of silicon-based radical acceptors can be exploited in the synthesis of complex molecular scaffolds through radical cyclization/fragmentation strategies.^35^

## Experimental Section

All reagents, purchased from Acros, Alfa, Sigma-Aldrich, TCI and VWR, were used as supplied. All air- and/or water-sensitive reactions were carried out under an atmosphere of argon in flame-dried glassware using standard Schlenk techniques. Anhydrous solvents were dried by pre-storing over activated 3 Å (MeOH) or 4 Å (all other solvents) molecular sieves and purged by argon sparging. Blue light irradiation was performed with RGB LED strips (5.4 W). Reactions were monitored by thin-layer chromatography (TLC) on pre-coated aluminum-based plates (TLC Silica gel 60 F_254_, Supelco). The plates were developed under UV irradiation (254 nm) or with vanillin or KMnO_4_ staining and subsequent heating. Column chromatography was performed with silica gel (Silica gel 60, irregular 40–63 μm for flash chromatography, VWR Chemicals). ^1^H NMR spectra were recorded at room temperature on a 400 MHz Bruker 9.4 Tesla Avance III HD system equipped with a SmartProbe (broad band). ^13^C NMR spectra (^1^H decoupled) were recorded at room temperature on the same machine operating at 101 MHz. All chemical shifts (δ) are reported in parts per million (ppm) with internal reference to residual protons/carbons in CDCl_3_ (δ 7.26/77.16) or MeCN-*d*_3_ (δ 1.94/118.26). Coupling constants (*J*) are given in Hz with an accuracy of 0.1 Hz. Infrared (IR) spectra were recorded as thin films or liquids on an Agilent Technologies Cary 630 FTIR 318 spectrometer. Wavelength of maximum absorbance (ν_max_) is reported in wavenumbers (cm^–1^). Only selected characteristic resonances are reported. High-resolution mass spectra (HRMS) were recorded by direct injection of the compounds as solutions in MeOH on a Thermo Scientific Orbitrap Exploris 120 Mass spectrometer, using dual electrospray ionization (ESI) and atmospheric pressure chemical ionization (APCI) probes. Characterization of compounds **1**–**4v** and ^1^H and ^13^C NMR spectra of all compounds are provided in the Supporting Information.

### Procedure I for the Synthesis of Epoxides **2**

Based on a literature procedure,^36^ aqueous 2 M NaOH was added to an aqueous 4 × 10^–4^ M EDTA solution (0.3 M with respect to the olefin) until pH 4. The mixture was added to a solution of olefin (1.0 equiv) in acetonitrile (85 mM with respect to the olefin). Sodium chlorite (3.1 equiv) was added, the reaction flask was equipped with a condenser, and the reaction mixture was stirred at 65 °C overnight. The colorless mixture turned yellow. It was cooled to 0 °C, and the mixture was slowly quenched by dropwise addition of an aqueous 1 M Na_2_S_2_O_3_ solution until no more peroxide was detected by starch/iodide paper. The colorless quenched mixture was reduced in vacuo before being extracted with CH_2_Cl_2_ (3 × 15 mL). The combined organic layers were washed with water and brine, dried over anhydrous Na_2_SO_4_, filtered, and concentrated in vacuo. The crude material was purified by column chromatography to afford the desired epoxide.

### Procedure II for the Synthesis of Epoxides **2**

Based on a literature procedure,^37^ hydrogen peroxide (30% in water, 6.0 equiv) was added to a mixture of olefin (1.0 equiv) and sodium bicarbonate (4.0 equiv) in acetonitrile/water 3:2 (50 mM with respect to the olefin). The reaction mixture was stirred at room temperature for 48 h before being quenched by slow addition of 1 M Na_2_S_2_O_3_ until no more peroxide was detected by starch/iodide paper. The mixture was reduced in vacuo, and the residue was extracted with EtOAc (3 × 10 mL). The combined organic layers were washed with brine, dried over anhydrous Na_2_SO_4_, filtered and concentrated in vacuo. The crude material was purified by column chromatography to afford the desired epoxide.

### Procedure A for the Synthesis of Iodohydrins **3**

Based on a literature procedure,^38^*N*-iodosuccinimide (1.1 equiv) and distilled water (1.0 M with respect to the olefin) were added to a mixture of olefin (1.0 equiv) and ammonium acetate (0.1 equiv) in acetone (0.25 M with respect to the olefin). The reaction mixture was stirred in the dark at room temperature. Upon completion as indicated by TLC, the mixture was concentrated in vacuo, and the residue was redissolved in water and extracted with ethyl acetate (3 × 10 mL). The combined organic layers were washed with an aqueous 10% solution of Na_2_S_2_O_3_, dried over anhydrous Na_2_SO_4_, filtered, and concentrated in vacuo. The crude material was purified by column chromatography to afford the desired iodohydrin.

### Procedure B for the Synthesis of Iodohydrins **3**

Hydroiodic acid (57 wt % in H_2_O, 1.2 equiv) was added to a solution of epoxide **2** (1.0 equiv) in CH_2_Cl_2_ (0.5 M with respect to the epoxide), and the reaction mixture was stirred in the dark at room temperature. Upon completion as indicated by TLC, the mixture was diluted with CH_2_Cl_2_ (20 mL). It was washed with an aqueous 10% solution of NaHCO_3_, an aqueous 10% solution of Na_2_S_2_O_3_, dried over anhydrous Na_2_SO_4_, filtered, and concentrated in vacuo. The crude material was purified by column chromatography to afford the desired iodohydrin.

### Procedure C for the Synthesis of Vinyl Silanes **4**

Chloro(dimethyl)vinylsilane (1.1 equiv) was added to a mixture of iodohydrin **3** (1.0 equiv), 4-dimethylaminopyridine (0.2 equiv), and Et_3_N (1.2 equiv) in dry CH_2_Cl_2_ (0.1 M with respect to the iodohydrin) under Ar. The reaction mixture was stirred for 20 min in the dark at room temperature. The reaction was quenched by slowly adding a few drops of isopropanol, and the mixture was concentrated in vacuo. The residue was redissolved in water and extracted with Et_2_O (3 × 10 mL). The combined organic layers were washed with a saturated aqueous NaHCO_3_ solution and brine, dried over anhydrous Na_2_SO_4_, filtered, and concentrated in vacuo. The crude material was purified by column chromatography to afford the desired vinyl silane.

### Procedure D for the Synthesis of Siloxanes **5**

Vinyl silane **4** (1.0 equiv) was added to a mixture of [Ir(dtbbpy)(ppy)_2_]PF_6_ (2.5 mol %) and Bu_3_N (2.0 equiv) in dry MeCN/MeOH 9:1 (0.1 M with respect to the silane) under Ar. The mixture was degassed by Ar sparging for 5 min before placing the reaction vial 1 cm away from the light source. A fan was placed on top of the setup to keep the reaction environment at room temperature. The reaction mixture was irradiated with blue LED light until full consumption of the substrate as indicated by TLC. The mixture was reduced in vacuo, and the crude material was purified by column chromatography to afford the desired siloxane.

**Methoxydimethyl((2-vinyl-2,3-dihydro-1*H*-inden-1-yl)oxy)silane (5a)** was synthesized from vinyl silane **4a** (42 mg, 0.12 mmol, 1.0 equiv), [Ir(dtbbpy)(ppy)_2_]PF_6_ (2.5 mg, 2.7 μmol, 2.2 mol %), and Bu_3_N (58 μL, 0.24 mmol, 2.0 equiv) in MeCN/MeOH 9:1 (1.2 mL) according to Procedure D. The crude was purified by column chromatography (Et_2_O/pentane 5%) to afford siloxane **5a** (20 mg, 79 μmol, 65%) as a colorless oil. **R_*f*_** 0.27 (Et_2_O/pentane 2.5%); IR (liquid, ν_max_/cm^–1^) 2962, 2940, 2910, 2840, 1261, 1063, 1022, 989, 888, 851, 799, 754; ^1^H NMR (400 MHz, CDCl_3_) δ 7.40–7.34 (1H, m, Ar*H*), 7.30–7.26 (1H, m, Ar*H*), 7.23 (2H, m, Ar*H*), 6.05 (1H, ddd, *J* = 17.3, 10.3, 7.8 Hz, C*H*CH_2_), 5.26 (1H, d, *J* = 5.4 Hz, C*H*O), 5.18 (1H, ddd, *J* = 17.4, 2.1, 0.9 Hz, CHC*H*_2_), 5.13 (1H, ddd, *J* = 10.4, 2.1, 0.6 Hz, CHC*H*_2_), 3.49 (3H, s, OC*H*_3_), 3.11–2.92 (3H, m, C*H*(CHCH_2_), C*H*_2_), 0.20 (3H, s, Si(C*H*_3_)_2_), 0.17 (3H, s, Si(C*H*_3_)_2_); ^13^C{^1^H} NMR (CDCl_3_, 101 MHz) δ 144.6, 142.9, 138.4, 128.3, 126.7, 124.9, 124.8, 115.9, 77.7, 50.39, 50.37, 36.5, −2.76, −2.81; HRMS (ESI) calc. for C_14_H_20_O_2_SiNa ([M + Na]^+^) 271.1125, found 271.1124.

**Methoxydimethyl(oct-1-en-4-yloxy)silane (5k)** was synthesized from vinyl silane **4k** (0.21 g, 0.68 mmol, 1.0 equiv), [Ir(dtbbpy)(ppy)_2_]PF_6_ (15 mg, 16 μmol, 2.4 mol %), and Bu_3_N (0.35 mL, 1.5 mmol, 2.2 equiv) in MeCN/MeOH 9:1 (7.7 mL) according to Procedure D. The crude was purified by column chromatography (Et_2_O/pentane 2.5%) to afford an inseparable 1:2 mixture of substrate **4k** (6.3 mg, 20 μmol) and siloxane **5k** (8.7 mg, 40 μmol, 6%) as a colorless oil. *Note: the NMR data were extracted from the analysis of the inseparable mixture.***R_*f*_** 0.26 (Et_2_O/pentane 2.5%); IR (liquid, ν_max_/cm^–1^) 2959, 2933, 2862, 1256, 1085, 1057, 914, 837, 788, 729; ^1^H NMR (400 MHz, CDCl_3_) δ 5.90–5.75 (1H, m, C*H*CH_2_), 5.12–4.99 (2H, m, CHC*H*_2_), 3.80 (1H, m, C*H*O), 3.49 (3H, s, OC*H*_3_), 2.25 (2H, m, CHOC*H*_2_CHCH_2_), 1.54–1.40 (2H, m, C*H*_2_CHO), 1.40–1.19 (4H, m, 2 × C*H*_2_), 0.94–0.85 (3H, m, C*H*_3_), 0.13 (6H, s, Si(C*H*_3_)_2_); ^13^C{^1^H} NMR (CDCl_3_, 101 MHz) δ 135.4, 117.0, 72.3, 50.3, 42.1, 36.7, 27.9, 22.9, 14.2, −2.87, −2.92; HRMS (ESI) calc. for C_11_H_24_O_2_SiNa ([M + Na]^+^) 239.1438, found 239.1437.

**Methoxydimethyl((1-phenylbut-3-en-1-yl)oxy)silane (5n)** was synthesized from vinyl silane **4n** (1.0 g, 3.1 mmol, 1.0 equiv), [Ir(dtbbpy)(ppy)_2_]PF_6_ (65 mg, 71 μmol, 2.3 mol %), and Bu_3_N (1.4 mL, 5.9 mmol, 1.9 equiv) in MeCN/MeOH 9:1 (30 mL) according to Procedure D. The crude was purified by column chromatography (Et_2_O/pentane 5%) to afford siloxane **5n** (0.17 g, 0.73 mmol, 24%) as a colorless oil. **R_*f*_** 0.37 (Et_2_O/pentane 5%); IR (liquid, ν_max_/cm^–1^) 2961, 2836, 1258, 1193, 1081, 1068, 994, 916, 845, 795, 700; **NMR** (400 MHz, CDCl_3_) δ 7.36–7.28 (4H, m, Ar*H*), 7.26–7.21 (1H, m, Ar*H*), 5.78 (1H, ddt, *J* = 17.2, 10.2, 7.0 Hz, C*H*CH_2_), 5.09–4.99 (2H, m, CHC*H*_2_), 4.81 (1H, m, C*H*O), 3.34 (3H, s, OC*H*_3_), 2.59–2.39 (2H, m, CHOC*H*_2_), 0.10 (3H, s, Si(C*H*_3_)_2_), 0.02 (3H, s, Si(C*H*_3_)_2_); ^13^C{^1^H} NMR (CDCl_3_, 101 MHz) δ 144.5, 135.1, 128.3 (2C), 127.3, 126.1 (2C), 117.3, 74.7, 50.3, 45.0, −2.99, −3.05; HRMS (ESI) calc. for C_13_H_20_O_2_SiNa ([M + Na]^+^) 259.1125, found 259.1122.

### Procedure E for the Synthesis of Homoallylic Alcohols **6** and Homopropargylic Alcohols **8**

Silylated substrate **4** or **7** (1.0 equiv) was added to a mixture of [Ir(dtbbpy)(ppy)_2_]PF_6_ (2.5 mol %) and Bu_3_N (2.0 equiv) in dry MeCN/MeOH 9:1 (0.1 M with respect to the silane) under Ar. The mixture was degassed by Ar sparging for 5 min before placing the reaction vial 1 cm away from the light source. A fan was placed on top of the setup to keep the reaction environment at room temperature. The reaction mixture was irradiated with blue LED light until full consumption of the substrate as indicated by TLC. After turning off the blue LED light, a solution of HCl 1.25 M in MeOH (2.0 equiv) was added, and the reaction mixture was stirred for 10 min at room temperature. Brine (2 mL) was added before concentrating the mixture in vacuo. The residue was further diluted with brine (10 mL) before being extracted with EtOAc (3 × 5 mL). The combined organic layers were washed with an aqueous 10% NaHCO_3_ solution and brine, dried over anhydrous Na_2_SO_4_, filtered, and concentrated in vacuo. The crude material was purified by column chromatography to afford the desired product.

**2-Vinyl-2,3-dihydro-1*H*-inden-1-ol (6a)** was synthesized from vinyl silane **4a** (0.11 g, 0.31 mmol, 1.0 equiv; *gram scale: 1.0 g, 3.0 mmol, 1.0 equiv),* [Ir(dtbbpy)(ppy)_2_]PF_6_ (6.4 mg, 7.0 μmol, 2.4 mol %; *gram scale: 59 mg, 65 μmol, 2.2 mol %),* Bu_3_N (0.14 mL, 0.59 mmol, 1.9 equiv; *gram scale: 1.4 mL, 6.0 mmol, 2.0 equiv*) in MeCN/MeOH 9:1 (3 mL; *gram scale: 30 mL*) and HCl 1.25 M in MeOH (0.50 mL, 0.63 mmol, 2.0 equiv; *gram scale: 4.8 mL, 6.0 mmol, 2.0 equiv*) according to Procedure E. The crude was purified by column chromatography (IPA/CH_2_Cl_2_ 1%) to afford **6a** (42 mg, 0.26 mmol, 84%; *gram scale: 0.38 g, 2.3 mmol, 78%*) as a colorless oil. ^1^H NMR (400 MHz, CDCl_3_) δ 7.44 (1H, d, *J* = 6.8 Hz, Ar*H*), 7.28 (3H, d, *J* = 3.6 Hz, Ar*H*), 5.99 (1H, ddd, *J* = 17.8, 10.5, 7.8 Hz, C*H*CH_2_), 5.30 (1H, d, *J* = 9.0 Hz, CHC*H*_2_), 5.27 (1H, s, CHC*H*_2_), 5.10 (1H, t, *J* = 5.2 Hz, C*H*O), 3.15 (1H, p, *J* = 7.1 Hz, C*H*(CHCH_2_)), 3.02 (2H, d, *J* = 7.1 Hz, C*H*_2_), 1.82 (1H, d, *J* = 4.8 Hz, O*H*); ^13^C{^1^H} NMR (CDCl_3_, 101 MHz) δ 144.3, 142.7, 137.0, 128.6, 126.9, 125.1, 124.9, 117.9, 77.2, 49.6, 35.4. Data consistent with literature values.^39^

**2-Ethynylcyclopentanol (6b)** was synthesized from vinyl silane **4b** (91 mg, 0.31 mmol, 1.0 equiv), [Ir(dtbbpy)(ppy)_2_]PF_6_ (6.4 mg, 7.0 μmol, 2.2 mol %), Bu_3_N (0.14 mL, 0.59 mmol, 1.9 equiv) in MeCN/MeOH 9:1 (3 mL), and HCl 1.25 M in MeOH (0.50 mL, 0.63 mmol, 2.0 equiv) according to Procedure E. The crude was purified by column chromatography (IPA/CH_2_Cl_2_ 0.5%) to afford **6b** (11 mg, 0.10 mmol, 33%) as a colorless oil. ^1^H NMR (400 MHz, CDCl_3_) δ 5.95 (1H, ddd, *J* = 17.3, 10.6, 6.8 Hz, C*H*CH_2_), 5.24–5.10 (2H, m, CHC*H*_2_), 4.16 (1H, dh, *J* = 6.7, 2.2 Hz, C*H*OH), 2.47 (1H, tddt, *J* = 8.1, 6.7, 4.4, 1.4 Hz, C*H*(CHCH_2_)), 1.86 (2H, tdd, *J* = 12.4, 9.1, 5.9 Hz, C*H*_2_), 1.79–1.53 (4H, m, 2 × C*H*_2_), 1.42 (1H, d, *J* = 3.0 Hz, O*H*); ^13^C{^1^H} NMR (CDCl_3_, 101 MHz) δ 137.7, 117.0, 75.4, 49.8, 34.2, 27.5, 22.1. Data consistent with literature values.^40^

**2-Vinylcyclohexan-1-ol (6c)** was synthesized from vinyl silane **4c** (94 mg, 0.30 mmol, 1.0 equiv), [Ir(dtbbpy)(ppy)_2_]PF_6_ (6.3 mg, 7.0 μmol, 2.3 mol %), Bu_3_N (0.14 mL, 0.59 mmol, 1.9 equiv) in MeCN/MeOH 9:1 (3 mL), and HCl 1.25 M in MeOH (0.50 mL, 0.63 mmol, 2.1 equiv) according to Procedure E. The crude was purified by column chromatography (IPA/CH_2_Cl_2_ 1%). A short second column chromatography (IPA/CH_2_Cl_2_ 0.5%) afforded **6c** (20 mg, 0.16 mmol, 53%) as a colorless oil. ^1^H NMR (400 MHz, CDCl_3_) δ 5.94 (1H, ddd, *J* = 17.3, 10.6, 6.6 Hz, C*H*CH_2_), 5.25–5.02 (2H, m, CHC*H*_2_), 3.86 (1H, s, C*H*OH), 2.27 (1H, dd, *J* = 10.4, 5.8 Hz, C*H*(CHCH_2_)), 1.83–1.20 (9H, m, 4 × C*H*_2_, O*H*); ^13^C{^1^H} NMR (CDCl_3_, 101 MHz) δ 140.0, 116.1, 69.4, 45.4, 32.3, 25.7, 24.4, 21.0. Data consistent with literature values.^41^

***tert*-Butyl 3-hydroxy-4-vinylpyrrolidine-1-carboxylate (6d)** was synthesized from vinyl silane **4d** (0.12 mg, 0.29 mmol, 1.0 equiv), [Ir(dtbbpy)(ppy)_2_]PF_6_ (6.4 mg, 7.0 μmol, 2.4 mol %), Bu_3_N (0.14 mL, 0.59 mmol, 2.0 equiv) in MeCN/MeOH 9:1 (3 mL), and HCl 1.25 M in MeOH (0.50 mL, 0.63 mmol, 2.1 equiv) according to Procedure E. The crude was purified by column chromatography (IPA/CH_2_Cl_2_ 2%) to afford **6d** (49 mg, 0.23 mmol, 78%) as a colorless oil. ^1^H NMR (400 MHz, CDCl_3_, mixture of rotamers) δ 5.89 (1H, dddd, *J* = 14.8, 11.0, 6.9, 4.5 Hz, CH(C*H*CH_2_)), 5.31–5.12 (2H, m, CH(CHC*H*_2_)), 4.24 (1H, dd, *J* = 3.6, 2.1 Hz, C*H*OH), 3.61–3.40 (3H, m, 3 × C*H*_2_), 3.34 (1H, dt, *J* = 12.4, 10.5 Hz, C*H*_2_), 2.84–2.71 (1H, m, C*H*(CHCH_2_)), 2.27 (1H, dd, *J* = 48.5, 3.5 Hz, O*H*), 1.43 (9H, s, 3 × C*H*_3_); ^13^C{^1^H} NMR (CDCl_3_, 101 MHz, mixture of rotamers) δ 154.8, 133.9, 133.7, 119.0, 118.6, 79.5, 72.8, 72.2, 54.3, 54.2, 48.1, 47.7, 47.2, 47.1, 28.6. Data consistent with literature values.^42^

**4-(2-Benzyloxy)propan-2-yl)-1-methyl-2-vinylcyclohexan-1-ol (6e)** was synthesized from vinyl silane **4e** (0.14 g, 0.30 mmol, 1.0 equiv), [Ir(dtbbpy)(ppy)_2_]PF_6_ (6.3 mg, 7.0 μmol, 2.4 mol %), Bu_3_N (0.14 mL, 0.60 mmol, 2.0 equiv) in MeCN/MeOH 9:1 (3 mL), and HCl 1.25 M in MeOH (0.50 mL, 0.63 mmol, 2.1 equiv) according to Procedure E. The crude was purified by column chromatography (IPA/CH_2_Cl_2_ 0.5–1%). A short second column chromatography (Et_2_O/pentane 30%) afforded **6e** (51 mg, 0.18 mmol, 59%) as a colorless oil. **R_*f*_** 0.35 (Et_2_O/pentane 40%); IR (thin film, ν_max_/cm^–1^) 2969, 2933, 2869, 1455, 1385, 1368, 1174, 1129, 1088, 1059, 1030, 998, 911, 734, 698; ^1^H NMR (400 MHz, CDCl_3_) δ 7.39–7.19 (5H, m, Ar*H*), 5.94 (1H, ddd, *J* = 17.2, 10.5, 8.1 Hz, C*H*CH_2_), 5.19–5.02 (2H, m, CHC*H*_2_), 4.44 (2H, s, OC*H*_2_Ph), 2.00 (1H, ddd, *J* = 12.0, 8.1, 3.6 Hz, C*H*(CHCH_2_)), 1.79 (1H, dt, *J* = 9.8, 2.6 Hz, C*H*_2_C(CH_3_)OH), 1.73–1.56 (3H, m, 2 × C*H*_2_, C*H*C(CH_3_)_2_), 1.53–1.34 (3H, m, 2 × C*H*_2_, C*H*_2_C(CH_3_)OH), 1.23 (7H, d, *J* = 2.7 Hz, C(C*H*_3_)_2_, O*H*), 1.19 (3H, s, C(C*H*_3_)OH); ^13^C{^1^H} NMR (CDCl_3_, 101 MHz) δ 140.2, 139.5, 128.4 (2C), 127.3 (2C), 127.1, 116.3, 77.3, 70.1, 63.3, 50.9, 46.1, 40.0, 29.3, 28.6, 23.1, 23.0, 22.7; HRMS (ESI) calc. for C_19_H_28_O_2_Na ([M + Na]^+^) 311.1982, 311.1981.

**2-Methyl-1-phenylbut-3-en-1-ol** (**6f)** was synthesized from vinyl silane **4f** (0.10 g, 0.30 mmol, 1.0 equiv), [Ir(dtbbpy)(ppy)_2_]PF_6_ (6.2 mg, 7.0 μmol, 2.2 mol %), Bu_3_N (0.14 mL, 0.59 mmol, 2.0 equiv) in MeCN/MeOH 9:1 (3 mL), and HCl 1.25 M in MeOH (0.50 mL, 0.63 mmol, 2.1 equiv) according to Procedure E. The crude was purified by column chromatography (IPA/CH_2_Cl_2_ 1%) to afford an inseparable 87:13 diastereomers mixture of **6f** (35 mg, 0.22 mmol, 72%) as a colorless oil. *Note: the NMR data were extracted from the analysis of the inseparable mixture.*^1^H NMR (400 MHz, CDCl_3_, *major*) δ 7.45–7.12 (5H, m, Ar*H*), 5.79 (1H, dt, *J* = 17.8, 9.1 Hz, C*H*CH_2_), 5.25–5.08 (2H, m, CHC*H*_2_), 4.33 (1H, d, *J* = 7.9 Hz, C*H*OH), 2.46 (1H, h, *J* = 7.5 Hz, C*H*CH_3_), 2.18 (1H, s, O*H*), 0.85 (3H, d, *J* = 6.8 Hz, C*H*_3_); ^13^C NMR (CDCl_3_, 101 MHz, *major*) δ 142.6, 140.8, 128.3 (2C), 127.7, 127.0 (2C), 116.9, 78.0, 46.4, 16.6. ^1^H NMR (400 MHz, CDCl_3_, *minor*) δ 7.45–7.12 (5H, m, Ar*H*), 5.70 (1H, d, *J* = 8.6 Hz, C*H*CH_2_), 5.09–4.93 (2H, m, CHC*H*_2_), 4.57 (1H, t, *J* = 4.3 Hz, C*H*OH), 2.56 (1H, q, *J* = 6.8 Hz, C*H*CH_3_), 0.99 (3H, d, *J* = 6.9 Hz, C*H*_3_); ^13^C{^1^H} NMR (CDCl_3_, 101 MHz, *minor*) δ 142.7, 140.4, 128.2 (2C), 127.4, 126.6 (2C), 115.6, 77.4, 44.8, 14.1. Data consistent with literature values.^43^

**5-Vinyloctan-4-ol (6g)** was synthesized from vinyl silane **4g** (0.10 g, 0.30 mmol, 1.0 equiv), [Ir(dtbbpy)(ppy)_2_]PF_6_ (6.6 mg, 7.2 μmol, 2.4 mol %), Bu_3_N (0.14 mL, 0.59 mmol, 1.9 equiv) in MeCN/MeOH 9:1 (3 mL), and HCl 1.25 M in MeOH (0.50 mL, 0.63 mmol, 2.1 equiv) according to Procedure E. The crude was purified by column chromatography (IPA/CH_2_Cl_2_ 0.5%) to afford an inseparable 86:14 diastereomers mixture of **6g** (27 mg, 0.17 mmol, 56%) as a colorless oil. *Note: the NMR data were extracted from the analysis of the inseparable mixture.***R_*f*_** 0.36 (IPA/CH_2_Cl_2_ 1%); IR (thin film, ν_max_/cm^–1^) 3351, 2958, 2930, 2874, 1639, 1467, 1459, 1423, 1380, 1119, 1060, 1022, 912, 848; ^1^H NMR (400 MHz, CDCl_3_, *major*) δ 5.63 (1H, m, (C*H*CH_2_)), 5.22–5.00 (2H, m, (CHC*H*_2_)), 3.45 (1H, m, C*H*OH), 2.04–1.96 (1H, m, C*H*(CHCH_2_)), 1.60–1.13 (9H, m, 4 × C*H*_2_, O*H*), 0.90 (6H, m, 2 × C*H*_3_); ^13^C{^1^H} NMR (CDCl_3_, 101 MHz, *major*) δ 139.1, 117.8, 73.5, 50.3, 37.0, 33.1, 20.6, 19.1, 14.3, 14.2. ^1^H NMR (400 MHz, CDCl_3_, *minor*) δ 5.63 (1H, m, (C*H*CH_2_)), 5.22–5.00 (2H, m, (CHC*H*_2_)), 3.45 (1H, m, C*H*OH), 2.12–2.07 (1H, m, C*H*(CHCH_2_)), 1.60–1.13 (9H, m, 4 × C*H*_2_, O*H*), 0.90 (6H, m, 2 × C*H*_3_); ^13^C{^1^H} NMR (CDCl_3_, 101 MHz, *minor*) δ 139.5, 117.1, 74.3, 50.7, 36.2, 32.3, 20.6, 19.4, 14.2 (2C); HRMS (ESI) calc. for C_10_H_20_ONa ([M + Na]^+^) 179.1406, found 179.1406.

**1-Phenyl-2-vinylpropane-1,3-diol (6h)** was synthesized from vinyl silane **4h** (0.14 g, 0.30 mmol, 1.0 equiv), [Ir(dtbbpy)(ppy)_2_]PF_6_ (6.8 mg, 7.3 μmol, 2.4 mol %), Bu_3_N (0.14 mL, 0.59 mmol, 1.9 equiv) in MeCN/MeOH 9:1 (3 mL), and HCl 1.25 M in MeOH (0.50 mL, 0.63 mmol, 2.1 equiv) according to Procedure E. The crude was purified by column chromatography (Et_2_O/pentane 20%). A short second column chromatography (IPA/CH_2_Cl_2_ 1–5%) afforded an inseparable 87:13 diastereomers mixture of **6 h** (9.6 mg, 54 μmol, 18%) as a colorless oil. *Note: the NMR data were extracted from the analysis of the inseparable mixture.*^1^H NMR (400 MHz, CDCl_3_, *major*) δ 7.35–7.11 (5H, m, Ar*H*), 5.74 (1H, ddd, *J* = 17.4, 10.5, 8.7 Hz, CHC*H*CH_2_), 5.24–5.16 (1H, m, CHCHC*H*_2_), 5.10 (1H, m, CHCHC*H*_2_), 4.75 (1H, m, ArC*H*OH), 3.55 (2H, m, C*H*_2_OH), 2.53 (1H,m, ArCH(*OH*)C*H*), 2.35 (1H, d, *J* = 3.2 Hz, ArCHO*H*), 1.66–1.63 (1H, m, CH_2_O*H*); ^13^C{^1^H} NMR (CDCl_3_, 101 MHz, *major*) δ 142.2, 135.4, 128.5 (2C), 127.9, 126.6 (2C), 119.9, 75.1, 63.9, 53.7. ^1^H NMR (400 MHz, CDCl_3_, *minor*) δ 7.36–7.10 (5H, m, Ar*H*), 5.53 (1H, m, CHC*H*CH_2_), 5.03–4.90 (2H, m, CHCHC*H*_2_), 4.71 (1H, m, ArC*H*OH), 3.83–3.75 (1H, m, C*H*_2_OH), 3.71 (1H, m, C*H*_2_OH), 2.60 (2H, m, ArCH(*OH*)C*H*, ArCHO*H*); ^13^C{^1^H} NMR (CDCl_3_, 101 MHz, *minor*) δ 142.7, 135.6, 128.5 (2C), 128.0, 126.8 (2C), 118.4, 77.3, 65.0, 52.3. Data consistent with literature values.^44^

**2-Vinylhexan-1,3-diol (6i)** was synthesized from vinyl silane **4i** (0.13 g, 0.29 mmol, 1.0 equiv), [Ir(dtbbpy)(ppy)_2_]PF_6_ (6.4 mg, 7.0 μmol, 2.3 mol %), Bu_3_N (0.14 mL, 0.59 mmol, 2.0 equiv) in MeCN/MeOH 9:1 (3 mL), and HCl 1.25 M in MeOH (0.50 mL, 0.63 mmol, 2.2 equiv) according to Procedure E. The crude was purified by column chromatography (IPA/CH_2_Cl_2_ 1–5%). A short second column chromatography (Et_2_O/pentane 10–100%) afforded an inseparable 88:12 diastereomers mixture of **6i** (12 mg, 84 μmol, 29%) as a colorless oil. *Note: the NMR data were extracted from the analysis of the inseparable mixture.*^1^H NMR (400 MHz, CDCl_3_, *major*) δ 5.85 (1H, ddd, *J* = 17.3, 10.4, 8.8 Hz, CH(C*H*CH_2_)), 5.27–5.24 (1H, m, CH(CHC*H*_2_)), 5.16–5.15 (1H, m, CH(CHC*H*_2_)), 3.83–3.73 (3H, m, C*H*OH, C*H*_2_OH), 2.33–2.27 (1H, m, C*H*(CHCH_2_)), 2.24 (1H, m, CHO*H*), 2.18–2.17 (1H, m, CH_2_O*H*), 1.52–1.30 (4H, m, 4 × C*H*_2_), 0.94–0.91 (3H, m, C*H*_3_); ^13^C{^1^H} NMR (CDCl_3_, 101 MHz, *major*) δ 134.9, 119.1, 72.7, 64.9, 50.9, 37.2, 19.1, 14.2. ^1^H NMR (400 MHz, CDCl_3_, *minor*) δ 5.69–5.60 (1H, m, CH(C*H*CH_2_)), 5.27–5.08 (2H, m, CH(CHC*H*_2_)), 3.83–3.73 (3H, m, C*H*OH, C*H*_2_OH), 2.33–2.27 (1H, m, C*H*(CHCH_2_)), 2.24 (1H, m, CHO*H*), 2.18–2.17 (1H, m, CH_2_O*H*), 1.52–1.30 (4H, m, 4 × C*H*_2_), 0.94–0.91 (3H, m, C*H*_3_); ^13^C{^1^H} NMR (CDCl_3_, 101 MHz, *minor*) δ 136.4, 118.2, 74.6, 65.4, 51.7, 37.8, 18.6, 14.2. Data consistent with literature values.^45^

**2-Vinylhexan-1-ol** (**6j)** was synthesized from vinyl silane **4j** (99 mg, 0.29 mmol, 1.0 equiv), [Ir(dtbbpy)(ppy)_2_]PF_6_ (6.5 mg, 7.1 μmol, 2.5 mol %), Bu_3_N (0.14 mL, 0.59 mmol, 2.1 equiv) in MeCN/MeOH 9:1 (3 mL), and HCl 1.25 M in MeOH (0.50 mL, 0.63 mmol, 2.2 equiv) according to Procedure E. The crude was purified by column chromatography (IPA/CH_2_Cl_2_ 1%). A short second column chromatography (Et_2_O/pentane 50%) afforded **6j** (20 mg, 0.15 mmol, 53%) as a colorless oil. ^1^H NMR (400 MHz, CDCl_3_) δ 5.58 (1H, dt, *J* = 18.0, 9.6 Hz, (C*H*CH_2_)), 5.13 (2H, dd, *J* = 13.7, 9.6 Hz, (CHC*H*_2_)), 3.55 (1H, q, *J* = 5.4 Hz, C*H*_2_OH), 3.40 (1H, t, *J* = 9.4 Hz, C*H*_2_OH), 2.30–2.08 (1H, m, C*H*(CHCH_2_)), 1.51 (1H, s, O*H*), 1.46–1.16 (6H, m, 3 × C*H*_2_), 0.88 (3H, t, *J* = 6.6 Hz, C*H*_3_); ^13^C{^1^H} NMR (CDCl_3_, 101 MHz) δ 140.3, 117.3, 65.8, 47.2, 30.5, 29.4, 22.9, 14.1. Data consistent with literature values.^46^

**Oct-1-en-4-ol (6k)** was synthesized from vinyl silane **4k** (78 mg, 0.25 mmol, 1.0 equiv), [Ir(dtbbpy)(ppy)_2_]PF_6_ (5.2 mg, 5.7 μmol, 2.3 mol %), Bu_3_N (0.12 mL, 0.48 mmol, 1.9 equiv) in MeCN/MeOH 9:1 (2.5 mL), and HCl 1.25 M in MeOH (0.40 mL, 0.51 mmol, 2.1 equiv) according to Procedure E. The crude was purified by column chromatography (IPA/CH_2_Cl_2_ 0.5%) to afford **6k** (6.2 mg, 48 μmol, 20%) as a colorless oil. ^1^H NMR (400 MHz, CDCl_3_) δ 5.83 (1H, ddd, *J* = 17.4, 14.7, 7.7 Hz, C*H*CH_2_), 5.22–5.04 (2H, m, CHC*H*_2_), 3.64 (1H, t, *J* = 6.3 Hz, C*H*OH), 2.31 (1H, dt, *J* = 11.6, 5.3 Hz, C*H*_2_CHCH_2_), 2.14 (1H, dt, *J* = 14.7, 7.9 Hz, C*H*_2_CHCH_2_), 1.63–1.20 (9H, m, 3 × C*H*_2_, O*H*), 0.91 (3H, t, *J* = 6.8 Hz, C*H*_3_); ^13^C{^1^H} NMR (CDCl_3_, 101 MHz) δ 135.1, 118.2, 70.8, 42.1, 36.7, 28.0, 22.9, 14.2. Data consistent with literature values.^47^

**1-Phenylhex-5-en-3-ol (6l)** was synthesized from vinyl silane **4l** (59 mg, 0.16 mmol, 1.0 equiv), [Ir(dtbbpy)(ppy)_2_]PF_6_ (3.4 mg, 3.8 μmol, 2.3 mol %), Bu_3_N (76 μL, 0.32 mmol, 1.9 equiv) in MeCN/MeOH 9:1 (1.7 mL) and HCl 1.25 M in MeOH (0.27 mL, 0.34 mmol, 2.1 equiv) according to Procedure E. The crude was purified by column chromatography (IPA/CH_2_Cl_2_ 1%) to afford **6l** (6.2 mg, 35 μmol, 22%) as a colorless oil. ^1^H NMR (400 MHz, CDCl_3_) δ 7.47–7.12 (5H, m, Ar*H*), 5.86 (1H, ddd, *J* = 17.2, 14.6, 7.7 Hz, C*H*CH_2_), 5.28–5.09 (2H, m, CHC*H*_2_), 3.82–3.62 (1H, m, C*H*OH), 2.86 (1H, m, C*H*_2_CH_2_CHOH), 2.73 (1H, m, C*H*_2_CH_2_CHOH), 2.37 (1H, m, CHOHC*H*_2_CH), 2.23 (1H, m, CHOHC*H*_2_CH), 1.84 (2H, m, CH_2_C*H*_2_CHOH), 1.65 (1H, m, O*H*); ^13^C{^1^H} NMR (CDCl_3_, 101 MHz) δ 142.2, 134.7, 128.6 (2C), 128.5 (2C), 126.0, 118.5, 70.1, 42.2, 38.6, 32.2. Data consistent with literature values.^48^

**Methyl 4-hydroxyhept-6-enoate (6m)** was synthesized from vinyl silane **4m** (0.10 g, 0.30 mmol, 1.0 equiv), [Ir(dtbbpy)(ppy)_2_]PF_6_ (6.6 mg, 7.2 μmol, 2.4 mol %), Bu_3_N (0.14 mL, 0.59 mmol, 2.0 equiv) in MeCN/MeOH 9:1 (3 mL) and HCl 1.25 M in MeOH (0.50 mL, 0.63 mmol, 2.1 equiv) according to Procedure E. The crude was purified by column chromatography (IPA/CH_2_Cl_2_ 2%). A short second column chromatography (Et_2_O/pentane 50%) afforded **6m** (8.8 mg, 56 μmol, 19%) as a colorless oil. **R_*f*_** 0.31 (IPA/CH_2_Cl_2_ 5%); IR (thin film, ν_max_/cm^–1^) 3457, 2952, 2923, 1738, 1441, 1357, 1219, 1208, 1170, 1082, 1074, 998, 918; ^1^H NMR (400 MHz, CDCl_3_) δ 5.82 (1H, m, C*H*CH_2_), 5.20–5.07 (2H, m, CHC*H*_2_), 3.68 (4H, s, C*H*OH, C*H*_3_), 2.48 (2H, td, *J* = 7.3, 2.5 Hz, C(*O*)C*H*_2_), 2.35–2.24 (1H, m, CH(*OH*)C*H*_2_CH), 2.19 (1H, m, CH(*OH*)C*H*_2_CH), 1.92–1.79 (2H, m, C*H*_2_CH(OH), O*H*), 1.73 (1H, m, C*H*_2_CH(OH)); ^13^C{^1^H} NMR (CDCl_3_, 101 MHz) δ 174.6, 134.5, 118.6, 70.1, 51.8, 42.2, 31.7, 30.6; HRMS (ESI) calc. for C_8_H_14_O_3_Na ([M + Na]^+^) 181.0835, found 181.0834.

**1-Phenylbut-3-en-1-ol (6n)** was synthesized from vinyl silane **4n** (0.10 g, 0.30 mmol, 1.0 equiv), [Ir(dtbbpy)(ppy)_2_]PF_6_ (6.6 mg, 7.2 μmol, 2.4 mol %), Bu_3_N (0.14 mL, 0.59 mmol, 1.9 equiv) in MeCN/MeOH 9:1 (3 mL), and HCl 1.25 M in MeOH (0.50 mL, 0.63 mmol, 2.1 equiv) according to Procedure E. The crude was purified by column chromatography (IPA/CH_2_Cl_2_ 1%) to afford **6n** (16 mg, 0.11 mmol, 35%) as a colorless oil. ^1^H NMR (400 MHz, CDCl_3_) δ 7.31–7.10 (5H, m, Ar*H*), 5.71 (1H, ddt, *J* = 17.2, 10.1, 7.2 Hz, C*H*CH_2_), 5.14–4.99 (2H, m, CHC*H*_2_), 4.63 (1H, m, C*H*OH), 2.50–2.32 (2H, m, CHOHC*H*_2_), 1.96 (1H, m, O*H*); ^13^C{^1^H} NMR (CDCl_3_, 101 MHz) δ 144.0, 134.6, 128.6 (2C), 127.7, 125.9 (2C), 118.6, 73.4, 44.0. Data consistent with literature values.^49^

**1-(4-Methoxyphenyl)but-3-en-1-ol (6o)** was synthesized from vinyl silane **4o** (0.11 g, 0.30 mmol, 1.0 equiv), [Ir(dtbbpy)(ppy)_2_]PF_6_ (6.6 mg, 7.2 μmol, 2.4 mol %), Bu_3_N (0.14 mL, 0.59 mmol, 2.0 equiv) in MeCN/MeOH 9:1 (3 mL) and HCl 1.25 M in MeOH (0.50 mL, 0.63 mmol, 2.1 equiv) according to Procedure E. The crude was purified by column chromatography (IPA/CH_2_Cl_2_ 0–4%) to afford **6o** (30 mg, 0.17 mmol, 57%) as a colorless oil. ^1^H NMR (400 MHz, CDCl_3_) δ 7.28 (2H, dd, *J* = 8.6, 1.7 Hz, Ar*H*), 6.89 (2H, dd, *J* = 8.6, 1.8 Hz, Ar*H*), 5.89–5.71 (1H, m, C*H*CH_2_), 5.25–5.06 (2H, m, CHC*H*_2_), 4.76–4.62 (1H, m, C*H*OH), 3.81 (3H, d, *J* = 1.7 Hz, OC*H*_3_), 2.50 (2H, t, *J* = 6.9 Hz, CH(*OH*)C*H*_2_), 1.96 (1H, dd, *J* = 3.1, 1.6 Hz, O*H*); ^13^C{^1^H} NMR (CDCl_3_, 101 MHz) δ 159.2, 136.2, 134.8, 127.2 (2C), 118.4, 114.0 (2C), 73.1, 55.4, 43.9. Data consistent with literature values.^50^

**1-(4-(Trifluoromethyl)phenyl)but-3-en-1-ol (6p)** was synthesized from vinyl silane **4p** (0.12 g, 0.30 mmol, 1.0 equiv), [Ir(dtbbpy)(ppy)_2_]PF_6_ (7.2 mg, 7.9 μmol, 2.6 mol %), Bu_3_N (0.14 mL, 0.59 mmol, 1.9 equiv) in MeCN/MeOH 9:1 (3 mL) and HCl 1.25 M in MeOH (0.50 mL, 0.63 mmol, 2.1 equiv) according to Procedure E. The crude was purified by column chromatography (IPA/CH_2_Cl_2_ 0.5%) to afford **6p** (24 mg, 0.11 mmol, 37%) as a colorless oil. ^1^H NMR (400 MHz, CDCl_3_) δ 7.61 (2H, d, *J* = 8.1 Hz, Ar*H*), 7.54–7.41 (2H, m, Ar*H*), 5.87–5.70 (1H, m, CH(*OH*)CH_2_C*H*CH_2_), 5.25–5.12 (2H, m, CH(*OH*)CH_2_CHC*H*_2_), 4.80 (1H, dt, *J* = 8.1, 3.9 Hz, C*H*OH), 2.60–2.39 (2H, m, CH(*OH*)C*H*_2_CHCH_2_), 2.19 (1H, d, *J* = 3.3 Hz, O*H*); ^13^C{^1^H} NMR (CDCl_3_, 101 MHz) δ 147.9, 133.8, 129.8 (q, ^2^*J*_C–F_ = 32.3 Hz), 126.2 (2C), 125.5 (2C, d, ^3^*J*_C–F_ = 3.8 Hz), 124.3 (q, ^1^*J*_C–F_ = 272.2 Hz), 119.4, 72.7, 44.0. Data consistent with literature values.^51^

**3-Phenylpent-4-en-2-ol (6q)** was synthesized from vinyl silane **4q** (0.10 g, 0.30 mmol, 1.0 equiv), [Ir(dtbbpy)(ppy)_2_]PF_6_ (6.6 mg, 7.2 μmol, 2.4 mol %), Bu_3_N (0.14 mL, 0.59 mmol, 1.9 equiv) in MeCN/MeOH 9:1 (3 mL) and HCl 1.25 M in MeOH (0.50 mL, 0.63 mmol, 2.1 equiv) according to Procedure E. The crude was purified by column chromatography (IPA/CH_2_Cl_2_ 10.5–1%) to afford **6q** (42 mg, 0.26 mmol, 85%) as a colorless oil. ^1^H NMR (400 MHz, CDCl_3_) δ 7.37–7.22 (5H, m, Ar*H*), 6.20–6.11 (1H, m, C*H*CH_2_), 5.28–5.24 (2H, m, CHC*H*_2_), 4.01 (1H, dq, *J* = 7.7, 6.2 Hz, C*H*(CHCH_2_)), 3.20 (1H, t, *J* = 8.4 Hz, C*H*OH), 1.96 (1H, m, O*H*), 1.11 (3H, d, *J* = 6.2 Hz, C*H*_3_); ^13^C{^1^H} NMR (CDCl_3_, 101 MHz) δ 141.6, 138.6, 128.8 (2C), 128.1 (2C), 126.8, 118.1, 70.3, 59.3, 20.7. Data consistent with literature values.^52^

**3-Hydroxy-4-phenylhex-5-en-2-one (6r)** was synthesized from vinyl silane **4r** (0.11 g, 0.30 mmol, 1.0 equiv), [Ir(dtbbpy)(ppy)_2_]PF_6_ (6.6 mg, 7.2 μmol, 2.4 mol %), Bu_3_N (0.14 mL, 0.59 mmol, 1.9 equiv) in MeCN/MeOH 9:1 (3 mL) and HCl 1.25 M in MeOH (0.50 mL, 0.63 mmol, 2.1 equiv) according to Procedure E. The crude was purified by column chromatography (IPA/CH_2_Cl_2_ 0.5–1%). A short second column chromatography (Et_2_O/pentane 50%) afforded **6r** (43 mg, 0.22 mmol, 75%) as a yellow oil. ^1^H NMR (400 MHz, CDCl_3_) δ 7.41–7.32 (4H, m, Ar*H*), 7.27 (1H, m, Ar*H*), 6.09 (1H, ddd, *J* = 17.1, 10.3, 8.9 Hz, C*H*CH_2_), 5.25–5.09 (2H, m, CHC*H*_2_), 4.48 (1H, dd, *J* = 4.9, 3.4 Hz, C*H*OH), 3.76 (1H, dd, *J* = 8.9, 3.5 Hz, C*H*(CHCH_2_)), 3.55 (1H, d, *J* = 5.0 Hz, O*H*), 2.19 (3H, s, C*H*_3_); ^13^C{^1^H} NMR (CDCl_3_, 101 MHz) δ 208.6, 140.6, 135.1, 128.8 (2C), 128.2 (2C), 127.1, 118.3, 80.4, 52.8, 26.2. Data consistent with literature values.^53^

**1-Isopropoxy-3-phenylpent-4-en-2-ol (6s)** was synthesized from vinyl silane **4s** (82 mg, 0.20 mmol, 1.0 equiv), [Ir(dtbbpy)(ppy)_2_]PF_6_ (4.5 mg, 4.9 μmol, 2.4 mol %), Bu_3_N (92 μL, 0.39 mmol, 1.9 equiv) in MeCN/MeOH 9:1 (2 mL) and HCl 1.25 M in MeOH (0.33 mL, 0.41 mmol, 2.0 equiv) according to Procedure E. The crude was purified by column chromatography (IPA/CH_2_Cl_2_ 0.5–1%). A short second column chromatography (Et_2_O/pentane 30%) afforded **6s** (28 mg, 0.13 mmol, 63%) as a light-yellow oil. **R_*f*_** 0.26 (Et_2_O/pentane 30%); IR (thin film, ν_max_/cm^–1^) 2972, 2868, 1454, 1382, 1370, 1128, 1076, 916, 760, 701; ^1^H NMR (400 MHz, CDCl_3_) δ 7.37–7.17 (5H, m, Ar*H*), 6.20 (1H, ddd, *J* = 17.1, 10.3, 8.3 Hz, C*H*CH_2_), 5.25–5.08 (2H, m, CHC*H*_2_), 3.98 (1H, ddt, *J* = 8.1, 6.8, 3.4 Hz, C*H*OH), 3.48 (1H, p, *J* = 6.1 Hz, C*H*(CH_3_)_2_), 3.41 (1H, t, *J* = 8.2 Hz, C*H*(CHCH_2_)), 3.33 (1H, dd, *J* = 9.5, 3.1 Hz, CH_2_O), 3.15 (1H, dd, *J* = 9.5, 6.8 Hz, CH_2_O), 2.51 (1H, d, *J* = 3.7 Hz, O*H*), 1.11 (6H, dd, *J* = 6.1, 2.2 Hz, 2 × C*H*_3_); ^13^C{^1^H} NMR (CDCl_3_, 101 MHz) δ 141.3, 138.7, 128.7 (2C), 128.2 (2C), 126.8, 117.1, 73.2, 72.2, 70.1, 53.5, 22.2, 22.1; HRMS (APCI) calc. for C_14_H_21_O_2_ ([M + H]^+^) 221.1536, found 221.1536.

**Methyl 2-(hydroxy(phenyl)methyl)but-3-enoate (6t)** was synthesized from vinyl silane **4t** (0.12 g, 0.31 mmol, 1.0 equiv), [Ir(dtbbpy)(ppy)_2_]PF_6_ (6.6 mg, 7.2 μmol, 2.3 mol %), Bu_3_N (0.14 mL, 0.59 mmol, 1.9 equiv) in MeCN/MeOH 9:1 (3 mL) and HCl 1.25 M in MeOH (0.50 mL, 0.63 mmol, 2.0 equiv) according to Procedure E. The crude was purified by column chromatography (IPA/CH_2_Cl_2_ 0.5–1%). A short second column chromatography (Et_2_O/pentane 50%) afforded **6t** (57 mg, 0.28 mmol, 89%) as a colorless oil. ^1^H NMR (400 MHz, CDCl_3_) δ 7.29 (5H, m, Ar*H*), 6.03–5.85 (1H, m, C*H*CH_2_), 5.25 (1H, m, CHC*H*_2_), 5.14 (1H, m, CHC*H*_2_), 5.01 (1H, m, C*H*O), 3.59 (3H, m, OC*H*_3_), 3.33 (1H, m, C*H*(CHCH_2_)), 2.93 (1H, m, O*H*); ^13^C{^1^H} NMR (CDCl_3_, 101 MHz) δ 173.0, 140.8, 131.8, 128.4 (2C), 128.0, 126.4 (2C), 120.8, 74.0, 58.3, 52.1. Data consistent with literature values.^54^

**2-(Hydroxy(phenyl)methyl)-*N*,*N*-dimethylbut-3-enamide (6u)** was synthesized from vinyl silane **4u** (0.13 g, 0.32 mmol, 1.0 equiv), [Ir(dtbbpy)(ppy)_2_]PF_6_ (7.3 mg, 8.0 μmol, 2.5 mol %), Bu_3_N (0.14 mL, 0.59 mmol, 1.9 equiv) in MeCN/MeOH 9:1 (3 mL) and HCl 1.25 M in MeOH (0.50 mL, 0.63 mmol, 2.0 equiv) according to Procedure E. The crude was purified by column chromatography (IPA/CH_2_Cl_2_ 1%) to afford **6u** (55 mg, 0.25 mmol, 79%) as a yellow oil. **R_*f*_** 0.27 (Et_2_O/pentane 80%); IR (thin film, ν_max_/cm^–1^) 3391, 2931, 1618, 1493, 1453, 1400, 1145, 1058, 922, 702; ^1^H NMR (400 MHz, CDCl_3_) δ 7.33–7.13 (5H, m, Ar*H*), 5.76 (1H, ddd, *J* = 17.4, 10.3, 8.1 Hz, C*H*CH_2_), 5.21–5.11 (1H, m, CHC*H*_2_), 5.08 (1H, m, C*H*OH), 4.98–4.92 (1H, m, O*H*), 4.85 (1H, m, CHC*H*_2_), 3.38 (1H, dd, *J* = 8.1, 3.3 Hz, C*H*CHCH_2_), 2.90 (3H, s, NC*H*_3_), 2.85 (3H, s, NC*H*_3_); ^13^C{^1^H} NMR (CDCl_3_, 101 MHz) δ 173.7, 141.2, 131.1, 128.0 (2C), 127.3, 126.3 (2C), 120.0, 73.7, 53.4, 37.3, 35.6; HRMS (APCI) calc. for C_13_H_18_NO_2_ ([M + H]^+^) 220.1332, found 220.1327.

**2-Vinyl-2-cyclohexenone (6v)** was synthesized from vinyl silane **4v** (97 mg, 0.30 mmol, 1.0 equiv), [Ir(dtbbpy)(ppy)_2_]PF_6_ (5.5 mg, 6.0 mol, 2.0 mol %), Bu_3_N (0.14 mL, 0.59 mmol, 2.0 equiv) in MeCN/MeOH 9:1 (3 mL) and HCl 1.25 M in MeOH (0.50 mL, 0.63 mmol, 2.1 equiv) according to Procedure E. The crude was purified by column chromatography (IPA/CH_2_Cl_2_ 0.5–1%) to afford **6v** (23 mg, 0.19 mmol, 62%) as a colorless oil. ^1^H NMR (400 MHz, CDCl_3_) δ 7.02 (1H, t, *J* = 4.5 Hz, CC*H*), 6.53 (1H, dd, *J* = 17.7, 11.2 Hz, C*H*CH_2_), 5.70–5.58 (1H, m, CHC*H*_2_), 5.14 (1H, d, *J* = 11.2 Hz, CHC*H*_2_), 2.44 (4H, m, 2 × C*H*_2_), 1.99 (2H, m, C*H*_2_); ^13^C{^1^H} NMR (CDCl_3_, 101 MHz) δ 198.5, 145.4, 136.7, 131.4, 115.8, 38.8, 26.4, 22.7. Data consistent with literature values.^55^

### Procedure F for the Synthesis of Alkynyl Silanes **7**

Based on a literature procedure,^56^ methyl iodide (2.2 equiv) was added dropwise to a solution of aminosilane **s2** (see the Supporting Information) (1.1 equiv) in dry CH_2_Cl_2_ (0.5 M with respect to the aminosilane) under Ar. The mixture was stirred for 1 h at room temperature before being cannulated into a mixture of iodohydrin **3** (1.0 equiv) and imidazole (1.1 equiv) in dry CH_2_Cl_2_ (0.2 M with respect to the iodohydrin) under Ar. After stirring for 1 h, the mixture was concentrated in vacuo. The residue was redissolved in EtOAc and was washed with an aqueous 10% Na_2_S_2_O_3_ solution and brine. The organic layer was dried over anhydrous Na_2_SO_4_, filtered, and concentrated in vacuo. The crude material was purified by column chromatography to afford the desired product.

**(2-Iodo-1-phenylpropoxy)dimethyl(trimethylsilyl)ethylsilane (7f)** was synthesized from iodohydrin **3f** (0.30 g, 1.1 mmol, 1.0 equiv), aminosilane **s2** (0.26 g, 1.3 mmol, 1.1 equiv), methyl iodide (0.16 mL, 2.5 mmol, 2.3 equiv), and imidazole (83 mg, 1.2 mmol, 1.1 equiv) in dry CH_2_Cl_2_ (7.5 mL) according to Procedure F. The crude was purified by column chromatography (CH_2_Cl_2_/pentane 10%) to afford **7f** (0.24 g, 0.57 mmol, 50%) as a colorless oil. **R_*f*_** 0.44 (CH_2_Cl_2_/pentane 10%); IR (liquid, ν_max_/cm^–1^) 2960, 1452, 1251, 1131, 1045, 829, 789, 752, 699; ^1^H NMR (400 MHz, CDCl_3_) δ 7.40–7.26 (5H, m, Ar*H*), 5.01 (1H, d, *J* = 5.1 Hz, C*H*O), 4.36 (1H, qd, *J* = 6.9, 5.0 Hz, C*H*I), 1.80 (3H, d, *J* = 6.9 Hz, CHIC*H*_3_), 0.32 (3H, s, Si(C*H*_3_)_2_), 0.13 (9H, s, 3 × Si(C*H*_3_)_3_), 0.12 (3H, s, Si(C*H*_3_)_2_); ^13^C{^1^H} NMR (CDCl_3_, 101 MHz) δ 141.5, 128.1 (2C), 127.9, 127.1 (2C), 115.5, 110.6, 80.7, 34.1, 22.7, 0.9, 0.6, −0.1 (3C); HRMS (APCI) calc. for C_16_H_26_IOSi_2_ ([M + H]^+^) 417.0561, found 417.0560.

**((1-Iodohexan-2-yl)oxy)dimethyl(trimethylsilyl)ethynylsilane (7k)** was synthesized from iodohydrin **3k** (0.29 g, 1.3 mmol, 1.0 equiv), aminosilane **s2** (0.26 g, 1.3 mmol, 1.0 equiv), methyl iodide (0.16 mL, 2.5 mmol, 1.9 equiv), and imidazole (82 mg, 1.2 mmol, 0.95 equiv) in dry CH_2_Cl_2_ (7.5 mL) according to Procedure F. The crude was purified by column chromatography (CH_2_Cl_2_/pentane 5%) to afford **7k** (91 mg, 0.24 mmol, 19%) as a colorless oil. **R_*f*_** 0.41 (CH_2_Cl_2_/pentane 5%); IR (liquid, ν_max_/cm^–1^) 2960, 2862, 1251, 1036, 831, 792, 756; ^1^H NMR (400 MHz, CDCl_3_) δ 3.76 (1H, ddt, *J* = 7.5, 6.0, 4.6 Hz, C*H*O), 3.33–3.20 (2H, m, C*H*_2_I), 1.69 (1H, dddd, *J* = 13.9, 9.3, 5.4, 2.9 Hz, C*H*_2_CHO), 1.62–1.49 (1H, m, C*H*_2_CHO), 1.43–1.22 (4H, m, 4 × C*H*_2_), 0.91 (3H, t, *J* = 7.0 Hz, C*H*_3_), 0.30 (3H, s, Si(C*H*_3_)), 0.27 (3H, s, Si(C*H*_3_)), 0.19 (9H, s, 3 × Si(C*H*_3_)); ^13^C{^1^H} NMR (CDCl_3_, 101 MHz) δ 115.2, 111.0, 72.9, 36.3, 27.6, 22.7, 14.2, 13.5, 0.90, 0.86, −0.1 (3C); HRMS (APCI) calc. for C_13_H_28_IOSi_2_ ([M + H]^+^) 383.0718, found 383.0717.

**((1-Iodo-3-isopropoxy-1-phenylpropan-2-yl)oxy)dimethyl(trimethylsilyl)ethynylsilane (7s)** was synthesized from iodohydrin **3 s** (0.31 g, 0.96 mmol, 1.0 equiv), aminosilane **s2** (0.26 g, 1.2 mmol, 1.2 equiv), methyl iodide (0.14 mL, 2.3 mmol, 2.4 equiv), and imidazole (70 mg, 1.0 mmol, 1.1 equiv) in dry CH_2_Cl_2_ (4.2 mL) according to Procedure F. The crude was purified by column chromatography (Et_2_O/pentane 5%) to afford an inseparable 62:38 diastereomers mixture of **7s** (0.17 g, 0.36 mmol, 37%) as a light-yellow oil. *Note: the NMR data were extracted from the analysis of the inseparable mixture.***R_*f*_** 0.48 (CH_2_Cl_2_/pentane 5%); IR (liquid, ν_max_/cm^–1^) 2971, 1251, 1117, 1032, 825, 792, 755, 697; ^1^H NMR (400 MHz, CDCl_3_, *major*) δ 7.47–7.43 (2H, m, Ar*H*), 7.30–7.18 (3H, m, Ar*H*), 5.32 (1H, d, *J* = 5.6 Hz, C*H*I), 4.44 (1H, q, *J* = 5.5 Hz, C*H*OSi), 3.59 (1H, m, C*H*_2_OCH(CH_3_)_2_), 3.51 (1H, m, C*H*(CH_3_)_2_), 3.40 (1H, m, C*H*_2_OCH(CH_3_)_2_), 1.24–1.07 (6H, m, CH(C*H*_3_)_2_), 0.31–0.14 (15H, m, 5 × Si(C*H*_3_)); ^13^C{^1^H} NMR (CDCl_3_, 101 MHz, *major*) δ 140.7, 129.6 (2C), 129.0, 128.2 (2C), 115.1, 111.3, 77.4, 72.2, 69.7, 34.1, 22.2, 1.3, 0.7, −0.1 (3C); ^1^H NMR (400 MHz, CDCl_3_, *minor*) δ 7.47–7.43 (2H, m, Ar*H*), 7.30–7.18 (3H, m, Ar*H*), 5.32 (1H, d, *J* = 5.6 Hz, C*H*I), 3.70 (1H, q, *J* = 5.3 Hz, C*H*OSi), 3.51 (1H, m, C*H*(CH_3_)_2_), 3.40 (2H, m, C*H*_2_OCH(CH_3_)_2_), 1.24–1.07 (6H, m, CH(C*H*_3_)_2_), 0.31–0.14 (15H, m, 5 × Si(C*H*_3_)); ^13^C{^1^H} NMR (CDCl_3_, 101 MHz, *minor*) δ 141.7, 128.4 (2C), 128.0, 127.9 (2C), 115.0, 111.4, 76.6, 72.2, 70.3, 39.0, 25.5, 1.3, 0.6, −0.1 (3C); HRMS (APCI) calc. for C_19_H_35_INO_2_Si_2_ ([M + NH_4_]^+^) 492.1246, found 492.1246.

**2-Methyl-1-phenyl-4-(trimethylsilyl)but-3-yn-1-ol (8f)** was synthesized from **7f** (0.13 g, 0.30 mmol, 1.0 equiv), [Ir(dtbbpy)(ppy)_2_]PF_6_ (6.3 mg, 6.9 μmol, 2.3 mol %), Bu_3_N (0.14 mL, 0.59 mmol, 2.0 equiv) in MeCN/MeOH 9:1 (3 mL) and HCl 1.25 M in MeOH (0.50 mL, 0.63 mmol, 2.1 equiv) according to Procedure E. The crude was purified by column chromatography (IPA/CH_2_Cl_2_ 0–0.2%) to afford **8f** (45 mg, 0.19 mmol, 65%) as a colorless oil. ^1^H NMR (400 MHz, CDCl_3_) δ 7.39–7.27 (5H, m, Ar*H*), 4.48 (1H, dd, *J* = 7.2, 3.6 Hz, C*H*OH), 2.80 (1H, p, *J* = 7.0 Hz, C*H*(CC)), 2.66 (1H, d, *J* = 3.6 Hz, O*H*), 1.08 (3H, d, *J* = 7.0 Hz, CHC*H*_3_), 0.18 (9H, s, Si(C*H*_3_)_3_); ^13^C{^1^H} NMR (CDCl_3_, 101 MHz) δ 141.4, 128.3 (2C), 128.1, 126.9 (2C), 107.8, 88.2, 77.5, 36.6, 17.3, 0.2 (3C). Data consistent with literature values.^57^

**1-(Trimethylsilyl)oct-1-yn-4-ol (8k)** was synthesized from **7k** (72 mg, 0.19 mmol, 1.0 equiv), [Ir(dtbbpy)(ppy)_2_]PF_6_ (4.3 mg, 4.7 μmol, 2.5 mol %), Bu_3_N (80 μL, 0.34 mmol, 1.8 equiv) in MeCN/MeOH 9:1 (1.8 mL), and HCl 1.25 M in MeOH (0.30 mL, 0.38 mmol, 2.0 equiv) according to Procedure E. The crude was purified by column chromatography (IPA/CH_2_Cl_2_ 0–0.5%) to afford **8k** (25 mg, 0.13 mmol, 68%) as a colorless oil. ^1^H NMR (400 MHz, CDCl_3_) δ 3.78–3.66 (1H, m, C*H*OH), 2.45 (1H, dd, *J* = 16.8, 4.7 Hz, C*H*_2_CCSi(CH_3_)_3_), 2.34 (1H, dd, *J* = 16.8, 6.9 Hz, C*H*_2_CCSi(CH_3_)_3_), 1.97 (1H, d, *J* = 4.8 Hz, O*H*), 1.52 (2H, m, C*H*_2_CHOH), 1.46–1.23 (4H, m, 4 × C*H*_2_), 0.90 (3H, t, *J* = 7.1 Hz), 0.15 (9H, s, Si(C*H*_3_)_3_); ^13^C{^1^H} NMR (CDCl_3_, 101 MHz) δ 103.5, 87.7, 70.0, 36.0, 29.0, 27.9, 22.7, 14.1, 0.2 (3C). Data consistent with literature values.^58^

**1-Isopropoxy-3-phenyl-5-(trimethylsilylpent-4-yn-2-ol (8s)** was synthesized from **7s** (0.14 g, 0.30 mmol, 1.0 equiv), [Ir(dtbbpy)(ppy)_2_]PF_6_ (6.3 mg, 6.9 μmol, 2.3 mol %), Bu_3_N (0.14 mL, 0.59 mmol, 2.0 equiv) in MeCN/MeOH 9:1 (3 mL), and HCl 1.25 M in MeOH (0.50 mL, 0.63 mmol, 2.1 equiv) according to Procedure E. The crude was purified by column chromatography (IPA/CH_2_Cl_2_ 0.5–1%). A short second column chromatography (Et_2_O/pentane 70%) afforded **8s** (23 mg, 78 μmol, 26%) as a colorless oil. **R_*f*_** 0.38 (Et_2_O/pentane 70%); IR (thin film, ν_max_/cm^–1^) 2972, 2172, 1740, 1371, 1250, 1130, 1076, 842, 760, 701; ^1^H NMR (400 MHz, CDCl_3_) δ 7.43–7.37 (2H, m, Ar*H*), 7.37–7.30 (2H, m, Ar*H*), 7.27 (1H, qd, *J* = 5.0, 3.9, 1.6 Hz, Ar*H*), 4.02 (1H, d, *J* = 5.5 Hz, C*H*CC), 3.87 (1H, p, *J* = 5.4 Hz, C*H*OH), 3.63–3.55 (1H, m, C*H*(CH_3_)_2_), 3.55–3.48 (1H, m, C*H*_2_), 3.32 (1H, dd, *J* = 9.6, 5.7 Hz, C*H*_2_), 2.36 (1H, d, *J* = 5.2 Hz, O*H*), 1.16 (6H, dd, *J* = 6.1, 4.1 Hz, (C*H*_3_)_2_), 0.20 (9H, s, Si(C*H*_3_)_3_); ^13^C{^1^H} NMR (CDCl_3_, 101 MHz) δ 138.1, 128.6 (2C), 128.5 (2C), 127.4, 104.6, 89.7, 74.1, 72.3, 69.2, 42.8, 22.2, 22.1, 0.2 (3C); HRMS (APCI) calc. for C_17_H_27_O_2_Si ([M + H]^+^) 291.1775, found 291.1778.

**1-(Allyloxy)-2-iodo-2,3-dihydro-1*H*-indene (9)** was synthesized according to a literature procedure.^59^ Indene (0.50 mL, 3.9 mmol, 1.0 equiv) and allyl alcohol (0.30 mL, 4.4 mmol, 1.1 equiv) were added to a solution of NIS (0.96 g, 4.3 mmol, 1.1 equiv) in dry CH_2_Cl_2_ (3.9 mL) at −78 °C under Ar. The reaction mixture was stirred 5 min at this temperature, before removing the cold bath. It was further stirred at room temperature overnight. The mixture was partially reduced in vacuo, and an aqueous 0.1 M Na_2_S_2_O_3_ solution (10 mL) was added to the residue. The mixture was extracted with CH_2_Cl_2_ (3 × 8 mL), and the combined organic layers were dried over anhydrous Na_2_SO_4_ before being concentrated in vacuo. The crude was purified by column chromatography (Et_2_O/pentane 4%) to afford **9** (0.39 g, 1.3 mmol, 33%) as a light-yellow oil. **R_*f*_** 0.39 (Et_2_O/pentane 5%); IR (liquid, ν_max_/cm^–1^) 3027, 3020, 2972, 2853, 1740, 1461, 1426, 1368, 1219, 1054, 923, 748; ^1^H NMR (400 MHz, CDCl_3_) δ 7.48–7.36 (1H, m, Ar*H*), 7.35–7.16 (3H, m, Ar*H*), 5.98 (1H, ddt, *J* = 17.3, 10.4, 5.7 Hz, OCH_2_C*H*CH_2_), 5.36 (1H, dq, *J* = 17.2, 1.6 Hz, OCH_2_CHC*H*_2_), 5.29–5.16 (2H, m, C*H*O, OCH_2_CHC*H*_2_), 4.48 (1H, ddd, *J* = 6.9, 4.9, 3.7 Hz, C*H*I), 4.32 (1H, ddt, *J* = 12.6, 5.5, 1.5 Hz, OC*H*_2_), 4.24 (1H, ddt, *J* = 12.7, 5.8, 1.4 Hz, OC*H*_2_), 3.74 (1H, dd, *J* = 16.9, 6.9 Hz, C*H*_2_), 3.29 (1H, dd, *J* = 16.9, 4.8 Hz, C*H*_2_); ^13^C{^1^H} NMR (CDCl_3_, 101 MHz) δ 141.6, 140.6, 134.8, 129.2, 127.3, 125.3, 124.8, 117.8, 91.4, 71.2, 43.7, 26.5; HRMS (ESI) calc. for C_12_H_13_OINa ([M + Na]^+^) 322.9903, found 322.9903.

**3-(Iodomethyl)-3,3a,4,8b-tetrahydro-2*H*-indeno[1,2-*b*]furan (10)** was synthesized from **9** (90 mg, 0.30 mmol, 1.0 equiv), [Ir(dtbbpy)(ppy)_2_]PF_6_ (5.8 mg, 6.3 μmol, 2.1 mol %), and Bu_3_N (0.14 mL, 0.59 mmol, 2.0 equiv) in MeCN/MeOH 9:1 (3 mL) according to Procedure D. The crude was redissolved in EtOAc (5 mL) and was transferred into a separatory funnel containing an aqueous 1 M HCl solution (20 mL). The aqueous layer was extracted with EtOAc (3 × 5 mL), and the combined organic layers were washed with an aqueous 2 M HCl solution, a saturated NaHCO_3_ solution, and brine. The organic layer was dried over anhydrous Na_2_SO_4_ and concentrated in vacuo. The crude was purified by column chromatography (Et_2_O/pentane 20%) to afford an inseparable 4:1 mixture of **10** (44 mg, 0.15 mmol, 48%) and **11** (6.3 mg, 36 μmol, 12%). *Note: the NMR data were extracted from the analysis of the inseparable mixture.***R_*f*_** 0.33 (Et_2_O/pentane 20%); IR (liquid, ν_max_/cm^–1^) 3024, 2922, 2848, 1740, 1459, 1368, 1219, 1184, 1180, 1021, 749; ^1^H NMR (400 MHz, CDCl_3_) δ 7.49–7.33 (1H, m, Ar*H*), 7.33–7.10 (3H, m, Ar*H*), 5.57 (1H, d, *J* = 6.7 Hz, C*H*O), 4.03 (1H, dd, *J* = 8.5, 6.7 Hz, OC*H*_2_), 3.32–3.18 (2H, m, OC*H*_2_, OCH_2_C*H*CH_2_I), 3.18–3.05 (2H, m, C*H*_2_I), 3.05–2.82 (3H, m, 2 × C*H*_2_, C*H*); ^13^C{^1^H} NMR (CDCl_3_, 101 MHz) δ 143.3, 142.0, 129.1, 127.3, 125.5, 124.6, 88.2, 71.8, 46.7, 45.9, 30.7, 2.3; HRMS (ESI) calc. for C_12_H_13_OINa ([M + Na]^+^) 322.9903, found 322.9904.

**3-Methyl-3,3a,4,8b-tetrahydro-2*H*-indeno[1,2-*b*]furan (11)** was synthesized from **9** (90 mg, 0.30 mmol, 1.0 equiv), [Ir(dtbbpy)(ppy)_2_]PF_6_ (5.8 mg, 6.3 μmol, 2.1 mol %), and Bu_3_N (0.14 mL, 0.59 mmol, 2.0 equiv) in MeCN/MeOH 9:1 (3 mL) according to Procedure D. The crude was redissolved in EtOAc (5 mL) and was transferred into a separatory funnel containing an aqueous 1 M HCl solution (20 mL). The aqueous layer was extracted with EtOAc (3 × 5 mL), and the combined organic layers were washed with an aqueous 2 M HCl solution, a saturated NaHCO_3_ solution, and brine. The organic layer was dried over anhydrous Na_2_SO_4_ and concentrated in vacuo. The crude was purified by column chromatography (Et_2_O/pentane 20%) to afford an inseparable 4:1 mixture of **10** (44 mg, 0.15 mmol, 48%) and **11** (6.3 mg, 36 μmol, 12%). *Note: the NMR data were extracted from the analysis of the inseparable mixture.***R_*f*_** 0.33 (Et_2_O/pentane 20%); IR (liquid, ν_max_/cm^–1^) 3024, 2922, 2848, 1740, 1459, 1368, 1219, 1184, 1180, 1021, 749; ^1^H NMR (400 MHz, CDCl_3_) δ 7.46–7.35 (1H, m, Ar*H*), 7.34–7.11 (3H, m, Ar*H*), 5.49 (1H, d, *J* = 6.7 Hz, C*H*O), 3.90 (1H, dd, *J* = 8.3, 6.9 Hz, OC*H*_2_), 3.32–3.18 (1H, m, C*H*_2_), 3.18–3.05 (2H, m, OC*H*_2_, C*H*), 3.05–2.80 (1H, m, C*H*_2_), 2.57–2.41 (1H, m, C*H*CH_3_), 1.01 (3H, d, *J* = 6.9 Hz, C*H*_3_); ^13^C{^1^H} NMR (CDCl_3_, 101 MHz) δ 144.2, 142.9, 128.7, 127.0, 125.5, 124.5, 88.1, 73.3, 45.6, 37.1, 31.2, 12.3; HRMS (ESI) calc. for C_12_H_14_ONa ([M + Na]^+^) 197.0937, found 197.0937.

**1,1,3,3-Tetramethyl-1,3-bis((2-vinyl-2,3-dihydro-1*H*-inden-1-yl)oxy)disiloxane (13a)** was synthesized according to a modified Procedure D. A mixture of vinyl silane **4a** (0.10 g, 0.29 mmol, 1.0 equiv), [Ir(dtbbpy)(ppy)_2_]PF_6_ (6.7 mg, 7.4 μmol, 2.5 mol %), and Bu_3_N (0.14 mL, 0.59 mmol, 2.0 equiv) in dry MeCN (3 mL) was prepared under Ar. The mixture was degassed 5 min by Ar sparging before placing the reaction vial 1 cm away from the light source. A fan was placed on top of the setup to keep the reaction environment at room temperature. The reaction mixture was irradiated with blue LED light until full consumption of the substrate as indicated by TLC. The mixture was reduced in vacuo, and the crude was purified by column chromatography (Et_2_O/pentane 1%) to afford **12a** (43 mg, 95 μmol, 64%) as a colorless oil. **R_*f*_** 0.38 (Et_2_O/pentane 3%); IR (liquid, ν_max_/cm^–1^) 3076, 2960, 2906, 1258, 1113, 1046, 1019, 985, 916, 882, 844, 795, 751, 721; ^1^H NMR (400 MHz, CD_3_CN) δ 7.32 (2H, m, Ar*H*), 7.26–7.12 (6H, m, Ar*H*), 6.00 (2H, m, 2 × C*H*CH_2_), 5.25 (2H, m, 2 × C*H*O), 5.13 (2H, m, 2 × CHC*H*_2_), 5.06 (2H, m, 2 × CHC*H*_2_), 3.07–2.95 (2H, m, 2 × C*H*(CHCH_2_)), 2.95–2.84 (4H, m, 2 × C*H*_2_), 0.12 (12H, m, 2 × Si(C*H*_3_)_2_); ^13^C{^1^H} NMR (CD_3_CN, 101 MHz) δ 145.7 (2C), 143.7 (2C), 139.6 (2C), 129.0 (2C), 127.3 (2C), 125.7 (2C), 125.6 (2C), 116.0 (2C), 78.2 (2C), 50.8 (2C), 36.7 (2C), 0.1 (2C), 0.0 (2C); HRMS (ESI) calc. orf C_26_H_34_O_3_Si_2_Na ([M + Na]^+^) 473.1939, found 473.1941.

## Data Availability

The data underlying this study are available in the published article, in its Supporting Information, and openly available in DataverseNO at https://doi.org/10.18710/U5RNGE.^60^

## Supporting Information Available

Additional experimental details, characterization of compounds **1**–**4v**, and ^1^H and ^13^C NMR spectra for all compounds (PDF)
