# Supplementary material for: Radical Group Transfer of Vinyl and Alkynyl Silanes Driven by Photoredox Catalysis
Source: J Org Chem. 2023 Aug 15;88(17):12451–63. doi: 10.1021/acs.joc.3c01213 (PMC10476183; doi:10.1021/acs.joc.3c01213)
Supplement: Supplementary file 1 — jo3c01213_si_001.pdf [file jo3c01213_si_001.pdf]

Supporting Information for:

Radical Group Transfer of Vinyl and Alkynyl Silanes Driven by Photoredox Catalysis

Floriane Baussière and Marius M. Haugland*

Department of Chemistry, UiT The Arctic University of Norway, 9037 Tromsø, Norway

* marius.m.haugland@uit.no

Contents

1. **Stability experiments** S2
2. **Control experiments – radical reaction** S2
3. **^{1}H NMR monitoring of the radical reaction initiated with BEt_3** S3
4. **^{1}H NMR monitoring of the reaction, full spectra** S3
5. **^{1}H NMR analysis of the fate of the iminium ion** S4
6. **Experimental methods** S5
7. **Photocatalysts synthesis** S5
8. **Silanes synthesis** S6
9. **Olefins 1e, 1i and 1s synthesis** S7
10. **Epoxides 2q, 2r and 2s synthesis** S8
11. **Iodohydrins 3a-v synthesis** S10
    - **11.1 Procedure A** S10
    - **11.2 Procedure B** S10
12. **Vinyl silanes 4a-v synthesis** S18
    - **12.1 Procedure C** S18
13. **References** S28
14. **NMRs** S30

1. Stability experiments

Stability experiments on **4k**, **4n** and **5n** were performed as follow. Mixtures of the investigated compounds (0.16 mmol, 1.0 equiv), [Ir(dtbbpy)(ppy)$_2$]PF$_6$ (see table) and Bu$_3$N (0.32 mmol, 2.0 equiv.) in MeCN-d$_3$/CD$_3$OD 9:1 (0.1 M with respect to the compound) were prepared under Ar. The reaction mixtures were degassed 5 min by Ar sparging, before transferring precise aliquots (0.40 mL) into NMR tubes together with precise volumes of ethynyl carbonate in MeCN-d$_3$ solution (0.10 mL). Quantitative ^{1}H NMR of each mixture were recorded. The mixtures in vials were irradiated by blue LED light for 24 h, before transferring precise aliquots (0.40 mL) into NMR tubes together with precise volumes of ethynyl carbonate in MeCN-d$_3$ solution (0.10 mL) again. Quantitative ^{1}H NMR of each mixture were recorded. For each NMR, the integral of the ethynyl carbonate peak was set to 4.00. The integral of one proton of **4k**, **4n** and **5n** was measured and reported in Table S1.

Table S1. Vinyl silanes 4k/n and siloxane 5n stability experiments

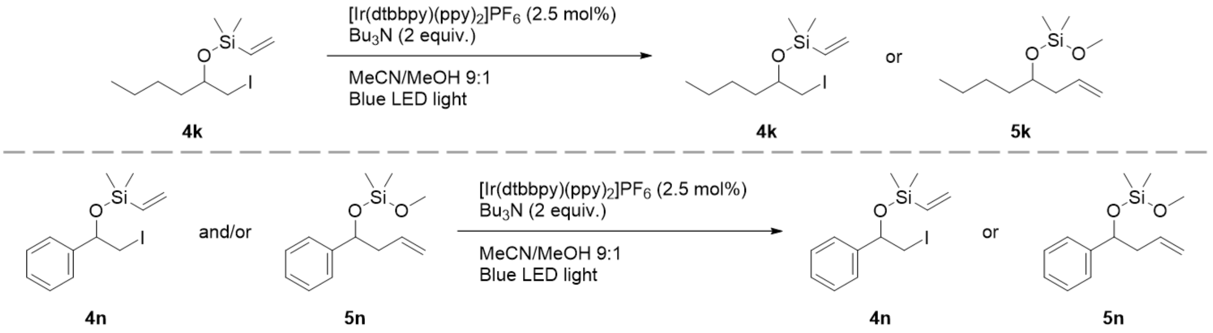

		integral of one proton				
		at 0 h		at 24 h		
in the reaction mixture before blue LED light irradiation	[Ir(dtbbpy)(ppy)$_2$]PF$_6$	**4k/n**	**5k/n**	**4k/n**	**5k/n**	yield (%)[d]
**4k**[a]	no photocatalyst	1.09	-	1.00	-	92
**4k**[b]	2.5 mol%	1.18	-	-	0.47	40
**4n**[c]	no photocatalyst	1.00	-	0.91	-	91
**4n**	2.5 mol%	1.32	-	-	0.53	40
1:1 **4n/5n** mixture[c]	2.5 mol%	1.62	1.38	-	1.97	66
**5n**[c]	2.5 mol%	-	2.79	-	2.38	85

[a]Reaction scale: 21 µmol. [b]Reaction scale: 0.38 mmol. [c]Reaction scale: 0.16 mmol. [d]Stability yields were calculated from the integrals of **4k**, **4n** and **5n** before and after 24 h of reaction.

2. Control experiments – radical reaction

Table S2. Photoredox-catalyzed radical reaction control experiments[a]

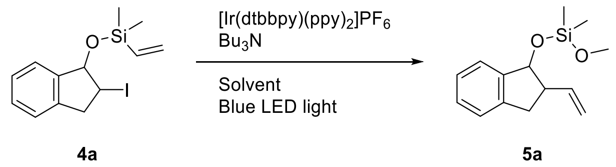

entry	[Ir(dtbbpy)(ppy)$_2$]PF$_6$	Bu$_3$N	solvent	blue LED light	time	yield (%)[b]
1	no photocatalyst	2 equiv.	MeCN/MeOH 9:1	yes	0.5 h	0
2	2.5 mol%	no base	MeCN/MeOH 9:1	yes	0.5 h	0
3	2.5 mol%	2 equiv.	MeCN/MeOH 9:1	no light	0.5 h	0

[a]Reaction scale: 0.10 mmol. [b]Yield determined by ^{1}H NMR integral comparison between substrate and product.

3. ^{1}H NMR monitoring of the radical reaction initiated with BEt_3

A mixture of substrate **4a** (21 mg, 62 µmol, 1.0 equiv.) and 1 M solution of BEt_3 in hexenes (12 µL, 12 µmol, 0.2 equiv.) in benzene-d_6 (0.6 mL) was prepared in triplicates in gas-tight NMR tubes under Ar. Air was injected to initiate the radical reaction before sealing the tubes. The mixtures were shaken, and the reaction was monitored by ^{1}H NMR. TBAF (1 M in THF, 0.12 mL, 0.12 mmol, 2.0 equiv.), MeOH (4.0 µL, 0.10 mmol, 2.0 equiv.) and Bu_3N (24 µL, 0.10 mmol, 2.0 equiv.) were added to the different tubes, the mixtures were shaken, and the reactions were monitored by ^{1}H NMR. The appearance of the vinyl proton peak at approximately 6 ppm indicates that the ring opened.

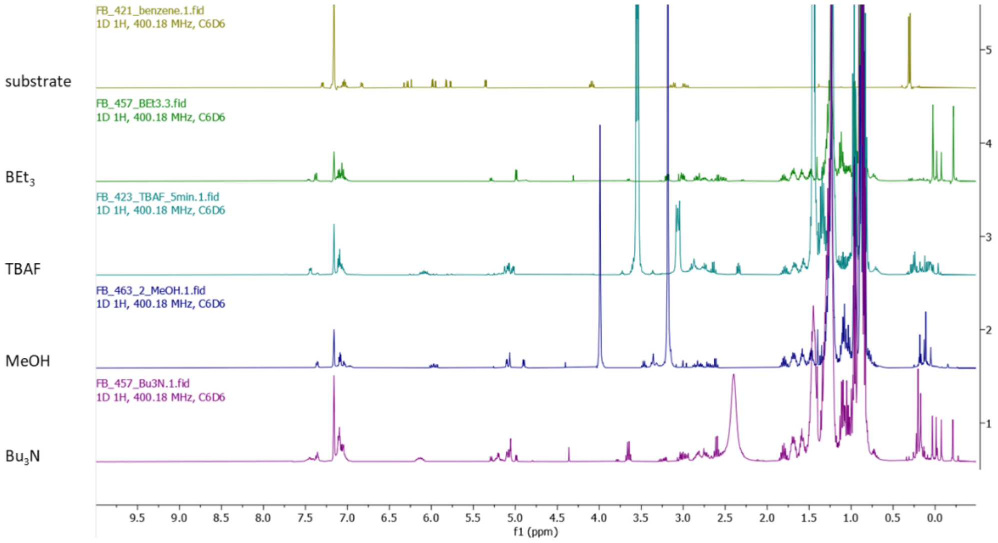

Figure S1. NMR following of the radical reaction initiated by BEt_3 in benzene-d_6.

4. ^{1}H NMR monitoring of the reaction, full spectra

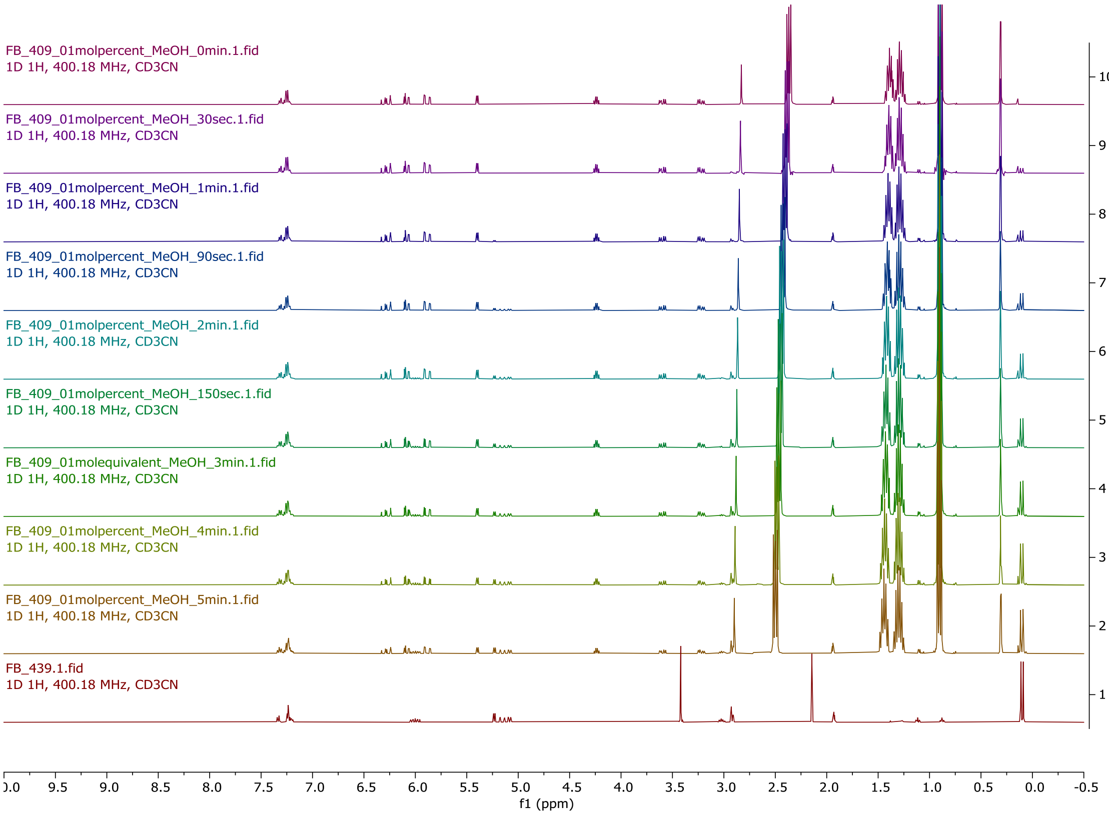

Figure S2. NMR following of the radical reaction in MeCN-d_3/CD_3OD 9:1.

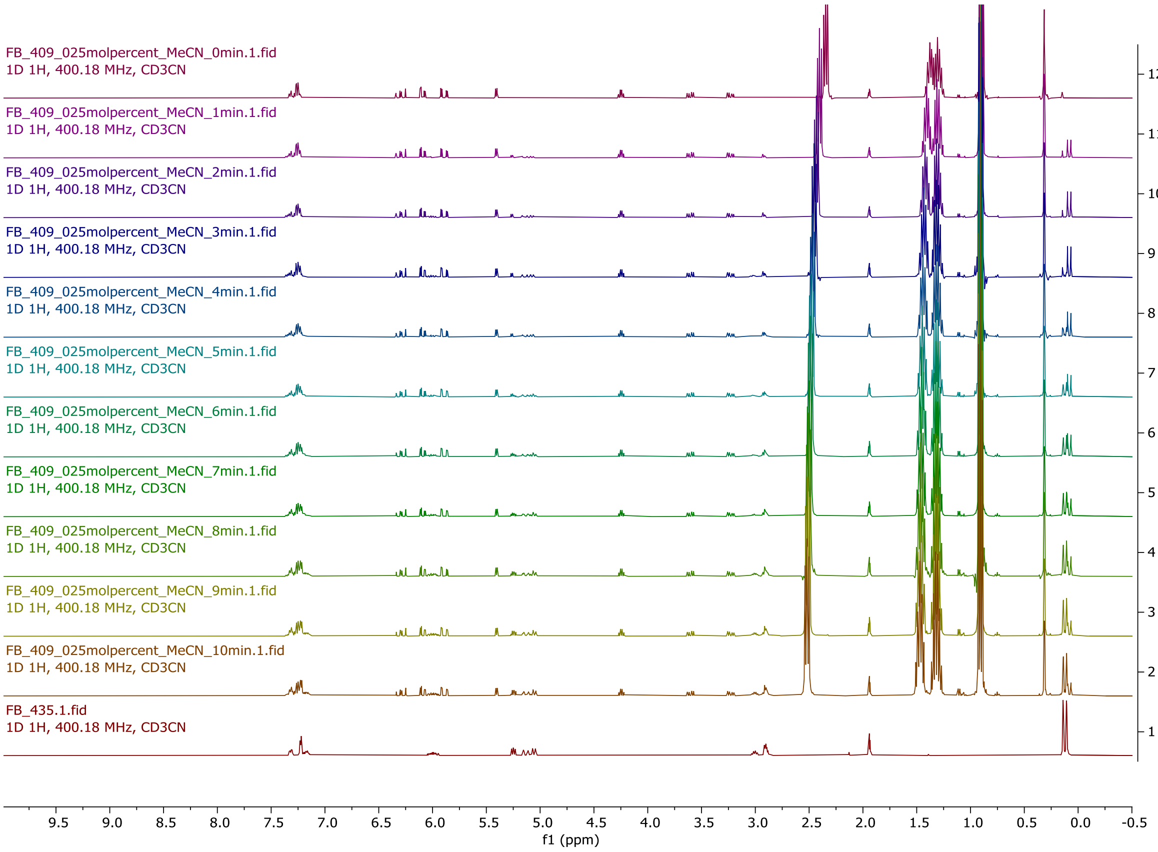

Figure S3. NMR following of the radical reaction in MeCN-d$_3$/CD$_3$OD 9:1.

5. ^{1}H NMR analysis of the fate of the iminium ion

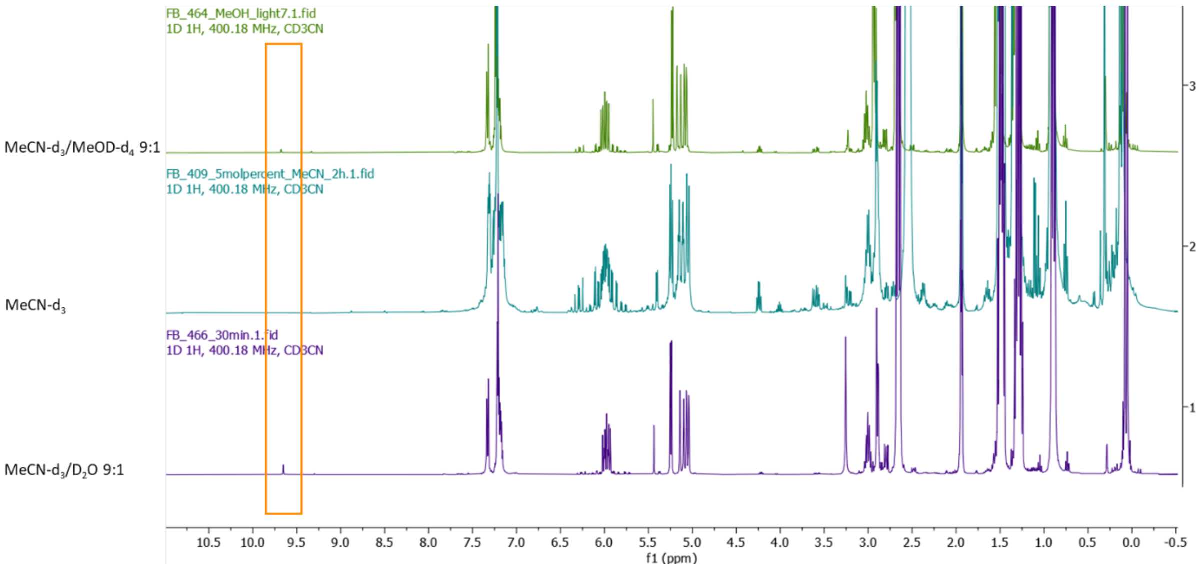

Figure S4. ^{1}H NMR analysis of the crude product of photoredox reactions of substrate **4a** performed in different solvents. Reactions were performed in deuterated solvents in gas-tight NMR tubes by direct irradiation. Reaction progress was monitored by ^{1}H NMR without opening the sealed NMR tubes. At full conversion, the amounts of butanal formed (relative to **6a** or **13a**) were <5% (MeCN-d_3/MeOD-d_4), trace (MeCN-d_3) and 8% (MeCN-d_3/D_2O).

6. Experimental methods

All reagents purchased from Acros, Alfa, Sigma-Aldrich, TCI and VWR were used as supplied. All air and/or water sensitive reactions were carried out under an atmosphere of argon in flame-dried glassware using standard Schlenk techniques. Anhydrous solvents were dried by pre-storing over activated 3 Å (MeOH) or 4 Å (all other solvents) molecular sieves and purged by argon sparging. Blue light irradiation was performed with RGB LED strips (IP20, 220-240 V, 5.4 W). Reactions were monitored by thin layer chromatography (TLC) on pre-coated aluminum-based plates (TLC Silica gel 60 F_{254}, Supelco). The plates were developed under UV irradiation (254 nm) or with vanillin or $KMnO_4$ staining and subsequent heating. Column chromatography was performed with silica gel (Silica gel 60, irregular 40-63 µm for flash chromatography, VWR Chemicals). ^{1}H NMR spectra were recorded at room temperature on a 400 MHz Bruker 9.4 Tesla Avance III HD system equipped with a SmartProbe (broad band). ^{13}C NMR spectra (^{1}H decoupled) were recorded at room temperature on the same machine operating at 101 MHz. All chemical shifts (δ) are reported in parts per million (ppm) with internal reference to residual protons in $CDCl_3$ (δ 7.26 or 77.16) or MeCN-d_3 (δ 1.94 or 118.26). Coupling constants (*J*) are given in Hz with an accuracy of 0.1 Hz. Infrared (IR) spectra were recorded as thin films or liquids on an Agilent Technologies Cary 630 FTIR 318 spectrometer. Wavelength of maximum absorbance (ν_{max}) are reported in wavenumbers (cm^{-1}). Only selections of characteristic resonances are reported. High-resolution mass spectra (HRMS) were recorded by direct injection of the compounds as solutions in MeOH on a Thermo Scientific Orbitrap Exploris 120 Mass spectrometer, using dual electrospray ionization (ESI) and atmospheric pressure chemical ionization (APCI) probes.

7. Photocatalysts synthesis

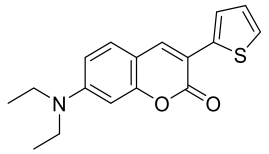

7-(Diethylamino)-3-(thiophen-2-yl)-2H-chromen-2-one was synthesized according to a procedure reported by Gualandi *et al.*[1] Triethylamine (2.0 mL, 14 mmol, 1.0 equiv.) was added to a mixture of 4-(diethylamino)salicylaldehyde (1.8 g, 9.3 mmol, 1.2 equiv.) and 2-thiopheneacetic acid (1.1 g, 7.6 mmol, 1.0 equiv.) in acetic anhydride (27 mL) under Ar. The reaction flask was topped with a condenser and the reaction mixture was refluxed for 3 h. The reaction mixture was allowed to reach room temperature before being cooled in an ice-water bath. The mixture was diluted with water (50 mL) and transferred into a separatory funnel where it was extracted with EtOAc (3 × 30 mL). The combined organic layers were washed with a saturated aqueous $NaHCO_3$ solution (7 × 50 mL), dried over anhydrous Na_2SO_4 and reduced *in vacuo*. The dark brown residue was purified by column chromatography (EtOAc/heptane 0-30%) to afford 7-(diethylamino)-3-(thiophen-2-yl)-2*H*-chromen-2-one as a yellow solid (0.79 g, 2.7 mmol, 35%).

^{1}H NMR (400 MHz, $CDCl_3$) δ 7.88 (1H, s, C*H*), 7.66 (1H, dd, *J* = 3.7, 1.2 Hz, SC*H*), 7.33 (1H, d, *J* = 8.8 Hz, NCCHC*H*C), 7.31 (1H, dd, *J* = 5.1, 1.2 Hz, SCC*H*), 7.08 (1H, dd, *J* = 5.1, 3.7 Hz, SCHC*H*), 6.61 (1H, dd, *J* = 8.8, 2.5 Hz, NCC*H*CHC), 6.53 (1H, d, *J* = 2.5 Hz, NCC*H*C), 3.43 (4H, q, *J* = 7.1 Hz, N(CH_2CH_3)$_2$), 1.22 (6H, t, *J* = 7.1 Hz, N(CH_2CH_3)$_2$); **^{13}C{^{1}H} NMR** ($CDCl_3$, 101 MHz) δ 160.7, 155.7, 150.6, 137.6, 137.0, 129.0, 127.4, 125.7, 125.0, 114.9, 109.4, 108.9, 97.3, 45.0 (2C), 12.6 (2C).

Data consistent with literature values.[1]

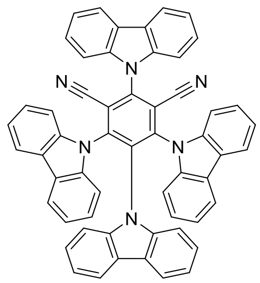

4CzIPN was synthesized according to a procedure reported by Filippini *et al.*[2] A solution of carbazole (1.7 g, 10 mmol, 5.0 equiv.) in dry THF (16 mL) was slowly added to a stirred solution of sodium hydride (60% dispersion in mineral oil, 0.62 g, 1.5 mmol, 7.6 equiv.) in dry THF (25 mL) under Ar. The mixture was stirred 30 min at room temperature before slowly adding a solution of tetrafluoroisophthalonitrile (0.41 g, 2.0 mmol, 1.0 equiv.) in dry THF (3 mL). The reaction mixture was stirred overnight at room temperature. Distilled water (0.2 mL) was added to quench the excess sodium hydride and the mixture was concentrated *in vacuo*. The crude was purified by column chromatography (CH_2Cl_2/pentane 25-80%) to afford 4CzIPN (1.4 g, 1.8 mmol, 86%) as a yellow solid.

^{1}H NMR (400 MHz, $CDCl_3$) δ 8.23 (2H, dt, *J* = 7.9, 1.0 Hz), 7.76–7.66 (8H, m), 7.49 (2H, ddd, *J* = 8.0, 6.6, 1.6 Hz), 7.33 (2H, dt, *J* = 7.6, 1.0 Hz), 7.25–7.19 (4H, m), 7.13–7.04 (8H, m), 6.87–6.78 (4H, m), 6.63 (2H, td, *J* = 7.7, 7.2, 1.2 Hz); **^{13}C{^{1}H} NMR** ($CDCl_3$, 101 MHz) δ 145.4, 140.1, 138.3, 137.1, 127.1, 125.9, 125.1, 124.9, 124.7, 124.0, 122.5, 122.1, 121.5, 121.1, 120.6, 119.8, 116.5, 111.8, 110.1, 109.62, 109.56.

Data consistent with literature values.[2]

8. Silanes synthesis

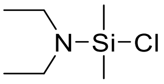

Chloro(diethylamino)dimethylsilane s1 was synthesized according to a procedure reported by Haugland *et al.*[3] A solution of diethylamine (8.6 mL, 83 mmol, 1.0 equiv.) in dry THF (11 mL) was added dropwise over 2 h to a suspension of dichlorodimethylsilane (10 mL, 83 mmol, 1.0 equiv.) and Et_3N (13 mL, 91 mmol, 1.1 equiv.) in dry THF (11 mL) under Ar at 0 °C. The reaction mixture was diluted was diluted with dry THF (35 mL), allowed to reach room temperature, and was stirred for 20 h. Dry pentane (20 mL) was added, and the mixture was filtered through celite under N_2 flow. The filtrate was reduced *in vacuo*. It was purified by vacuum distillation (68 °C under 90 mbar) to afford chloro(diethylamino)dimethylsilane **s1** (3.3 g, 20 mmol, 24%) as a colorless oil.

^{1}H NMR (400 MHz, $CDCl_3$) δ 2.89 (4H, q, *J* = 7.0 Hz, 2 x C*H*$_2$CH$_3$), 1.04 (6H, t, *J* = 7.1 Hz, 2 x CH$_2$C*H*$_3$), 0.46 (6H, s, Si(C*H*$_3$)$_2$); **^{13}C{^{1}H} NMR** ($CDCl_3$, 101 MHz) δ 40.0 (2C), 15.4 (2C), 2.1 (2C).

Data consistent with literature values.[3]

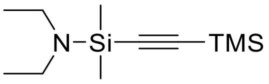

Trimethylsilylethynyl(diethylamino)dimethylsilane s2 was synthesized according to a procedure reported by Haugland *et al.*[3] n-BuLi 2.3 M in hexenes (5.8 mL, 13 mmol, 1.1 equiv.) was added dropwise over 20 min to a solution of TMSA (1.7 mL, 12 mmol, 1.0 equiv.) in dry THF (20 mL) under Ar at -78 °C. After 30 min of stirring at room temperature, the mixture was added dropwise to a solution of chloro(diethylamino)dimethylsilane **s1** (2.0 g, 12 mmol, 1.0 equiv.) in dry THF (1.3 mL) under Ar at -78 °C. The mixture was allowed to slowly reach room temperature while stirring overnight. THF (5 mL) was added to quench the unreacted n-BuLi and the mixture was reduced *in vacuo*. The crude was purified by vacuum distillation to afford trimethylsilylethynyl(diethylamino)dimethylsilane **s2** (1.9 g, 8.5 mmol, 70%) as a colorless oil.

^{1}H NMR (400 MHz, $CDCl_3$) δ 2.85 (4H, q, *J* = 7.0 Hz, 2 x C*H*$_2$CH$_3$), 1.01 (6H, t, *J* = 7.0 Hz, 2 x CH$_2$C*H*$_3$), 0.19 (6H, s, Si(C*H*$_3$)$_2$), 0.16 (9H, s, Si(C*H*$_3$)$_3$); **^{13}C{^{1}H} NMR** ($CDCl_3$, 101 MHz) δ 114.1, 112.6, 40.3 (2C), 15.6 (2C), 0.3 (2C), 0.1 (3C).

Data consistent with literature values.[3]

9. Olefins 1e, 1i and 1s synthesis

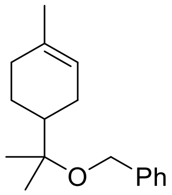

4-(1-(Benzyloxy)-1-methylethyl)-1-methylcyclohexene 1e. A solution of (-)-α-terpinol (0.96 g, 6.2 mmol, 1.0 equiv.) in dry THF (20 mL) was added dropwise over 30 min to a solution of sodium hydride (60% dispersion in mineral oil, 0.50 g, 13 mmol, 2.0 equiv.) in dry THF (40 mL) at 0 °C under Ar. The mixture was stirred 30 min at 0 °C, benzyl bromide (2.2 g, 13 mmol, 2.1 equiv.) was added dropwise and the reaction mixture was stirred 1 h before being allowed to slowly reach room temperature. It was stirred 1 h at room temperature before being stirred at 27 °C for 68 h. Isopropanol (5 mL) was slowly added to quench the residual sodium hydrate and the reaction mixture was concentrated *in vacuo*. The residue was redissolved in water and extracted with CH_2Cl_2 (3 x 30 mL). The combined organic layers were washed with brine, dried over anhydrous Na_2SO_4, filtered and concentrated *in vacuo*. The crude was purified by column chromatography (Et_2O/pentane 0-2%) to afford 4-(1-(benzyloxy)-1-methylethyl)-1-methylcyclohexene **1e** (0.59 g, 2.4 mmol, 39%) as a colorless oil.

^{1}H NMR (400 MHz, $CDCl_3$) δ 7.39–7.18 (5H, m, Ar*H*), 5.47–5.35 (1H, m, C(CH$_3$)C*H*), 4.45 (2H, s, OC*H*$_2$Ar), 2.14–1.73 (6H, m, 2 x C*H*$_2$, C*H*), 1.66 (3H, dd, *J* = 2.2, 1.1 Hz, C(C*H*$_3$)CH), 1.33 (1H, tt, *J* = 12.1, 6.0 Hz, C*H*$_2$), 1.22 (6H, d, *J* = 1.0 Hz, 2 x C*H*$_3$); **^{13}C{^{1}H} NMR** ($CDCl_3$, 101 MHz) δ 140.3, 134.2, 128.4 (2C), 127.3 (2C), 127.1, 121.1, 77.4, 63.2, 42.5, 31.3, 27.1, 24.2, 23.5, 23.2, 22.8.

Data consistent with literature values.[4]

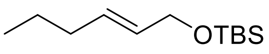

***tert*-Butyl(hex-2-en-1-yloxy)dimethylsilane 1i** was synthesized according to a procedure reported by Wata *et al.*[5] *tert*-Butylchlorodimethylsilane (0.74 g, 4.9 mmol, 1.2 equiv.) was added to a mixture of *trans*-hex-2-en-1-ol (0.49 mL, 4.0 mmol, 1.0 equiv.) and imidazole (0.41 g, 6.1 mmol, 1.5 equiv.) in dry CH_2Cl_2 (4 mL) under Ar at 0 °C. The reaction mixture was stirred overnight at room temperature before being quenched with water (15 mL). The mixture was extracted with CH_2Cl_2 (3 x 5 mL) and the combined organic layers were washed with brine, dried over anhydrous Na_2SO_4, filtered and concentrated *in vacuo*. The crude was purified by column chromatography (EtOAc/heptane 1-4%) to afford *tert*-butyl(hex-2-en-1-yloxy)dimethylsilane **1i** (0.79 g, 3.7 mmol, 93%) as a colorless oil.

^{1}H NMR (400 MHz, $CDCl_3$) δ 5.70–5.58 (1H, m, C*H*CH), 5.58–5.46 (1H, m, CHC*H*), 4.18–4.06 (2H, m, C*H*$_2$O), 2.06–1.95 (2H, m, C*H*$_2$), 1.40 (2H, h, *J* = 7.4 Hz, C*H*$_2$), 0.90 (12H, d, *J* = 3.0 Hz, C*H*$_3$, C(C*H*$_3$)$_3$), 0.07 (6H, s, Si(C*H*$_3$)$_2$); **^{13}C{^{1}H} NMR** ($CDCl_3$, 101 MHz) δ 131.5, 129.4, 64.3, 34.4, 26.2 (3C), 22.5, 18.6, 13.8, -4.9 (2C).

Data consistent with literature values.[6]

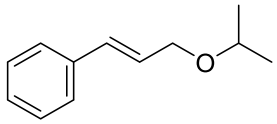

1-Phenyl-3-isopropoxy-1-propene 1s. Dry isopropanol (0.54 mL, 7.0 mmol, 1.0 equiv.) was added dropwise to a solution of sodium hydride (60% dispersion in mineral oil, 0.25 g, 11 mmol, 1.5 equiv.) in dry THF under Ar. The mixture was stirred 30 min at room temperature before being cooled to 0 °C. Cinnamyl bromide (1.6 mL, 11 mmol, 1.5 equiv.) was added dropwise. The mixture was stirred 15 min at 0 °C before being allowed to slowly reach room temperature. It was stirred at room temperature overnight. The reaction was quenched by slowly adding isopropanol (5 mL) and the mixture was reduced *in vacuo*. The residue was redissolved in water and extracted with EtOAc (3 x 20 mL). The combined organic layers were washed with brine, dried over anhydrous Na_2SO_4, filtered and concentrated *in vacuo*. The crude was purified by column chromatography (Et_2O/pentane 0-5%) to afford 1-phenyl-3-isopropoxy-1-propene **1s** (0.79 g, 4.5 mmol, 64%) as a light yellow oil.

^{1}H NMR (400 MHz, $CDCl_3$) δ 7.44–7.18 (5H, m, Ar*H*), 6.61 (1H, dt, *J* = 16.0, 1.6 Hz, ArC*H*), 6.31 (1H, dt, *J* = 15.9, 6.0 Hz, C*H*CH$_2$O), 4.15 (2H, dd, *J* = 6.0, 1.5 Hz, C*H*$_2$O), 3.69 (1H, hept, *J* = 6.1 Hz, C*H*(CH$_3$)$_2$), 1.21 (6H, d, *J* = 6.1 Hz, 2 x C*H*$_3$); **^{13}C{^{1}H} NMR** ($CDCl_3$, 101 MHz) δ 137.0, 131.9, 128.6 (2C), 127.7, 127.0, 126.6 (2C), 71.1, 68.9, 22.3 (2C).

Data consistent with literature values.[7]

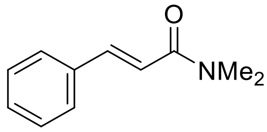

***N,N*-Dimethylcinnamamide 1u.** Oxalyl chloride (1.4 g, 11 mmol, 1.5 equiv.) and DMF (5 drops) were added to a solution of *trans*-cinnamic acid (1.2 g, 7.8 mmol, 1.0 equiv.) in dry CH_2Cl_2 (30 mL) under Ar. The reaction mixture was stirred at room temperature for 2 h before being cooled to 0 °C. A solution of dimethylamine 2 M in THF (15 mL, 30 mmol, 3.9 equiv.) was added dropwise to the cooled mixture. The reaction mixture was stirred 15 min at 0 °C before being stirred 2 h at room temperature. Water (25 mL) was slowly added to quench the reaction, and the mixture was reduced *in vacuo*. The residue was extracted with CH_2Cl_2 (3 x 10 mL), and the combined organic layers were washed with an aqueous 1 M HCl solution, a saturated aqueous $NaHCO_3$ solution and brine. The organic layer was dried over anhydrous Na_2SO_4, filtered and concentrated *in vacuo*. The crude was purified by column chromatography (MeOH/CH_2Cl_2 5%) to afford *N*,*N*-dimethylcinnamamide **1u** (1.1 g, 6.0 mmol, 77%) as a light orange solid.

^{1}H NMR (400 MHz, $CDCl_3$) δ 7.67 (1H, dd, *J* = 15.4, 2.2 Hz, PhC*H*), 7.53 (2H, d, *J* = 7.0 Hz, Ar*H*), 7.43–7.29 (3H, m, Ar*H*), 6.89 (1H, dd, *J* = 15.5, 2.3 Hz, PhCHC*H*CO), 3.12 (6H, d, *J* = 41.2 Hz, C*H*$_3$); **^{13}C{^{1}H} NMR** ($CDCl_3$, 101 MHz) δ 166.8, 142.5, 135.6, 129.7, 128.9, 127.9, 117.6, 37.6, 36.1.

Data consistent with literature values.[8]

10. Epoxides 2q, 2r and 2s synthesis

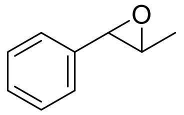

2-Methyl-3-phenyloxirane 2q was synthesized according to a procedure reported by Geng *et al.*[9] Aqueous 2 M NaOH (4 µL) was added to an aqueous $4 \cdot 10^{-4}$ M EDTA solution (9 mL) and the pH was adjusted to 4 with dilute aqueous NaOH. The mixture was added to a solution of *trans*-β-methylstyrene (0.40 mL, 3.1 mmol, 1.0 equiv.) in acetonitrile (36 mL). Sodium chlorite (1,1 g, 9.3 mmol, 3.1 equiv.) was added, the reaction flask was topped with a condenser and the reaction mixture was stirred at 65 °C overnight. The colorless mixture turned yellow. It was

cooled to 0 °C, and the mixture was slowly quenched by dropwise addition of an aqueous 1 M $Na_2S_2O_3$ solution until no more peroxide was detected by starch paper. The colorless quenched mixture was reduced *in vacuo* before being extracted with CH_2Cl_2 (3 x 15 ml). The combined organic layers were washed with water and brine, dried over anhydrous Na_2SO_4, filtered and concentrated *in vacuo*. The crude was purified by column chromatography (Et_2O/pentane 1:19-1:9) to afford 2-methyl-3-phenyloxirane **2q** (0.28 g, 2.1 mmol, 68%) as a colorless oil.

^{1}H NMR (400 MHz, $CDCl_3$) δ 7.37–7.21 (5H, m, Ar*H*), 3.56 (1H, d, *J* = 2.0 Hz, ArC*H*O), 3.03 (1H, qd, *J* = 5.1, 2.1 Hz, C*H*O), 1.44 (3H, d, *J* = 5.1 Hz, C*H*$_3$); **^{13}C{^{1}H} NMR** ($CDCl_3$, 101 MHz) δ 137.9, 128.6 (2C), 128.2, 125.7 (2C), 59.7, 59.2, 18.1.

Data consistent with literature values.[10]

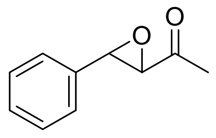

1-(3-Phenyloxiran-2-yl)ethan-1-one 2r was synthesized according to a procedure reported by Yao *et al.*[11] Hydrogen peroxide (30% in water, 3.7 mL, 48 mmol, 6.0 equiv.) was added to a mixture of benzalacetone (1.2 g, 8.0 mmol, 1.0 equiv.) and sodium bicarbonate (2.7 g, 32 mmol, 4.0 equiv.) in acetonitrile/water 3:2 (160 mL). The reaction mixture was stirred at room temperature for 48 h before being quenched by slow addition of 1 M $Na_2S_2O_3$ until no more peroxide was visible on starch paper. The mixture was reduced *in vacuo* and the residue was extracted with EtOAc (3 x 10 mL). The combined organic layers were washed with brine, dried over anhydrous Na_2SO_4, filtered and concentrated *in vacuo*. The crude was purified by column chromatography (Et_2O/pentane 10-20%) to afford a 3:2 mixture of 1-(3-phenyloxiran-2-yl)ethan-1-one **2r** (0.30 g, 1.9 mmol, 23%) and benzalacetone (0.20 g, 1.4 mmol) as a light-yellow oil. Due to the instability of the epoxide, the mixture was used without further purification for the next step.

^{1}H NMR (400 MHz, $CDCl_3$) δ 7.59–7.23 (5H, m), 4.01 (1H, d, *J* = 1.8 Hz), 3.49 (1H, d, *J* = 1.8 Hz), 2.20 (3H, s); **^{13}C{^{1}H} NMR** ($CDCl_3$, 101 MHz) δ 204.3, 135.2, 129.2, 128.9 (2C), 125.8 (2C), 63.6, 57.9, 24.9.

Data consistent with literature values.[12]

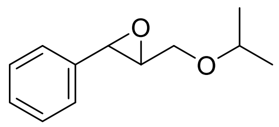

2-(Isopropoxymethyl)-3-phenyloxirane 2s was synthesized according to a procedure reported by Geng *et al.*[9] Aqueous 2 M NaOH (6 µL) was added to an aqueous $4 \cdot 10^{-4}$ M EDTA solution (13 mL) and the pH was adjusted to 4 with dilute aqueous NaOH. The mixture was added to a solution of 1-phenyl-3-isopropoxy-1-propene **1s** (0.79 g, 4.5 mmol, 1.0 equiv.) in acetonitrile (53 mL). Sodium chlorite (1.6 g, 14 mmol, 3.1 equiv.) was added, the reaction flask was topped with a condenser and the reaction mixture was stirred at 65 °C overnight. The colorless mixture turned yellow. It was cooled to 0 °C, and the mixture was slowly quenched by dropwise addition of an aqueous 1 M $Na_2S_2O_3$ solution until no more peroxide was detected by starch paper. The colorless quenched mixture was reduced *in vacuo* before being extracted with CH_2Cl_2 (3 x 15 ml). The combined organic layers were washed with water and brine, dried over anhydrous Na_2SO_4, filtered and concentrated *in vacuo*. The crude was purified by column chromatography (Et_2O/pentane 5-10%) to afford 2-(isopropoxymethyl)-3-phenyloxirane **2s** (0.23 g, 1.2 mmol, 26%) as a light-yellow oil.

R_f 0.24 (Et_2O/pentane 10%); **IR** (thin film, ν_{max}/cm^{-1}) 2974, 2926, 2921, 2871, 1466, 1382, 1371, 1150, 1130, 1091, 885, 751, 700; **^{1}H NMR** (400 MHz, $CDCl_3$) δ 7.41–7.22 (5H, m, Ar*H*), 3.80 (2H, dd, *J* = 11.4, 2.9 Hz, C*H*O, C*H*$_2$), 3.69

(1H, hept, J = 6.1 Hz, $CH(CH_3)_2$), 3.58 (1H, dd, J = 11.5, 5.2 Hz, CH_2), 3.20 (1H, ddd, J = 5.3, 3.1, 2.1 Hz, CHO), 1.20 (6H, t, J = 5.8 Hz, 2 x CH_3); **$^{13}C\{^1H\}$ NMR** ($CDCl_3$, 101 MHz) δ 137.2, 128.6, 128.3, 125.9, 72.5, 68.2, 61.7, 56.2, 22.3, 22.1; **HRMS** (APCI) calc. for $C_{12}H_{17}O_2$ ($[M+H]^+$) 193.1223, found 193.1223.

11. Iodohydrins 3a-v synthesis

11.1 Procedure A

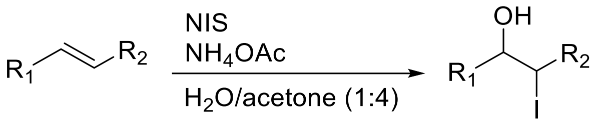

1 **3**

Based on a literature procedure.[13] *N*-iodosuccinimide (1.1 equiv.) and distilled water (1.0 M with respect to the olefin) were added to a mixture of olefin (1.0 equiv.) and ammonium acetate (0.1 equiv.) in acetone (0.25 M with respect to the olefin). The reaction mixture was stirred in the dark at room temperature. Upon completion as indicated by TLC, the mixture was concentrated *in vacuo*, and the residue was redissolved in water and extracted with ethyl acetate (3 × 10 mL). The combined organic layers were washed with an aqueous 10% solution of $Na_2S_2O_3$, dried over anhydrous Na_2SO_4, filtered and concentrated *in vacuo*. The crude was purified by column chromatography to afford the wanted iodohydrin **3**.

11.2 Procedure B

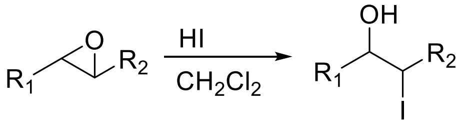

2 **3**

Hydroiodic acid (57 wt.% in H_2O, 1.2 equiv.) was added to a solution of epoxide **2** (1.0 equiv.) in CH_2Cl_2 (0.5 M with respect to the epoxide) and the reaction mixture was stirred in the dark. Upon completion as indicated by TLC, the mixture was diluted with CH_2Cl_2 (20 mL). It was washed with an aqueous 10% solution of $NaHCO_3$, an aqueous 10% solution of $Na_2S_2O_3$, dried over anhydrous Na_2SO_4, filtered and concentrated *in vacuo*. The crude was purified by column chromatography to afford the wanted iodohydrin **3**.

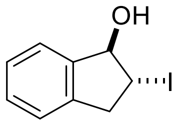

2-Iodo-2,3-dihydro-1*H*-inden-1-ol 3a was synthesized from indene (1.0 mL, 7.7 mmol, 1.0 equiv.), *N*-iodosuccinimide (1.9 g, 8.7 mmol, 1.1 equiv.) and NH_4OAc (65 mg, 0.83 mmol, 0.11 equiv.) in H_2O/acetone 1:4 (38 mL) according to Procedure A. The crude was purified by column chromatography (EtOAc/pentane 20%) to afford 2-iodo-2,3-dihydro-1*H*-inden-1-ol **3a** (1.3 g, 5.0 mmol, 65%) as a white solid.

^1H NMR (400 MHz, $CDCl_3$) δ 7.48–7.39 (1H, m, ArH), 7.32–7.27 (2H, m, ArH), 7.25–7.21 (1H, m, ArH), 5.40 (1H, t, J = 6.3 Hz, CHOH), 4.22 (1H, ddd, J = 8.0, 7.3, 6.4 Hz, CHI), 3.60 (1H, dd, J = 16.2, 7.3 Hz, CH_2), 3.32 (1H, dd, J = 16.2, 8.0 Hz, CH_2), 2.31 (1H, d, J = 6.2 Hz, OH); **$^{13}C\{^1H\}$ NMR** ($CDCl_3$, 101 MHz) δ 142.2, 141.1, 129.0, 127.7, 124.5, 124.0, 85.2, 42.4, 30.2.

Data consistent with literature values.[14]

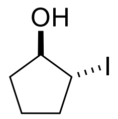

2-Iodocyclopentanol 3b was synthesized from cyclopentene (0.22 mL, 2.5 mmol, 1.0 equiv.), *N*-iodosuccinimide (0.63 g, 2.8 mmol, 1.1 equiv.) and NH_4OAc (26 mg, 0.33 mmol, 0.13 equiv.) in H_2O/acetone 1:4 (12 mL) according to Procedure A. The crude was purified by column chromatography (EtOAc/heptane 30%) to afford 2-iodocyclopentanol **3b** (0.25 g, 1.2 mmol, 47%) as a colorless oil.

^{1}**H NMR** (400 MHz, $CDCl_3$) δ 4.45 (1H, dd, *J* = 6.6, 4.0 Hz, C*H*OH), 4.04 (1H, q, *J* = 5.9 Hz, C*H*I), 2.37 (1H, dq, *J* = 14.4, 7.3 Hz, C*H*$_2$), 2.09 (2H, ddq, *J* = 28.4, 13.9, 7.1 Hz, C*H*$_2$), 1.95 (1H, d, *J* = 3.5 Hz, O*H*), 1.82 (2H, p, *J* = 7.6 Hz, C*H*$_2$), 1.65–1.51 (1H, m, C*H*$_2$); ^{13}**C{^{1}H} NMR** ($CDCl_3$, 101 MHz) δ 82.3, 35.8, 34.3, 31.1, 22.2.

Data consistent with literature values.[15]

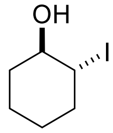

2-Iodocyclohexanol 3c was synthesized from cyclohexene (1.0 mL, 9.9 mmol, 1.0 equiv.), *N*-iodosuccinimide (2.5 g, 11 mmol, 1.1 equiv.) and NH_4OAc (79 mg, 1.0 mmol, 0.10 equiv.) in H_2O/acetone 1:4 (50 mL) according to Procedure A. The crude was purified by column chromatography (EtOAc/pentane 20%) to afford 2-iodocyclohexanol **3c** (1.5 g, 6.8 mmol, 69%) as a colorless oil.

^{1}**H NMR** (400 MHz, $CDCl_3$) δ 4.04 (1H, ddd, *J* = 12.3, 9.7, 4.3 Hz, C*H*I), 3.65 (1H, tdd, *J* = 10.0, 4.5, 2.6 Hz, C*H*O), 2.47 (1H, dqd, *J* = 13.3, 3.6, 1.9 Hz, CHIC*H*$_2$), 2.31 (1H, d, *J* = 2.7 Hz, O*H*), 2.17–2.07 (1H, m, C*H*$_2$CHOH), 2.07–2.01 (1H, m, CHIC*H*$_2$), 1.91–1.79 (1H, m, C*H*$_2$CH$_2$CHOH), 1.59–1.47 (1H, m, CHICH$_2$C*H*$_2$), 1.47–1.19 (3H, m, CHICH$_2$C*H*$_2$, C*H*$_2$CH$_2$CHOH, C*H*$_2$CHOH); ^{13}**C{^{1}H} NMR** ($CDCl_3$, 101 MHz) δ 76.1, 43.6, 38.8, 33.8, 28.1, 24.6.

Data consistent with literature values.[14]

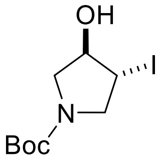

***tert*-Butyl 3-hydroxy-4-iodopyrrolidine-1-carboxylate 3d** was synthesis according to a procedure reported by Narender *et al.*[16] A solution of β-cyclodextrin (2.8 g, 2.5 mmol, 1.0 equiv.) in water (30 mL) was stirred at 65 °C until full dilution. A solution of *N*-Boc-2,5-dihydro-1*H*-pyrrole (0.45 mL, 2.5 mmol, 1.0 equiv.) in acetone (2 mL) was added dropwise to the warm solution, and the reaction mixture was allowed to cool to room temperature. *N*-iodosaccharin (0.88 g, 2.8 mmol, 1.1 equiv.) was added and the mixture was stirred in the dark at room temperature for 1 h. EtOAc (30 mL) was added, and the reaction mixture was filtered under reduced pressure. The filtrate was extracted with EtOAc (3 x 10 mL), the combined organic layers were washed with an aqueous 10% solution of $Na_2S_2O_3$, dried over anhydrous Na_2SO_4, filtered and concentrated *in vacuo*. The crude was purified by column chromatography (EtOAc/heptane 50%) to afford *tert*-butyl 3-hydroxy-4-iodopyrrolidine-1-carboxylate **3d** (0.47 g, 1.5 mmol, 60%) as a colorless oil.

R*f* 0.31 (EtOAc/heptane 50%); **IR** (thin film, ν_{max}/cm^{-1}) 3387, 2977, 2928, 1670, 1421, 1369, 1167, 1116, 959, 869, 771; ^{1}**H NMR** (400 MHz, $CDCl_3$, mixture of rotamers) δ 4.54 (1H, d, *J* = 5.6 Hz, C*H*OH), 4.16–4.03 (2H, m, C*H*I, C*H*$_2$CHI), 3.97–3.85 (1H, m, C*H*$_2$CHOH), 3.85–3.70 (1H, m, C*H*$_2$CHI), 3.37 (1H, t, *J* = 11.5 Hz, C*H*$_2$CHOH), 2.54 (1H, d, *J* = 3.9 Hz, O*H*), 1.47 (9H, s, 3 x C*H*$_3$); ^{13}**C{^{1}H} NMR** ($CDCl_3$, 101 MHz, mixture of rotamers) δ 154.5 (2C), 80.3

(2C), 78.9, 78.0, 54.5, 54.0, 51.5, 51.1, 29.2, 28.6, 26.1, 25.7; **HRMS** (APCI) calc. for C_4H_9INO ([M-Boc+H]$^+$) 213.9723, found 213.9721.

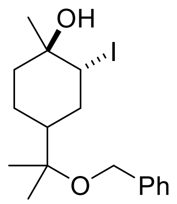

4-(2-(Benzyloxy)propan-2-yl)-2-iodo-1-methylcyclohexanol 3e was synthesized from 4-(1-(benzyloxy)-1-methylethyl)-1-methylcyclohexene **1e** (0.44 g, 1.8 mmol, 1.0 equiv.), *N*-iodosuccinimide (0.44 g, 2.0 mmol, 1.1 equiv.) and NH_4OAc (28 mg, 0.36 mmol, 0.20 equiv.) in H_2O/acetone 1:4 (8.2 mL) according to Procedure A. The crude was purified by column chromatography (Et_2O/pentane 50%) to afford 4-(2(benzyloxy)propan-2-yl)-2-iodo-1-methylcyclohexanol **3e** (0.23 g, 0.59 mmol, 33%) as a colorless oil.

R*f* 0.32 (Et_2O/pentane 50%); **IR** (thin film, v_{max}/cm^{-1}) 2974, 2934, 2879, 1454, 1384, 1320, 1230, 1207, 1167, 1128, 1086, 1059, 1029, 987, 948, 907, 885, 735, 698; **^{1}H NMR** (400 MHz, $CDCl_3$) δ 7.41–7.20 (5H, m, Ar*H*), 4.54–4.39 (3H, m, OC*H*$_2$, C*H*I), 2.26–1.96 (4H, m, 3 x C*H*$_2$, C*H*), 1.70–1.53 (3H, m, 3 x C*H*$_2$), 1.51 (3H, s, C(C*H*$_3$)OH), 1.49–1.42 (1H, m, O*H*), 1.27 (3H, s, C*H*$_3$), 1.21 (3H, s, C*H*$_3$); **^{13}C{^{1}H} NMR** ($CDCl_3$, 101 MHz) δ 140.0, 128.4 (2C), 127.5 (2C), 127.2, 76.8, 71.5, 63.3, 43.0, 41.0, 33.7, 33.3, 32.9, 23.7, 23.6, 22.5; **HRMS** (APCI) calc. for $C_{17}H_{29}INO_2$ ([M+NH$_4$]$^+$) 406.1238, found 406.1237.

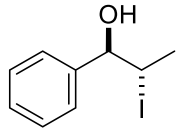

2-Iodo-1-phenylpropan-1-ol 3f was synthesized from *trans*-β-methylstyrene (1.0 mL, 7.6 mmol, 1.0 equiv.), *N*-iodosuccinimide (2.1 g, 9.2 mmol, 1.2 equiv.) and NH_4OAc (60 mg, 0.78 mmol, 0.10 equiv.) in H_2O/acetone 1:4 (36 mL) according to Procedure A. The crude was purified by column chromatography (Et_2O/heptane 10%) to afford 2-iodo-1-phenylpropan-1-ol **3f** (1.4 g, 5.4 mmol, 71%) as a colorless oil.

^{1}H NMR (400 MHz, $CDCl_3$) δ 7.44–7.28 (5H, m, Ar*H*), 4.96 (1H, t, *J* = 3.5 Hz, C*H*OH), 4.53 (1H, qd, *J* = 7.0, 3.7 Hz, C*H*I), 2.36 (1H, d, *J* = 3.4 Hz, O*H*), 1.74 (3H, d, *J* = 7.0 Hz, C*H*$_3$); **^{13}C{^{1}H} NMR** ($CDCl_3$, 101 MHz) δ 139.9, 128.5 (2C), 128.2, 126.6 (2C), 78.6, 36.1, 21.4.

Data consistent with literature values.[17]

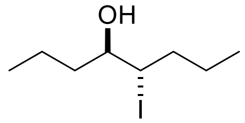

5-Iodooctan-4-ol 3g was synthesized from trans-4-octene (1.0 mL, 6.2 mmol, 1.0 equiv.), *N*-iodosuccinimide (1.7 g, 7.5 mmol, 1.2 equiv.) and NH_4OAc (48 mg, 0.63 mmol, 0.10 equiv.) in H_2O/acetone 1:4 (29 mL) according to Procedure A. The crude was purified by column chromatography (Et_2O/pentane 10-20%) to afford 5-iodooctan-4-ol **3g** (1.2 g, 4.8 mmol, 77%) as a light-yellow oil.

R*f* 0.38 (Et_2O/pentane 20%); **IR** (liquid, v_{max}/cm^{-1}) 3409, 2959, 2933, 2873, 1465, 1383, 1257, 1115, 1067, 1007, 847, 747; **^{1}H NMR** (400 MHz, $CDCl_3$) δ 4.34 (1H, dt, *J* = 10.5, 3.4 Hz, C*H*I), 3.38–3.25 (1H, m, C*H*OH), 1.95–1.79 (2H, m, O*H*, C*H*$_2$CHOH), 1.72–1.49 (5H, m, 3 x C*H*$_2$CH$_2$CH$_3$, 2 x CH$_2$C*H*$_2$CH$_3$), 1.47–1.30 (2H, m, 2 x CH$_2$C*H*$_2$CH$_3$), 1.02–

0.88 (6H, m, 2 x CH_3); **^{13}C{^{1}H} NMR** ($CDCl_3$, 101 MHz) δ 75.3, 49.9, 37.0, 36.8, 23.4, 19.3, 14.2, 13.4; **HRMS** (APCI) calc. for $C_8H_{21}INO$ [$(M+NH_4)^+$] 274.0662, found 274.0685.

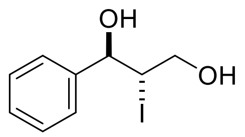

2-Iodo-1-phenyl-propan-1,3-diol 3h was synthesized from cinnamyl alcohol (0.55 g, 4.0 mmol, 1.0 equiv.), *N*-iodosuccinimide (0.99 g, 4.4 mmol, 1.1 equiv.) and NH_4OAc (31 mg, 0.40 mmol, 0.10 equiv.) in H_2O/acetone 1:4 (19 mL) according to Procedure A. The crude was purified by column chromatography (Et_2O/pentane 50%) to afford 2-iodo-1-phenyl-propan-1,3-diol **3h** (0.65 g, 2.3 mmol, 58%) as a white solid.

^{1}H NMR (400 MHz, $CDCl_3$) δ 7.45–7.28 (5H, m, Ar*H*), 5.06 (1H, dd, *J* = 6.3, 4.1 Hz, C*H*OH), 4.46 (1H, ddd, *J* = 6.3, 5.4, 4.6 Hz, C*H*I), 3.97 (1H, ddd, *J* = 12.4, 6.8, 4.6 Hz, CH_2), 3.82 (1H, ddd, *J* = 12.3, 6.2, 5.4 Hz, CH_2), 2.92 (1H, d, *J* = 4.1 Hz, CHO*H*), 2.54 (1H, t, *J* = 6.5 Hz, CH_2O*H*); **^{13}C{^{1}H} NMR** ($CDCl_3$, 101 MHz) δ 141.0, 128.8 (2), 128.7, 126.7 (2), 78.7, 66.1, 41.6.

Data consistent with literature values.[18]

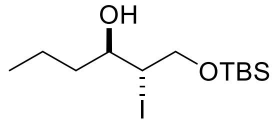

1-((*tert*-Butyldimethylsilyl)oxy)-2-iodohexan-3-ol 3i was synthesized from **1i** (0.78 g, 3.7 mmol, 1.0 equiv.), N-iodosuccinimide (0.93 g, 4.1 mmol, 1.1 equiv) and ammonium acetate (38 mg, 0.49 mmol, 0.10 equiv.) in H_2O/acetone 1:4 (18 mL) according to Procedure A. The crude was purified by column chromatography (EtOAc/heptane 5-50%) to afford 1-((*tert*-butyldimethylsilyl)oxy)-2-iodohexan-3-ol **3i** (88 mg, 0.25 mmol, 7%) as a colorless oil.

R_f 0.33 (Et_2O/pentane 10%); **IR** (liquid, ν_{max}/cm^{-1}) 2956, 2929, 2856, 1465, 1256, 1091, 1059, 835, 777; **^{1}H NMR** (400 MHz, $CDCl_3$) δ 4.22 (1H, dt, *J* = 7.9, 5.4 Hz, C*H*I), 4.07 (1H, dd, *J* = 11.0, 4.8 Hz, CHICH_2), 3.91 (1H, dd, *J* = 10.9, 8.0 Hz, CHICH_2), 3.68 (1H, dtd, *J* = 8.5, 5.5, 2.9 Hz, C*H*OH), 2.99 (1H, d, *J* = 5.2 Hz, O*H*), 1.71 (1H, ddd, *J* = 12.7, 6.9, 3.5 Hz, CH_3CH_2), 1.61–1.47 (2H, m, CH_3CH_2, CH_3CH_2CH_2), 1.47–1.34 (1H, m, CH_3CH_2CH_2), 0.95 (3H, t, *J* = 7.1 Hz, C$H_3$$CH_2$), 0.91 (9H, s, C(C$H_3$)$_3$), 0.10 (5H, d, *J* = 2.0 Hz, Si(CH_3)$_2$); **^{13}C{^{1}H} NMR** ($CDCl_3$, 101 MHz) δ 74.5, 67.8, 41.5, 38.4, 25.9, 18.8, 18.3, 14.1, -5.2, -5.3; **HRMS** (APCI) calc. for $C_{12}H_{25}IOSi$ ($[M+H-H_2O]^+$) 341.0792, found 341.0792.

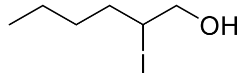

2-Iodohexan-1-ol 3j was synthesized from 1-hexene (1.0 mL, 7.8 mmol, 1.0 equiv.), *N*-iodosuccinimide (2.1 g, 9.4 mmol, 1.2 equiv.) and NH_4OAc (62 mg, 0.79 mmol, 0.10 equiv.) in H_2O/acetone 1:4 (36 mL) according to Procedure A. The reaction produced a 1:4 mixture of 2-iodohexan-1-ol **3j** and 1-iodohexan-2-ol **3k**. The isomers were separated by column chromatography (Et_2O/pentane 0-30%) to afford 2-iodohexan-1-ol **3j** (0.25 g, 1.1 mmol, 14%) as a colorless oil.

R_f 0.33 (Et_2O/pentane 30%); **IR** (liquid, ν_{max}/cm^{-1}) 3367, 2955, 2926, 2859, 1459, 1380, 1231, 1154, 1099, 1053, 1034, 1002, 732; **^{1}H NMR** (400 MHz, $CDCl_3$) δ 4.22 (1H, ddt, *J* = 9.4, 6.5, 4.9 Hz, C*H*I), 3.80–3.65 (2H, m, CH_2O), 1.96 (1H, m O*H*), 1.87 (1H, m, CH_2CHI), 1.77 (1H, m, CH_2CHI), 1.59–1.45 (1H, m, C$H_2$$CH_2$CHI), 1.45–1.26 (3H, m,

CH_2CH_2CHI, 2 x CH_3CH_2), 0.92 (3H, t, *J* = 7.1 Hz, CH_3); **$^{13}C\{^1H\}$ NMR** ($CDCl_3$, 101 MHz) δ 68.7, 42.2, 36.1, 31.7, 22.1, 14.0; **HRMS** (ESI) calc. for $C_6H_{13}IONa$ ($[M+Na]^+$) 250.9903, found 250.9903.

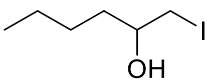

1-Iodohexan-2-ol 3k was synthesized from 1-hexene (1.0 mL, 7.8 mmol, 1.0 equiv.), *N*-iodosuccinimide (2.1 g, 9.4 mmol, 1.2 equiv.) and NH_4OAc (62 mg, 0.79 mmol, 0.10 equiv.) in H_2O/acetone 1:4 (36 mL) according to Procedure A. The reaction produced a 1:4 mixture of 2-iodohexan-1-ol **3j** and 1-iodohexan-2-ol **3k**. The isomers were separated by column chromatography (Et_2O/pentane 0-30%) to afford 1-iodohexan-2-ol **3k** (1.1 g, 4.8 mmol, 62%) as a colorless oil.

^1H NMR (400 MHz, $CDCl_3$) δ 3.57–3.46 (1H, m, CHOH), 3.40 (1H, dd, *J* = 10.1, 3.5 Hz, CH_2I), 3.23 (1H, dd, *J* = 10.1, 6.8 Hz, CH_2I), 1.93 (1H, d, *J* = 5.3 Hz, OH), 1.56 (2H, dtd, *J* = 7.9, 5.9, 3.5 Hz, CH_2CHOH), 1.48–1.27 (4H, m, $CH_2CH_2CH_3$, $CH_2CH_2CH_3$), 0.98–0.85 (3H, m, CH_3); **$^{13}C\{^1H\}$ NMR** ($CDCl_3$, 101 MHz) δ 71.1, 36.5, 28.0, 22.7, 17.0, 14.1.

Data consistent with literature values.[19]

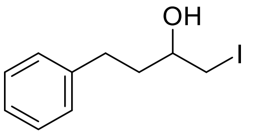

1-Iodo-4-phenylbutan-2-ol 3l was synthesized from 4-phenyl-1-butene (0.60 mL, 4.0 mmol, 1.0 equiv.), *N*-iodosuccinimide (1.1 g, 4.8 mmol, 1.2 equiv.) and NH_4OAc (34 mg, 0.44 mmol, 0.11 equiv.) in H_2O/acetone 1:4 (19 mL) according to Procedure A. The crude was purified by column chromatography (Et_2O/pentane 0-40%) to afford 1-iodo-4-phenylbutan-2-ol **3l** (0.51 g, 1.9 mmol, 47%) as a colorless oil.

^1H NMR (400 MHz, $CDCl_3$) δ 7.38–7.21 (5H, m, ArH), 3.58 (1H, dddd, *J* = 12.4, 6.8, 5.4, 3.5 Hz, CHOH), 3.43 (1H, dd, *J* = 10.2, 3.5 Hz, CH_2I), 3.30 (1H, dd, *J* = 10.2, 6.8 Hz, CH_2I), 2.91–2.81 (1H, m, CH_2CHOH), 2.76 (1H, dt, *J* = 13.8, 8.1 Hz, CH_2CHOH), 2.04 (1H, d, *J* = 5.4 Hz, OH), 1.97–1.88 (2H, m, CH_2); **$^{13}C\{^1H\}$ NMR** ($CDCl_3$, 101 MHz) δ 141.4, 128.7 (2C), 128.6 (2C), 128.6, 126.2, 70.3, 38.3, 32.1, 16.7.

Data consistent with literature values.[20]

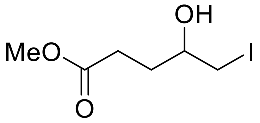

Methyl 4-hydroxy-5-iodopentanoate 3m was synthesized from methyl 4 -pentanoate (0.31 g, 2.7 mmol, 1.0 equiv.), *N*-iodosuccinimide (0.68 g, 3.0 mmol, 1.1 equiv.) and NH_4OAc (28 mg, 0.35 mmol, 0.13 equiv.) in H_2O/acetone 1:4 (12.5 mL) according to Procedure A. The crude was purified by column chromatography (Et_2O/pentane 50-60%) to afford methyl 4-hydroxy-5-iodopentanoate **3m** (0.34 g, 1.3 mmol, 48%) as a colorless oil.

R_f 0.26 (Et_2O/pentane 50%); **IR** (thin film, ν_{max}/cm^{-1}) 3318, 2950, 2837, 1720, 1655, 1442, 1016; **^1H NMR** (400 MHz, $CDCl_3$) δ 3.69 (3H, s, OCH_3), 3.61 (1H, dh, J = 14.4, 3.9 Hz, CHOH), 3.36 (1H, dd, J = 10.2, 4.0 Hz, CH_2I), 3.24 (1H, dd, J = 10.2, 6.6 Hz, CH_2I), 2.50 (2H, t, *J* = 7.1 Hz, CH_3OCOCH_2), 2.35 (1H, d, *J* = 5.5 Hz, OH), 1.96 (1H, dtd, *J* = 14.5, 7.3, 3.5 Hz, CH_2), 1.82 (1H, ddt, *J* = 14.0, 8.8, 6.9 Hz, CH_2); **$^{13}C\{^1H\}$ NMR** ($CDCl_3$, 101 MHz) δ 174.2, 70.4, 52.0, 31.5, 30.4, 15.5; **HRMS** (APCI) calc. for $C_6H_{12}IO_3$ ($[M+H]^+$) 258.9826, found 258.9824.

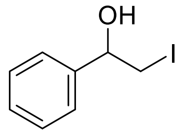

2-Iodo1-phenylethan-1-ol 3n was synthesized from styrene (0.30 mL, 2.6 mmol, 1.0 equiv.), *N*-iodosuccinimide (0.61 g, 2.7 mmol, 1.0 equiv.) and NH_4OAc (24 mg, 0.30 mmol, 0.12 equiv.) in H_2O/acetone 1:4 (12 mL) according to Procedure A. The crude was purified by column chromatography (Et_2O/pentane 30%) to afford 2-iodo-1-phenylethan-1-ol **3n** (0.44 g, 1.8 mmol, 69%) as an orange oil.

^{1}H NMR (400 MHz, $CDCl_3$) δ 7.38 (5H, m, Ar*H*), 4.84 (1H, m, C*H*O), 3.50 (1H, m, CH_2I), 3.41 (1H, m, CH_2I), 2.51–2.37 (1H, m, O*H*); **^{13}C{^{1}H} NMR** ($CDCl_3$, 101 MHz) δ 141.2, 128.9, 128.5, 125.9, 74.2, 15.5.

Data consistent with literature values.[21]

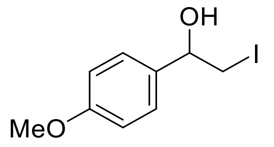

2-Iodo-1-(4-methoxyphenyl)ethan-1-ol 3o was synthesized from 1-methoxy-4-vinylbenzene (1.4 mL, 10 mmol, 1.0 equiv.), *N*-iodosuccinimide (2.4 g, 11 mmol, 1.1 equiv.) and NH_4OAc (0.11 g, 1.4 mmol, 0.14 equiv.) in H_2O/acetone 1:4 (48 mL) according to Procedure A. The crude was purified by column chromatography (Et_2O/pentane 30%) to afford 2-iodo-1-(4-methoxyphenyl)ethan-1-ol **3o** (1.5 g, 5.2 mmol, 52%) as a dark-red oil.

^{1}H NMR (400 MHz, $CDCl_3$) δ 7.30 (2H, d, *J* = 8.7 Hz, Ar*H*), 6.90 (2H, d, *J* = 8.8 Hz, Ar*H*), 4.80 (1H, d, *J* = 7.4 Hz, C*H*OH), 3.81 (3H, s, OCH_3), 3.52–3.33 (2H, m, CH_2I), 2.42 (1H, s, O*H*); **^{13}C{^{1}H} NMR** ($CDCl_3$, 101 MHz) δ 159.8, 133.4, 127.1 (2C), 114.2 (2C), 73.9, 55.5, 15.7.

Data consistent with literature values.[14]

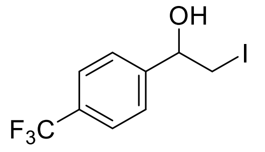

2-Iodo-1-(4-(trifluromethyl)phenyl)ethan-1-ol 3p was synthesized from 4-(trifluoromethyl)styrene (0.37 mL, 2.5 mmol, 1.0 equiv.), *N*-iodosuccinimide (0.61 g, 2.7 mmol, 1.1 equiv.) and NH_4OAc (29 mg, 0.37 mmol, 0.15 equiv.) in H_2O/acetone 1:4 (12 mL) according to Procedure A. The crude was purified by column chromatography (Et_2O/pentane 20%) to afford 2-iodo-1-(4-(trifluoromethyl)phenyl)ethan-1-ol **3p** (0.32 g, 1.0 mmol, 41%) as a colorless oil.

^{1}H NMR (400 MHz, $CDCl_3$) δ 7.64 (2H, d, *J* = 8.1 Hz, Ar*H*), 7.58–7.46 (2H, m, Ar*H*), 4.88 (1H, dt, *J* = 8.0, 3.7 Hz, C*H*OH), 3.52 (1H, dd, *J* = 10.4, 3.6 Hz, CH_2I), 3.39 (1H, dd, *J* = 10.4, 8.5 Hz, CH_2I), 2.54 (1H, d, *J* = 3.8 Hz, O*H*); **^{13}C{^{1}H} NMR** ($CDCl_3$, 101 MHz) δ 145.0, 130.5, 126.3 (2C), 125.8 (2C, q, $^3J_{C–F}$ = 3.8 Hz), 73.4, 14.9. *Note: The ^{13}C signal for the CF_3 group was not visible due to its low intensity.*

Data consistent with literature values.[22]

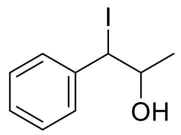

1-Iodo-1-phenylpropan-2-ol 3q was synthesized from 2-methyl-3-phenyloxirane **2q** (0.25 g, 1.9 mmol, 1.0 equiv.) and hydroiodic acid (57 wt.% in H_2O, 0.3 mL, 2.3 mmol, 1.2 equiv.) in CH_2Cl_2 (3.8 mL) according to Procedure B. The crude was purified by column chromatography (Et_2O/pentane 30%) to afford an inseparable 89:11 diastereomers mixture of 1-iodo-1-phenylpropan-2-ol **3q** (0.27 g, 1.0 mmol, 54%) as a colorless oil. *Note: the product degraded when kept under Ar at 8 °C overnight. It was resynthesized and used straight away for the next step. The NMR data were extracted from the inseparable mixture NMR.*

R*f* 0.30 (Et_2O/pentane 30%); **IR** (thin film, ν_{max}/cm^{-1}) 3419, 2975, 1492, 1453, 1116, 1076, 942, 759, 698; **HRMS** (APCI) calc. for $C_9H_{12}IO$ ($[M+H]^+$) 262.9927, found 262.9925.

major: **^{1}H NMR** (400 MHz, $CDCl_3$) δ 7.53–7.20 (5H, m, Ar*H*), 5.04 (1H, d, *J* = 6.8 Hz, C*H*I), 4.18 (1H, pd, *J* = 6.3, 3.8 Hz, C*H*OH), 2.04 (1H, d, *J* = 3.9 Hz, O*H*), 1.41 (3H, d, *J* = 6.1 Hz, C*H*$_3$); **^{13}C{^{1}H} NMR** ($CDCl_3$, 101 MHz) δ 140.3, 129.0 (2C), 128.9 (2C), 128.6, 72.5, 41.5, 21.4.

minor: **^{1}H NMR** (400 MHz, $CDCl_3$) δ 7.53–7.20 (5H, m, Ar*H*), 5.07-5.06 (1H, m, C*H*I), 4.06-3.98 (1H, m, C*H*OH), 2.34 (1H, d, *J* = 3.9 Hz, O*H*), 1.13 (3H, d, *J* = 6.1 Hz, C*H*$_3$); **^{13}C{^{1}H} NMR** ($CDCl_3$, 101 MHz) δ 128.5 (2C), 128.1 (3C), 72.2, 47.4, 20.1.

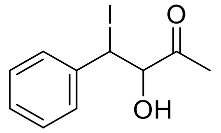

3-Hydroxy-4-iodo-4-phenylbutan-2-one 3r was synthesized from 1-(3-phenyloxiran-2-yl)ethan-1-one **2r** (0.30 g, 1.8 mmol, 1.0 equiv.) and hydroiodic acid (57 wt.% in H_2O, 0.29 mL, 2.3 mmol, 1.2 equiv.) in CH_2Cl_2 (3.7 mL) according to Procedure B. The crude was purified by column chromatography (Et_2O/pentane 20%) to afford an inseparable 86:14 diastereomers mixture of 3-hydroxy-4-iodo-4-phenylbutan-2-one **3r** (0.35 g, 1.2 mmol, 65%) as a light-yellow oil. *Note: the NMR data were extracted from the inseparable mixture NMR.*

R*f* 0.30 (Et_2O/pentane 20%); **IR** (thin film, ν_{max}/cm^{-1}) 3442, 1719, 1455, 1357, 1239, 1194, 1101, 699; **HRMS** (APCI) calc. for $C_{10}H_{12}IO_2$ ($[M+H]^+$) 290.9877, found 290.9876.

major: **^{1}H NMR** (400 MHz, $CDCl_3$) δ 7.53–7.44 (2H, m, Ar*H*), 7.33–7.22 (3H, m, Ar*H*), 5.54 (1H, d, *J* = 4.2 Hz, C*H*$_2$I), 4.59 (1H, dd, *J* = 4.9, 4.1 Hz, C*H*OH), 3.59 (1H, d, *J* = 4.8 Hz, O*H*), 2.15 (3H, s, C*H*$_3$); **^{13}C{^{1}H} NMR** ($CDCl_3$, 101 MHz) δ 204.4, 138.7, 129.0, 128.8, 128.7, 128.7, 82.3, 29.9, 26.9.

minor: **^{1}H NMR** (400 MHz, $CDCl_3$) δ 7.62-7.59 (2H, m, Ar*H*), 7.33-7.28 (3H, m, Ar*H*), 5.51 (1H, d, *J* = 2.9 Hz, C*H*$_2$I), 4.01 (1H, dd, *J* = 5.9, 2.8 Hz, C*H*OH), 3.89 (1H, d, *J* = 5.9 Hz, O*H*), 2.28 (3H, s, C*H*$_3$); **^{13}C{^{1}H} NMR** ($CDCl_3$, 101 MHz) δ 80.3, 34.3, 25.5. Note: *Carbonyl and aromatic carbons missing due to low intensity.*

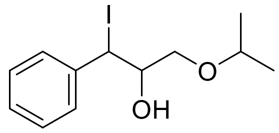

1-Iodo-3-isopropoxy-1-phenylpropan-2-ol 3s was synthesized from 2-(isopropoxymethyl)-3-phenyloxirane **2s** (0.23 g, 1.2 mmol, 1.0 equiv.) and hydroiodic acid (57 wt.% in H_2O, 0.19 mL, 1.4 mmol, 1.2 equiv.) in CH_2Cl_2 (2.3 mL) according to Procedure B. The crude was purified by column chromatography (Et_2O/pentane 20%) to afford

an inseparable 64:36 diastereomers mixture of 1-iodo-3-isopropoxy-1-phenylpropan-2-ol **3s** (0.26 g, 0.82 mmol, 70%) as a yellow oil. *Note: the NMR data were extracted from the inseparable mixture NMR.*

R*f* 0.22 (Et_2O/pentane 20%); **IR** (thin film, ν_{max}/cm^{-1}) 3422, 2971, 2869, 1736, 1454, 1382, 1371, 1147, 1128, 1095, 926, 765, 697; **HRMS** (APCI) calc. for $C_{12}H_{17}IO_2Na$ ([M+Na]$^+$) 343.0165, found 343.0166.

major: **^{1}H NMR** (400 MHz, $CDCl_3$) δ 7.46 (2H, m, Ar*H*), 7.36–7.21 (3H, m, Ar*H*), 5.22 (1H, d, *J* = 7.9 Hz, C*H*I), 4.29 (1H, dtd, *J* = 7.9, 5.1, 3.9 Hz, C*H*OH), 3.74 (1H, m, C*H*$_2$), 3.69 (1H, m, C*H*$_2$), 3.61 (1H, m, C*H*(CH_3)$_2$), 2.59 (1H, d, *J* = 4.9 Hz, O*H*), 1.18 (6H, dd, *J* = 8.8, 6.1 Hz, 2 x C*H*$_3$); **^{13}C{^{1}H} NMR** ($CDCl_3$, 101 MHz) δ 140.7, 128.9, 128.8 (2C), 128.7 (2C), 74.4, 72.5, 69.9, 34.0, 22.2 (2C).

minor: **^{1}H NMR** (400 MHz, $CDCl_3$) δ 7.46 (2H, m, Ar*H*), 7.37–7.20 (3H, m, Ar*H*), 5.26 (1H, d, *J* = 7.5 Hz, C*H*I), 3.85–3.78 (1H, m, C*H*OH), 3.52–3.44 (1H, m, C*H*(CH_3)$_2$), 3.35 (1H, m, C*H*$_2$), 3.22 (1H, m, C*H*$_2$), 2.73 (1H, d, *J* = 5.6 Hz, O*H*), 1.10 (6H, dd, *J* = 11.8, 6.1 Hz, 2 x C*H*$_3$); **^{13}C{^{1}H} NMR** ($CDCl_3$, 101 MHz) δ 141.4, 128.4, 128.3 (2C), 128.2 (2C), 74.7, 72.5, 68.7, 39.4, 22.3, 22.1.

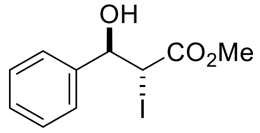

Methyl 3-hydroxy-2-iodo-3-phenylpropanoate 3t was synthesized according to a procedure reported by Urankar *et al.*[23] Water (0.5 mL) was added to a mixture of methyl *trans*-cinnamate (0.42 g, 2.6 mmol, 1.0 equiv.) and *N*-iodosaccharin (0.89 g, 2.8 mmol, 1.1 equiv.) in acetonitrile (5 mL). The reaction mixture was stirred in the dark at room temperature for 48 h. The reaction mixture was reduced *in vacuo*, the residue was redissolved in Et_2O and was washed with a saturated aqueous solution of $NaHCO_3$ and an aqueous 10% solution of $Na_2S_2O_4$. The organic layer was dried over anhydrous Na_2SO_4, filtered and concentrated *in vacuo*. The crude was purified by column chromatography (EtOAc/heptane 30%) to afford methyl 3-hydroxy-2-iodo-3-phenylpropanoate **3t** (0.21 g, 0.70 mmol, 27%) as a white solid.

^{1}H NMR (400 MHz, $CDCl_3$) δ 7.43–7.30 (5H, m, 5 x Ar*H*), 5.09 (1H, dd, *J* = 8.3, 5.8 Hz, C*H*OH), 4.59 (1H, d, *J* = 8.3 Hz, C*H*I), 3.78 (3H, s, OC*H*$_3$), 3.33 (1H, d, *J* = 5.8 Hz, O*H*); **^{13}C{^{1}H} NMR** ($CDCl_3$, 101 MHz) δ 171.9, 139.5, 128.9, 128.8 (2C), 127.1 (2C), 76.5, 53.3, 24.5.

Data consistent with literature values.[23]

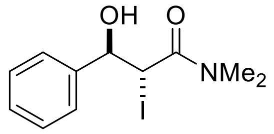

3-Hydroxy-2-iodo-*N,N*-dimethyl-3-phenylpropanamide 3u. *N*-iodosaccharin (0.87 g, 2.8 mmol, 1.1 equiv.) was added to a mixture of *N*,*N*-dimethylcinnamamide **1u** (0.44 g, 2.5 mmol, 1.0 equiv.) and ammonium acetate (26 mg, 0.34 mmol, 0.14 equiv.) .) in H_2O/acetone 1:4 (12 mL). The reaction mixture was stirred in the dark at room temperature for 69 h before being reduced *in vacuo*. The crude was redissolved in CH_2Cl_2 and was washed with a saturated aqueous $NaHCO_3$ solution and an aqueous 10% $Na_2S_2O_3$ solution. The organic layer was dried over anhydrous Na_2SO_4, filtered and reduced *in vacuo*. The crude was purified by column chromatography (Et_2O/pentane 90%) to afford 3-hydroxy-2-iodo-*N*,*N*-dimethyl-3-phenylpropanamide **3u** (0.49 g, 1.5 mmol, 61%) as a colorless oil.

R*f* 0.14 (Et_2O/pentane 80%); **IR** (thin film, ν_{max}/cm^{-1}) 3381, 2930, 1736, 1627, 1495, 1455, 1401, 1334, 1179, 1159, 1015, 760, 703; **^{1}H NMR** (400 MHz, $CDCl_3$) δ 7.46–7.28 (5H, m, Ar*H*), 5.14 (1H, t, *J* = 6.5 Hz, C*H*OH), 5.03 (1H, d, *J* = 6.6 Hz, O*H*), 4.65 (1H, d, *J* = 6.5 Hz, C*H*I), 2.93 (6H, d, *J* = 3.7 Hz, N(C*H*$_3$)$_2$); **^{13}C{^{1}H} NMR** ($CDCl_3$, 101 MHz) δ 170.8,

140.0, 128.6 (2), 128.4, 127.1 (2), 77.4, 38.1, 36.4, 24.0; **HRMS** (APCI) calc. for $C_{11}H_{15}INO_2$ ($[M+H]^+$) 320.0142, found 320.0141.

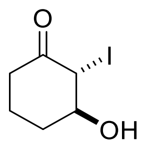

3-Hydroxy-2-iodocyclohexan-1-one 3v was synthesis according to a procedure reported by Narender *et al.*[16] A solution of β-cyclodextrin (3.2 g, 2.8 mmol, 1.1 equiv.) in water (30 mL) was stirred at 65 °C until full dilution. A solution of 2-cyclohexen-1-one (0.26 mL, 2.5 mmol, 1.0 equiv.) in acetone (2 mL) was added dropwise to the warm solution, and the reaction mixture was allowed to cool to room temperature. *N*-iodosaccharin (0.87 g, 2.8 mmol, 1.1 equiv.) was added and the mixture was stirred in the dark at room temperature for 21 h. EtOAc (30 mL) was added, and the reaction mixture was filtered under reduced pressure. The filtrate was extracted with EtOAc (3 x 10 mL), the combined organic layers were washed with an aqueous 10% solution of $Na_2S_2O_3$, dried over anhydrous Na_2SO_4, filtered and concentrated *in vacuo*. The crude was purified by column chromatography (Et_2O/pentane 60%) to afford 3-hydroxy-2-iodocyclohexan-1-one **3v** (0.17 g, 0.71 mmol, 28%) as a light-orange oil.

R*f* 0.35 (Et_2O/pentane 60%); **IR** (liquid, v_{max}/cm^{-1}) 3391, 2951, 1701, 1420, 1222, 1059, 971; **^{1}H NMR** (400 MHz, $CDCl_3$) δ 4.54 (1H, d, *J* = 6.6 Hz, C*H*I), 4.17 (1H, dt, J = 7.0, 3.5 Hz, C*H*OH), 2.97 (1H, ddd, J = 14.0, 8.0, 5.4 Hz, COC*H*₂), 2.49–2.33 (2H, m, COC*H*₂, CH(OH)C*H*₂), 2.32 (1H, d, *J* = 3.0 Hz, O*H*), 2.19–2.03 (1H, m, C*H*₂), 1.85 (1H, dtd, *J* = 13.5, 7.6, 3.6 Hz, CH(OH)C*H*₂), 1.73 (1H, dtq, *J* = 17.7, 8.3, 4.1 Hz, C*H*₂); **^{13}C{^{1}H} NMR** ($CDCl_3$, 101 MHz) δ 202.5, 76.0, 40.8, 37.2, 29.6, 20.4; **HRMS** (APCI) calc. for $C_6H_{10}IO_2$ ($[M+H]^+$) 240.9720, found 240.9718.

12. Vinyl silanes 4a-v synthesis

12.1 Procedure C

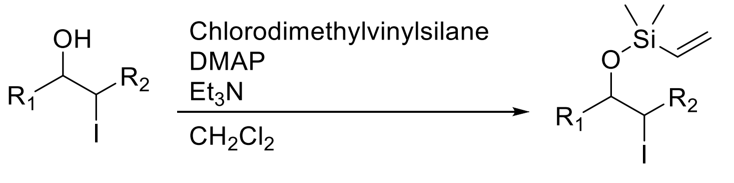

3 **4**

Chloro(dimethyl)vinylsilane (1.1 equiv.) was added to a mixture of iodohydrin **3** (1.0 equiv.), 4-dimethylaminopyridine (0.2 equiv.) and Et_3N (1.2 equiv.) in dry CH_2Cl_2 (0.1 M with respect to the iodohydrin) under Ar. The reaction mixture was stirred 20 min in the dark at room temperature. The reaction was quenched by slowly adding a few drops of isopropanol, and the mixture was reduced *in vacuo*. The residue was redissolved in water and extracted with Et_2O (3 x 10 mL). The combined organic layers were washed with a saturated aqueous $NaHCO_3$ solution and brine, dried over anhydrous Na_2SO_4, filtered and concentrated *in vacuo*. The crude was purified by column chromatography to afford the wanted silylated product **4**.

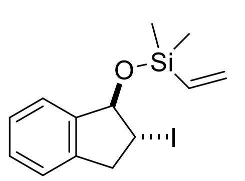

((2-Iodo-2,3-dihydro-1*H*-inden-1-yl)oxy)dimethylvinylsilane 4a was synthesized from 2-iodo-2,3-dihydro-1*H*-inden-1-ol **3a** (0.92 g, 3.5 mmol, 1.0 equiv.), chlorodimethylvinylsilane (0.61 mL, 4.2 mmol, 1.2 equiv.), 4-dimethylaminopyridine (85 mg, 0.70 mmol, 0.2 equiv.) and Et_3N (0.59 mL, 4.2 mmol, 1.2 equiv.) in dry CH_2Cl_2 (20

mL) according to Procedure C. The crude was purified by column chromatography (Et_2O/pentane 2.5%) to afford ((2-iodo-2,3-dihydro-1*H*-inden-1-yl)oxy)dimethylvinylsilane **4a** (1.1 g, 3.1 mmol, 86%) as a colorless oil.

$\mathbf{R_f}$ 0.47 (Et_2O/pentane 2.5%); **IR** (liquid, ν_{max}/cm^{-1}) 3048, 2955, 2854, 1719, 1596, 1465, 1410, 1361, 1257, 1220, 1179, 1127, 1086, 1041, 1011, 959, 862, 784, 747, 721, 706; **^{1}H NMR** (400 MHz, $CDCl_3$) δ 7.29 (1H, q, *J* = 5.4 Hz, Ar*H*), 7.26–7.22 (2H, m, 2 × Ar*H*), 7.20 (1H, q, *J* = 4.5 Hz, Ar*H*), 6.30 (1H, dd, *J* = 20.3, 14.9 Hz, Si(C*H*CH$_2$)), 6.10 (1H, dd, *J* = 14.9, 3.8 Hz, Si(CHC*H*$_2$)), 5.89 (1H, dd, *J* = 20.3, 3.8 Hz, Si(CHC*H*$_2$)), 5.40 (1H, d, *J* = 5.8 Hz, C*H*O), 4.21 (1H, td, *J* = 7.4, 5.8 Hz, C*H*I), 3.60 (1H, dd, *J* = 16.3, 7.3 Hz, CHIC*H*$_2$), 3.28 (1H, dd, *J* = 16.2, 7.6 Hz, CHIC*H*$_2$), 0.37 (3H, s, Si(C*H*$_3$)$_2$), 0.35 (3H, s, Si(C*H*$_3$)$_2$); **^{13}C{^{1}H} NMR** ($CDCl_3$, 101 MHz) δ 143.0, 141.0, 137.6, 134.0, 128.6, 127.4, 124.4, 124.2, 85.5, 42.8, 30.4, -0.9 (2C); **HRMS** (ESI) calc. for $C_{13}H_{17}IOSiNa$ ([M+Na]$^+$) 366.9986, found 366.9988.

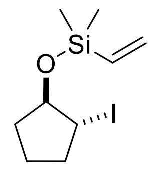

(2-Iodocyclopentyloxy)dimethylvinylsilane 4b was synthesized from 2-iodocyclopentanol **3b** (0.24 g, 1.1 mmol, 1.0 equiv.), chlorodimethylvinylsilane (0.20 mL, 1.4 mmol, 1.2 equiv.), 4-dimethylaminopyridine (40 mg, 0.32 mmol, 0.3 equiv.) and Et_3N (0.20 mL, 1.4 mmol, 1.2 equiv.) in dry CH_2Cl_2 (7.3 mL) according to Procedure C. The crude was purified by column chromatography (EtOAc/heptane 5%) to afford (2-iodocyclopentyloxy)dimethylvinylsilane **4b** (0.21 g, 0.70 mmol, 61%) as a colorless oil.

^{1}H NMR (400 MHz, $CDCl_3$) δ 6.14 (1H, dd, *J* = 20.0, 14.9 Hz, Si(C*H*CH$_2$)), 6.03 (1H, dd, *J* = 14.9, 4.2 Hz, Si(CHC*H*$_2$)), 5.79 (1H, dd, *J* = 19.9, 4.2 Hz, Si(CHC*H*$_2$)), 4.43 (1H, dt, *J* = 6.9, 3.8 Hz, C*H*O), 4.03 (1H, dt, *J* = 8.3, 4.4 Hz, C*H*I), 2.33 (1H, dq, *J* = 14.5, 7.4 Hz, C*H*$_2$), 2.05 (2H, dddd, *J* = 21.5, 19.3, 8.2, 6.0 Hz, C*H*$_2$), 1.79 (2H, p, *J* = 8.0 Hz, C*H*$_2$), 1.62–1.48 (1H, m, C*H*$_2$), 0.21 (3H, s, Si(C*H*$_3$)$_2$), 0.20 (3H, s, Si(C*H*$_3$)$_2$); **^{13}C{^{1}H} NMR** ($CDCl_3$, 101 MHz) δ 137.6, 133.5, 82.7, 36.0, 34.8, 32.4, 22.5, -1.4 (2C).

Data consistent with literature values.[24]

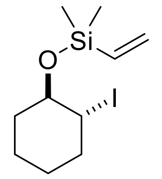

(2-Iodocyclohexyloxy)dimethylvinylsilane 4c was synthesized from 2-iodocyclohexanol **3c** (0.31 g, 1.4 mmol, 1.0 equiv.), chlorodimethylvinylsilane (0.22 mL, 1.6 mmol, 1.2 equiv.), 4-dimethylaminopyridine (33 mg, 0.27 mmol, 0.2 equiv.) and Et_3N (0.22 mL, 1.6 mmol, 1.2 equiv.) in dry CH_2Cl_2 (7.2 mL) according to Procedure C. The crude was purified by column chromatography (EtOAc/heptane 5%) to afford (2-iodocyclohexyloxy)dimethylvinylsilane **4c** (0.35 g, 1.1 mmol, 82%) as a colorless oil.

^{1}H NMR (400 MHz, $CDCl_3$) δ 6.21 (1H, dd, *J* = 20.3, 14.9 Hz, Si(CH$_3$)$_2$C*H*CH$_2$), 6.02 (1H, dd, *J* = 14.9, 3.9 Hz, Si(CH$_3$)$_2$CHC*H*$_2$), 5.81 (1H, dd, *J* = 20.3, 3.9 Hz, Si(CH$_3$)$_2$CHC*H*$_2$), 3.99 (1H, ddd, *J* = 11.1, 8.7, 4.2 Hz, C*H*I), 3.70 (1H, td, *J* = 8.8, 4.4 Hz, C*H*O), 2.49–2.36 (1H, m), 2.05–1.88 (2H, m), 1.78 (1H, qdd, *J* = 7.3, 6.0, 3.7, 1.9 Hz), 1.58–1.44 (1H, m), 1.43–1.17 (3H, m), 0.26 (3H, s, Si(C*H*$_3$)$_2$), 0.24 (3H, s, Si(C*H*$_3$)$_2$); **^{13}C{^{1}H} NMR** ($CDCl_3$, 101 MHz) δ 138.1, 133.3, 76.4, 39.6, 38.0, 35.2, 27.4, 24.0, -1.0, -1.2.

Data consistent with literature values.[24]

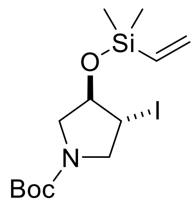

***tert*-Butyl 3-((dimethylvinylsilyl)oxy)-4-iodopyrrolidine-1-carboxylate 4d** was synthesized from *tert*-butyl 3-hydroxy-4-iodopyrrolidine-1-carboxylate **3d** (0.47 g, 1.5 mmol, 1.0 equiv.), chlorodimethylvinylsilane (0.26 mL, 1.8 mmol, 1.2 equiv.), 4-dimethylaminopyridine (51 mg, 0.42 mmol, 0.3 equiv.) and Et_3N (0.26 mL, 1.9 mmol, 1.2 equiv.) in dry CH_2Cl_2 (9.6 mL) according to Procedure C. The crude was purified by column chromatography (EtOAc/heptane 15%) to afford *tert*-butyl 3-((dimethylvinylsilyl)oxy)-4-iodopyrrolidine-1-carboxylate **4d** (0.42 g, 1.1 mmol, 71%) as a light-yellow oil.

R_f 0.47 (EtOAc/heptane 15%); **IR** (thin film, v_{max}/cm^{-1}) 2974, 1702, 1400, 1367, 1255, 1168, 1111, 1081, 1009, 838, 782; **^{1}H NMR** (400 MHz, $CDCl_3$, mixture of rotamers) δ 6.19–6.01 (2H, m, Si(C*H*CH$_2$), Si(CHC*H*$_2$)), 5.80 (1H, dd, *J* = 18.1, 5.9 Hz, Si(CHC*H*$_2$)), 4.44 (1H, td, *J* = 5.4, 2.5 Hz, C*H*O), 4.02 (2H, d, *J* = 10.5 Hz, C*H*I, C*H*$_2$I), 3.89–3.64 (2H, m, C*H*$_2$I, C*H*$_2$CHO), 3.34–3.19 (1H, m, C*H*$_2$CHO), 1.47 (9H, d, *J* = 2.6 Hz, 3 x C*H*$_3$), 0.22 (3H, s, Si(C*H*$_3$)$_2$), 0.21 (3H, s, Si(C*H*$_3$)$_2$); **^{13}C{^{1}H} NMR** ($CDCl_3$, 101 MHz, mixture of rotamers) δ 156.0 (2), 136.8 (2), 134.4 (2), 80.0 (2), 79.3, 78.4, 54.1, 54.0, 52.0, 51.5, 28.6 (6), 26.7 (2), -1.6 (4); **HRMS** (APCI) calc. for $C_8H_{17}INOSi$ ([M-Boc+H]$^+$) 298.0119, 298.0118.

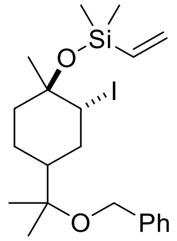

((4-(2-Benzyloxy)propan-2-yl)-2-iodo-1-methylcyclohexyl)oxy)dimethylvinylsilane 4e was synthesized from 4-(2-(benzyloxy)propan-2-yl)-2-iodo-1-methylcyclohexanol **3e** (0.22 g, 0.57 mmol, 1.0 equiv.), chlorodimethylvinylsilane (97 µL, 0.68 mmol, 1.2 equiv.), 4-dimethylaminopyridine (16 mg, 0.13 mmol, 0.2 equiv.) and Et_3N (95 µL, 0.69 mmol, 1.2 equiv.) in dry CH_2Cl_2 (3.1 mL) according to Procedure C. The crude was purified by column chromatography (Et_2O/pentane 2%) to afford ((4-(2-benzyloxy)propan-2-yl)-2-iodo-1-methylcyclohexyl)dimethylvinylsilane **4e** (0.17 g, 0.33 mmol, 63%) as a colorless oil.

R_f 0.34 (Et_2O/pentane 2%); **IR** (thin film, v_{max}/cm^{-1}) 2947, 1454, 1379, 1364, 1252, 1174, 1090, 1051, 1032, 1009, 833, 781, 733, 696; **^{1}H NMR** (400 MHz, $CDCl_3$) δ 7.41–7.20 (5H, m, Ar*H*), 6.15 (1H, dd, *J* = 20.2, 14.8 Hz, Si(C*H*CH$_2$)), 5.95 (1H, dd, *J* = 14.8, 3.8 Hz, Si(CHC*H*$_2$)), 5.76 (1H, dd, *J* = 20.2, 3.9 Hz, Si(CHC*H*$_2$)), 4.46 (3H, q, *J* = 11.1 Hz, C*H*I, OC*H*$_2$), 2.25–1.88 (4H, m, 2 x CHIC*H*$_2$, C*H*C(CH$_3$)$_2$O, C(CH$_3$)(OR)C*H*$_2$), 1.68–1.52 (3H, m, C(CH$_3$)(OR)C*H*$_2$, C*H*$_2$), 1.50 (3H, s, C(C*H*$_3$)(OR)), 1.26 (3H, s, C(C*H*$_3$)$_2$), 1.20 (3H, s, C(C*H*$_3$)$_2$), 0.19 (6H, s, Si(C*H*$_3$)$_2$); **^{13}C{^{1}H} NMR** ($CDCl_3$, 101 MHz) δ 140.1, 139.4, 132.3, 128.4 (2C), 127.5 (2C), 127.1, 76.9, 74.5, 63.2, 44.6, 40.6, 34.4, 33.0, 32.0, 23.7, 23.5, 22.4, 0.7, 0.6; **HRMS** (APCI) calc. for $C_{21}H_{37}INO_2Si$ ([M+NH$_4$]$^+$) 490.1633, found 490.1633.

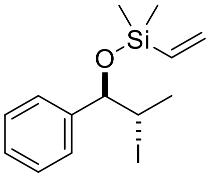

(2-Iodo-1-phenylpropoxy)dimethylvinylsilane 4f was synthesized from 2-iodo-1-phenylpropan-1-ol **3f** (0.20 g, 0.77 mmol, 1.0 equiv.), chlorodimethylvinylsilane (0.16 mL, 1.1 mmol, 1.5 equiv.), 4-dimethylaminopyridine (24 mg, 0.20 mmol, 0.3 equiv.) and Et_3N (0.16 mL, 1.2 mmol, 1.5 equiv.) in dry CH_2Cl_2 (5.1 mL) according to Procedure C. The crude was purified by column chromatography (Et_2O/pentane 3%) to afford (2-iodo-1-phenylpropoxy)dimethylvinylsilane **4f** (0.22 g, 0.63 mmol, 82%) as a colorless oil.

R_f 0.51 (Et_2O /heptane 3%); **IR** (liquid, ν_{max}/cm^{-1}) 2954, 1497, 1455, 1408, 1252, 1072, 1051, 1008, 959, 834, 784, 747, 698; **^{1}H NMR** (400 MHz, $CDCl_3$) δ 7.35–7.27 (5H, m, Ar*H*), 6.09 (1H, dd, *J* = 20.0, 14.9 Hz, SiC*H*CH$_2$), 5.97 (1H, dd, *J* = 14.8, 4.2 Hz, SiCHC*H*$_2$'), 5.74 (1H, dd, *J* = 20.0, 4.2 Hz, SiCHC*H*$_2$), 4.87 (1H, d, *J* = 4.7 Hz, C*H*$_2$O), 4.32 (1H, qd, *J* = 6.9, 4.7 Hz, C*H*I), 1.76 (3H, d, *J* = 6.9 Hz, C*H*$_3$), 0.17 (3H, s, Si(C*H*$_3$)$_2$), 0.12 (3H, s, Si(C*H*$_3$)$_2$); **^{13}C{^{1}H} NMR** ($CDCl_3$, 101 MHz) δ 141.9, 137.4, 133.5, 128.1 (2C), 127.9, 127.0 (2C), 80.2, 34.6, 22.3, -1.3, -1.5; **HRMS** (APCI) calc. for $C_{13}H_{19}IOSiNa$ ([M+Na]$^+$) 369.0142, found 369.0141.

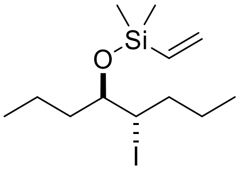

((5-Iodooctan-4-yl)oxy)dimethylvinylsilane 4g was synthesized from 5-iodooctan-4-ol **3g** (0.31 g, 1.2 mmol, 1.0 equiv.), chlorodimethylvinylsilane (0.20 mL, 1.4 mmol, 1.2 equiv.), 4-dimethylaminopyridine (30 mg, 0.24 mmol, 0.2 equiv.) and Et_3N (0.20 mL, 1.4 mmol, 1.2 equiv.) in dry CH_2Cl_2 (6.4 mL) according to Procedure C. The crude was purified by column chromatography (Et_2O/pentane 0-1%) to afford ((5-iodooctan-4-yl)oxy)dimethylvinylsilane **4g** (0.36 g, 1.1 mmol, 87%) as a colorless oil.

R_f 0.55 (Et_2O/pentane 1%); **IR** (liquid, ν_{max}/cm^{-1}) 2959, 2936, 2873, 1465, 1410, 1257, 1149, 1127, 1078, 1041, 1011, 963, 907, 829, 788, 702; **^{1}H NMR** (400 MHz, $CDCl_3$) δ 6.18 (1H, dd, J = 20.2, 14.9 Hz, Si(C*H*CH$_2$)), 6.02 (1H, dd, J = 14.8, 4.0 Hz, Si(CHC*H*$_2$)), 5.80 (1H, dd, J = 20.2, 4.0 Hz, Si(CHC*H*$_2$)), 4.13 (1H, dt, J = 10.0, 3.7 Hz, C*H*I), 3.33 (1H, ddd, J = 8.2, 4.1, 3.0 Hz, C*H*O), 1.87–1.71 (1H, m, CHIC*H*$_2$), 1.68–1.54 (3H, m, CHIC*H*$_2$, CHOC*H*$_2$, CHICH$_2$C*H*$_2$), 1.54–1.42 (2H, m, CHOC*H*$_2$, CHOCH$_2$C*H*$_2$), 1.39–1.30 (2H, m, CHOCH$_2$C*H*$_2$, CHICH$_2$C*H*$_2$), 0.92 (6H, m, 2 x C*H*$_3$), 0.24 (3H, s, Si(C*H*$_3$)$_2$), 0.23 (3H, s, Si(C*H*$_3$)$_2$); **^{13}C{^{1}H} NMR** ($CDCl_3$, 101 MHz) δ 137.9, 133.4, 76.0, 46.3, 37.9, 37.8, 23.2, 18.8, 14.3, 13.4, -1.08, -1.14.; **HRMS** (ESI) calc. for $C_{12}H_{25}IOSiNa$ ([M+Na]$^+$) 363.0612, found 363.0611.

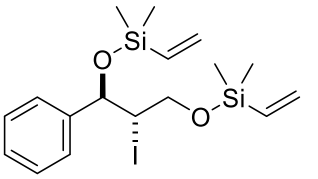

6-Iodo-3,3,9,9-tetramethyl-5-phenyl-4,8-dioxa-3,9-disilaundeca-1,10-diene 4h was synthesized from 2-iodo-1-phenyl-propan-1,3-diol **3h** (0.33 g, 1.2 mmol, 1.0 equiv.), chlorodimethylvinylsilane (0.36 mL, 2.6 mmol, 2.2 equiv.), 4-dimethylaminopyridine (29 mg, 0.24 mmol, 0.2 equiv.) and Et_3N (0.36 mL, 2.6 mmol, 2.2 equiv.) in dry CH_2Cl_2 (7.6 mL) according to Procedure C. The crude was purified by column chromatography (Et_2O/pentane 1%) to afford 6-iodo-3,3,9,9-tetramethyl-5-phenyl-4,8-dioxa-3,9-disilaundeca-1,10-diene **4h** (0.19 g, 0.43 mmol, 36%) as a colorless oil.

R*f* 0.30 (Et_2O/pentane 1%); **IR** (thin film, v_{max}/cm^{-1}) 2960, 1409, 1253, 1127, 1096, 1072, 1052, 1009, 959, 857, 838, 787, 701; **^{1}H NMR** (400 MHz, $CDCl_3$) δ 7.37–7.27 (5H, m, Ar*H*), 6.20–5.90 (4H, m, 2 x Si(C*H*CH$_2$), 2 x Si(CHC*H*$_2$)), 5.75 (2H, ddd, *J* = 30.1, 19.8, 4.4 Hz, 2 x Si(CHC*H*$_2$)), 4.89 (1H, d, *J* = 5.9 Hz, C*H*O), 4.33 (1H, q, *J* = 5.8 Hz, C*H*I), 3.94 (1H, dd, *J* = 11.3, 6.0 Hz, CHIC*H*$_2$O), 3.71 (1H, dd, *J* = 11.3, 5.5 Hz, CHIC*H*$_2$O), 0.20 (6H, s, Si(C*H*$_3$)$_2$), 0.12 (3H, s, Si(C*H*$_3$)$_2$), 0.09 (3H, s, Si(C*H*$_3$)$_2$); **^{13}C{^{1}H} NMR** ($CDCl_3$, 101 MHz) δ 141.8, 137.4, 137.2, 133.7, 133.4, 127.9 (3C), 127.4 (2C), 75.5, 64.8, 43.9, -1.4, -1.6, -1.9 (2C); **HRMS** (APCI) calc. for $C_{17}H_{28}IO_2Si_2$ ([M+H]$^+$) 447.0667, found 447.0667.

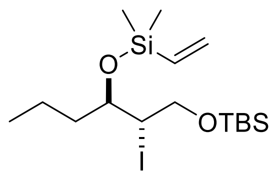

(1-((*tert*-Butyldimethylsilyl)oxy)-2-iodohexan-3-oxy)dimethylvinylsilane 4i was synthesized from 1-((*tert*-butyldimethylsilyl)oxy)-2-iodohexan-3-ol **3i** (0.19 g, 0.52 mmol, 1.0 equiv.), chlorodimethylvinylsilane (85 µL, 0.70 mmol, 1.4 equiv.), 4-dimethylaminopyridine (16 mg, 0.13 mmol, 0.2 equiv.) and Et_3N (98 µL, 0.70 mmol, 1.4 equiv.) in dry CH_2Cl_2 (3.7 mL) according to Procedure C. The crude was purified by column chromatography (Et_2O/pentane 2%) to afford (1-((*tert*-butyldimethylsilyl)oxy)-2-iodohexan-3-oxy)dimethylvinylsilane **4i** (0.15 g, 0.33 mmol, 65%) as a colorless oil.

R*f* 0.60 (Et_2O/pentane 2%); **IR** (thin film, v_{max}/cm^{-1}) 2957, 2930, 2861, 1473, 1466, 1254, 1100, 1090, 1059, 1054, 1050, 838, 785, 779; **^{1}H NMR** (400 MHz, $CDCl_3$) δ 6.17 (1H, dd, *J* = 20.2, 14.8 Hz, Si(C*H*CH$_2$)), 6.02 (1H, dd, *J* = 14.9, 3.9 Hz, Si(CHC*H*$_2$)), 5.80 (1H, dd, *J* = 20.2, 4.0 Hz, Si(CHC*H*$_2$)), 4.22 (1H, ddd, *J* = 7.9, 5.7, 4.1 Hz, C*H*I), 3.88 (1H, dd, *J* = 10.9, 5.7 Hz, CHIC*H*$_2$O), 3.79 (1H, dd, *J* = 10.9, 8.1 Hz, CHIC*H*$_2$O), 3.57 (1H, dt, *J* = 8.2, 3.1 Hz, C*H*O), 1.66–1.40 (3H, m, 2 x CH$_3$C*H*$_2$, CH$_3$CH$_2$C*H*$_2$), 1.38–1.22 (1H, m, CH$_3$CH$_2$C*H*$_2$), 0.90 (12H, s, C*H*$_3$, C(C*H*$_3$)$_3$), 0.23 (3H, s, Si(C*H*$_3$)$_2$(CHCH$_2$)), 0.22 (3H, s, Si(C*H*$_3$)$_2$(CHCH$_2$)), 0.07 (3H, s, Si(C*H*$_3$)$_2$C(CH$_3$)$_3$), 0.06 (3H, s, Si(C*H*$_3$)$_2$C(CH$_3$)$_3$); **^{13}C{^{1}H} NMR** ($CDCl_3$, 101 MHz) δ 137.9, 133.4, 71.9, 66.1, 44.5, 37.5, 26.0 (3C), 18.6, 18.3, 14.2, -1.2, -1.3, -5.1, -5.3; **HRMS** (APCI) calc. for $C_{16}H_{36}IO_2Si_2$ ([M+H]$^+$) 443.1293, found 443.1292.

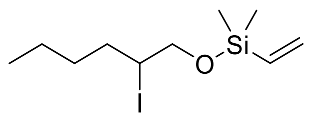

((2-Iodohexyl)oxy)dimethylvinylsilane 4j was synthesized from 2-iodohexan-1-ol **3j** (0.11 g, 0.47 mmol, 1.0 equiv.), chlorodimethylvinylsilane (80 µL, 0.56 mmol, 1.2 equiv.), 4-dimethylaminopyridine (13 mg, 0.11 mmol, 0.2 equiv.) and Et_3N (80 µL, 0.58 mmol, 1.2 equiv.) in dry CH_2Cl_2 (2.5 mL) according to Procedure C. The crude was purified by column chromatography (Et_2O/pentane 1%) to afford ((2-iodohexyl)oxy)dimethylvinylsilane **4j** (0.11 g, 0.35 mmol, 76%) as a colorless oil.

R*f* 0.34 (Et_2O/pentane 1%); **IR** (thin film, v_{max}/cm^{-1}) 2957, 2931, 2871, 2860, 1408, 1252, 1077, 835, 783, 701; **^{1}H NMR** (400 MHz, $CDCl_3$) δ 6.21–6.07 (1H, m, Si(C*H*CH$_2$)), 6.04 (1H, ddd, *J* = 15.2, 4.8, 2.3 Hz, Si(CHC*H*$_2$)), 5.80 (1H, ddd, *J* = 19.7, 4.5, 2.1 Hz, Si(CHC*H*$_2$)), 4.14–3.99 (1H, m, C*H*I), 3.87 (1H, dd, *J* = 11.1, 5.0 Hz, C*H*$_2$O), 3.77–3.64 (1H, m, C*H*$_2$O), 1.85 (1H, d, *J* = 13.1 Hz, C*H*$_2$CHI), 1.79–1.63 (1H, m, C*H*$_2$CHI), 1.58–1.48 (1H, m, CH$_3$C*H*$_2$), 1.44–1.28 (3H, m, CH$_3$C*H*$_2$, 2 x C*H*$_3$CH$_2$), 0.92 (3H, dt, *J* = 7.9, 3.9 Hz, C*H*$_3$), 0.21 (6H, s, 2 x Si(C*H*$_3$)$_2$); **^{13}C{^{1}H} NMR** ($CDCl_3$, 101 MHz) δ 137.1, 133.8, 68.9, 38.1, 35.8, 31.6, 22.1, 14.1, -1.9, -2.0; **HRMS** (APCI) calculated for $C_{10}H_{21}IOSiNa$ ([M+Na]$^+$) 335.0299, found 335.0299.

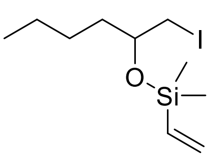

((1-Iodohexan-2-yl)oxy)dimethylvinylsilane 4k was synthesized from 1-iodohexan-2-ol **3k** (0.30 g, 1.3 mmol, 1.0 equiv.), chlorodimethylvinylsilane (0.23 mL, 1.6 mmol, 1.2 equiv.), 4-dimethylaminopyridine (34 mg, 0.27 mmol, 0.2 equiv.) and Et_3N (0.22 mL, 1.6 mmol, 1.2 equiv.) in dry CH_2Cl_2 (7.2 mL) according to Procedure C. The crude was purified by column chromatography (Et_2O/pentane 1%) to afford ((1-iodohexan-2-yl)oxy)dimethylvinylsilane **4k** (0.34 g, 1.1 mmol, 82%) as a colorless oil.

R*f* 0.38 (Et_2O /pentane 1%); **IR** (liquid, ν_{max}/cm^{-1}) 2959, 2936, 2862, 1410, 1257, 1037, 1011, 963, 940, 840, 817, 784, 698; **^{1}H NMR** (400 MHz, $CDCl_3$) δ 6.17 (1H, dd, *J* = 20.1, 14.9 Hz, Si(C*H*CH$_2$)), 6.03 (1H, dd, *J* = 14.8, 4.0 Hz, Si(CHC*H*$_2$)), 5.80 (1H, dd, *J* = 20.1, 4.0 Hz, Si(CHC*H*$_2$)), 3.60 (1H, dq, *J* = 7.2, 5.1 Hz, C*H*O), 3.25–3.12 (2H, m, C*H*$_2$I), 1.72–1.57 (1H, m, C*H*$_2$CHO), 1.57–1.42 (1H, m, C*H*$_2$CHO), 1.42–1.18 (4H, m, C*H*$_2$CH$_2$CH$_3$, CH$_2$C*H*$_2$CH$_3$), 0.90 (3H, dd, *J* = 7.8, 6.2 Hz, CH$_2$CH$_2$C*H*$_3$), 0.23 (6H, s, 2 x Si(C*H*$_3$)$_2$); **^{13}C{^{1}H} NMR** ($CDCl_3$, 101 MHz) δ 137.7, 133.6, 72.2, 36.7, 27.6, 22.7, 14.2, 14.0, -1.2, -1.3; **HRMS** (APCI) calc. for $C_{10}H_{21}IOSiNa$ ([M+Na]$^+$) 335.0299, found 335.0299.

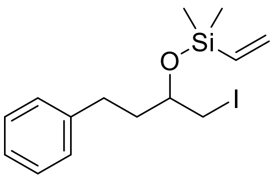

((1-Iodo-4-phenylbutan-2-yl)oxy)dimethylvinylsilane 4l was synthesized from 1-iodo-4-phenylbutan-2-ol **3l** (0.48 g, 1.7 mmol, 1.0 equiv.), chlorodimethylvinylsilane (0.30 mL, 2.1 mmol, 1.2 equiv.), 4-dimethylaminopyridine (42 mg, 0.35 mmol, 0.2 equiv.) and Et_3N (0.29 mL, 2.1 mmol, 1.2 equiv.) in dry CH_2Cl_2 (9.3 mL) according to Procedure C. The crude was purified by column chromatography (Et_2O/pentane 3%) to afford ((1-iodo-4-phenylbutan-2-yl)oxy)dimethylvinylsilane **4l** (0.52 g, 1.5 mmol, 83%) as a colorless oil.

R*f* 0.45 (Et_2O /pentane 3%); **IR** (liquid, ν_{max}/cm^{-1}) 2950, 1595, 1497, 1455, 1408, 1251, 1052, 959, 835, 783, 698; **^{1}H NMR** (400 MHz, $CDCl_3$) δ 7.33–7.26 (2H, m, Ar*H*), 7.23–7.15 (3H, m, Ar*H*), 6.18 (1H, dd, *J* = 20.1, 14.8 Hz, SiC*H*CH$_2$), 6.04 (1H, dd, *J* = 14.9, 4.0 Hz, SiCHC*H*$_2$), 5.82 (1H, dd, *J* = 20.1, 4.0 Hz, SiCHC*H*$_2$), 3.71–3.62 (1H, m, C*H*O), 3.22 (2H, d, *J* = 5.4 Hz, C*H*$_2$I), 2.71 (1H, ddd, *J* = 13.7, 10.5, 5.7 Hz, CC*H*$_2$), 2.59 (1H, ddd, *J* = 13.7, 10.5, 5.9 Hz, CC*H*$_2$), 1.98 (1H, dddd, *J* = 13.7, 10.4, 5.9, 4.3 Hz, C*H*$_2$CHO), 1.86 (1H, dddd, *J* = 13.8, 10.5, 7.3, 5.7 Hz, C*H*$_2$CHO), 0.25 (3H, s, Si(C*H*$_3$)$_2$), 0.24 (3H, s, Si(C*H*$_3$)$_2$); **^{13}C{^{1}H} NMR** ($CDCl_3$, 101 MHz) δ 141.8, 137.6, 133.8, 128.6 (2C), 128.5 (2C), 126.1, 71.7, 38.7, 31.7, 13.4, -1.2, -1.3; **HRMS** (APCI) calc. for $C_{14}H_{21}IOSiNa$ ([M+Na]$^+$) 383.0299, found 383.0298.

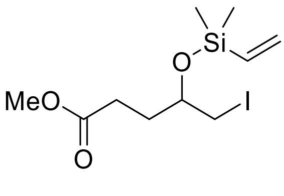

Methyl 4-((dimethylvinylsilyl)oxy)-5-iodopentanoate 4m was synthesized from methyl 4-hydroxy-5-iodopentanoate **3m** (0.34 g, 1.3 mmol, 1.0 equiv.), chlorodimethylvinylsilane (0.24 mL, 1.7 mmol, 1.4 equiv.), 4-dimethylaminopyridine (44 mg, 0.36 mmol, 0.3 equiv.) and Et_3N (0.24 mL, 1.7 mmol, 1.3 equiv.) in dry CH_2Cl_2 (8.4 mL) according to Procedure C. The crude was purified by column chromatography (Et_2O/pentane 10%) to afford methyl 4-((dimethylvinylsilyl)oxy)-5-iodopentanoate **4m** (0.23 g, 0.66 mmol, 50%) as a colorless oil.

R_f 0.29 (Et_2O /pentane 10%); **IR** (liquid, ν_{max}/cm^{-1}) 2954, 1736, 1438, 1252, 1171, 1100, 1066, 1008, 960, 837, 814, 783, 700; **^{1}H NMR** (400 MHz, $CDCl_3$) δ 6.15 (1H, dd, *J* = 19.9, 14.9 Hz, Si(C*H*CH_2)), 6.04 (1H, dd, *J* = 14.9, 4.2 Hz, Si(CHC*H*$_2$)), 5.80 (1H, dd, *J* = 19.9, 4.2 Hz, Si(CHC*H*$_2$)), 3.67 (4H, s, OC*H*$_3$, C*H*OH), 3.17 (2H, dd, *J* = 5.5, 2.6 Hz, C*H*$_2$I), 2.37 (2H, td, *J* = 7.8, 3.0 Hz, COC*H*$_2$), 2.02 (1H, dtd, *J* = 15.5, 7.7, 3.6 Hz, C*H*$_2$), 1.81 (1H, dq, *J* = 14.5, 7.4 Hz, C*H*$_2$), 0.22 (6H, s, 2 x Si(C*H*$_3$)$_2$); **^{13}C{^{1}H} NMR** ($CDCl_3$, 101 MHz) δ 173.8, 137.3, 134.0, 70.9, 51.8, 31.8, 29.8, 12.6, -1.4, -1.5; **HRMS** (APCI) cal. for $C_{10}H_{19}IO_3Si$ ([M+H]$^+$) 343.0221, found 343.0220.

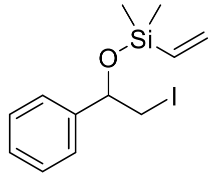

(2-Iodo-1-phenylethoxy)dimethylvinylsilane 4n was synthesized from 2-iodo1-phenylethan-1-ol **3n** (0.26 g, 1.0 mmol, 1.0 equiv.), chlorodimethylvinylsilane (0.20 mL, 1.4 mmol, 1.4 equiv.), 4-dimethylaminopyridine (36 mg, 0.30 mmol, 0.3 equiv.) and Et_3N (0.20 mL, 1.4 mmol, 1.4 equiv.) in dry CH_2Cl_2 (6.7 mL) according to Procedure C. The crude was purified by column chromatography (Et_2O/pentane 1%) to afford (2-iodo-1-phenylethoxy)dimethylvinylsilane **4n** (0.21 g, 0.62 mmol, 61%) as a light-yellow oil.

R_f 0.38 (Et_2O /pentane 1%); **IR** (liquid, ν_{max}/cm^{-1}) 2961, 1407, 1252, 1098, 997, 889, 836, 811, 784, 699; **^{1}H NMR** (400 MHz, $CDCl_3$) δ 7.39–7.21 (5H, m, Ar*H*), 6.09 (1H, dd, *J* = 19.9, 14.9 Hz, Si(C*H*CH_2)), 5.98 (1H, dd, *J* = 14.9, 4.4 Hz, Si(CHC*H*$_2$)), 5.75 (1H, dd, *J* = 19.8, 4.3 Hz, Si(CHC*H*$_2$)), 4.80 (1H, dd, *J* = 7.6, 4.9 Hz, C*H*O), 3.42–3.26 (2H, m, C*H*$_2$I), 0.18 (3H, s, Si(C*H*$_3$)$_2$), 0.13 (3H, s, Si(C*H*$_3$)$_2$); **^{13}C{^{1}H} NMR** ($CDCl_3$, 101 MHz) δ 142.7, 137.3, 133.8, 128.6 (2C), 128.2, 126.2 (2C), 75.6, 14.8, -1.3, -1.5; **HRMS** (ESI) calc. for $C_{12}H_{17}IOSiNa$ ([M+Na]$^+$) 354.9986, found 354.9986.

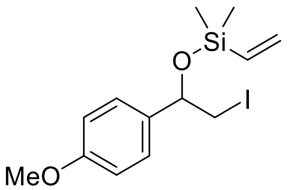

(2-Iodo-1-(4-methoxyphenyl)ethoxy)dimethylvinylsilane 4o was synthesized from 2-iodo-1-(4-methoxyphenyl)ethan-1-ol **3o** (1.5 g, 5.2 mmol, 1.0 equiv.), chlorodimethylvinylsilane (0.95 mL, 6.7 mmol, 1.3 equiv.), 4-dimethylaminopyridine (0.18 g, 1.4 mmol, 0.3 equiv.) and Et_3N (0.95 mL, 6.8 mmol, 1.3 equiv.) in dry CH_2Cl_2 (33 mL) according to Procedure C. The crude was purified by column chromatography (Et_2O/pentane 5%) to afford (2-iodo-1-(4-methoxyphenyl)ethoxy)dimethylvinylsilane **4o** (0.98 g, 2.7 mmol, 52%) as a colorless oil.

R_f 0.29 (Et_2O /pentane 5%); **IR** (liquid, ν_{max}/cm^{-1}) 2956, 1611, 1512, 1249, 1171, 1100, 1037, 999, 893, 837, 787; **^{1}H NMR** (400 MHz, $CDCl_3$) δ 7.24 (2H, d, *J* = 8.6 Hz, Ar*H*), 6.87 (2H, d, *J* = 8.5 Hz, Ar*H*), 6.08 (1H, dd, *J* = 19.9, 14.8 Hz, Si(C*H*CH_2)), 5.98 (1H, dd, *J* = 14.9, 4.4 Hz, Si(CHC*H*$_2$)), 5.75 (1H, dd, *J* = 19.8, 4.4 Hz, Si(CHC*H*$_2$)), 4.76 (1H, t, *J* = 6.4 Hz, C*H*O), 3.81 (3H, s, OC*H*$_3$), 3.32 (2H, d, *J* = 6.4 Hz, C*H*$_2$I), 0.17 (3H, s, Si(C*H*$_3$)$_2$), 0.11 (3H, s, Si(C*H*$_3$)$_2$); **^{13}C{^{1}H} NMR** ($CDCl_3$, 101 MHz) δ 159.4, 137.4, 134.8, 133.6, 127.3 (2C), 113.9 (2C), 75.1, 55.4, 15.0, -1.3, -1.5; **HRMS** (ESI) calc. for $C_{13}H_{19}IO_2SiNa$ ([M+Na]$^+$) 385.0091, found 385.0091.

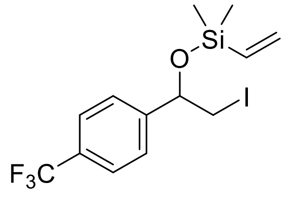

(2-Iodo-1-(4-(trifluoromethyl)phenyl)ethoxy)dimethylvinylsilane 4p was synthesized from 2-iodo-1-(4-(trifluromethyl)phenyl)ethan-1-ol **3p** (0.32 g, 1.0 mmol, 1.0 equiv.), chlorodimethylvinylsilane (0.17 mL, 1.2 mmol, 1.2 equiv.), 4-dimethylaminopyridine (27 mg, 0.22 mmol, 0.2 equiv.) and Et_3N (0.17 mL, 1.2 mmol, 1.2 equiv.) in dry CH_2Cl_2 (6.5 mL) according to Procedure C. The crude was purified by column chromatography (Et_2O/pentane 2%) to afford (2-iodo-1-(4-(trifluoromethyl)phenyl)ethoxy)dimethylvinylsilane **4p** (0.29 g, 0.72 mmol, 71%) as a colorless oil.

R*f* 0.53 (Et_2O /pentane 2%); **IR** (liquid, ν_{max}/cm^{-1}) 2960, 1412, 1326, 1255, 1168, 1128, 1103, 1069, 890, 840, 790; **^{1}H NMR** (400 MHz, $CDCl_3$) δ 7.60 (2H, d, *J* = 8.1 Hz, Ar*H*), 7.49–7.40 (2H, m, Ar*H*), 6.16–5.94 (2H, m, Si(C*H*CH$_2$), Si(CHC*H*$_2$)), 5.76 (1H, dd, *J* = 19.1, 5.0 Hz, Si(CHC*H*$_2$)), 4.83 (1H, dd, *J* = 7.2, 5.0 Hz, C*H*O), 3.39–3.26 (2H, m, C*H*$_2$I), 0.21 (3H, s, Si(C*H*$_3$)$_2$), 0.15 (3H, s, Si(C*H*$_3$)$_2$); **^{13}C{^{1}H} NMR** ($CDCl_3$, 101 MHz) δ 146.6, 136.9, 134.2, 130.3 (q, $^2J_{C–F}$ = 32.3 Hz), 126.5 (2C), 125.5 (2C, q, $^3J_{C–F}$ = 3.8 Hz), 124.3 (q, $^1J_{C–F}$ = 272.2 Hz), 74.7, 13.8, -1.4, -1.6; **HRMS** (APCI) calc. for $C_{13}H_{17}F_3IOSi$ ($[M+H]^+$) 401.0040, found 401.0038.

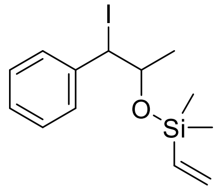

((1-Iodo-1-phenylpropan-2-yl)oxy)dimethylvinylsilane 4q was synthesized from 1-iodo-1-phenylpropan-2-ol **3q** (0.31 g, 1.2 mmol, 1.0 equiv.), chlorodimethylvinylsilane (0.21 mL, 1.4 mmol, 1.2 equiv.), 4-dimethylaminopyridine (30 mg, 0.25 mmol, 0.2 equiv.) and Et_3N (0.20 mL, 1.5 mmol, 1.2 equiv.) in dry CH_2Cl_2 (6.5 mL) according to Procedure C. The crude was purified by column chromatography (Et_2O/pentane 5%) to afford ((1-iodo-1-phenylpropan-2-yl)oxy)dimethylvinylsilane **4q** (0.14 g, 0.40 mmol, 34%) as a light-yellow oil.

R*f* 0.59 (Et_2O /pentane 5%); **IR** (liquid, ν_{max}/cm^{-1}) 2953, 1453, 1376, 1250, 1090, 1076, 1059, 987, 960, 828, 783, 695; **^{1}H NMR** (400 MHz, $CDCl_3$) δ 7.48–7.15 (5H, m, Ar*H*), 6.10–5.91 (2H, m, Si(C*H*CH$_2$), Si(CHC*H*$_2$)), 5.69 (1H, dd, *J* = 17.4, 6.8 Hz, Si(CHC*H*$_2$)), 4.95 (1H, d, *J* = 6.7 Hz, C*H*I), 4.12 (1H, p, *J* = 6.2 Hz, C*H*O), 1.37 (3H, d, *J* = 6.0 Hz, C*H*$_3$), 0.06 (3H, s, Si(C*H*$_3$)$_2$), 0.04 (3H, s, Si(C*H*$_3$)$_2$); **^{13}C{^{1}H} NMR** ($CDCl_3$, 101 MHz) δ 141.5, 137.6, 133.4, 129.3 (2C), 128.3 (2C), 127.9, 73.5, 41.0, 23.2, -1.4, -1.7; **HRMS** (APCI) calc. for $C_{13}H_{20}IOSi$ ($[M+H]^+$) 347.0323, found 347.0322.

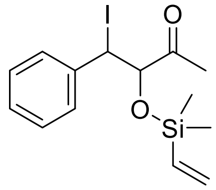

3-((Dimethylvinylsilyl)oxy)-4-iodo-4-phenylbutan-2-one 4r was synthesized from 3-hydroxy-4-iodo-4-phenylbutan-2-one **3r** (0.35 g, 1.2 mmol, 1.0 equiv.), chlorodimethylvinylsilane (0.21 mL, 1.4 mmol, 1.2 equiv.), 4-dimethylaminopyridine (30 mg, 0.24 mmol, 0.2 equiv.) and Et_3N (0.20 mL, 1.4 mmol, 1.2 equiv.) in dry CH_2Cl_2 (6.4 mL) according to Procedure C. The crude was purified by column chromatography (Et_2O/pentane 5-10%) to afford an inseparable 87:13 diastereomers mixture of 3-((dimethylvinylsilyl)oxy)-4-iodo-4-phenylbutan-2-one **4r** (0.18 g, 0.49 mmol, 41%) as a yellow oil. *Note: the NMR data were extracted from the inseparable mixture data.*

R_f 0.59 (Et_2O /pentane 5%); **IR** (liquid, ν_{max}/cm^{-1}) 2961, 1724, 1674, 1408, 1354, 1254, 1123, 1074, 871, 842, 789, 698; **HRMS** (APCI) calc. for $C_{14}H_{20}IO_2Si$ ([M+H]$^+$) 375.0272, found 375.0269.

major: **^{1}H NMR** (400 MHz, $CDCl_3$) δ 7.44 (2H, dd, *J* = 7.9, 1.8 Hz, Ar*H*), 7.30–7.23 (3H, m, Ar*H*), 6.04 (1H, d, *J* = 2.7 Hz, Si(C*H*CH$_2$)), 6.01 (1H, d, *J* = 1.9 Hz, Si(CHC*H*$_2$)), 5.79–5.68 (1H, m, Si(CHC*H*$_2$)), 5.20 (1H, d, *J* = 6.3 Hz, C*H*I), 4.43 (1H, d, *J* = 6.2 Hz, C*H*O), 1.96 (3H, s, C*H*$_3$), 0.17 (3H, s, Si(C*H*$_3$)$_2$), 0.16 (3H, s, Si(C*H*$_3$)$_2$); **^{13}C{^{1}H} NMR** ($CDCl_3$, 101 MHz) δ 207.7, 139.5, 136.3, 134.6, 129.4 (2C), 128.6, 128.5 (2C), 84.0, 31.3, 26.0, -1.6, -1.8.

minor: **^{1}H NMR** (400 MHz, $CDCl_3$) δ 7.81-7.79 (2H, m, Ar*H*), 7.40-7.22 (3H, m, Ar*H*), 6.63 (1H, s, C*H*O), 6.35-6.26 (1H, m, Si(C*H*CH$_2$)), 5.98-5.97 (1H, m, Si(CHC*H*$_2$)), 5.82-5.81 (1H, m, Si(CHC*H*$_2$)), 5.78-5.70 (1H, m, C*H*I), 2.44 (3H, s, C*H*$_3$), 0.36 (6H, s, Si(C*H*$_3$)$_2$); **^{13}C{^{1}H} NMR** ($CDCl_3$, 101 MHz) δ 195.5, 147.7, 138.6, 134.4, 132.6, 130.2 (2C), 128.6, 120.6, 77.4, 31.3, 25.3, -0.0 (2C).

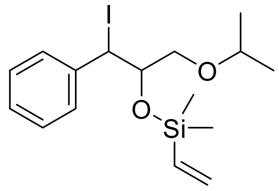

((1-Iodo-3-isopropoxy-1-phenylpropan-2-yl)oxy)dimethylvinylsilane 4s was synthesized from 1-iodo-3-isopropoxy-1-phenylpropan-2-ol **3s** (0.26 g, 0.82 mmol, 1.0 equiv.), chlorodimethylvinylsilane (0.14 mL, 1.0 mmol, 1.2 equiv.), 4-dimethylaminopyridine (23 mg, 0.20 mmol, 0.2 equiv.) and Et_3N (0.14 mL, 1.0 mmol, 1.2 equiv.) in dry CH_2Cl_2 (4.4 mL) according to Procedure C. The crude was purified by column chromatography (Et_2O/pentane 1.5%) to afford a separable 80:20 diastereomers mixture of ((1-iodo-3-isopropoxy-1-phenylpropan-2-yl)oxy)dimethylvinylsilane **4s** (0.12 g, 0.29 mmol, 35%) as a colorless oil.

R_f 0.29 (Et_2O/pentane 1.5%); **IR** (thin film, ν_{max}/cm^{-1}) 2971, 1251, 1114, 1074, 960, 826, 784, 696; **HRMS** (APCI) calc. for $C_{16}H_{26}IO_2Si$ ([M+Na]$^+$) 405.0741, found 405.0742.

major: **^{1}H NMR** (400 MHz, $CDCl_3$) δ 7.49–7.38 (2H, m, Ar*H*), 7.30–7.17 (3H, m, Ar*H*), 6.10 (1H, dd, *J* = 20.1, 14.9 Hz, Si(C*H*CH$_2$)), 5.97 (1H, dd, *J* = 14.8, 4.2 Hz, Si(CHC*H*$_2$)), 5.72 (1H, dd, *J* = 20.1, 4.2 Hz, Si(CHC*H*$_2$)), 5.25 (1H, d, *J* = 5.7 Hz, C*H*I), 4.27 (1H, q, *J* = 5.6 Hz, C*H*O), 3.49 (1H, dq, *J* = 12.2, 5.9 Hz, C*H*(CH$_3$)$_2$), 3.38 (2H, d, *J* = 5.5 Hz, C*H*$_2$O), 1.12 (6H, dd, *J* = 10.1, 6.1 Hz, CH(C*H*$_3$)$_2$), 0.15 (3H, s, Si(C*H*$_3$)$_2$), 0.14 (3H, s, Si(C*H*$_3$)$_2$); **^{13}C{^{1}H} NMR** ($CDCl_3$, 101 MHz) δ 140.9, 137.9, 133.3, 129.5 (2C), 128.2 (2C), 127.9, 77.0, 72.2, 70.4, 34.4, 22.1 (2C), -1.1, -1.4.

minor: **^{1}H NMR** (400 MHz, $CDCl_3$) δ 7.47–7.38 (2H, m, Ar*H*), 7.31–7.17 (3H, m, Ar*H*), 6.06 (1H, dd, *J* = 19.8, 14.9 Hz, Si(C*H*CH$_2$)), 5.96 (1H, dd, *J* = 14.9, 4.5 Hz, Si(CHC*H*$_2$)), 5.74 (1H, dd, *J* = 19.7, 4.5 Hz, Si(CHC*H*$_2$)), 5.25 (1H, d, *J* = 5.4 Hz, C*H*I), 3.55–3.42 (2H, m, C*H*O, C*H*(CH$_3$)$_2$), 3.40–3.21 (2H, m, C*H*$_2$O), 1.10 (6H, dd, *J* = 10.5, 6.2 Hz, CH(C*H*$_3$)$_2$), 0.18 (3H, s, Si(C*H*$_3$)$_2$), 0.16 (3H, s, Si(C*H*$_3$)$_2$); **^{13}C{^{1}H} NMR** ($CDCl_3$, 101 MHz) δ 141.7, 137.9, 133.4, 129.0 (2C), 128.4 (2C), 128.1, 76.2, 72.2, 71.1, 39.7, 22.2, 22.1, -1.1, -1.2.

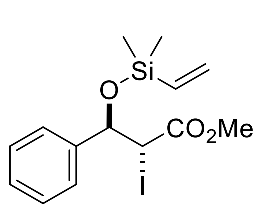

Methyl 3-((dimethylvinylsilyl)oxy)-2-iodo-3-phenylpropanoate 4t was synthesized from methyl 3-hydroxy-2-iodo-3-phenylpropanoate **3t** (0.21 g, 0.68 mmol, 1.0 equiv.), chlorodimethylvinylsilane (0.14 mL, 0.98 mmol, 1.4 equiv.), 4-dimethylaminopyridine (25 mg, 0.20 mmol, 0.3 equiv.) and Et_3N (0.14 mL, 1.0 mmol, 1.5 equiv.) in dry CH_2Cl_2 (4.5 mL) according to Procedure C. The crude was purified by column chromatography (Et_2O/pentane 5%)

to afford methyl 3-((dimethylvinylsilyl)oxy)-2-iodo-3-phenylpropanoate **4t** (0.19 g, 0.48 mmol, 70%) as a colorless oil.

R*f* 0.27 (Et_2O/heptane 5%); **IR** (liquid, $ν_{max}/cm^{-1}$) 2952, 1740, 1252, 1168, 1050, 855, 836, 785, 698; **^{1}H NMR** (400 MHz, $CDCl_3$) δ 7.34 (5H, d, *J* = 2.7 Hz, Ar*H*), 6.01–5.85 (2H, m, Si(C*H*CH$_2$), Si(CHC*H*$_2$)), 5.71–5.57 (1H, m, Si(CHC*H*$_2$)), 5.07 (1H, d, *J* = 10.2 Hz, C*H*O), 4.39 (1H, d, *J* = 10.2 Hz, C*H*I), 3.80 (3H, s, OC*H*$_3$), 0.02 (3H, s, Si(C*H*$_3$)$_2$), -0.02 (3H, s, Si(C*H*$_3$)$_2$); **^{13}C{^{1}H} NMR** ($CDCl_3$, 101 MHz) δ 171.0, 140.4, 136.9, 133.5, 128.8, 128.3 (2C), 127.9 (2C), 77.6, 52.9, 27.5, -1.6, -1.8; **HRMS** (APCI) calculated for $C_{14}H_{20}IO_3Si$ ($[M+H]^+$) 391.0221, found 391.0220.

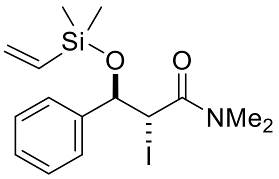

3-((Dimethylvinylsilyl)oxy)-2-iodo-*N*,*N*-dimethyl-3-phenylpropanamide 4u was synthesized from 3-hydroxy-2-iodo-*N*,*N*-dimethyl-3-phenylpropanamide **3u** (0.40 g, 1.3 mmol, 1.0 equiv.), chlorodimethylvinylsilane (0.21 mL, 1.5 mmol, 1.2 equiv.), 4-dimethylaminopyridine (34 mg, 0.28 mmol, 0.2 equiv.) and Et_3N (0.21 mL, 1.5 mmol, 1.2 equiv.) in dry CH_2Cl_2 (8.1 mL) according to Procedure C. The crude was purified by column chromatography (Et_2O/pentane 50%) to afford 3-((dimethylvinylsilyl)oxy)-2-iodo-*N*,*N*-dimethyl-3-phenylpropanamide **4u** (0.37 g, 0.92 mmol, 73%) as a yellow oil.

R*f* 0.46 (Et_2O/pentane 50%); **IR** (liquid, $ν_{max}/cm^{-1}$) 2956, 1655, 1496, 1455, 1400, 1251, 1065, 879, 855, 841, 787, 701; **^{1}H NMR** (400 MHz, $CDCl_3$) δ 7.43–7.26 (5H, m, Ar*H*), 6.03–5.84 (2H, m, Si(C*H*CH$_2$), Si(CHC*H*$_2$)), 5.61 (1H, dd, *J* = 19.2, 5.0 Hz, Si(CHC*H*$_2$)), 5.24 (1H, d, *J* = 9.7 Hz, C*H*O), 4.63 (1H, d, *J* = 9.8 Hz, C*H*I), 3.09 (3H, s, NC*H*$_3$), 3.00 (3H, s, NC*H*$_3$), 0.03 (3H, s, Si(C*H*$_3$)$_2$), 0.02 (3H, s, Si(C*H*$_3$)$_2$); **^{13}C{^{1}H} NMR** ($CDCl_3$, 101 MHz) δ 169.6, 141.6, 137.5, 133.1, 128.4 (2C), 128.1, 127.9 (2), 77.5, 38.0, 36.7, 27.5, -1.5, -1.6; **HRMS** (APCI) calc. for $C_{15}H_{23}INO_2Si$ ($[M+H]^+$) 404.0537, found 404.0536.

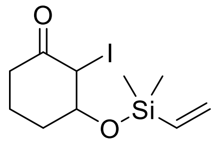

3-((Dimethylvinylsilyl)oxy)-2-iodocyclohexan-1-one 4v was synthesized from 3-hydroxy-2-iodocyclohexan-1-one **3v** (0.17 g, 0.69 mmol, 1.0 equiv.), chlorodimethylvinylsilane (0.12 mL, 0.83 mmol, 1.2 equiv.), 4-dimethylaminopyridine (23 mg, 0.19 mmol, 0.3 equiv.) and Et_3N (0.12 mL, 0.83 mmol, 1.2 equiv.) in dry CH_2Cl_2 (4.4 mL) according to Procedure C. The crude was purified by column chromatography (Et_2O/pentane 5%) to afford a separable 71:29 diastereomers mixture of 3-((dimethylvinylsilyl)oxy)-2-iodocyclohexan-1-one **4v** (0.14 g, 0.44 mmol, 64%) as a colorless oil.

R*f* 0.39 (*major*), 0.21 (*minor*) (Et_2O/pentane 5%); **IR** (liquid, $ν_{max}/cm^{-1}$) 2955, 1716, 1254, 1114, 1065, 1007, 880, 851, 791; **HRMS** (APCI) calc. for $C_{10}H_{18}IO_2Si$ ($[M+H]^+$) 325.0115, found 325.0114

major: **^{1}H NMR** (400 MHz, $CDCl_3$) δ 6.20–6.07 (1H, m, Si(C*H*CH$_2$)), 6.04 (1H, d, *J* = 9.7 Hz, Si(CHC*H*$_2$)), 5.79 (1H, dd, *J* = 18.9, 5.2 Hz, Si(CHC*H*$_2$)), 4.32–4.22 (2H, m, C*H*I, C*H*O), 3.14 (1H, ddd, *J* = 14.6, 12.2, 5.9 Hz, C*H*$_2$O), 2.52–2.38 (1H, m, C*H*$_2$CHO), 2.29 (1H, dt, *J* = 14.4, 4.5 Hz, C*H*$_2$O), 2.22–2.06 (1H, m, C*H*$_2$), 1.84–1.69 (2H, m, C*H*$_2$CHO, C*H*$_2$), 0.20 (3H, s, Si(C*H*$_3$)$_2$), 0.19 (3H, s, Si(C*H*$_3$)$_2$); **^{13}C{^{1}H} NMR** ($CDCl_3$, 101 MHz) δ 205.0, 136.9, 134.2, 75.8, 35.7, 32.4, 27.7, 20.3, -1.6 (2C).

minor: **^{1}H NMR** (400 MHz, $CDCl_3$) δ 6.13 (1H, dd, *J* = 19.8, 14.7 Hz, Si(C*H*CH$_2$)), 6.03 (1H, dd, *J* = 15.0, 4.6 Hz, Si(CHC*H*$_2$)), 5.80 (1H, dd, *J* = 19.8, 4.3 Hz, Si(CHC*H*$_2$)), 4.73–4.63 (1H, m, C*H*I), 3.36–3.25 (1H, m, C*H*O), 3.11 (1H, ddd, *J* = 14.7, 11.0, 6.1 Hz, C*H*$_2$O), 2.28 (1H, dt, *J* = 14.7, 5.2 Hz, C*H*$_2$O), 2.03–1.68 (3H, m, 2 x C*H*$_2$CHO, C*H*$_2$), 1.66–

1.50 (1H, m, CH_2), 0.23 (3H, s, Si(CH_3)$_2$), 0.22 (3H, s, Si(CH_3)$_2$); **^{13}C{^{1}H} NMR** (CDCl$_3$, 101 MHz) δ 203.6, 137.1, 134.2, 72.5, 44.5, 35.8, 31.8, 20.5, -1.4, -1.4.

13. References

1. Gualandi, A.; Rodeghiero, G.; Della Rocca, E.; Bertoni, F.; Marchini, M.; Perciaccante, R.; Jansen, T. P.; Ceroni, P.; Cozzi, P. G., Application of coumarin dyes for organic photoredox catalysis. *Chem. Comm.* **2018,** *54* (72), 10044-10047.
2. Filippini, D.; Silvi, M., Visible light-driven conjunctive olefination. *Nat. Chem.* **2022**, *14*, 66-70.
3. Haugland, M. M.; El-Sagheer, A. H.; Porter, R. J.; Peña, J.; Brown, T.; Anderson, E. A.; Lovett, J. E., 2′-Alkynylnucleotides: A Sequence- and Spin Label-Flexible Strategy for EPR Spectroscopy in DNA. *J. Am. Chem. Soc.* **2016,** *138* (29), 9069-9072.
4. Yadav, J. S.; Reddy, B. V. S.; Narasimhulu, G.; Purnima, K. V., $FeCl_3$-catalyzed functionalization of monoterpenes via hydroalkylation of unactivated alkenes. *Tetrahedron Lett.* **2009,** *50* (42), 5783-5785.
5. Wata, C.; Hashimoto, T., Organoiodine-Catalyzed Enantioselective Intermolecular Oxyamination of Alkenes. *J. Am. Chem. Soc.* **2021,** *143* (4), 1745-1751.
6. Pospisil, J.; Marko, I. E., Efficient and stereoselective synthesis of allylic ethers and alcohols. *Org. Lett.* **2006,** *8* (26), 5983-5986.
7. Kelly, C. B.; Ovian, J. M.; Cywar, R. M.; Gosselin, T. R.; Wiles, R. J.; Leadbeater, N. E., Oxidative cleavage of allyl ethers by an oxoammonium salt. *Org. Biomol. Chem.* **2015,** *13* (14), 4255-4259.
8. Zhang, M. Z.; Wang, X.; Gong, M. Y.; Chen, L.; Shi, W. B.; He, S. H.; Jiang, Y.; Chen, T. Q., An efficient iodine pentoxide-triggered iodocarbocyclization for the synthesis of iodooxindoles in water. *Org. Biomol. Chem.* **2018,** *16* (28), 5197-5202.
9. Geng, X. L.; Wang, Z.; Li, X. Q.; Zhang, C., A simple method for epoxidation of olefins using sodium chlorite as an oxidant without a catalyst. *J. Org. Chem.* **2005,** *70* (23), 9610-9613.
10. Zhu, Y. X.; Colomer, I.; Thompson, A. L.; Donohoe, T. J., HFIP Solvent Enables Alcohols To Act as Alkylating Agents in Stereoselective Heterocyclization. *J. Am. Chem. Soc.* **2019,** *141* (16), 6489-6493.
11. Yao, H. R.; Richardson, D. E., Epoxidation of alkenes with bicarbonate-activated hydrogen peroxide. *J. Am. Chem. Soc.* **2000,** *122* (13), 3220-3221.
12. Ren, J.; Yu, P. C.; Zhang, M. J.; Zhao, Y. X.; Zhong, J.; Hu, K., Discovery of alpha-methylene-gamma-lactone-delta-epoxy derivatives with anti-cancer activity: synthesis, SAR study, and biological activity. *Med. Chem. Res.* **2022,** *31* (10), 1803-1817.
13. Das, B.; Venkateswarlu, K.; Damodar, K.; Suneel, K., Ammonium acetate catalyzed improved method for the regioselective conversion of olefins into halohydrins and haloethers at room temperature. *J. Mol. Catal. A: Chem.* **2007,** *269* (1-2), 17-21.
14. Yi, W.; Wang, P. F.; Lu, M.; Liu, Q. Q.; Bai, X.; Chen, K. D.; Zhang, J. W.; Liu, G. Q., Environmentally Friendly Protocol for the Oxidative Iodofunctionalization of Olefins in a Green Solvent. *ACS Sustain. Chem. Eng.* **2019,** *7* (19), 16777-16785.
15. Hu, Q. Y.; Rege, P. D.; Corey, E. J., Simple, catalytic enantioselective syntheses of estrone and desogestrel. *J. Am. Chem. Soc.* **2004,** *126* (19), 5984-5986.
16. Narender, M.; Reddy, M. S.; Nageswar, Y. D.; Rao, K. R., Aqueous phase synthesis of vic-halohydrins from olefins and N-halosuccinimides in the presence of β-cyclodextrin. *J. Mol. Catal. A: Chem.* **2006,** *258* (1-2), 10-14.
17. Shimizu, A.; Hayashi, R.; Ashikari, Y.; Nokami, T.; Yoshida, J., Switching the reaction pathways of electrochemically generated β-haloalkoxysulfonium ions - synthesis of halohydrins and epoxides. *Beilstein J. Org. Chem.* **2015,** *11*, 242-248.
18. Dhokale, R. A.; Seidl, F. J.; Shinde, A. H.; Mague, J. T.; Sathyamoorthi, S., Tethered Silanoxyiodination of Alkenes. *J. Org. Chem.* **2021,** *86* (13), 9233-9243.
19. Sanseverino, A. M.; de Mattos, M. C. S., Iodohydrins: An easy route to epoxides from alkenes. *Synth. Commun.* **1998,** *28* (3), 559-572.
20. Braun, H. A.; Meusinger, R.; Schmidt, B., 2-Iodoethanols from aldehydes, diiodomethane and isopropylmagnesium chloride. *Tetrahedron Lett.* **2005,** *46* (15), 2551-2554.
21. Sharma, S. M. L.; Singh, J., Envirocat (K10-MX)-Catalyzed Regioselective Transformation of Alkenes into Iodohydrins and β-Iodo Ethers and Further Conversion of Iodohydrins to Epoxides Using Al_2O_3-Na_2CO_3 Under MWI. *Synth. Commun.* **2012,** *42* (9), 1306-1324.
22. Luan, S. N.; Castanheiro, T.; Poisson, T., Electrochemical Synthesis of Iodohydrins. *Adv. Synth. Catal.* **2022,** *364* (16), 2741-2747.

23. Urankar, D.; Rutar, I.; Modec, B.; Dolenc, D., Synthesis of bromo- and iodohydrins from deactivated alkenes by use of N-bromo- and N-iodosaccharin. *Eur. J. Org. Chem.* **2005,** *2005* (11), 2349-2353.
24. Someya, H.; Ohmiya, H.; Yorimitsu, H.; Oshima, K., Cobalt-catalyzed sequential cyclization/cross-coupling reactions of 6-halo-1-hexene derivatives with Grignard reagents and their application to the synthesis of 1,3-diols. *Tetrahedron* **2007,** *63* (35), 8609-8618.

7-(Diethylamino)-3-(thiophen-2-yl)-2H-chromen-2-one

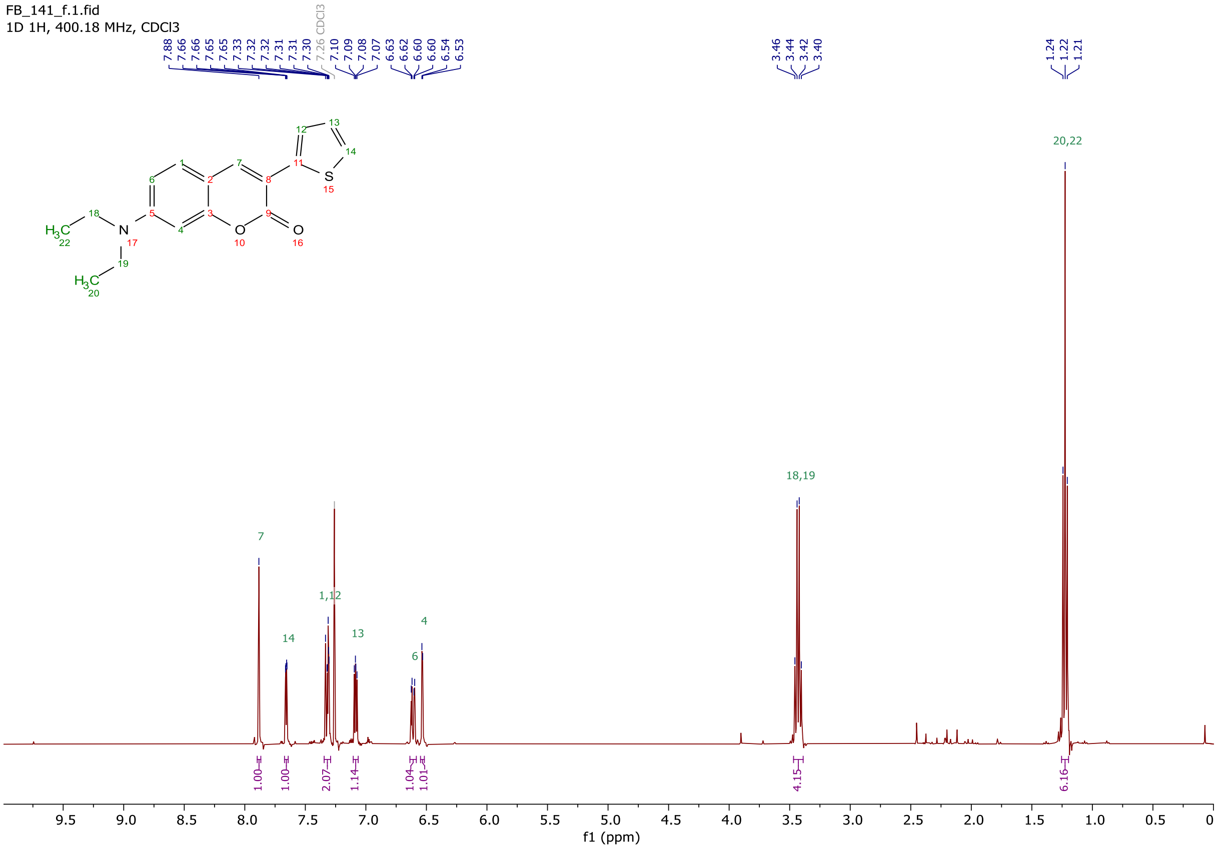

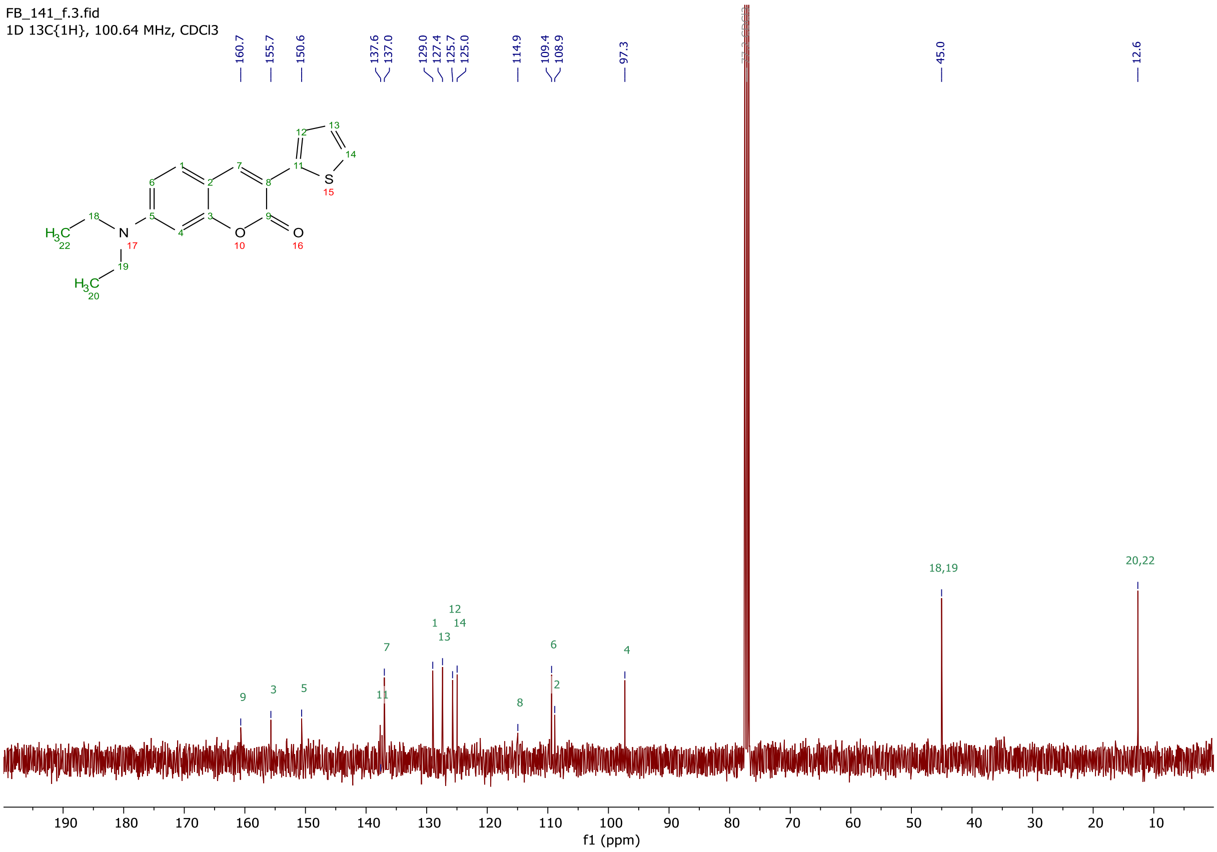

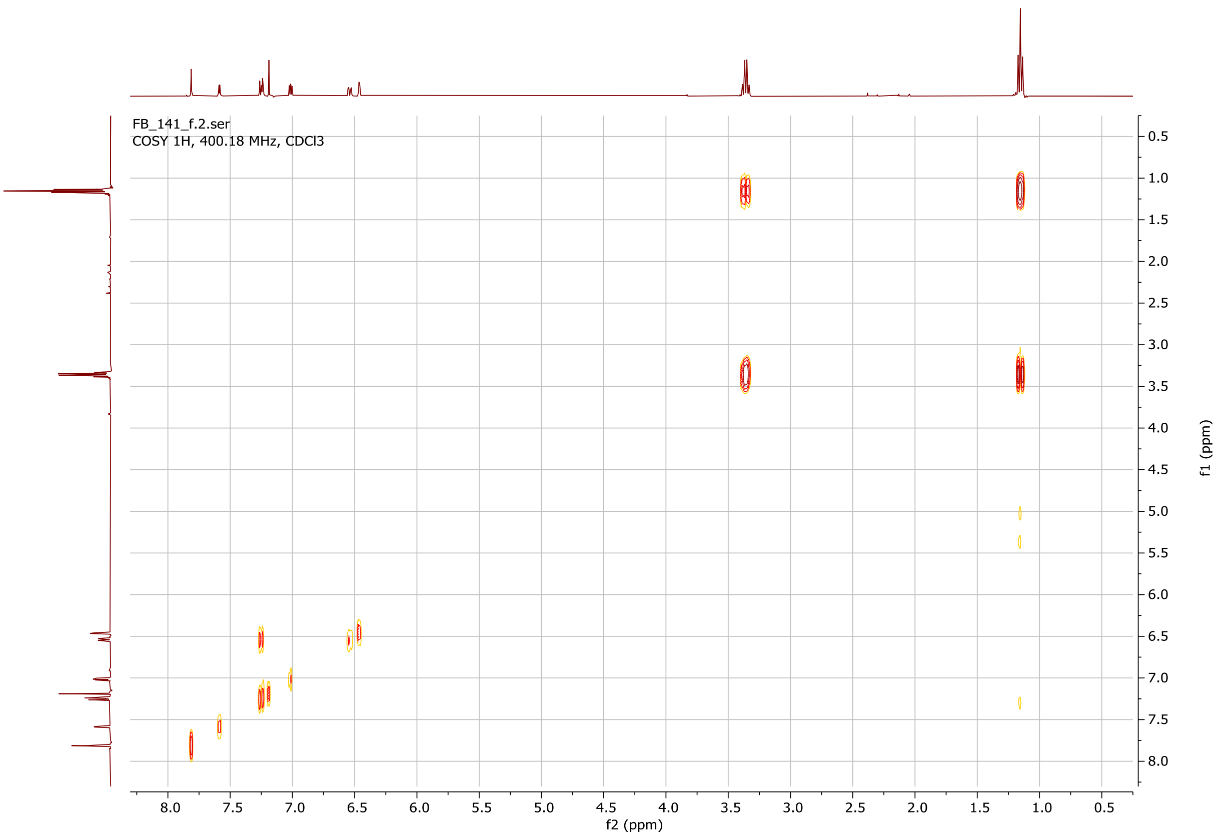
FB_141_f.2.ser
COSY 1H, 400.18 MHz, CDCl3
f2 (ppm)
f1 (ppm)

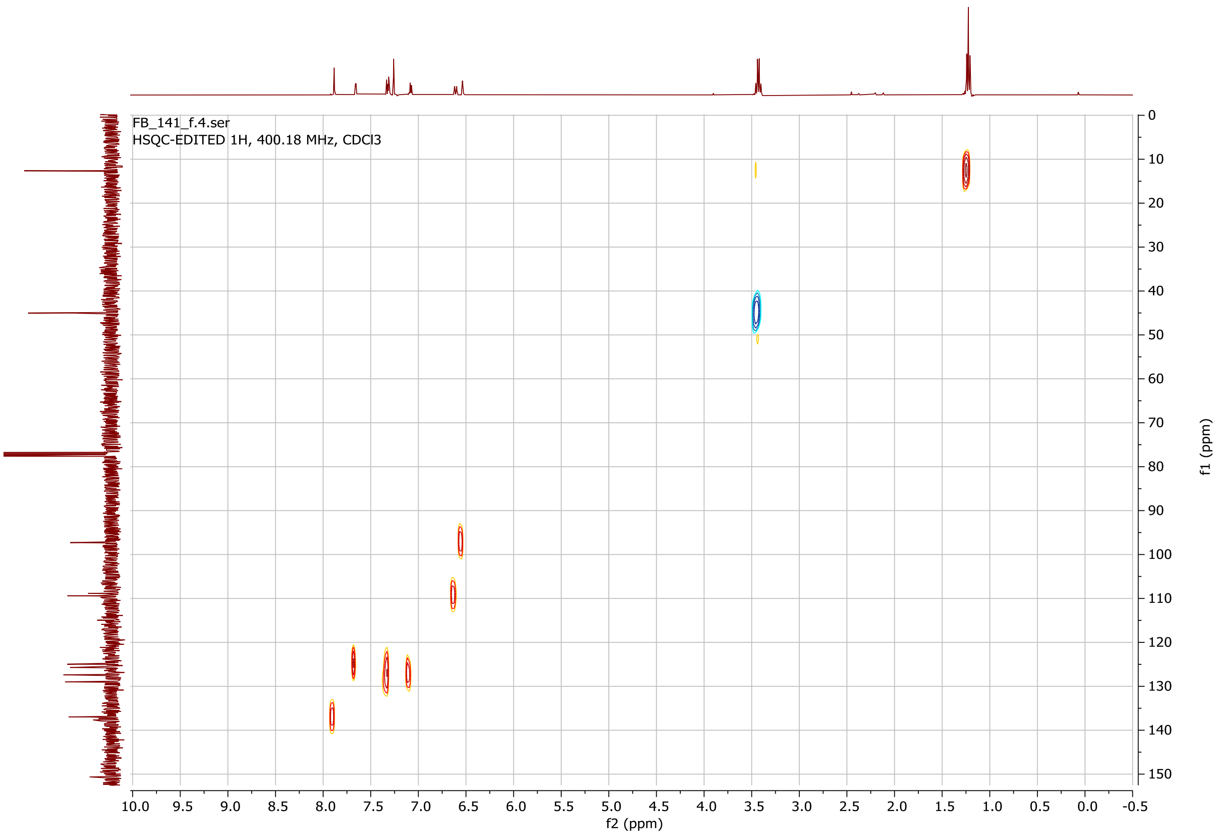
FB_141_f.4.ser
HSQC-EDITED 1H, 400.18 MHz, CDCl3
f2 (ppm)
f1 (ppm)

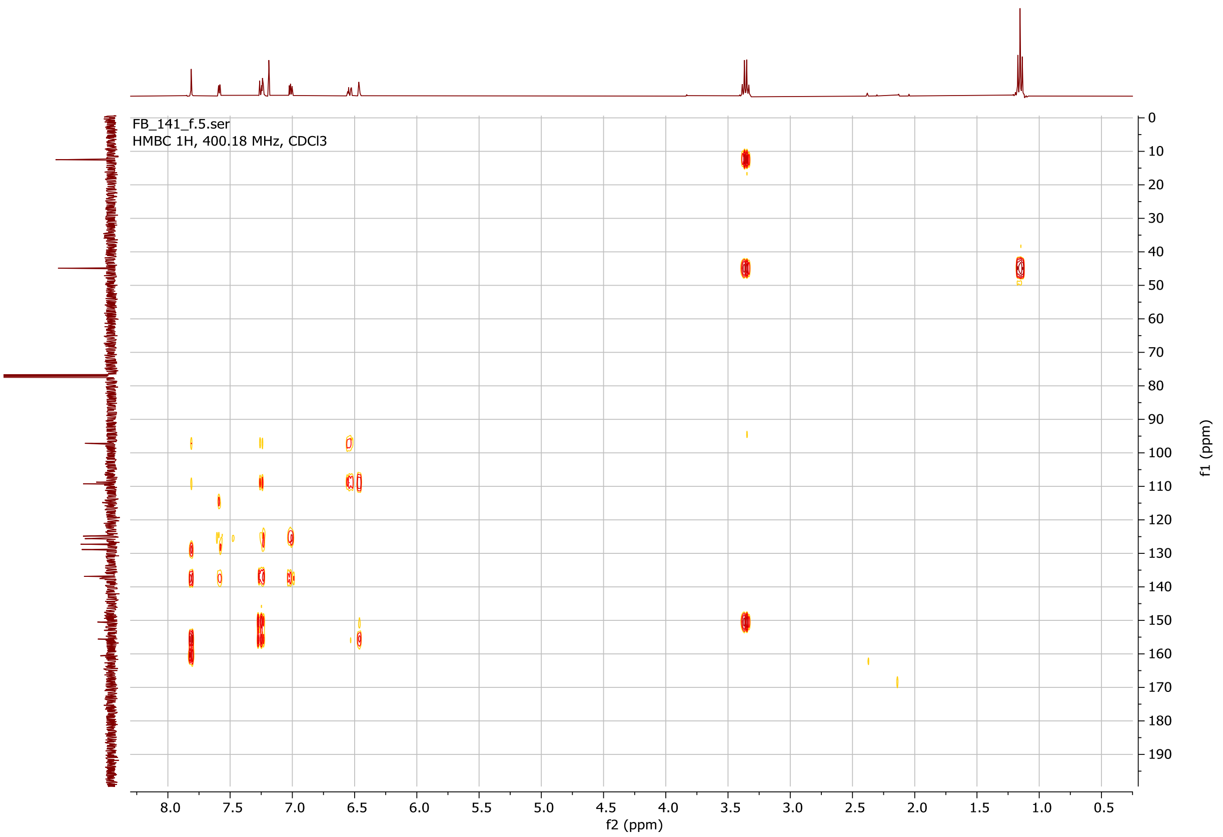

FB_141_f.5.ser
HMBC 1H, 400.18 MHz, CDCl3
f2 (ppm)
f1 (ppm)

4CzIPN

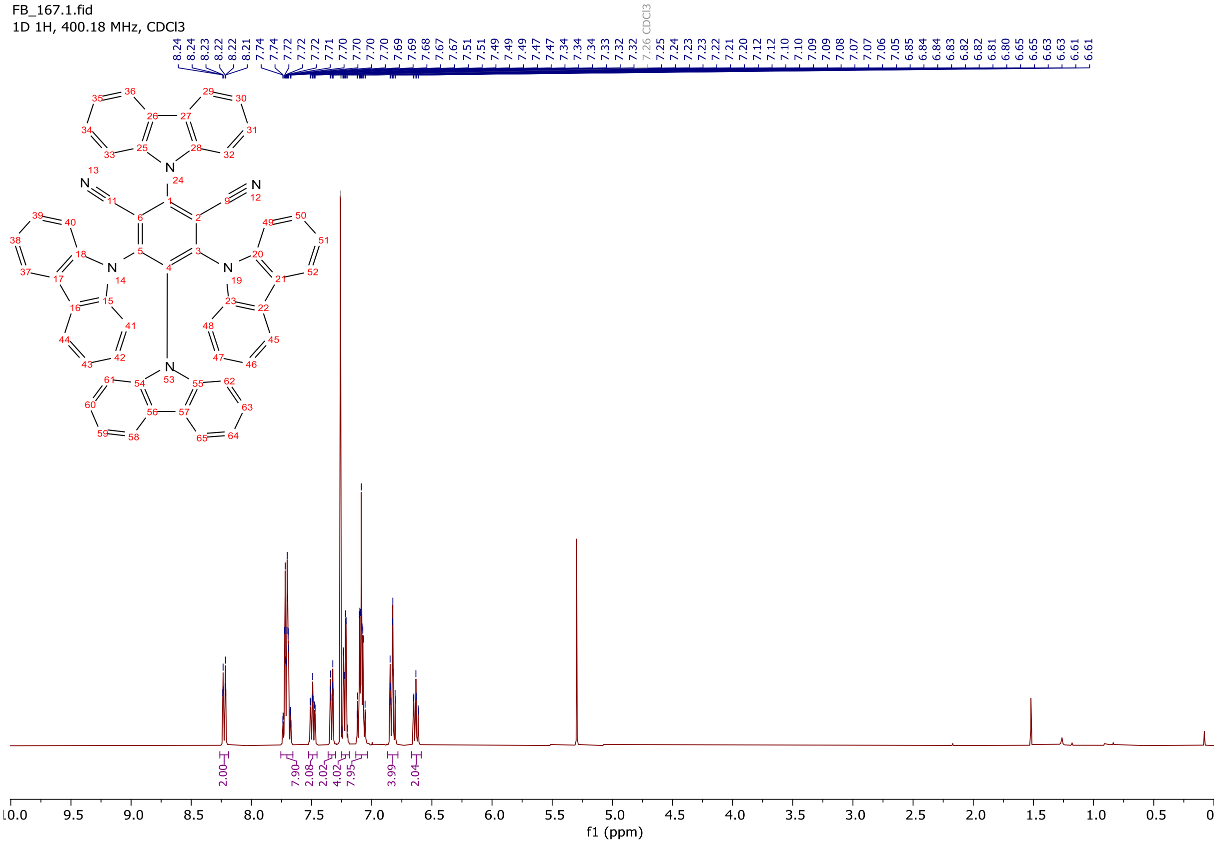

FB_167.1.fid
1D 1H, 400.18 MHz, CDCl3
7.26 CDCl3
2.00
7.90
2.08
2.02
4.02
7.95
3.99
2.04
f1 (ppm)

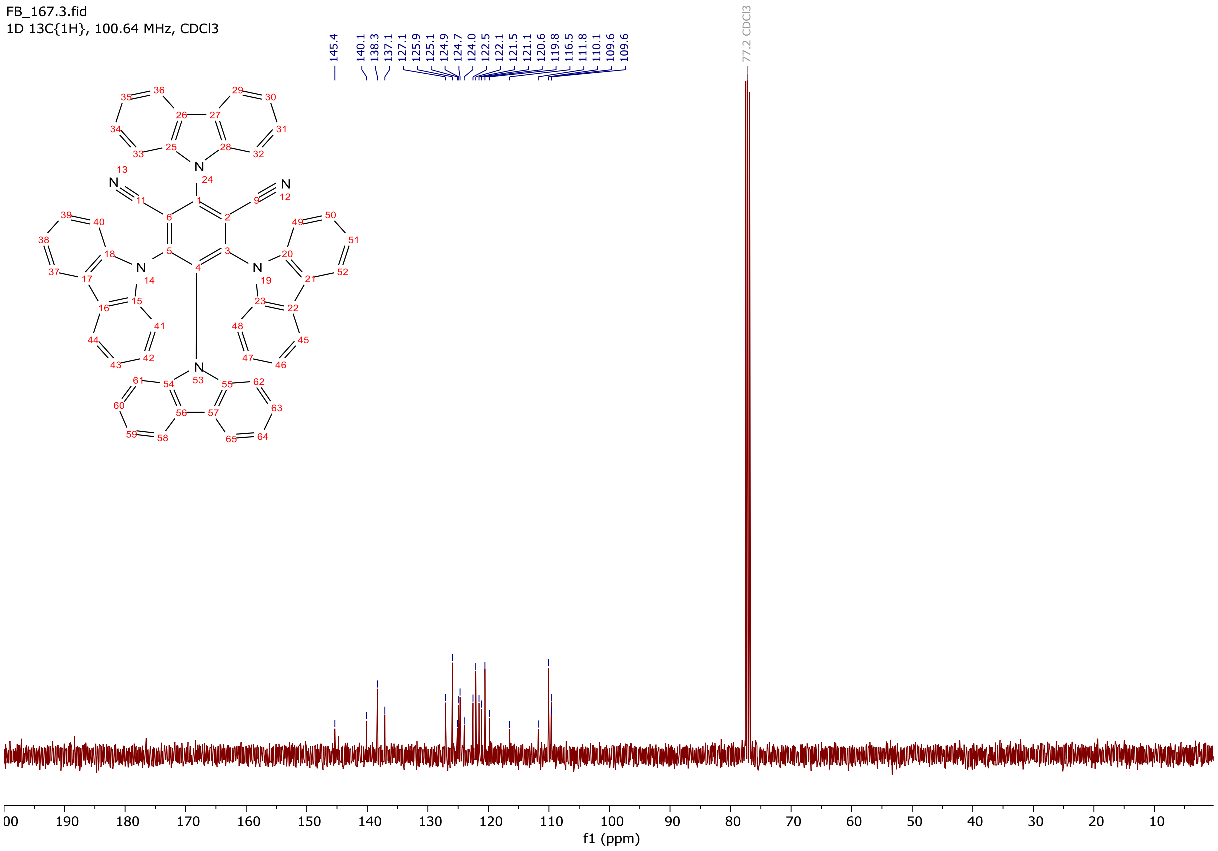

Chloro(diethylamino)dimethylsilane **s1**

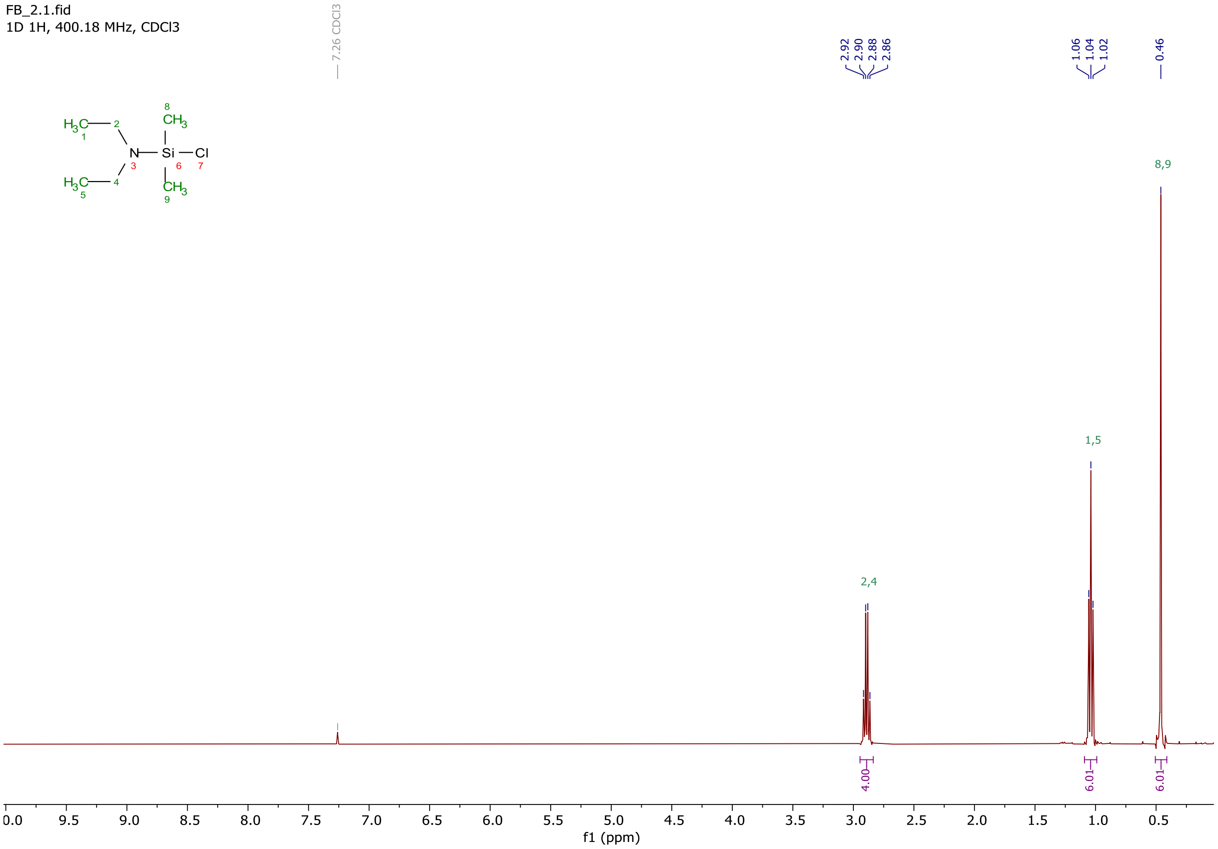

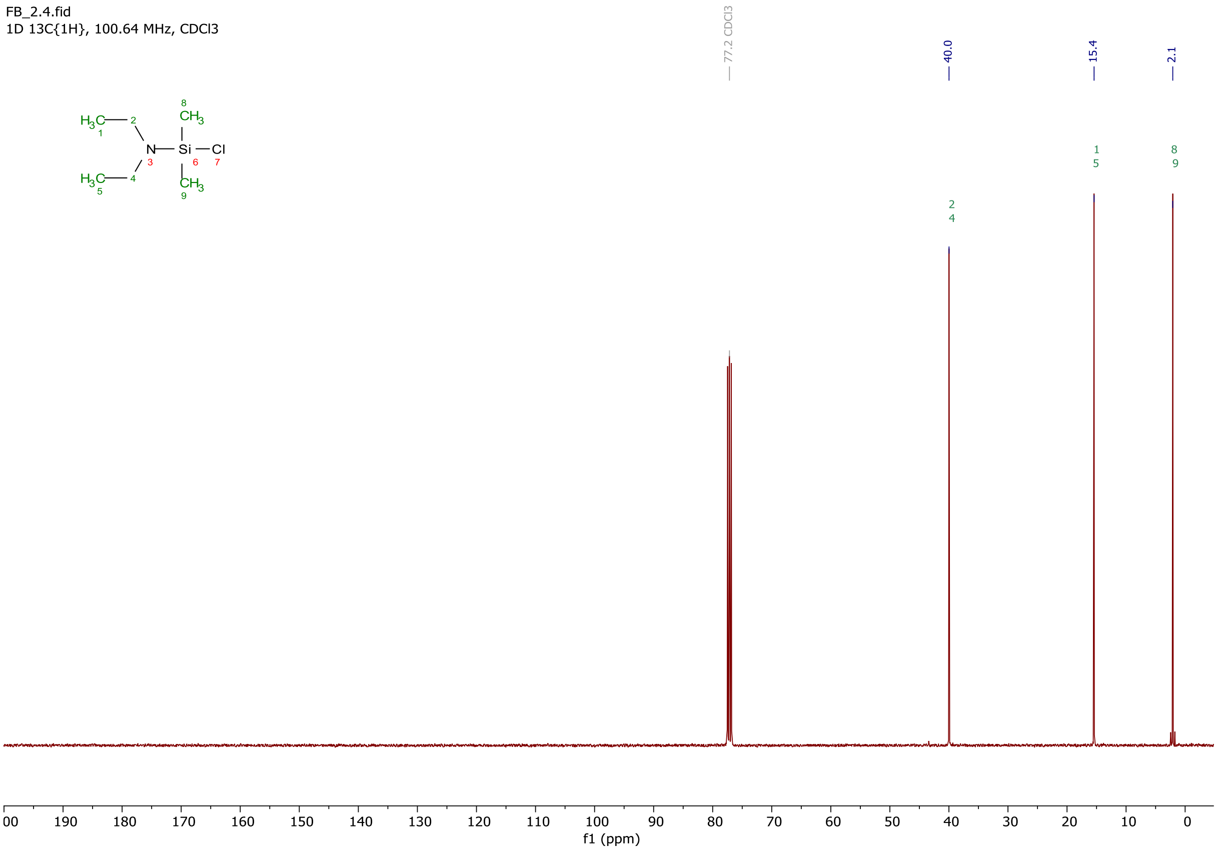

FB_2.4.fid
1D 13C{1H}, 100.64 MHz, CDCl3
77.2 CDCl3
40.0
15.4
2.1
f1 (ppm)

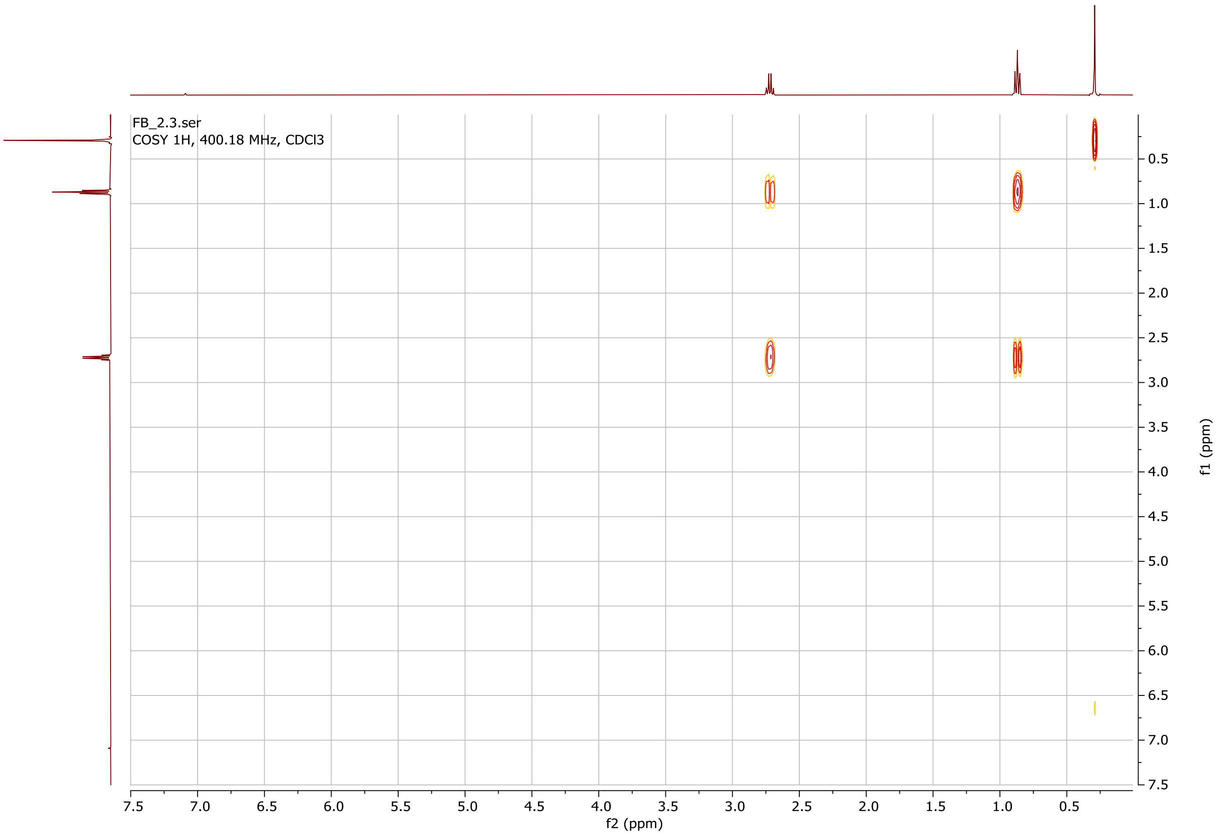

FB_2.3.ser
COSY 1H, 400.18 MHz, CDCl3
f2 (ppm)
f1 (ppm)

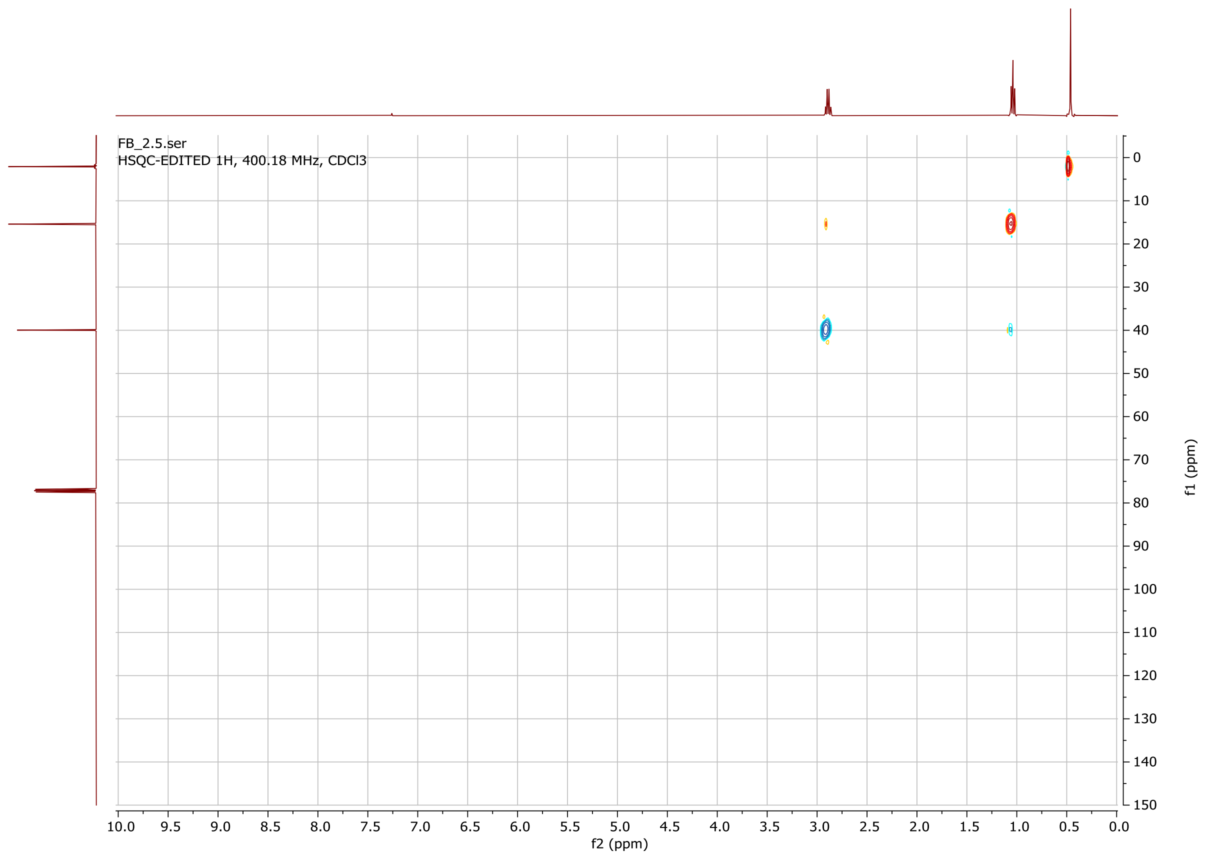

Trimethylsilylethynyl(diethylamino)dimethylsilane **s2**

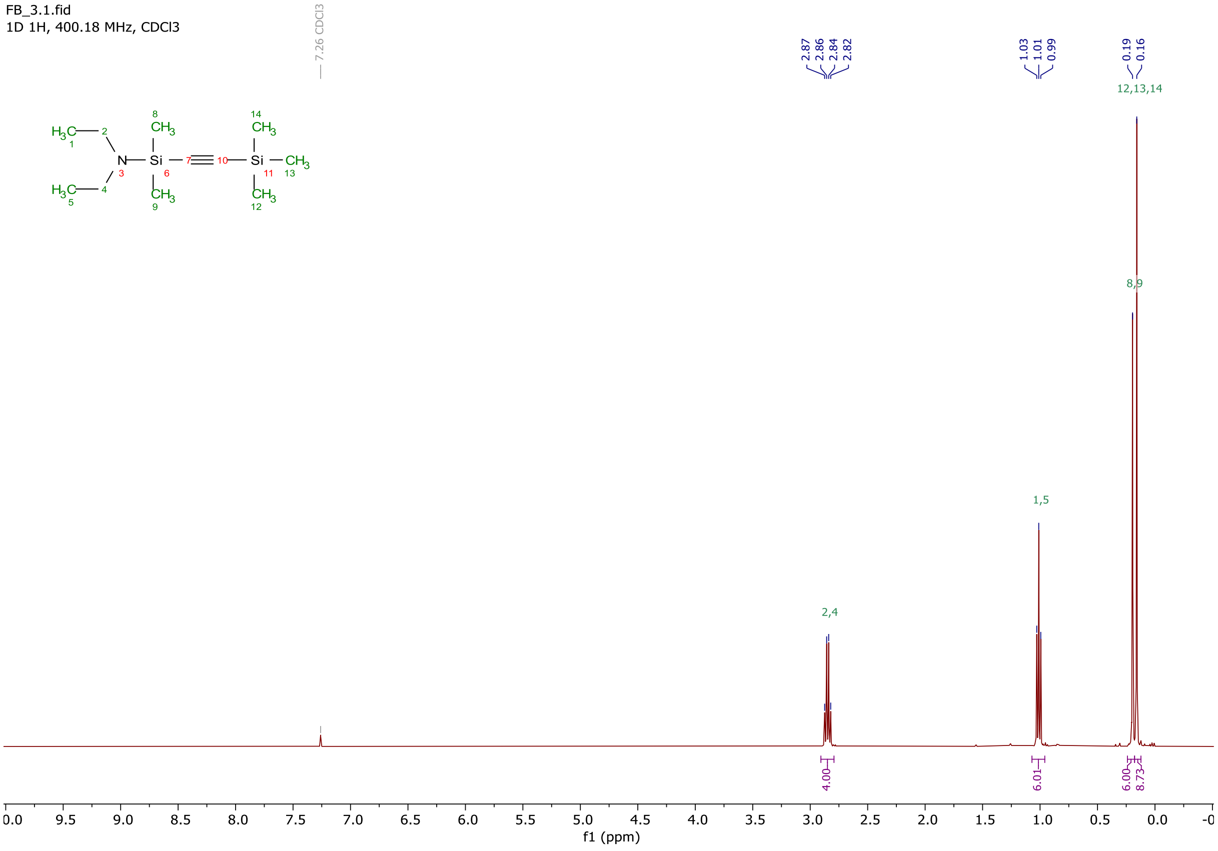

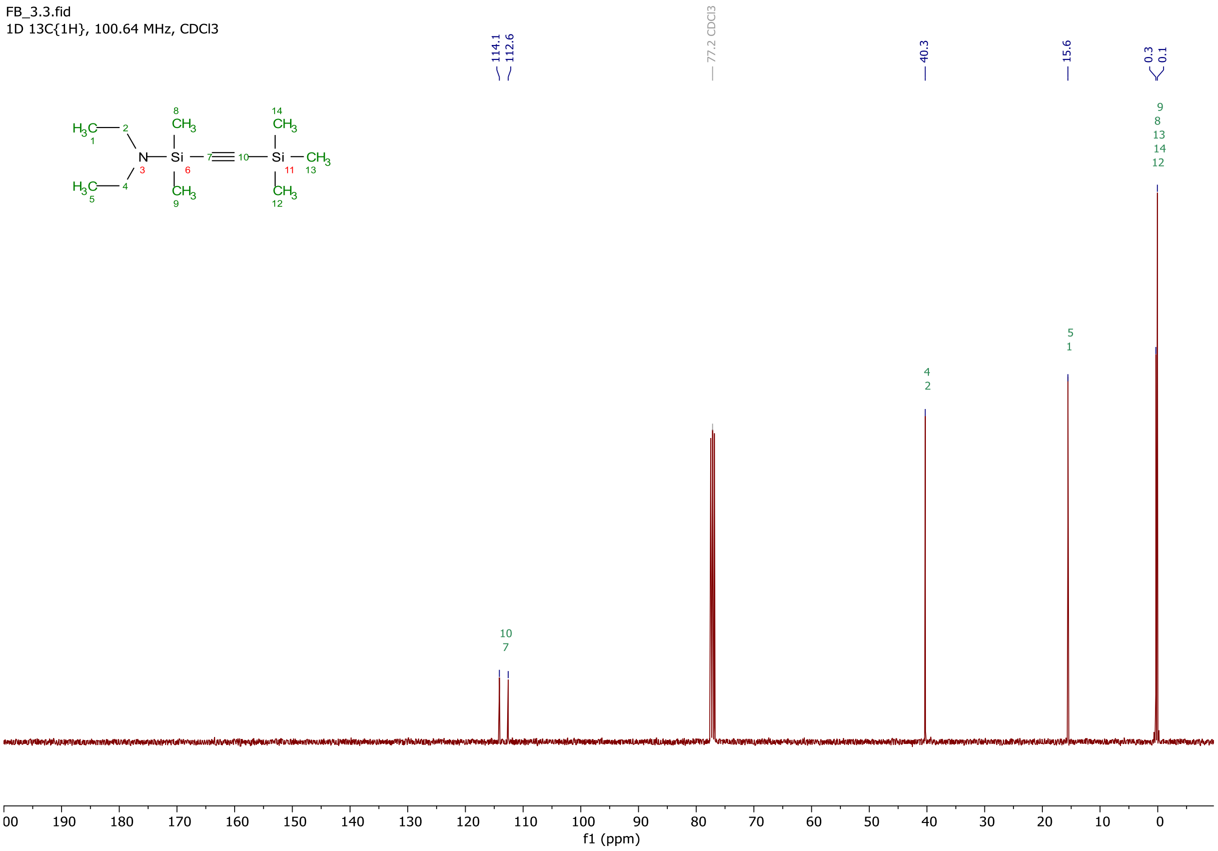
FB_3.3.fid
1D 13C{1H}, 100.64 MHz, CDCl3
114.1
112.6
77.2 CDCl3
40.3
15.6
0.3
0.1
f1 (ppm)

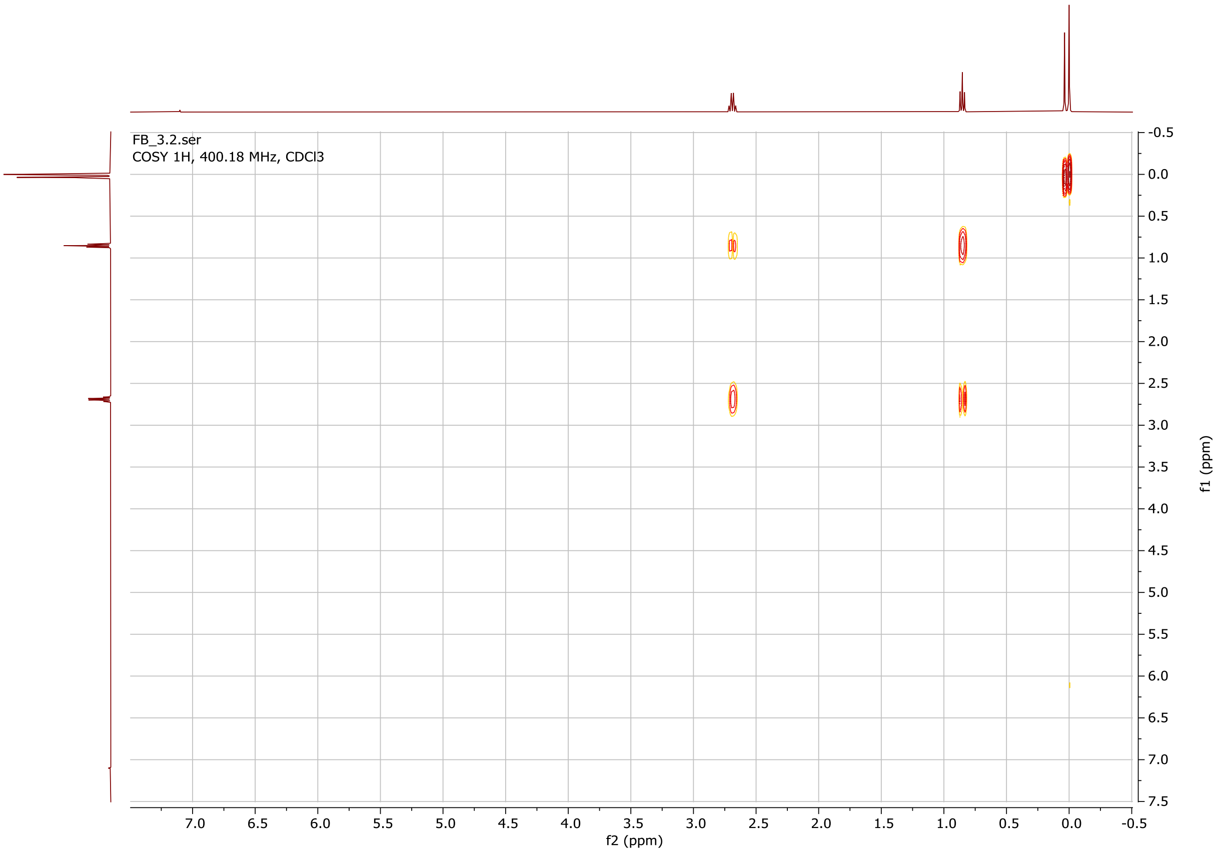
FB_3.2.ser
COSY 1H, 400.18 MHz, CDCl3
f2 (ppm)
f1 (ppm)

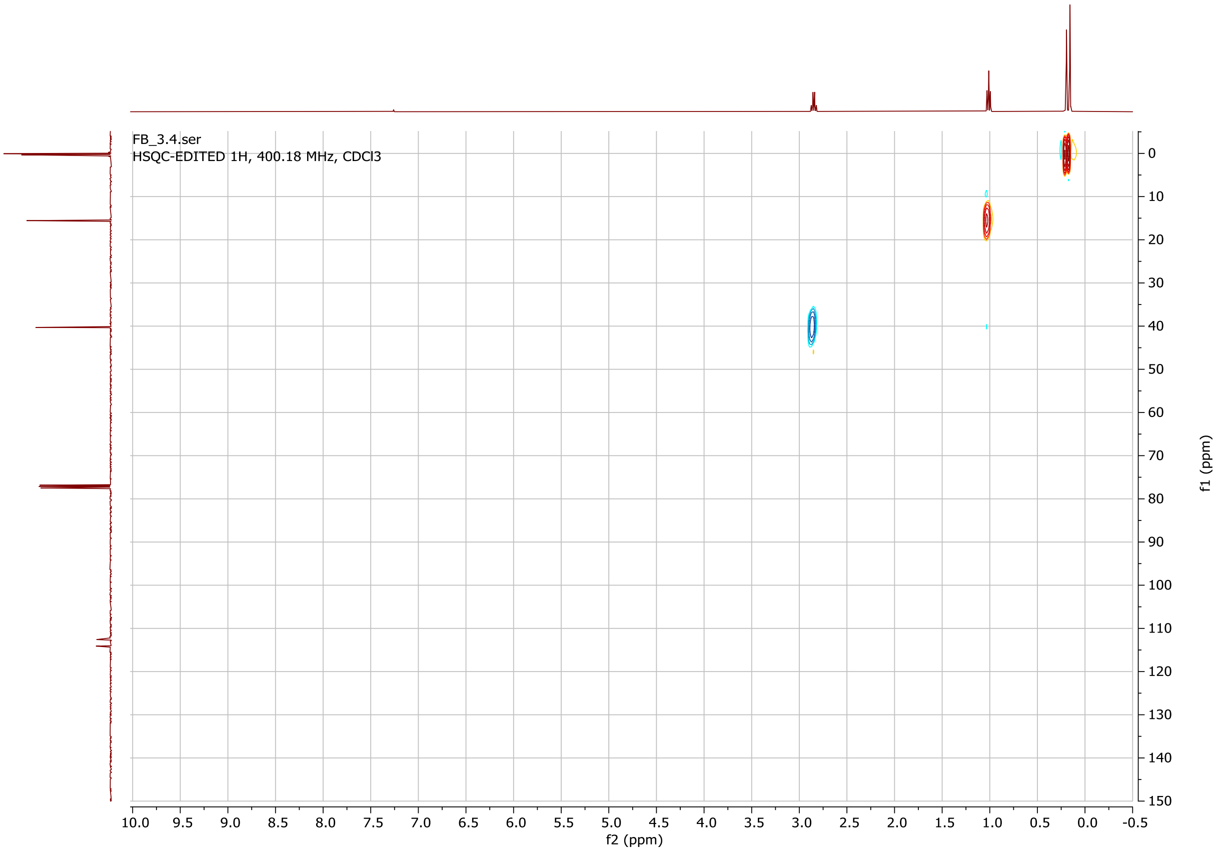

4-(1-(Benzyloxy)-1-methylethyl)-1-methylcyclohexene **1e**

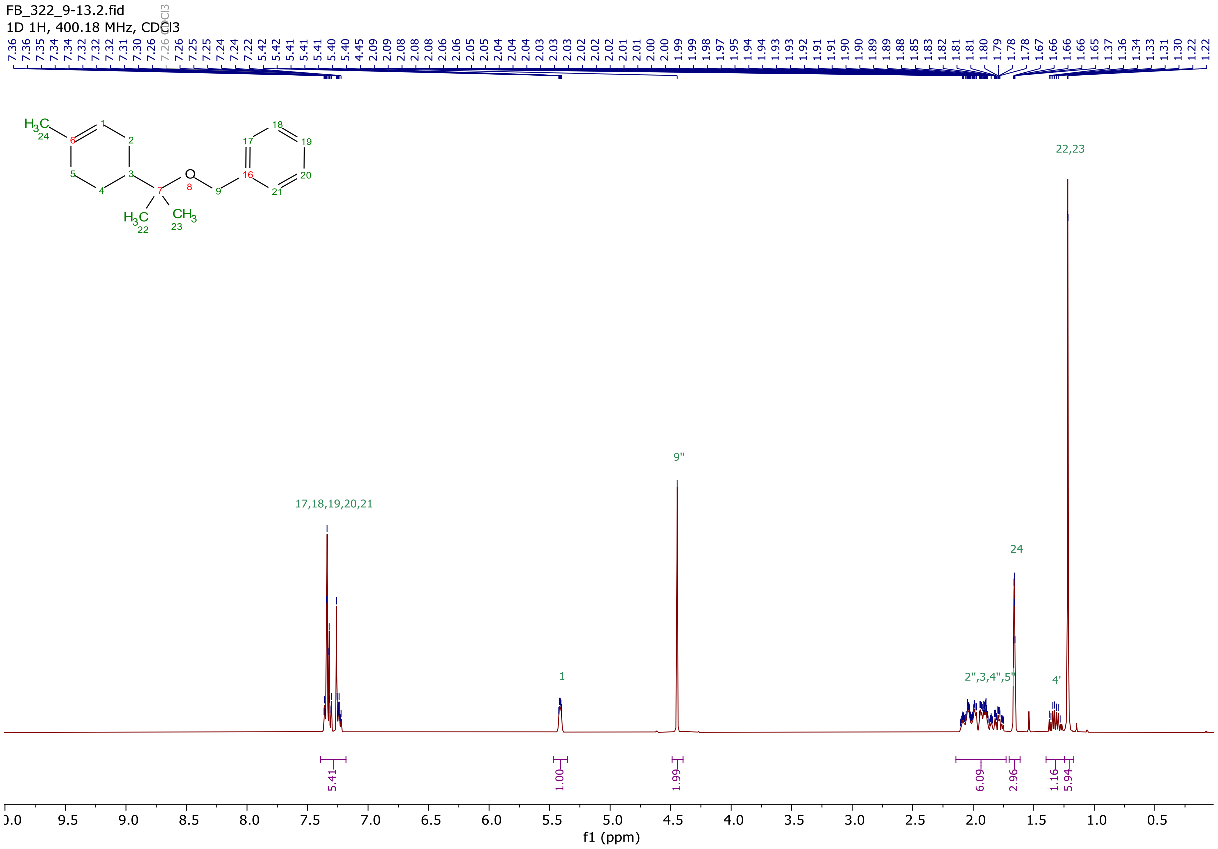

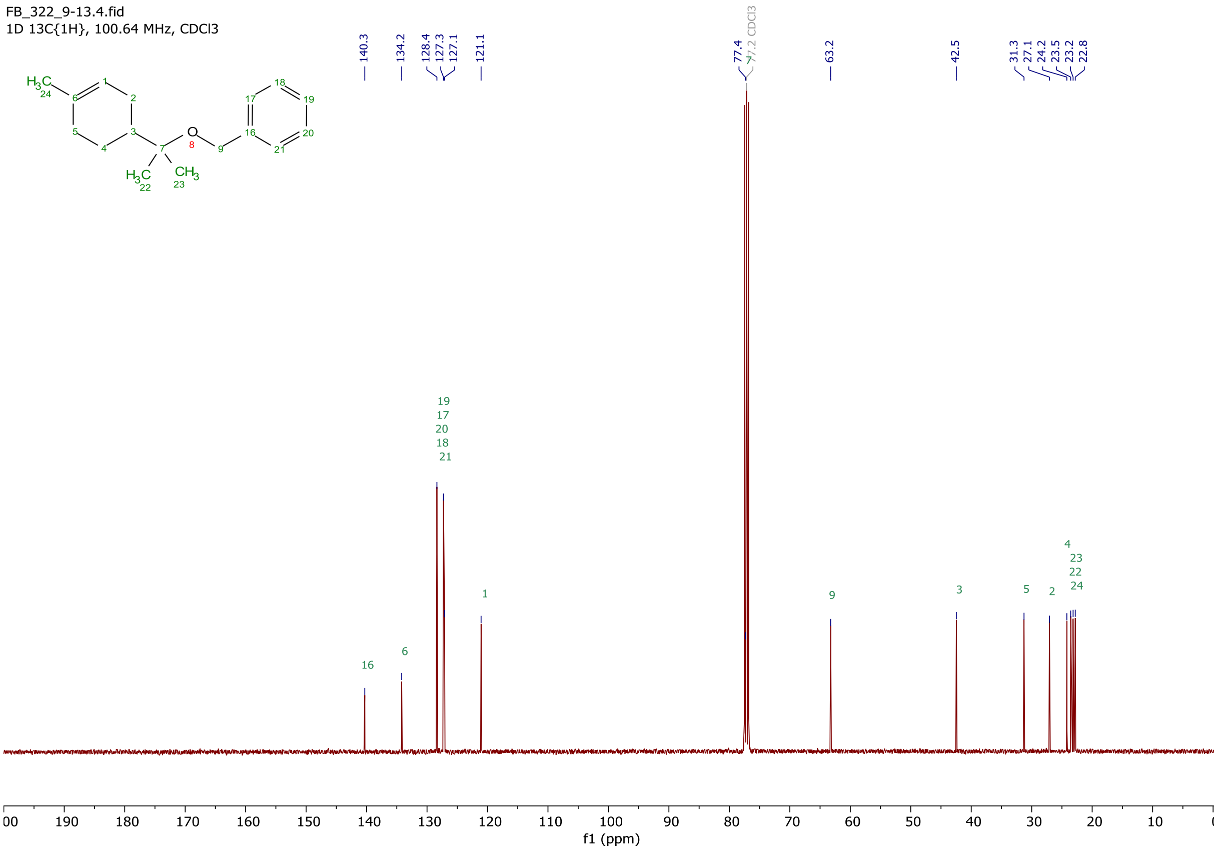

FB_322_9-13.4.fid
1D 13C{1H}, 100.64 MHz, CDCl3
140.3
134.2
128.4
127.3
127.1
121.1
77.4
77.2 CDCl3
63.2
42.5
31.3
27.1
24.2
23.5
23.2
22.8
f1 (ppm)

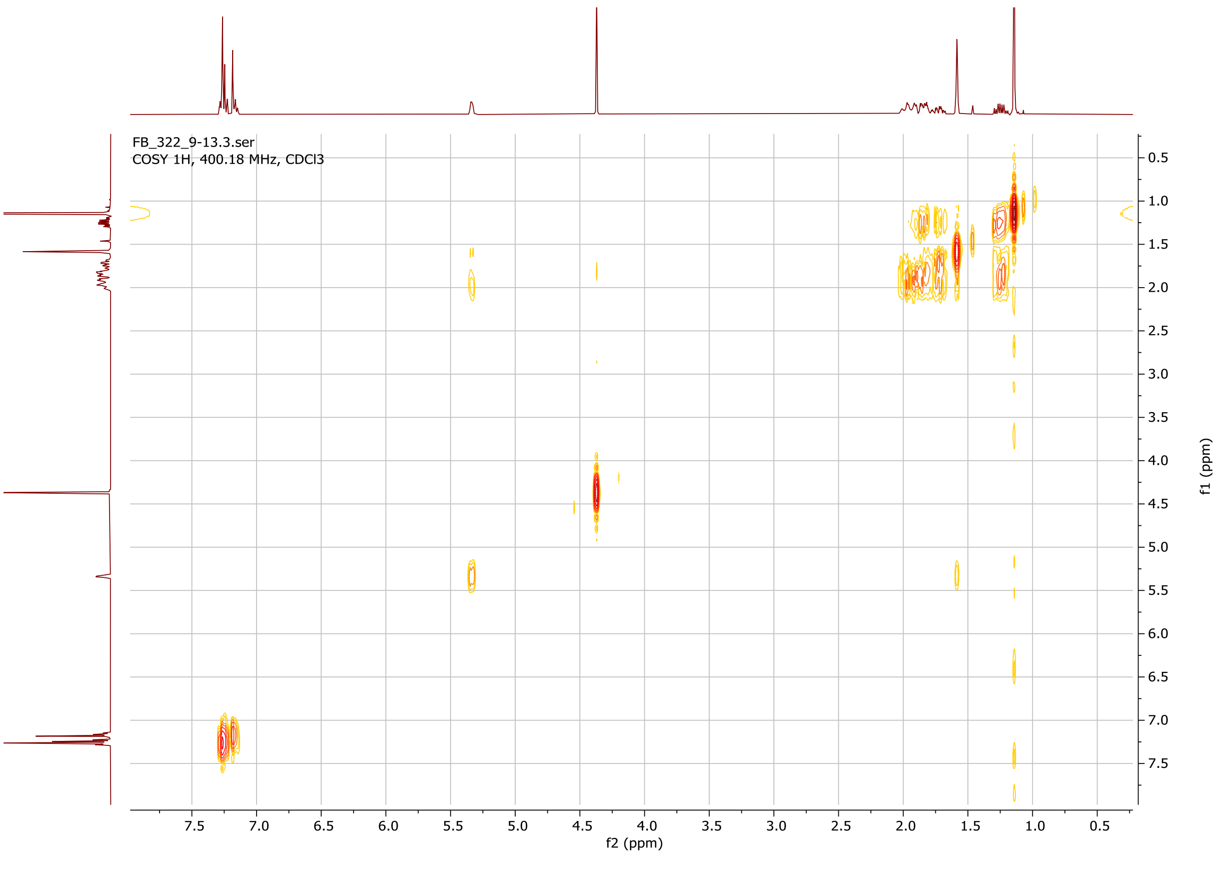

FB_322_9-13.3.ser
COSY 1H, 400.18 MHz, CDCl3
f2 (ppm)
f1 (ppm)

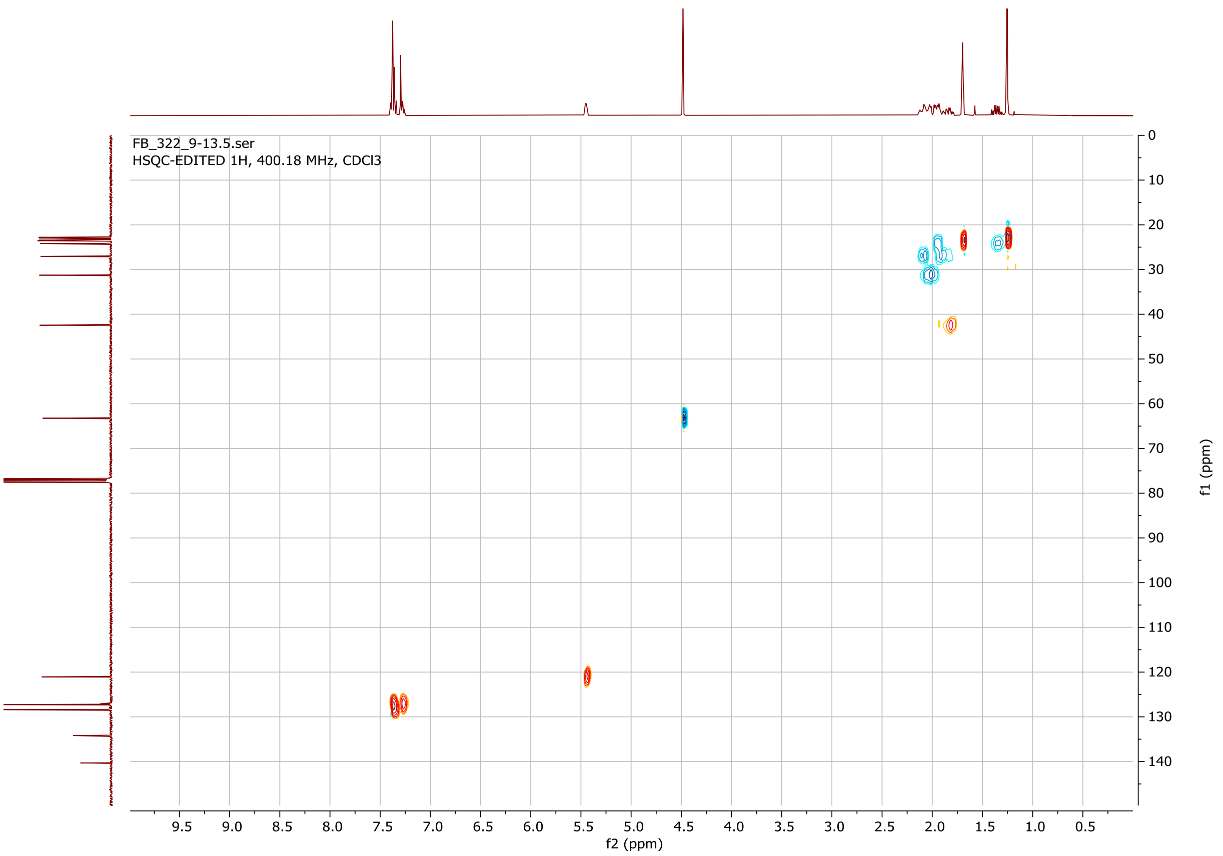
FB_322_9-13.5.ser
HSQC-EDITED 1H, 400.18 MHz, CDCl3
f2 (ppm)
f1 (ppm)

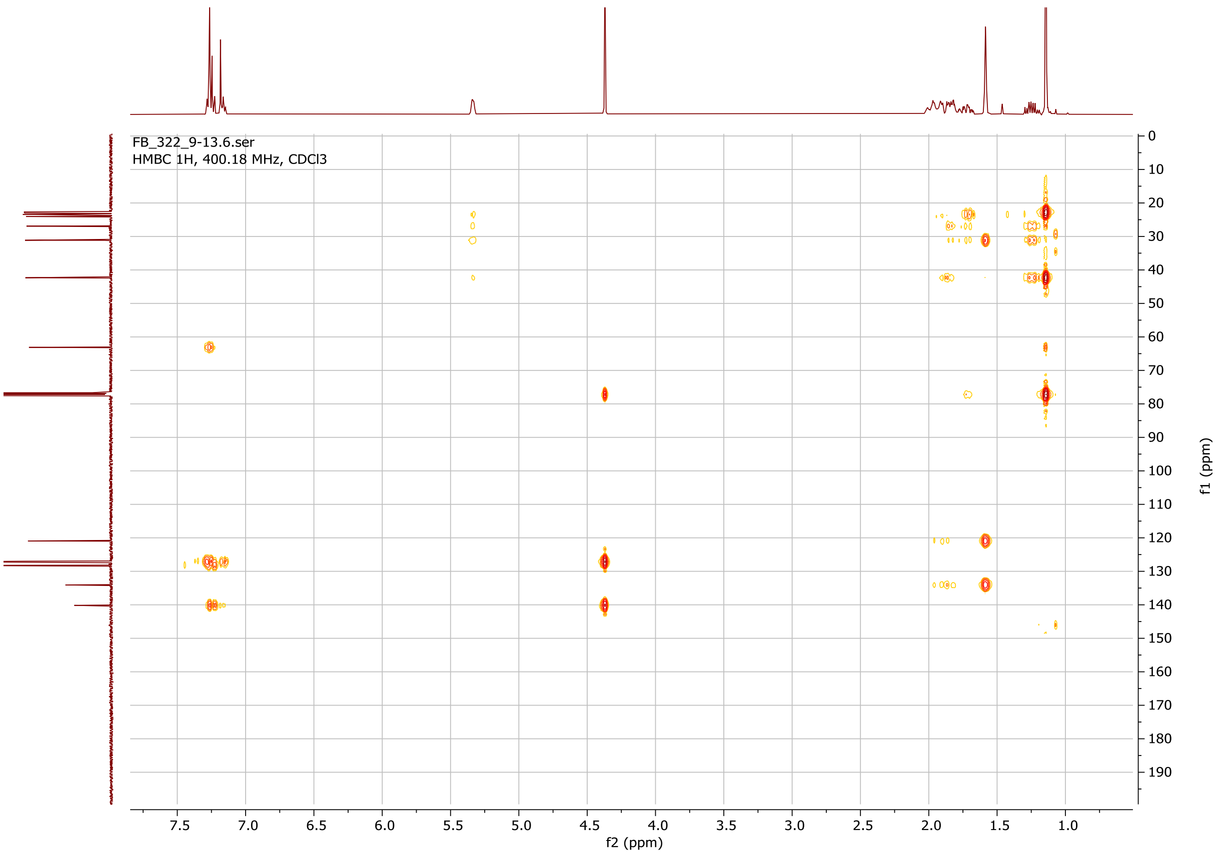
FB_322_9-13.6.ser
HMBC 1H, 400.18 MHz, CDCl3
f2 (ppm)
f1 (ppm)

tert-Butyl(hex-2-en-1-yloxy)dimethylsilane **1i**

FB_266.6.fid
1D 1H, 400.18 MHz, CDCl3

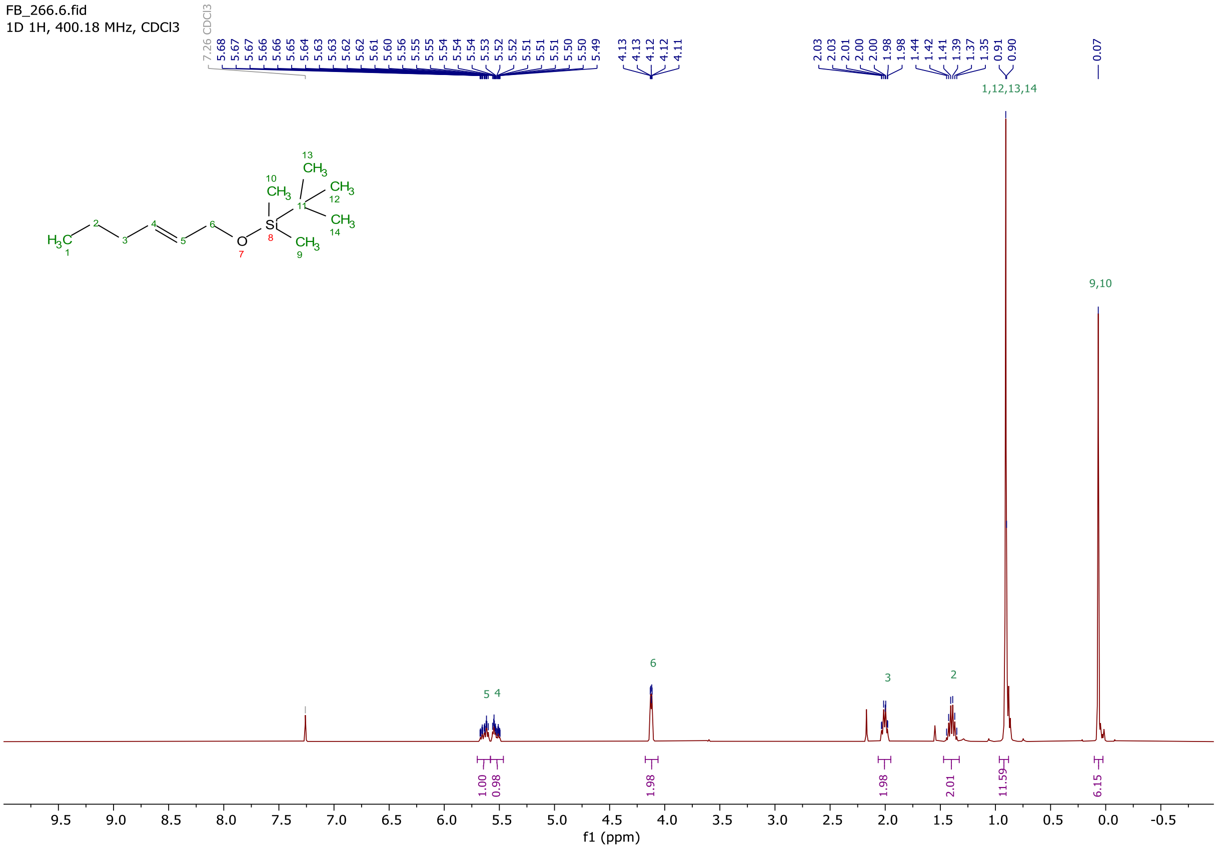

FB_266.8.fid
1D 13C{1H}, 100.64 MHz, CDCl3

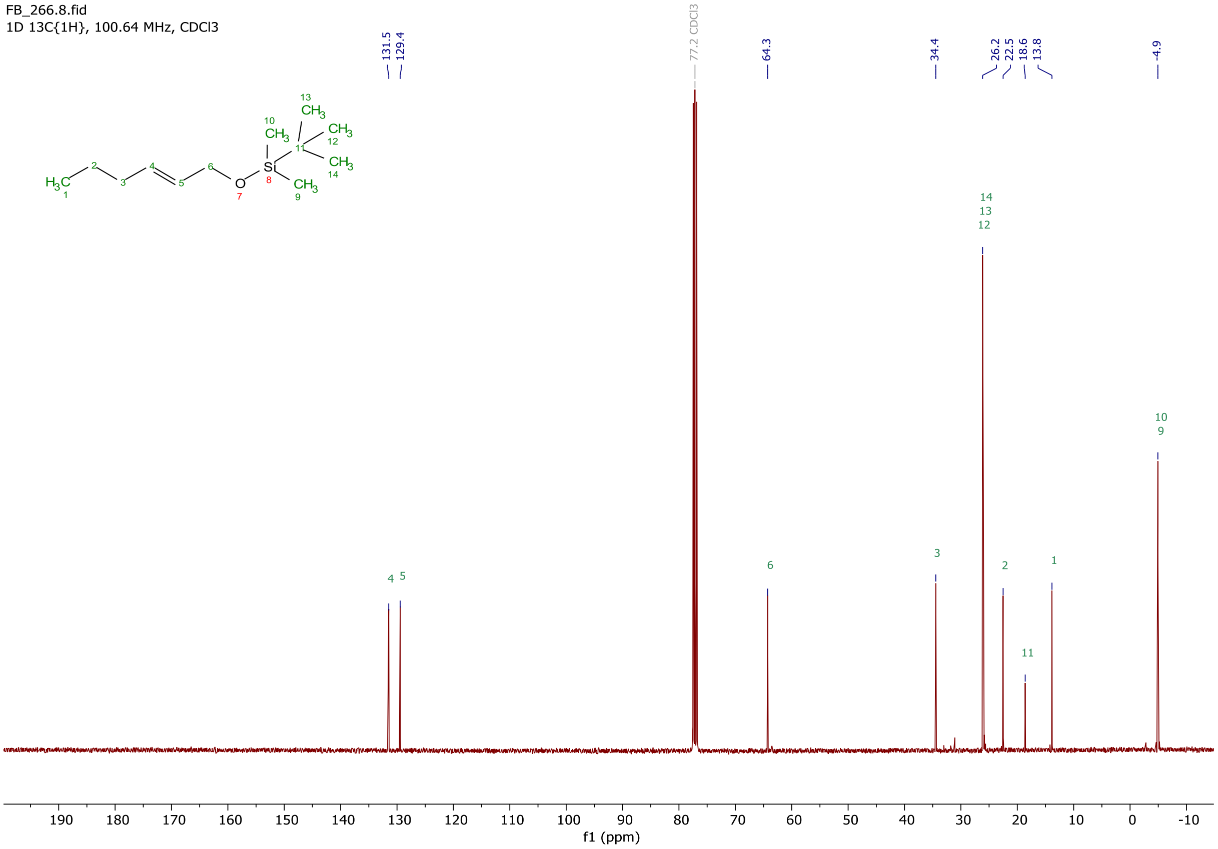

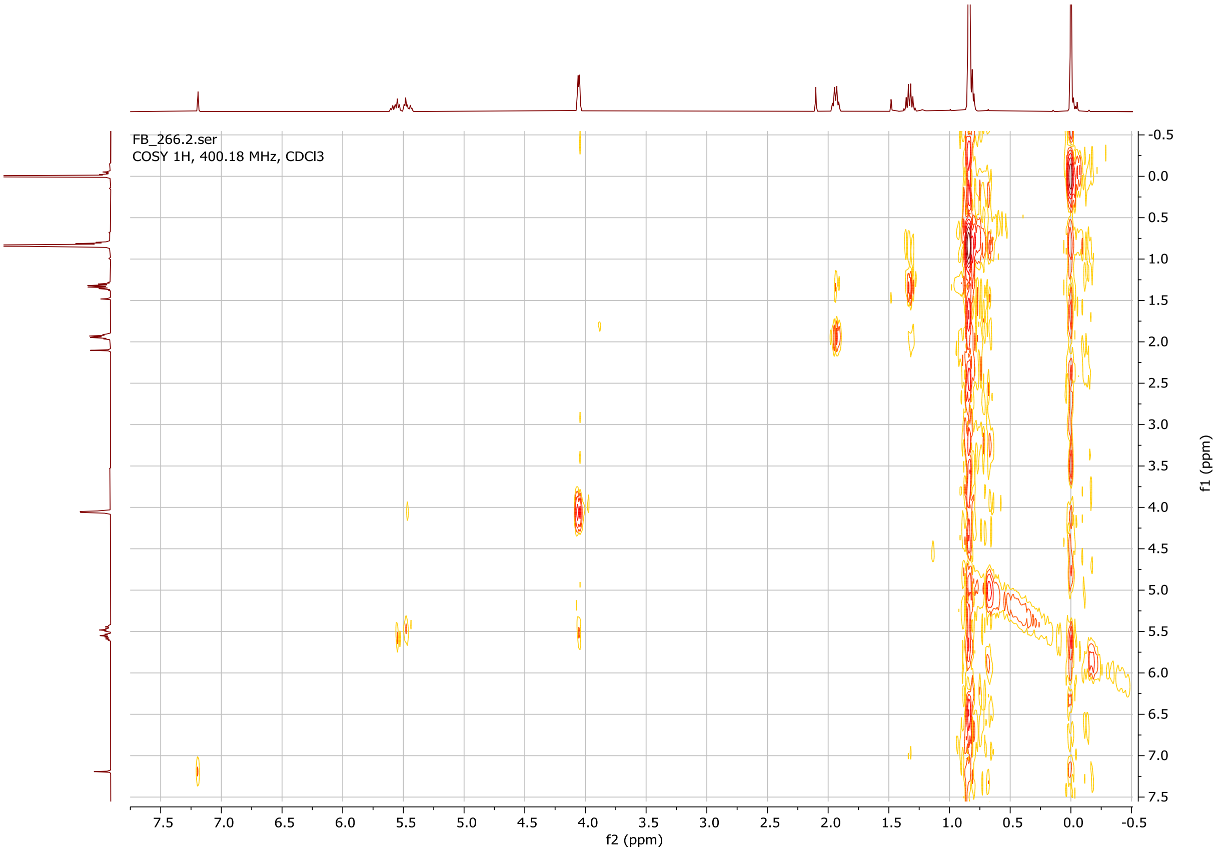
FB_266.2.ser
COSY 1H, 400.18 MHz, CDCl3
f2 (ppm)
f1 (ppm)

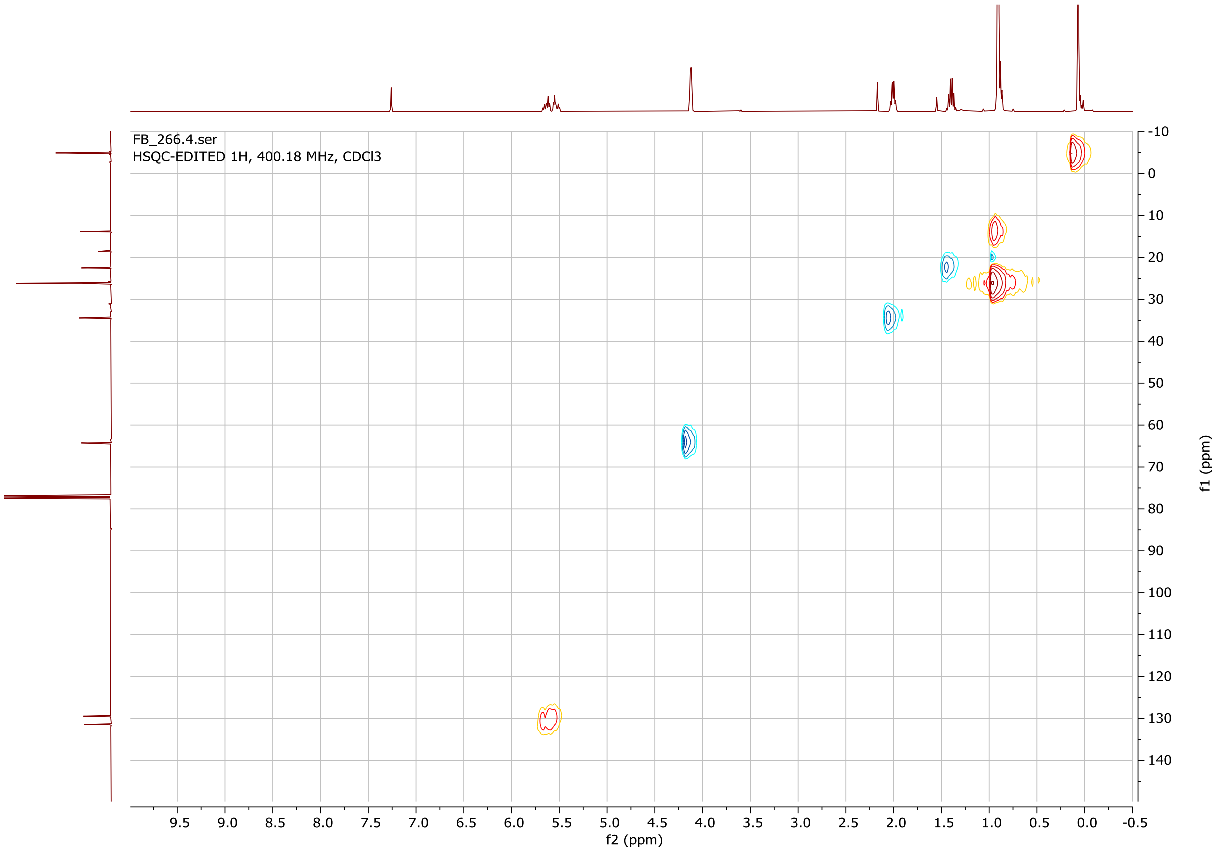
FB_266.4.ser
HSQC-EDITED 1H, 400.18 MHz, CDCl3
f2 (ppm)
f1 (ppm)

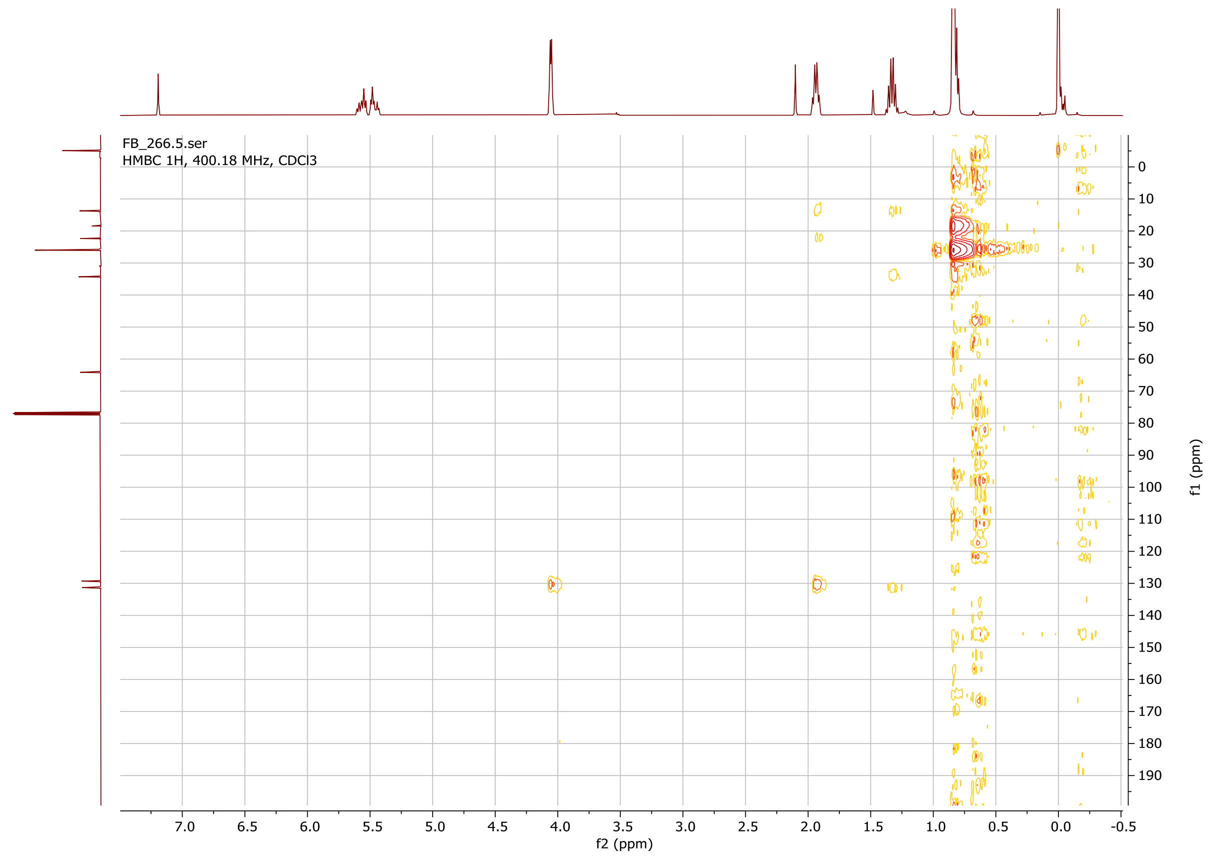

1-Phenyl-3-isopropoxy-1-propene **1s**

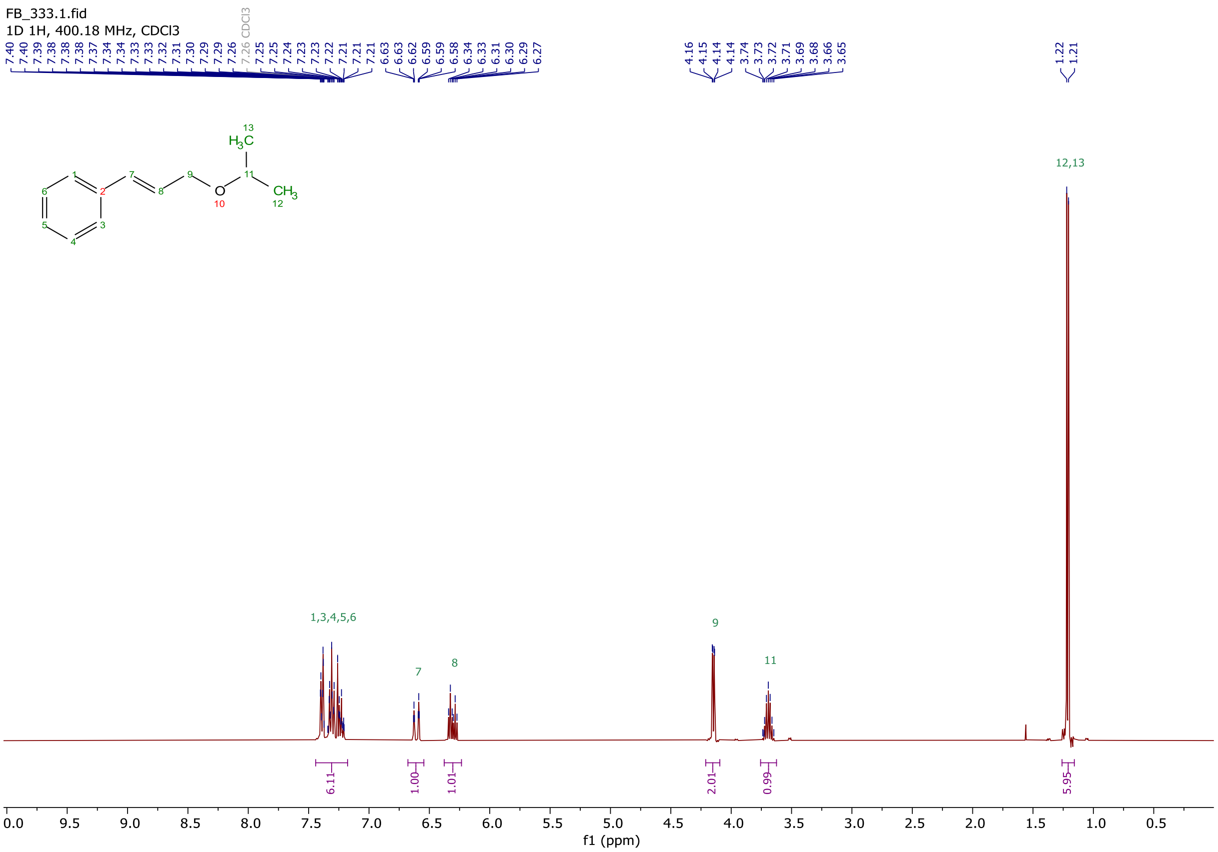

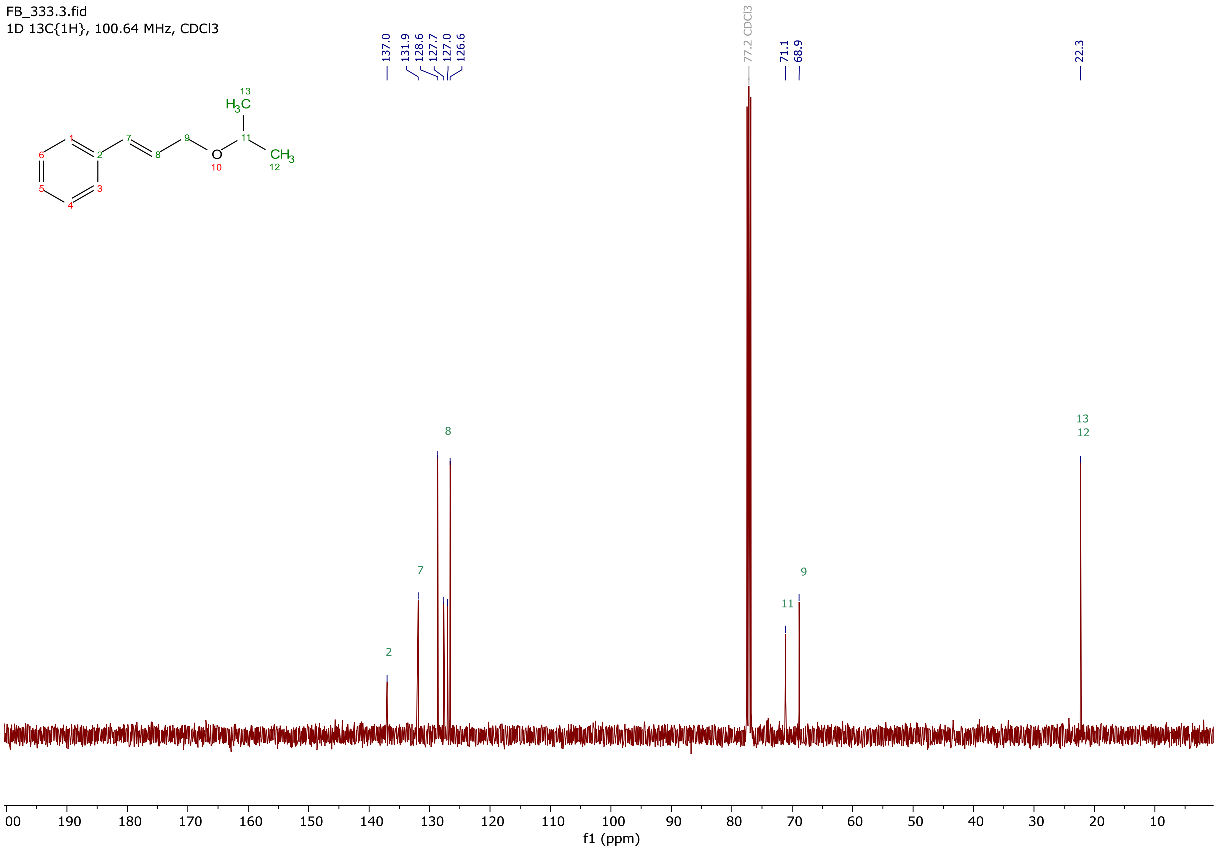
FB_333.3.fid
1D 13C{1H}, 100.64 MHz, CDCl3
137.0
131.9
128.6
127.7
127.0
126.6
77.2 CDCl3
71.1
68.9
22.3
f1 (ppm)

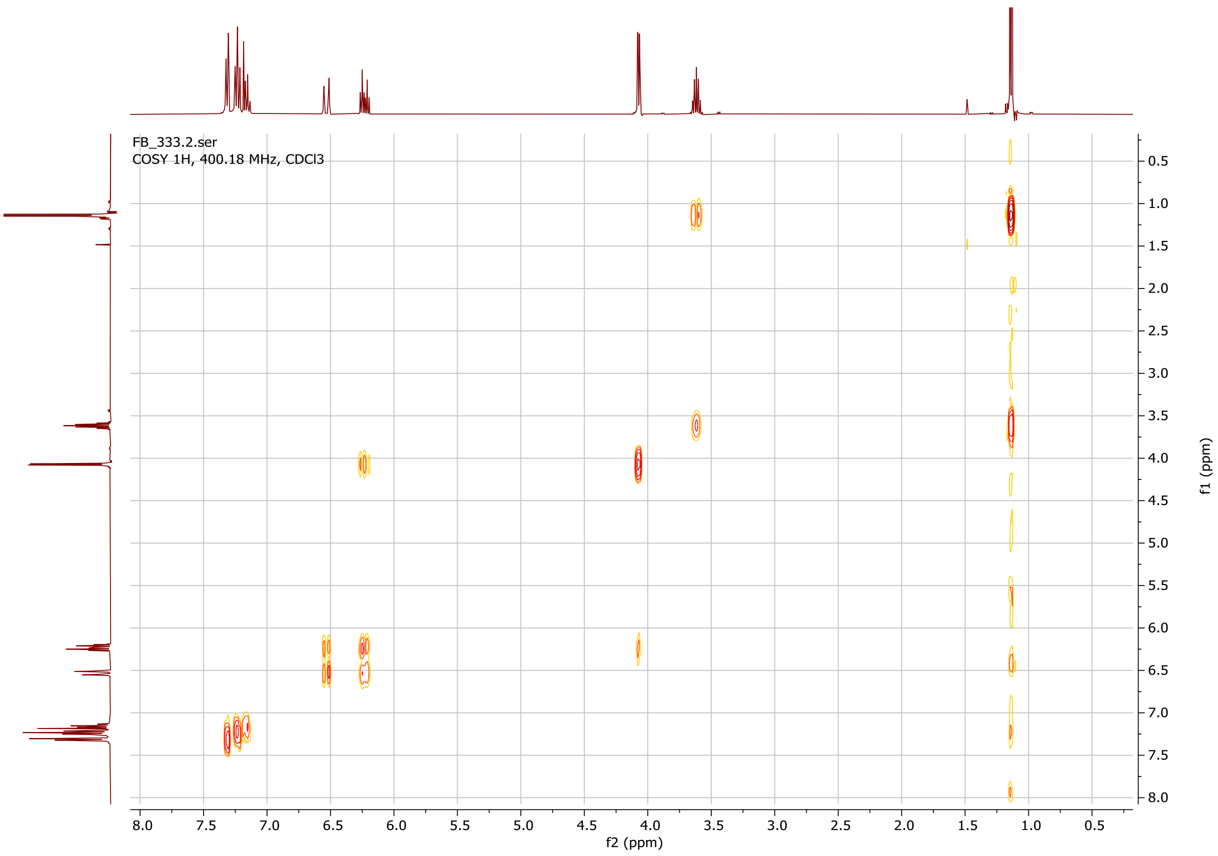
FB_333.2.ser
COSY 1H, 400.18 MHz, CDCl3
f2 (ppm)
f1 (ppm)

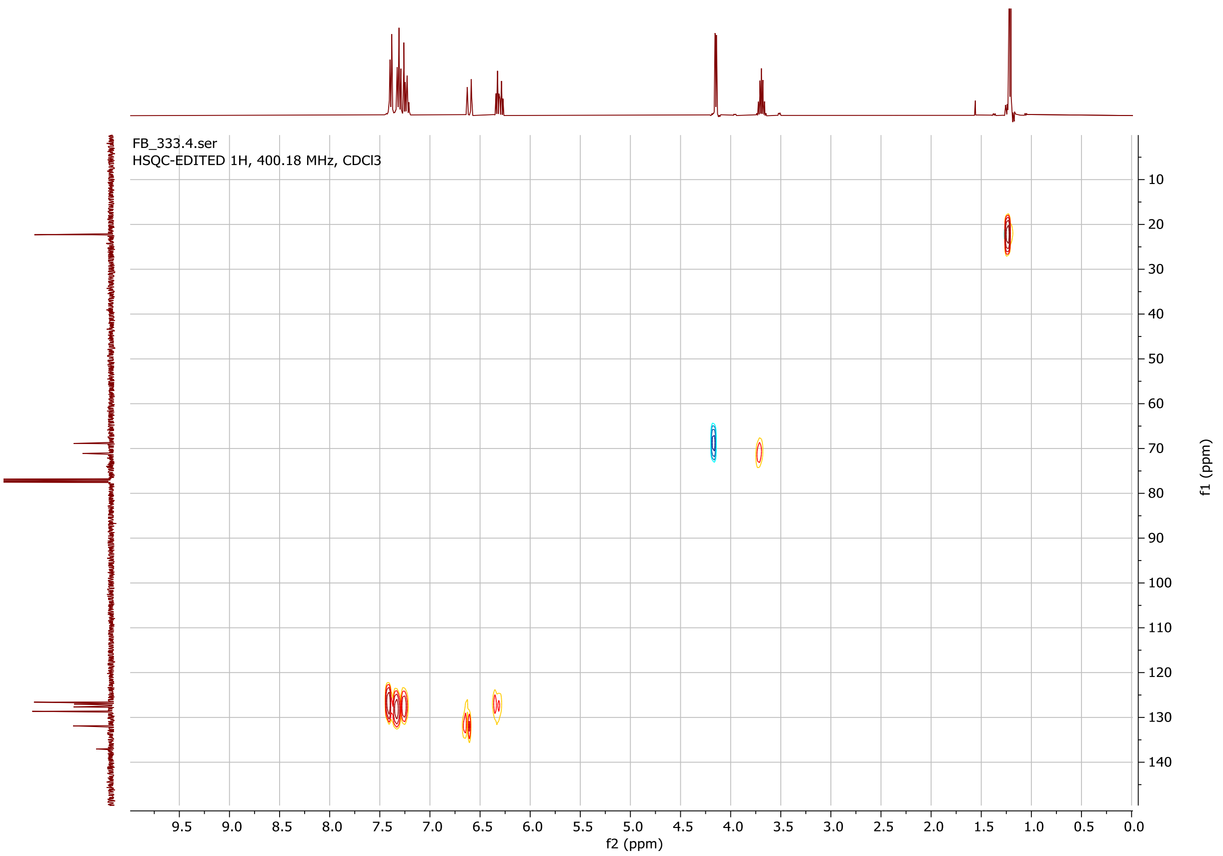
FB_333.4.ser
HSQC-EDITED 1H, 400.18 MHz, CDCl3
f2 (ppm)
f1 (ppm)

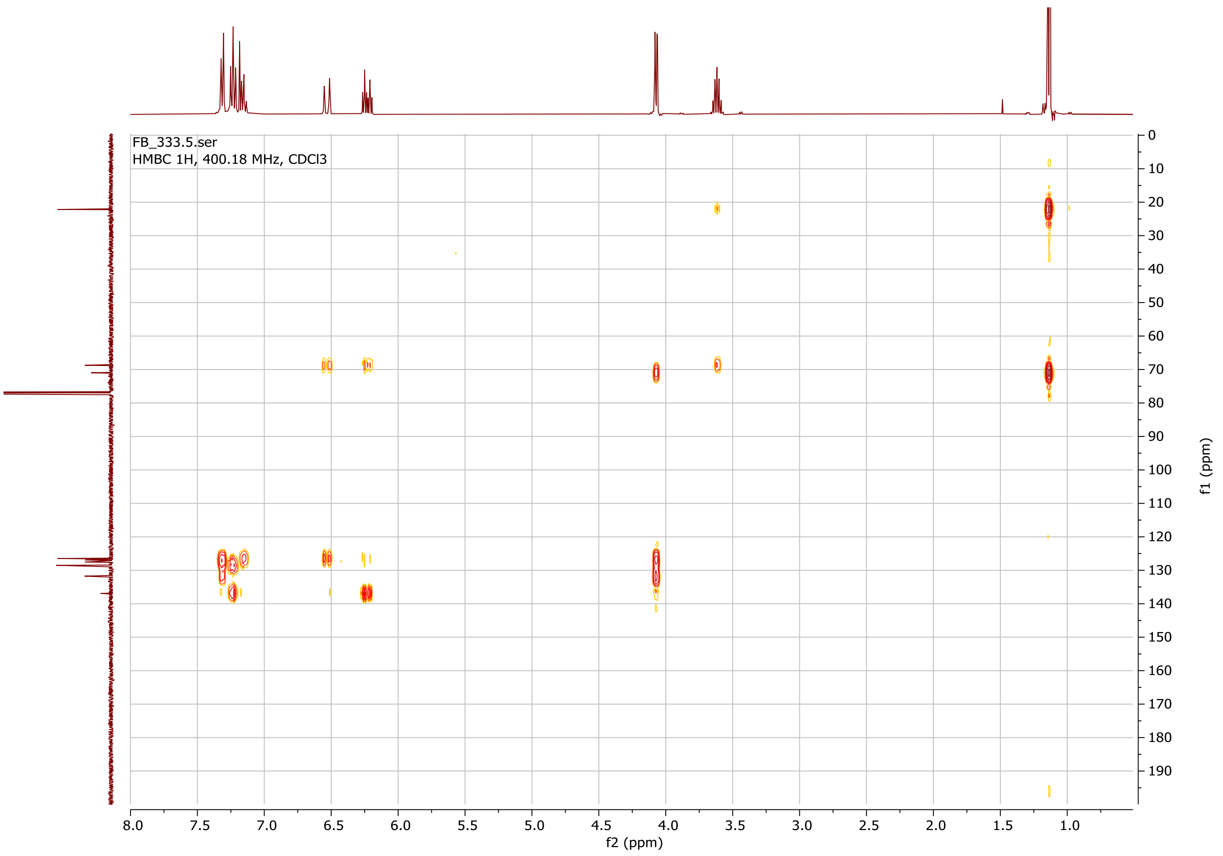
FB_333.5.ser
HMBC 1H, 400.18 MHz, CDCl3
f2 (ppm)
f1 (ppm)

N,*N*-Dimethylcinnamamide **1u**

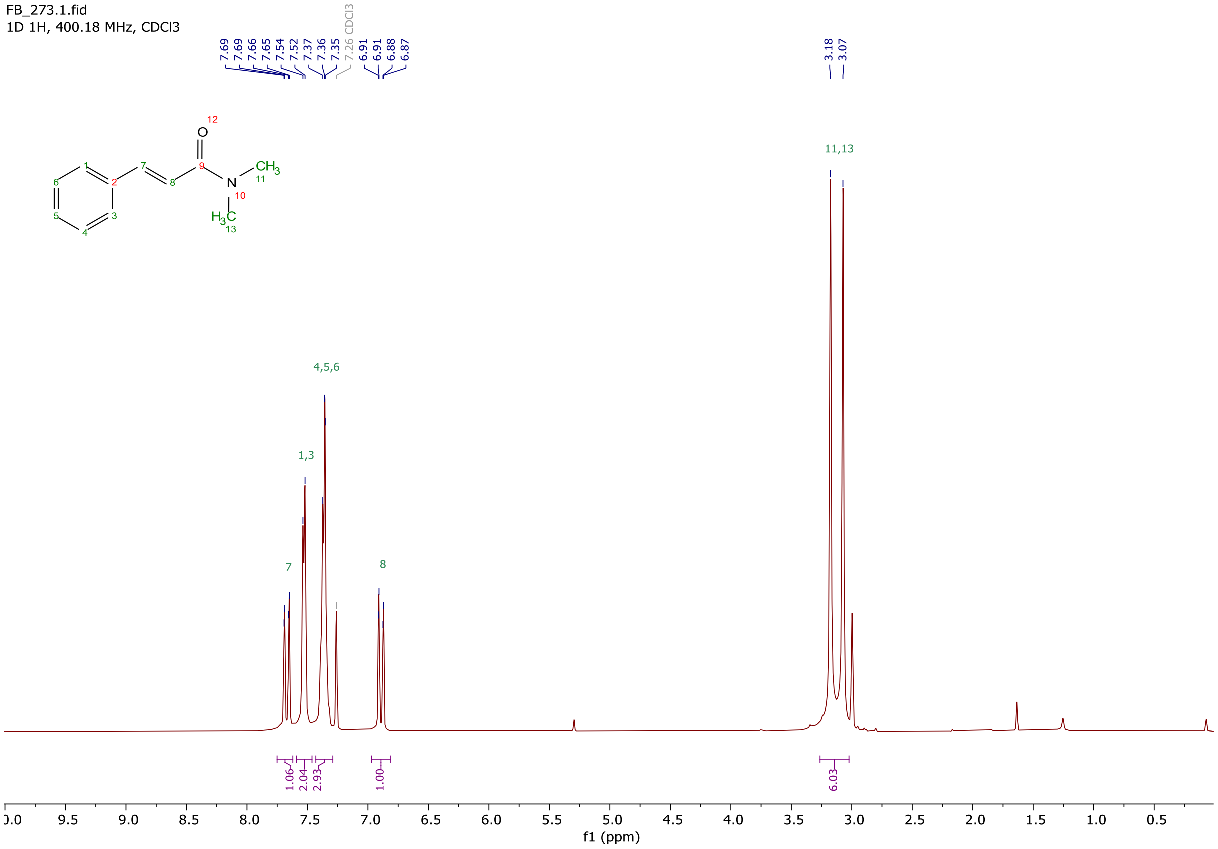

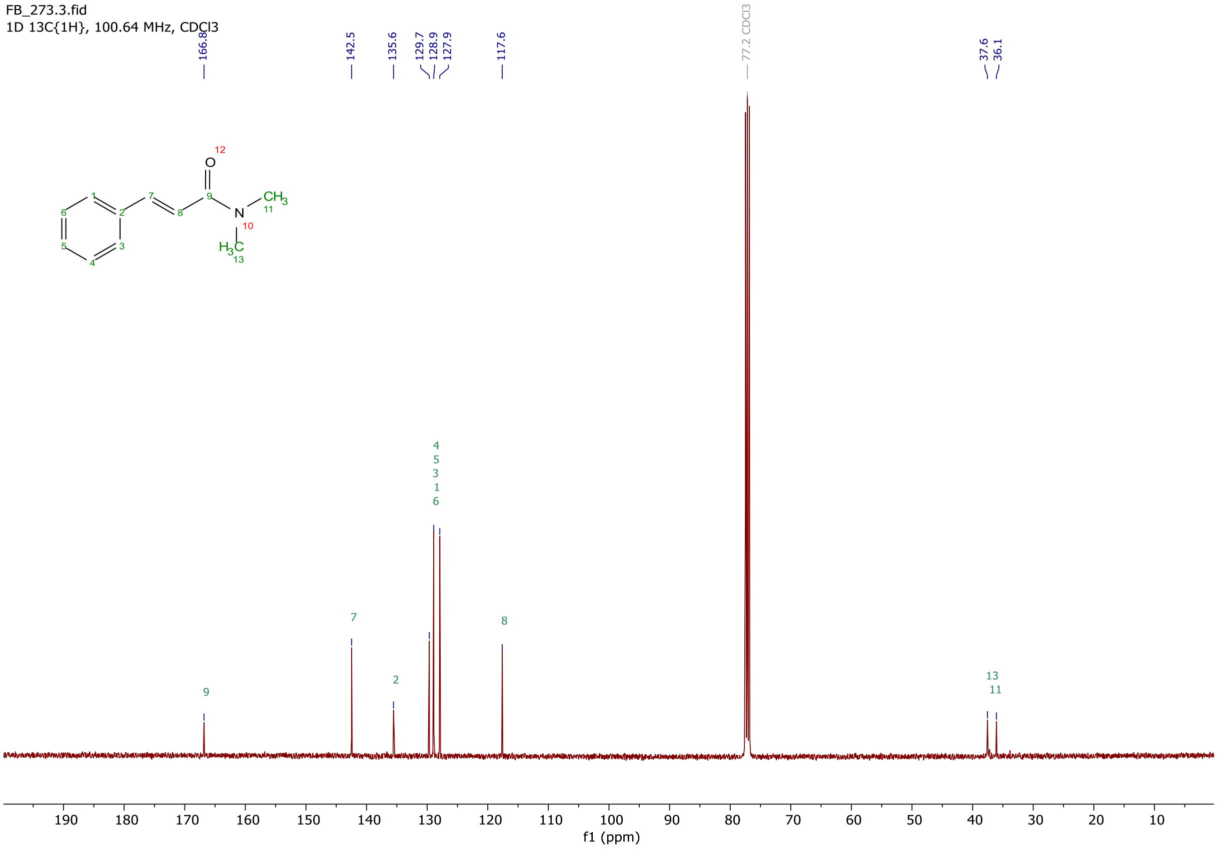

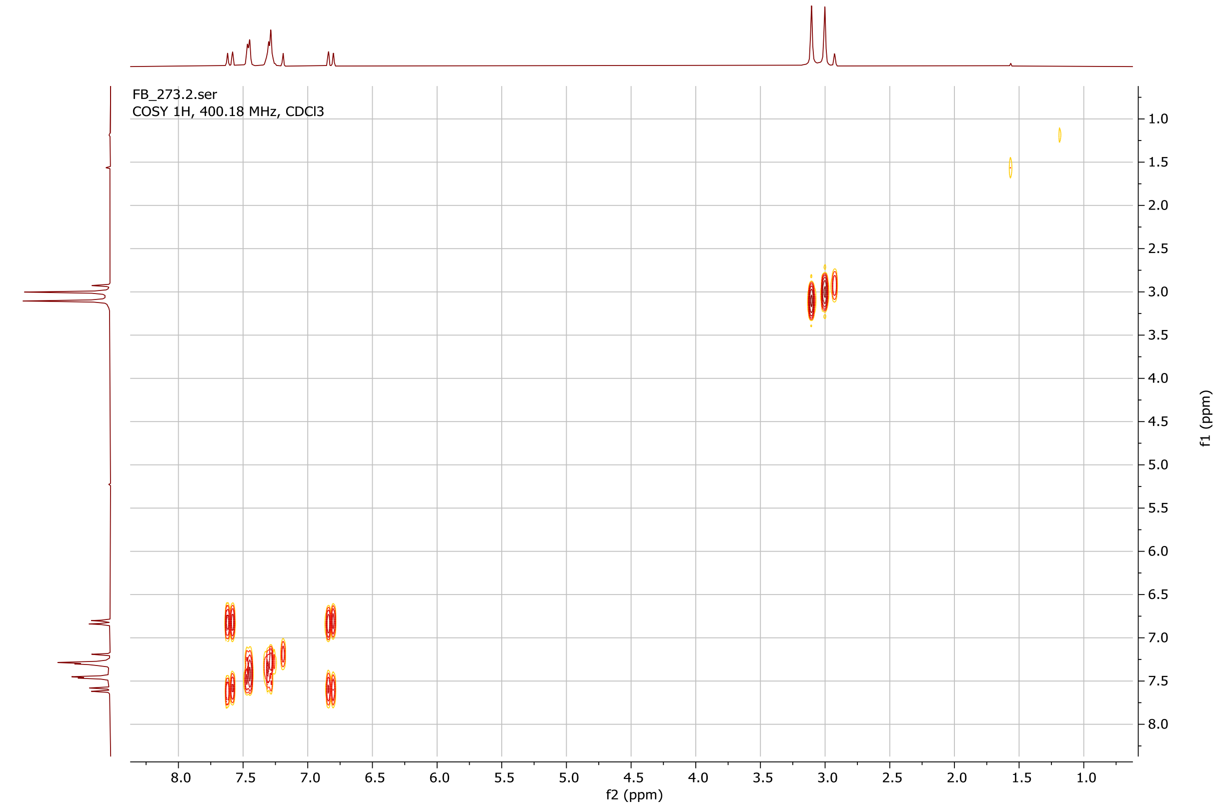

FB_273.2.ser
COSY 1H, 400.18 MHz, CDCl3
f2 (ppm)
f1 (ppm)

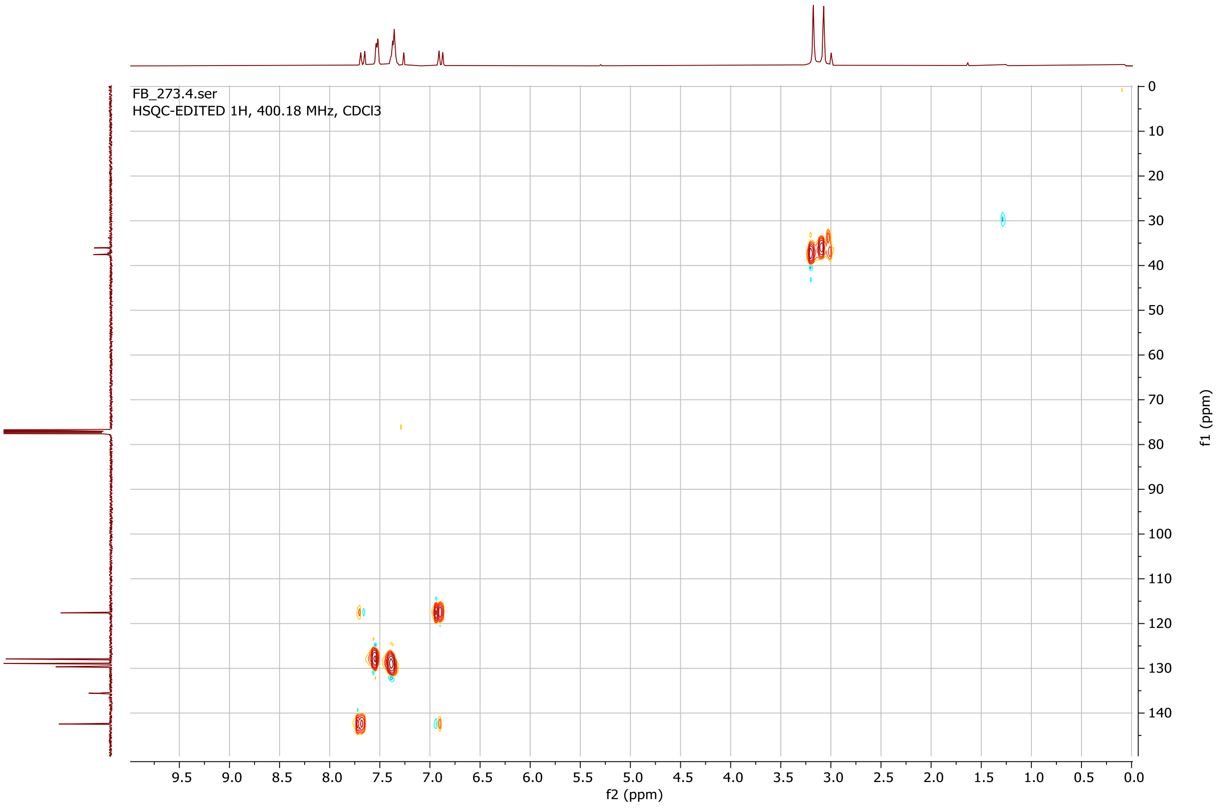

FB_273.4.ser
HSQC-EDITED 1H, 400.18 MHz, CDCl3
f2 (ppm)
f1 (ppm)

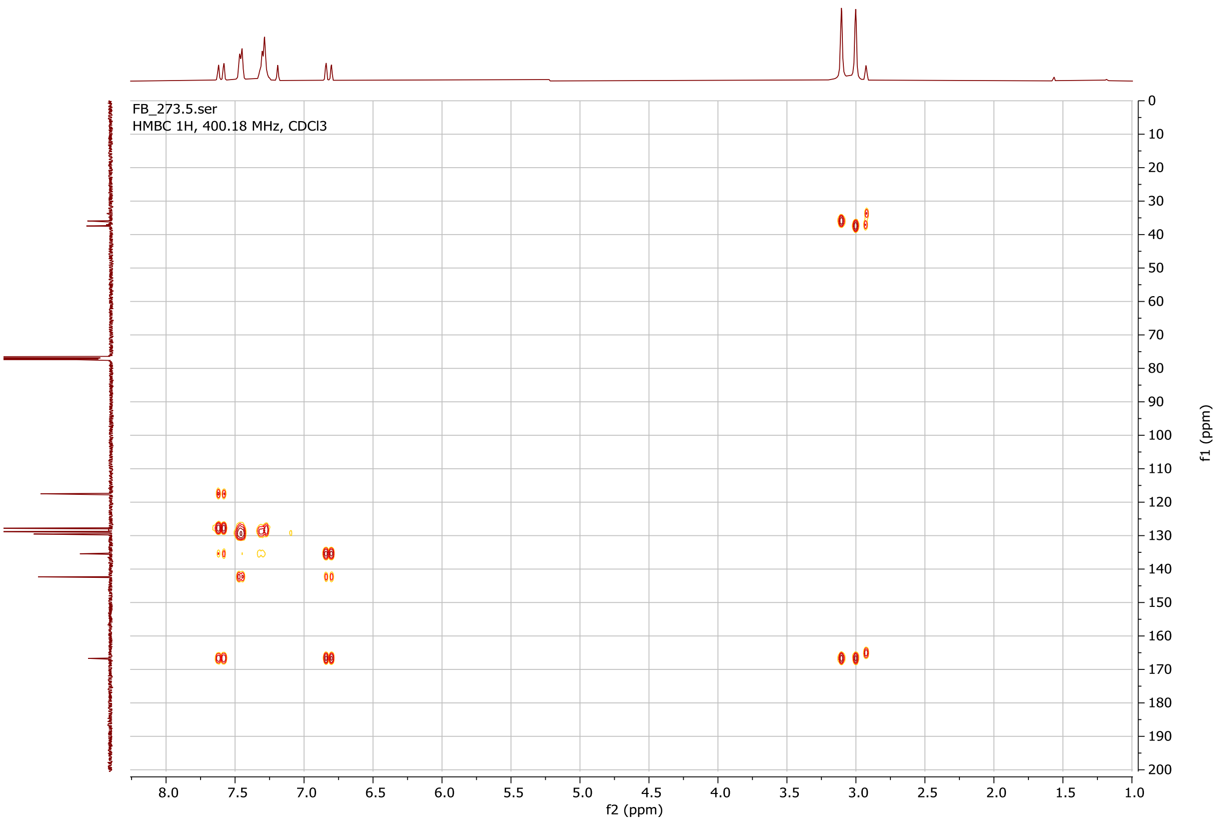

2-Methyl-3-phenyloxirane **2q**

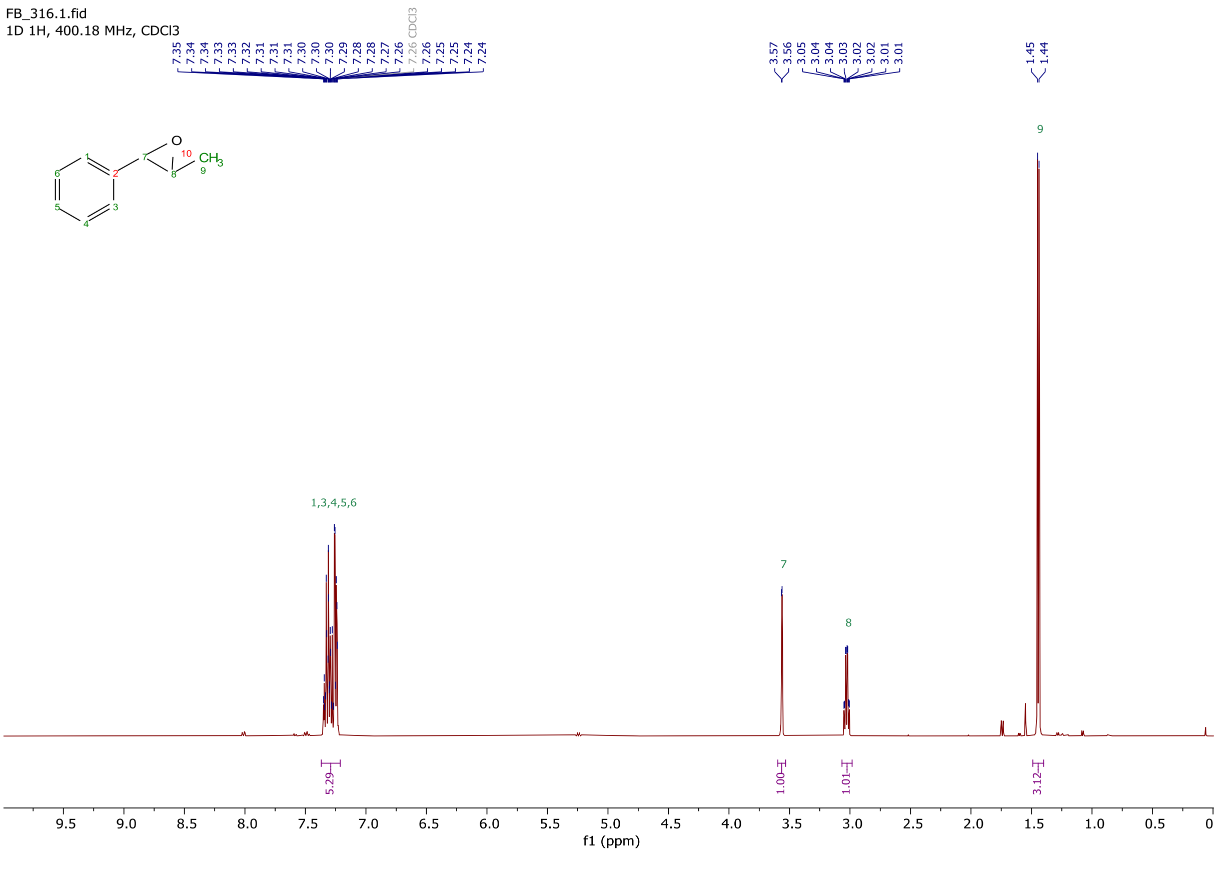

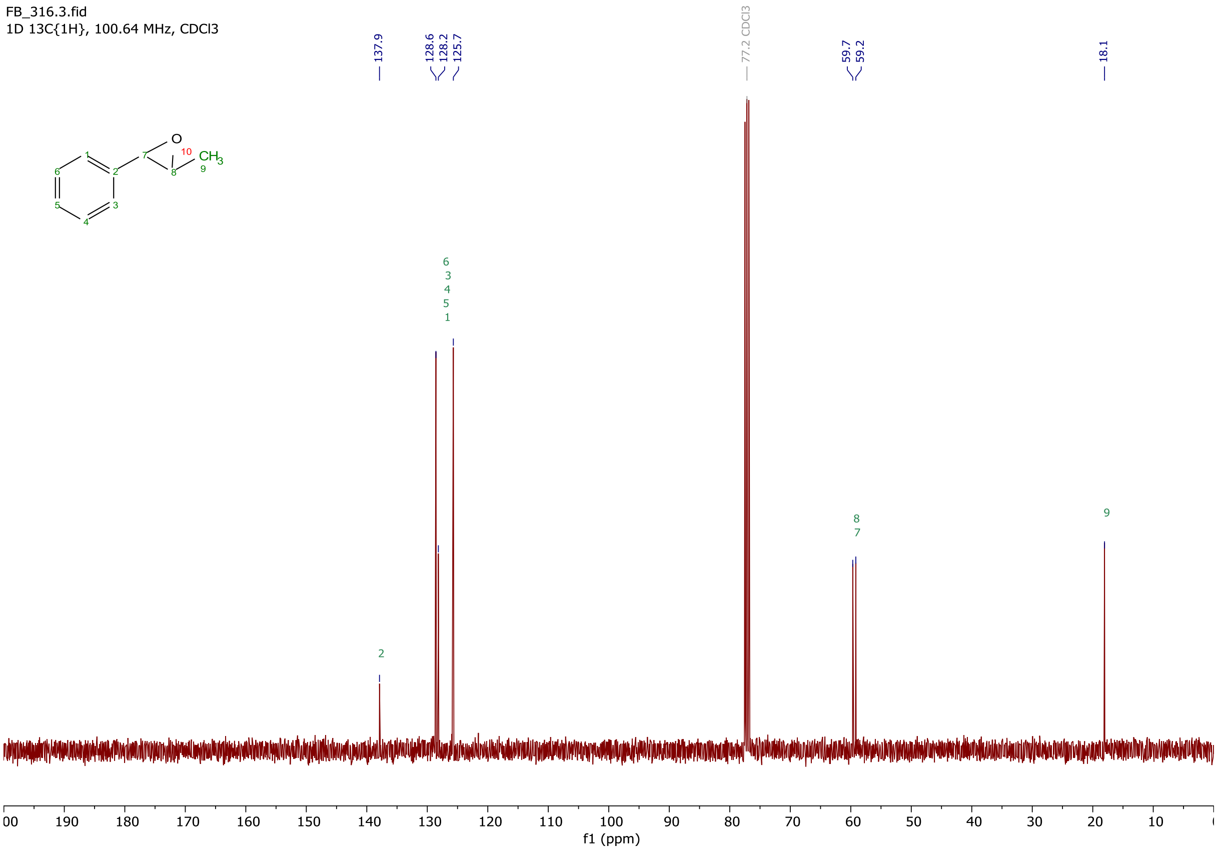
FB_316.3.fid
1D 13C{1H}, 100.64 MHz, CDCl3
137.9
128.6
128.2
125.7
77.2 CDCl3
59.7
59.2
18.1
O
CH3
f1 (ppm)

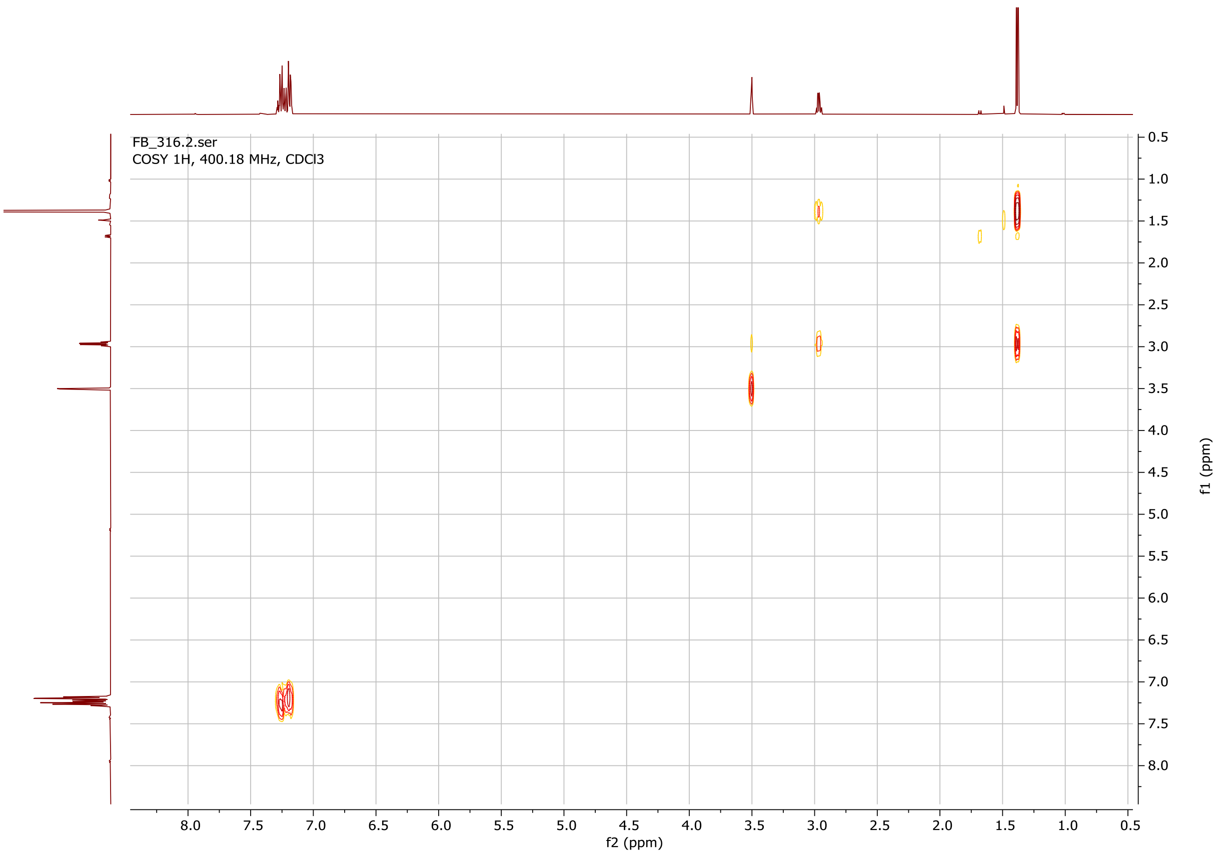
FB_316.2.ser
COSY 1H, 400.18 MHz, CDCl3
f2 (ppm)
f1 (ppm)

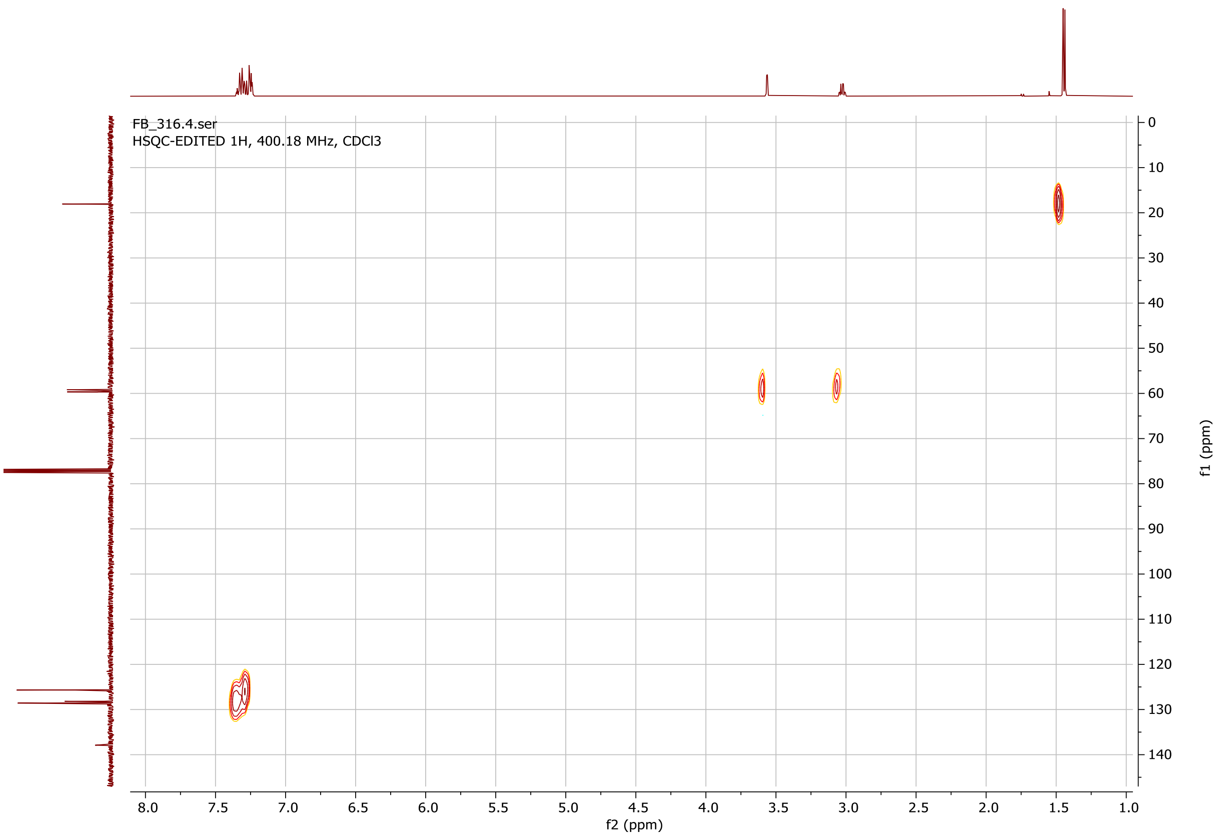
FB_316.4.ser
HSQC-EDITED 1H, 400.18 MHz, CDCl3
f2 (ppm)
f1 (ppm)

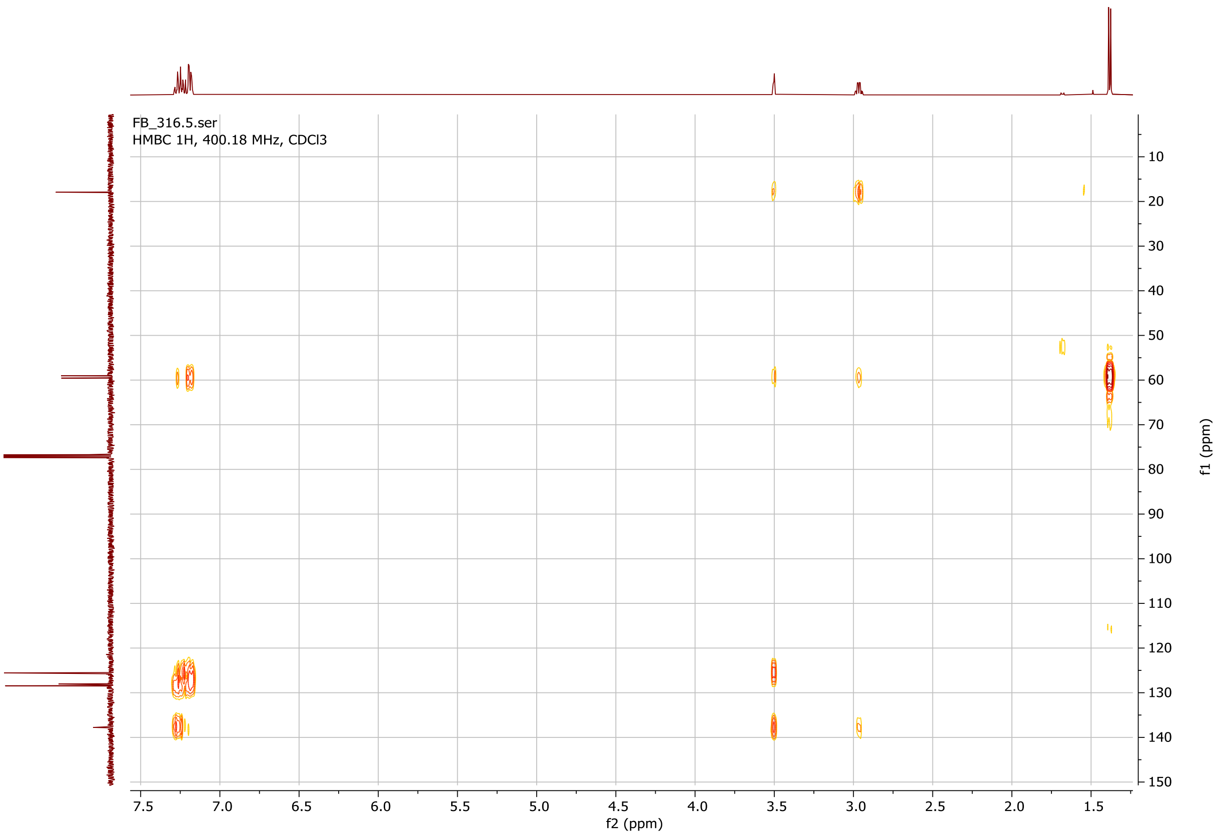
FB_316.5.ser
HMBC 1H, 400.18 MHz, CDCl3
f2 (ppm)
f1 (ppm)

1-(3-Phenyloxiran-2-yl)ethan-1-one **2r**

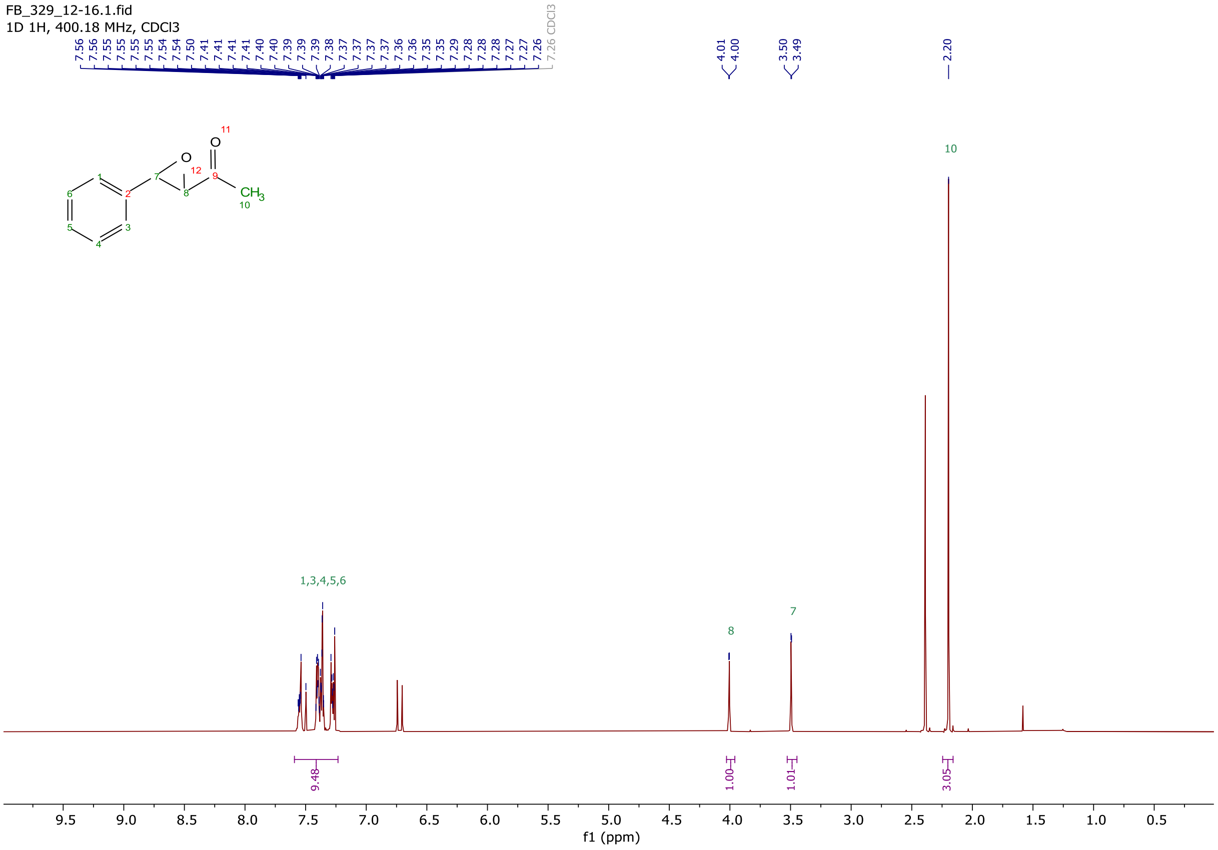

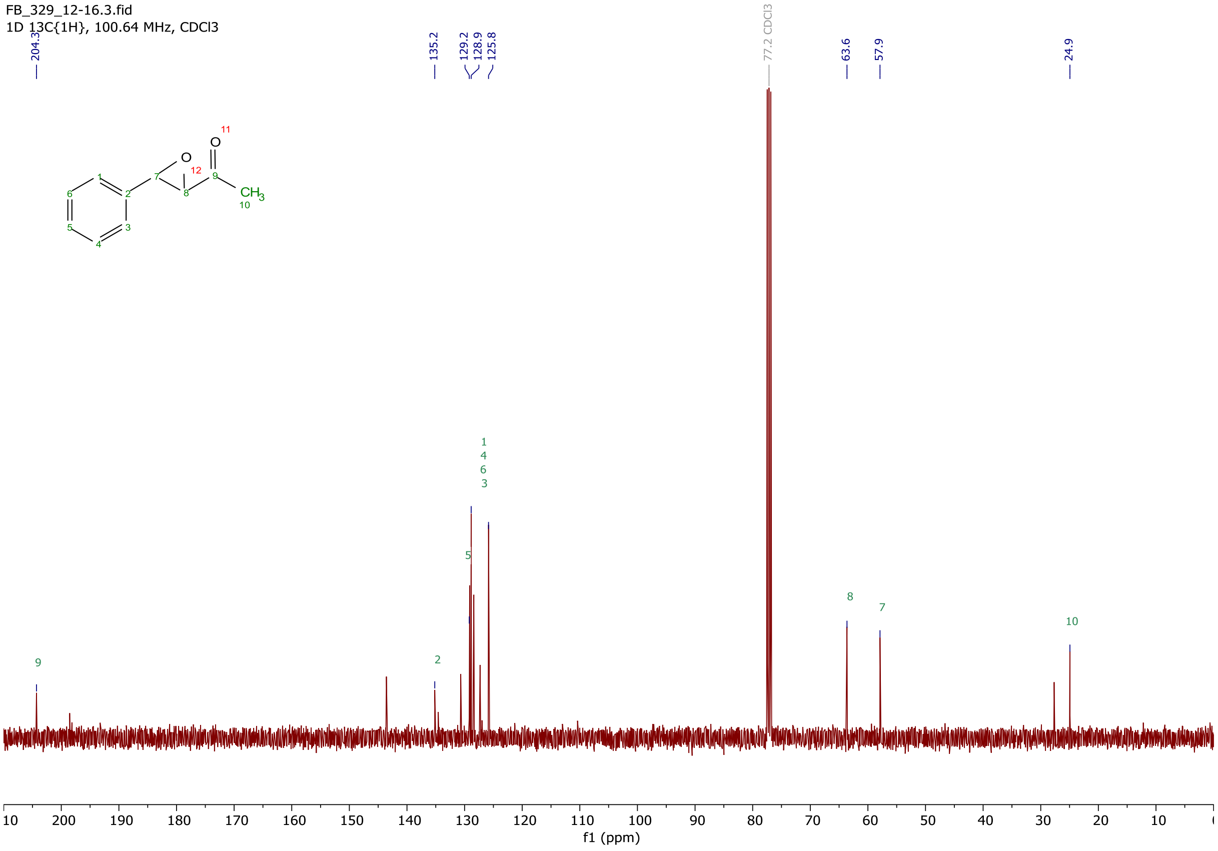

2-(Isopropoxymethyl)-3-phenyloxirane **2s**

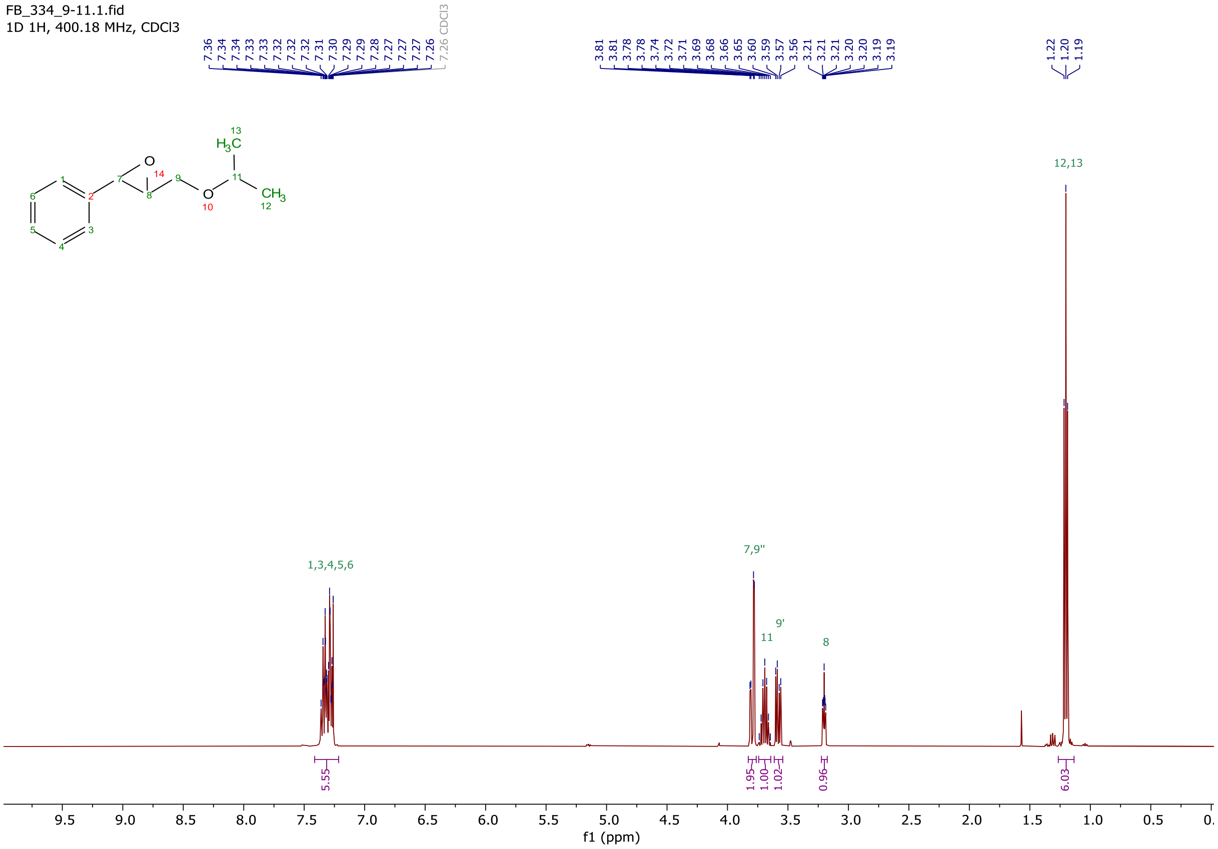

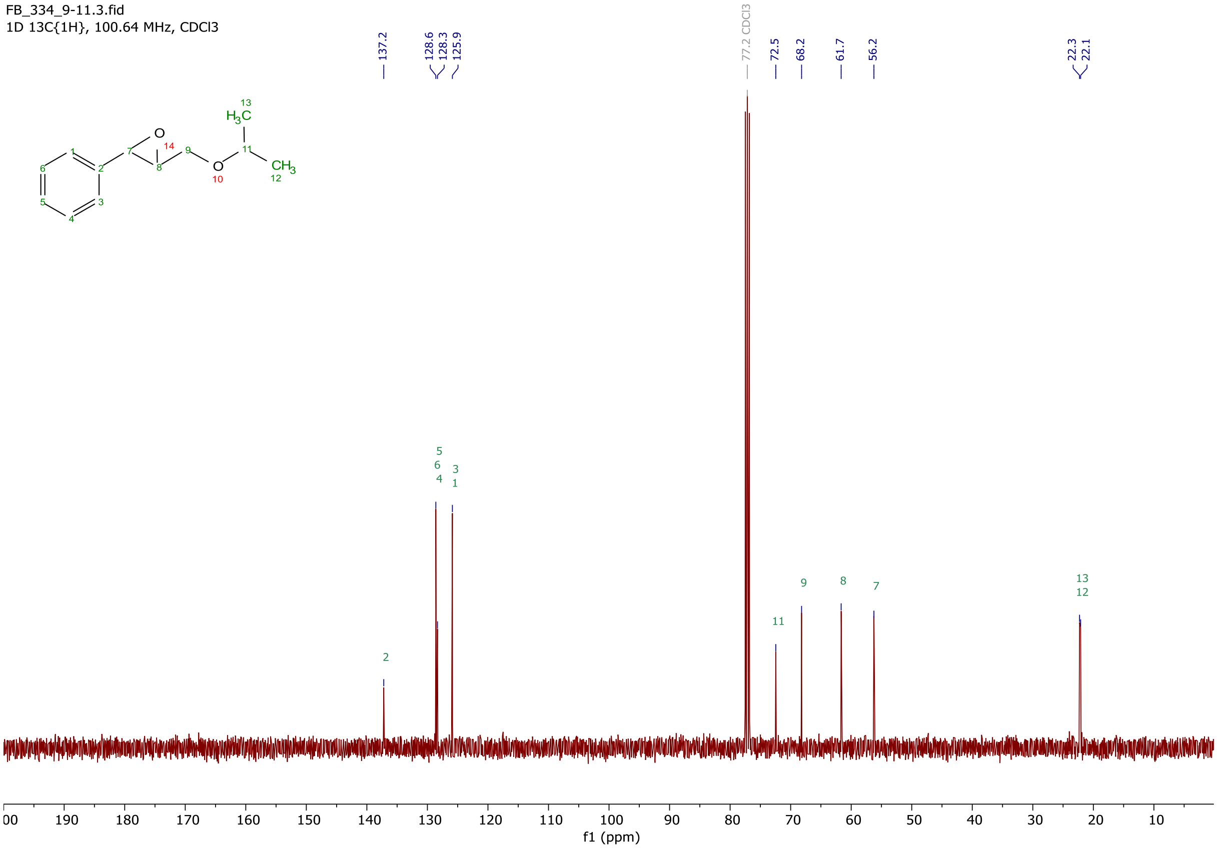

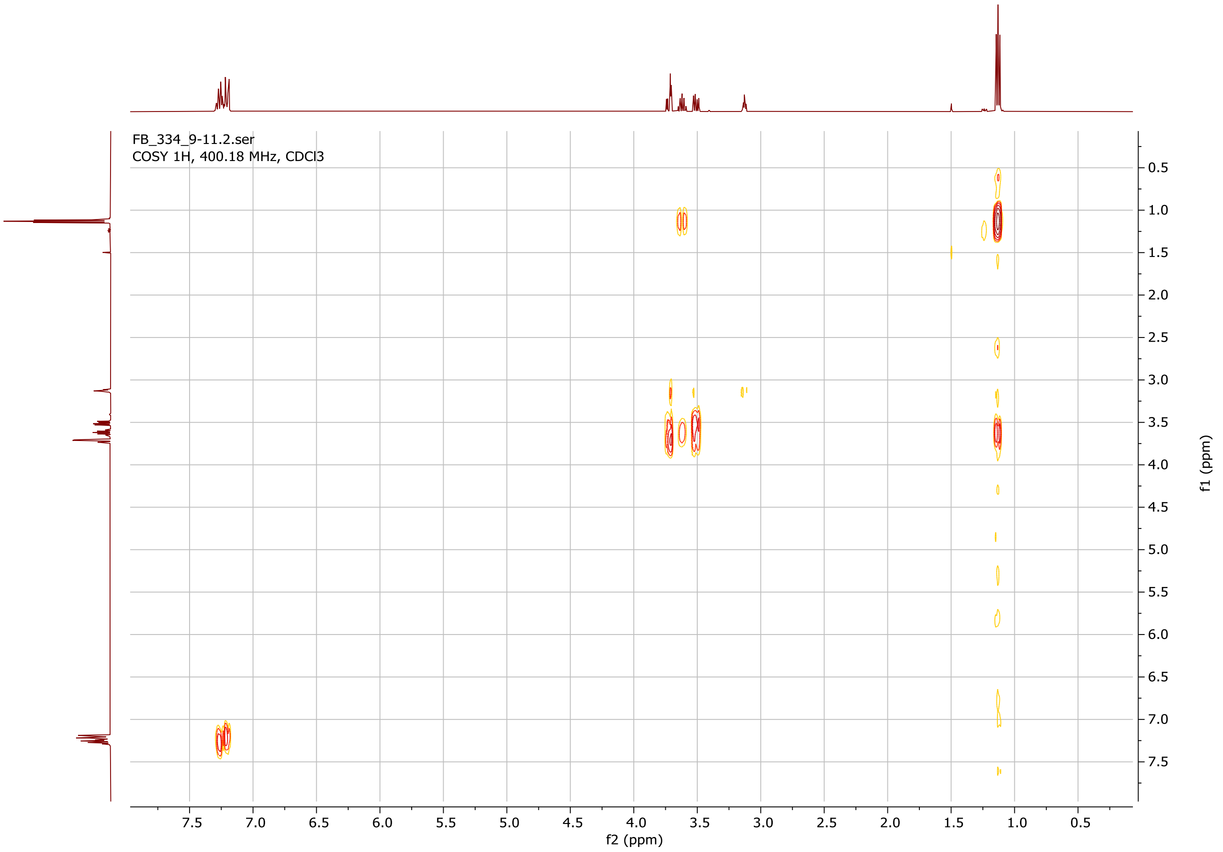
FB_334_9-11.2.ser
COSY 1H, 400.18 MHz, CDCl3
f2 (ppm)
f1 (ppm)

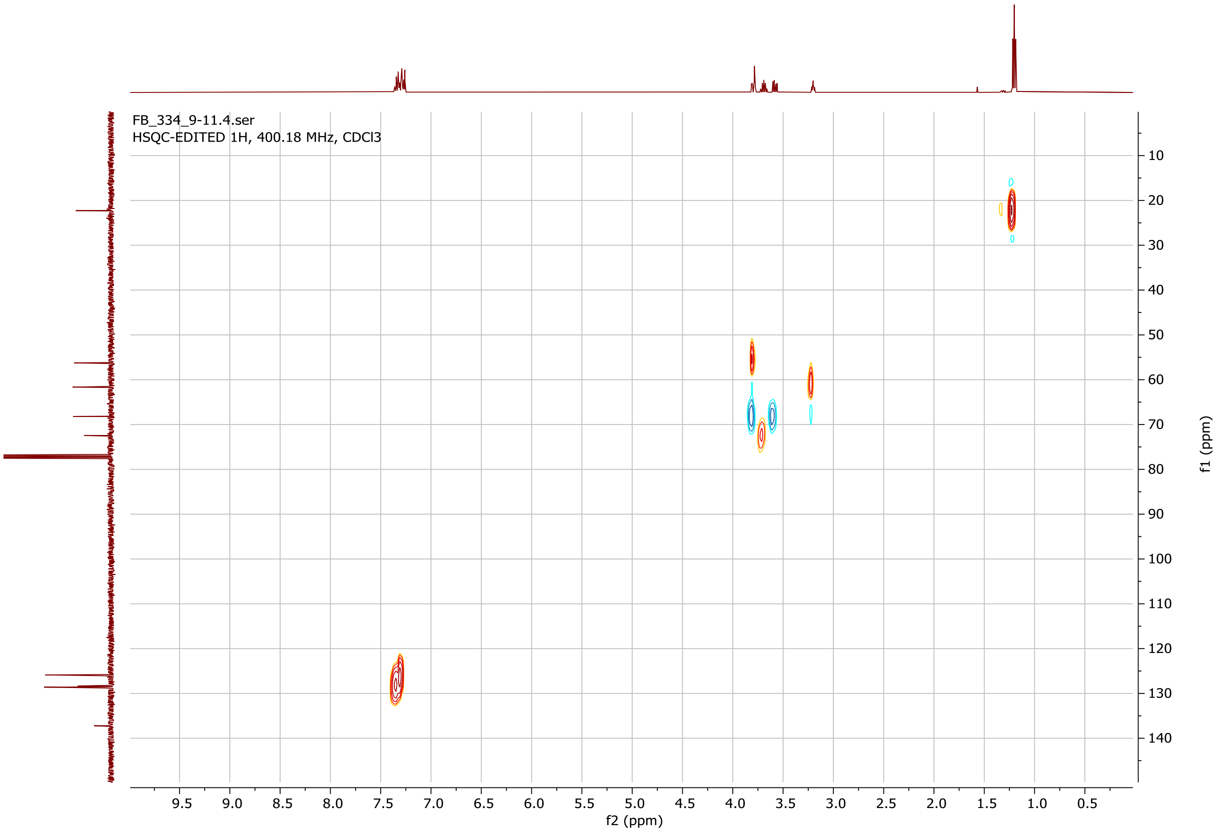
FB_334_9-11.4.ser
HSQC-EDITED 1H, 400.18 MHz, CDCl3
f2 (ppm)
f1 (ppm)

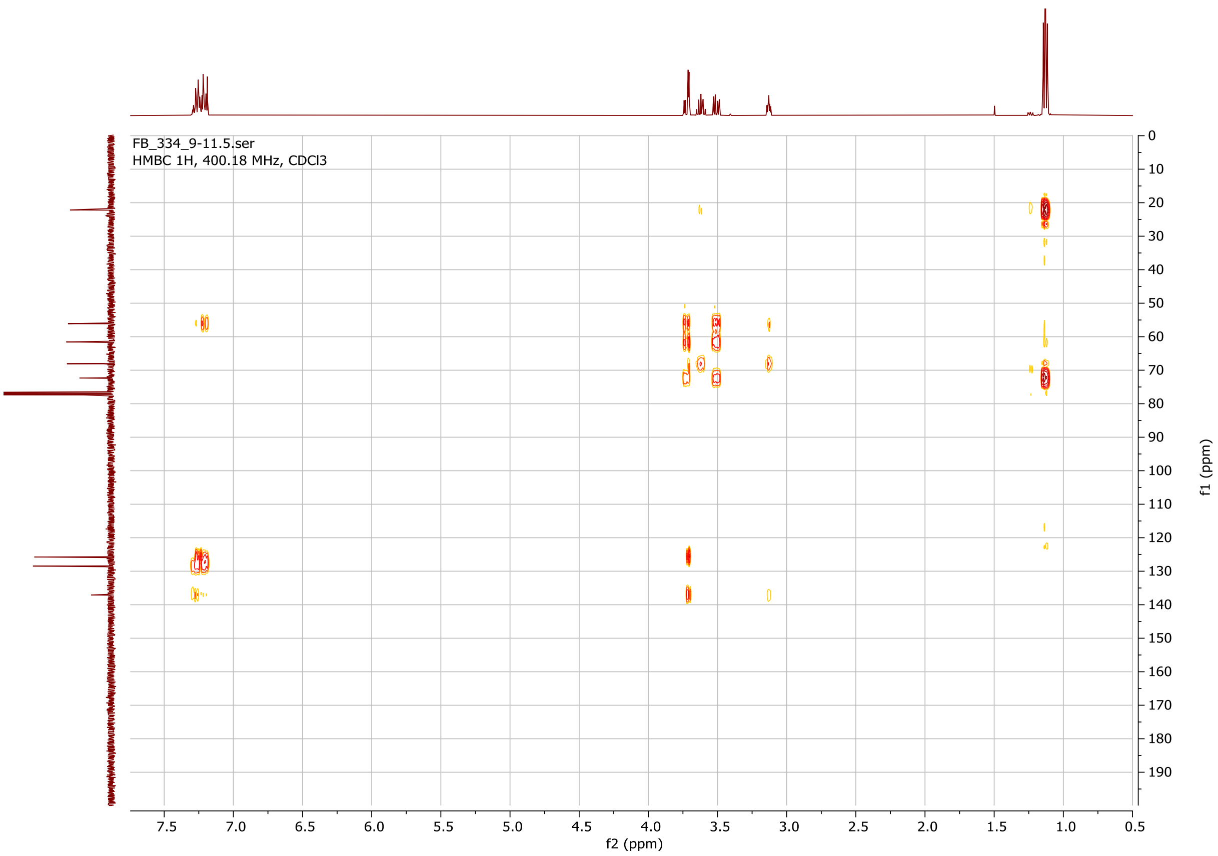

2-Iodo-2,3-dihydro-1*H*-inden-1-ol **3a**

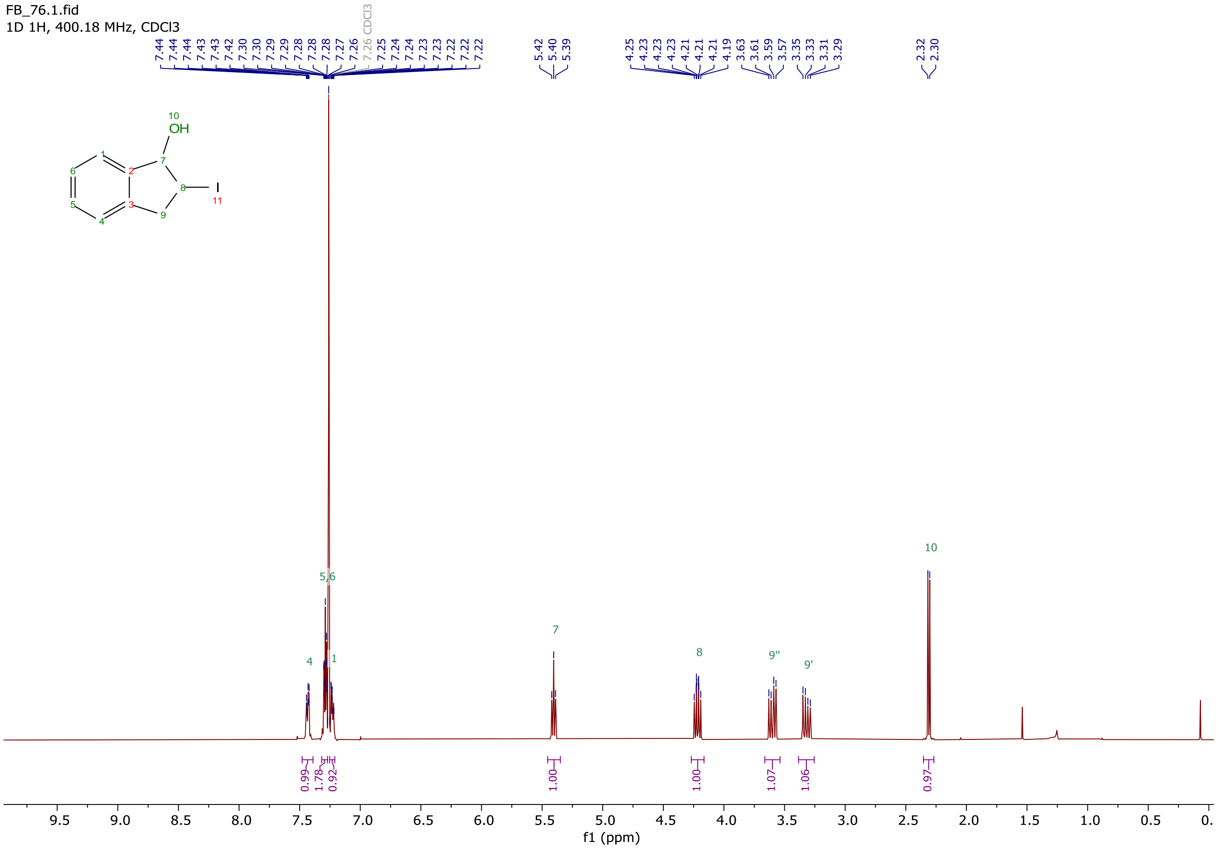

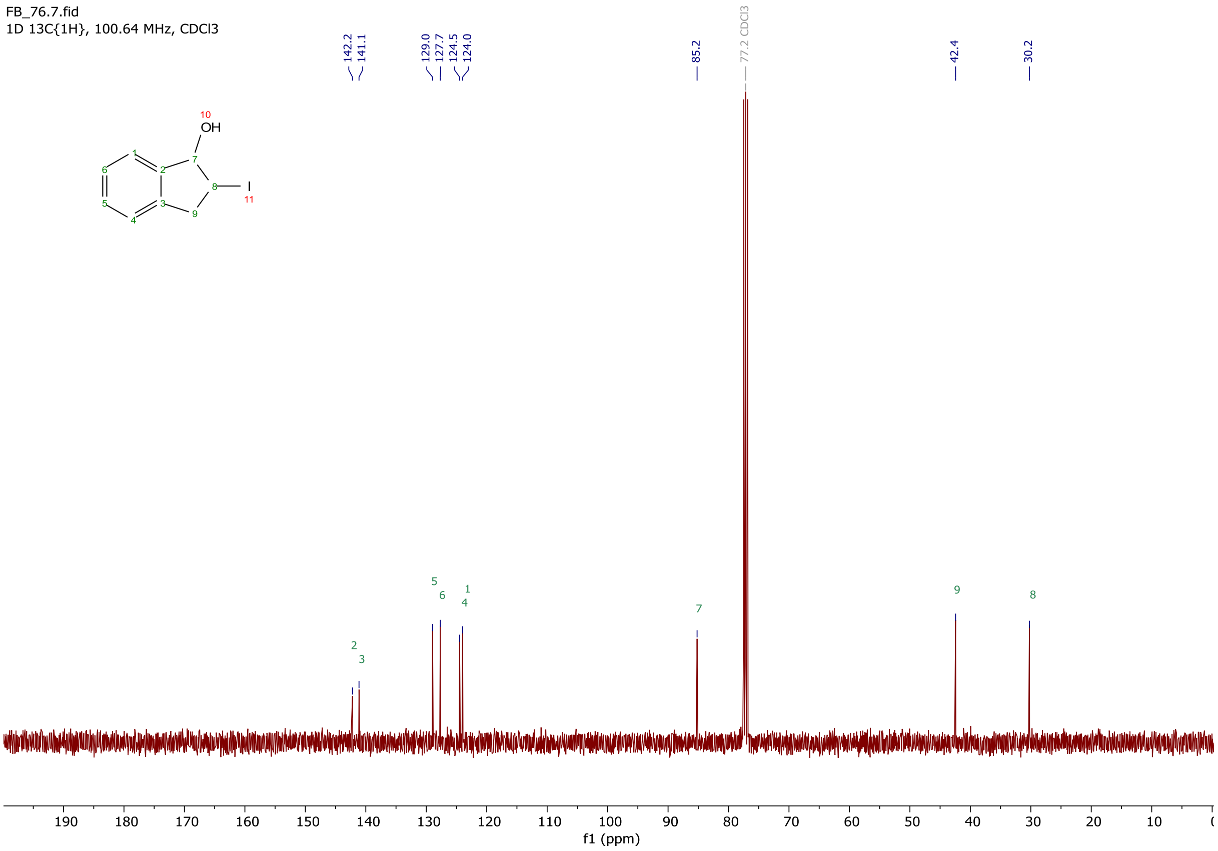
FB_76.7.fid
1D 13C{1H}, 100.64 MHz, CDCl3
142.2
141.1
129.0
127.7
124.5
124.0
85.2
77.2 CDCl3
42.4
30.2
OH
I
f1 (ppm)

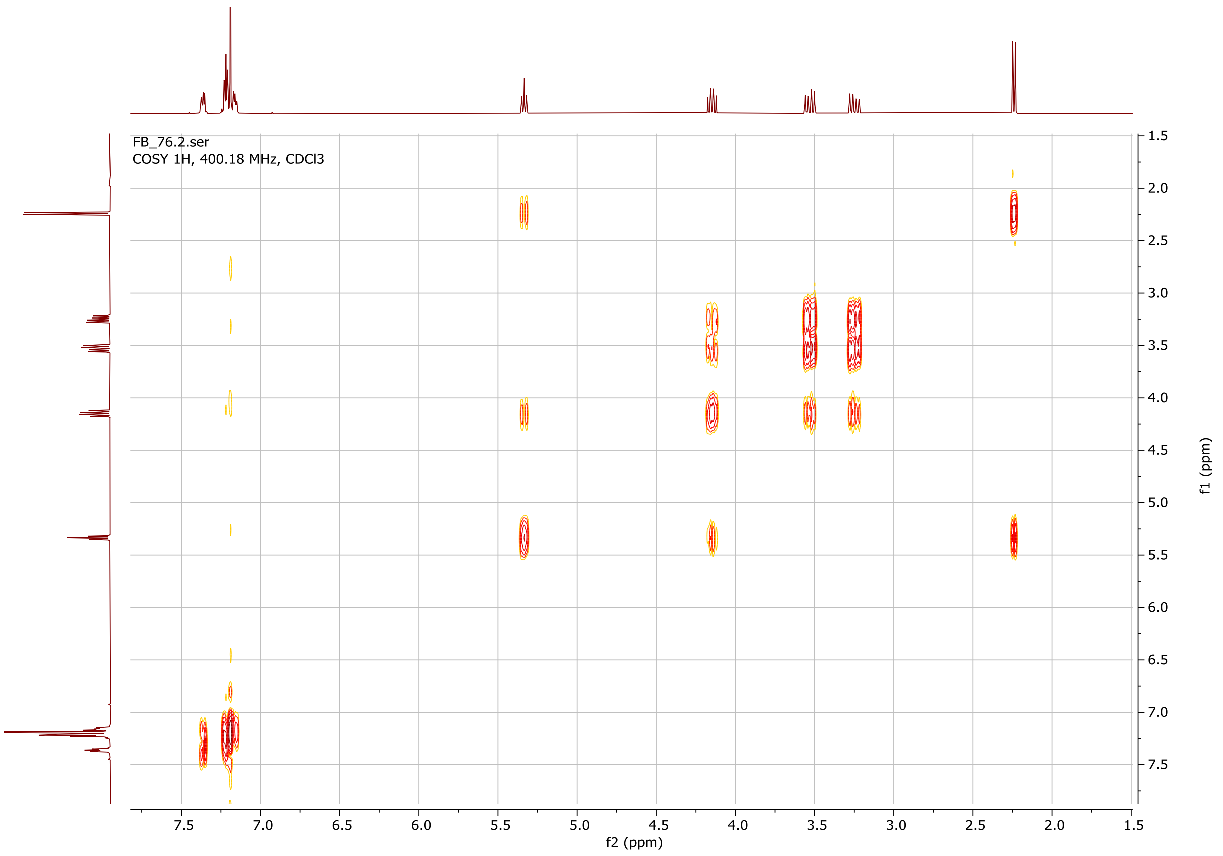
FB_76.2.ser
COSY 1H, 400.18 MHz, CDCl3
f2 (ppm)
f1 (ppm)

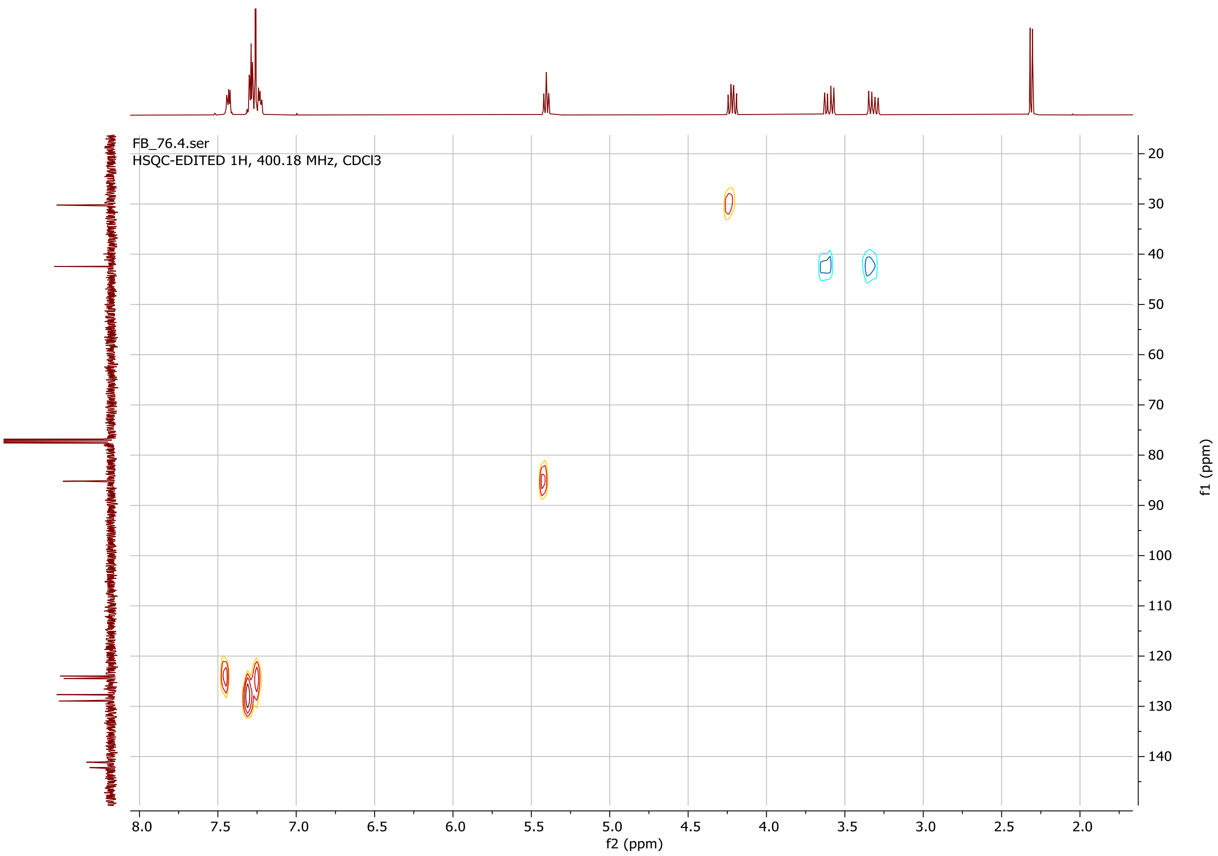

2-Iodocyclopentanol **3b**

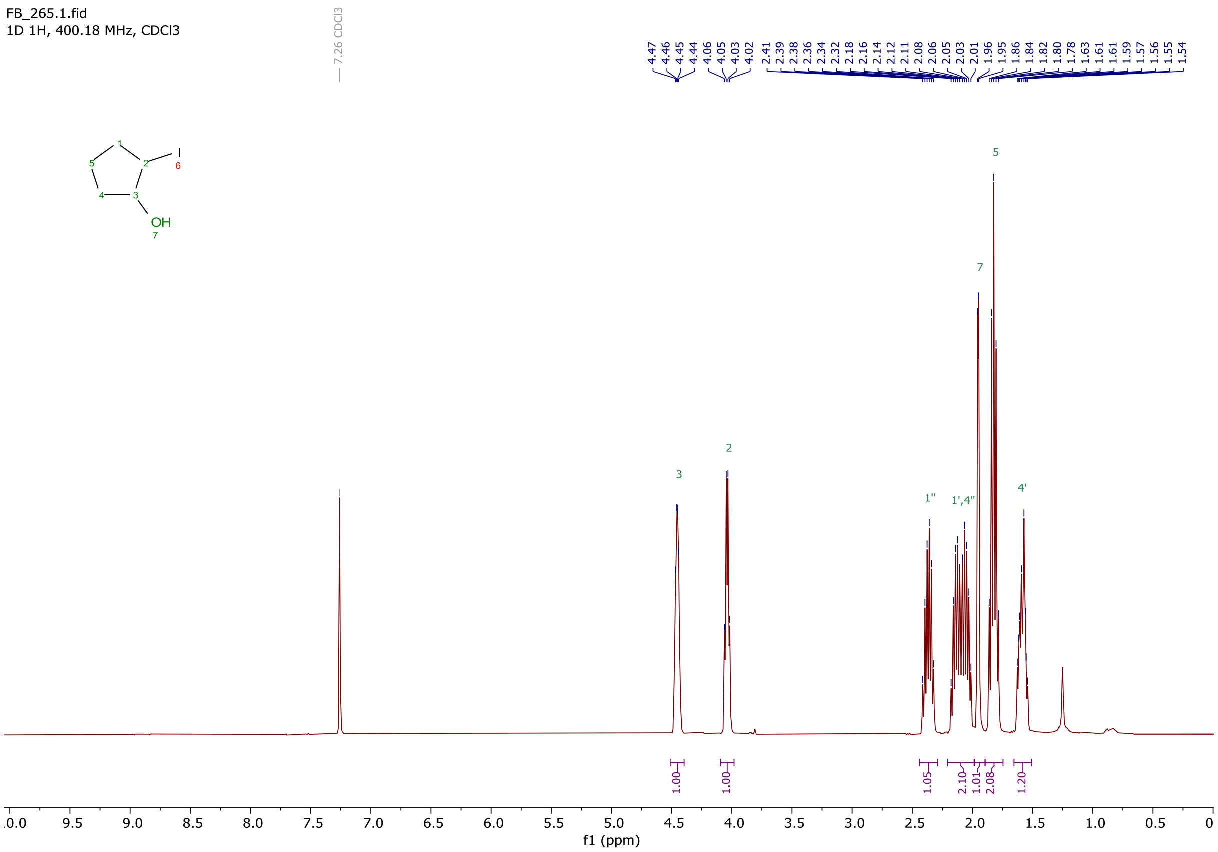

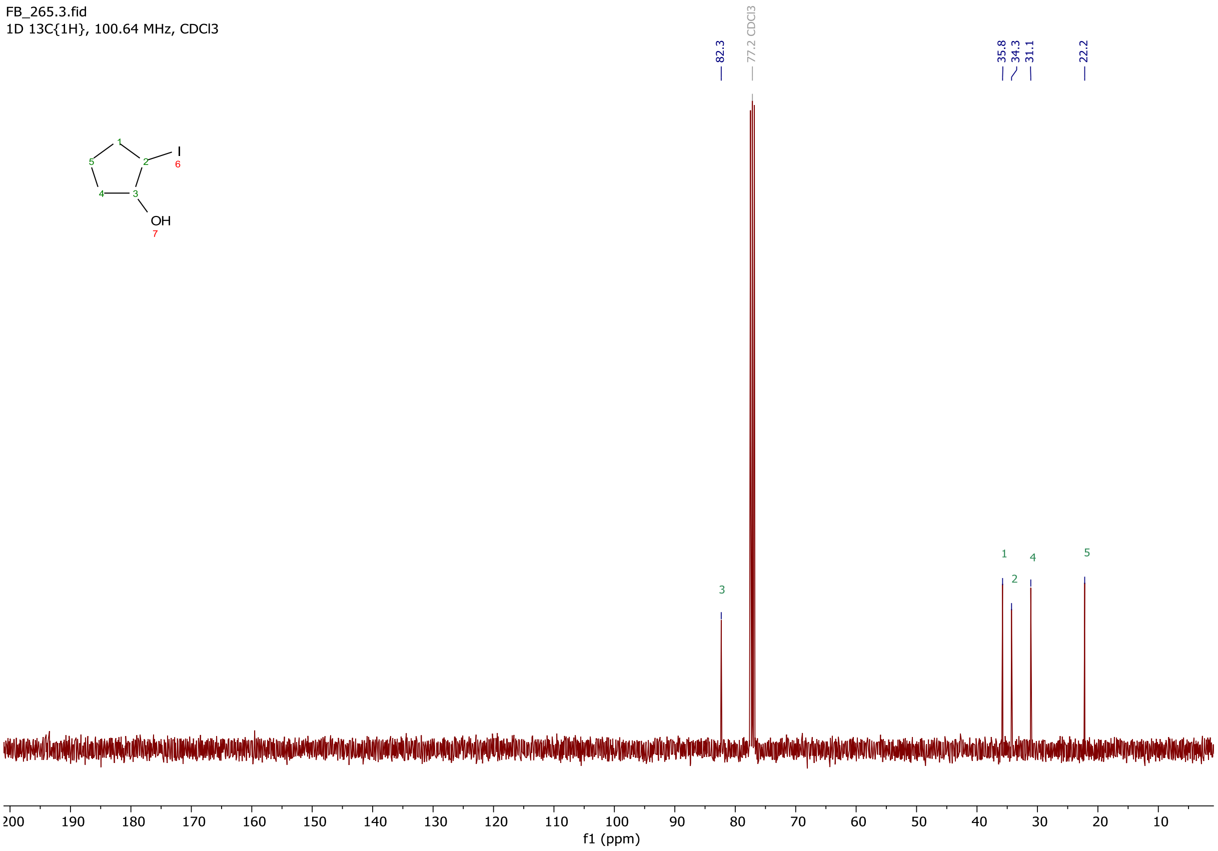
FB_265.3.fid
1D 13C{1H}, 100.64 MHz, CDCl3
82.3
77.2 CDCl3
35.8
34.3
31.1
22.2
OH
f1 (ppm)

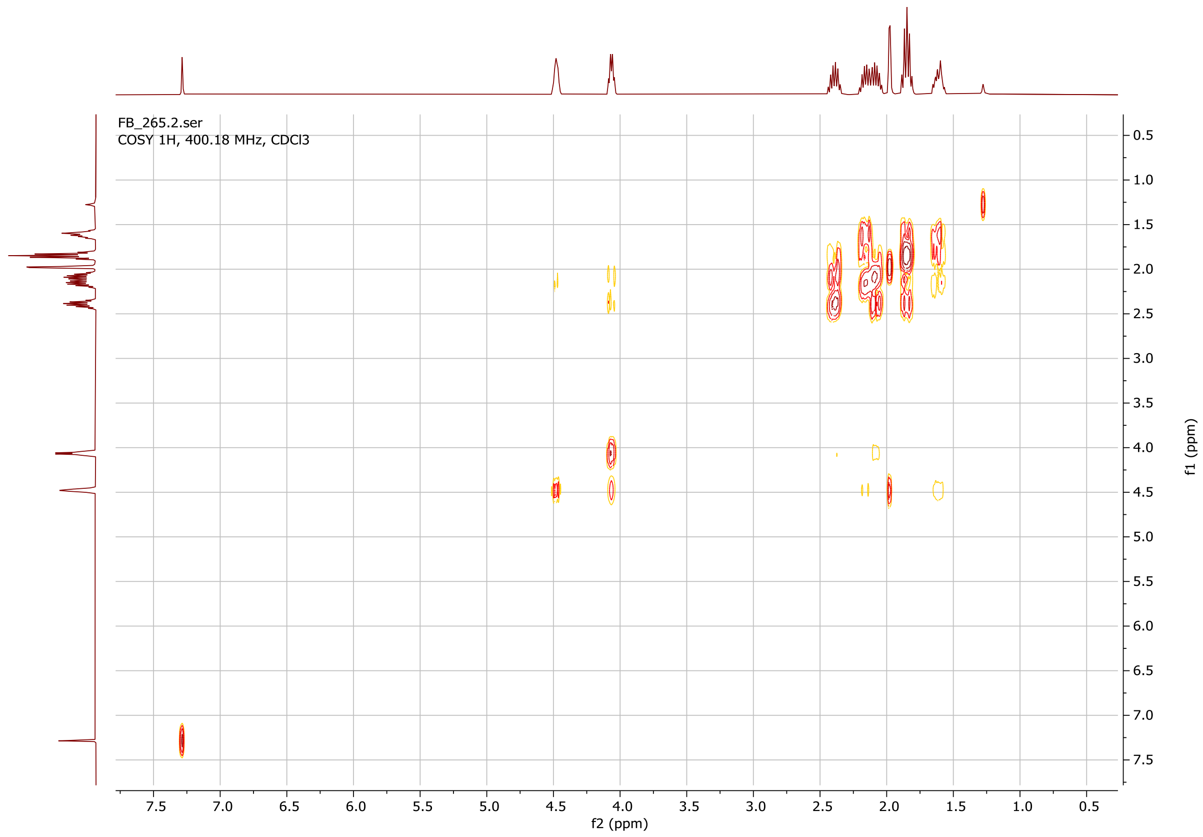
FB_265.2.ser
COSY 1H, 400.18 MHz, CDCl3
f2 (ppm)
f1 (ppm)

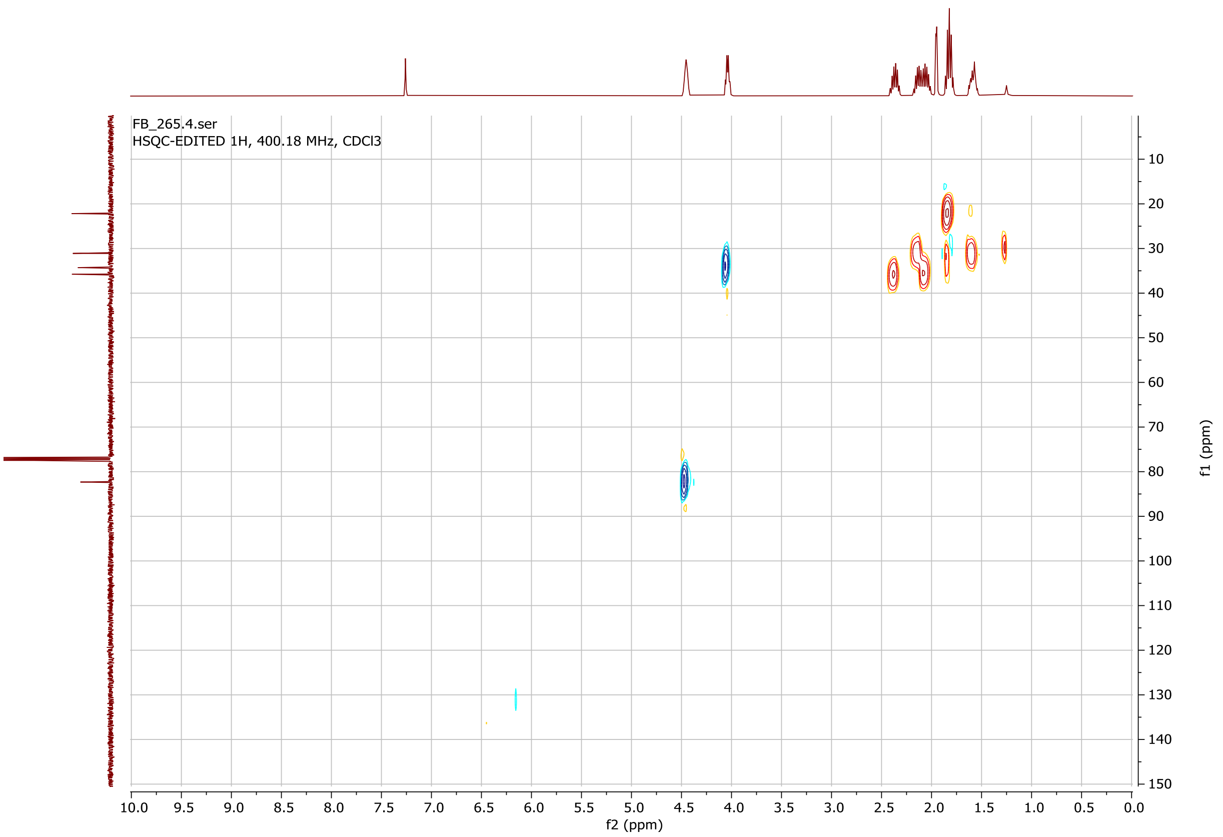

FB_265.4.ser
HSQC-EDITED 1H, 400.18 MHz, CDCl3
f2 (ppm)
f1 (ppm)

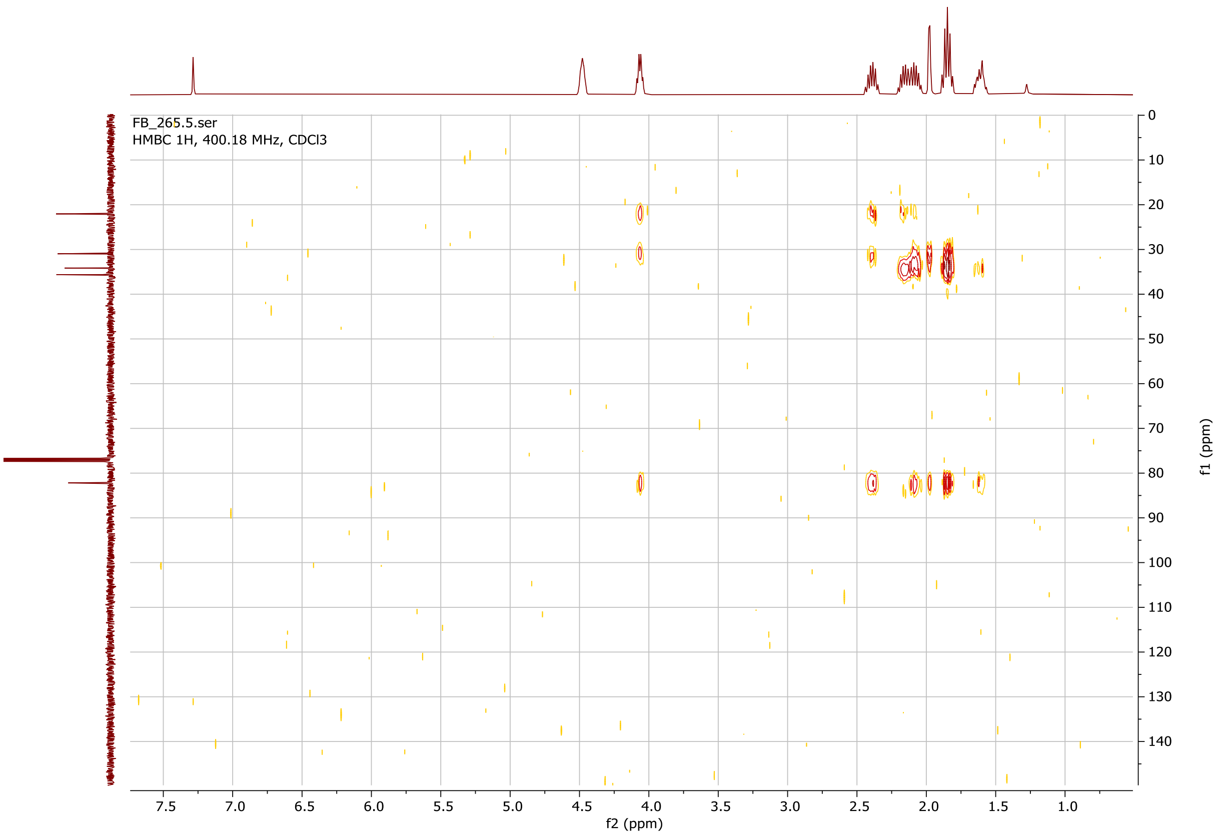

FB_265.5.ser
HMBC 1H, 400.18 MHz, CDCl3
f2 (ppm)
f1 (ppm)

2-Iodocyclohexanol **3c**

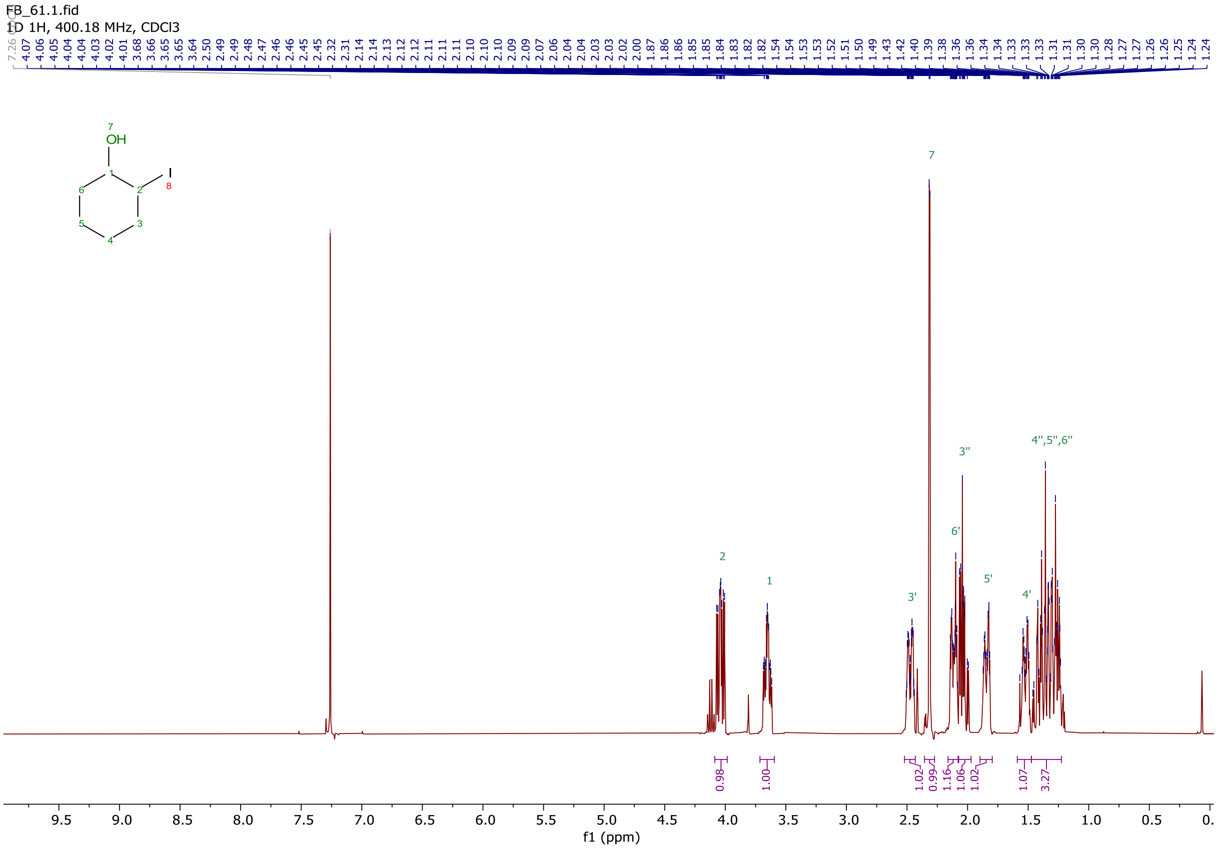

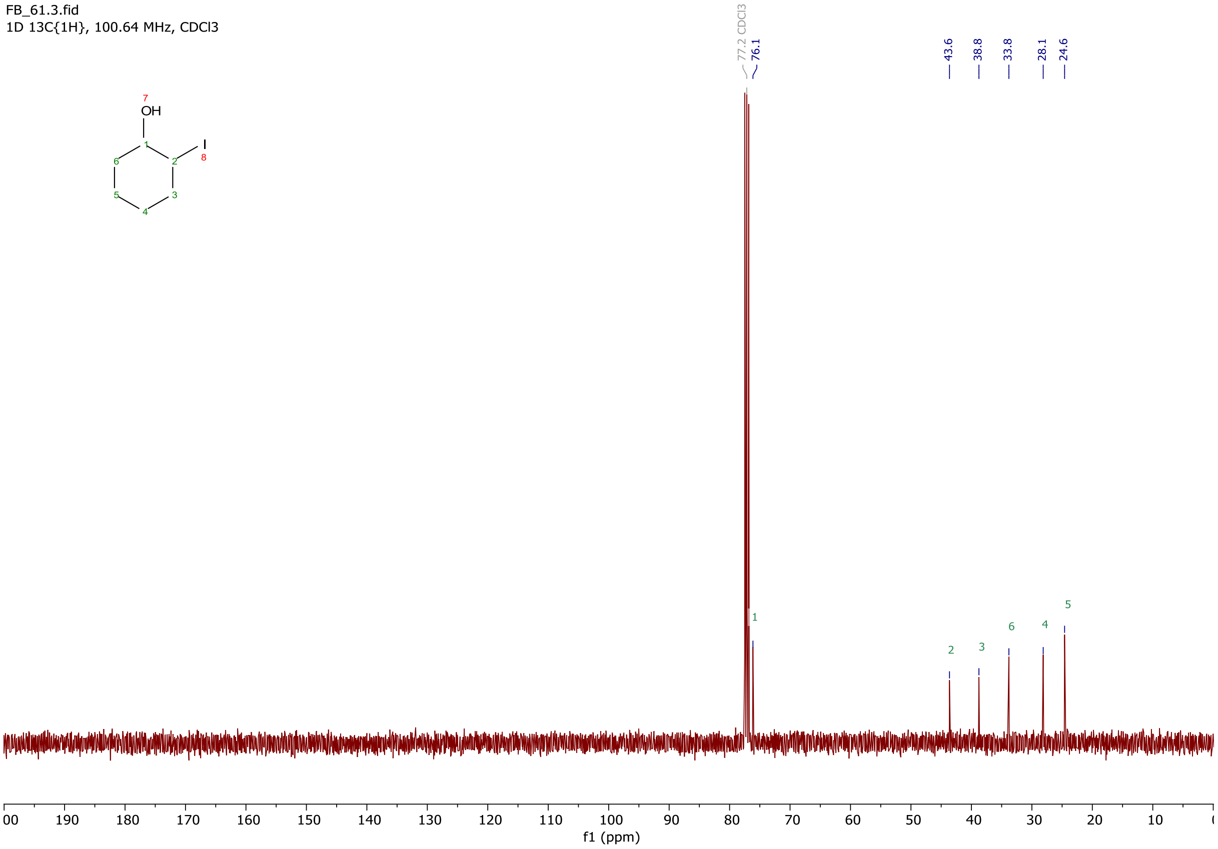

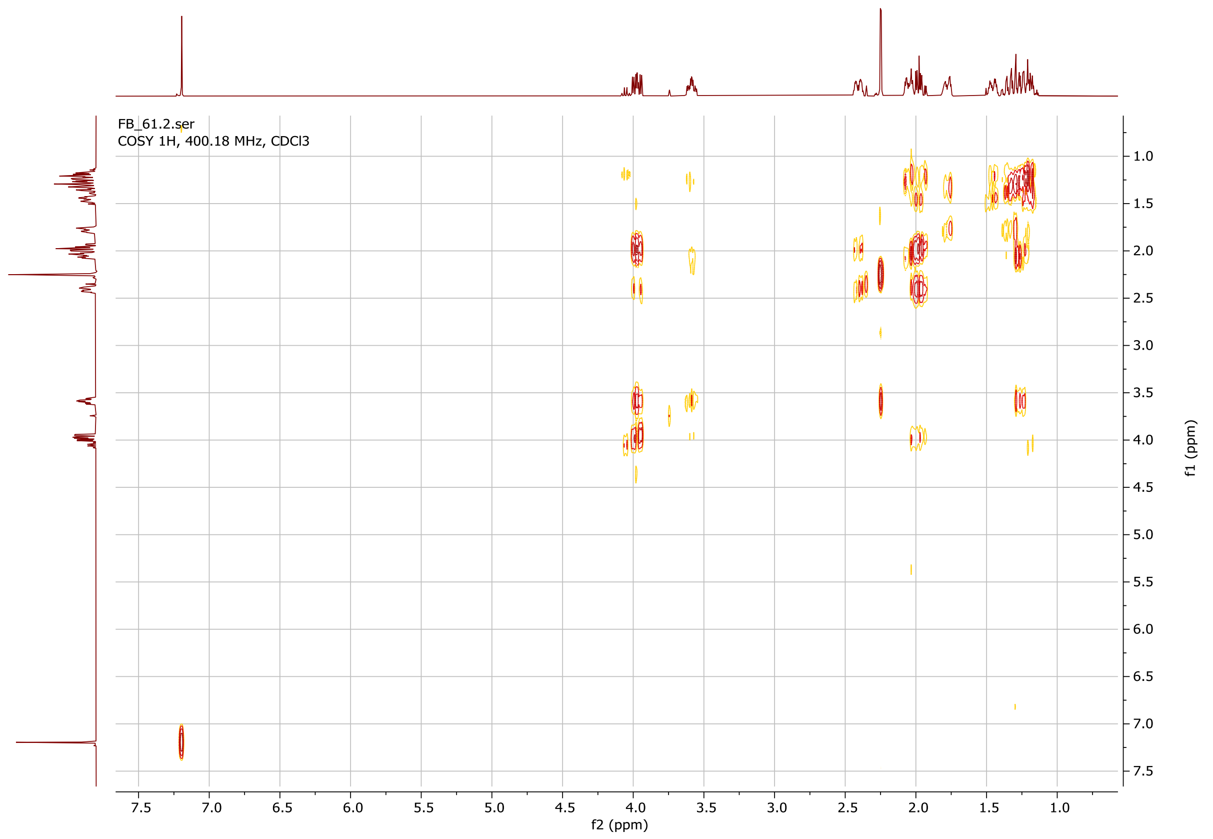

FB_61.2.ser
COSY 1H, 400.18 MHz, CDCl3
f2 (ppm)
f1 (ppm)

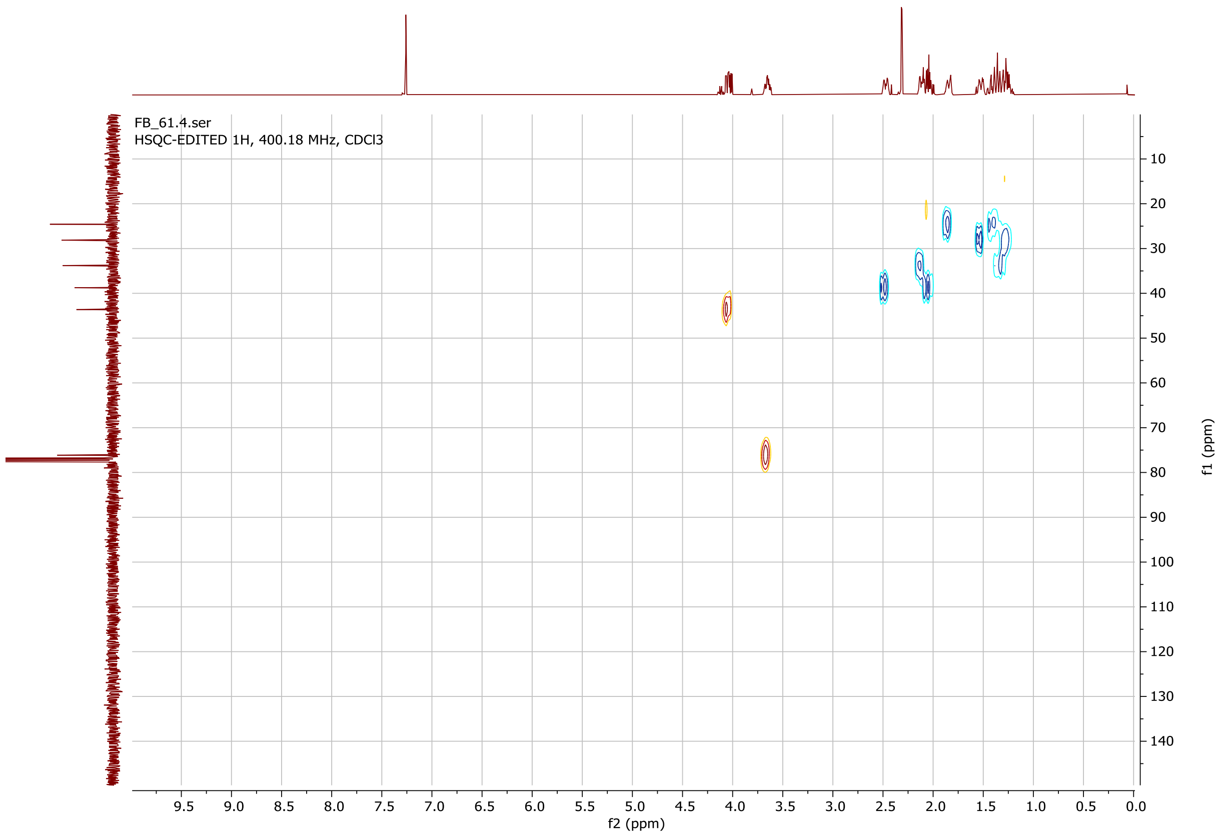

FB_61.4.ser
HSQC-EDITED 1H, 400.18 MHz, CDCl3
f2 (ppm)
f1 (ppm)

tert-Butyl 3-hydroxy-4-iodopyrrolidine-1-carboxylate **3d** *(mixture of rotamers)*

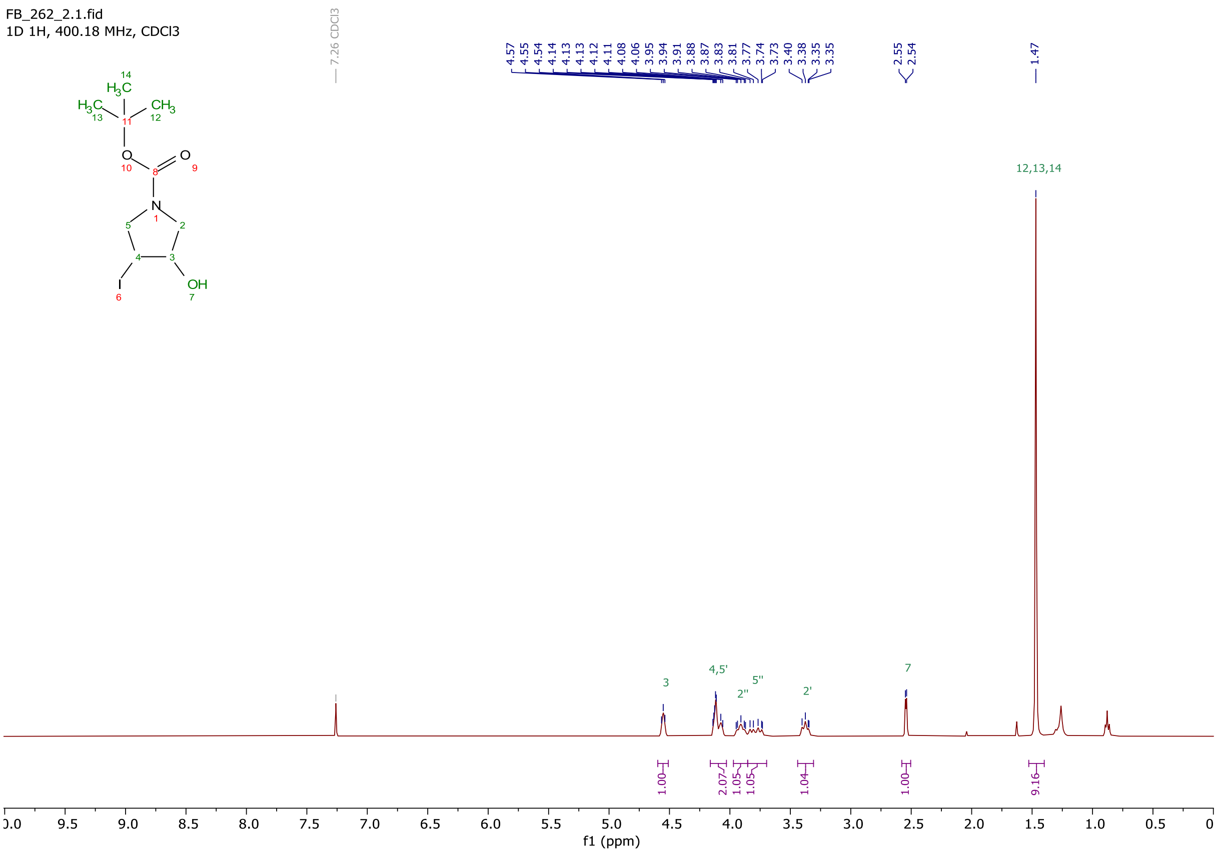

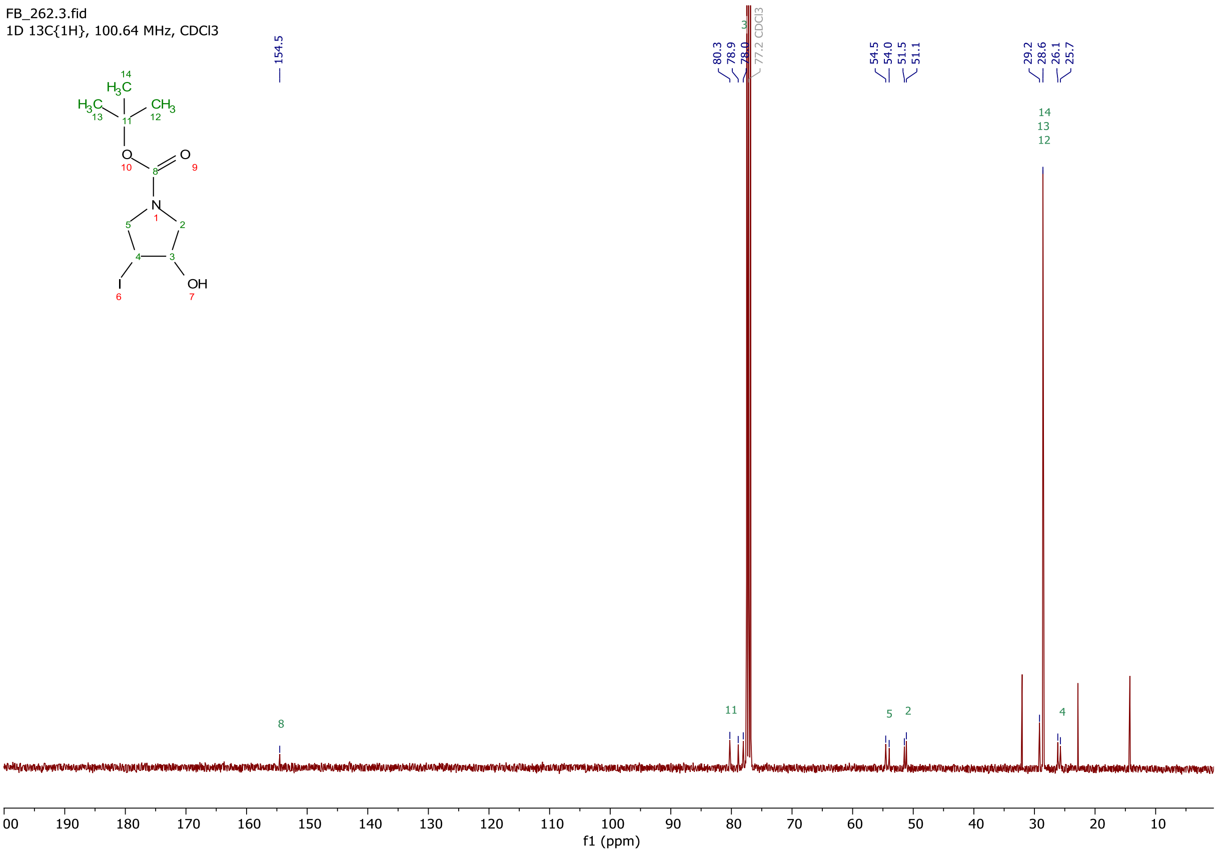

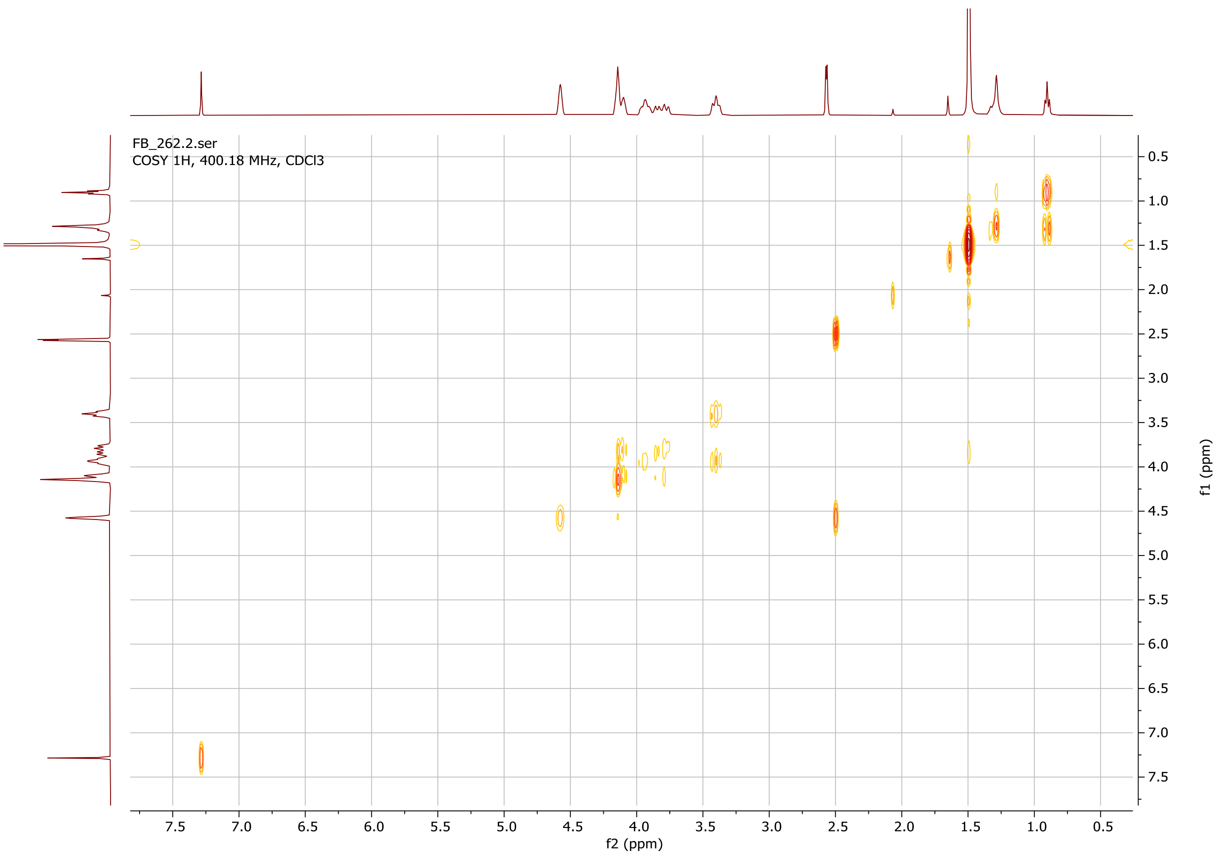
FB_262.2.ser
COSY 1H, 400.18 MHz, CDCl3
f2 (ppm)
f1 (ppm)

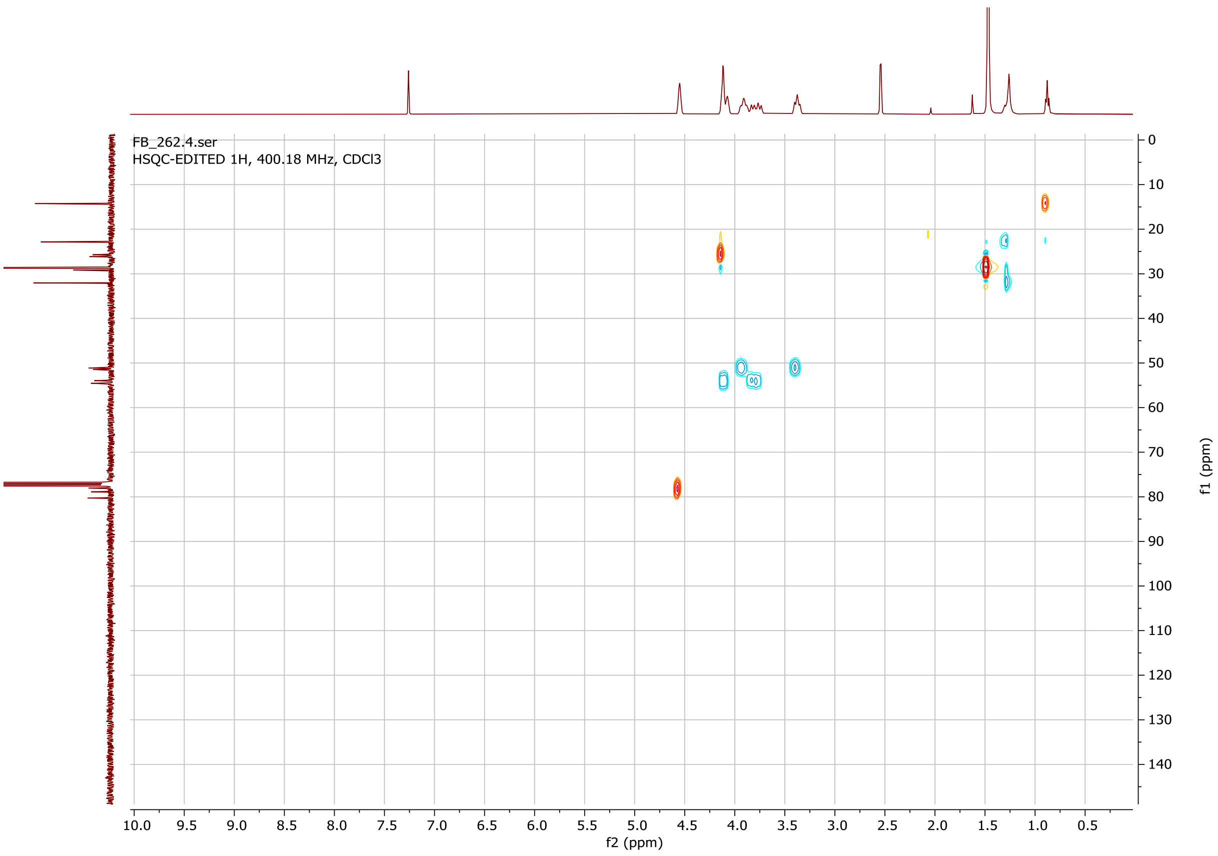
FB_262.4.ser
HSQC-EDITED 1H, 400.18 MHz, CDCl3
f2 (ppm)
f1 (ppm)

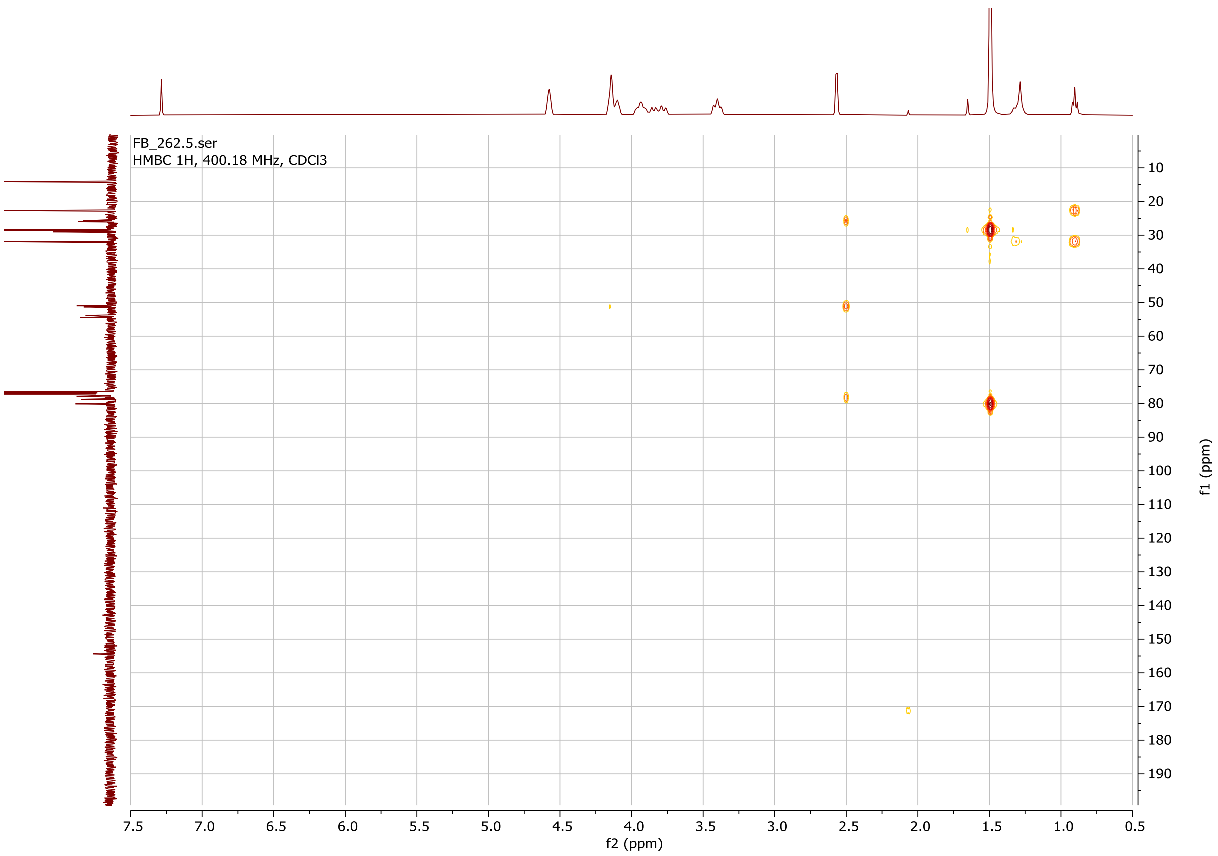

4-(2-(Benzyloxy)propan-2-yl)-2-iodo-1-methylcyclohexanol **3e**

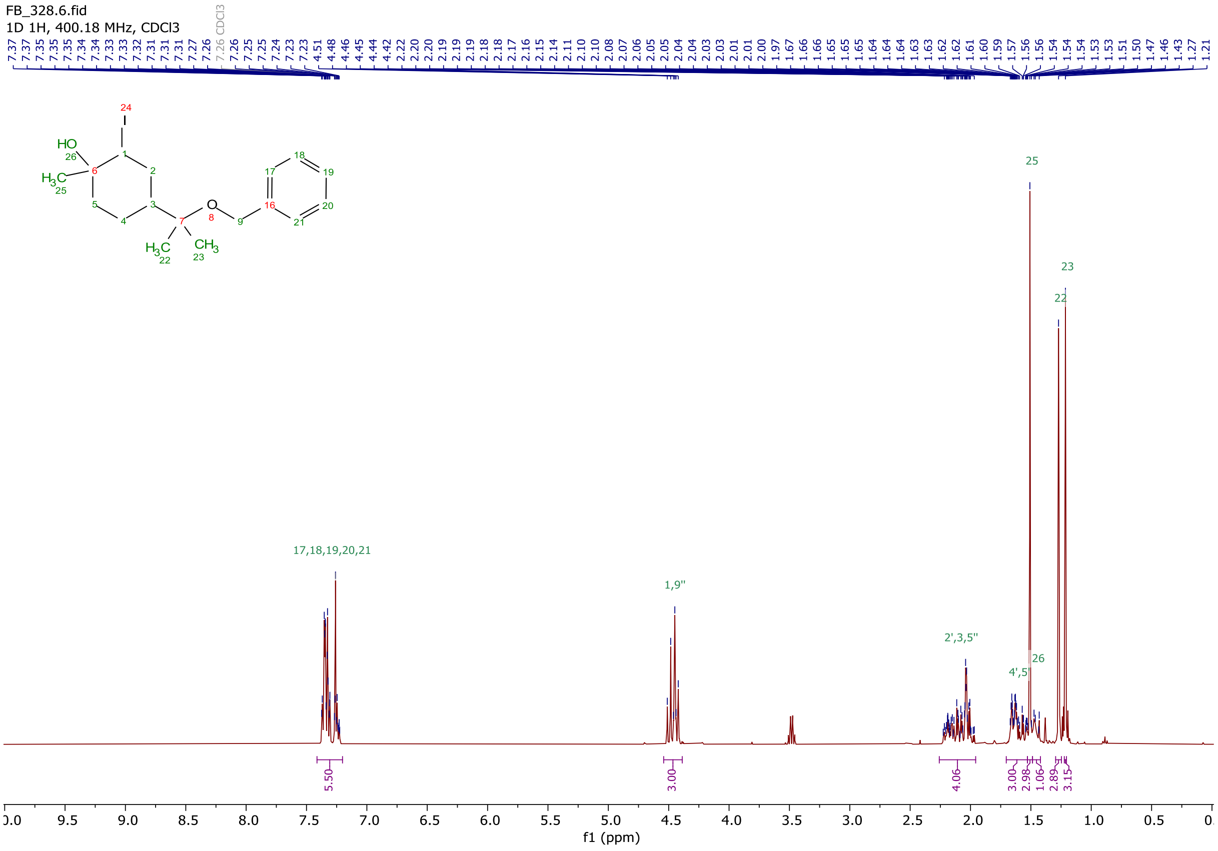

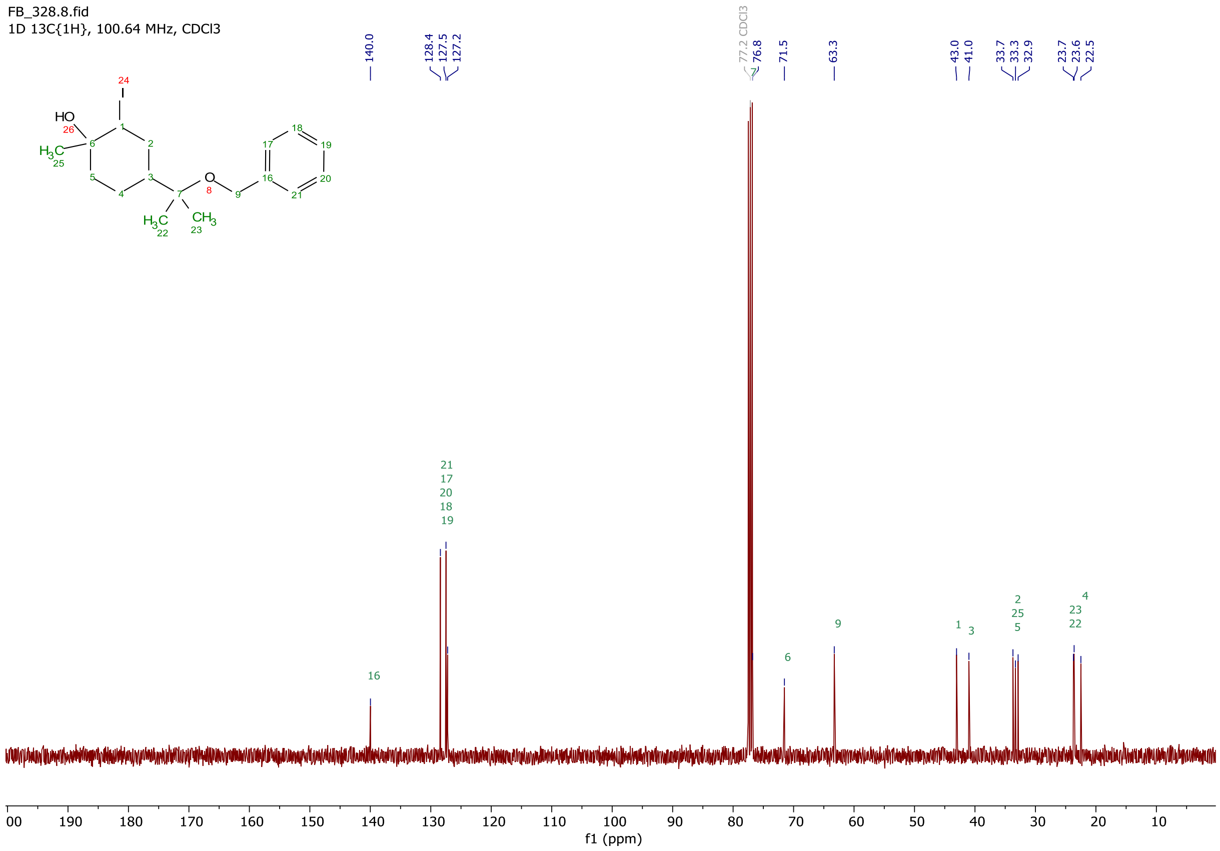
FB_328.8.fid
1D 13C{1H}, 100.64 MHz, CDCl3
140.0
128.4
127.5
127.2
77.2 CDCl3
76.8
71.5
63.3
43.0
41.0
33.7
33.3
32.9
23.7
23.6
22.5
HO
f1 (ppm)

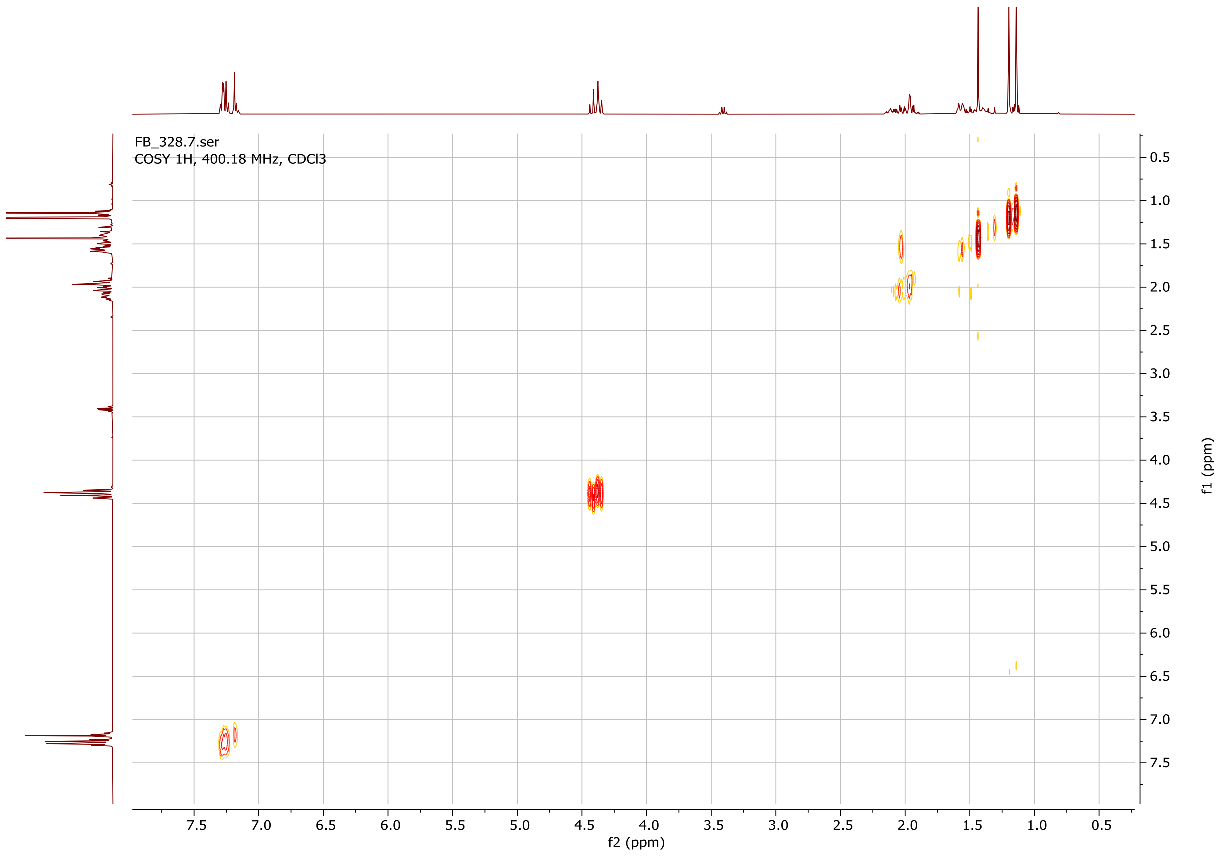
FB_328.7.ser
COSY 1H, 400.18 MHz, CDCl3
f2 (ppm)
f1 (ppm)

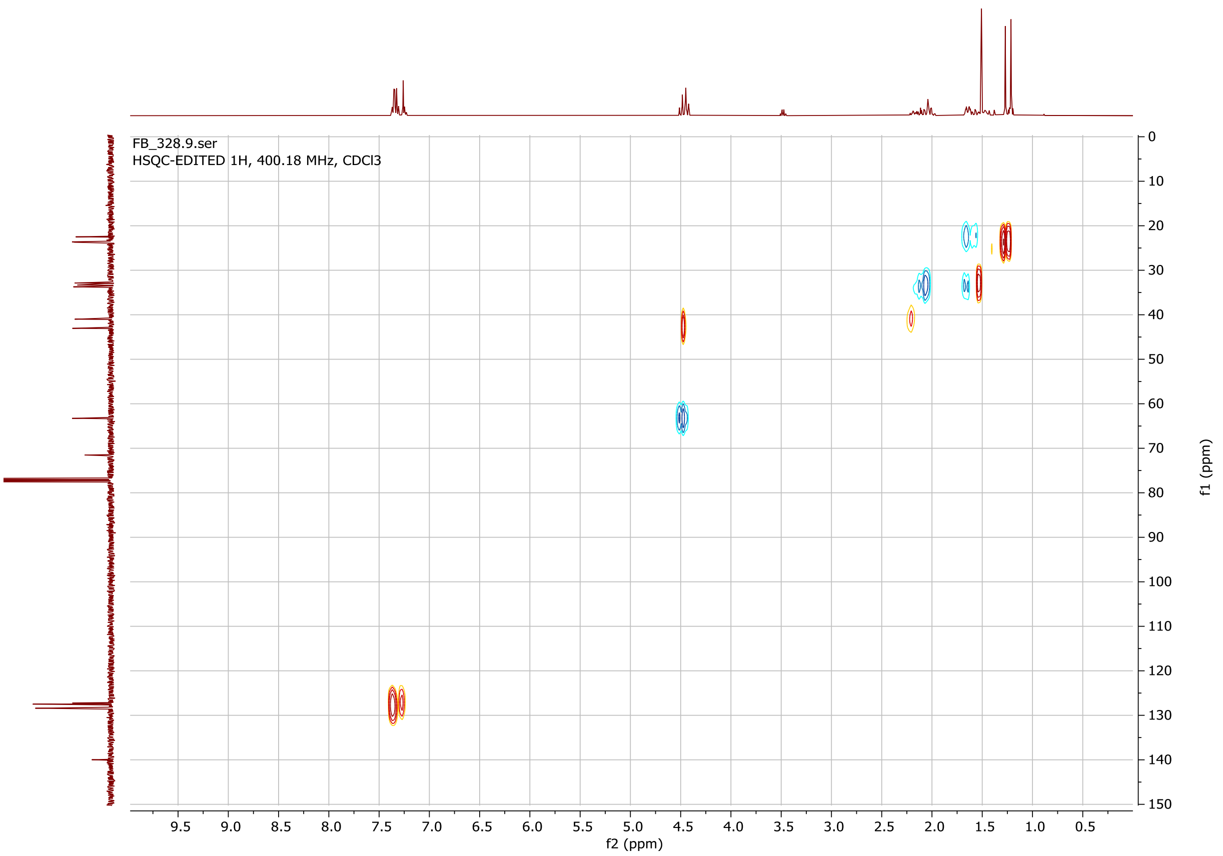
FB_328.9.ser
HSQC-EDITED 1H, 400.18 MHz, CDCl3
f2 (ppm)
f1 (ppm)

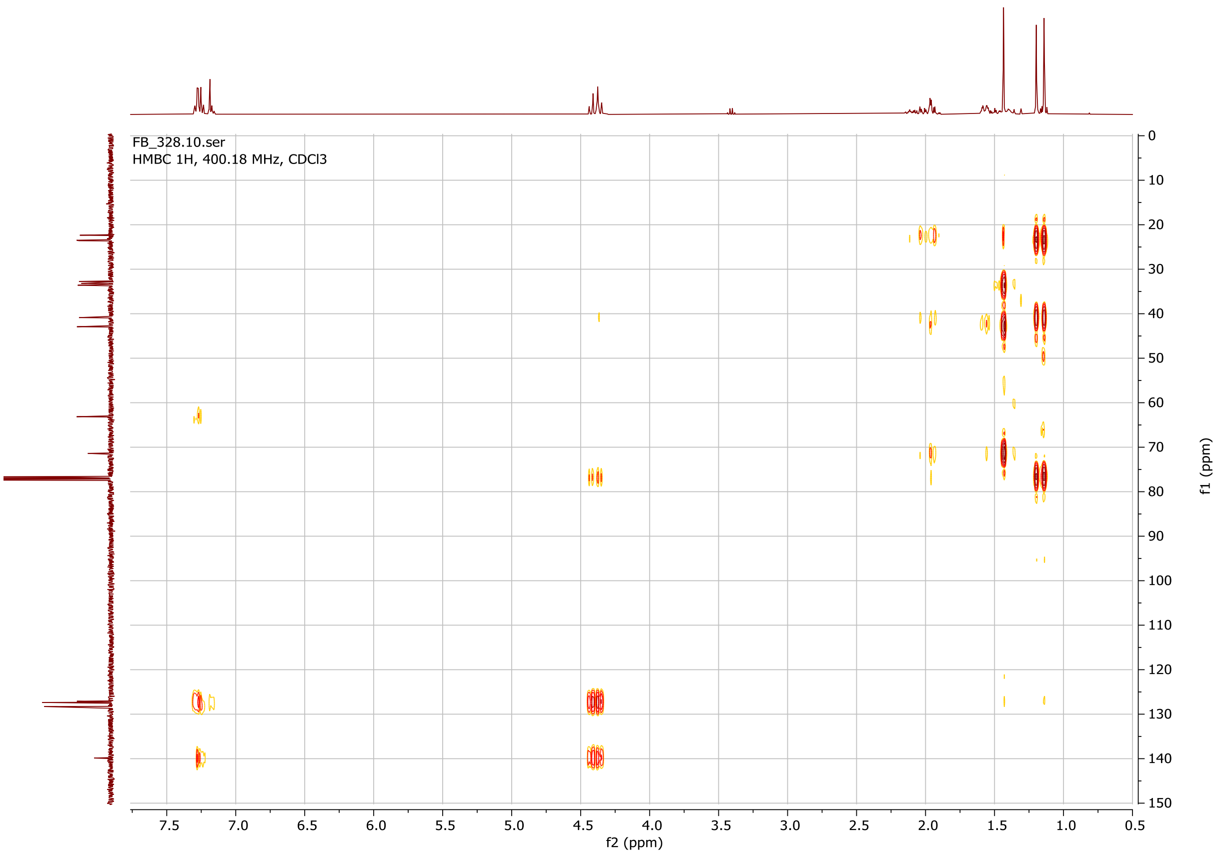
FB_328.10.ser
HMBC 1H, 400.18 MHz, CDCl3
f2 (ppm)
f1 (ppm)

2-Iodo-1-phenylpropan-1-ol **3f**

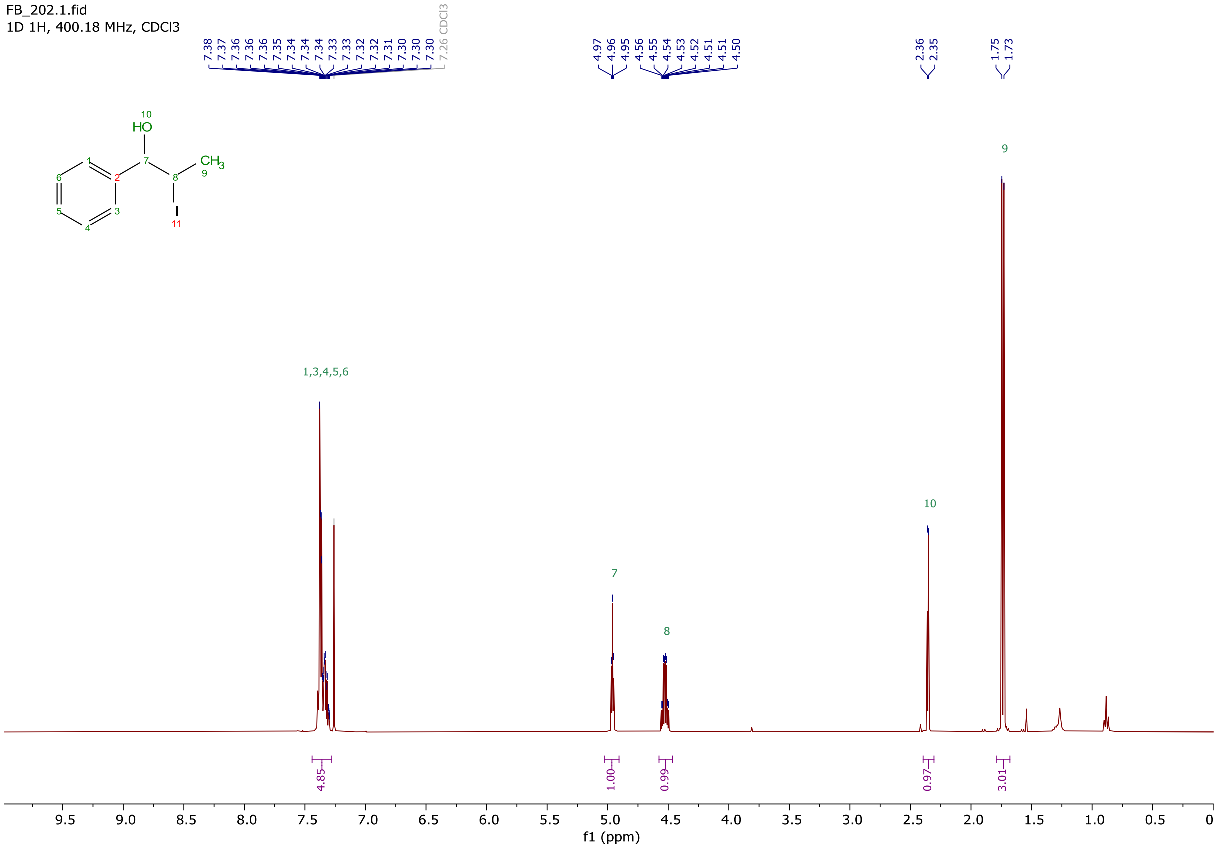

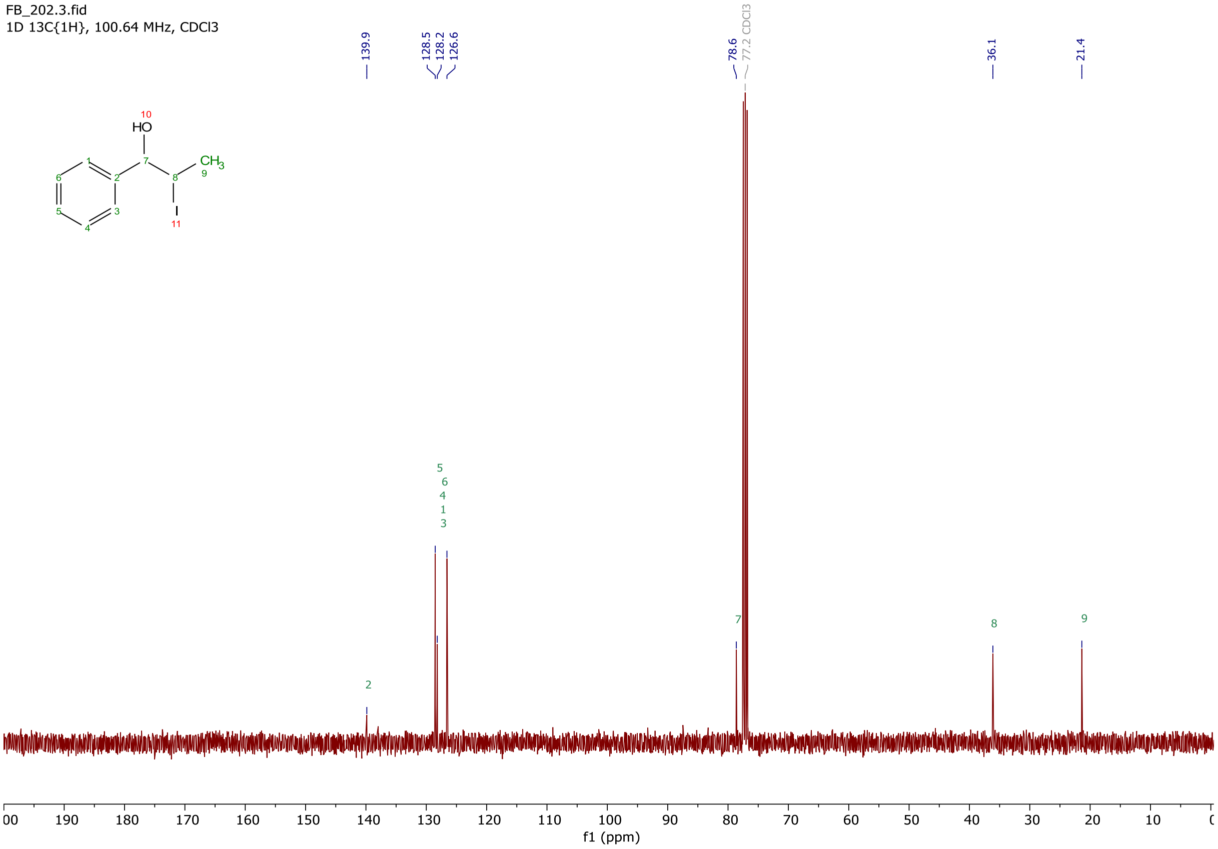

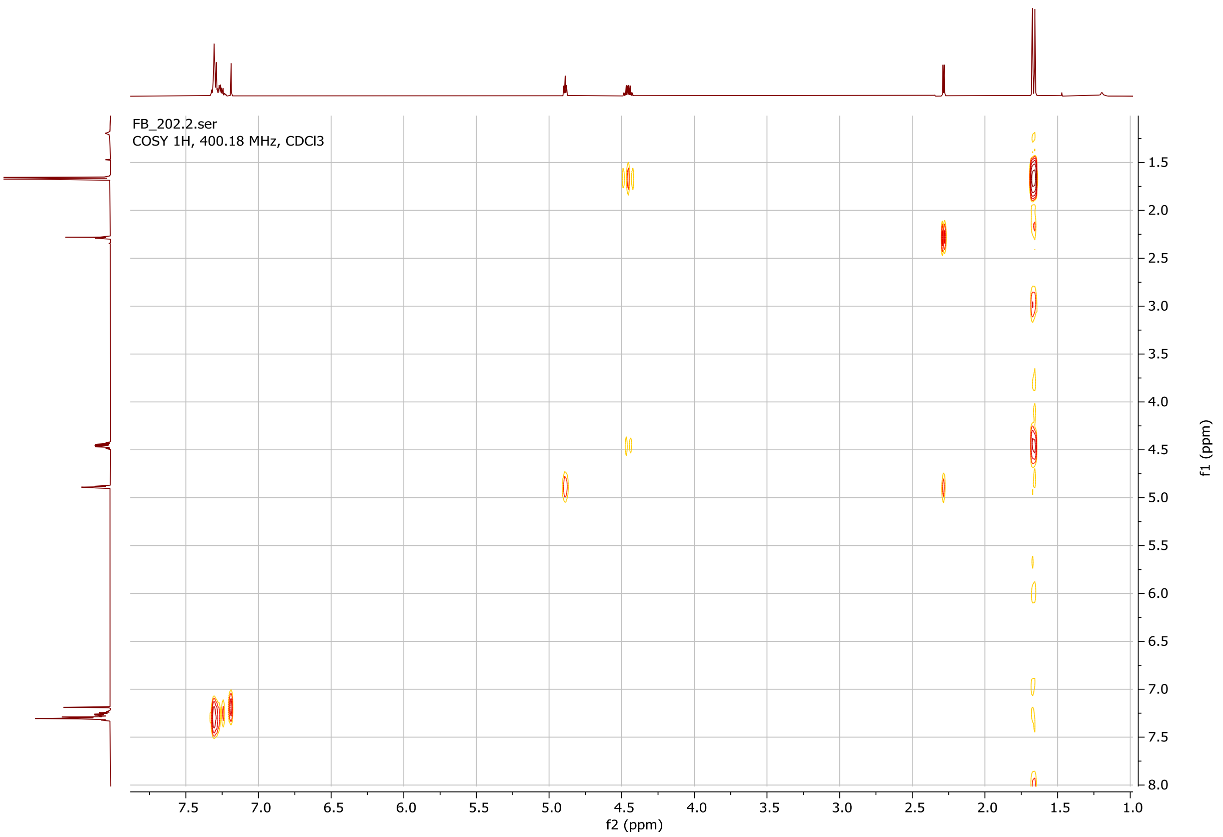
FB_202.2.ser
COSY 1H, 400.18 MHz, CDCl3
f2 (ppm)
f1 (ppm)

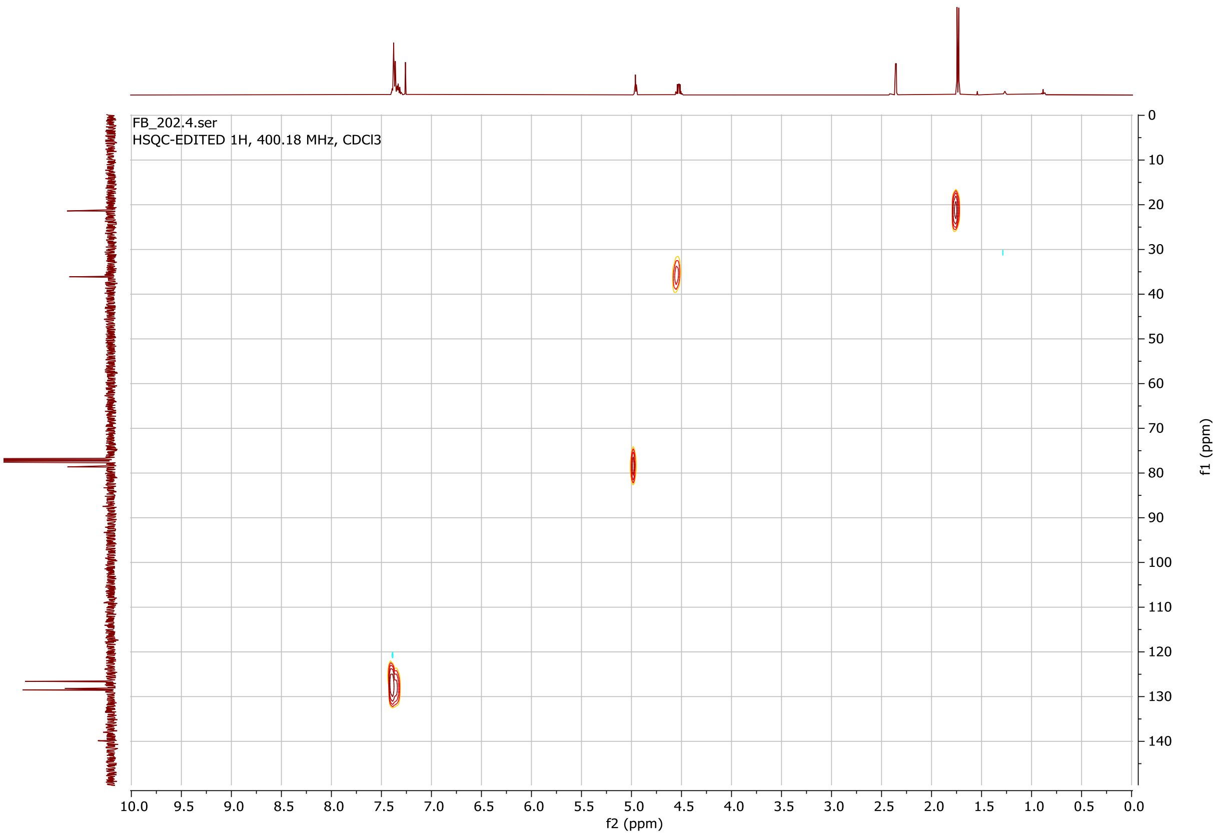
FB_202.4.ser
HSQC-EDITED 1H, 400.18 MHz, CDCl3
f2 (ppm)
f1 (ppm)

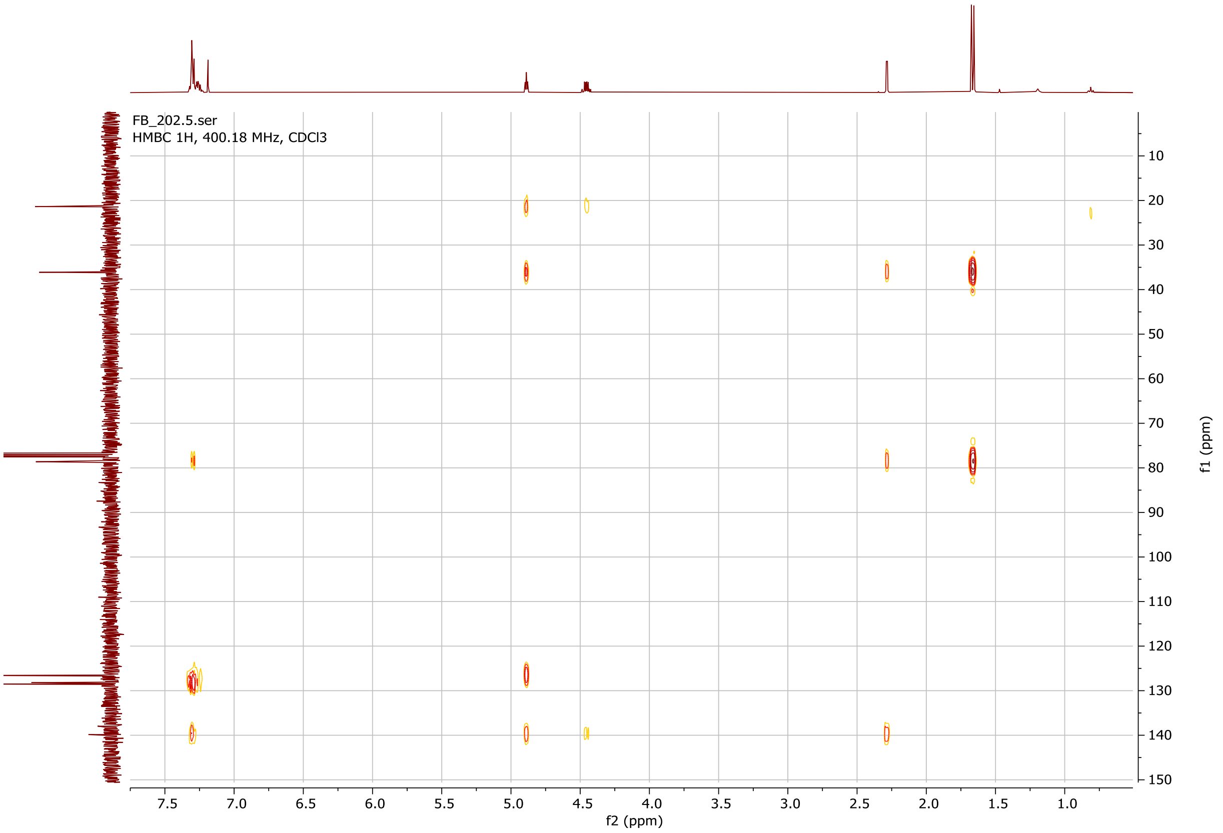

5-Iodooctan-4-ol **3g**

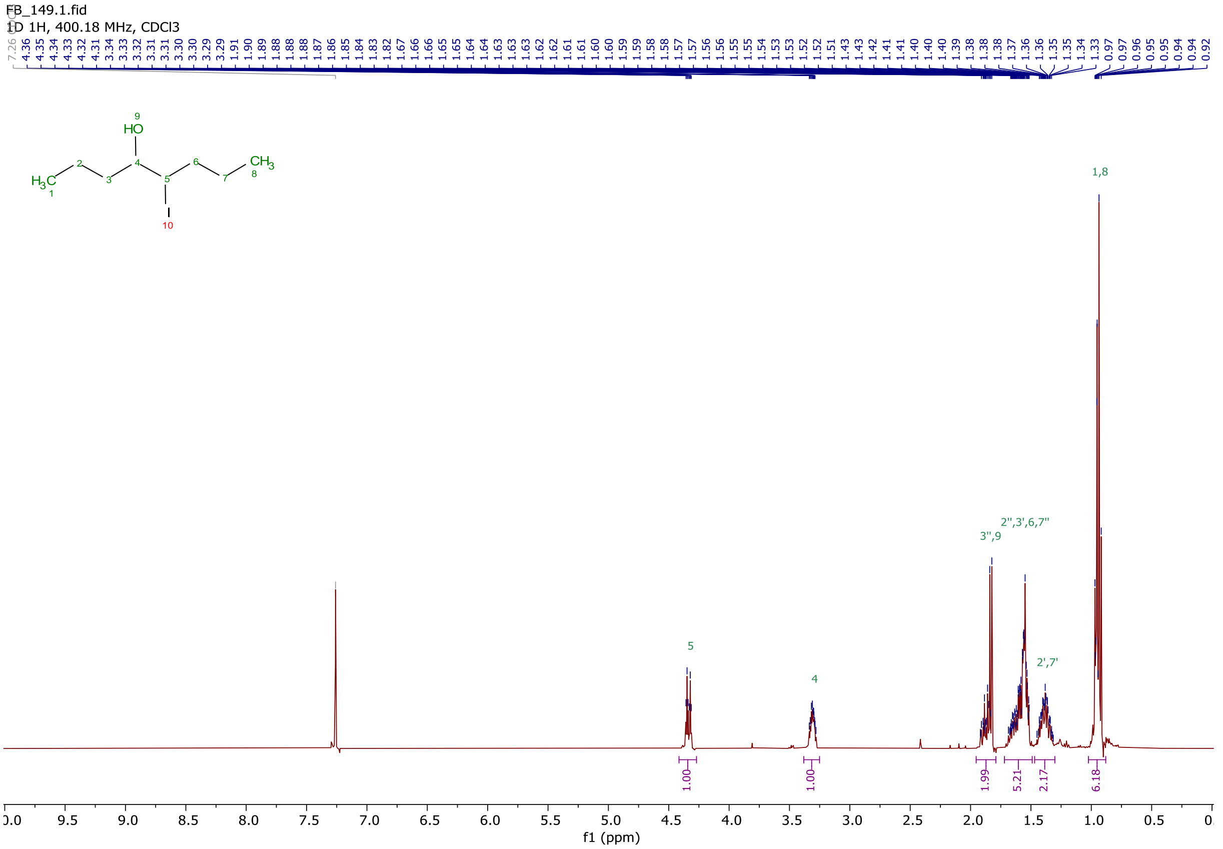

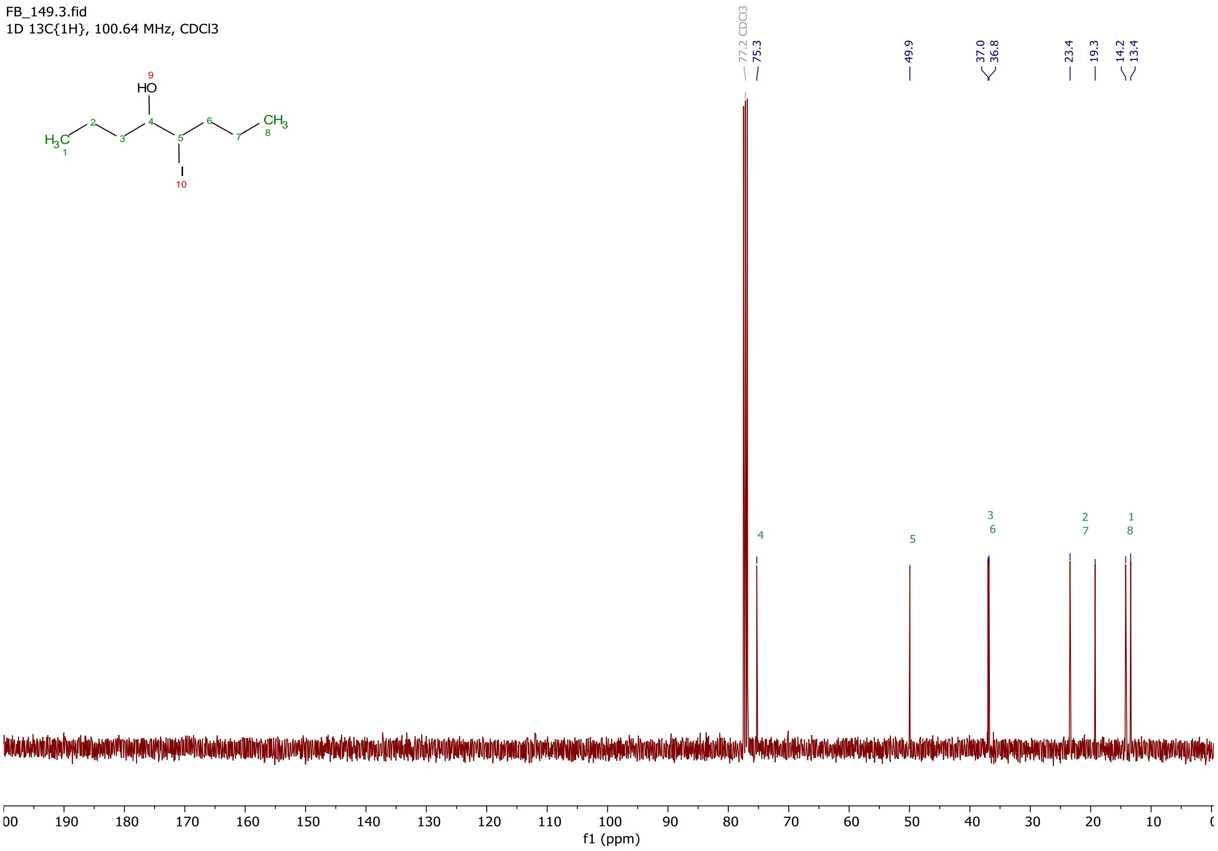
FB_149.3.fid
1D 13C{1H}, 100.64 MHz, CDCl3
HO
H_3C
CH_3
I
77.2 CDCl3
75.3
49.9
37.0
36.8
23.4
19.3
14.2
13.4
f1 (ppm)

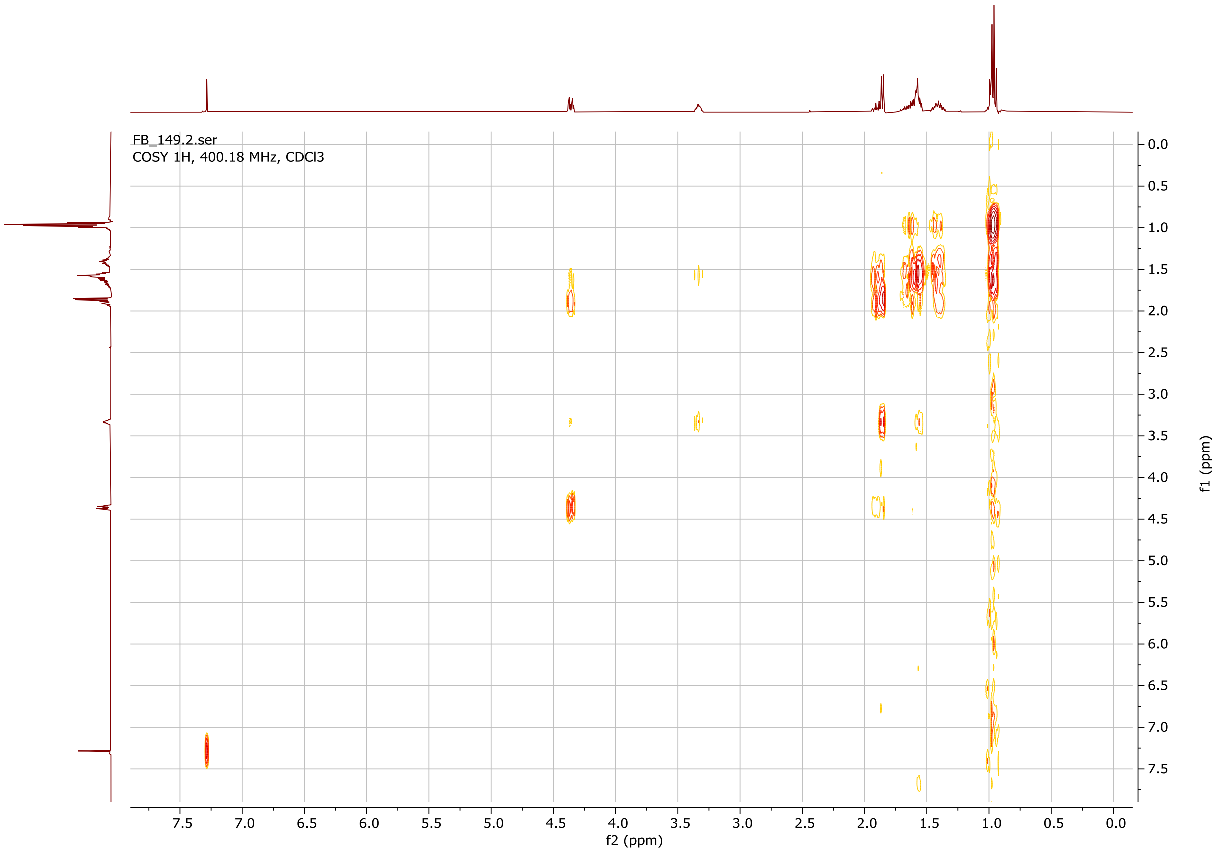
FB_149.2.ser
COSY 1H, 400.18 MHz, CDCl3
f2 (ppm)
f1 (ppm)

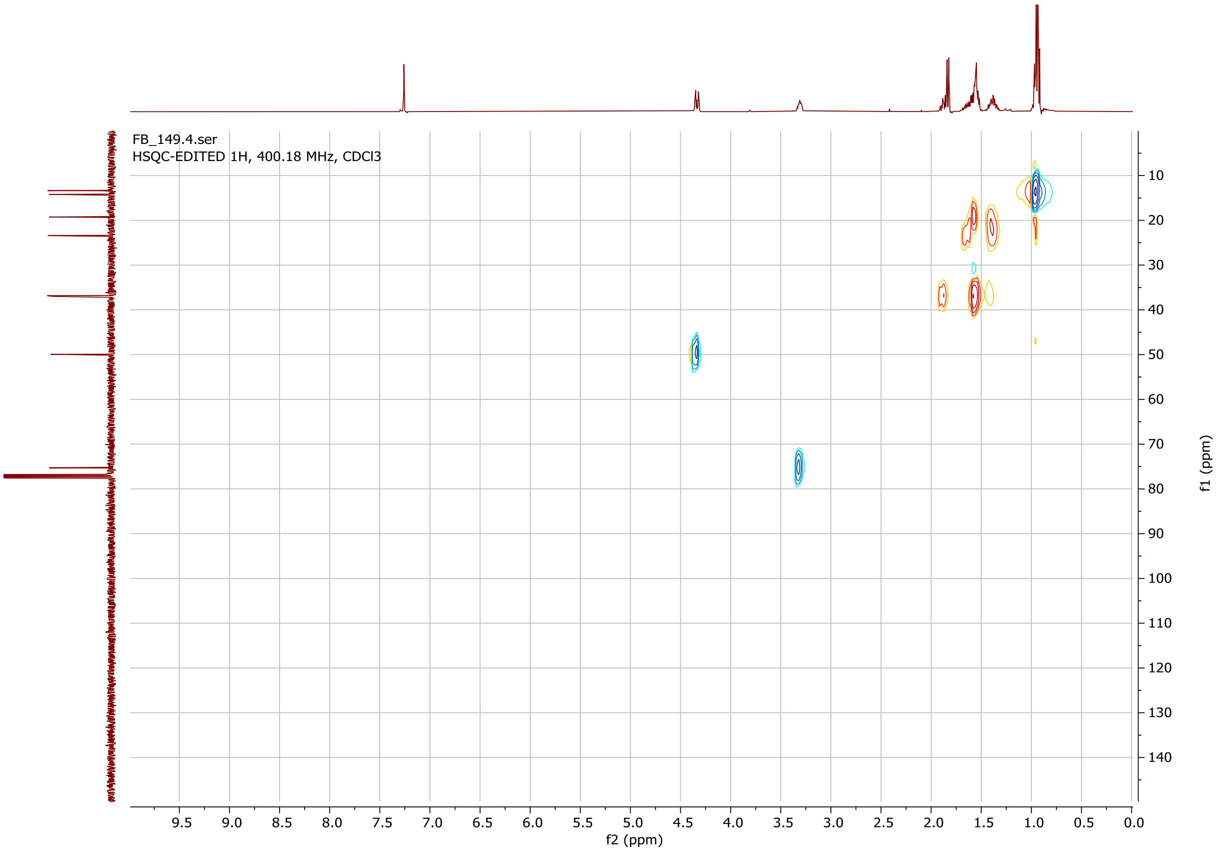

FB_149.4.ser
HSQC-EDITED 1H, 400.18 MHz, CDCl3
f2 (ppm)
f1 (ppm)

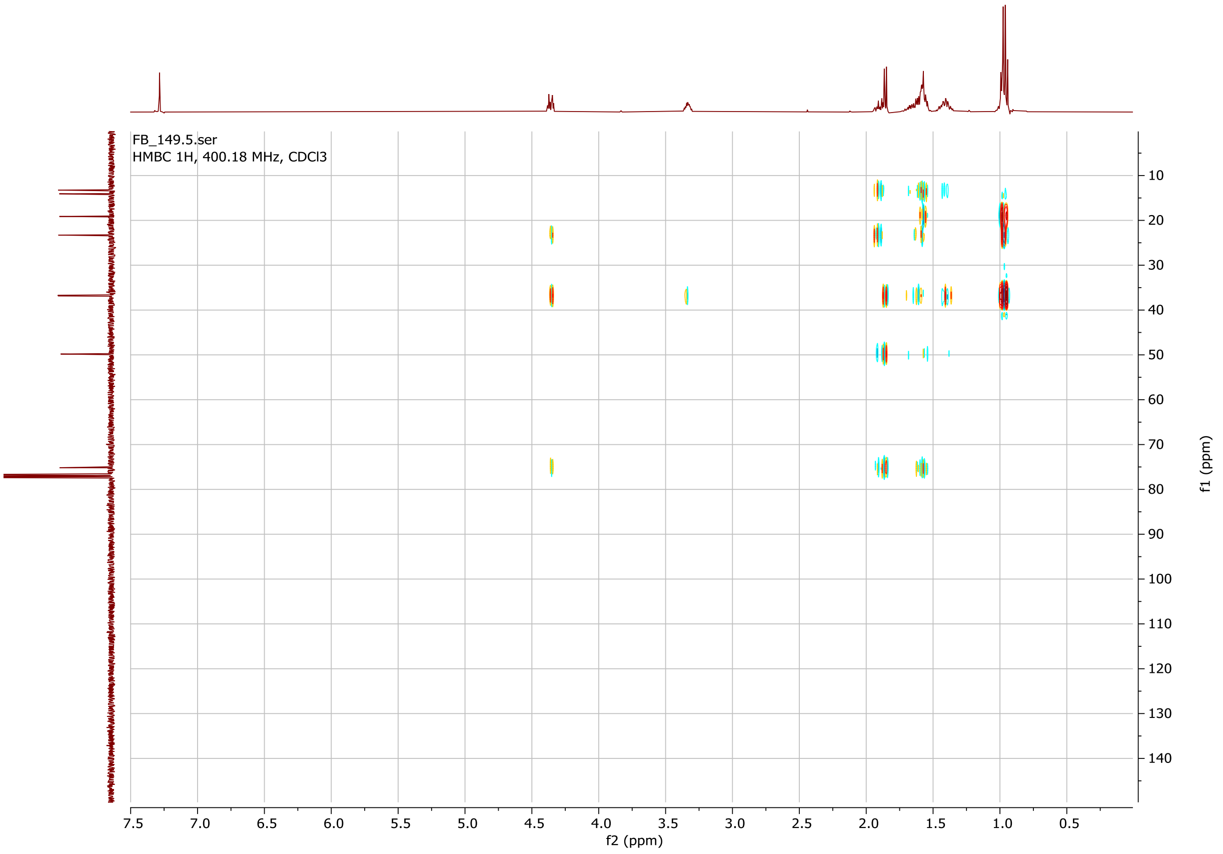

FB_149.5.ser
HMBC 1H, 400.18 MHz, CDCl3
f2 (ppm)
f1 (ppm)

2-Iodo-1-phenyl-propan-1,3-diol **3h**

FB_287_dry.1.fid
1D 1H, 400.18 MHz, CDCl3

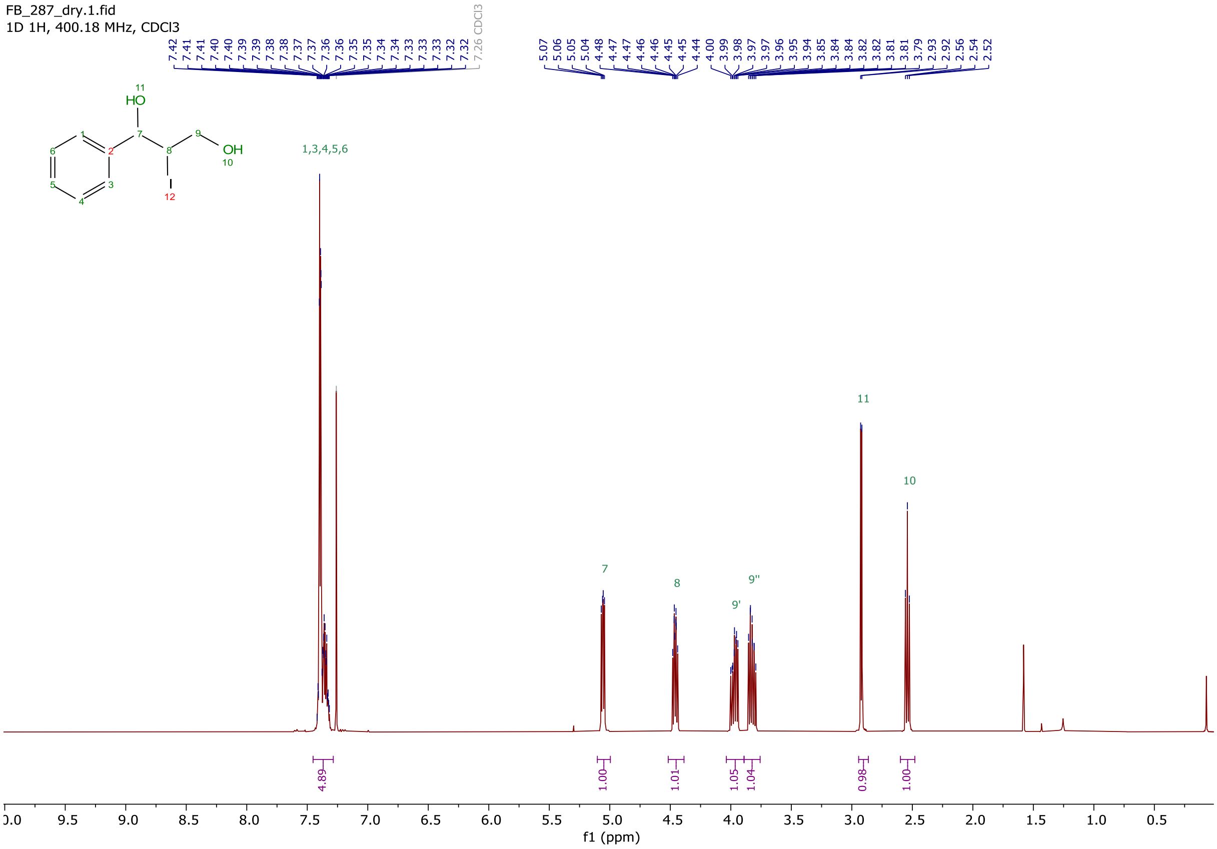

FB_287_dry.3.fid
1D 13C{1H}, 100.64 MHz, CDCl3

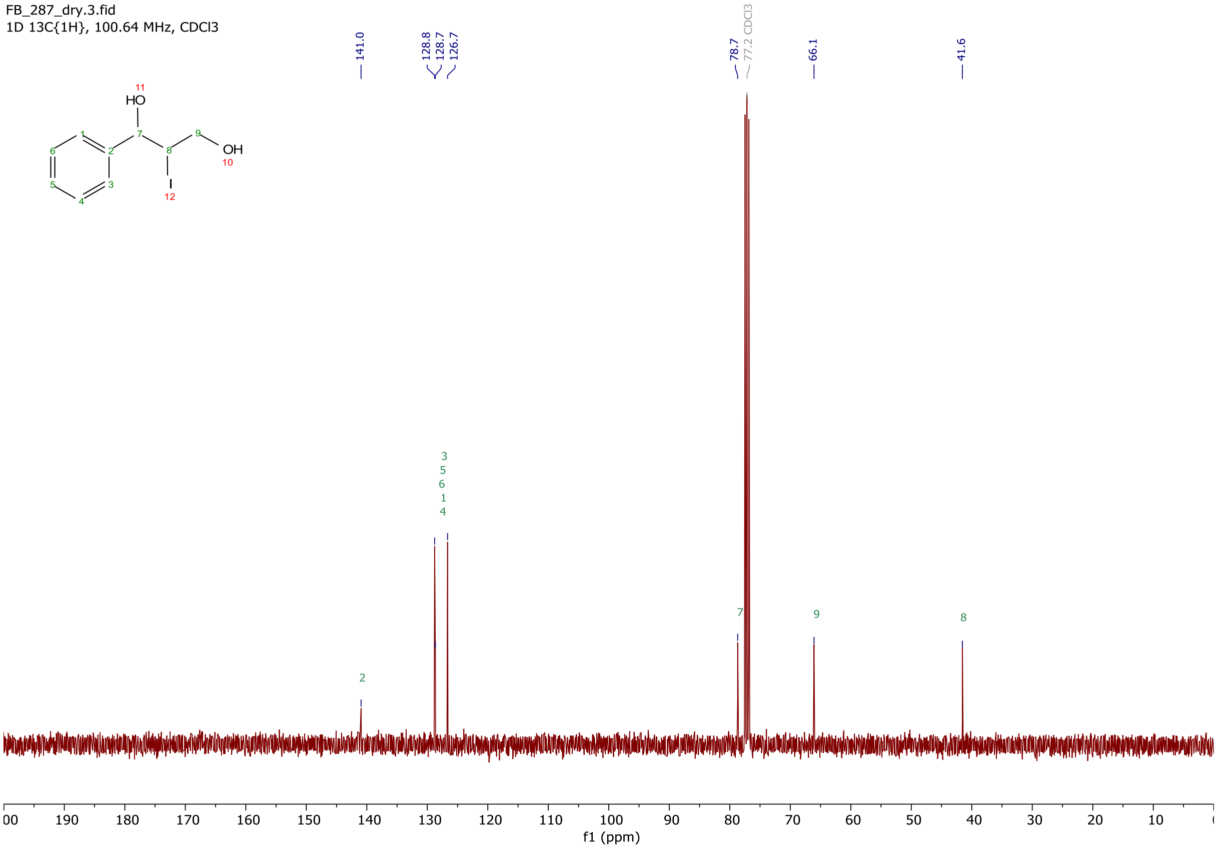

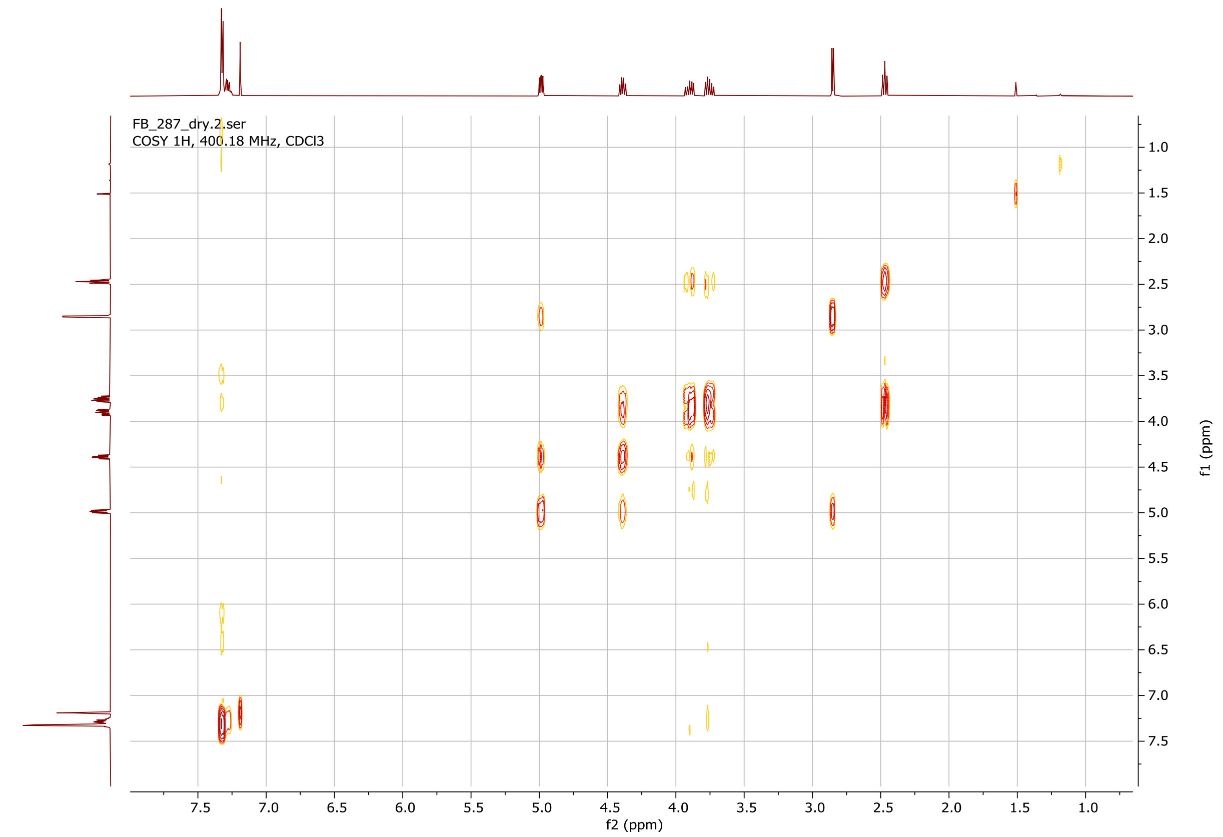
FB_287_dry.2.ser
COSY 1H, 400.18 MHz, CDCl3
f2 (ppm)
f1 (ppm)

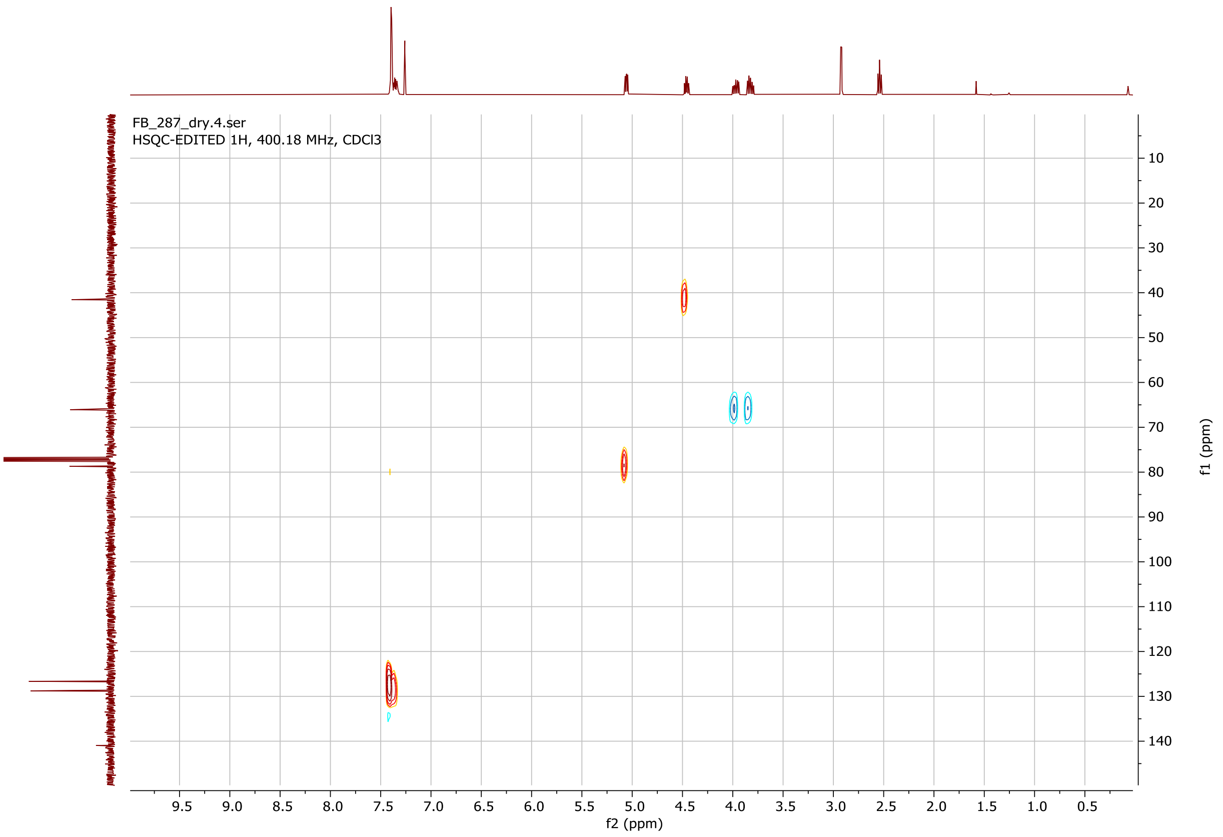
FB_287_dry.4.ser
HSQC-EDITED 1H, 400.18 MHz, CDCl3
f2 (ppm)
f1 (ppm)

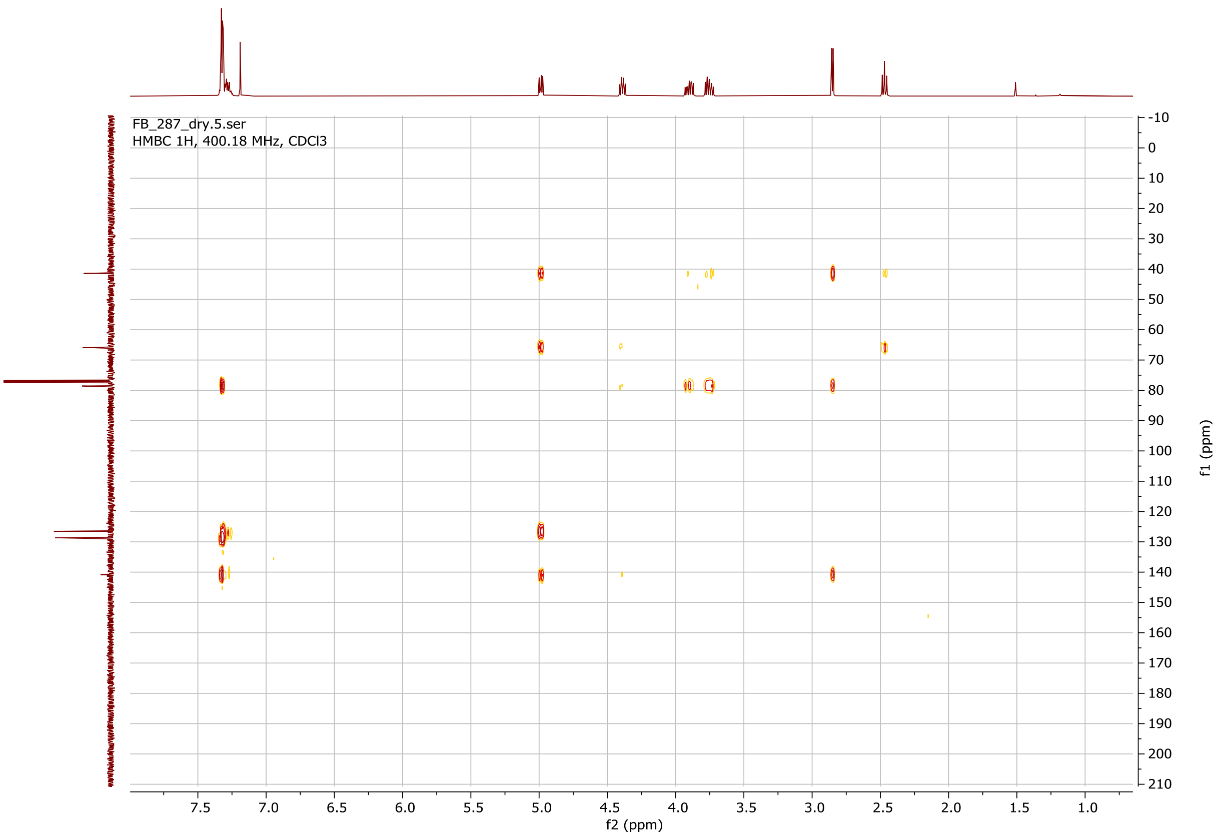

1-((*tert*-Butyldimethylsilyl)oxy)-2-iodohexan-3-ol **3i**

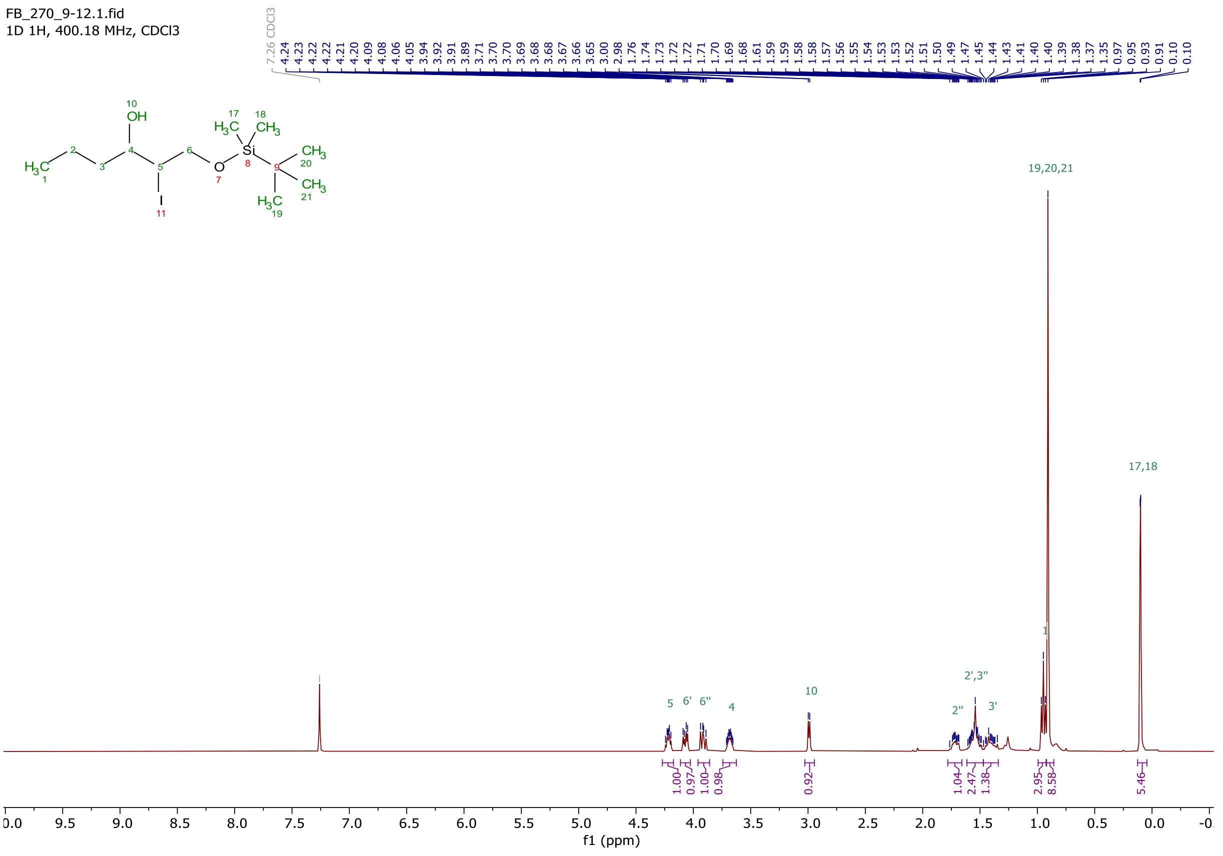

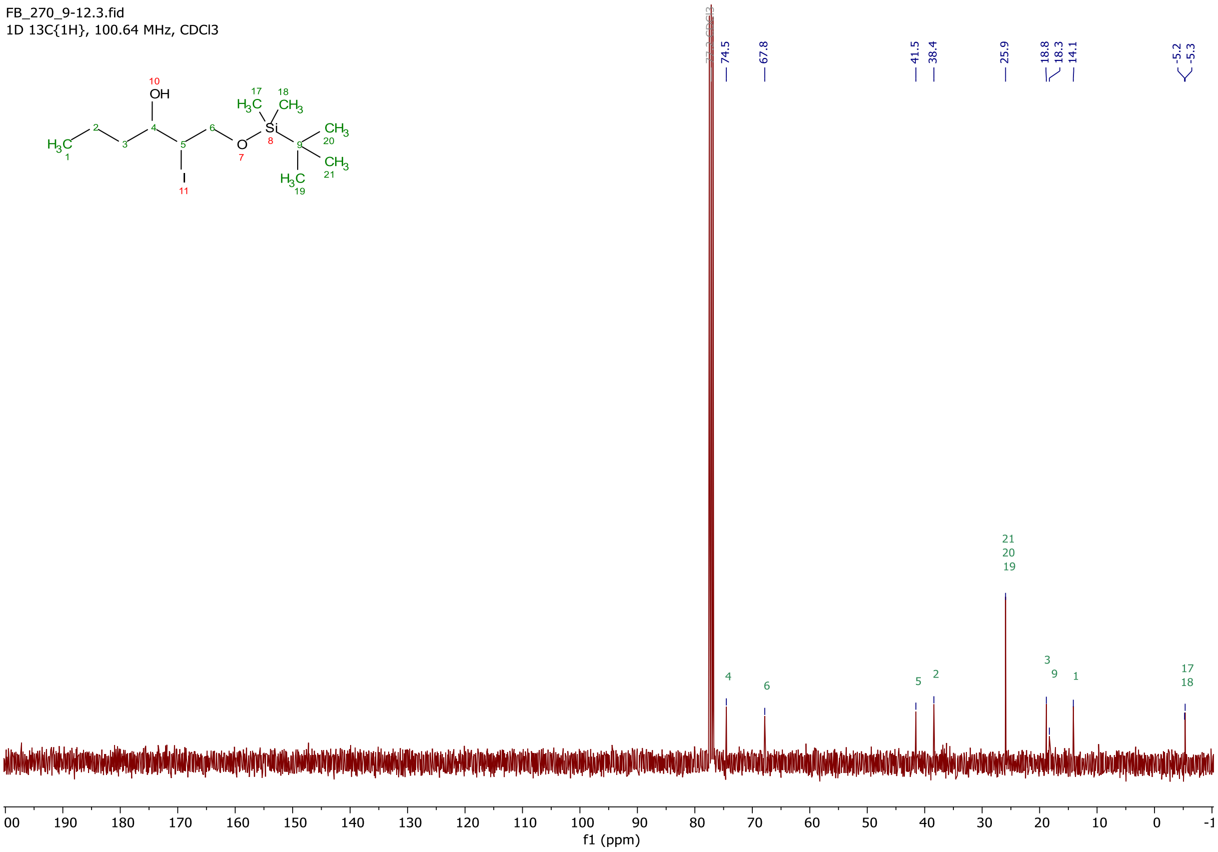

FB_270_9-12.3.fid
1D 13C{1H}, 100.64 MHz, CDCl3
74.5
67.8
41.5
38.4
25.9
18.8
18.3
14.1
-5.2
-5.3
f1 (ppm)

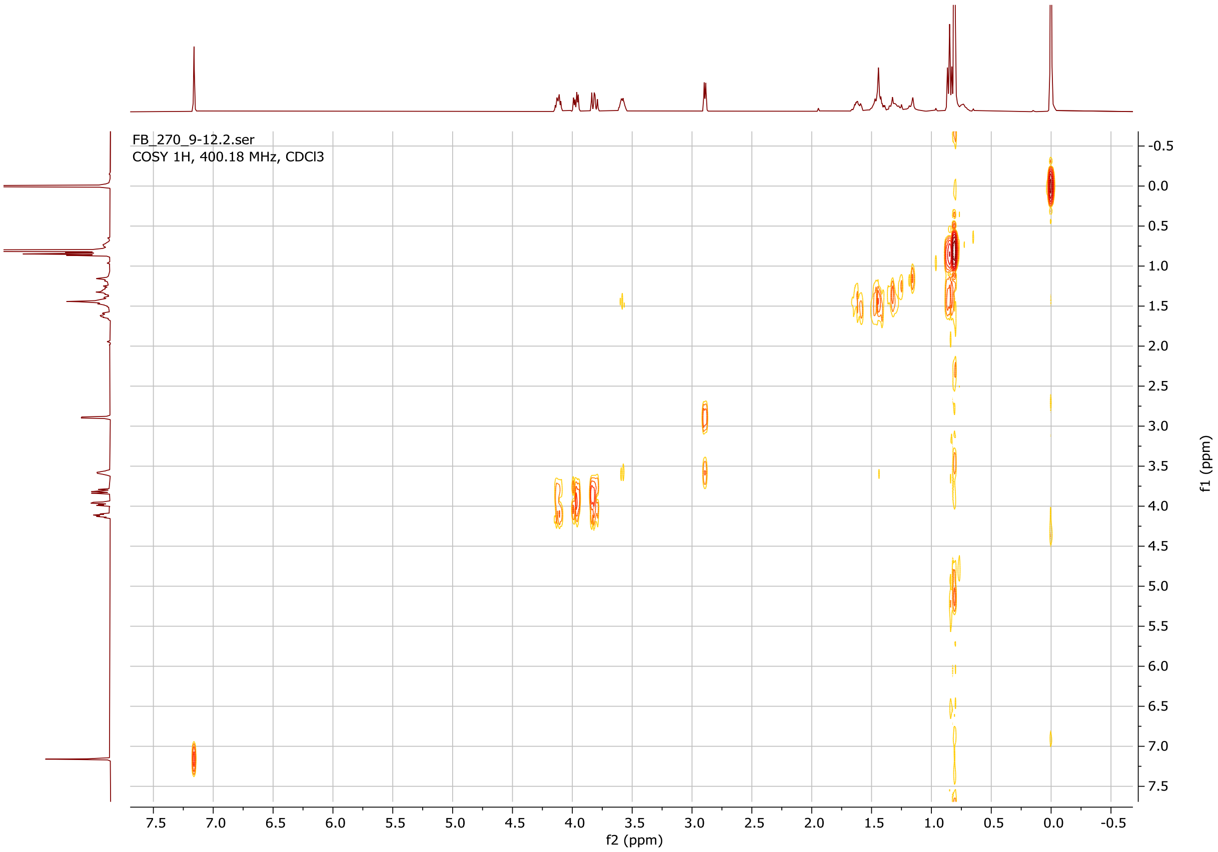

FB_270_9-12.2.ser
COSY 1H, 400.18 MHz, CDCl3
f2 (ppm)
f1 (ppm)

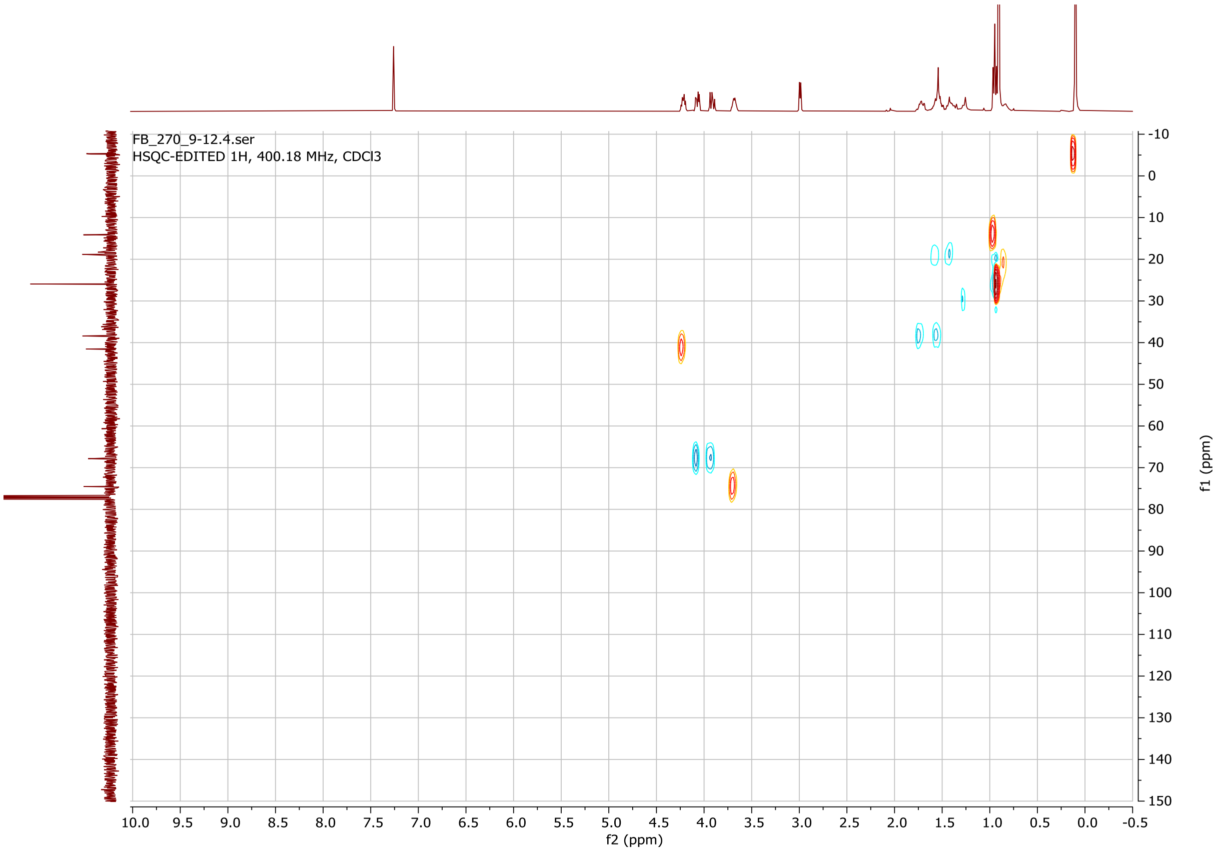
FB_270_9-12.4.ser
HSQC-EDITED 1H, 400.18 MHz, CDCl3
f2 (ppm)
f1 (ppm)

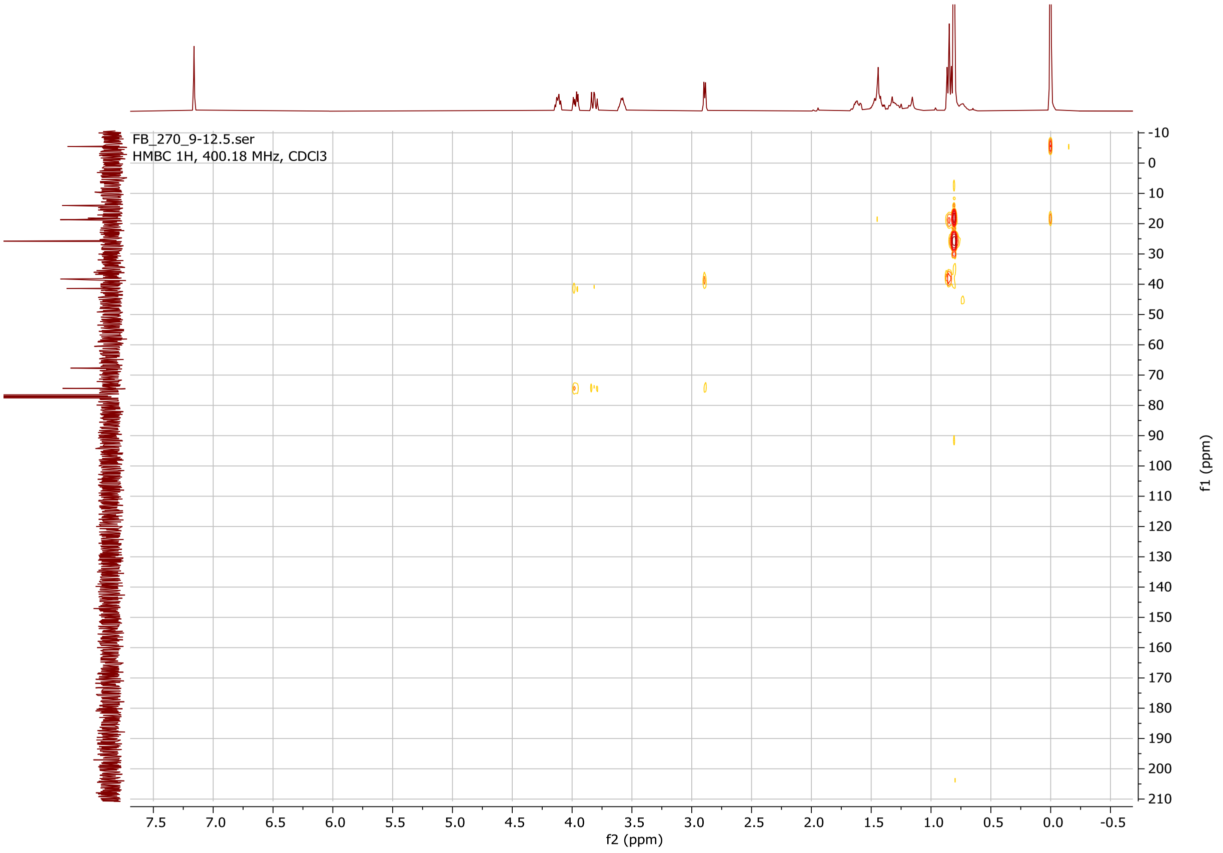
FB_270_9-12.5.ser
HMBC 1H, 400.18 MHz, CDCl3
f2 (ppm)
f1 (ppm)

2-Iodohexan-1-ol **3j**

FB_427_63.1.fid
1D 1H, 400.18 MHz, CDCl3

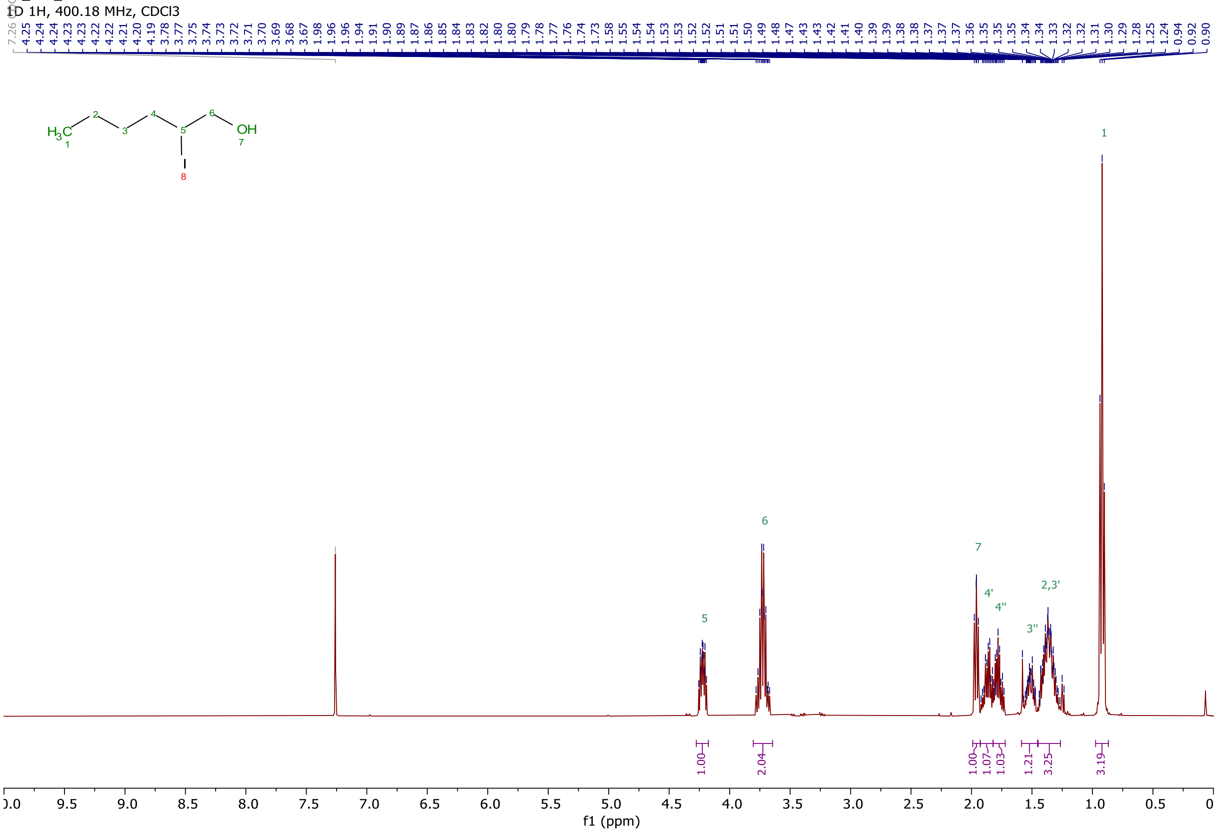

FB_427_63.4.fid
1D 13C{1H}, 100.64 MHz, CDCl3

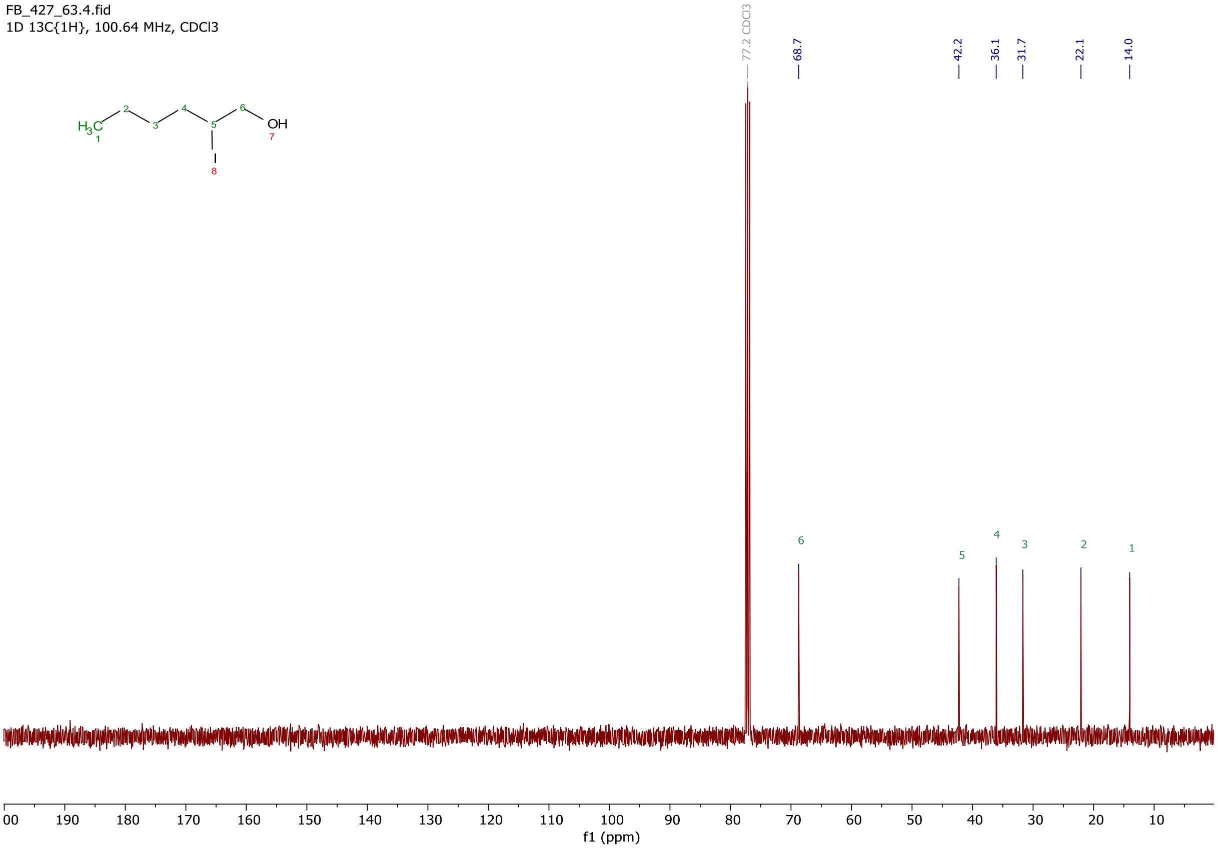

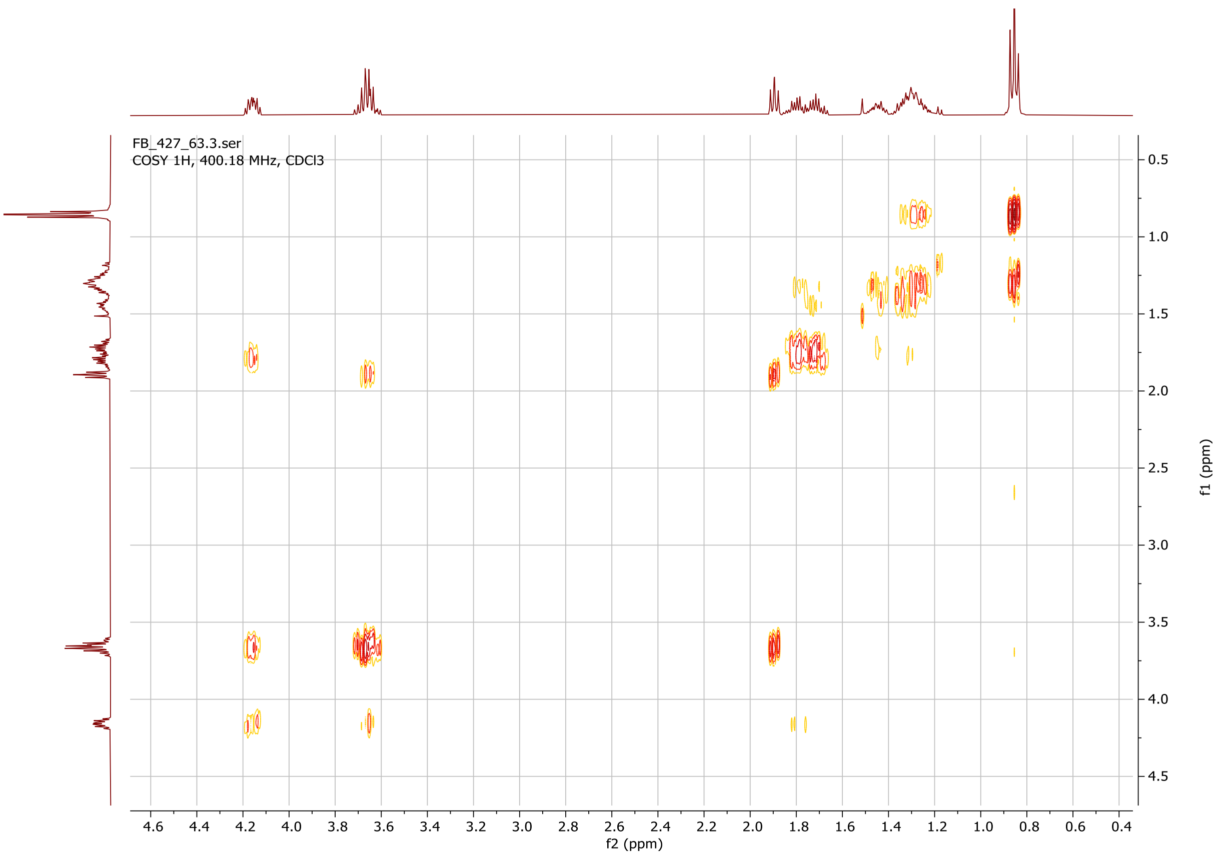
FB_427_63.3.ser
COSY 1H, 400.18 MHz, CDCl3
f2 (ppm)
f1 (ppm)

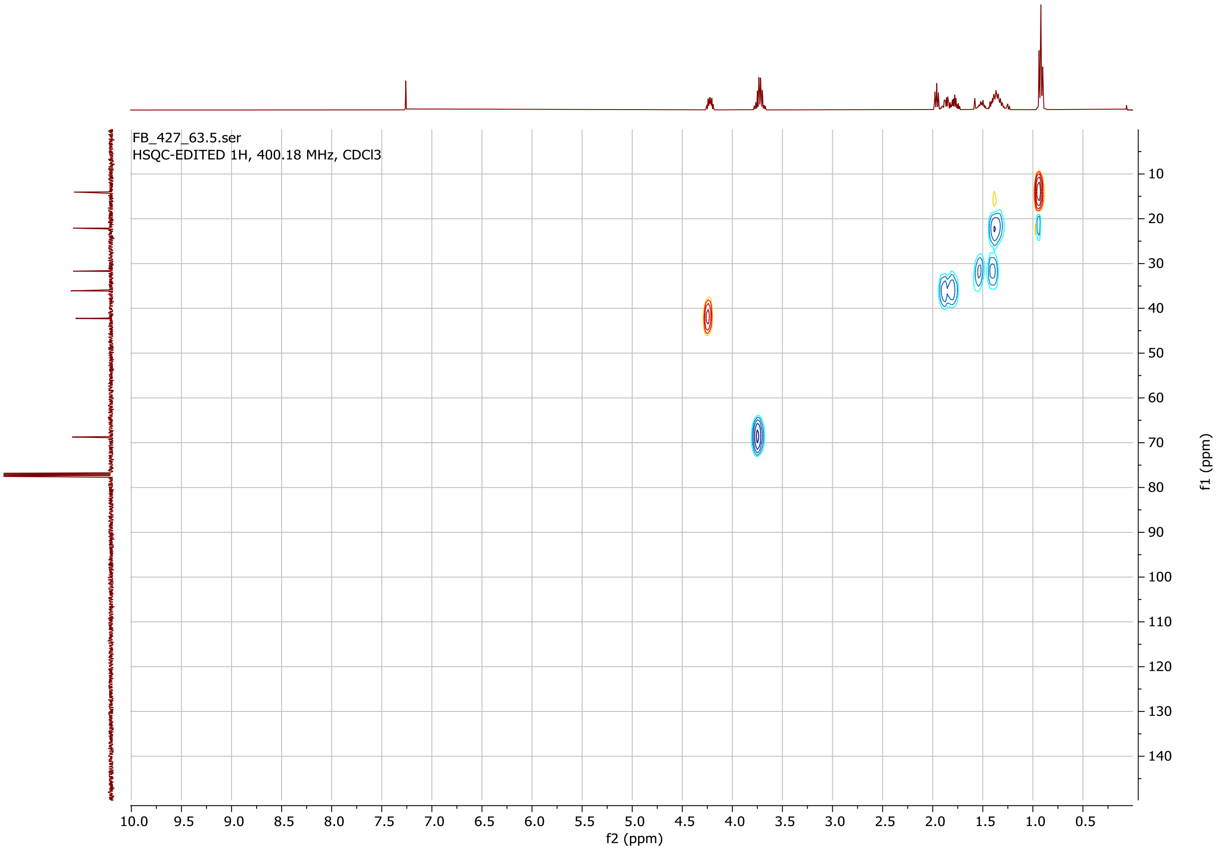
FB_427_63.5.ser
HSQC-EDITED 1H, 400.18 MHz, CDCl3
f2 (ppm)
f1 (ppm)

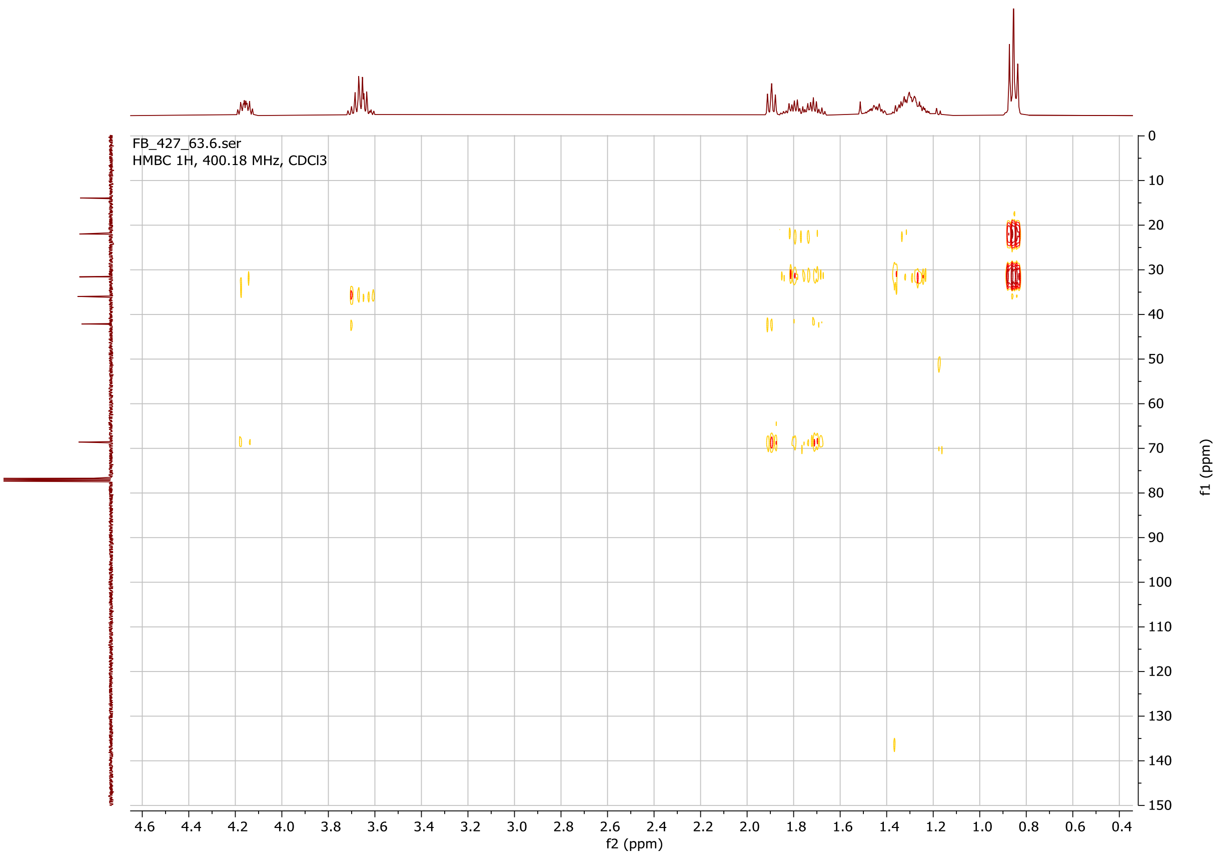

1-Iodohexan-2-ol **3k**

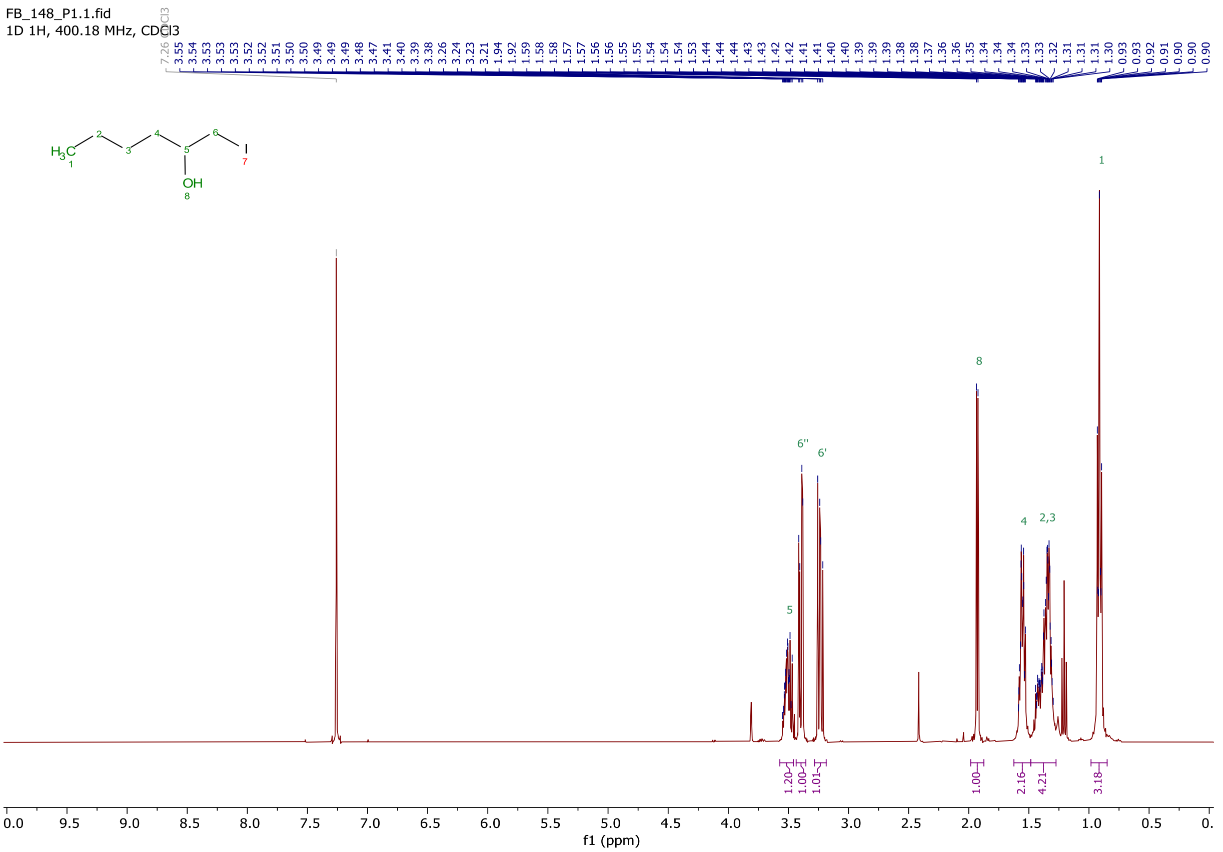

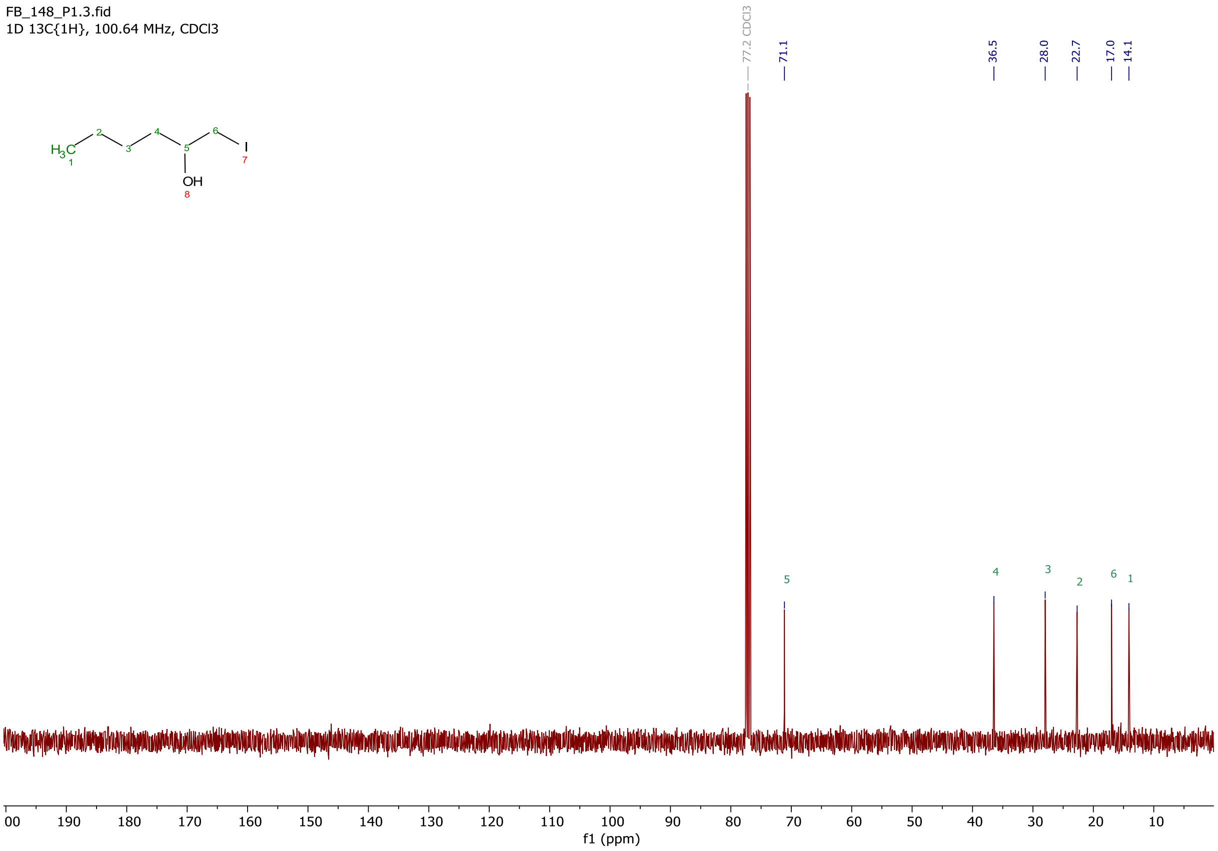
FB_148_P1.3.fid
1D 13C{1H}, 100.64 MHz, CDCl3
77.2 CDCl3
71.1
36.5
28.0
22.7
17.0
14.1
OH
f1 (ppm)

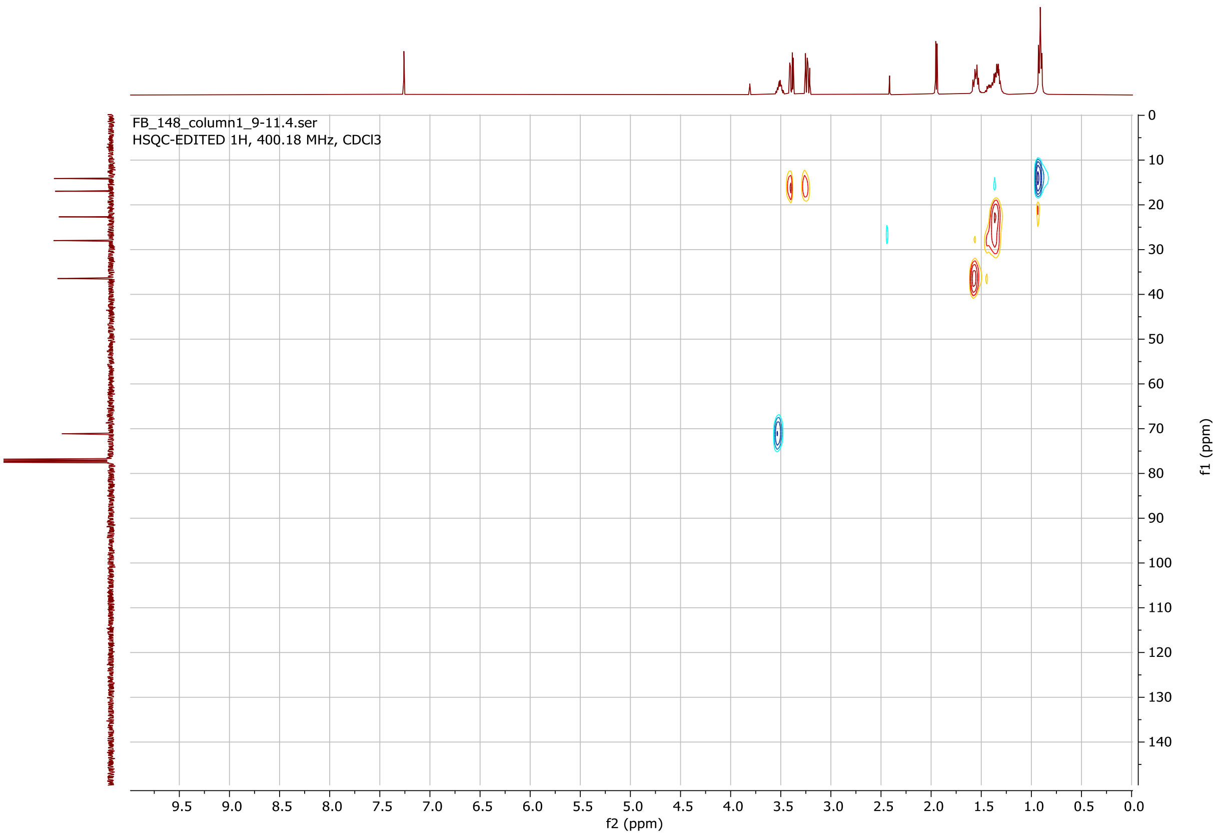
FB_148_column1_9-11.4.ser
HSQC-EDITED 1H, 400.18 MHz, CDCl3
f2 (ppm)
f1 (ppm)

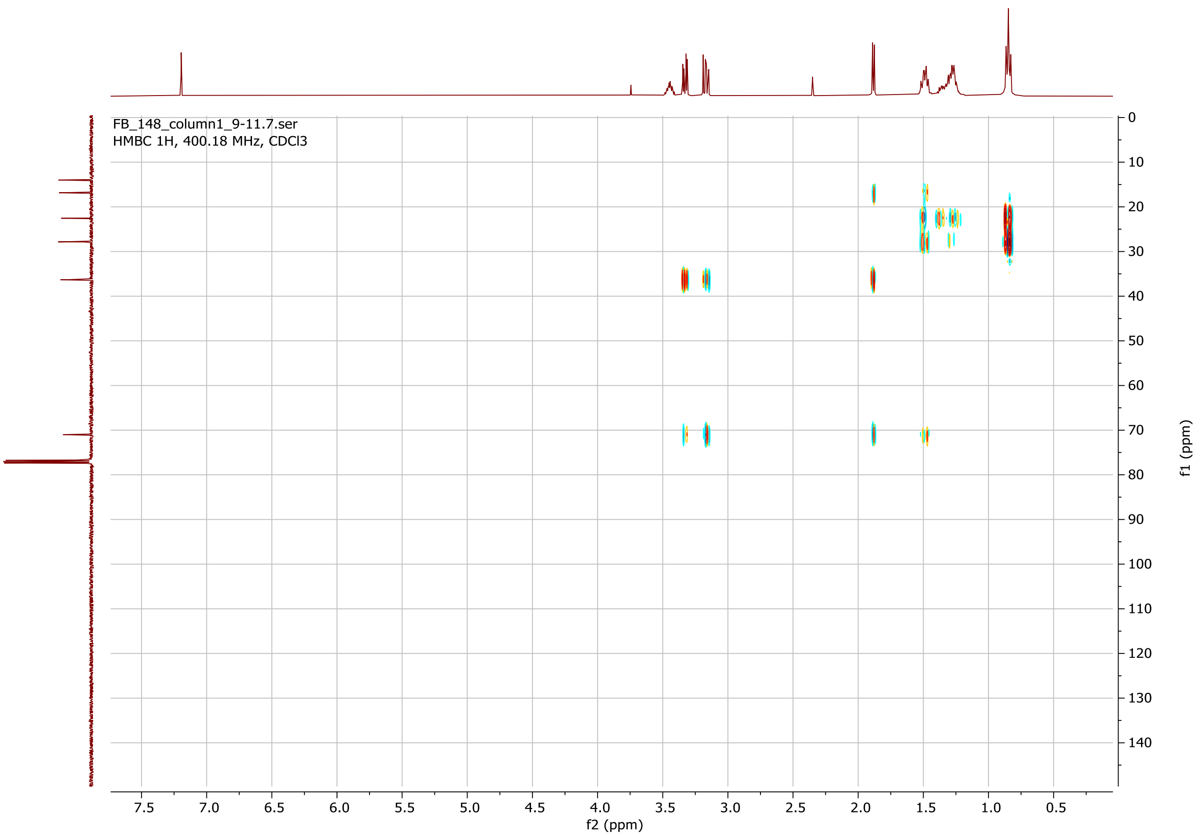

1-Iodo-4-phenylbutan-2-ol **3l**

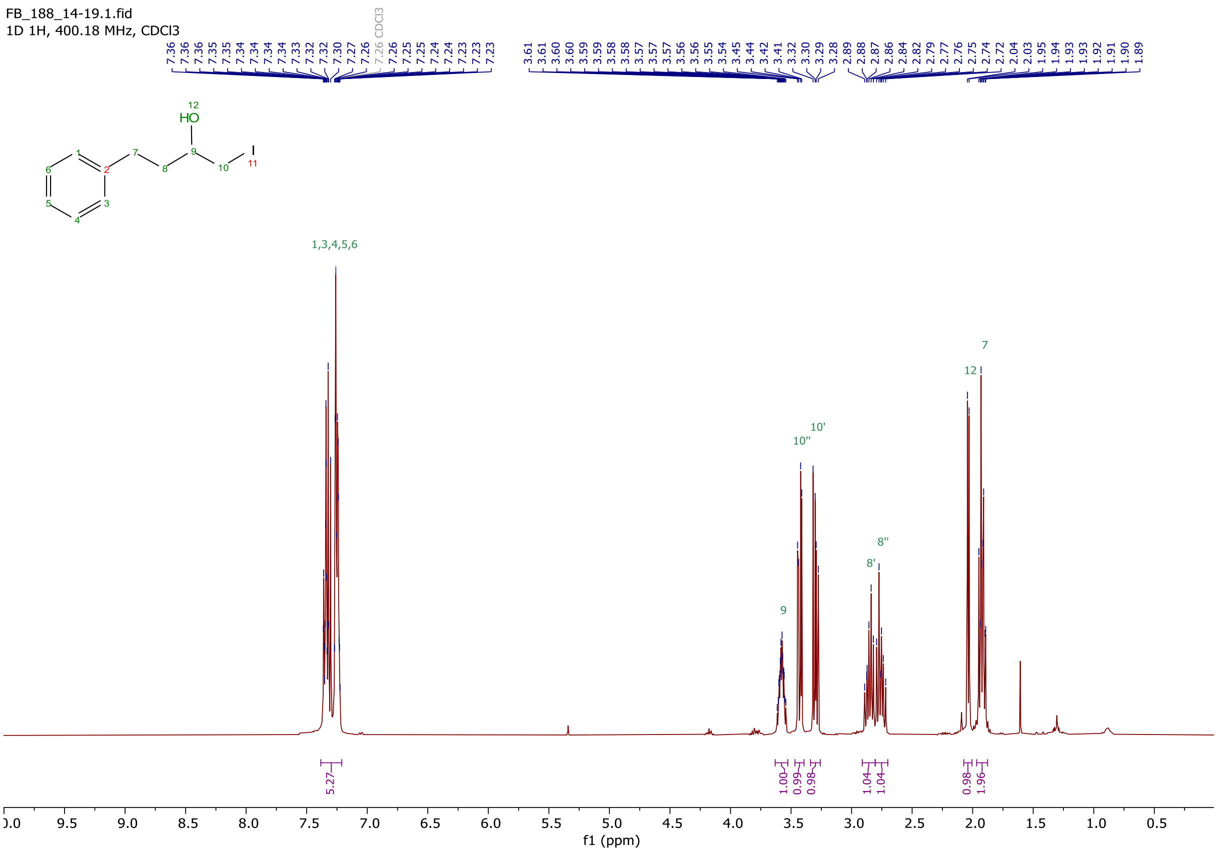

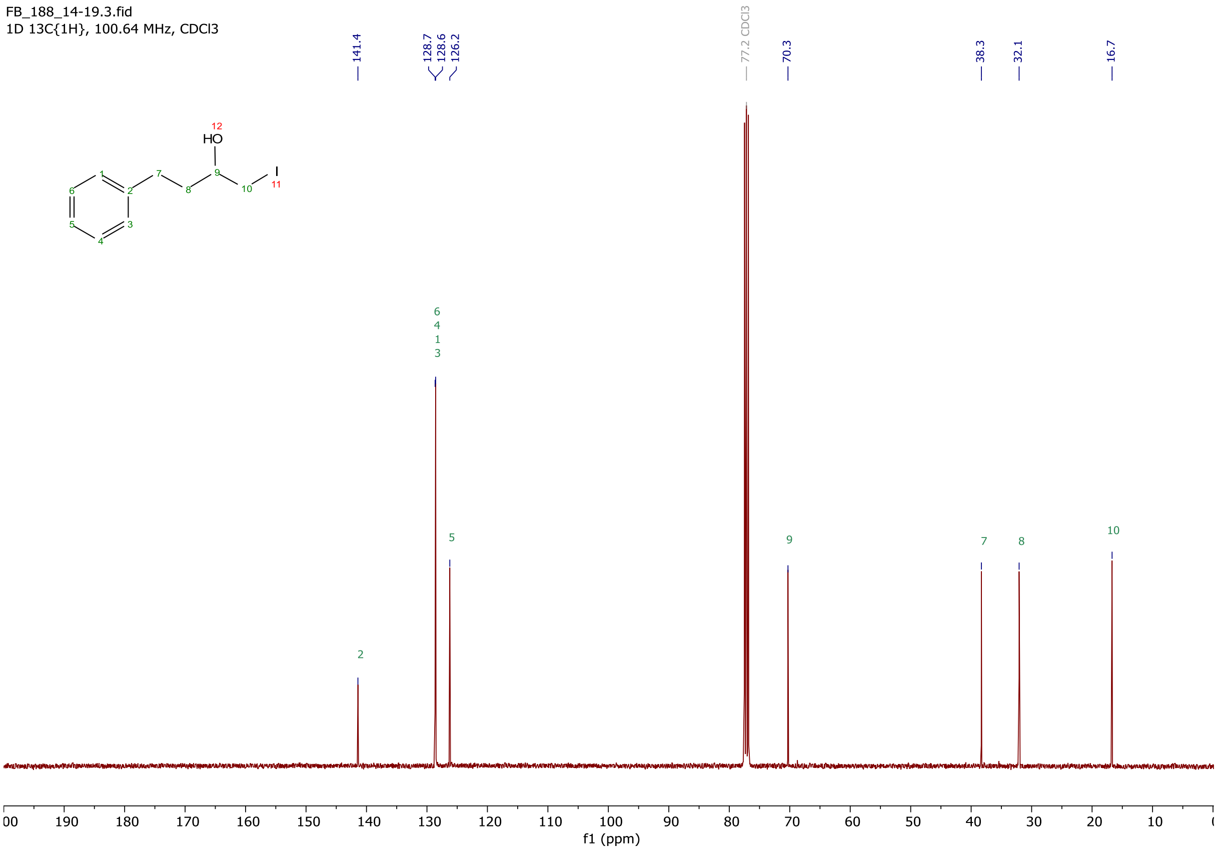
FB_188_14-19.3.fid
1D 13C{1H}, 100.64 MHz, CDCl3
141.4
128.7
128.6
126.2
77.2 CDCl3
70.3
38.3
32.1
16.7
HO
I
f1 (ppm)

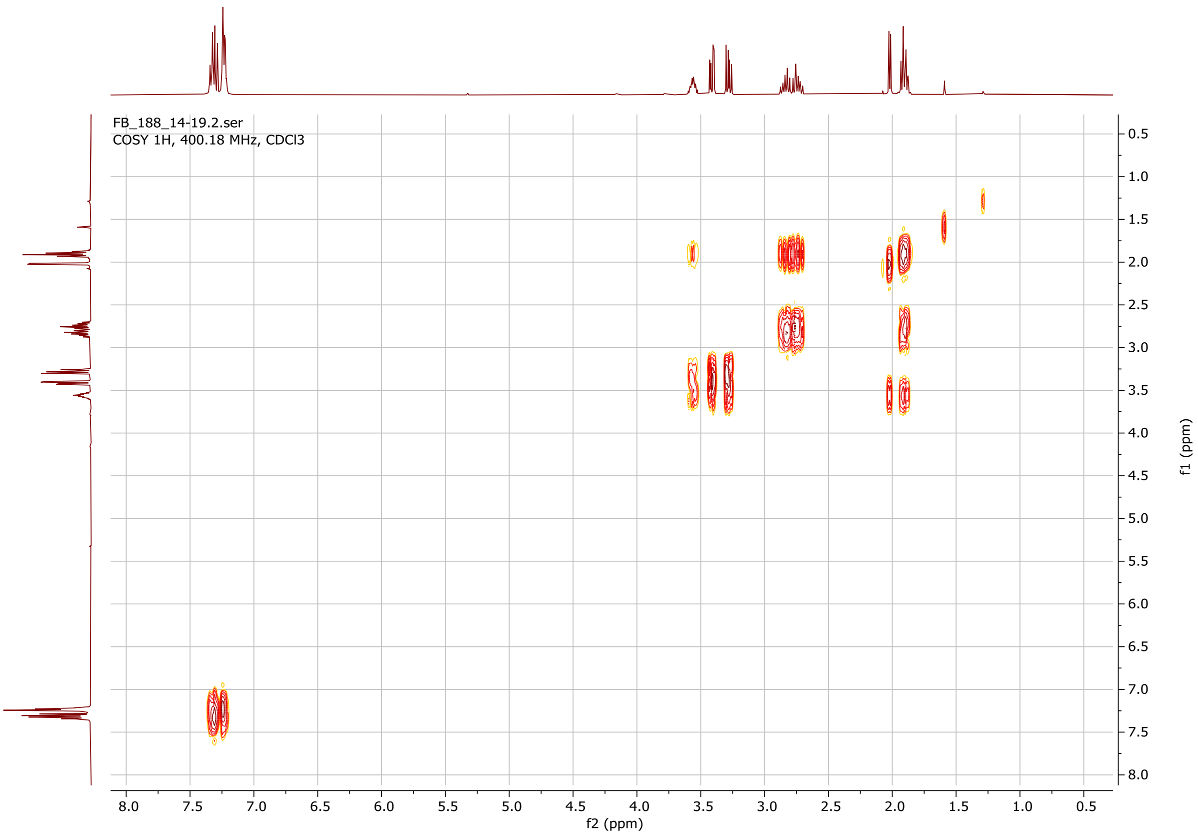
FB_188_14-19.2.ser
COSY 1H, 400.18 MHz, CDCl3
f2 (ppm)
f1 (ppm)

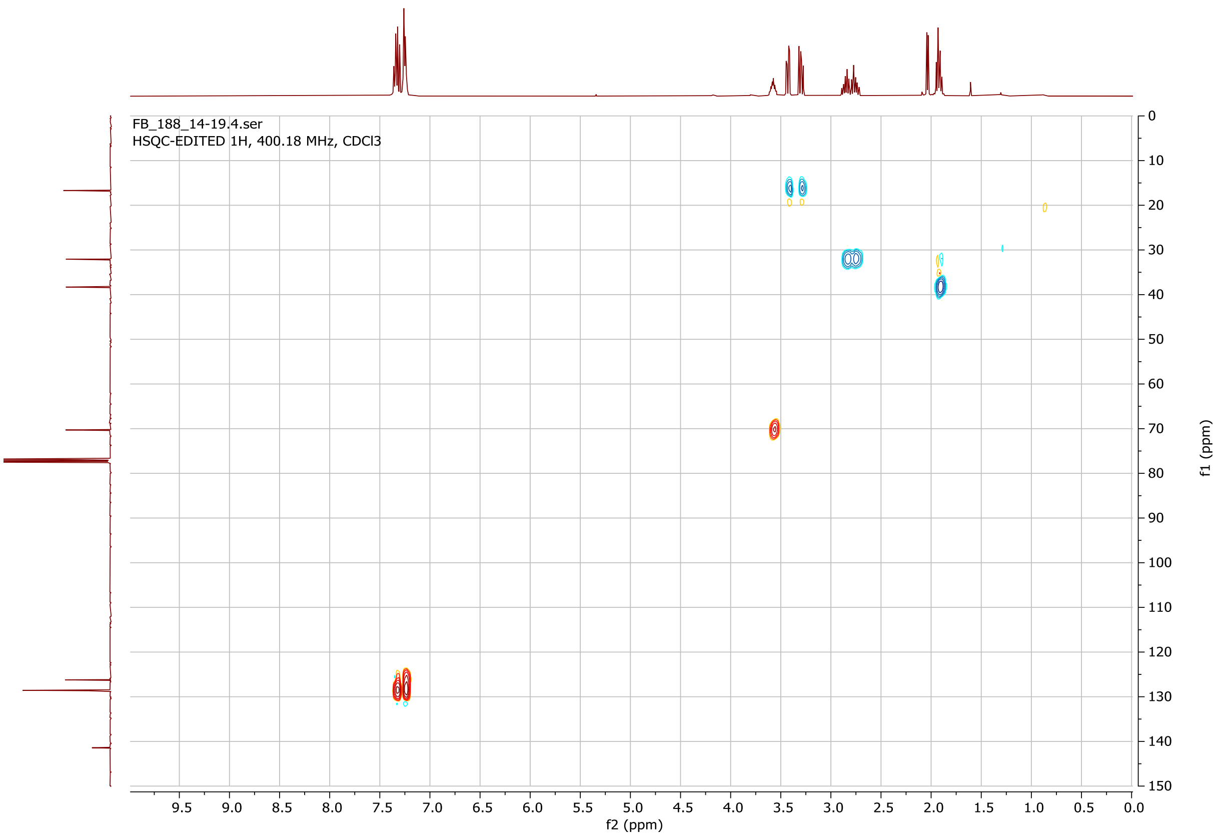
FB_188_14-19.4.ser
HSQC-EDITED 1H, 400.18 MHz, CDCl3
f2 (ppm)
f1 (ppm)

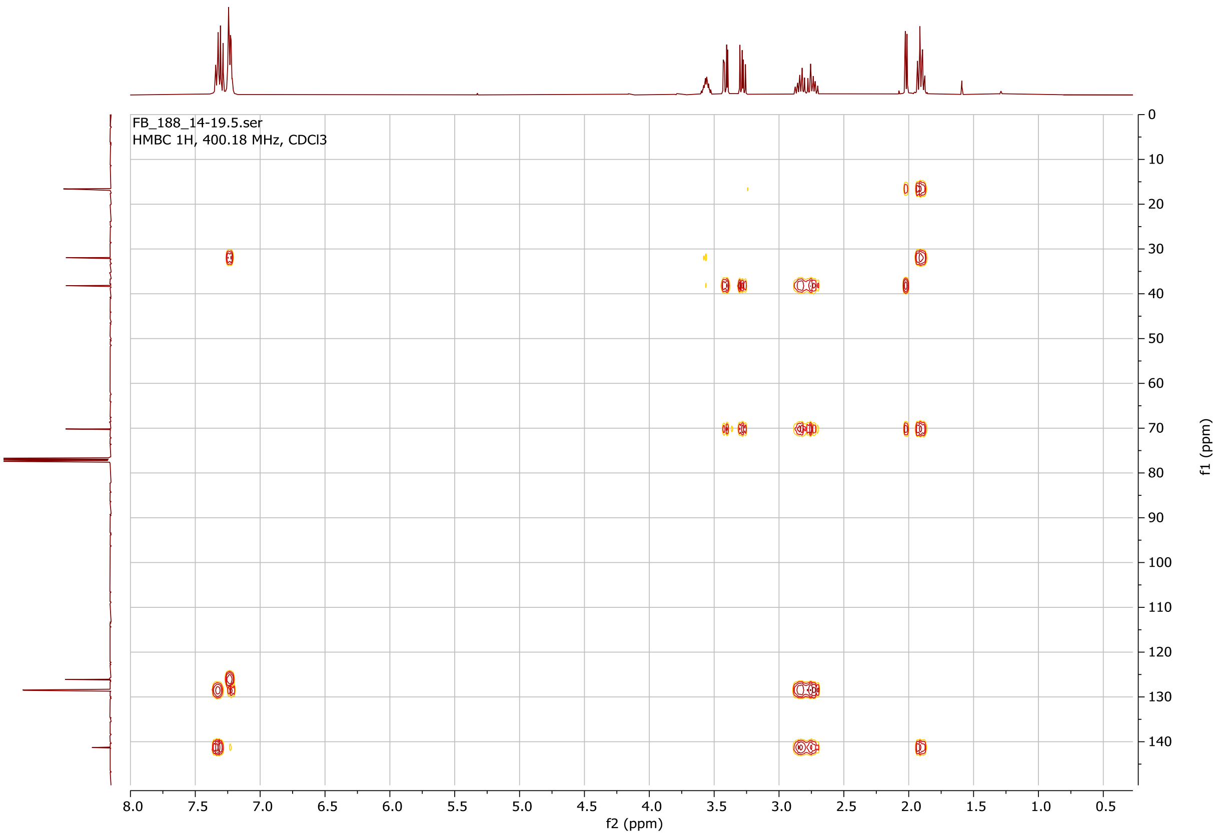
FB_188_14-19.5.ser
HMBC 1H, 400.18 MHz, CDCl3
f2 (ppm)
f1 (ppm)

Methyl 4-hydroxy-5-iodopentanoate **3m**

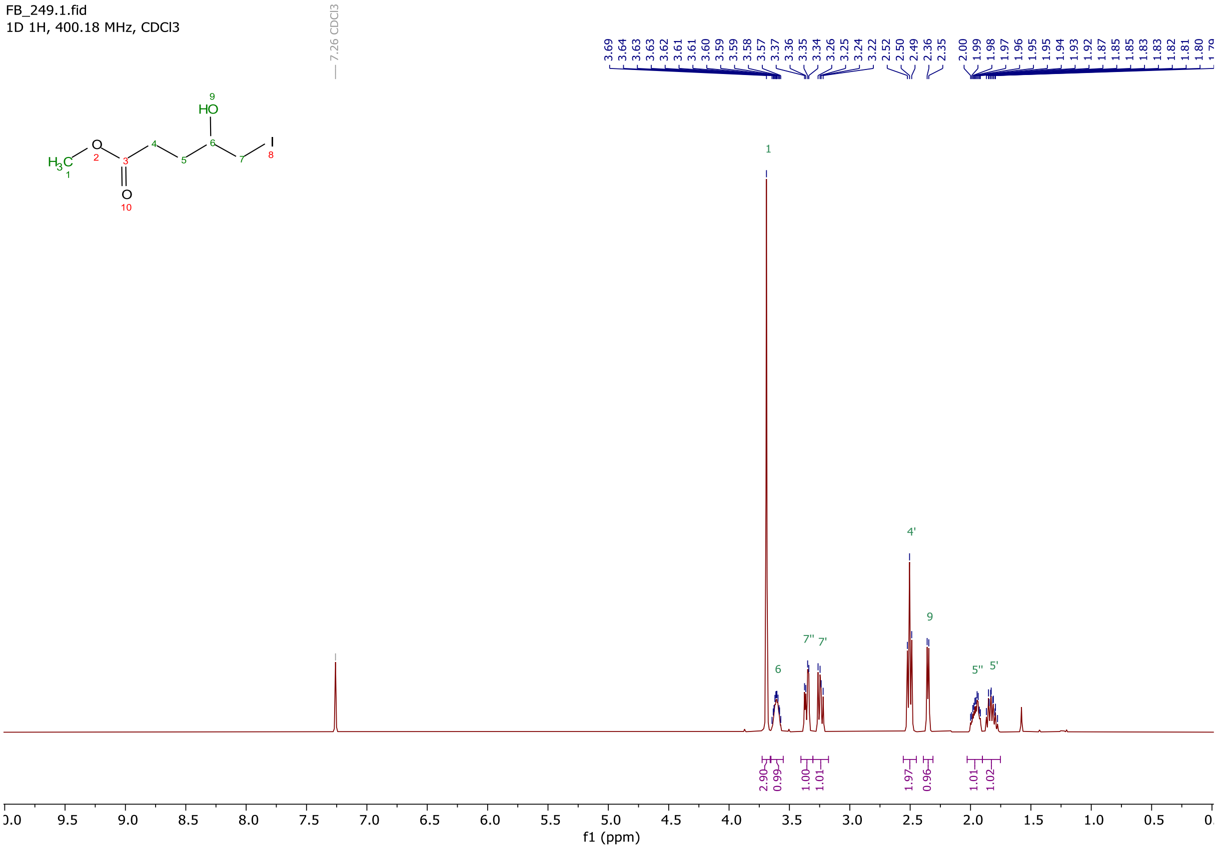

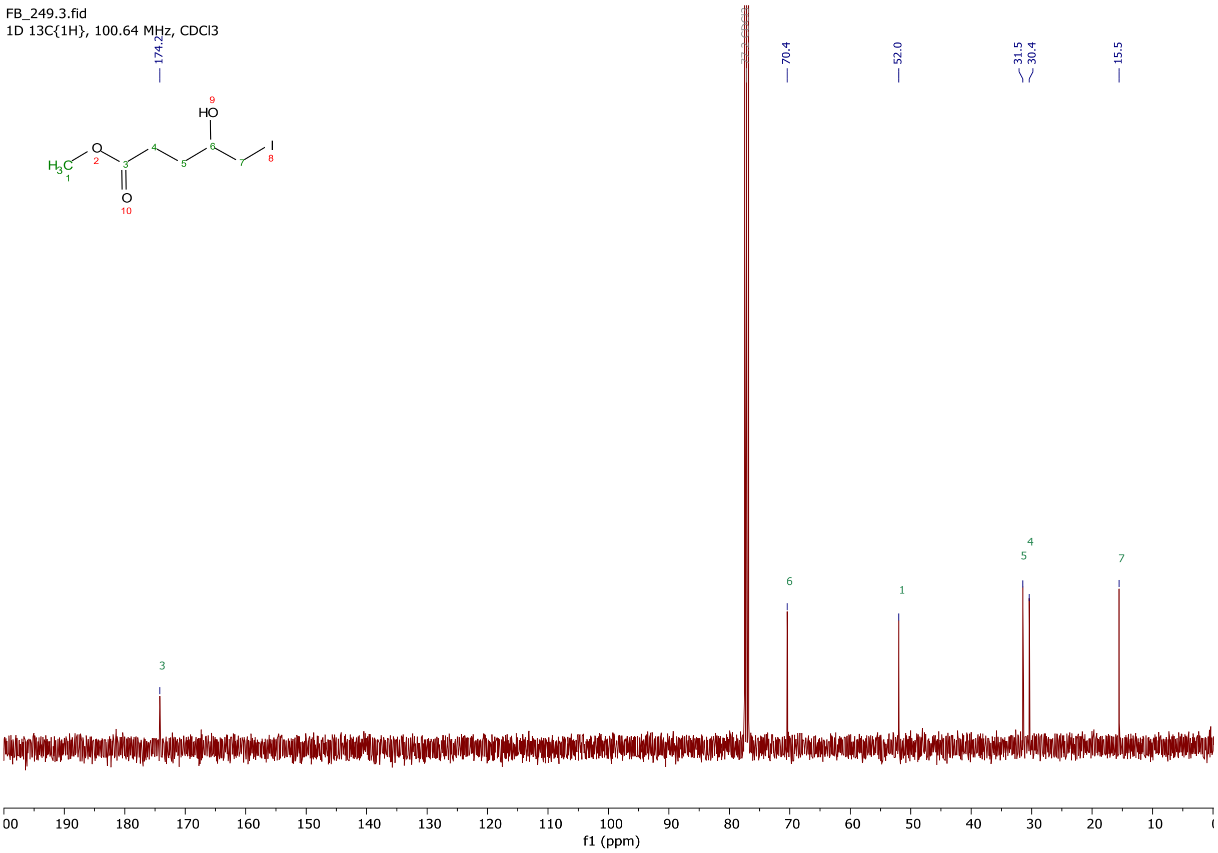

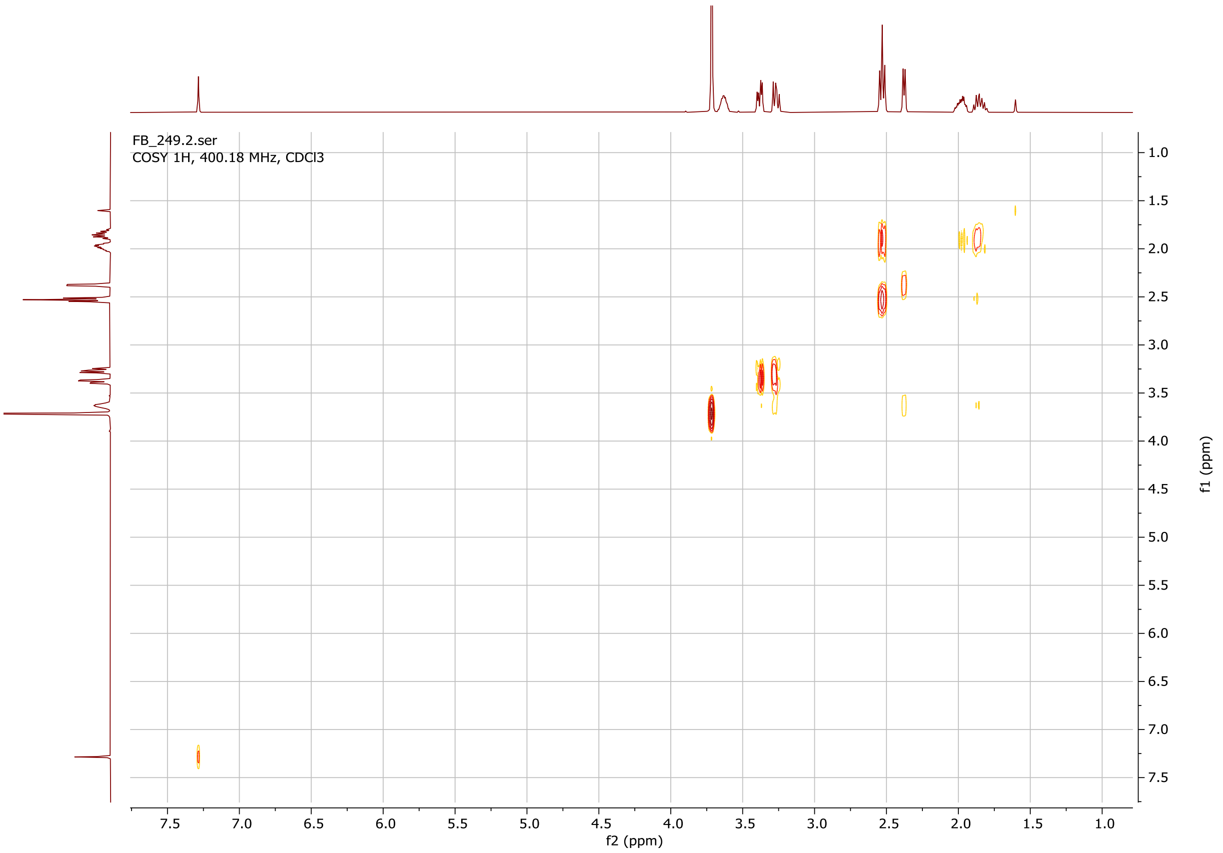
FB_249.2.ser
COSY 1H, 400.18 MHz, CDCl3
f2 (ppm)
f1 (ppm)

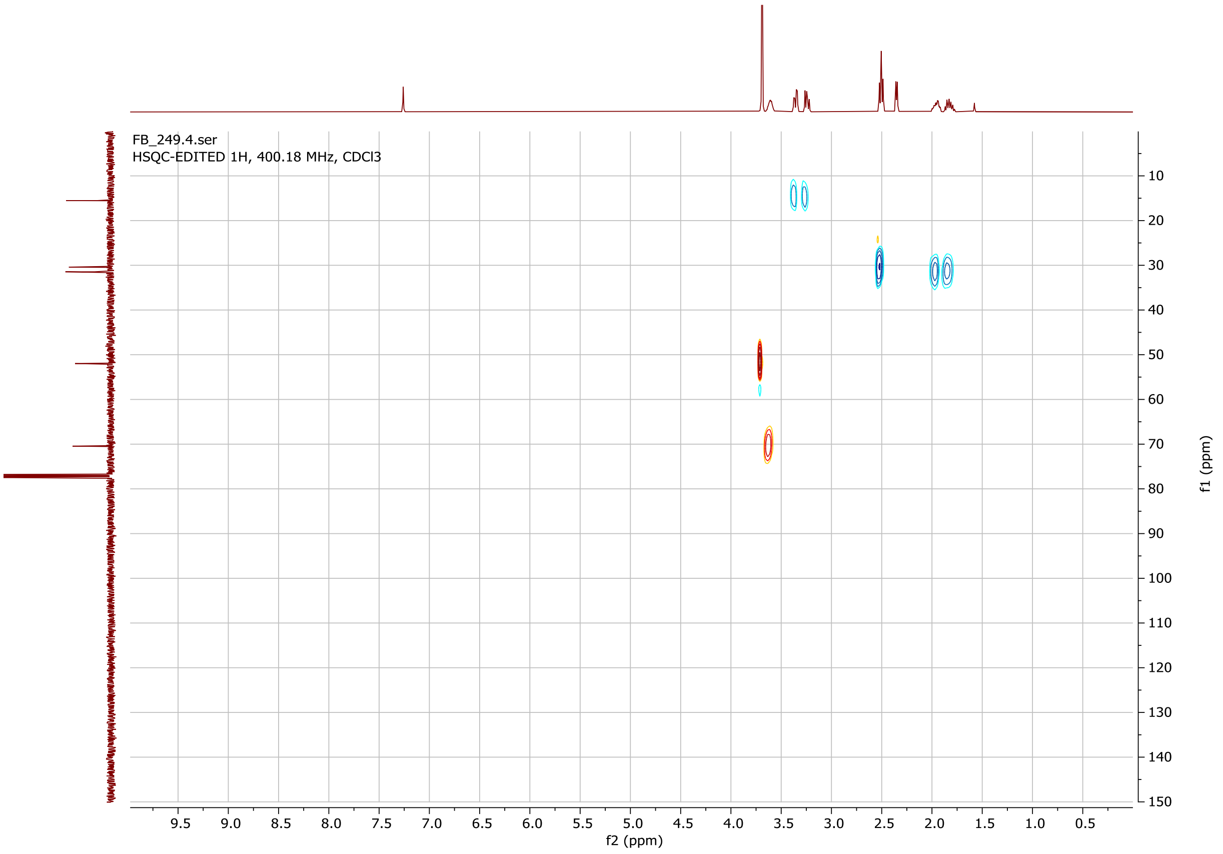
FB_249.4.ser
HSQC-EDITED 1H, 400.18 MHz, CDCl3
f2 (ppm)
f1 (ppm)

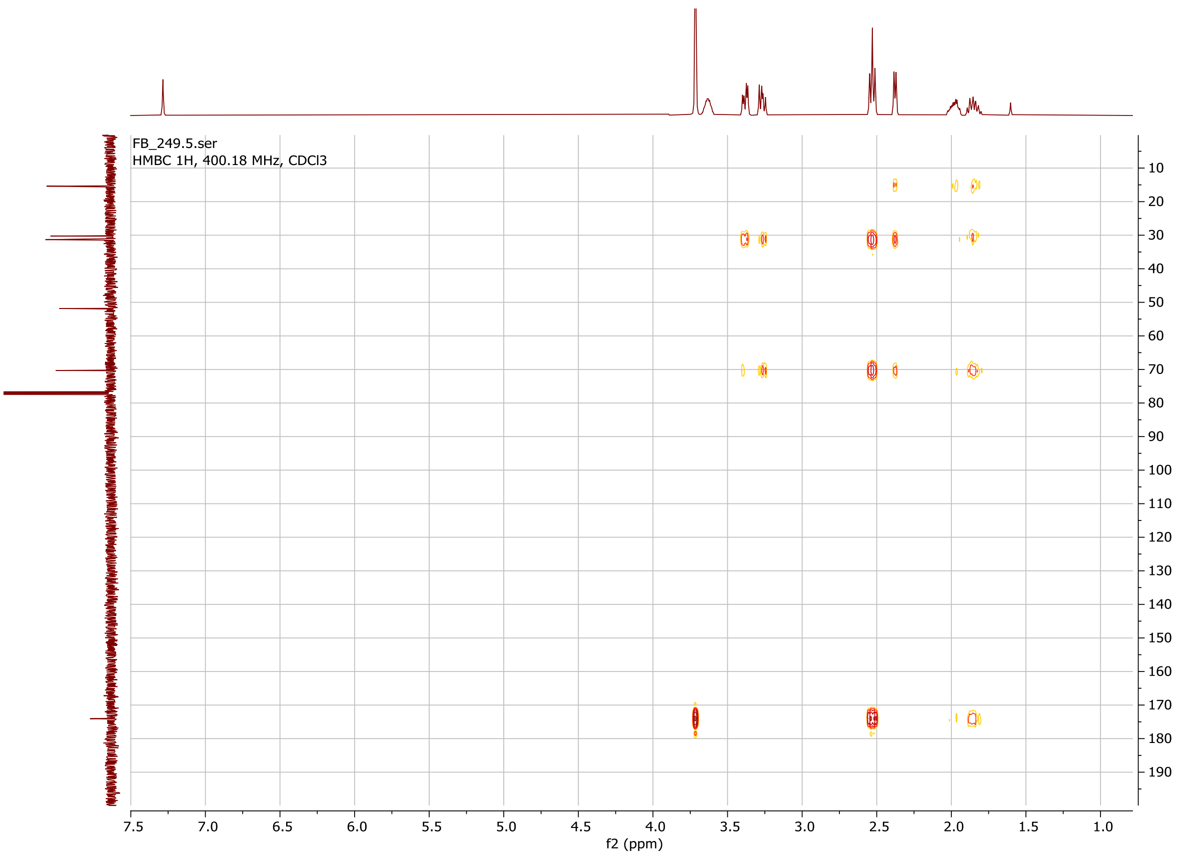

2-Iodo1-phenylethan-1-ol **3n**

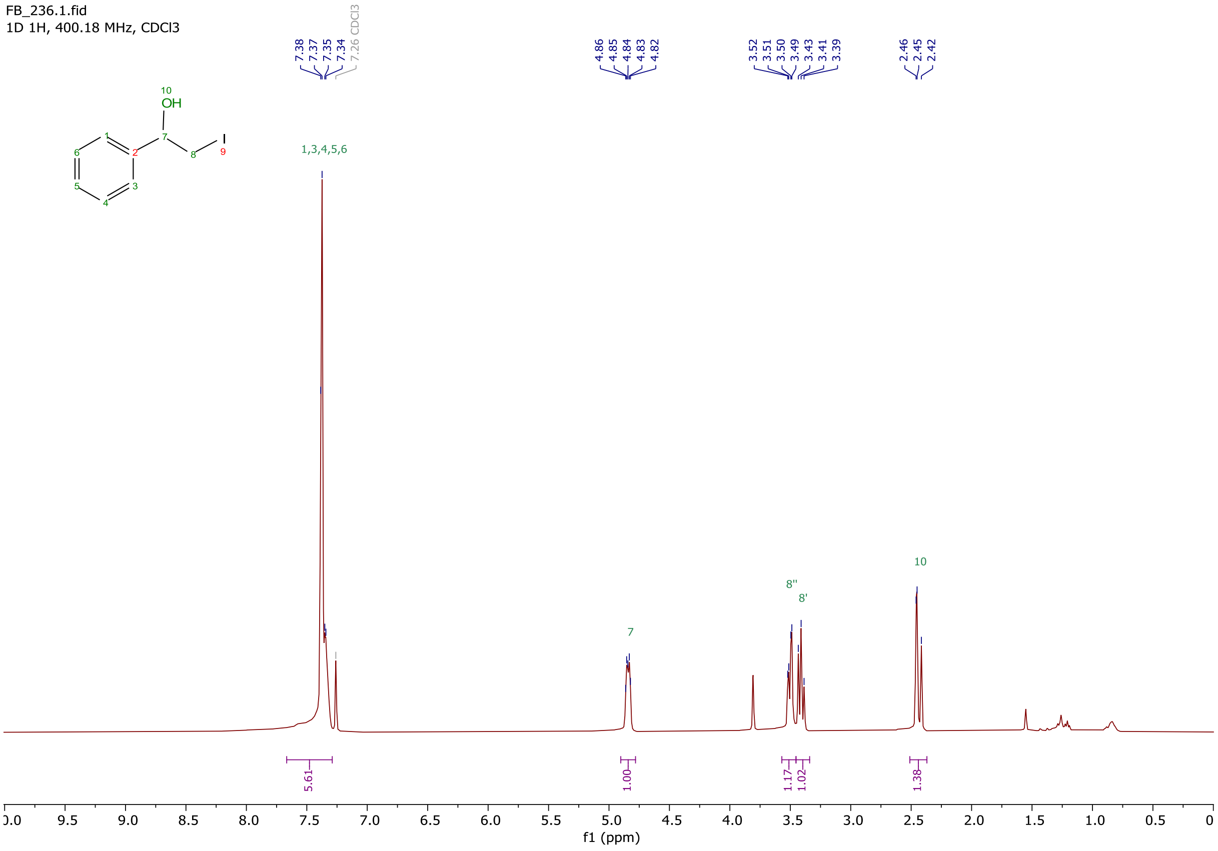

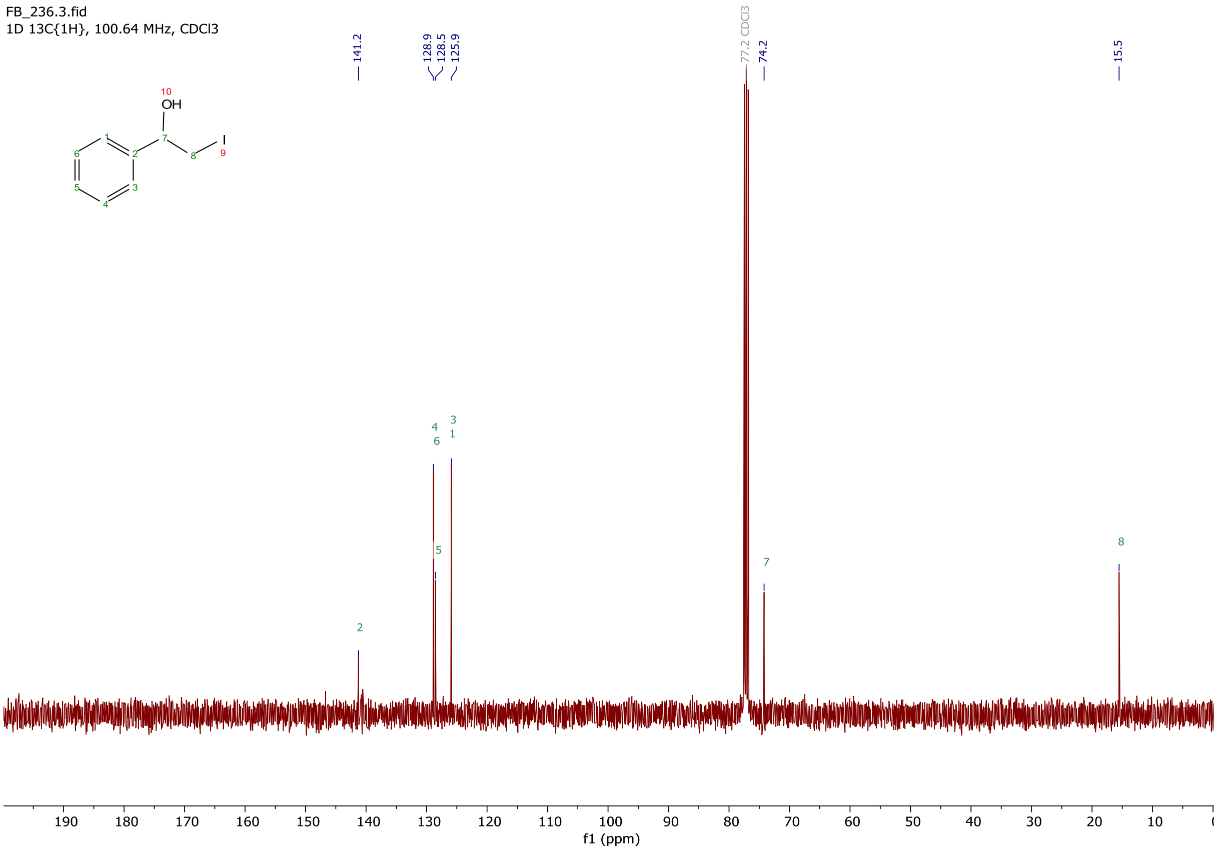
FB_236.3.fid
1D 13C{1H}, 100.64 MHz, CDCl3
141.2
128.9
128.5
125.9
77.2 CDCl3
74.2
15.5
OH
f1 (ppm)

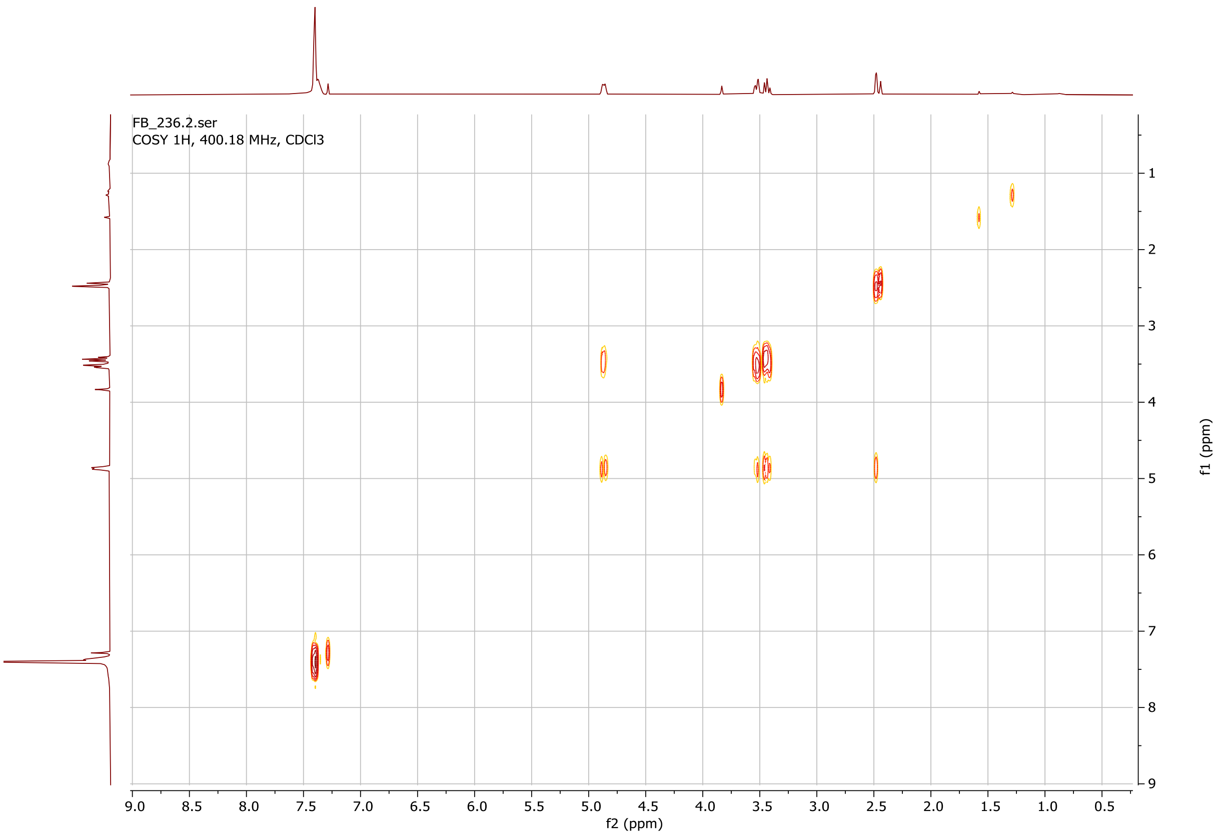
FB_236.2.ser
COSY 1H, 400.18 MHz, CDCl3
f2 (ppm)
f1 (ppm)

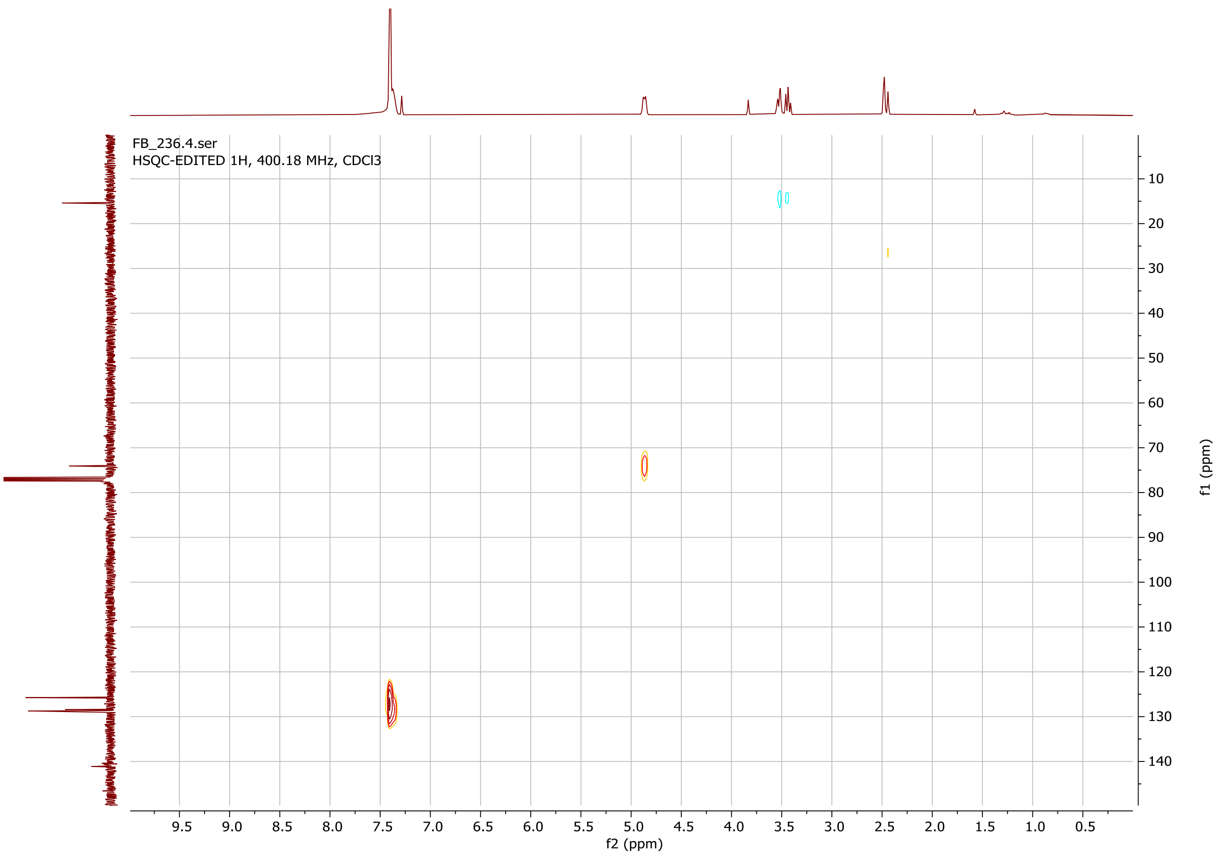

FB_236.4.ser
HSQC-EDITED 1H, 400.18 MHz, CDCl3
f1 (ppm)
f2 (ppm)

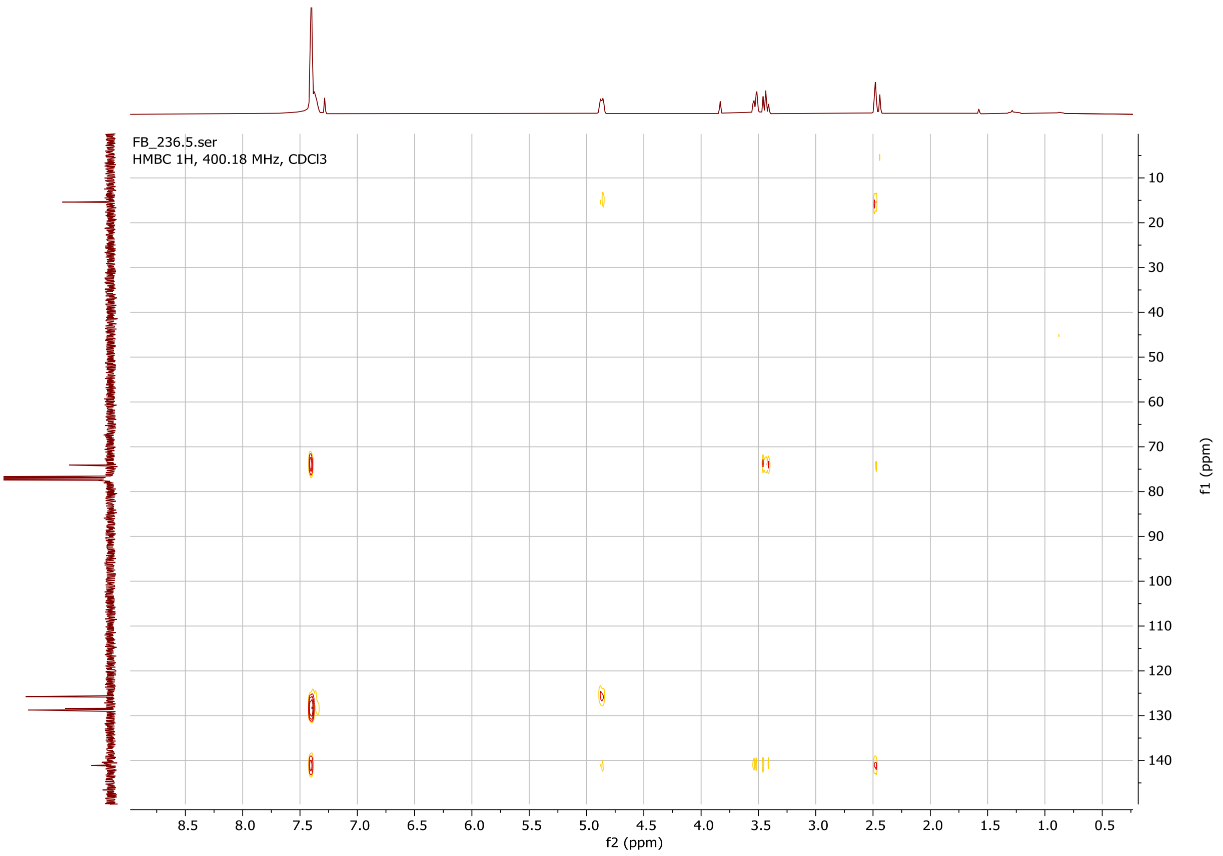

FB_236.5.ser
HMBC 1H, 400.18 MHz, CDCl3
f1 (ppm)
f2 (ppm)

2-Iodo-1-(4-methoxyphenyl)ethan-1-ol **3o**

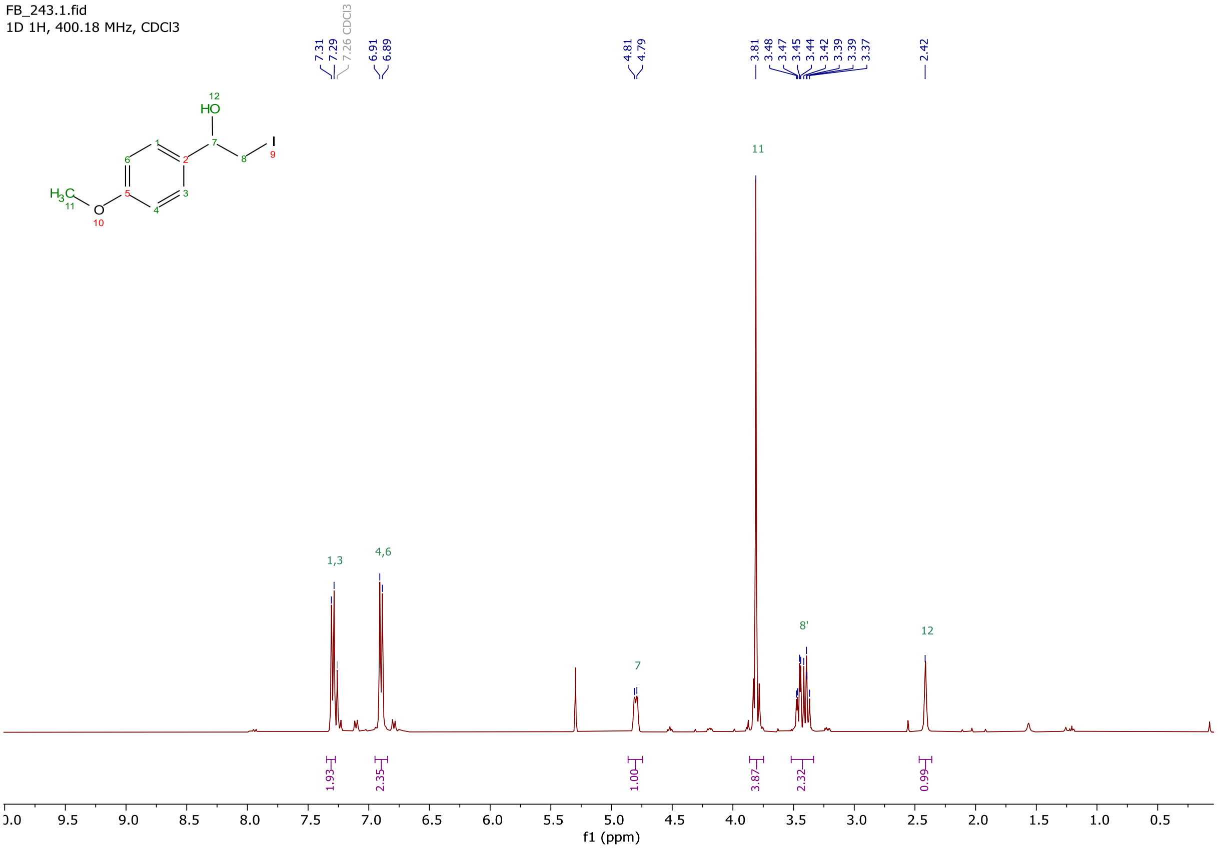

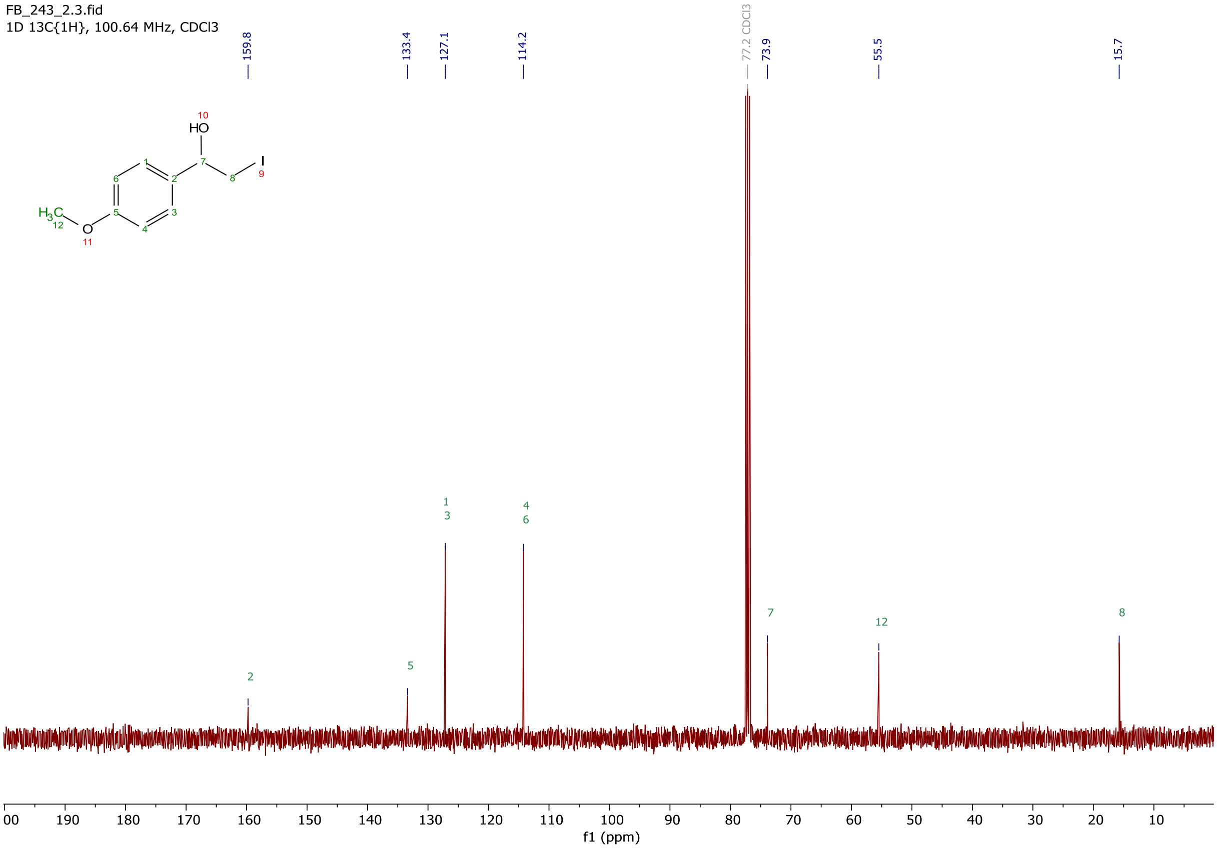

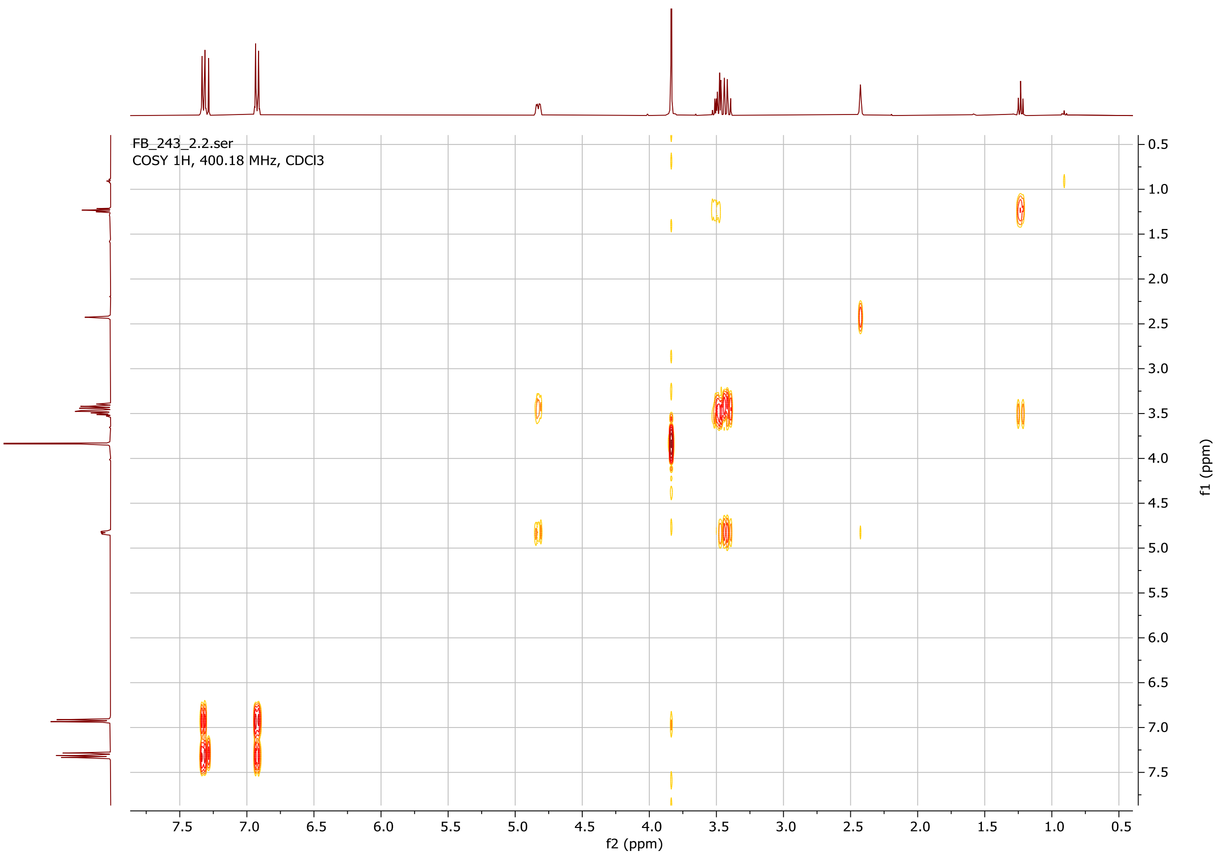
FB_243_2.2.ser
COSY 1H, 400.18 MHz, CDCl3
f2 (ppm)
f1 (ppm)

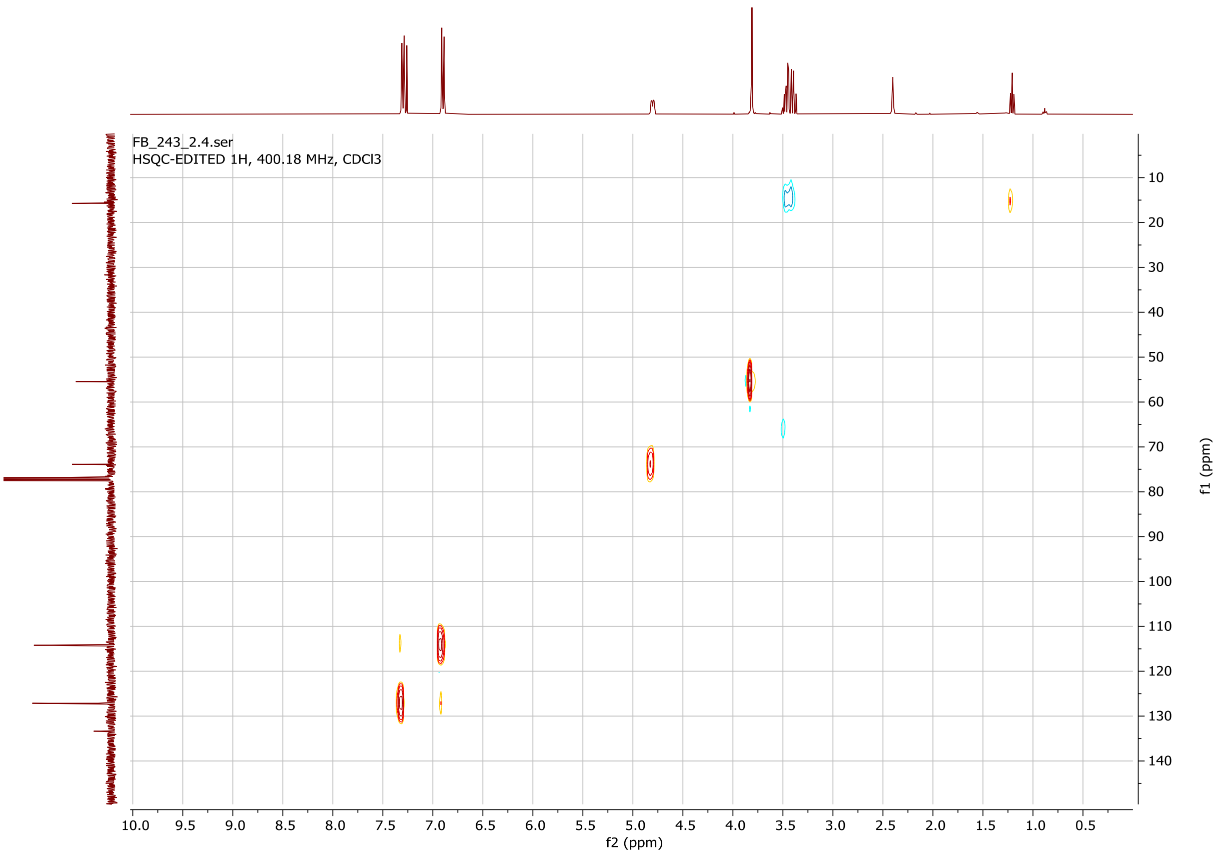
FB_243_2.4.ser
HSQC-EDITED 1H, 400.18 MHz, CDCl3
f2 (ppm)
f1 (ppm)

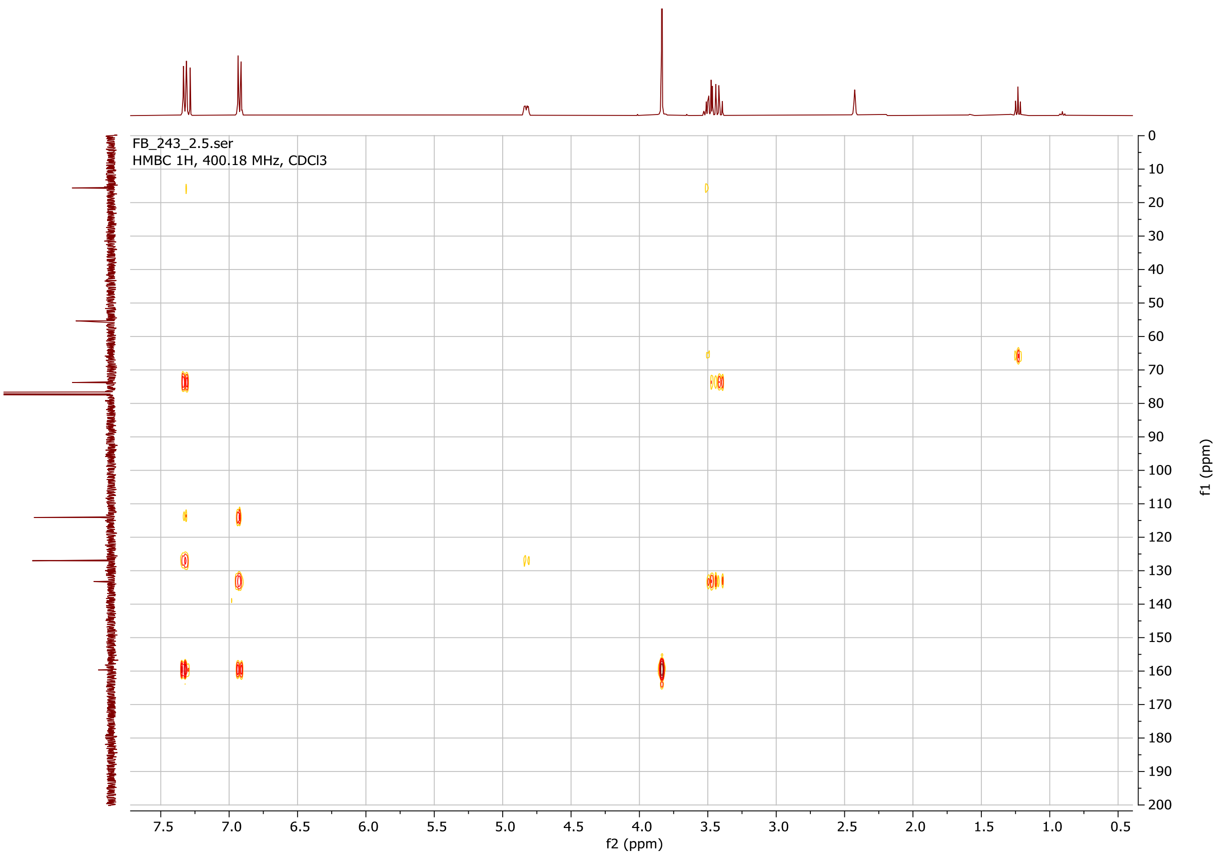

2-Iodo-1-(4-(trifluromethyl)phenyl)ethan-1-ol **3p**

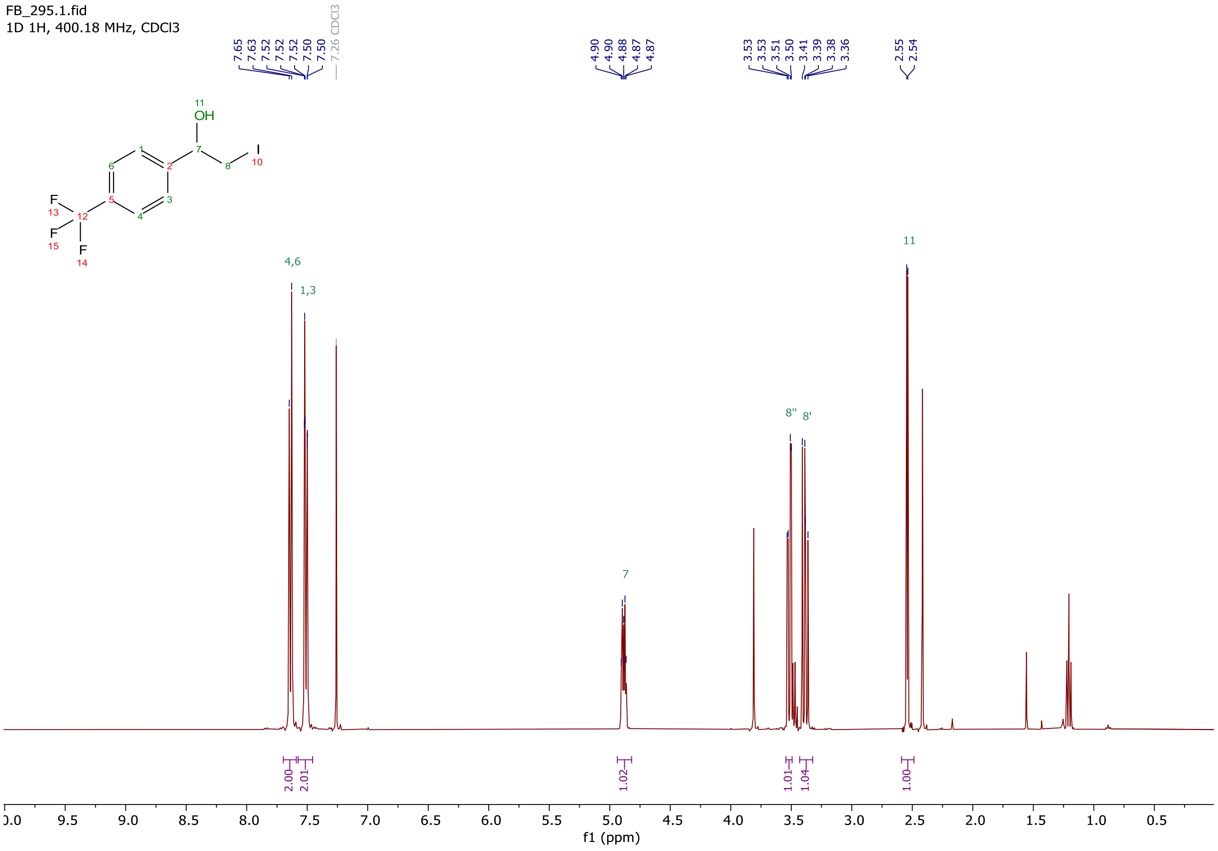

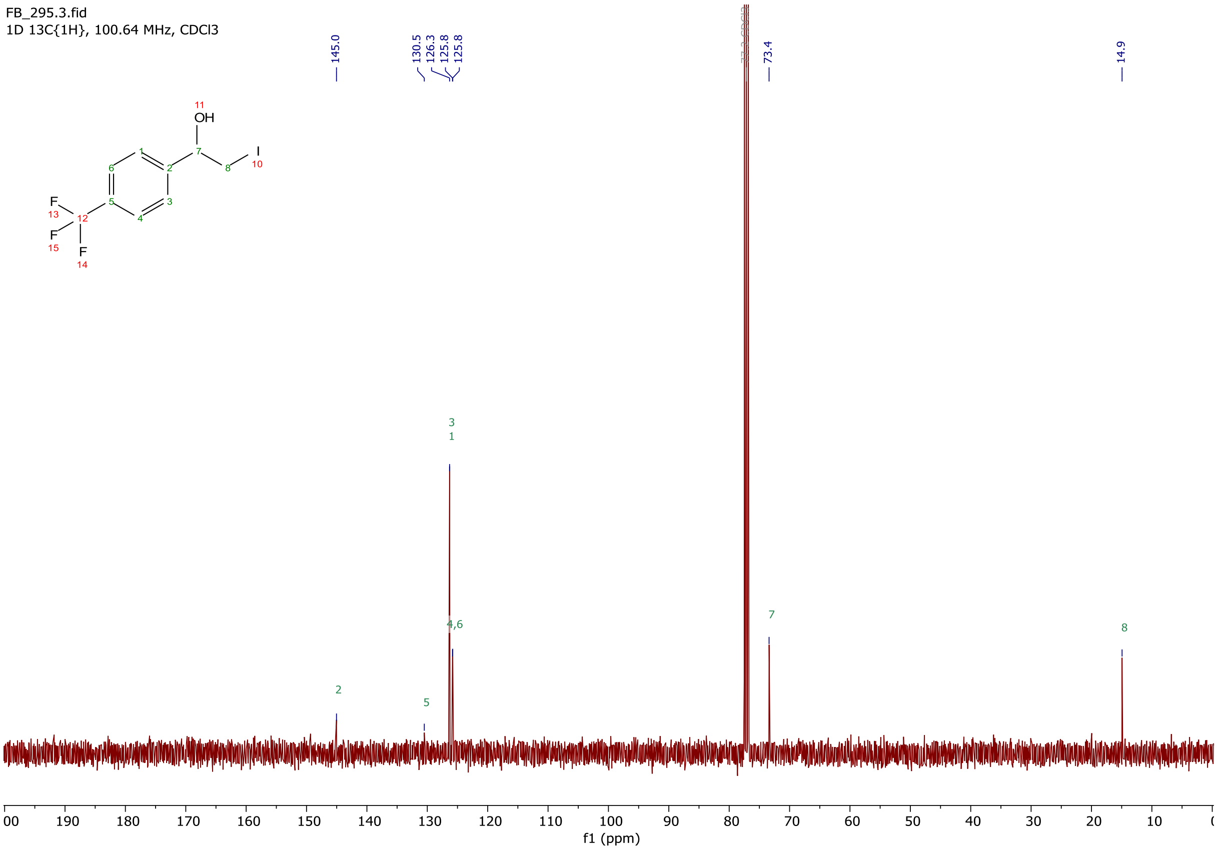
FB_295.3.fid
1D 13C{1H}, 100.64 MHz, CDCl3
145.0
130.5
126.3
125.8
125.8
73.4
14.9
OH
F
F
F
f1 (ppm)

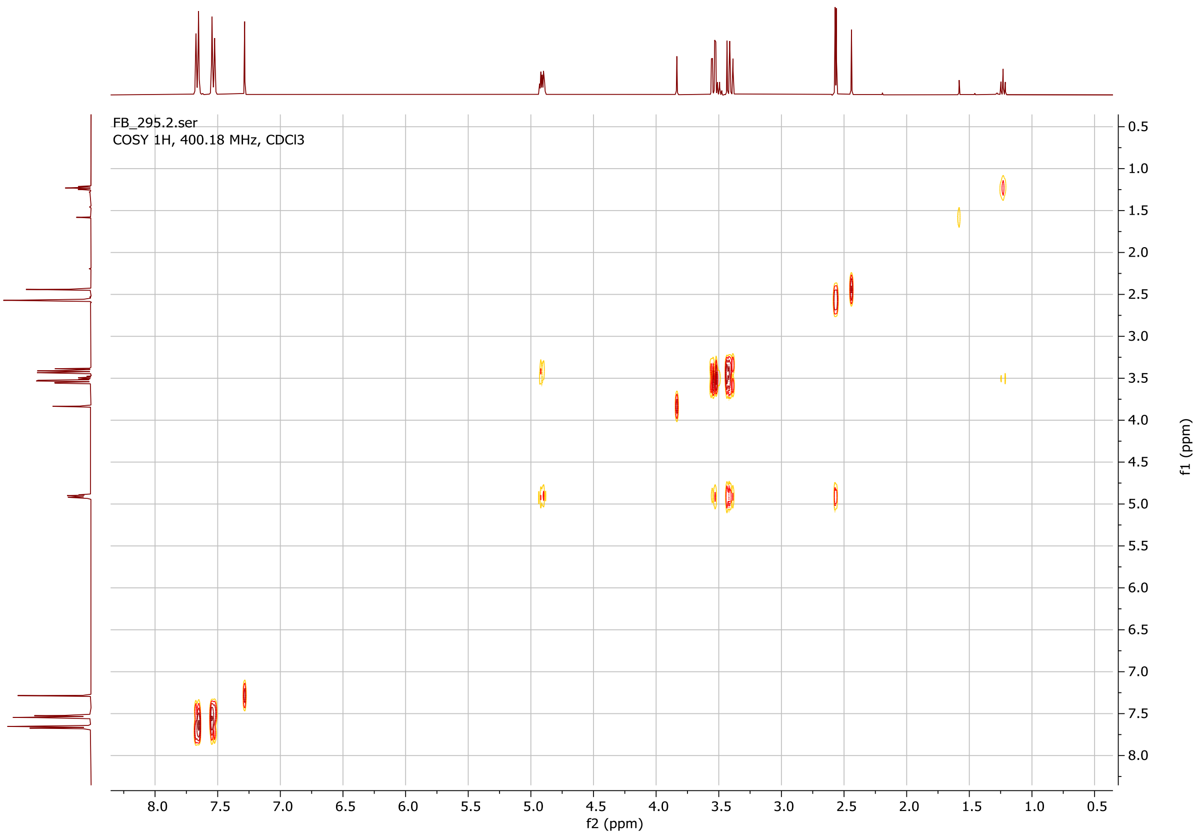
FB_295.2.ser
COSY 1H, 400.18 MHz, CDCl3
f2 (ppm)
f1 (ppm)

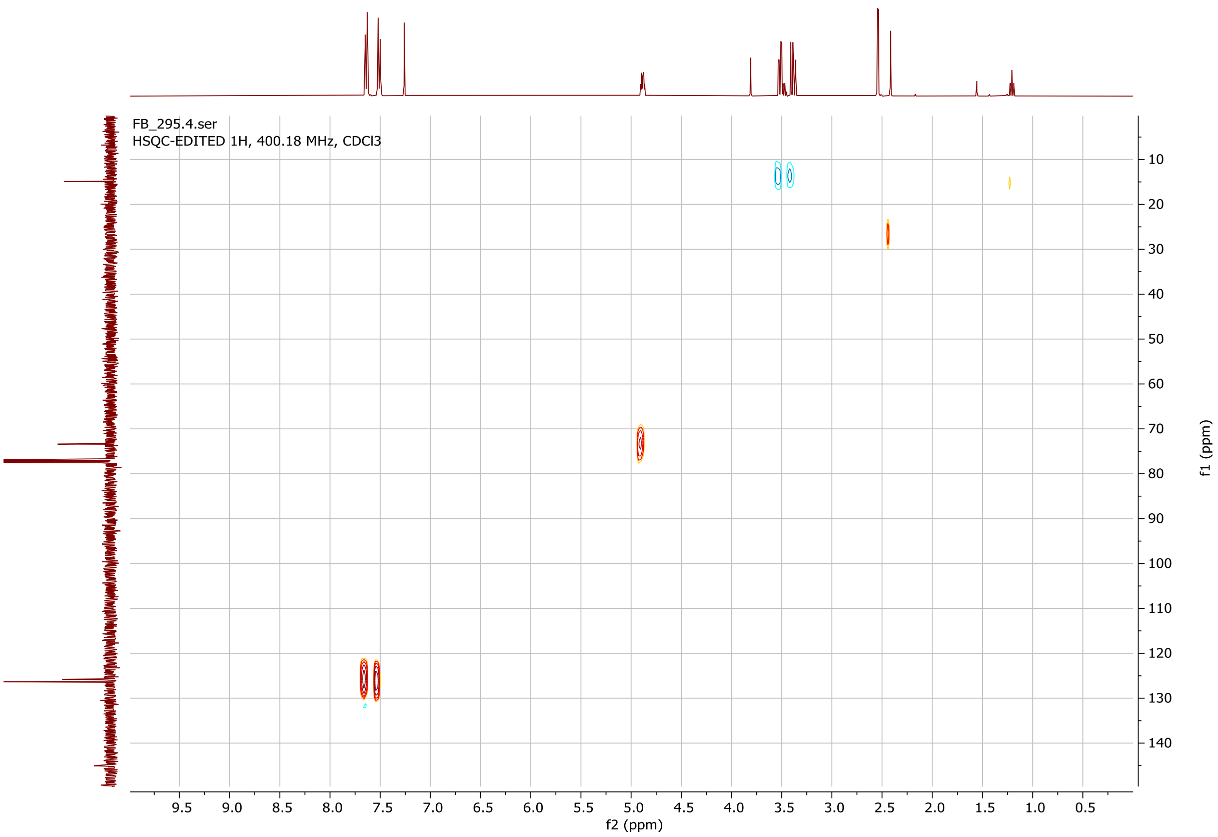
FB_295.4.ser
HSQC-EDITED 1H, 400.18 MHz, CDCl3
f2 (ppm)
f1 (ppm)

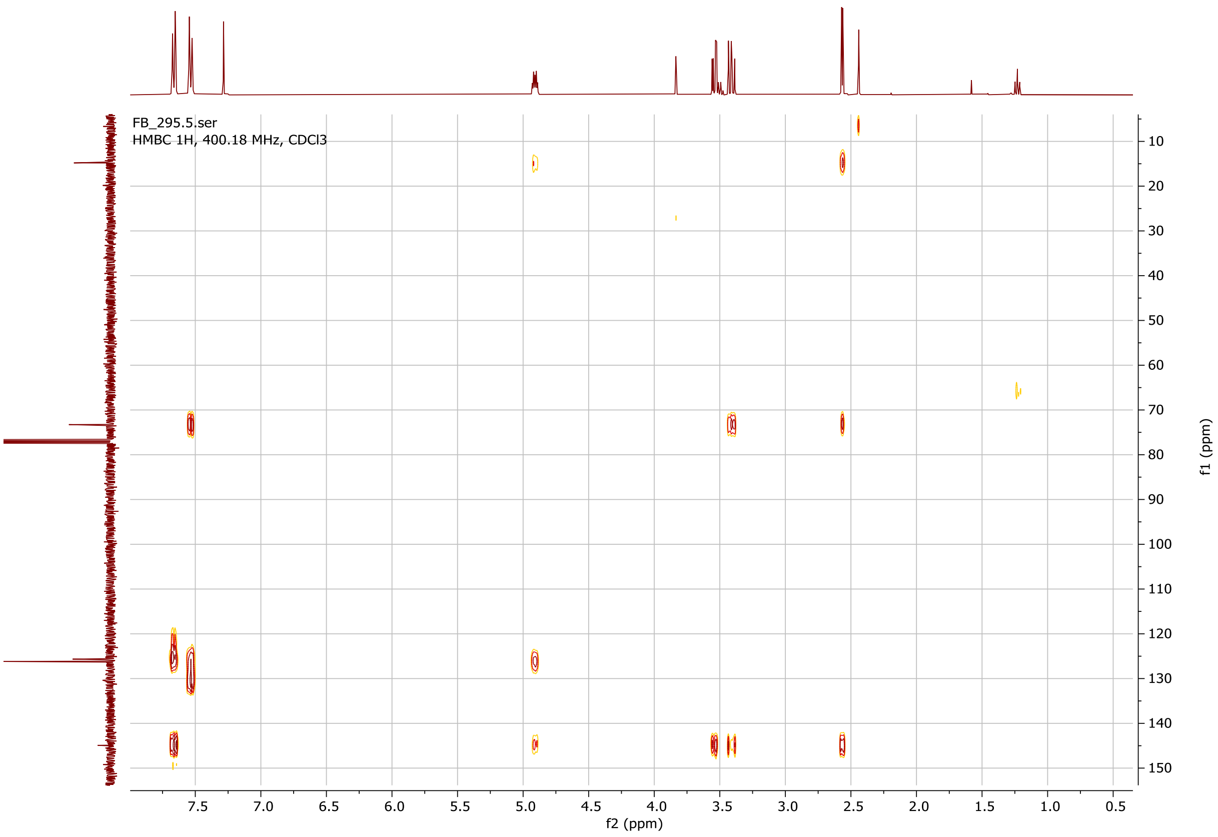
FB_295.5.ser
HMBC 1H, 400.18 MHz, CDCl3
f2 (ppm)
f1 (ppm)

1-Iodo-1-phenylpropan-2-ol **3q** *(inseparable 89:11 diastereomers mixture)*

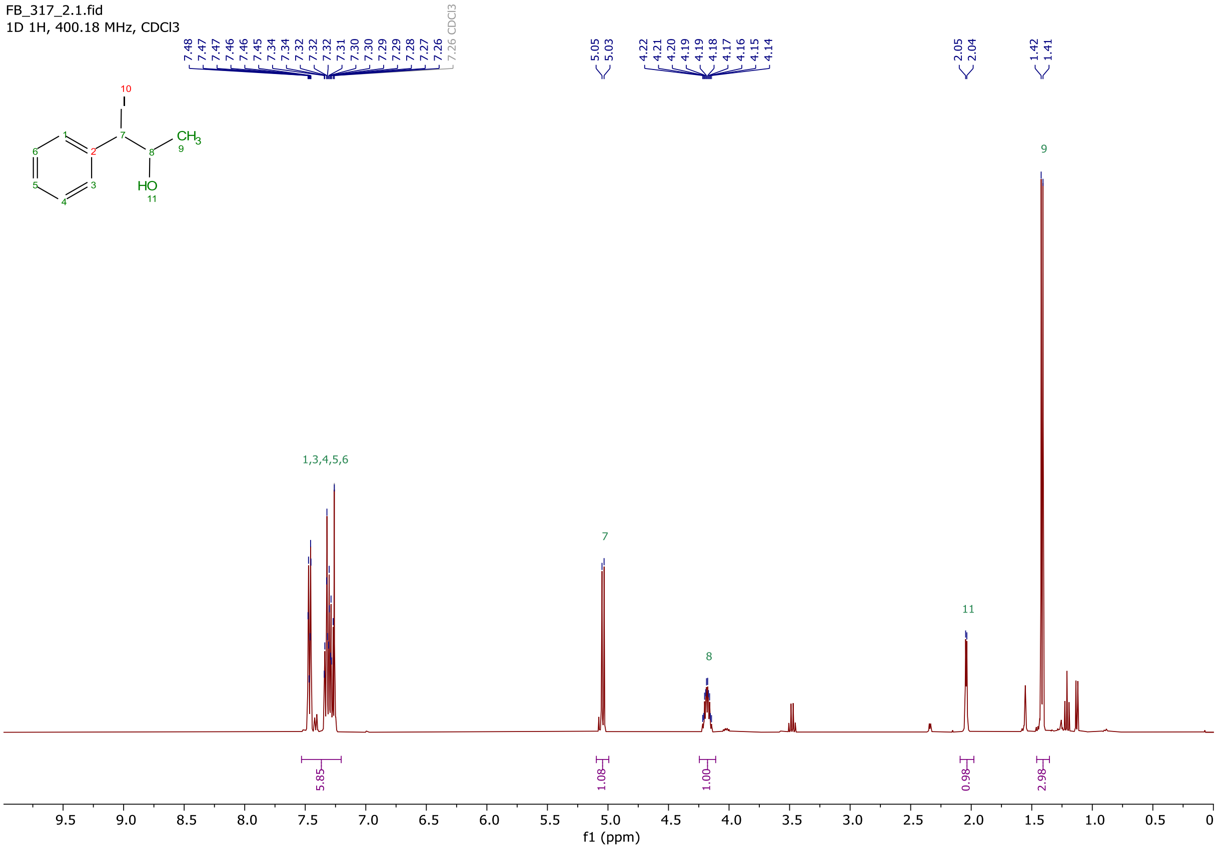

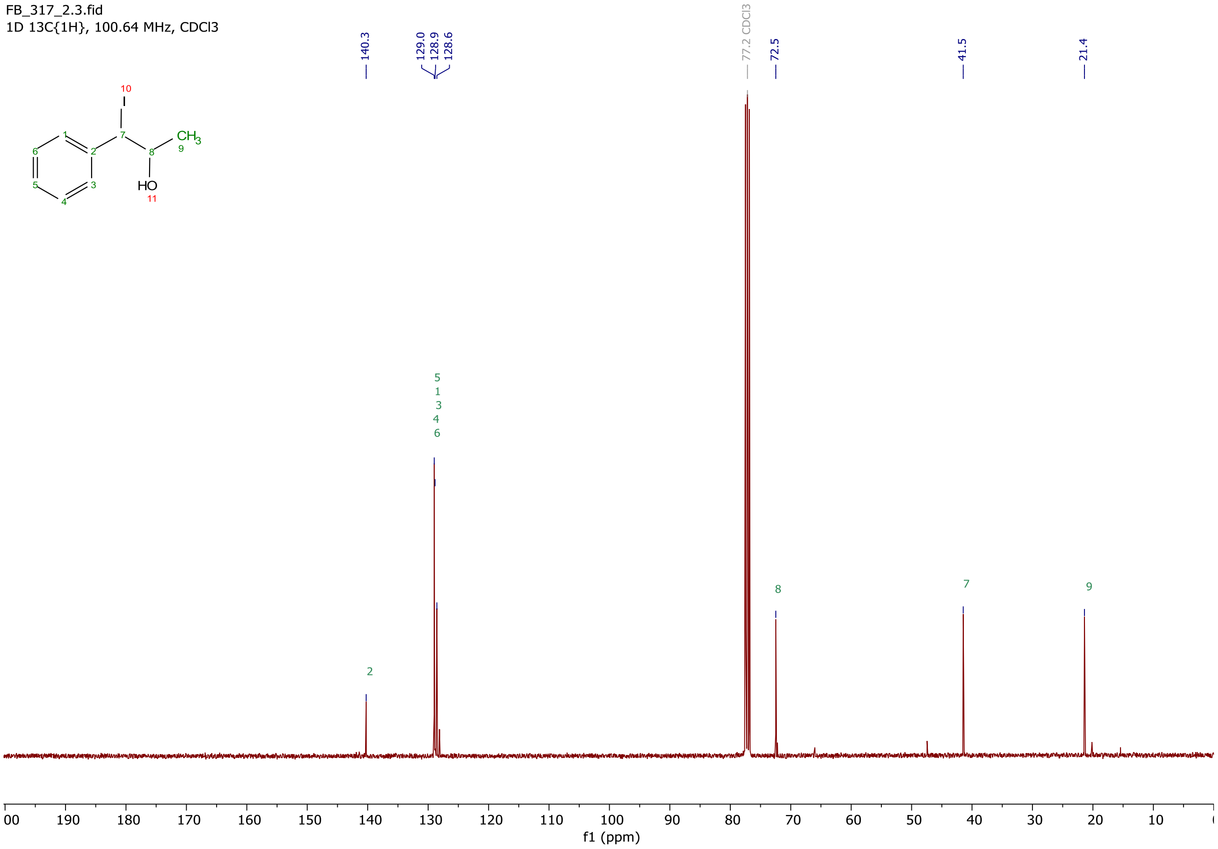

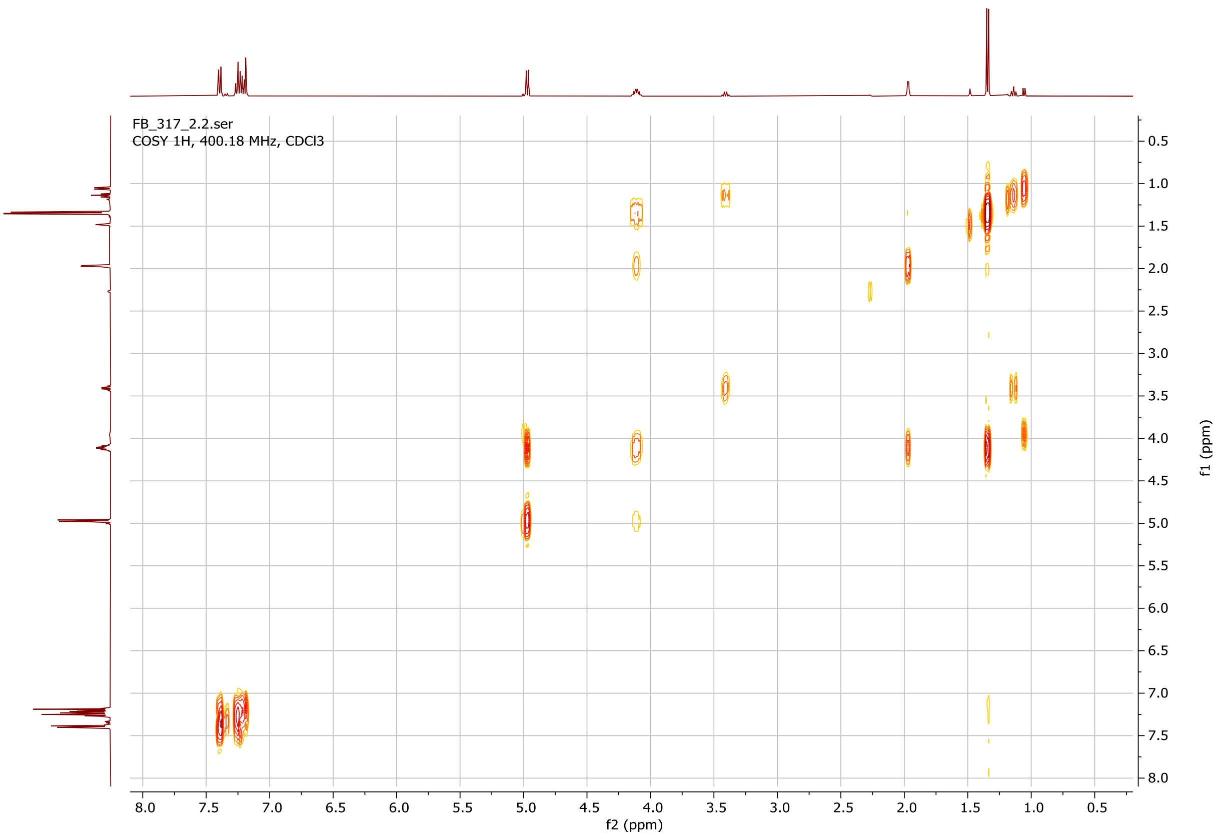
FB_317_2.2.ser
COSY 1H, 400.18 MHz, CDCl3
f2 (ppm)
f1 (ppm)

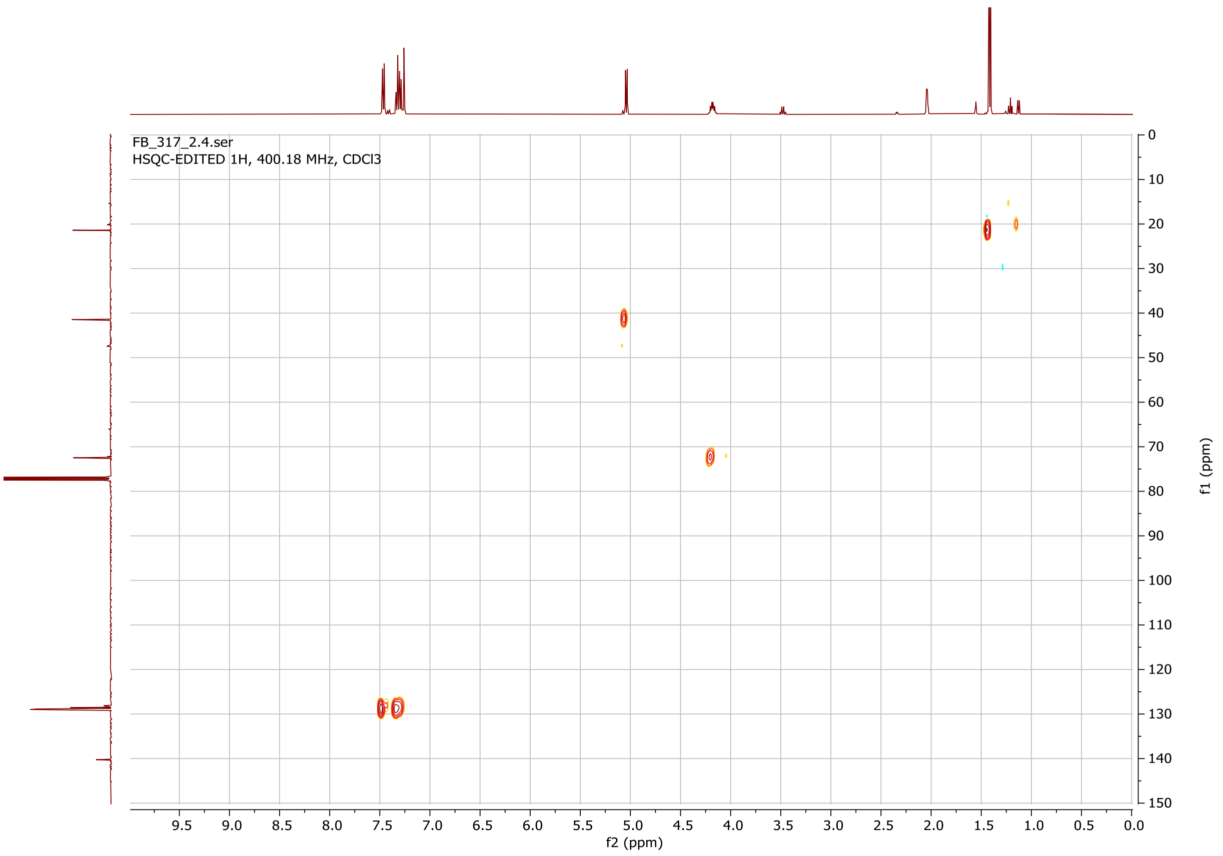
FB_317_2.4.ser
HSQC-EDITED 1H, 400.18 MHz, CDCl3
f2 (ppm)
f1 (ppm)

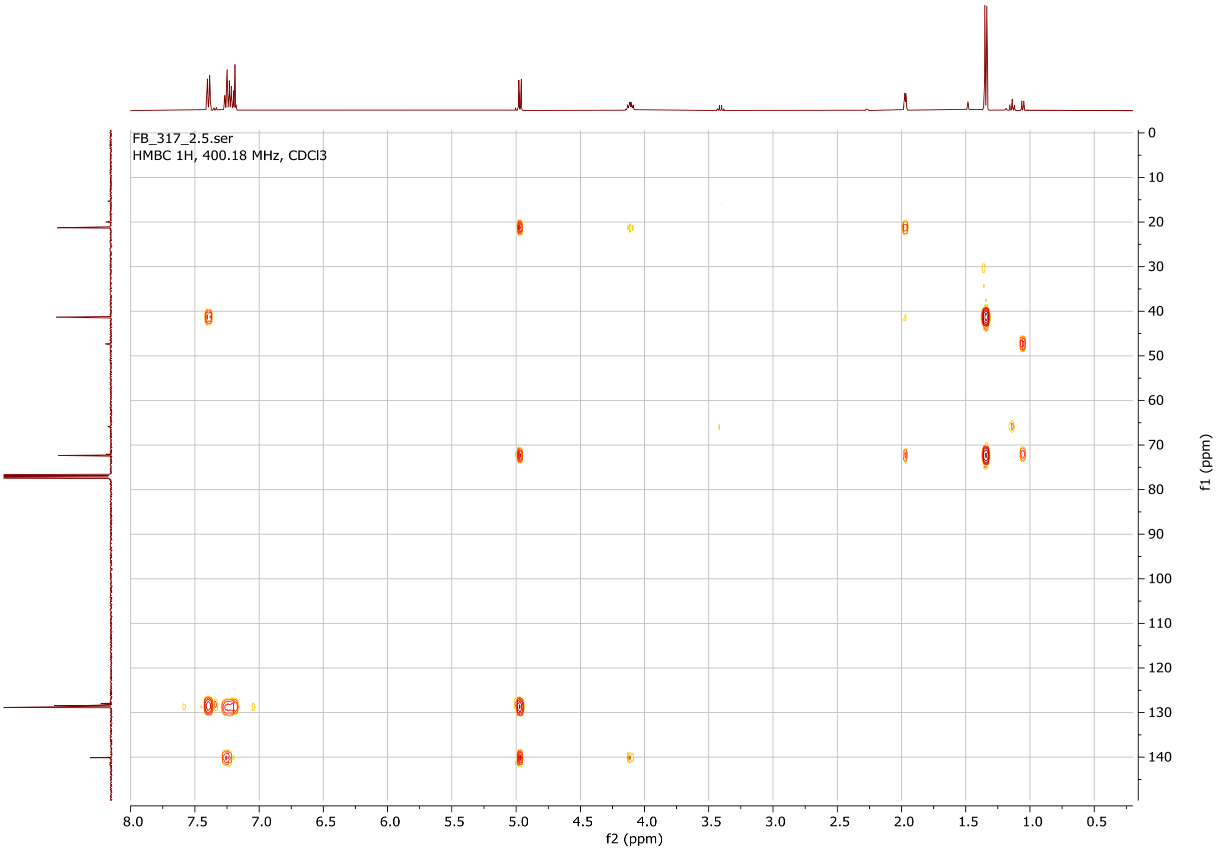

3-Hydroxy-4-iodo-4-phenylbutan-2-one **3r** *(inseparable 86:14 diastereomers mixture)*

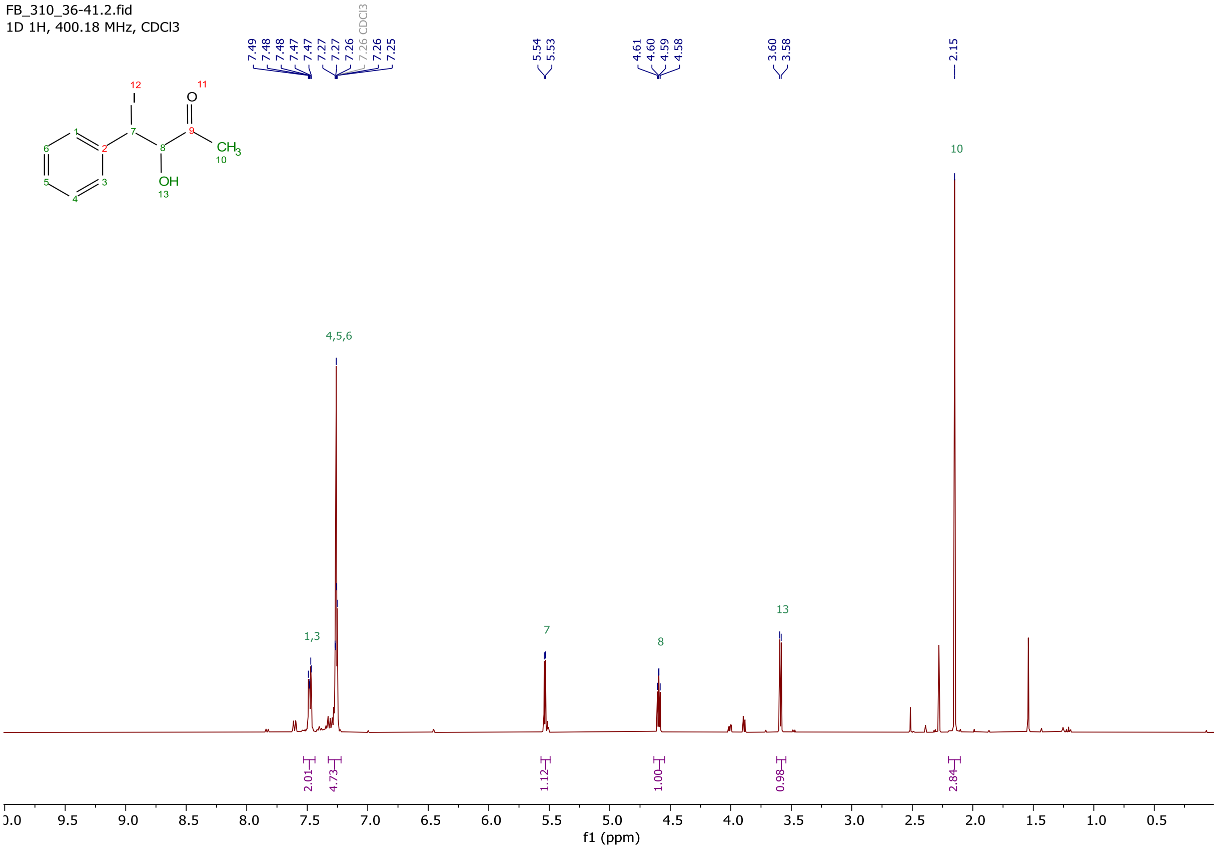

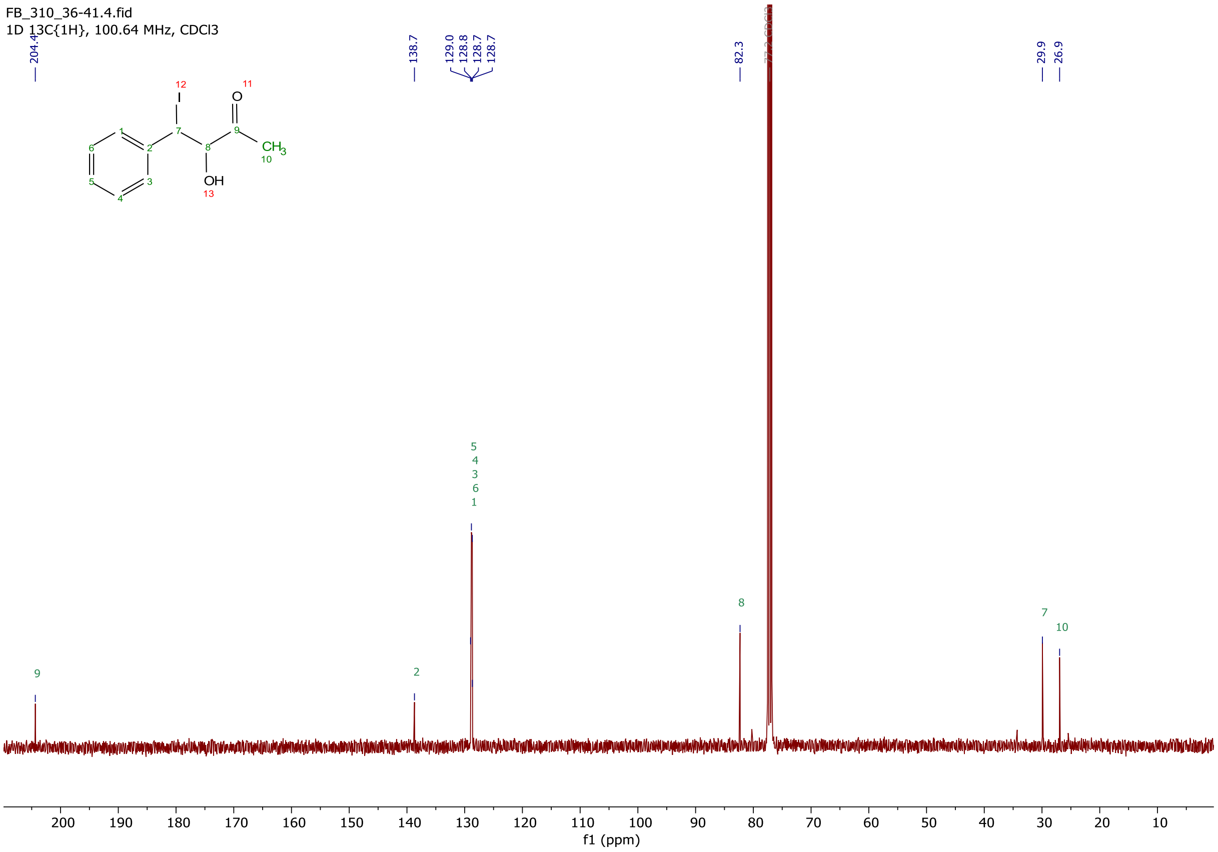

FB_310_36-41.4.fid
1D 13C{1H}, 100.64 MHz, CDCl3
204.4
138.7
129.0
128.8
128.7
128.7
82.3
29.9
26.9
f1 (ppm)

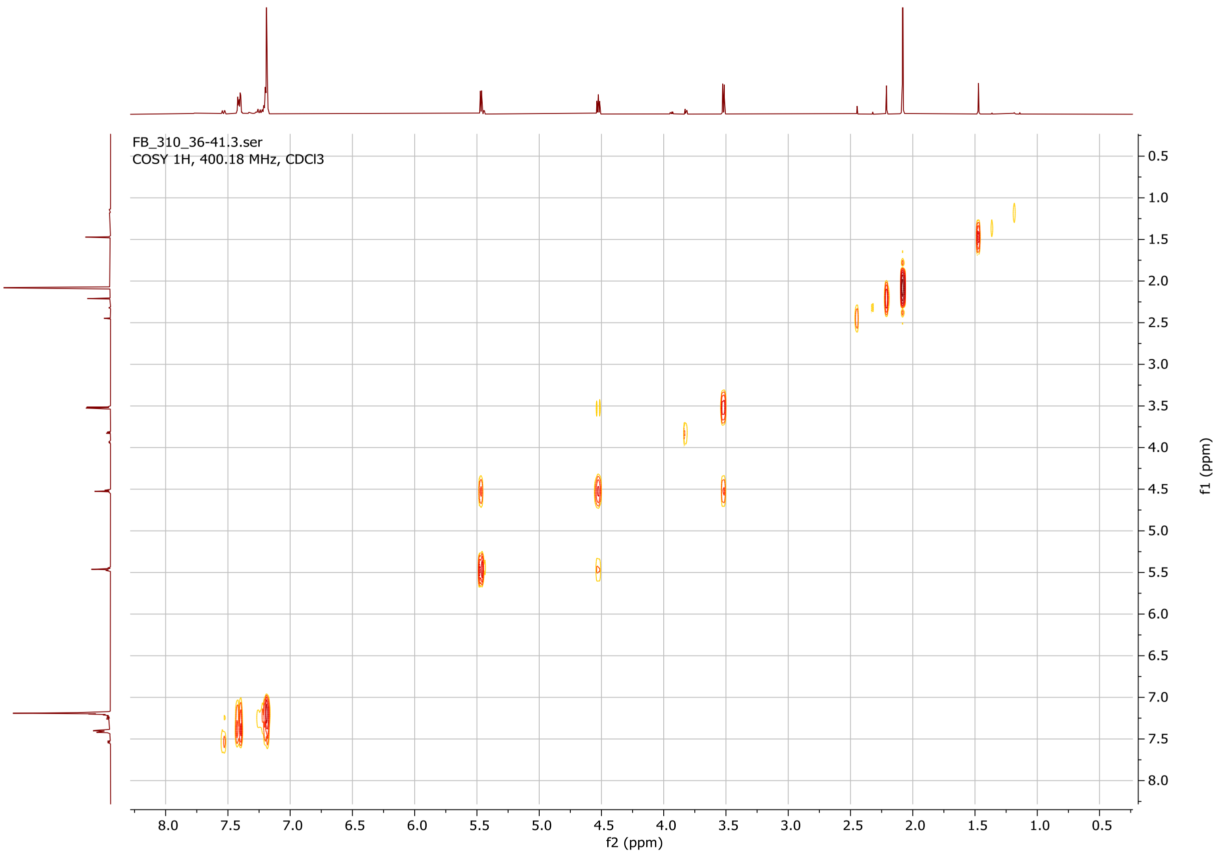

FB_310_36-41.3.ser
COSY 1H, 400.18 MHz, CDCl3
f2 (ppm)
f1 (ppm)

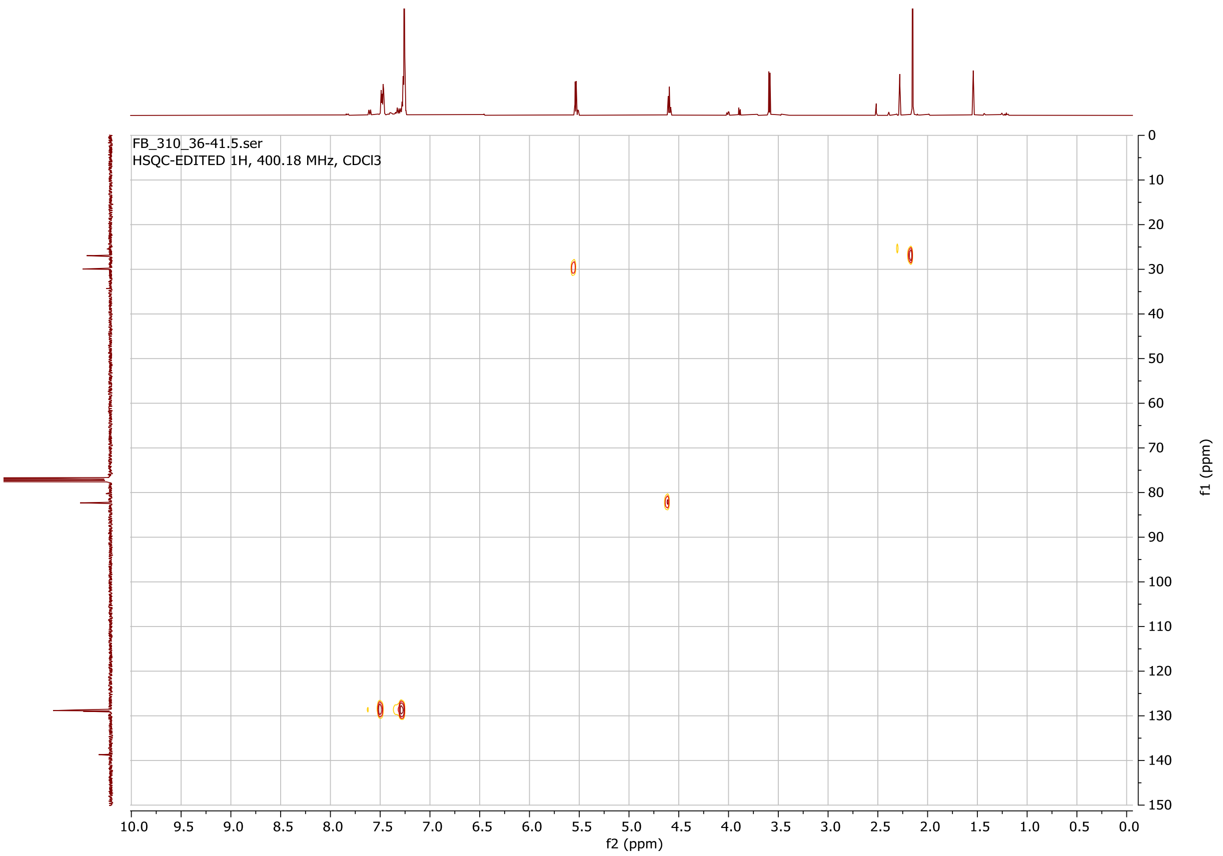
FB_310_36-41.5.ser
HSQC-EDITED 1H, 400.18 MHz, CDCl3
f2 (ppm)
f1 (ppm)

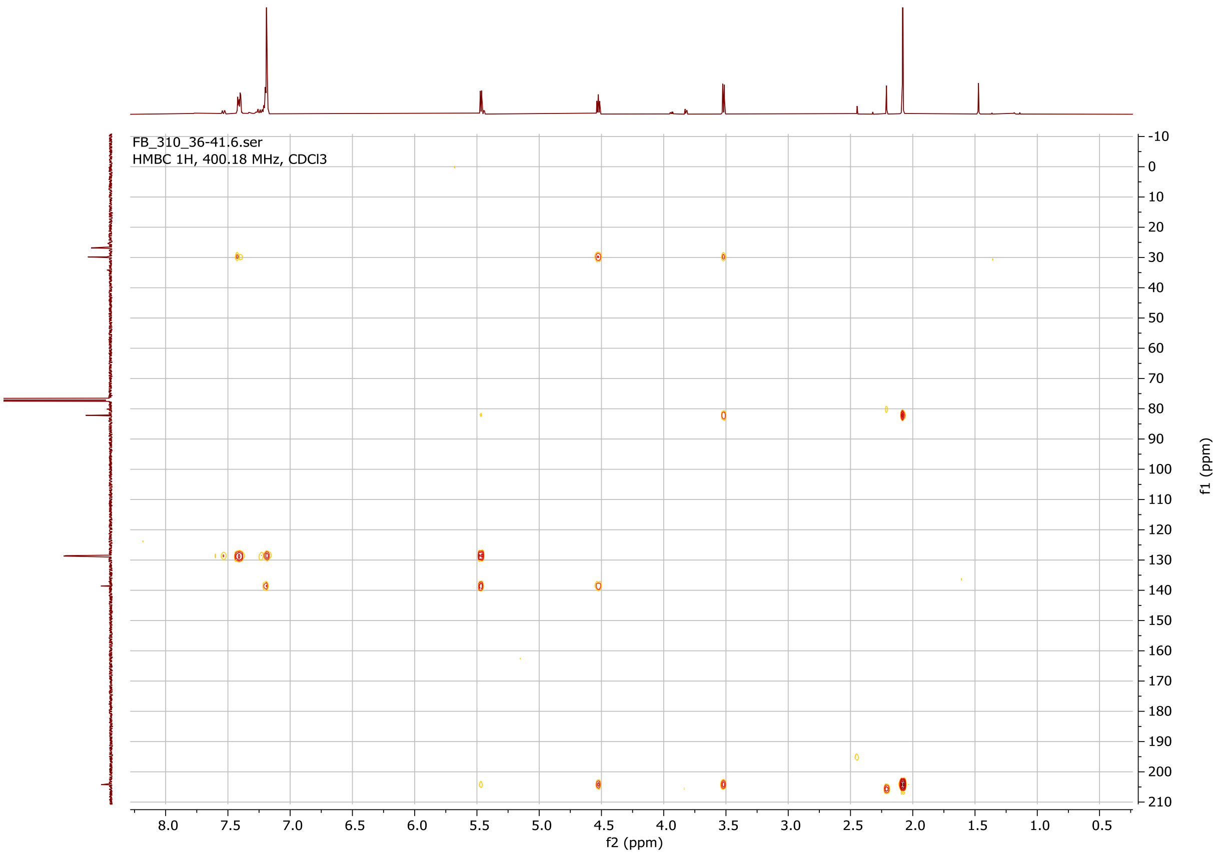
FB_310_36-41.6.ser
HMBC 1H, 400.18 MHz, CDCl3
f2 (ppm)
f1 (ppm)

1-Iodo-3-isopropoxy-1-phenylpropan-2-ol **3s** *(inseparable 64:36 diastereomers mixture)*

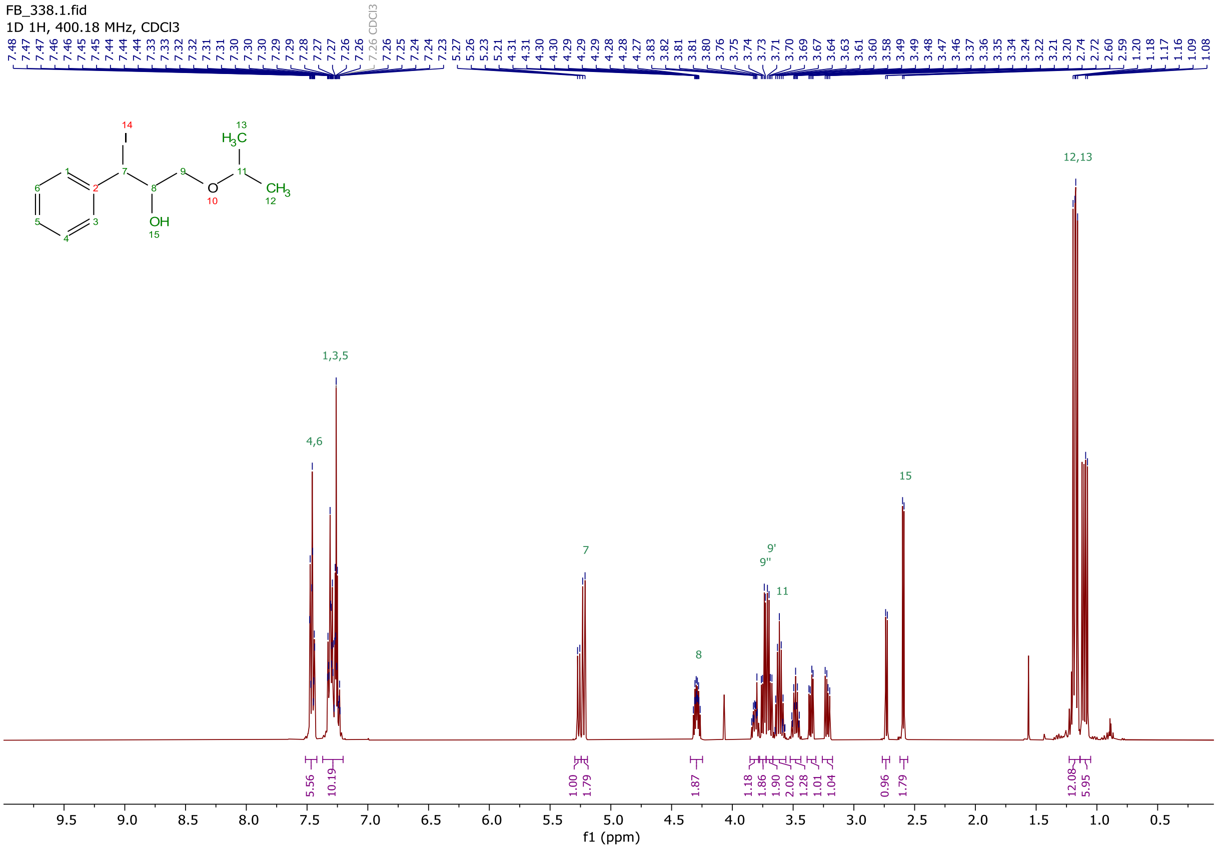

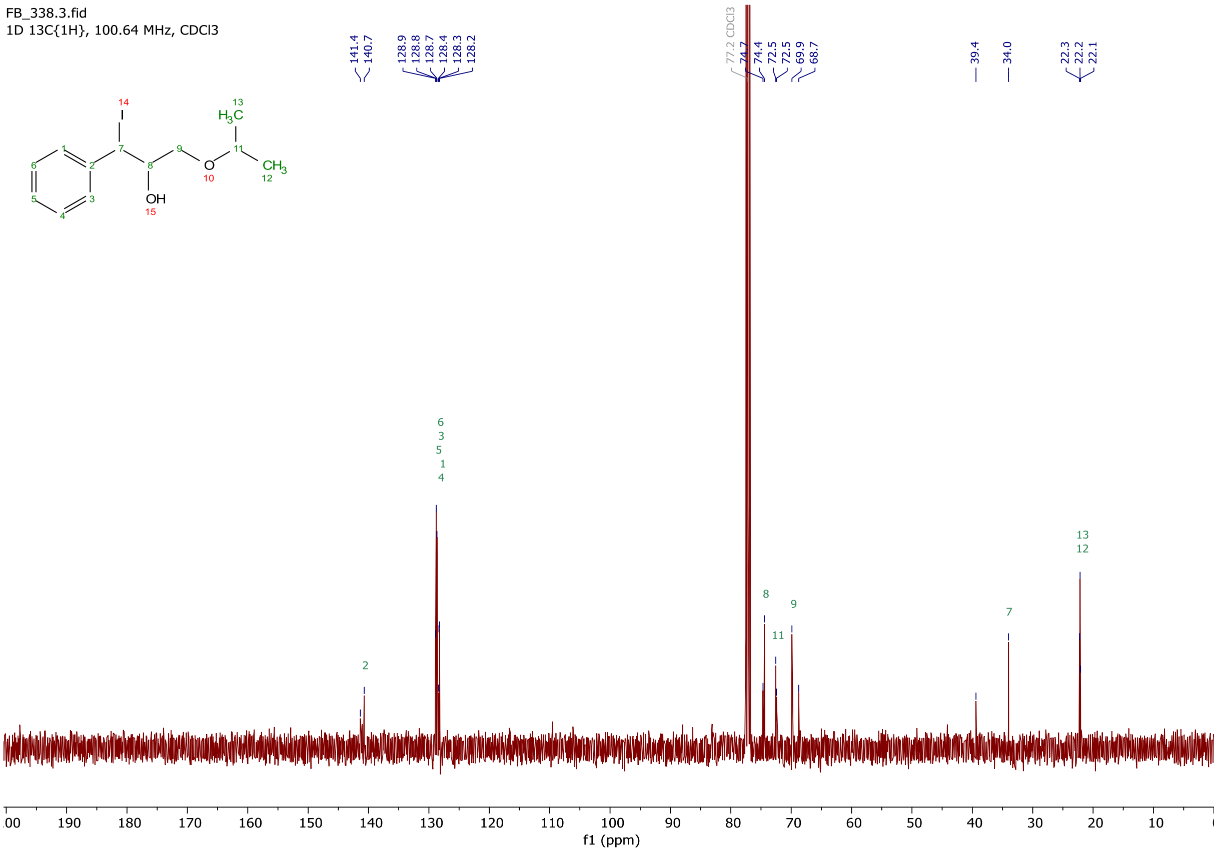

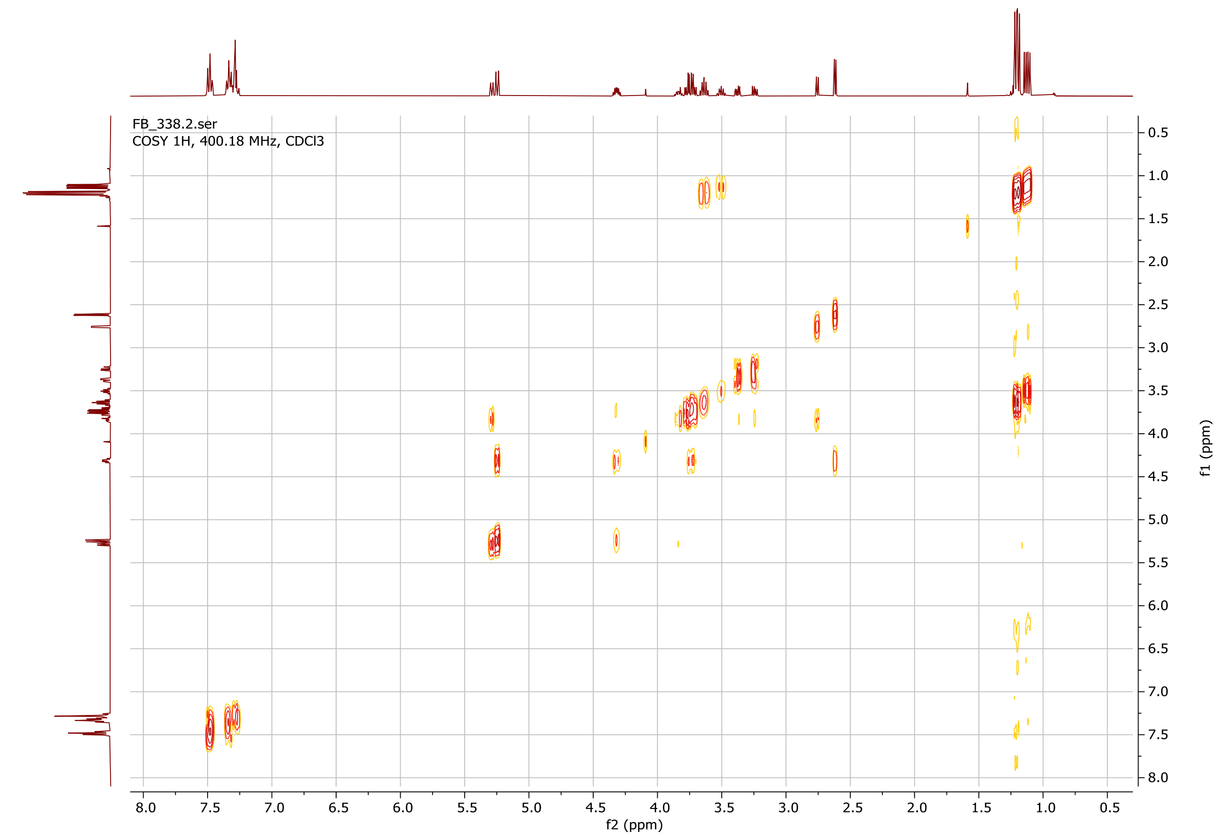
FB_338.2.ser
COSY 1H, 400.18 MHz, CDCl3
f1 (ppm)
f2 (ppm)

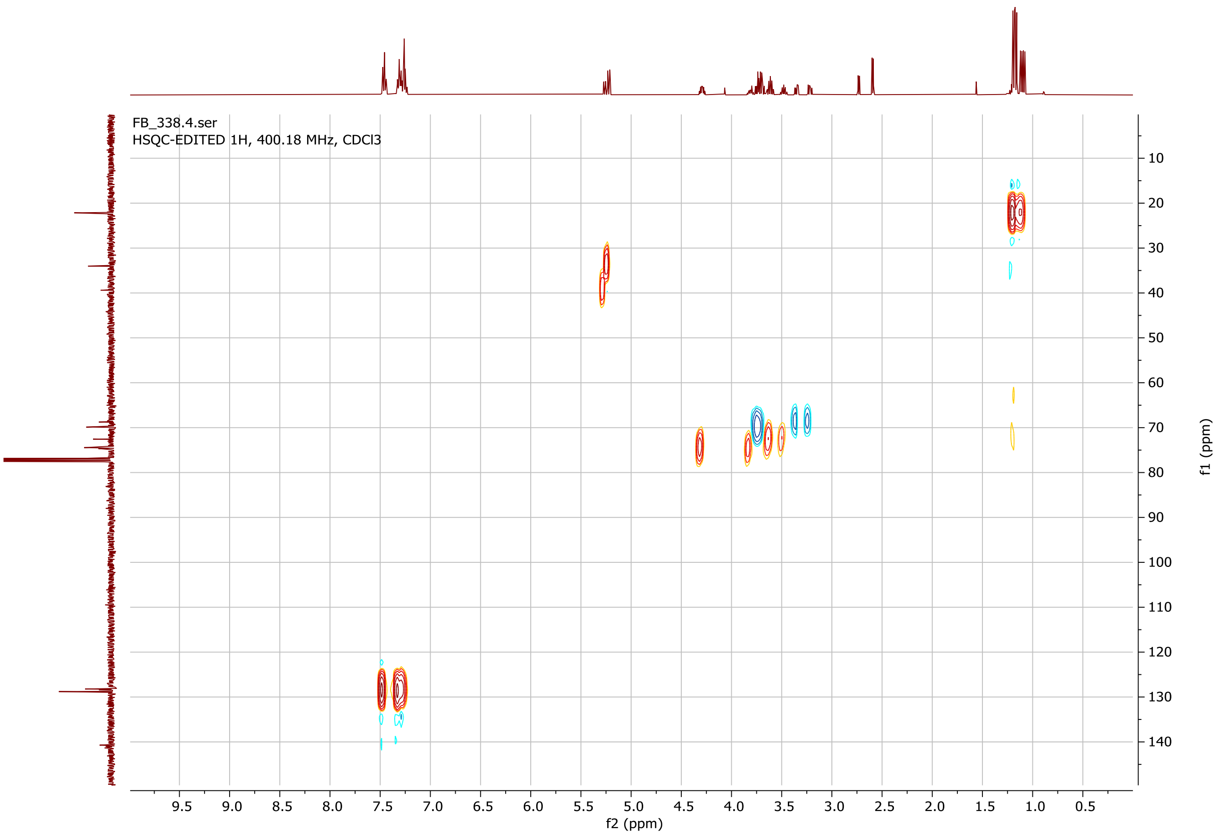
FB_338.4.ser
HSQC-EDITED 1H, 400.18 MHz, CDCl3
f1 (ppm)
f2 (ppm)

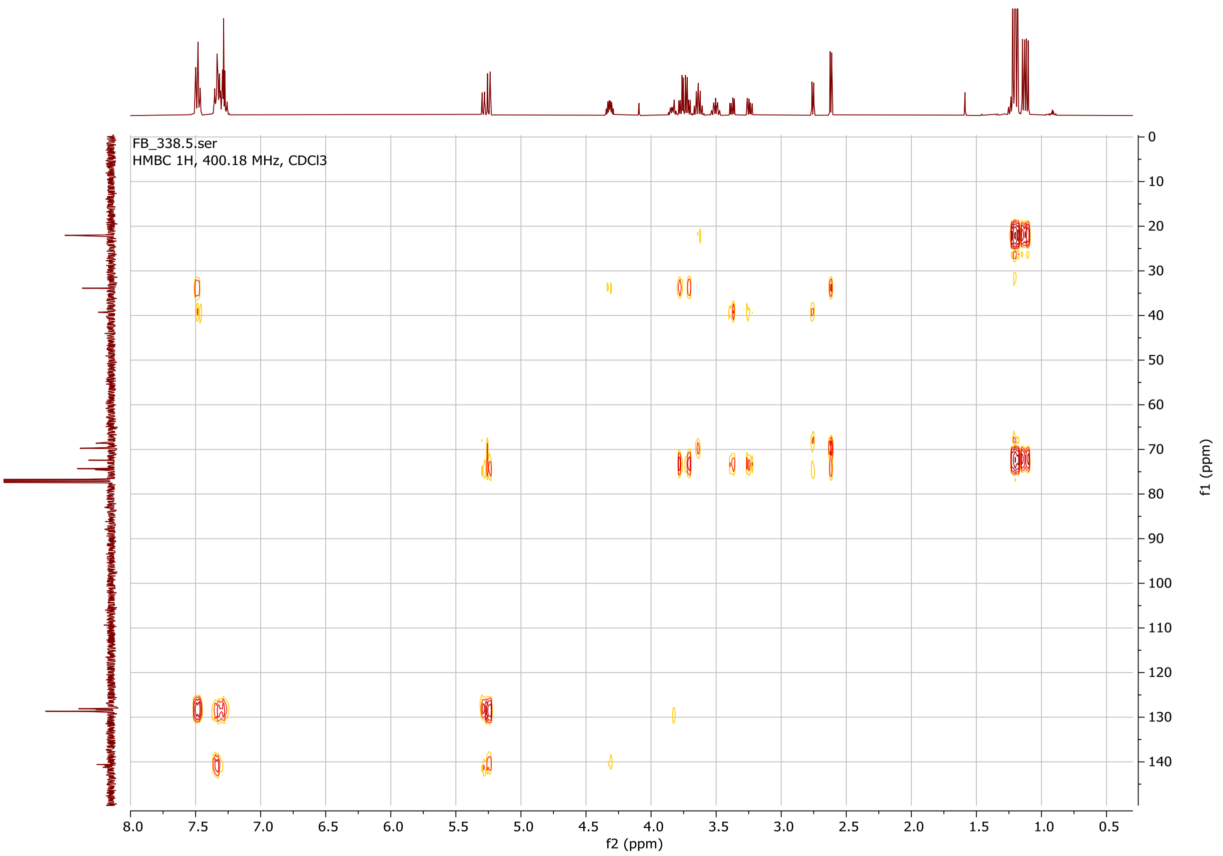

Methyl 3-hydroxy-2-iodo-3-phenylpropanoate **3t**

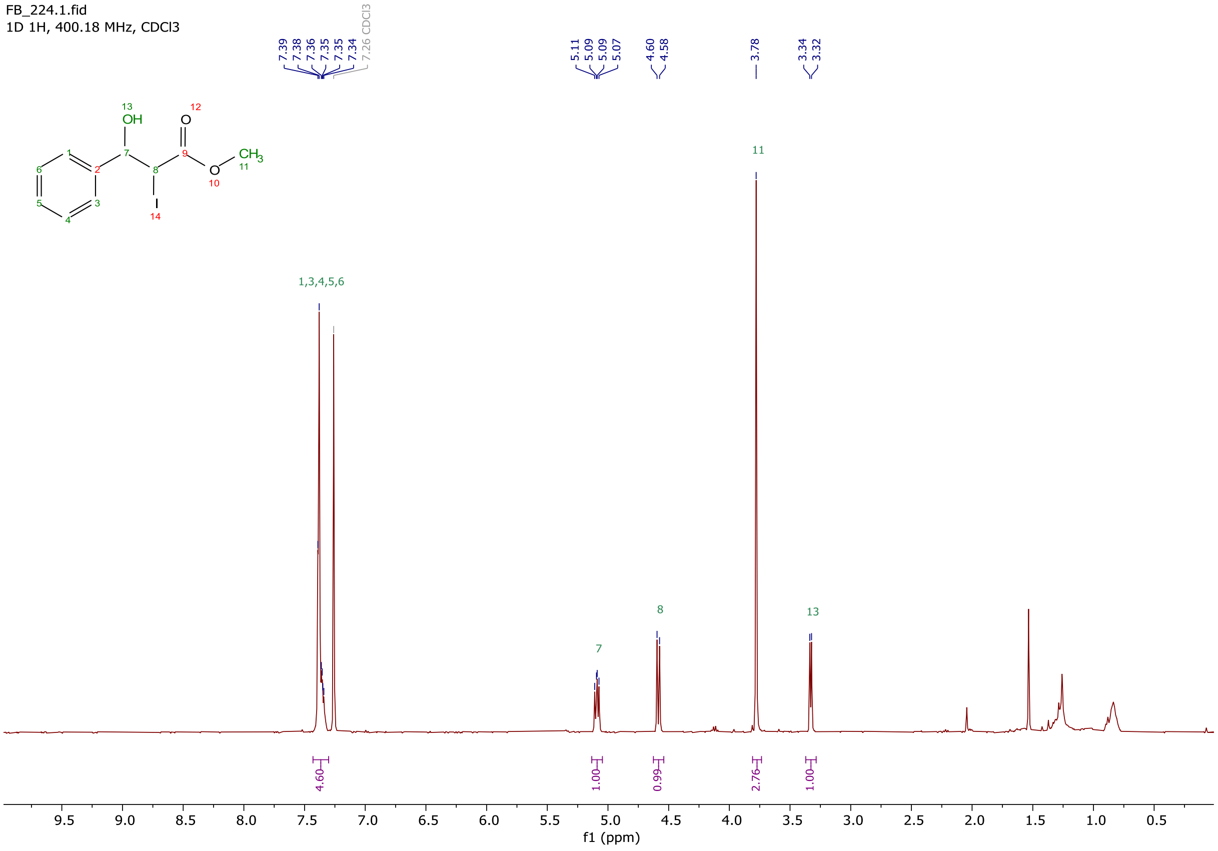

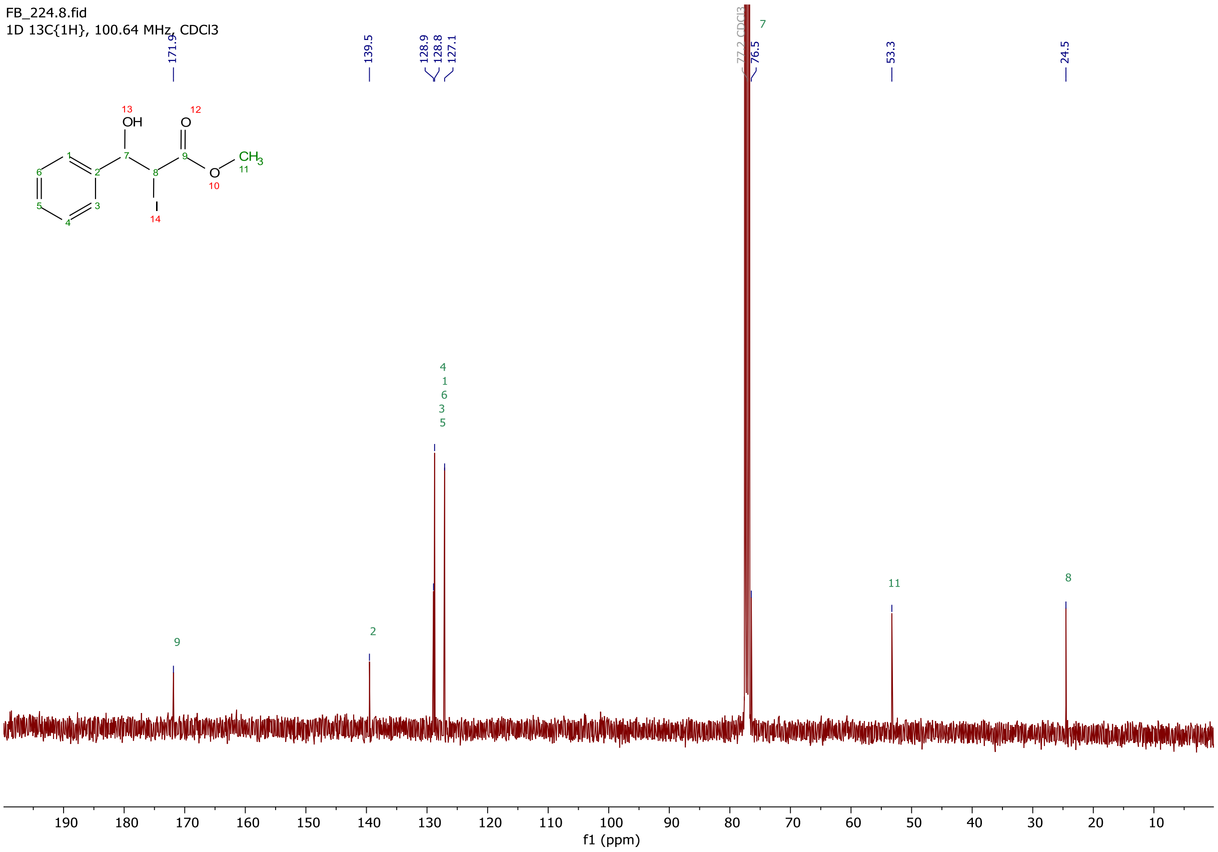
FB_224.8.fid
1D 13C{1H}, 100.64 MHz, CDCl3
171.9
139.5
128.9
128.8
127.1
77.2 CDCl3
76.5
53.3
24.5
OH
O
CH3
I
9
2
4
1
6
3
5
7
11
8
f1 (ppm)

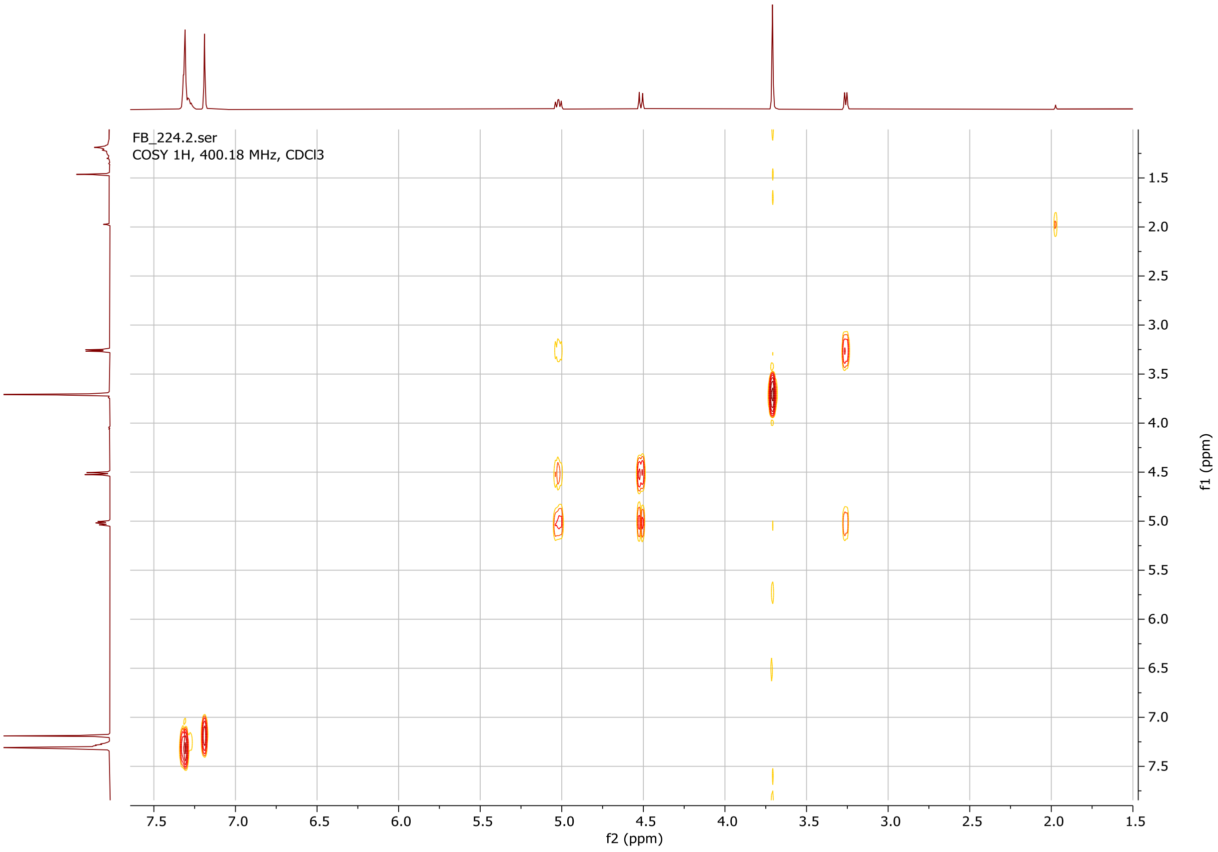
FB_224.2.ser
COSY 1H, 400.18 MHz, CDCl3
f2 (ppm)
f1 (ppm)

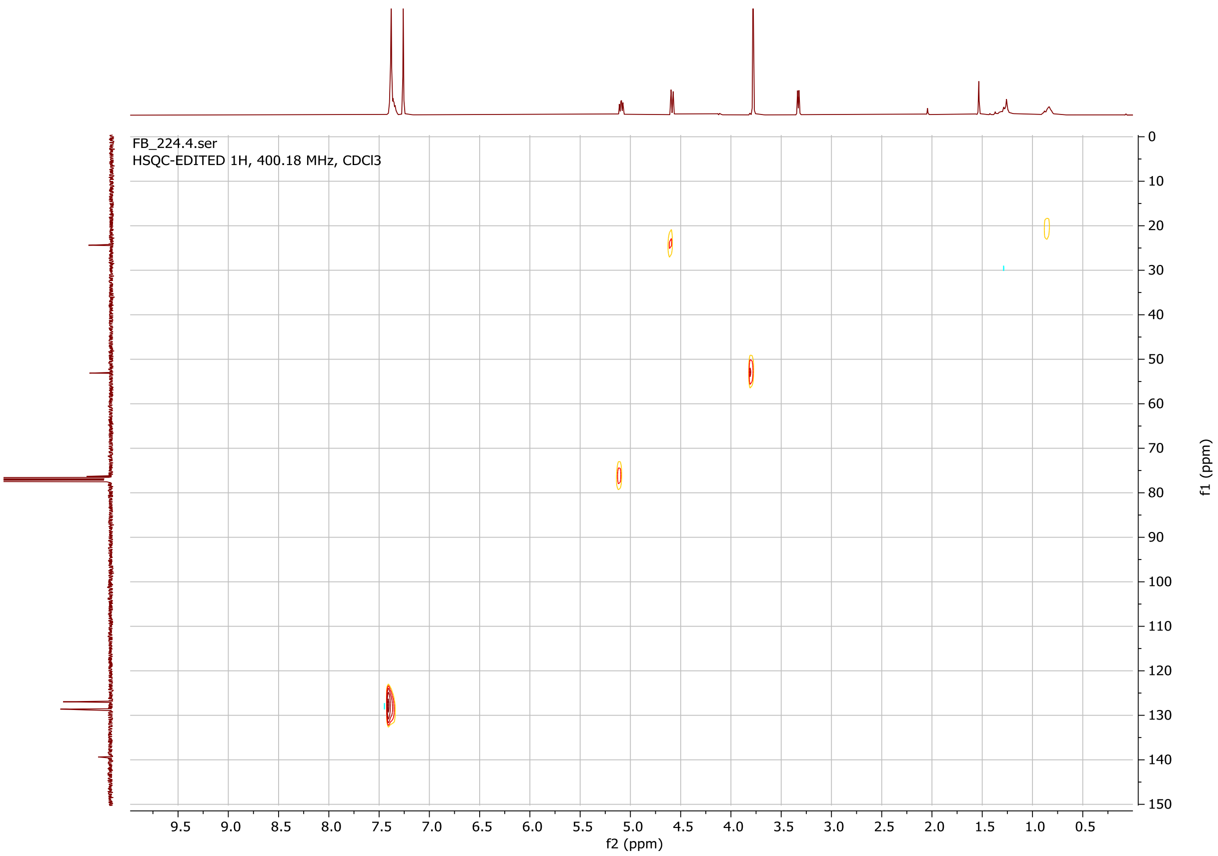
FB_224.4.ser
HSQC-EDITED 1H, 400.18 MHz, CDCl3
f2 (ppm)
f1 (ppm)

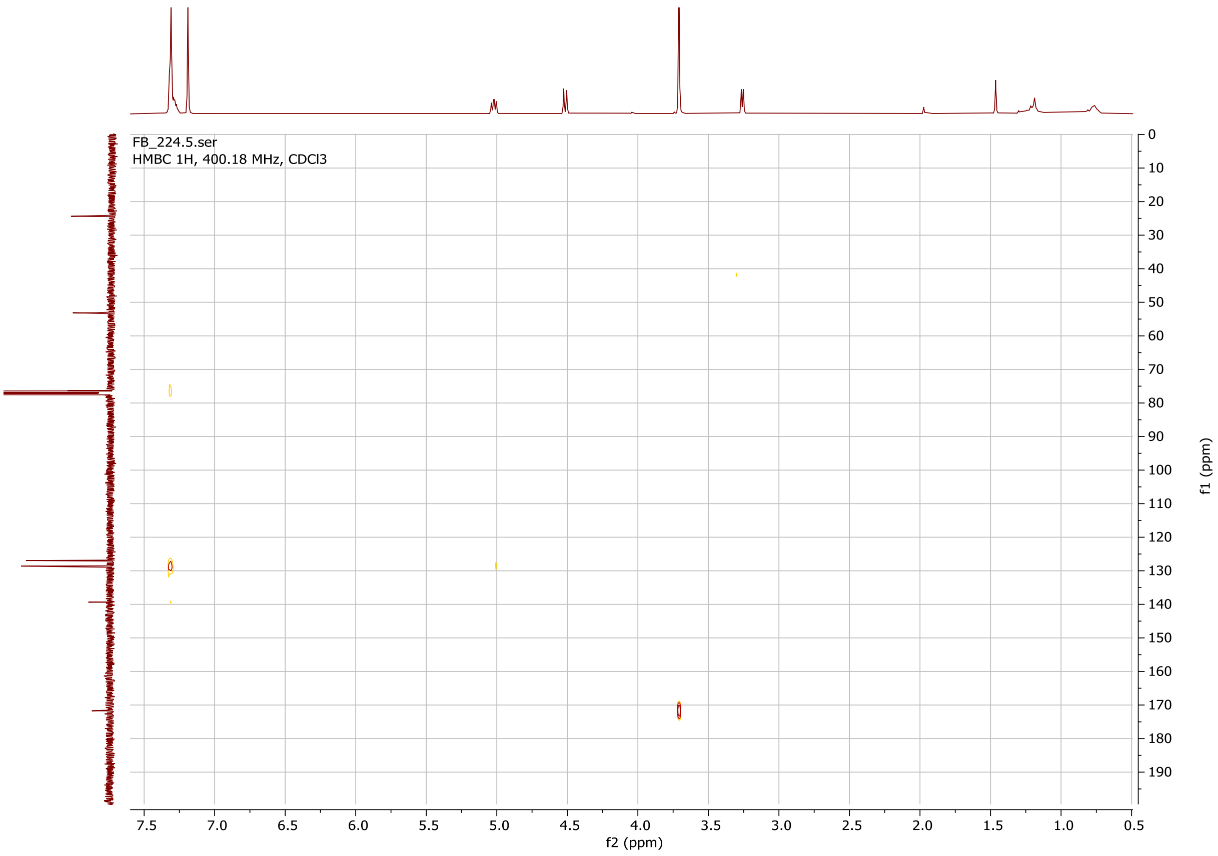
FB_224.5.ser
HMBC 1H, 400.18 MHz, CDCl3
f2 (ppm)
f1 (ppm)

3-Hydroxy-2-iodo-*N*,*N*-dimethyl-3-phenylpropanamide **3u**

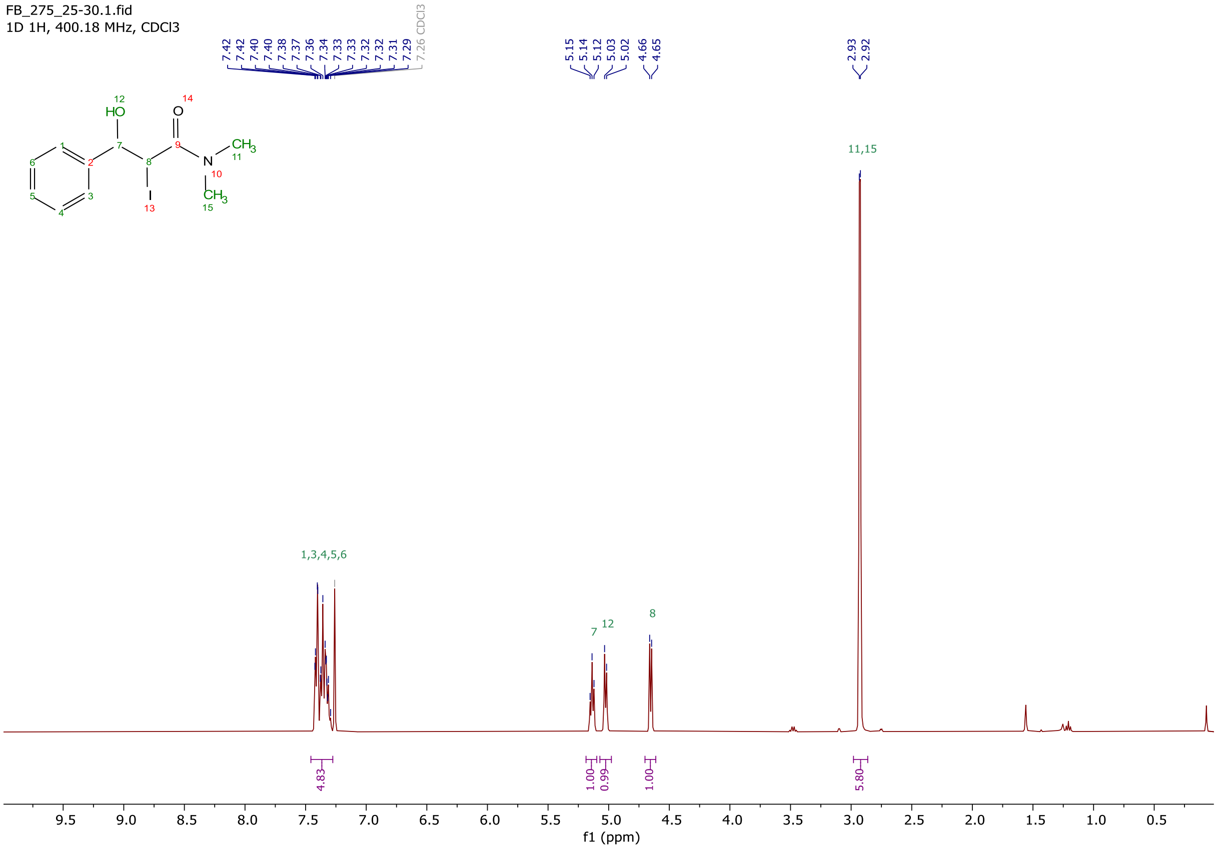

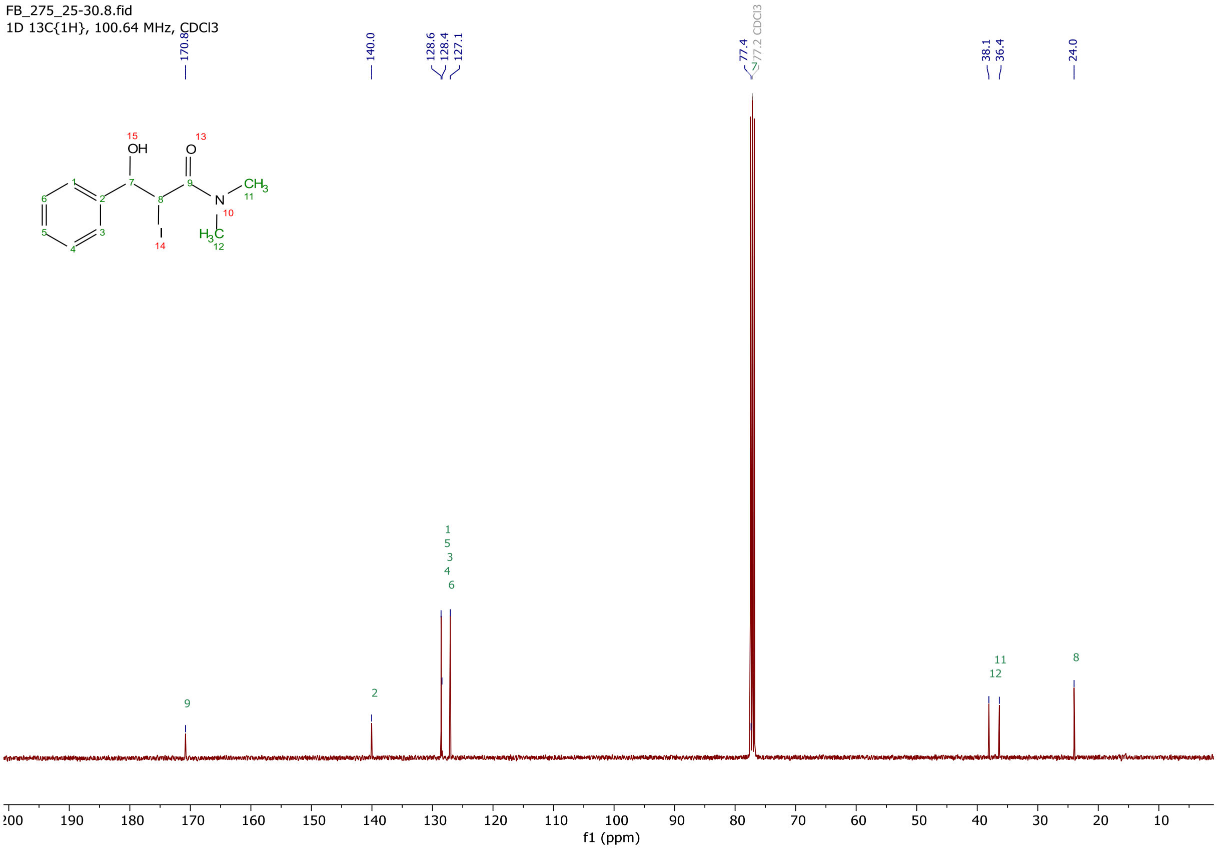

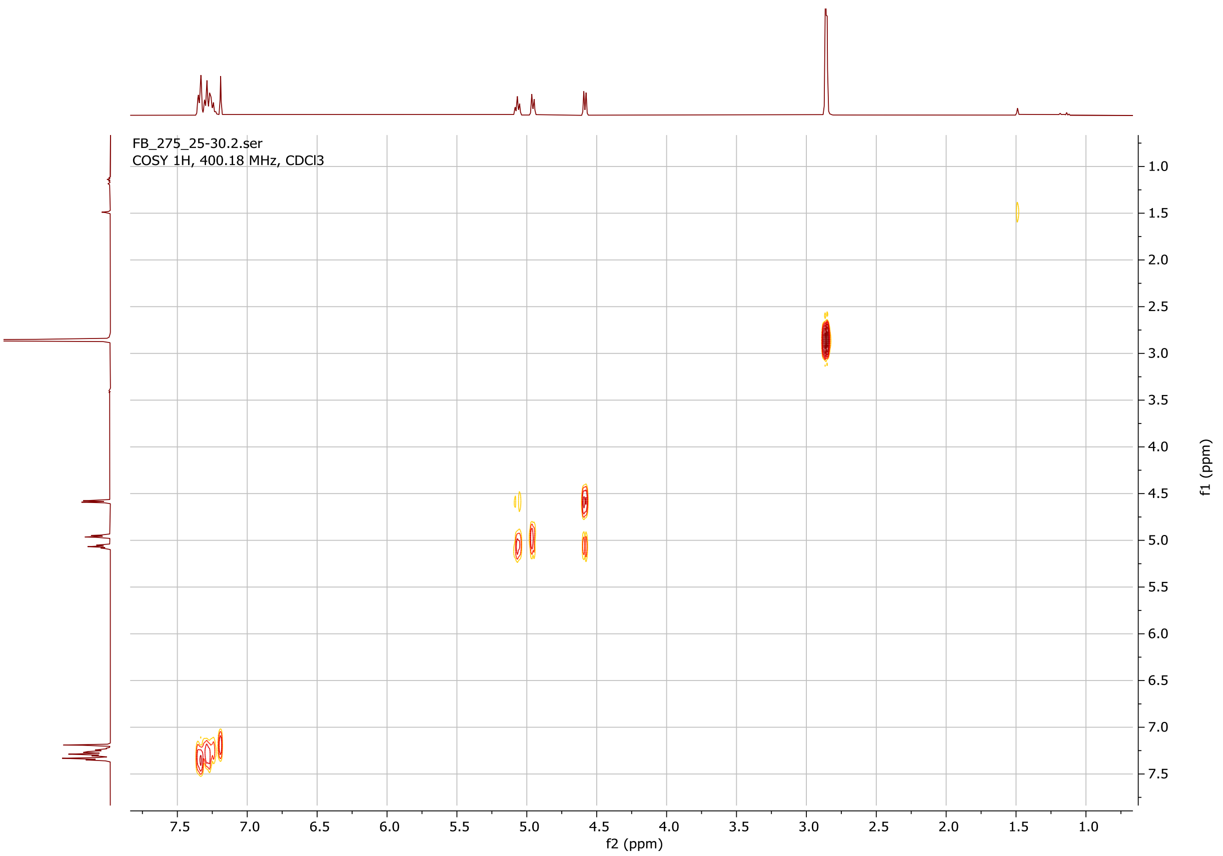
FB_275_25-30.2.ser
COSY 1H, 400.18 MHz, CDCl3
f2 (ppm)
f1 (ppm)

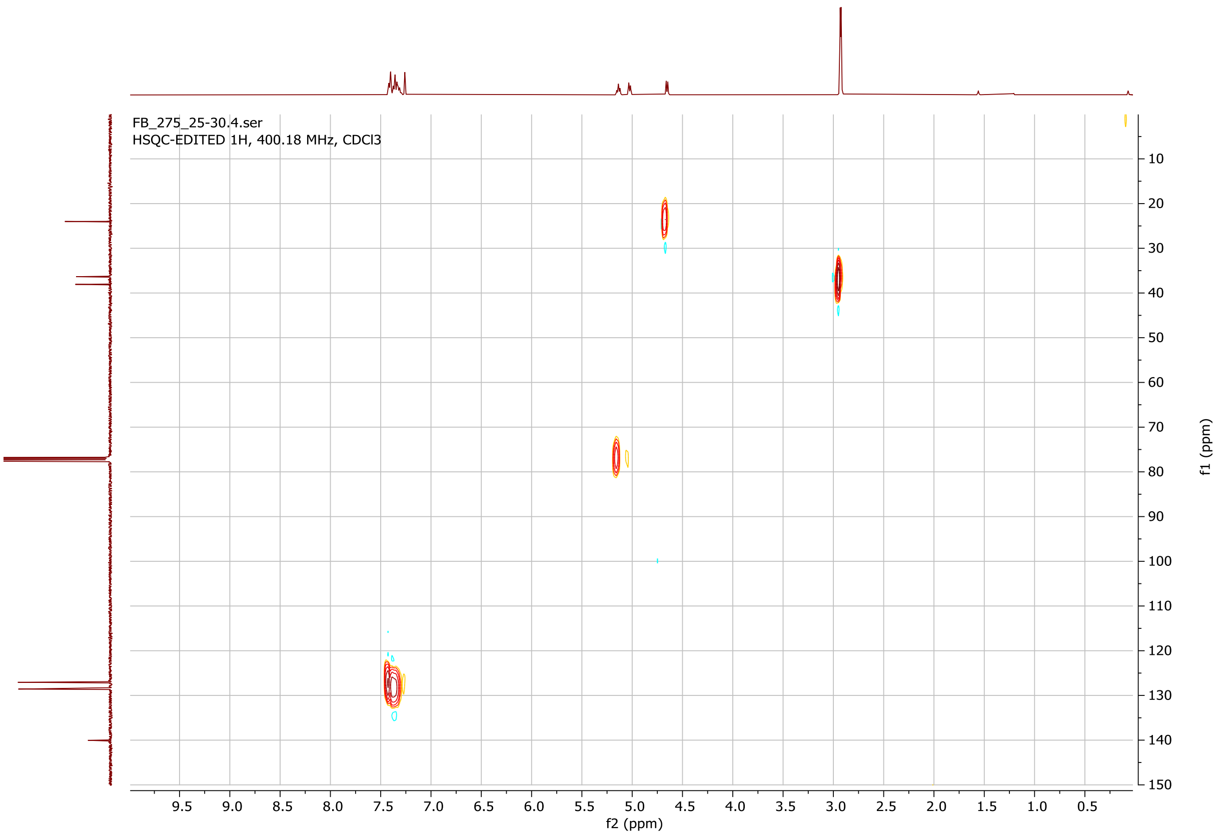
FB_275_25-30.4.ser
HSQC-EDITED 1H, 400.18 MHz, CDCl3
f2 (ppm)
f1 (ppm)

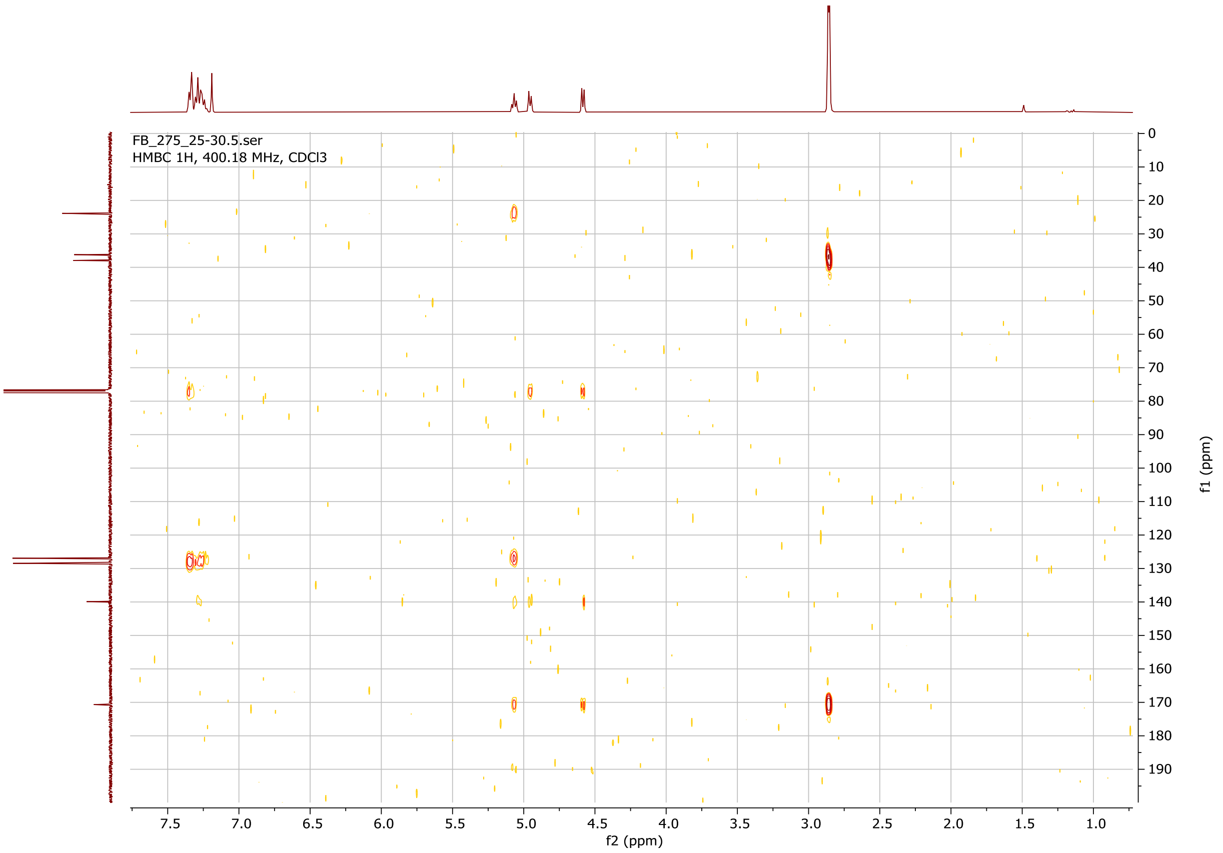

3-Hydroxy-2-iodocyclohexan-1-one **3v** *(Et_2O peaks visible at 3.48 and 1.21 ppm)*

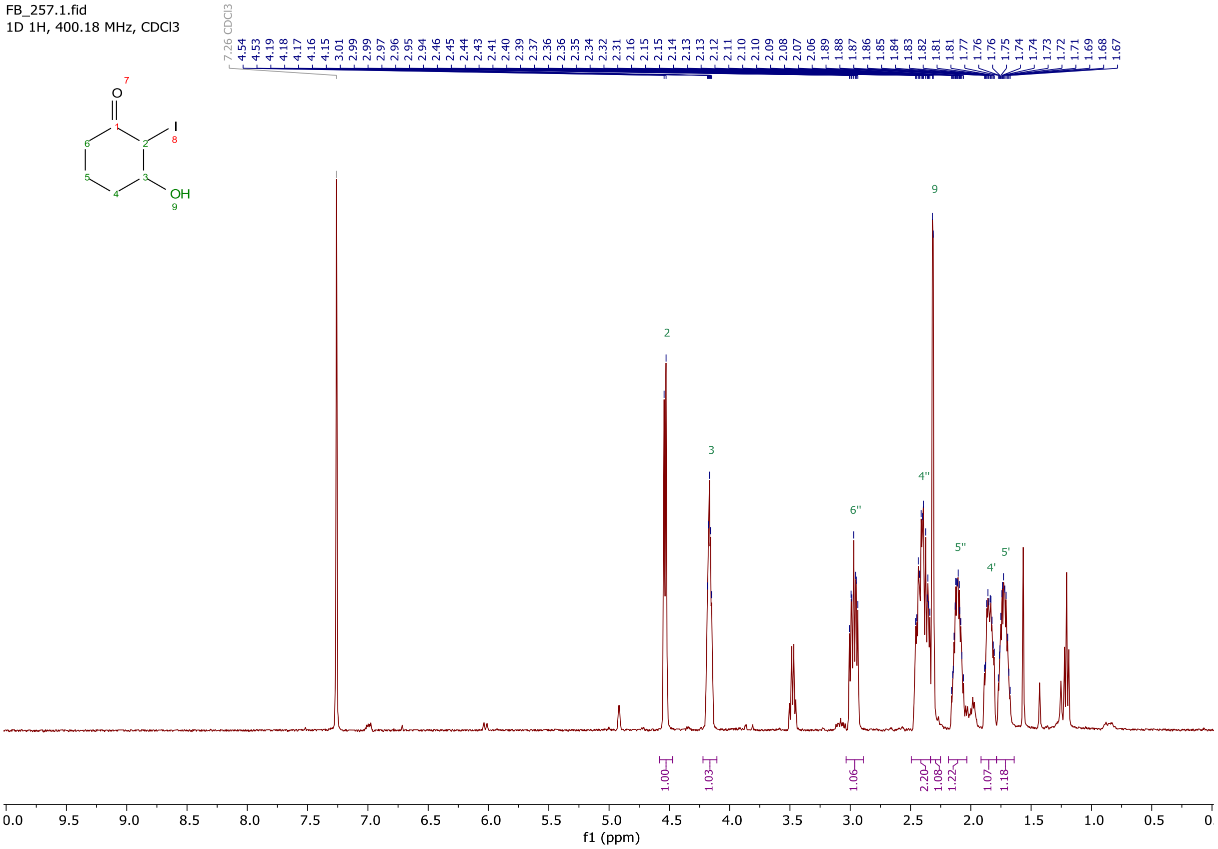

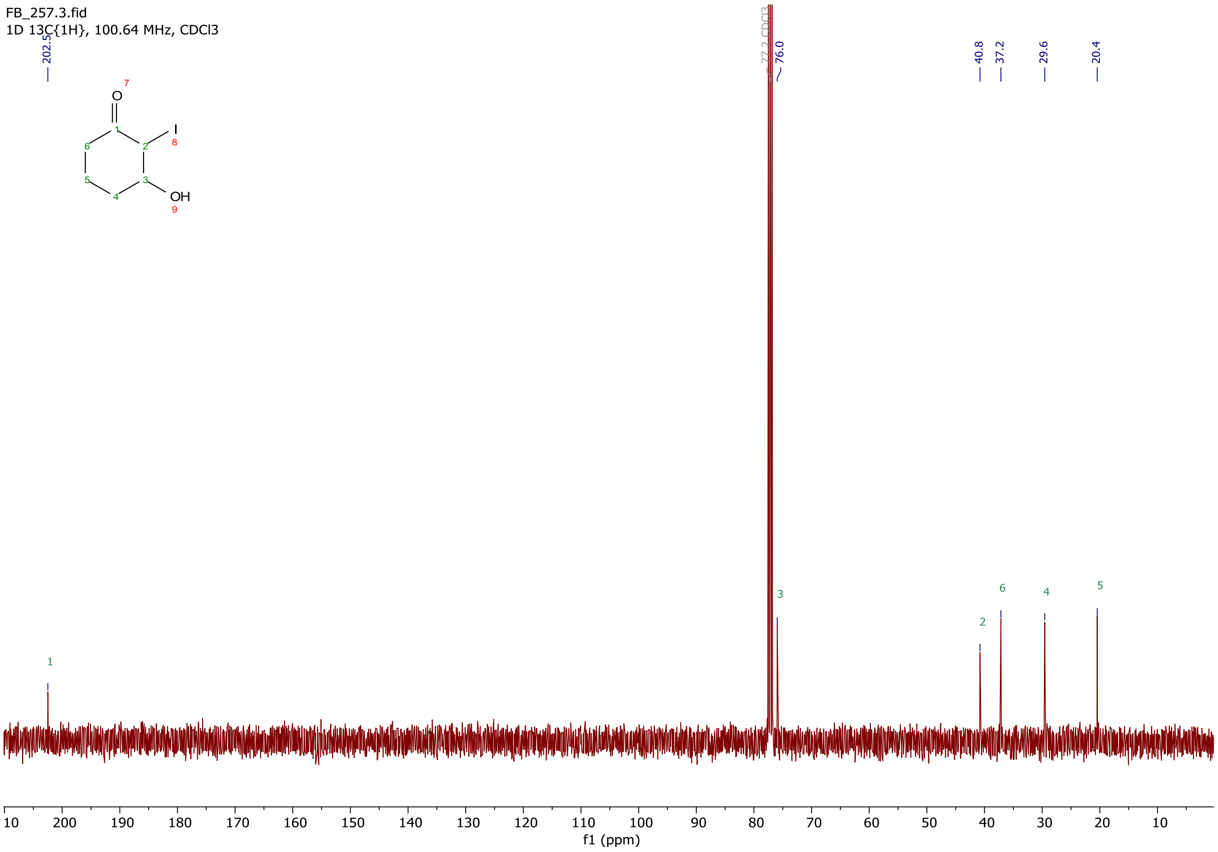
FB_257.3.fid
1D 13C{1H}, 100.64 MHz, CDCl3
202.5
77.2 CDCl3
76.0
40.8
37.2
29.6
20.4
O
I
OH
f1 (ppm)

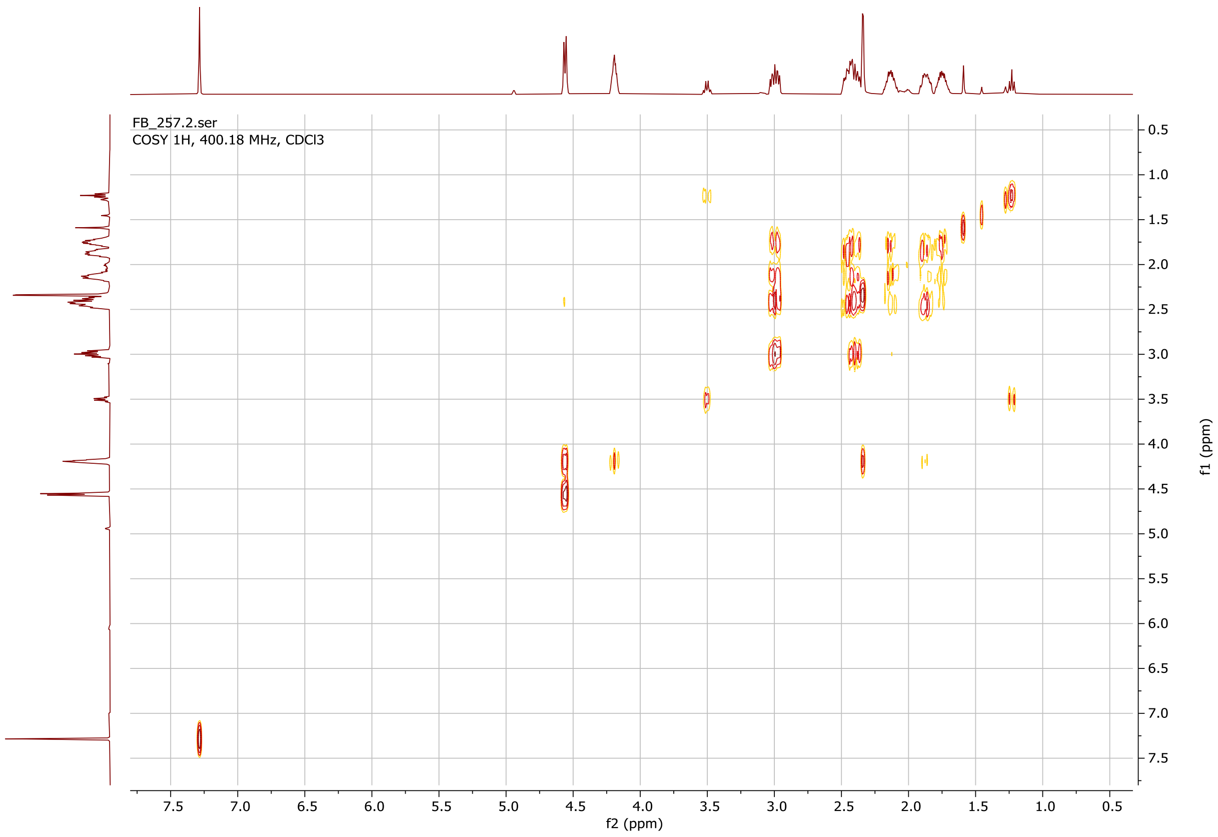
FB_257.2.ser
COSY 1H, 400.18 MHz, CDCl3
f2 (ppm)
f1 (ppm)

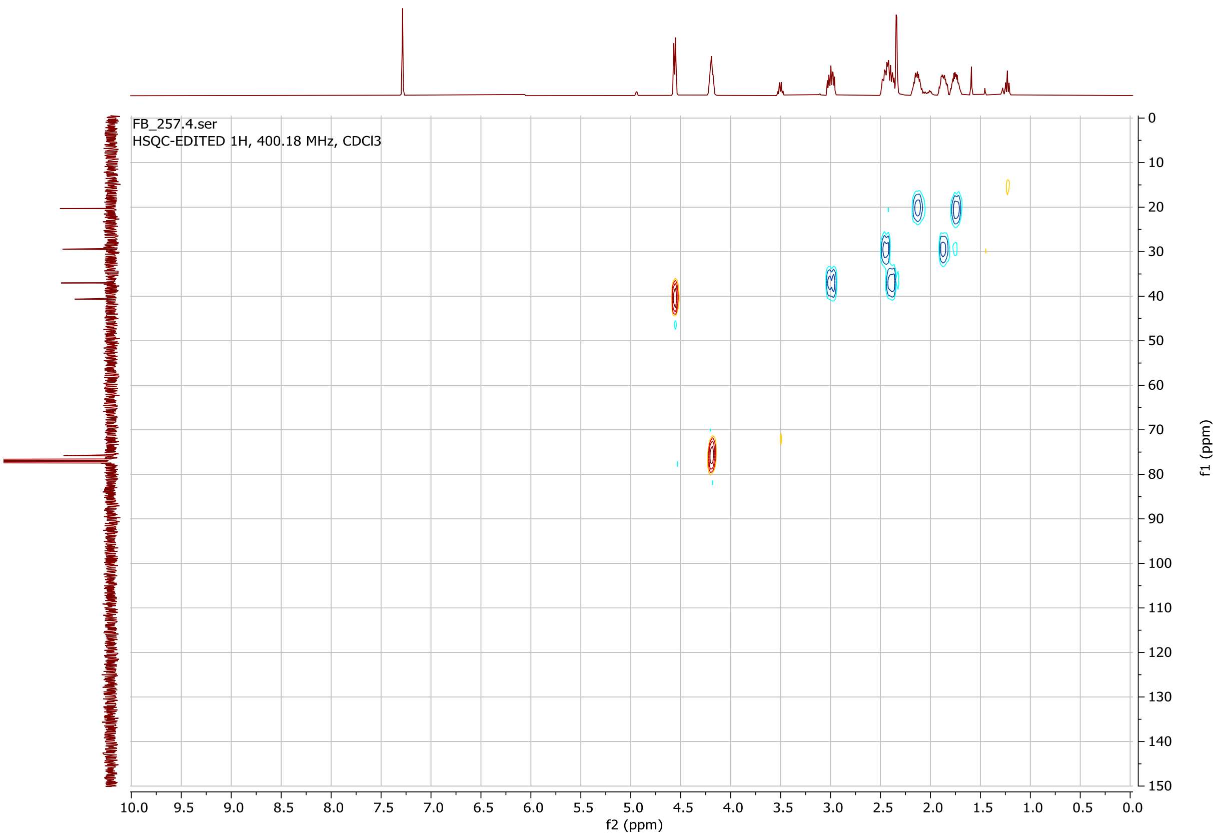
FB_257.4.ser
HSQC-EDITED 1H, 400.18 MHz, CDCl3
f2 (ppm)
f1 (ppm)

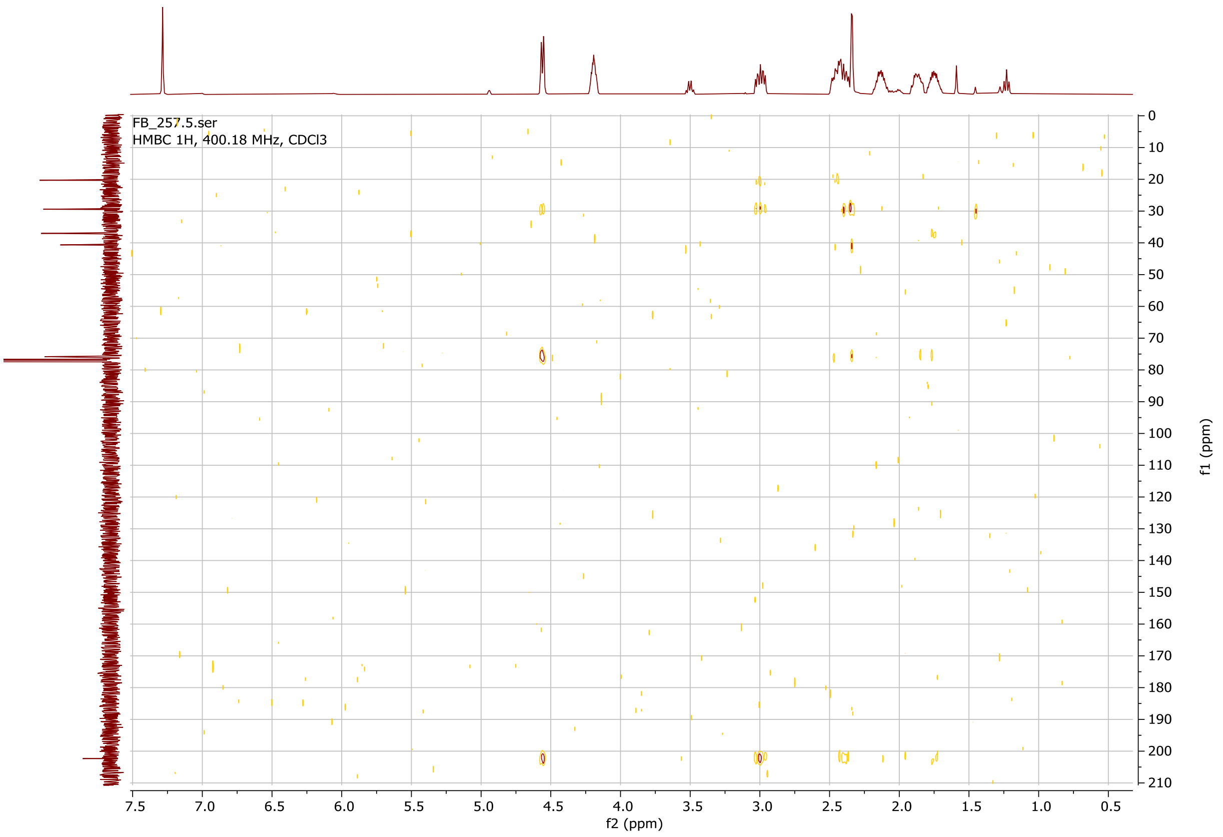
FB_257.5.ser
HMBC 1H, 400.18 MHz, CDCl3
f2 (ppm)
f1 (ppm)

((2-Iodo-2,3-dihydro-1*H*-inden-1-yl)oxy)dimethylvinylsilane **4a**

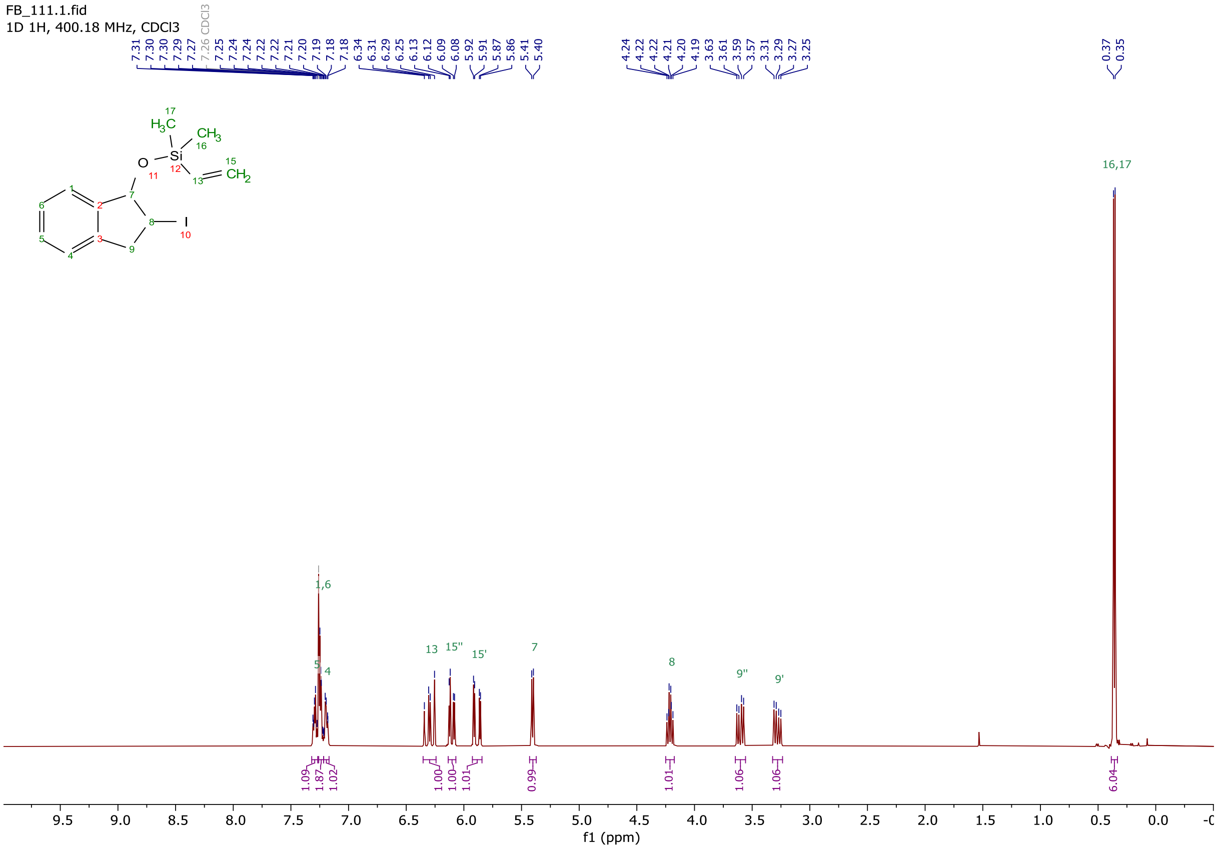

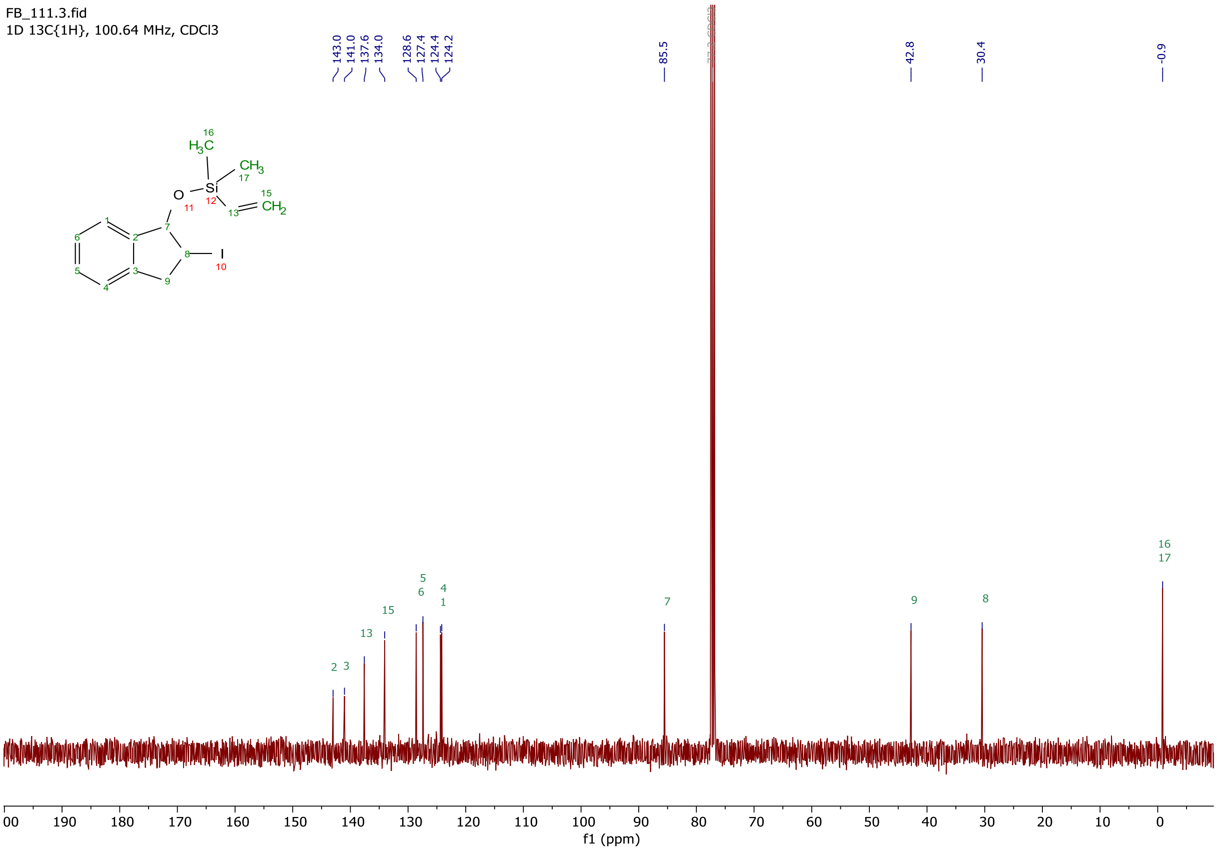

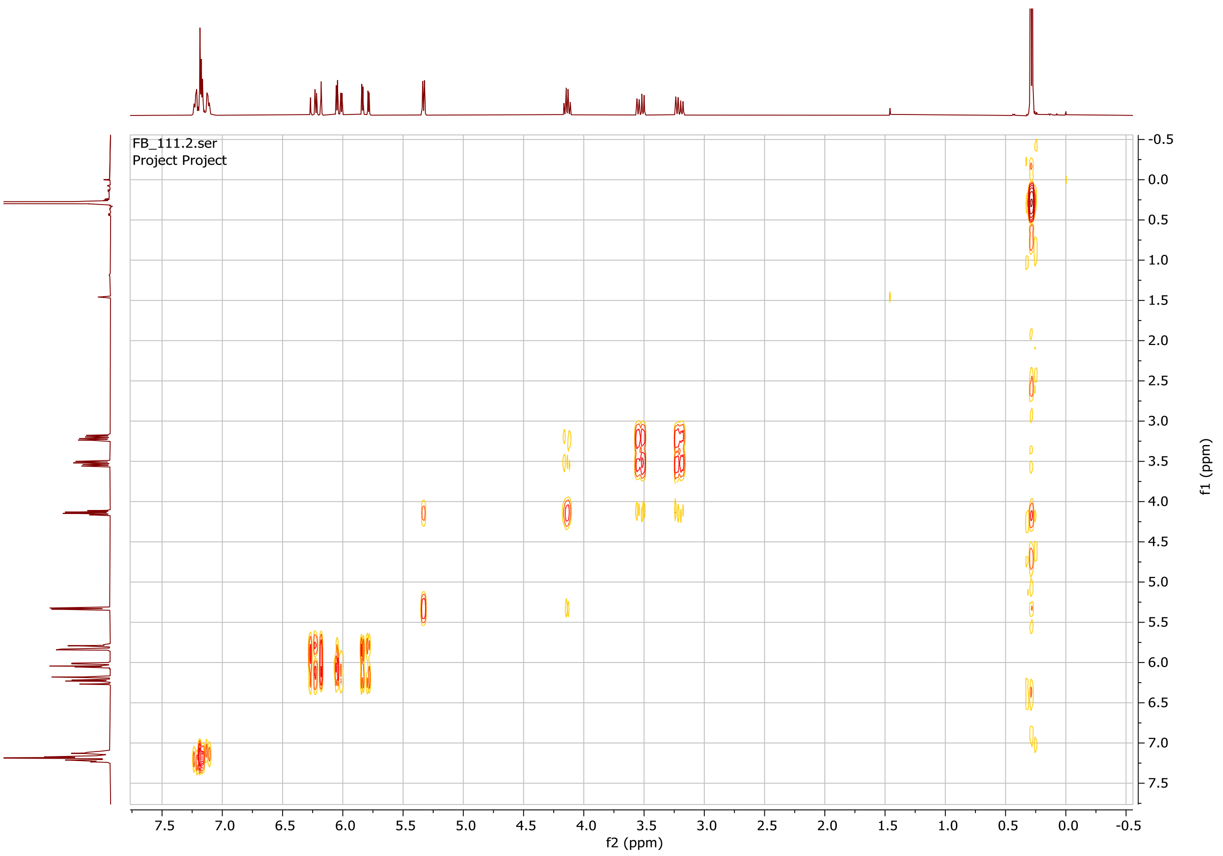
FB_111.2.ser
Project Project
f2 (ppm)
f1 (ppm)

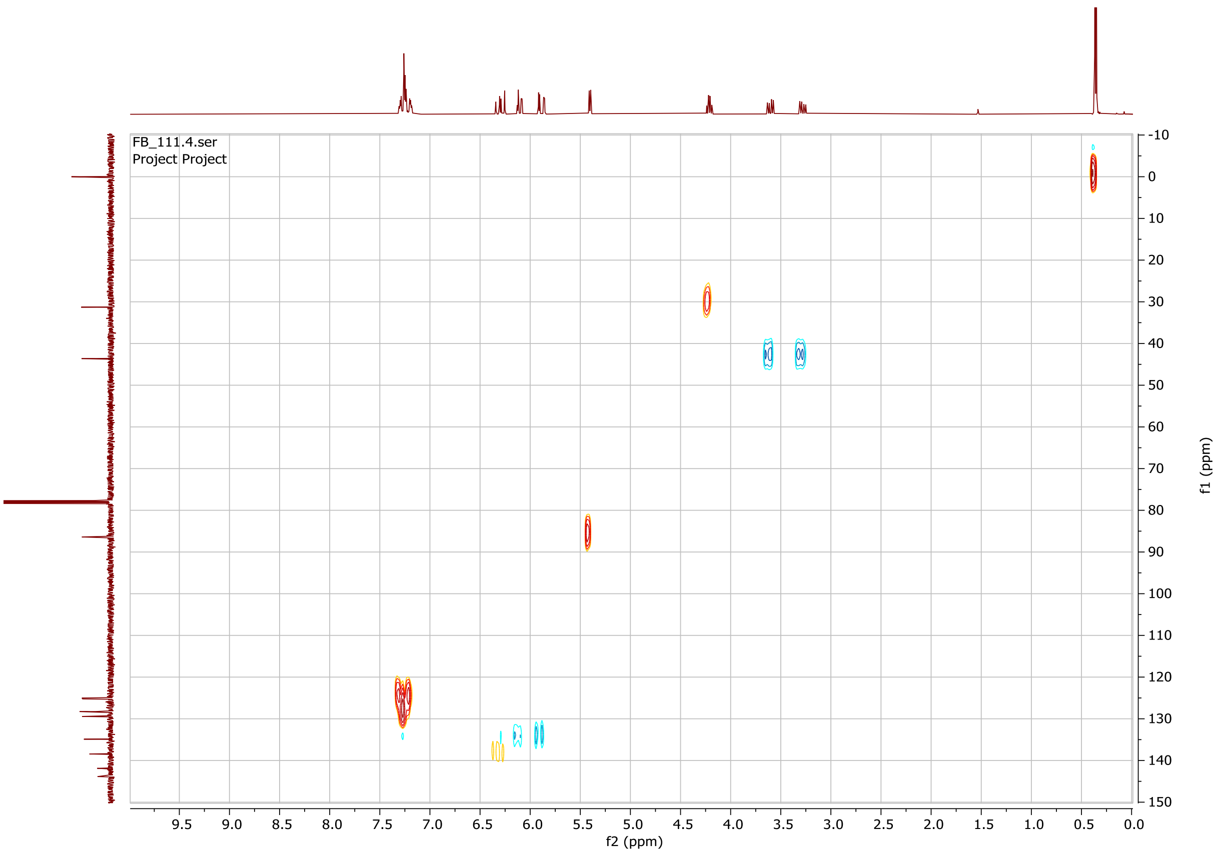
FB_111.4.ser
Project Project
f2 (ppm)
f1 (ppm)

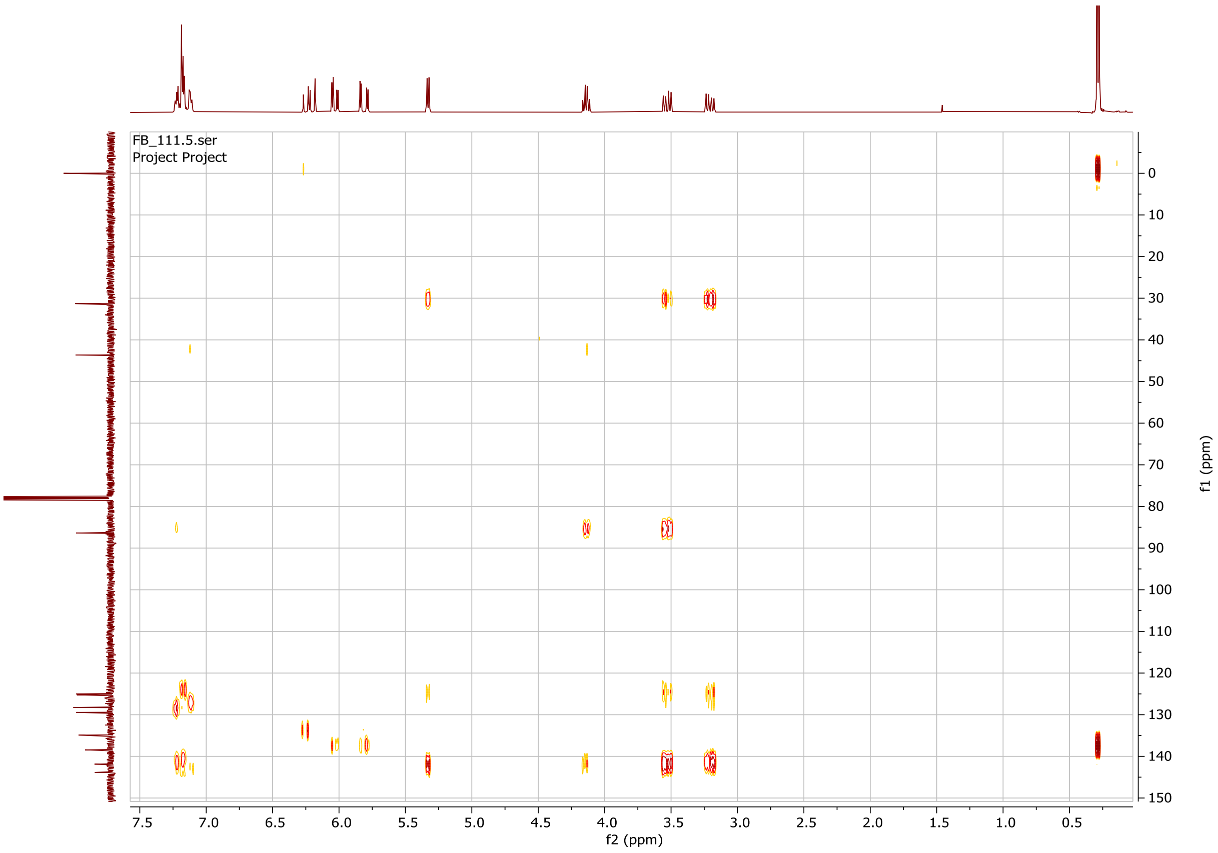

(2-Iodocyclopentyloxy)dimethylvinylsilane **4b**

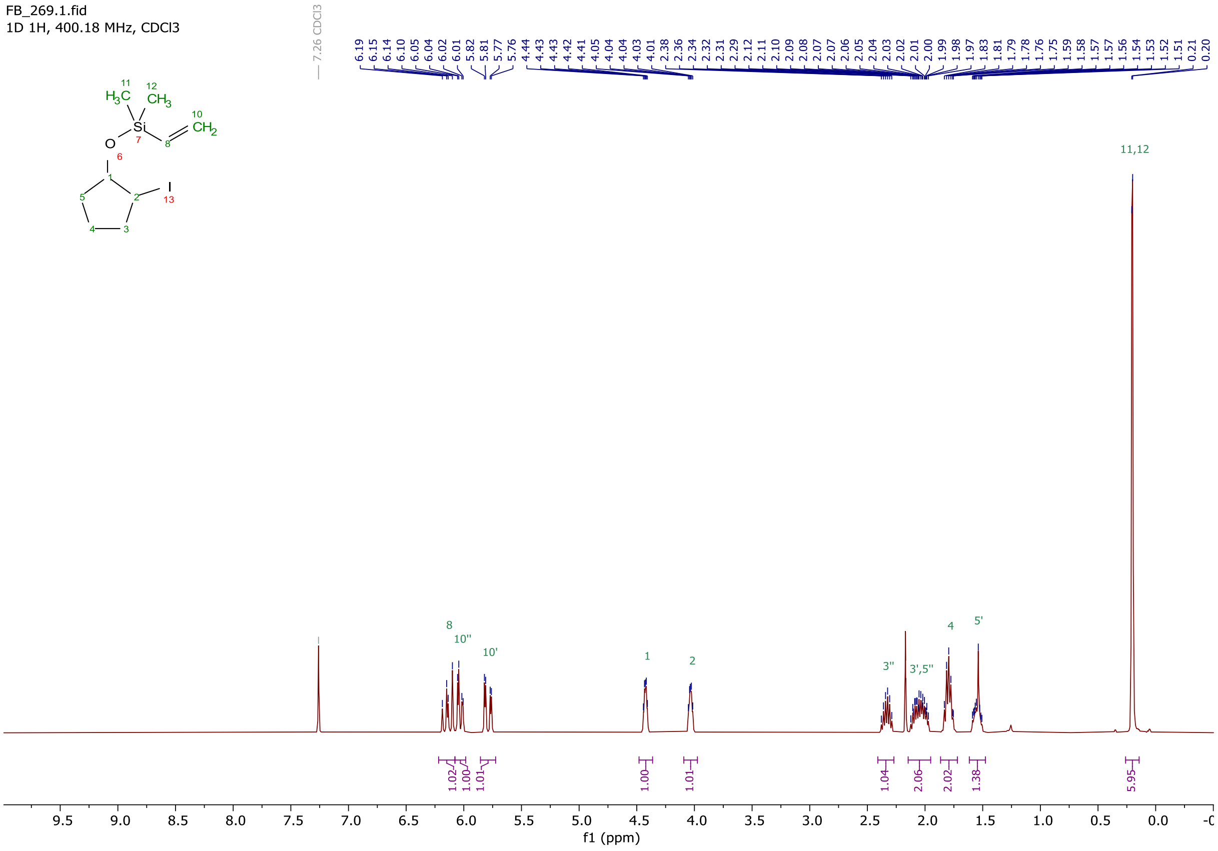

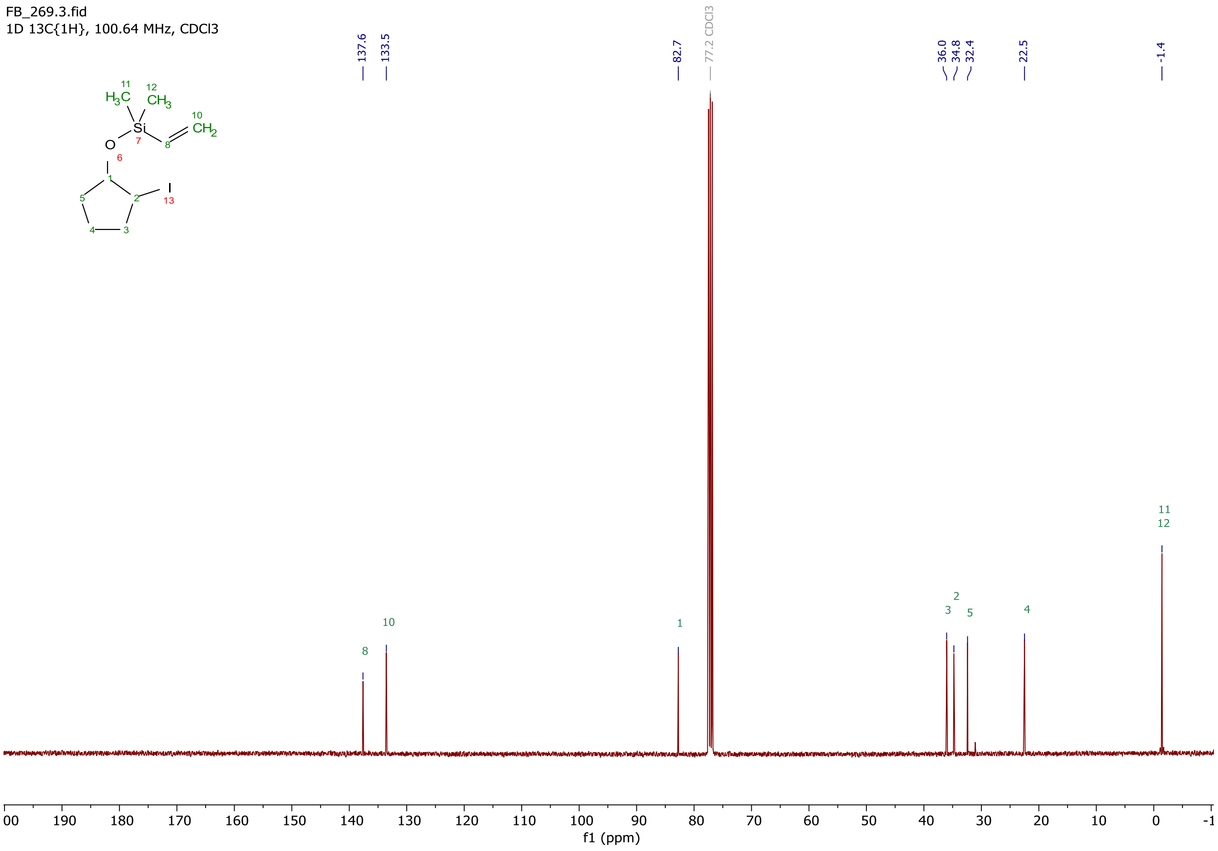
FB_269.3.fid
1D 13C{1H}, 100.64 MHz, CDCl3
137.6
133.5
82.7
77.2 CDCl3
36.0
34.8
32.4
22.5
-1.4
f1 (ppm)

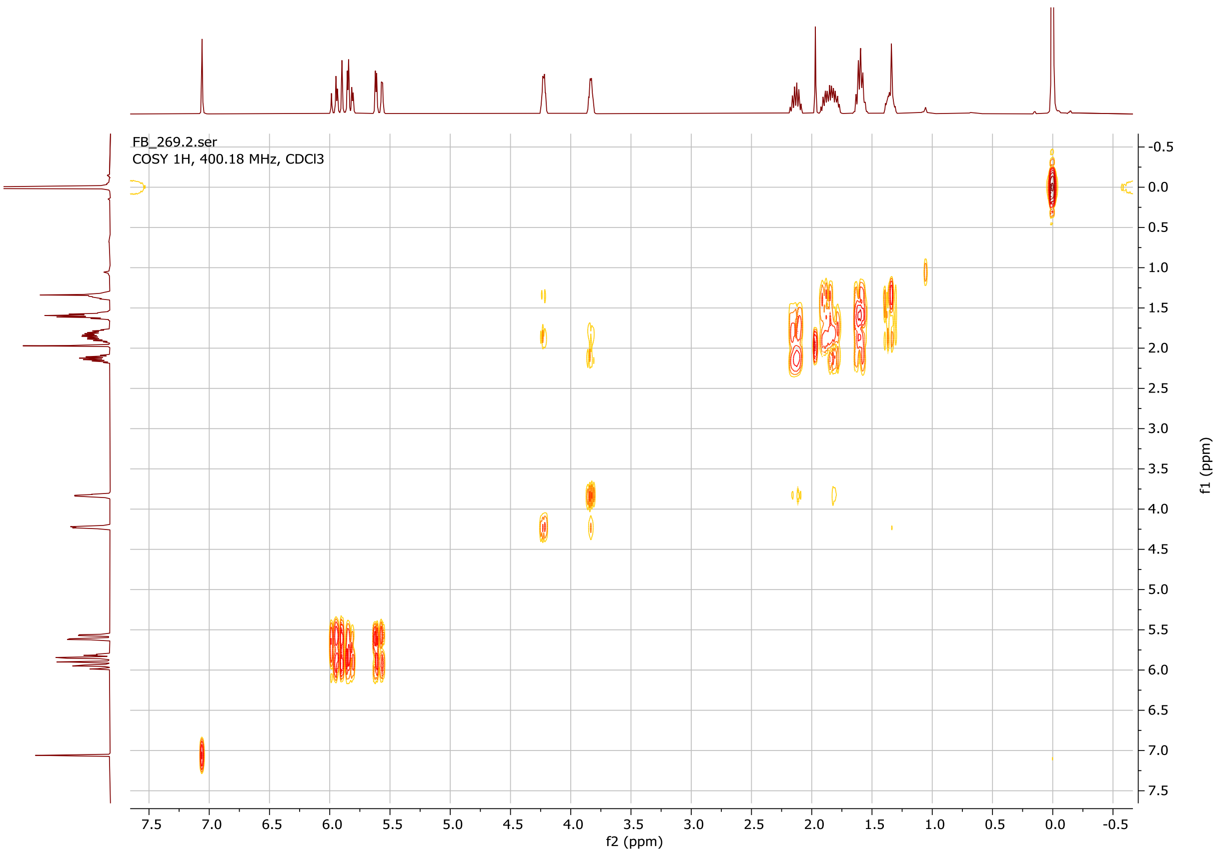
FB_269.2.ser
COSY 1H, 400.18 MHz, CDCl3
f2 (ppm)
f1 (ppm)

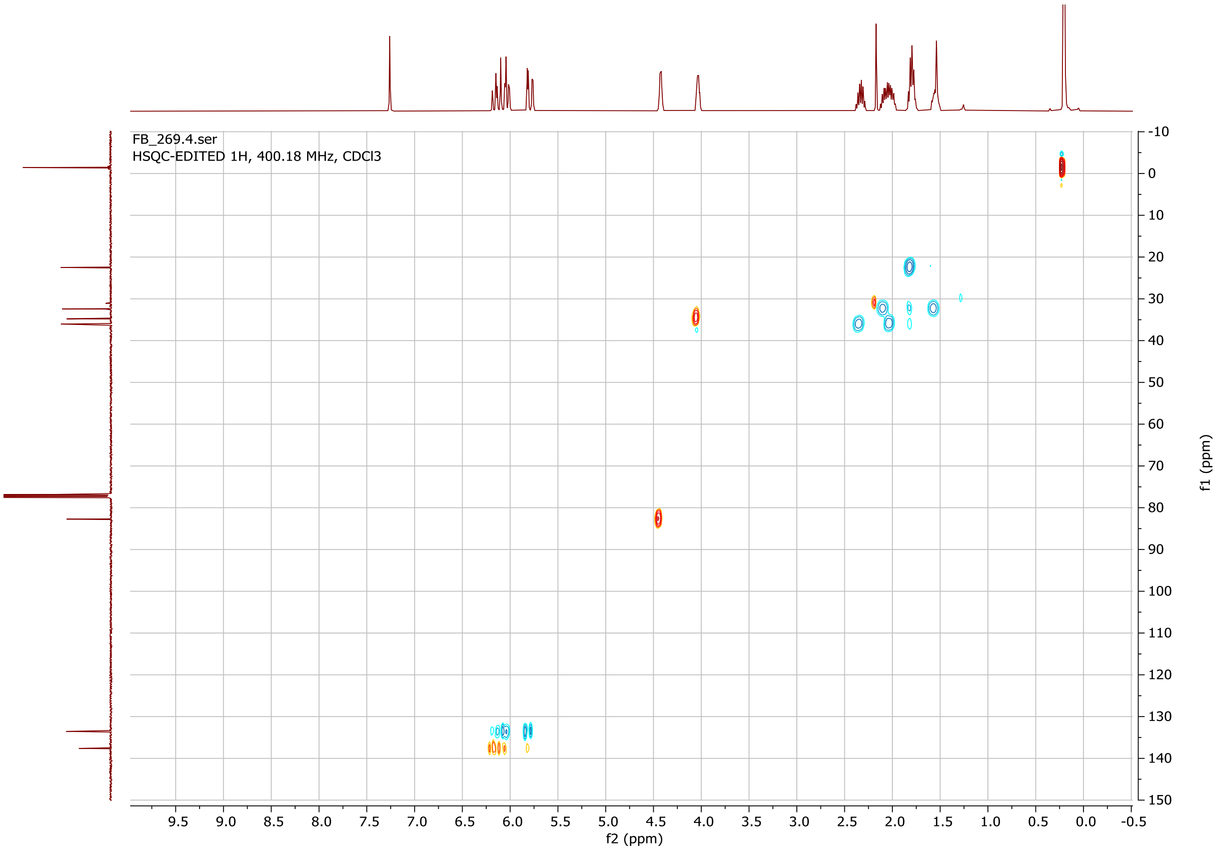
FB_269.4.ser
HSQC-EDITED 1H, 400.18 MHz, CDCl3
f2 (ppm)
f1 (ppm)

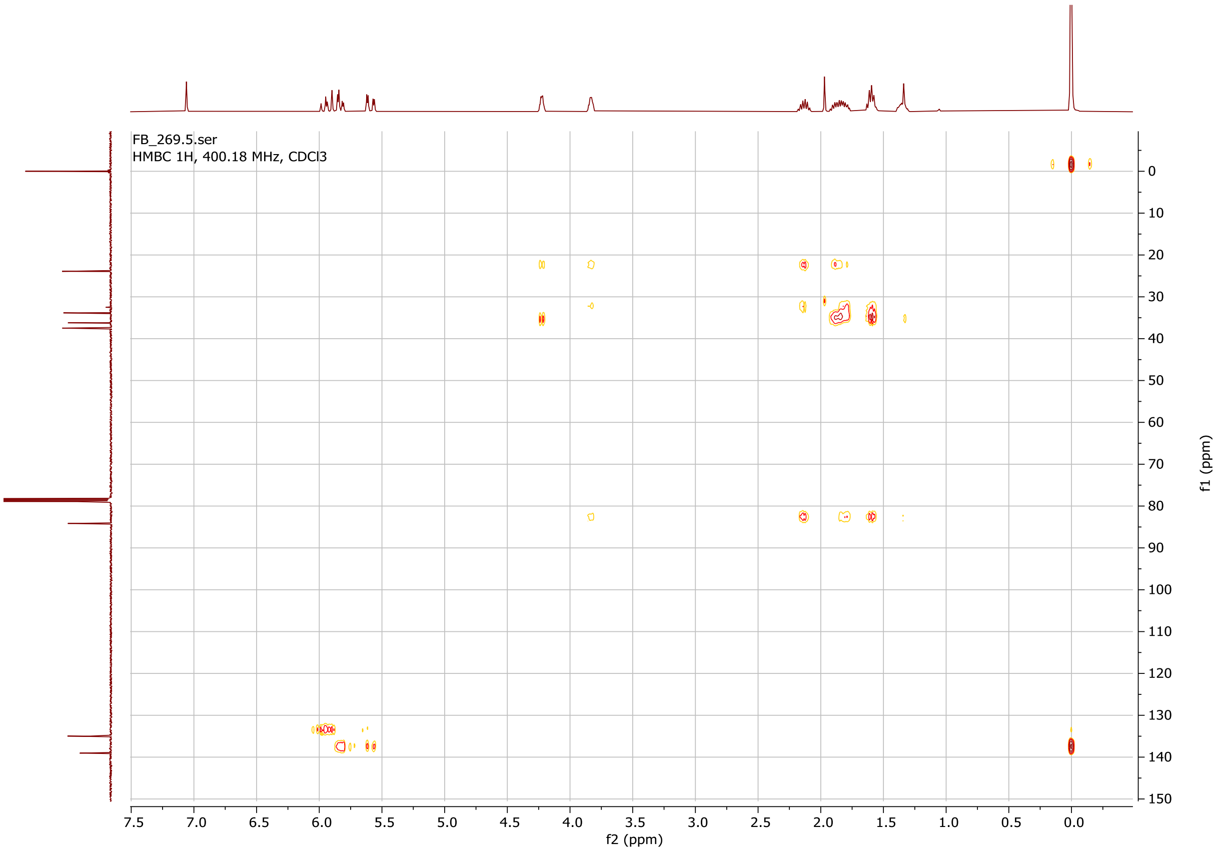
FB_269.5.ser
HMBC 1H, 400.18 MHz, CDCl3
f2 (ppm)
f1 (ppm)

(2-Iodocyclohexyloxy)dimethylvinylsilane **4c**

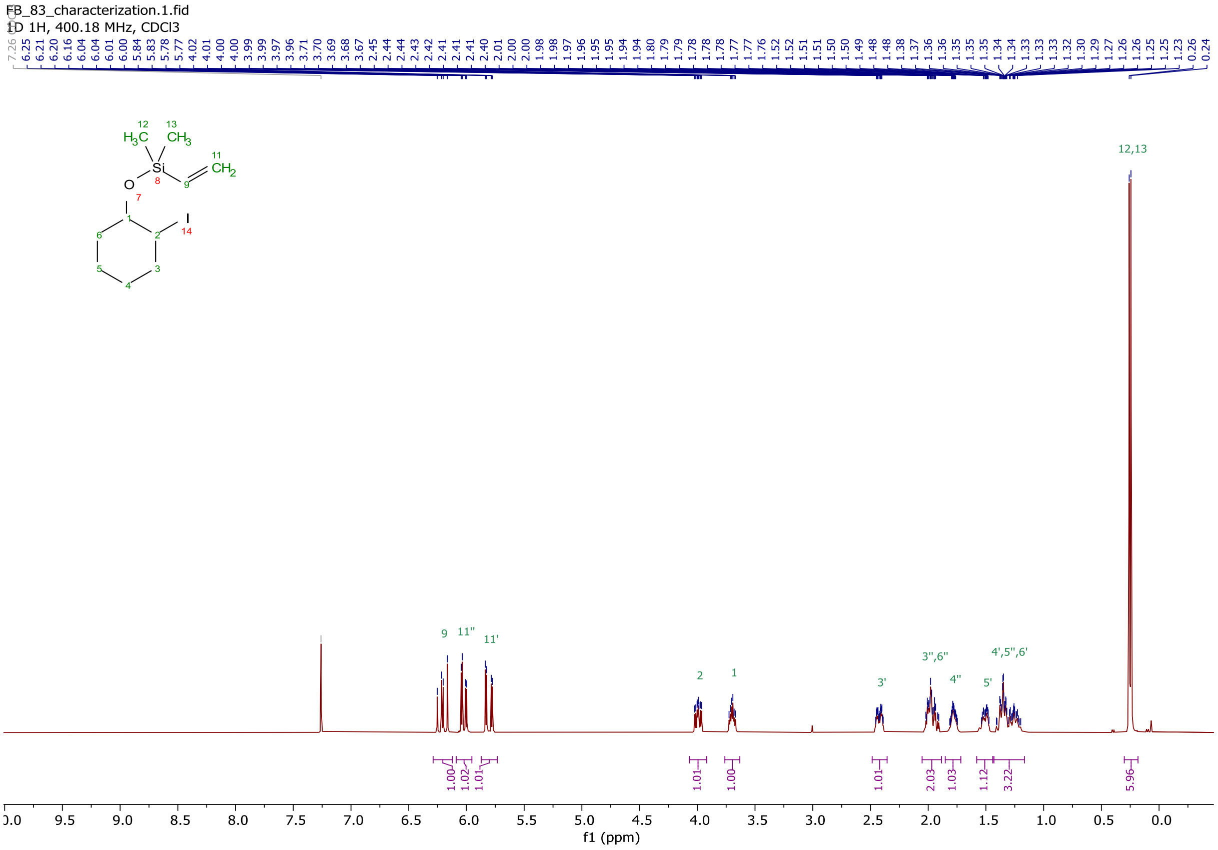

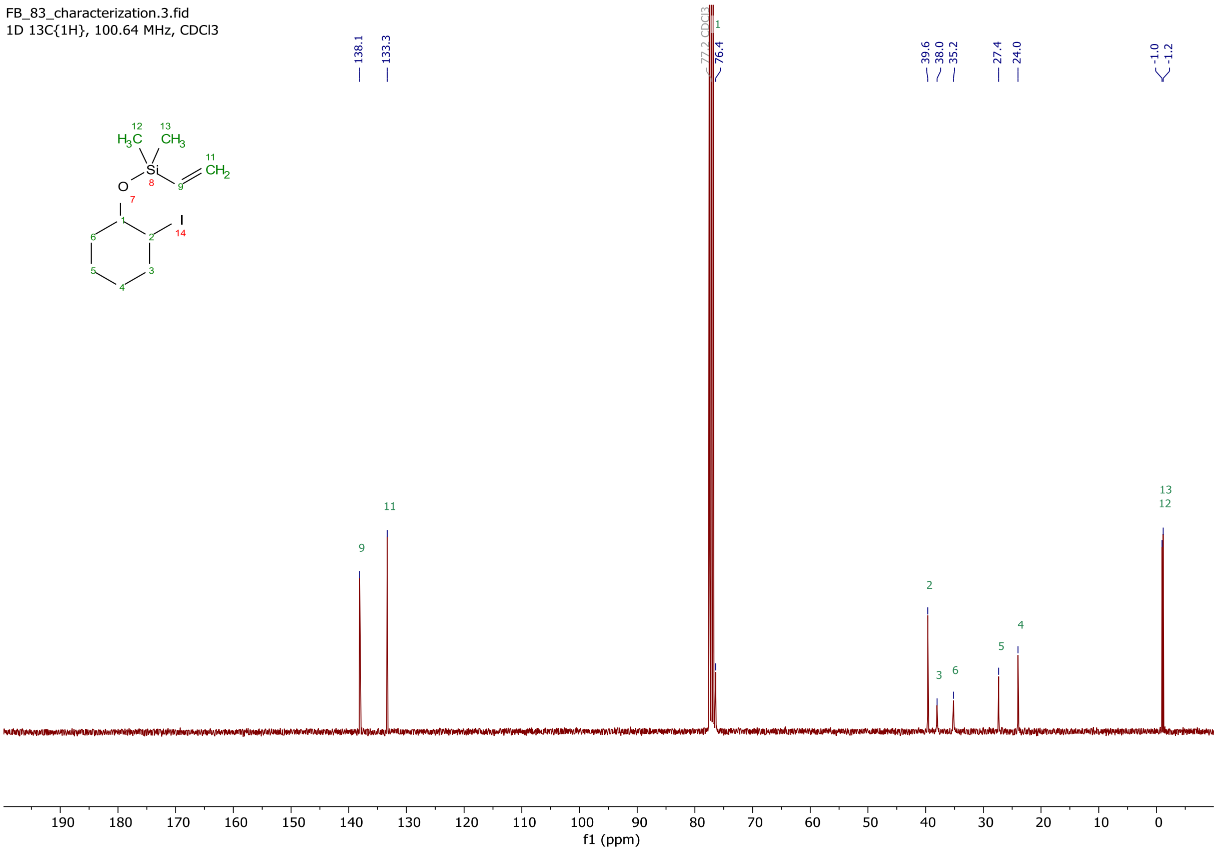

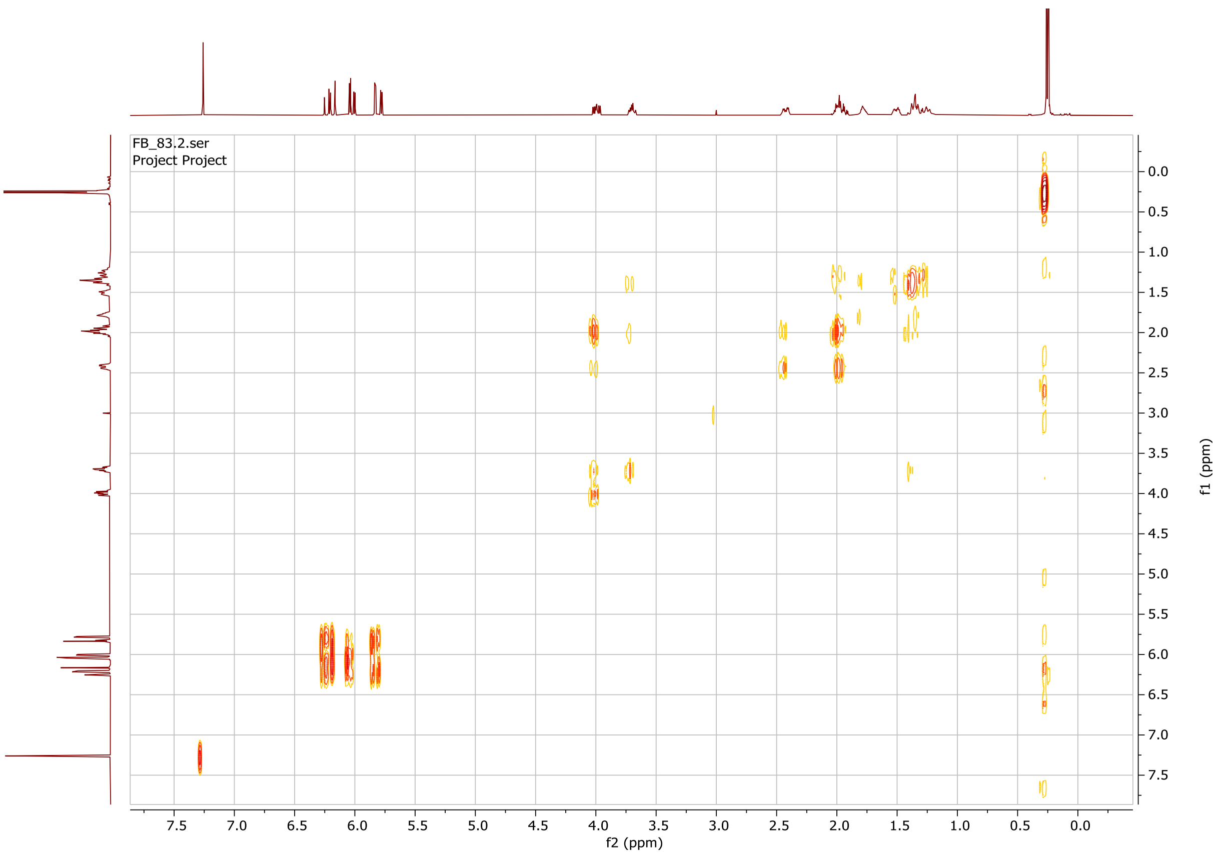
FB_83.2.ser
Project Project
f1 (ppm)
f2 (ppm)

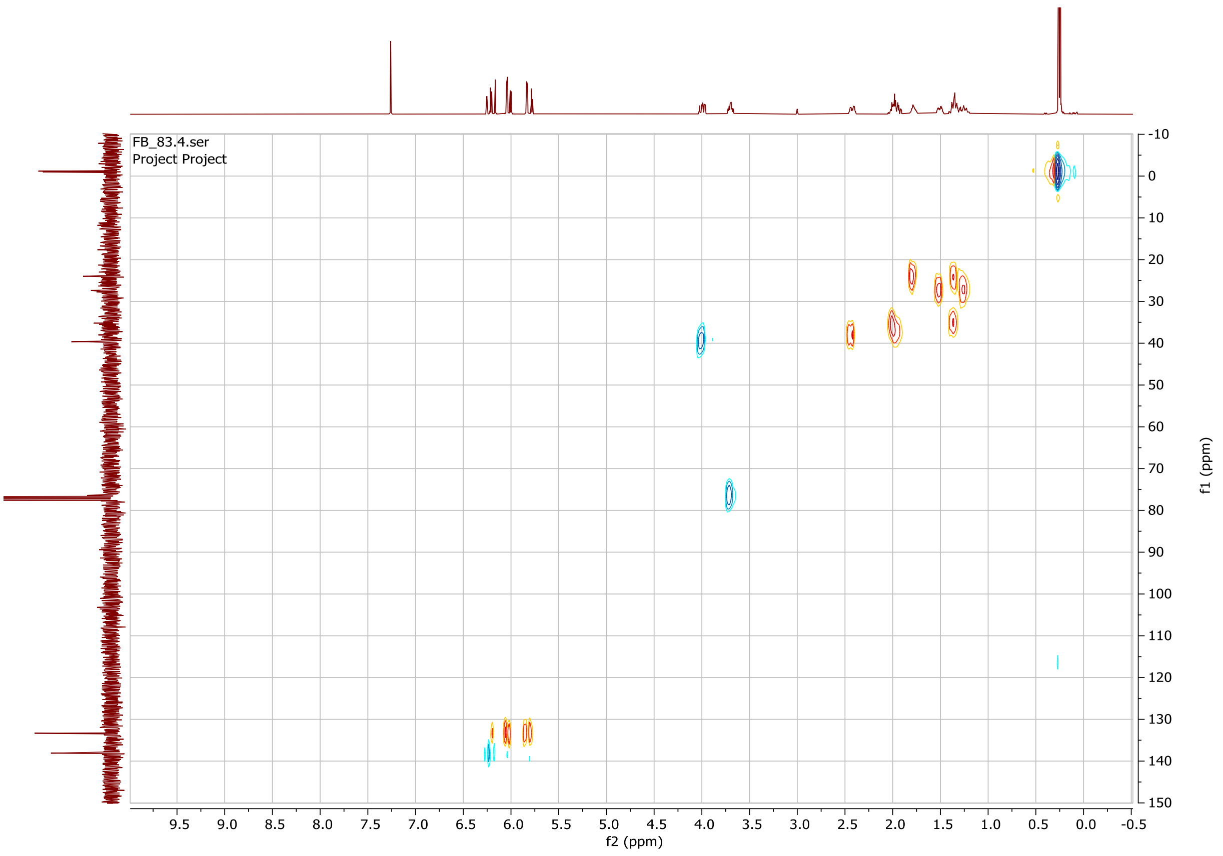
FB_83.4.ser
Project Project
f1 (ppm)
f2 (ppm)

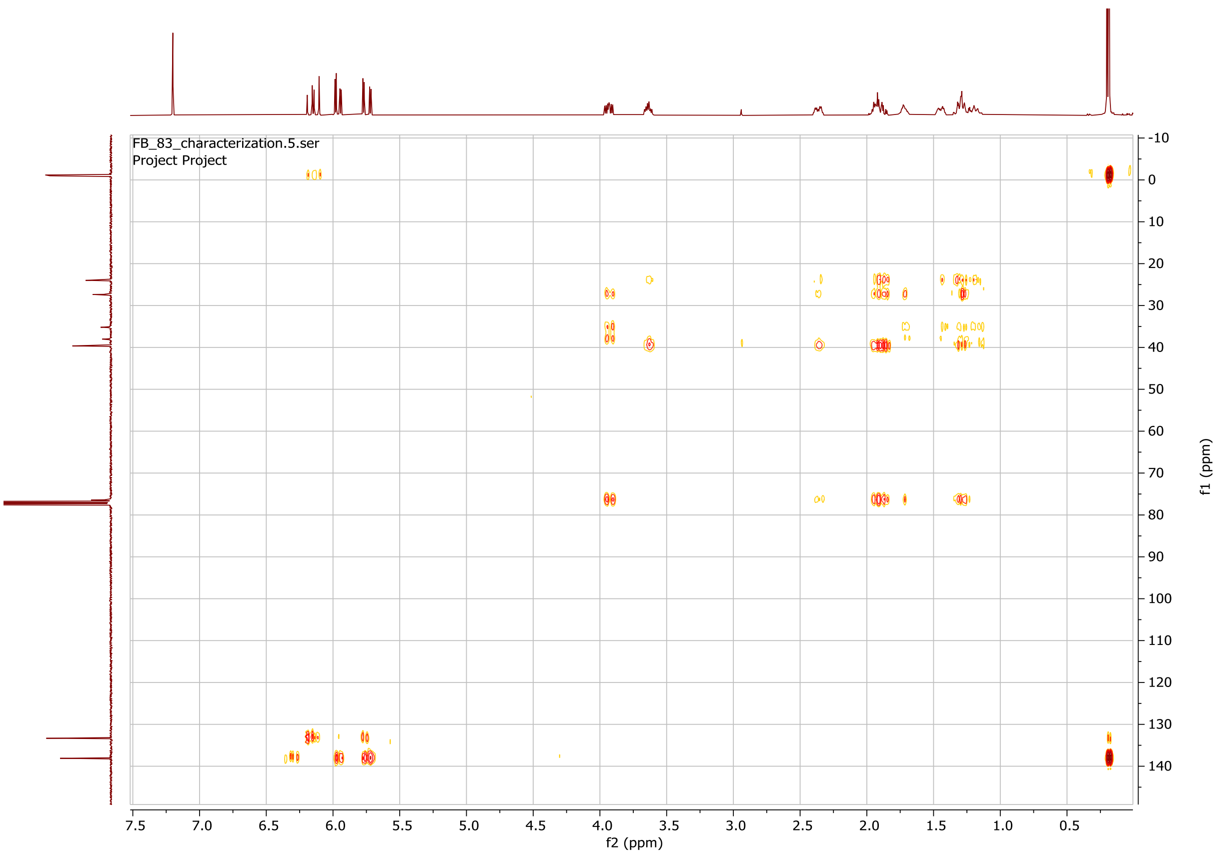

tert-Butyl 3-((dimethylvinylsilyl)oxy)-4-iodopyrrolidine-1-carboxylate **4d** *(mixture of rotamers)*

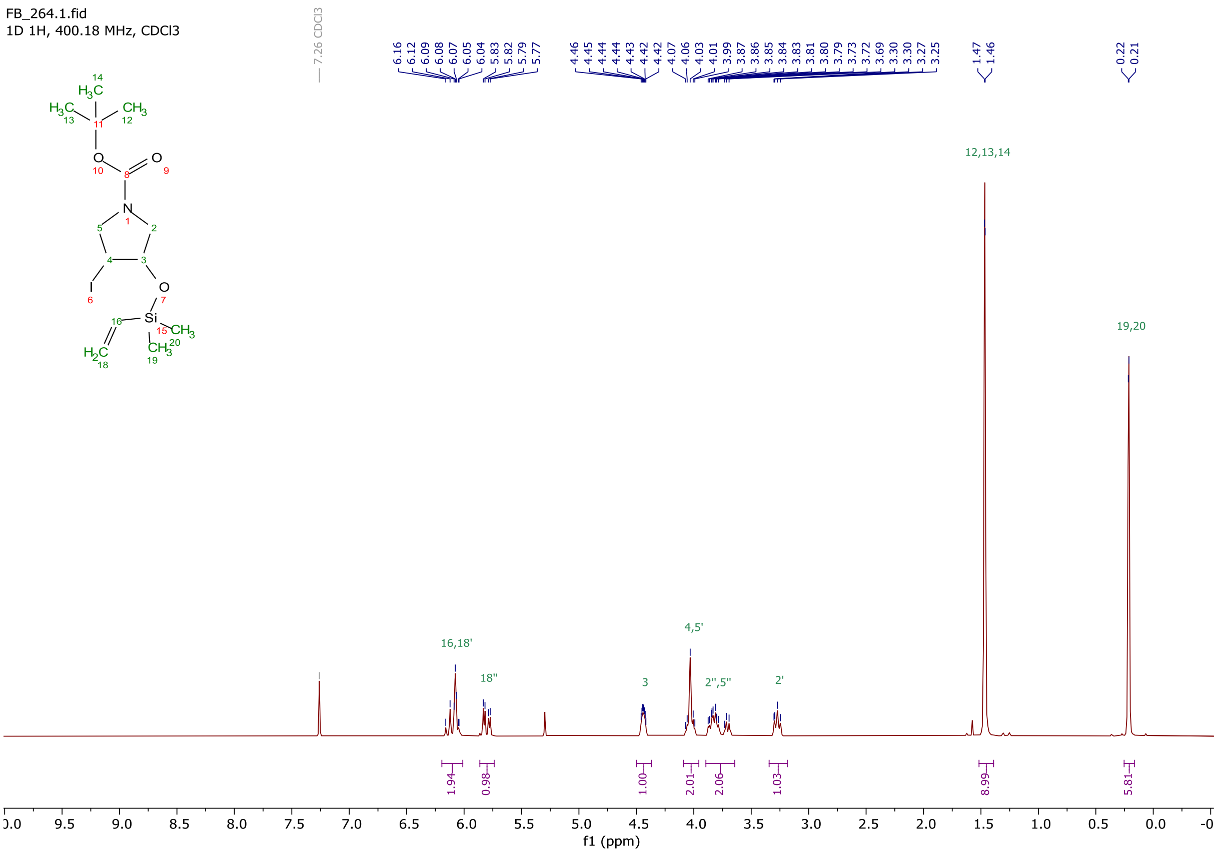

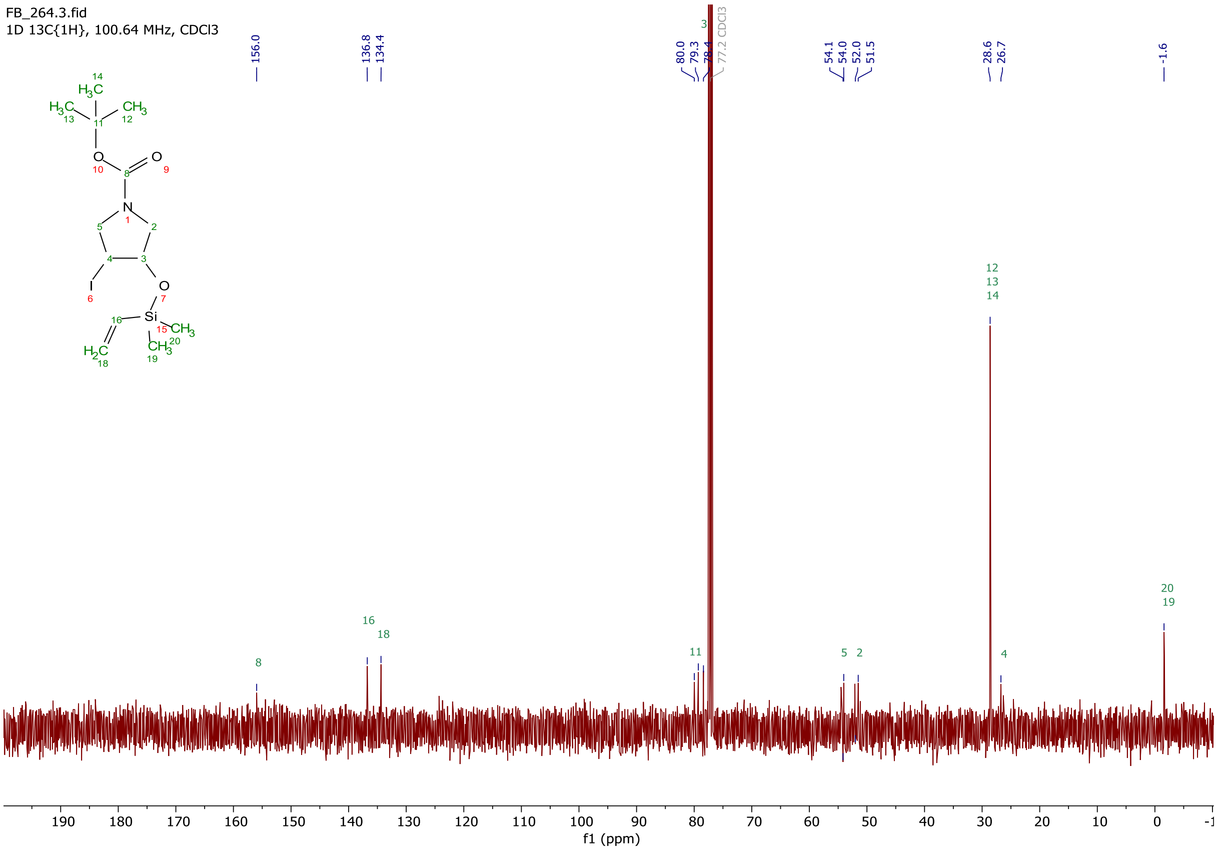
FB_264.3.fid
1D 13C{1H}, 100.64 MHz, CDCl3
156.0
136.8
134.4
80.0
79.3
78.4
77.2 CDCl3
54.1
54.0
52.0
51.5
28.6
26.7
-1.6
f1 (ppm)

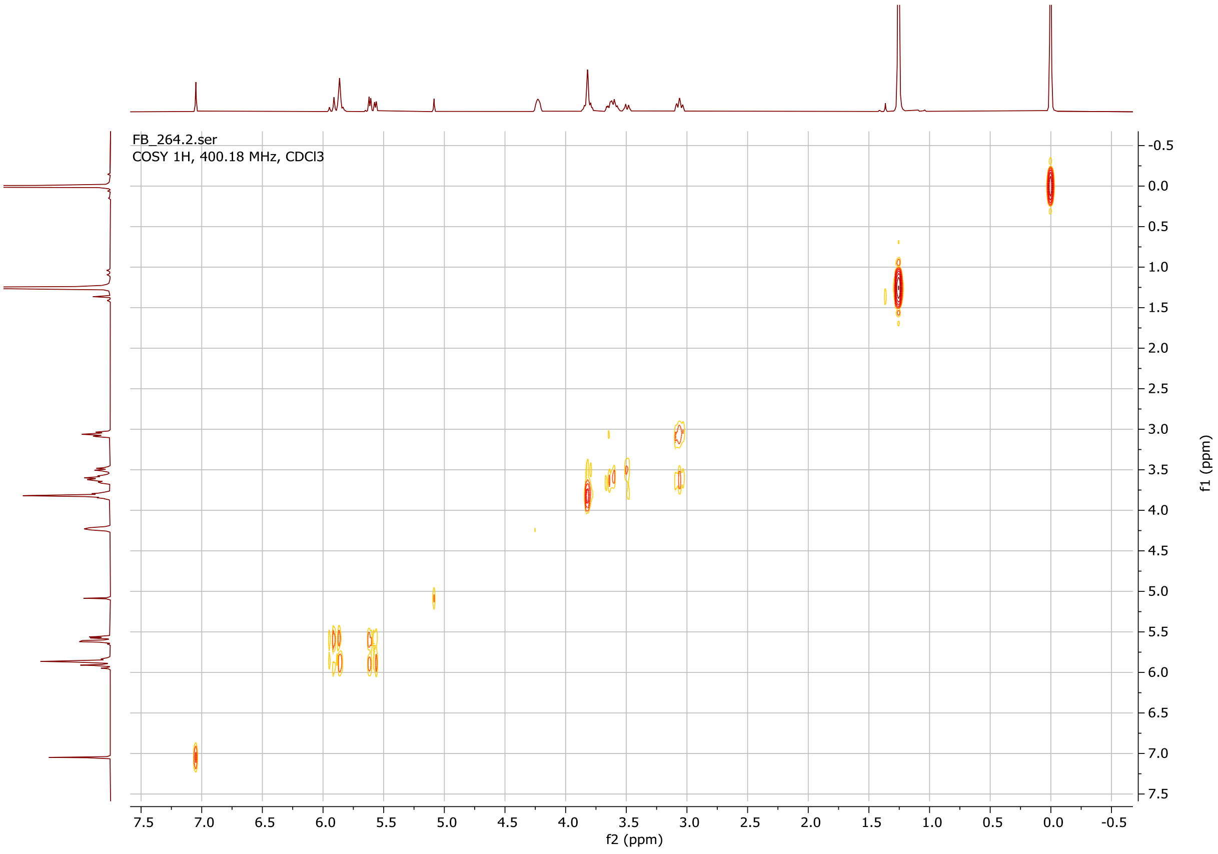
FB_264.2.ser
COSY 1H, 400.18 MHz, CDCl3
f2 (ppm)
f1 (ppm)

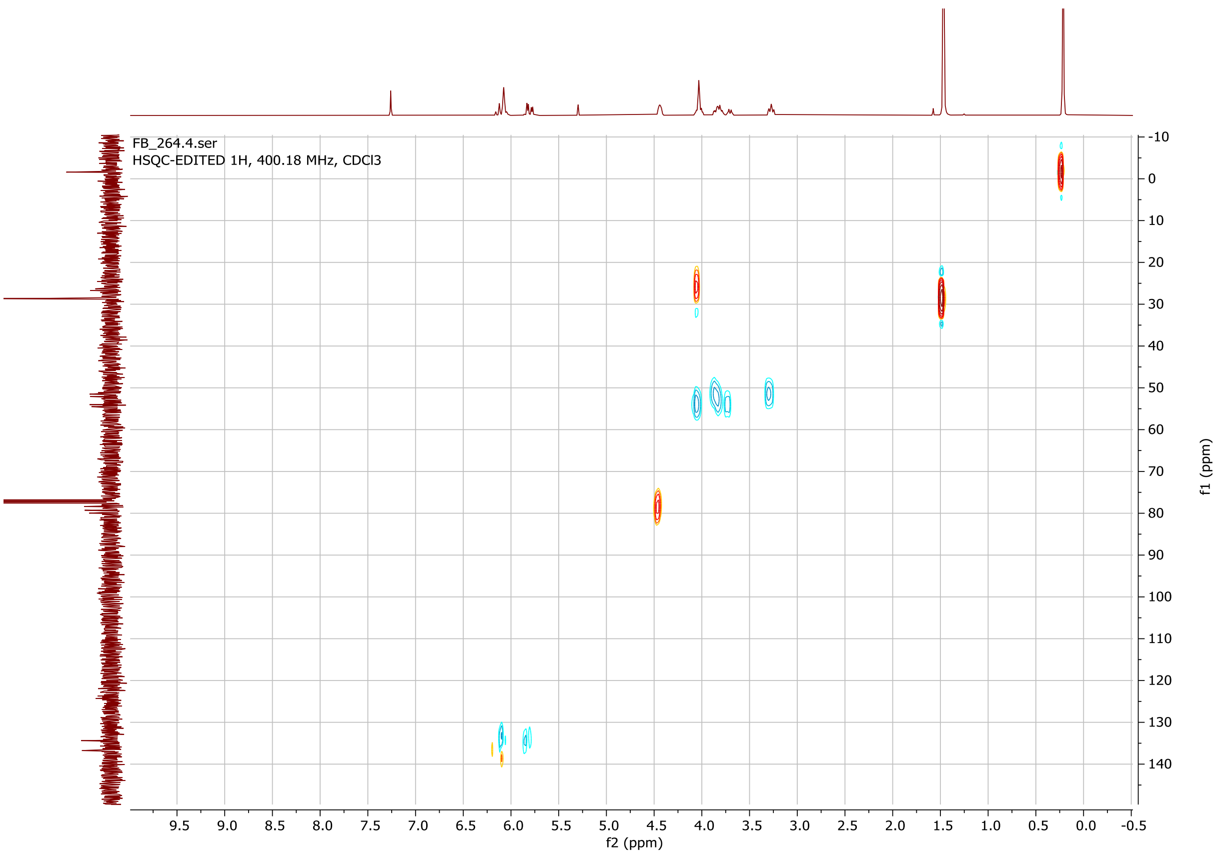
FB_264.4.ser
HSQC-EDITED 1H, 400.18 MHz, CDCl3
f2 (ppm)
f1 (ppm)

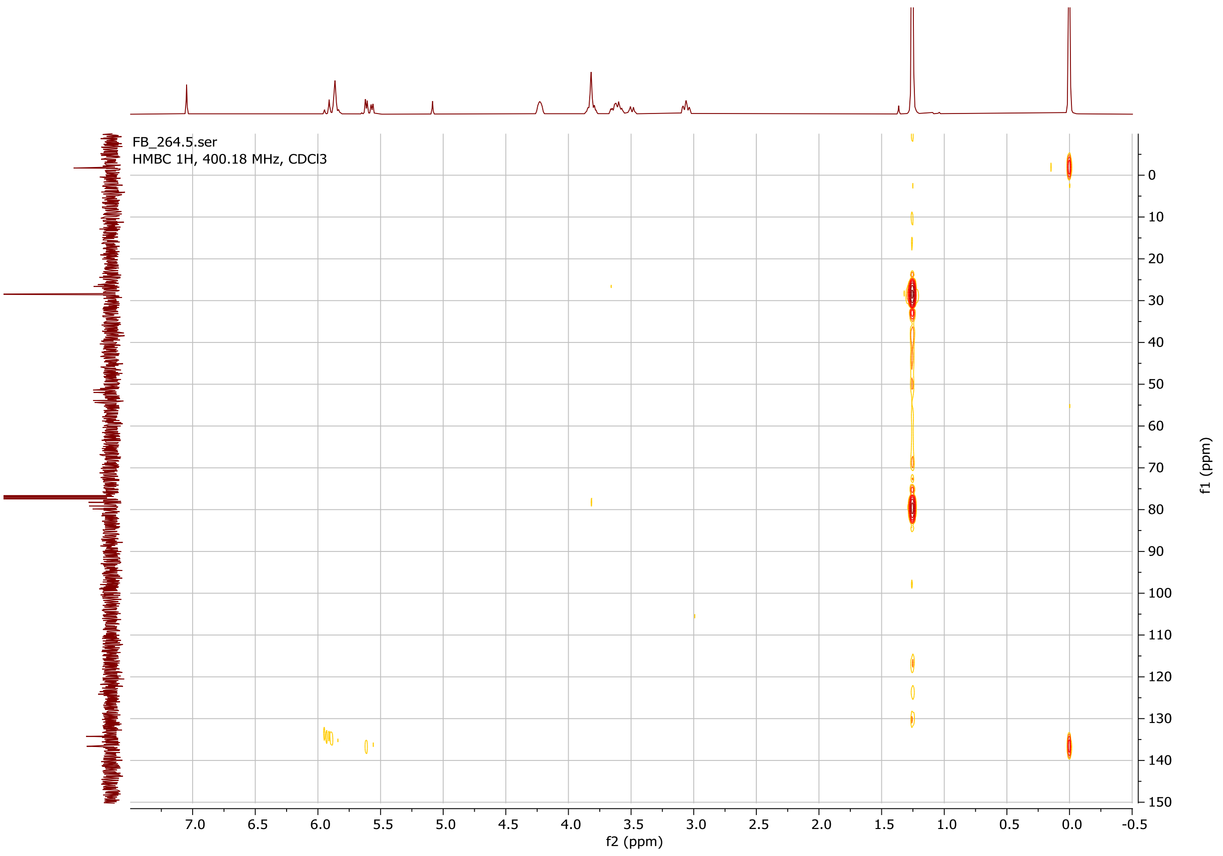
FB_264.5.ser
HMBC 1H, 400.18 MHz, CDCl3
f2 (ppm)
f1 (ppm)

((4-(2-Benzyloxy)propan-2-yl)-2-iodo-1-methylcyclohexyl)oxy)dimethylvinylsilane **4e**

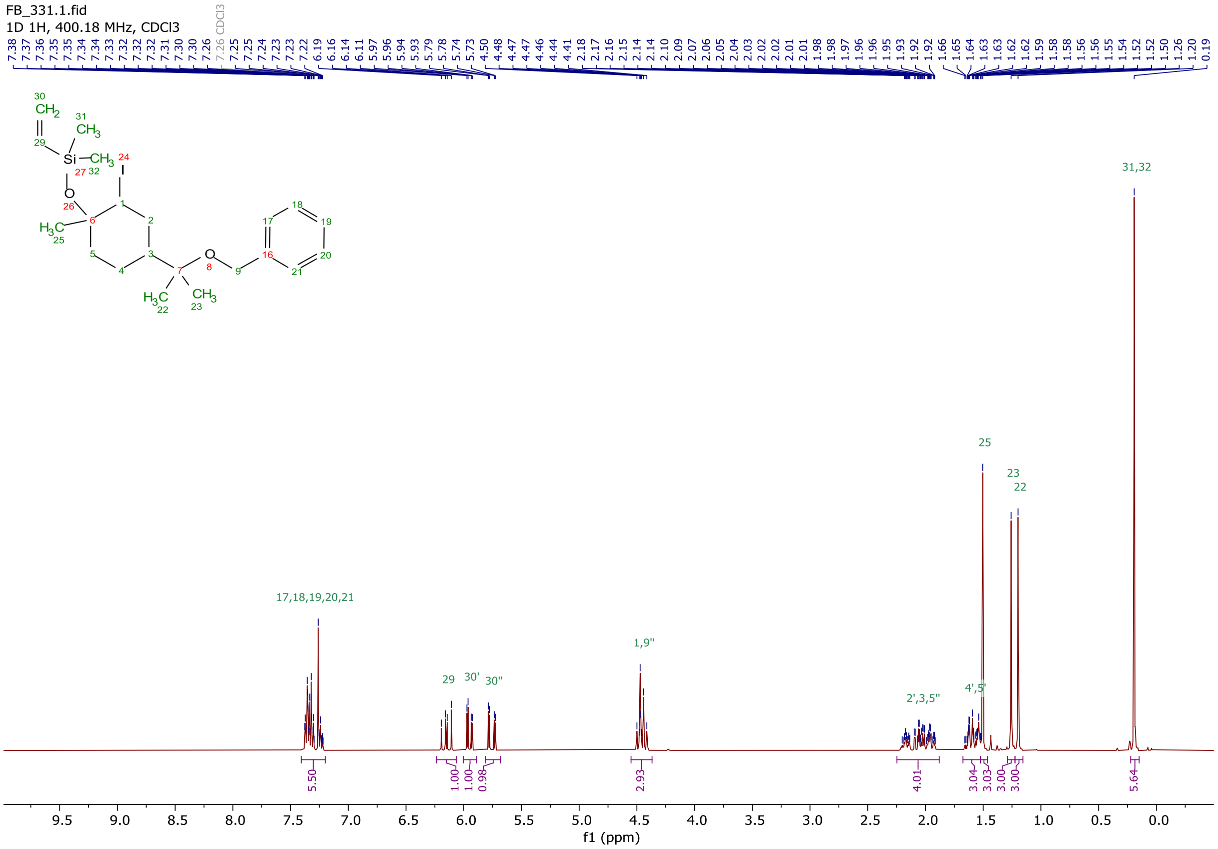

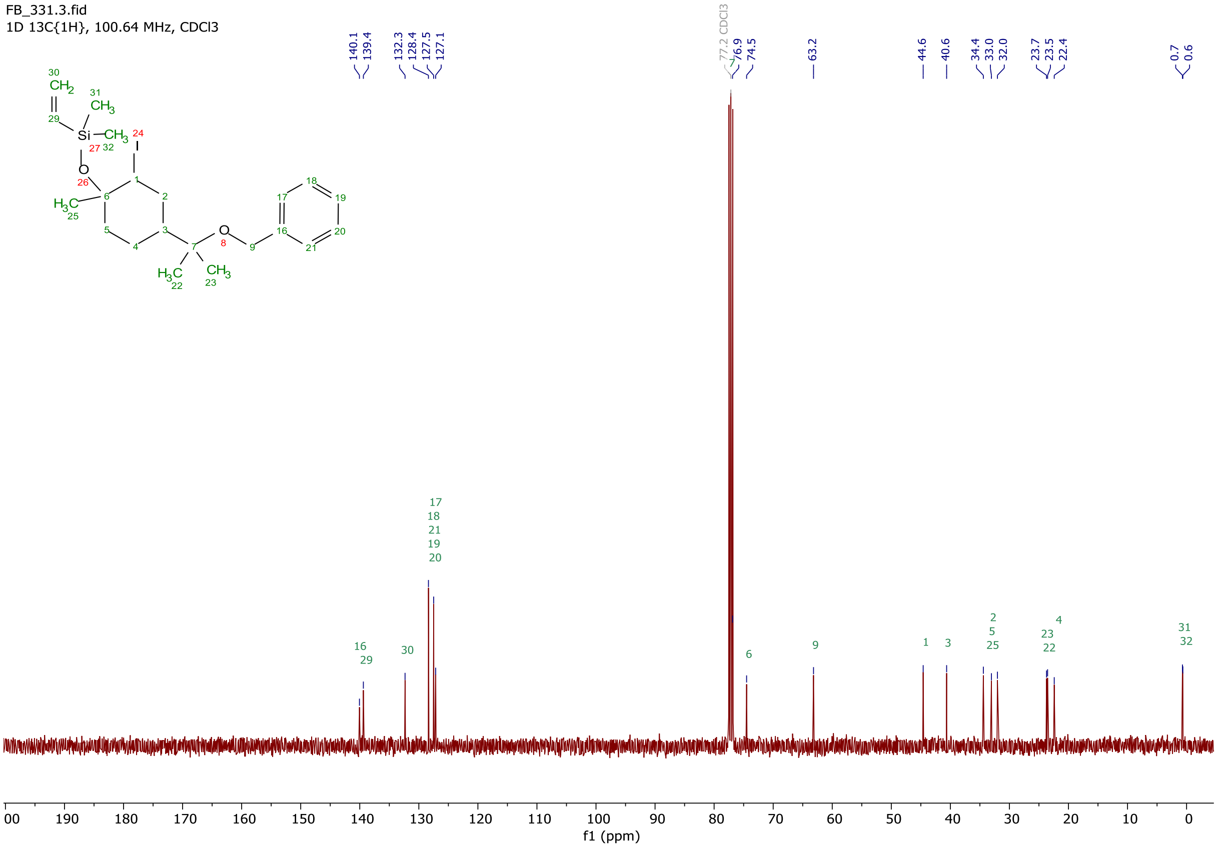

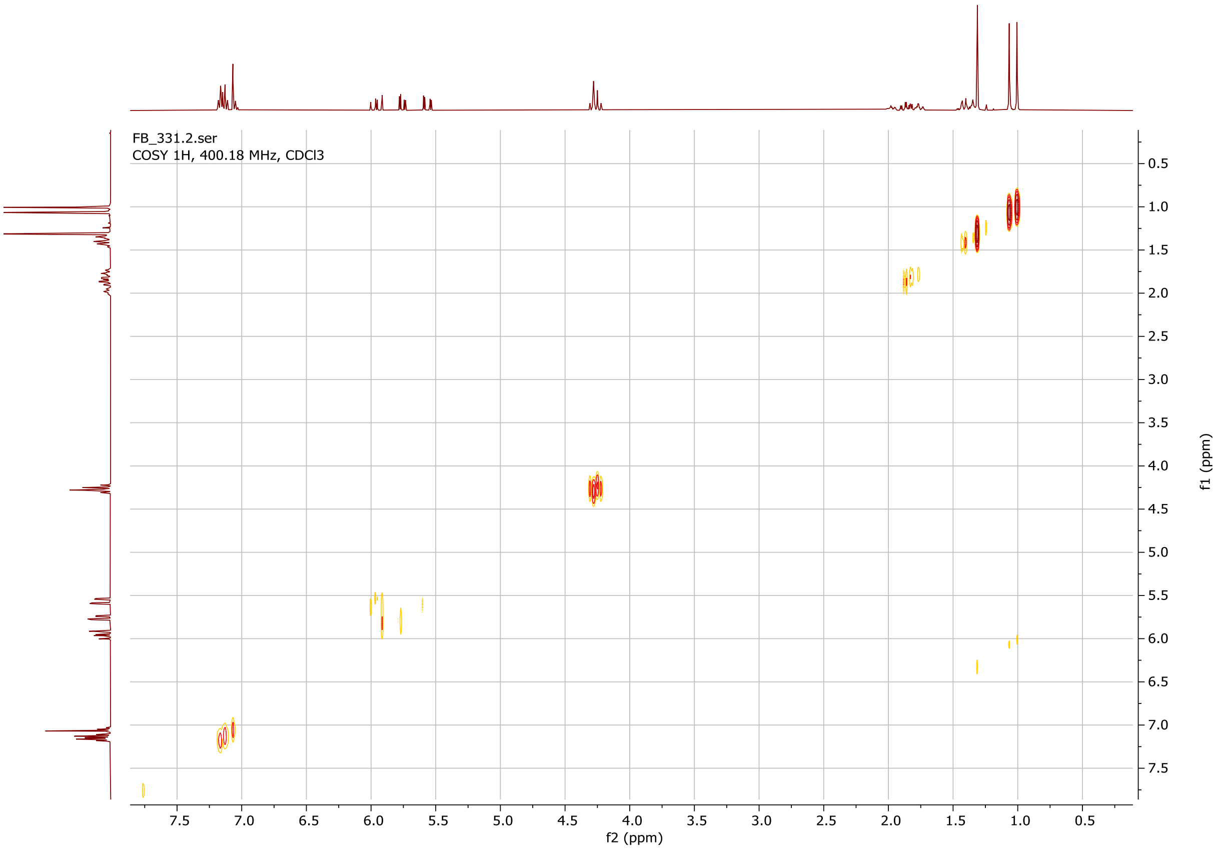
FB_331.2.ser
COSY 1H, 400.18 MHz, CDCl3
f1 (ppm)
f2 (ppm)

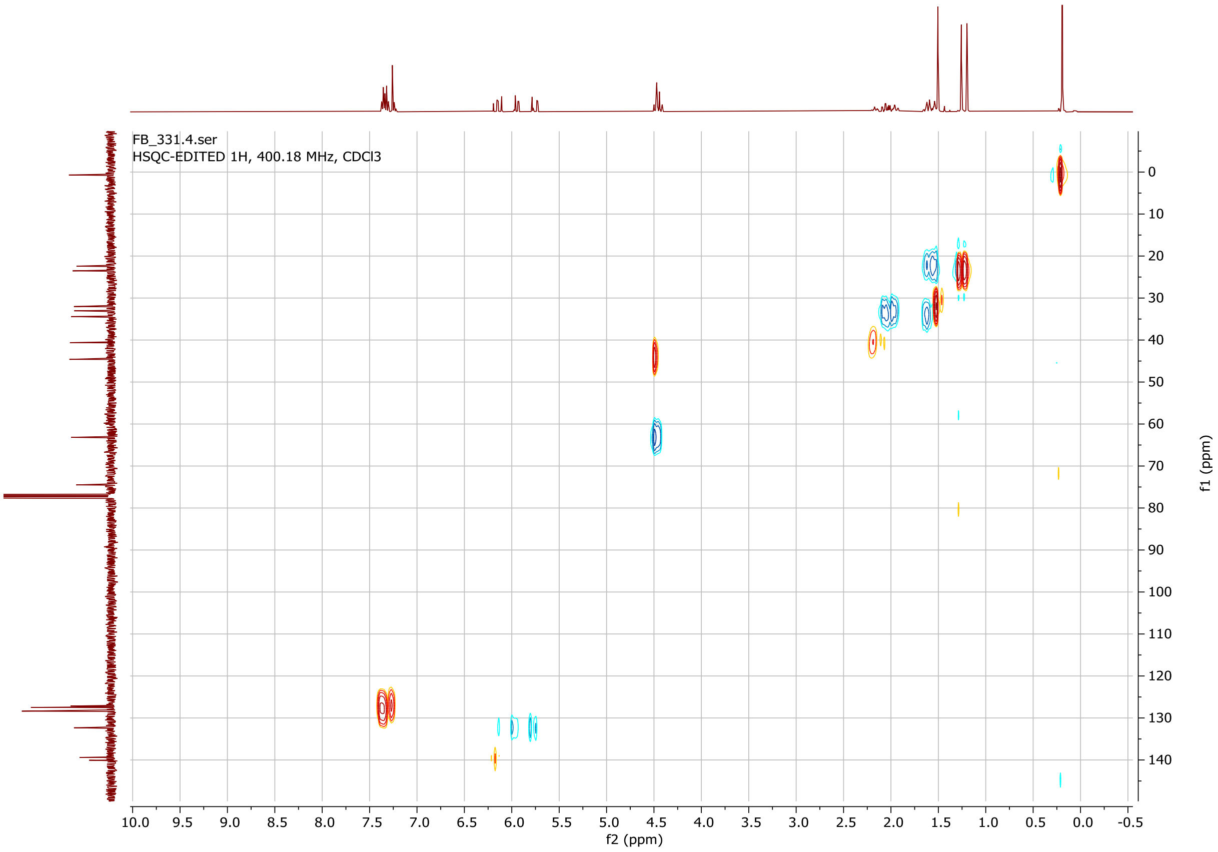
FB_331.4.ser
HSQC-EDITED 1H, 400.18 MHz, CDCl3
f1 (ppm)
f2 (ppm)

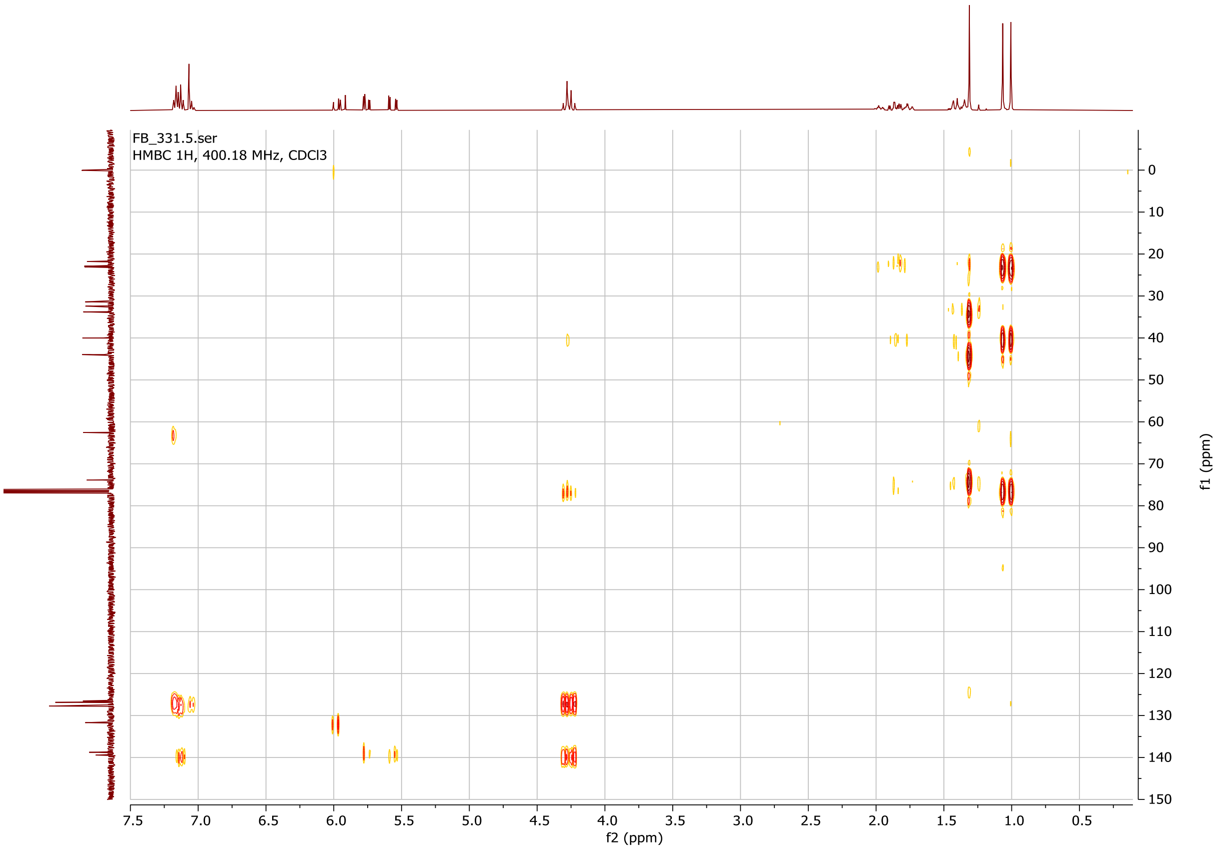

(2-Iodo-1-phenylpropoxy)dimethylvinylsilane **4f**

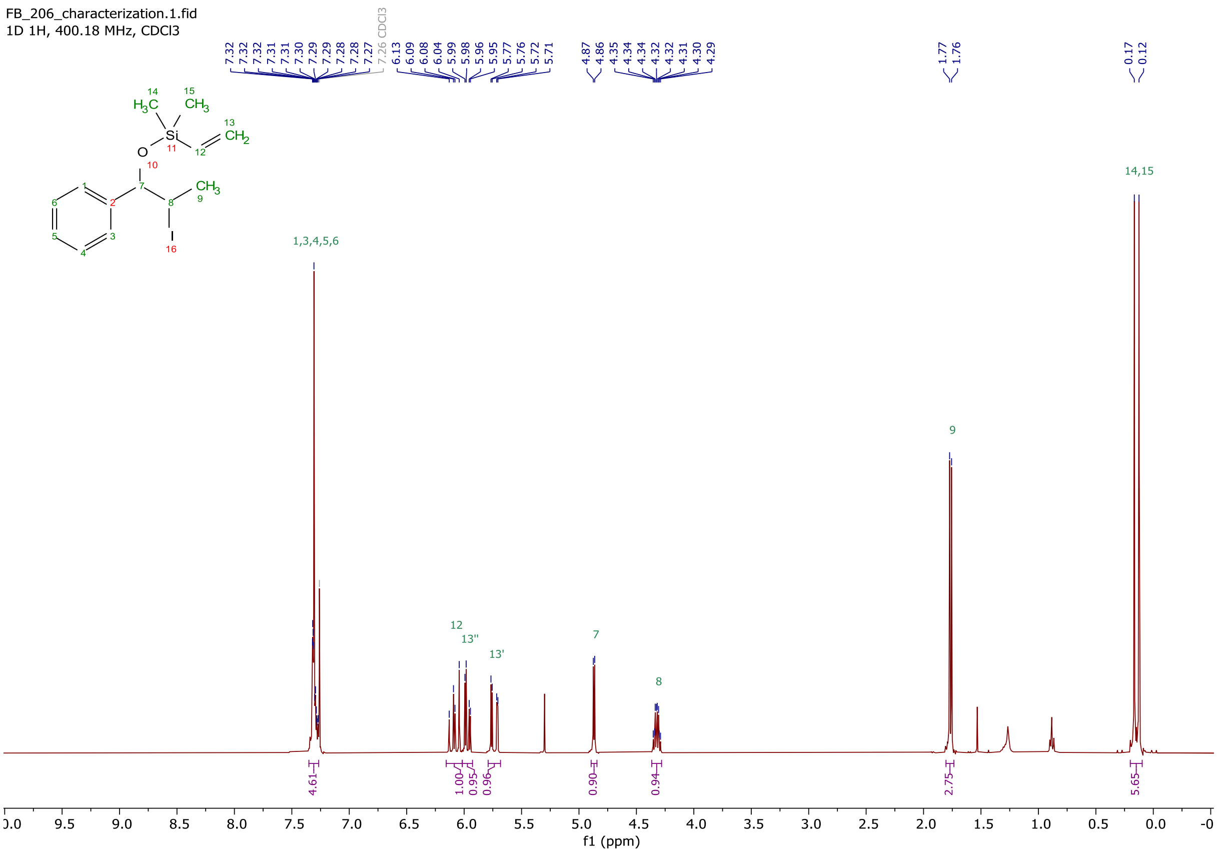

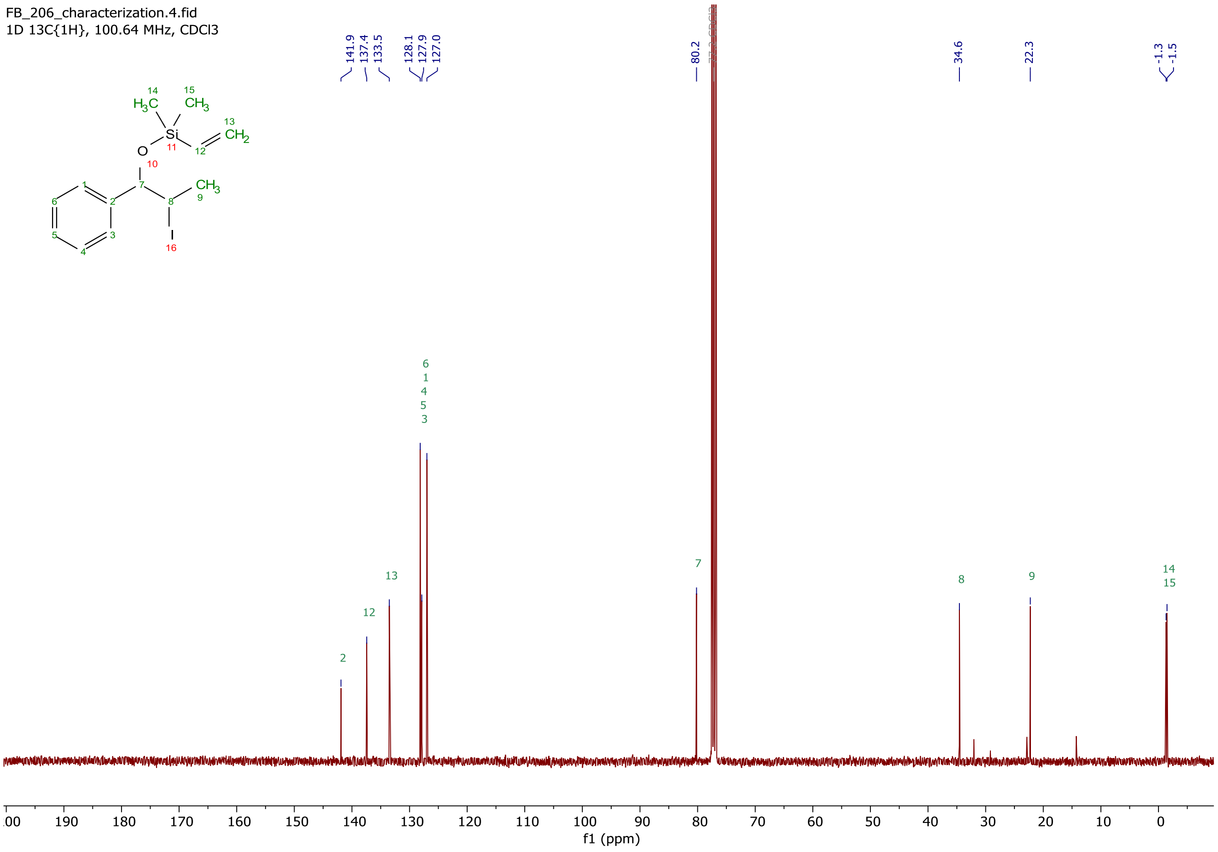
FB_206_characterization.4.fid
1D 13C{1H}, 100.64 MHz, CDCl3
141.9
137.4
133.5
128.1
127.9
127.0
80.2
34.6
22.3
-1.3
-1.5
f1 (ppm)

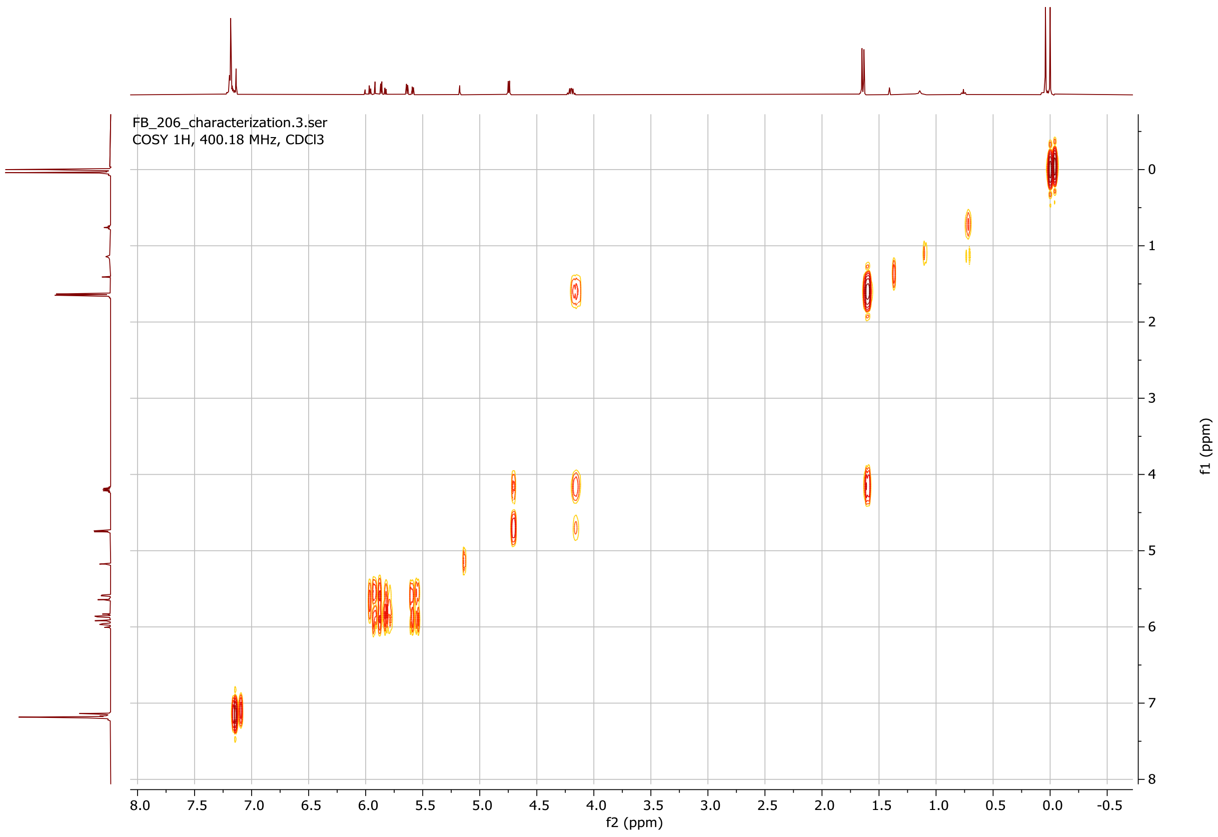
FB_206_characterization.3.ser
COSY 1H, 400.18 MHz, CDCl3
f2 (ppm)
f1 (ppm)

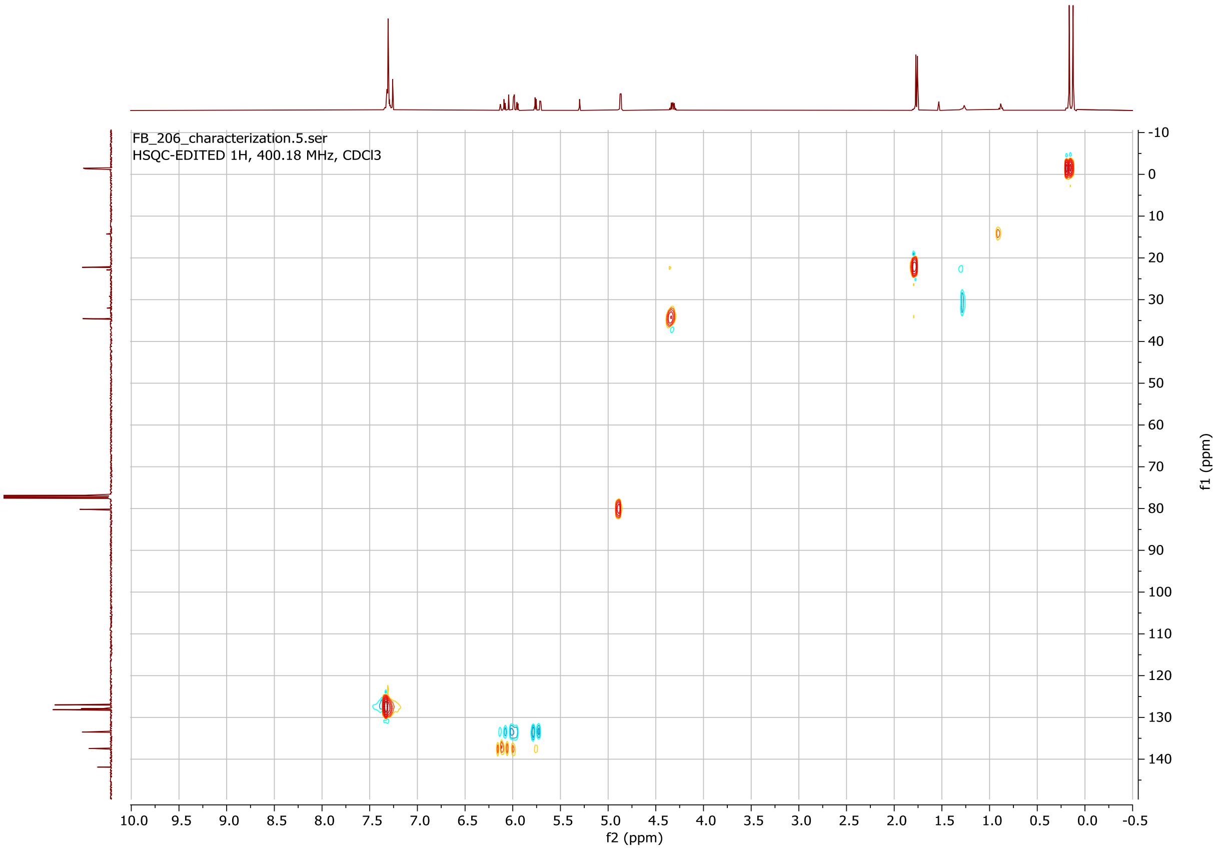

FB_206_characterization.5.ser
HSQC-EDITED 1H, 400.18 MHz, CDCl3
f2 (ppm)
f1 (ppm)

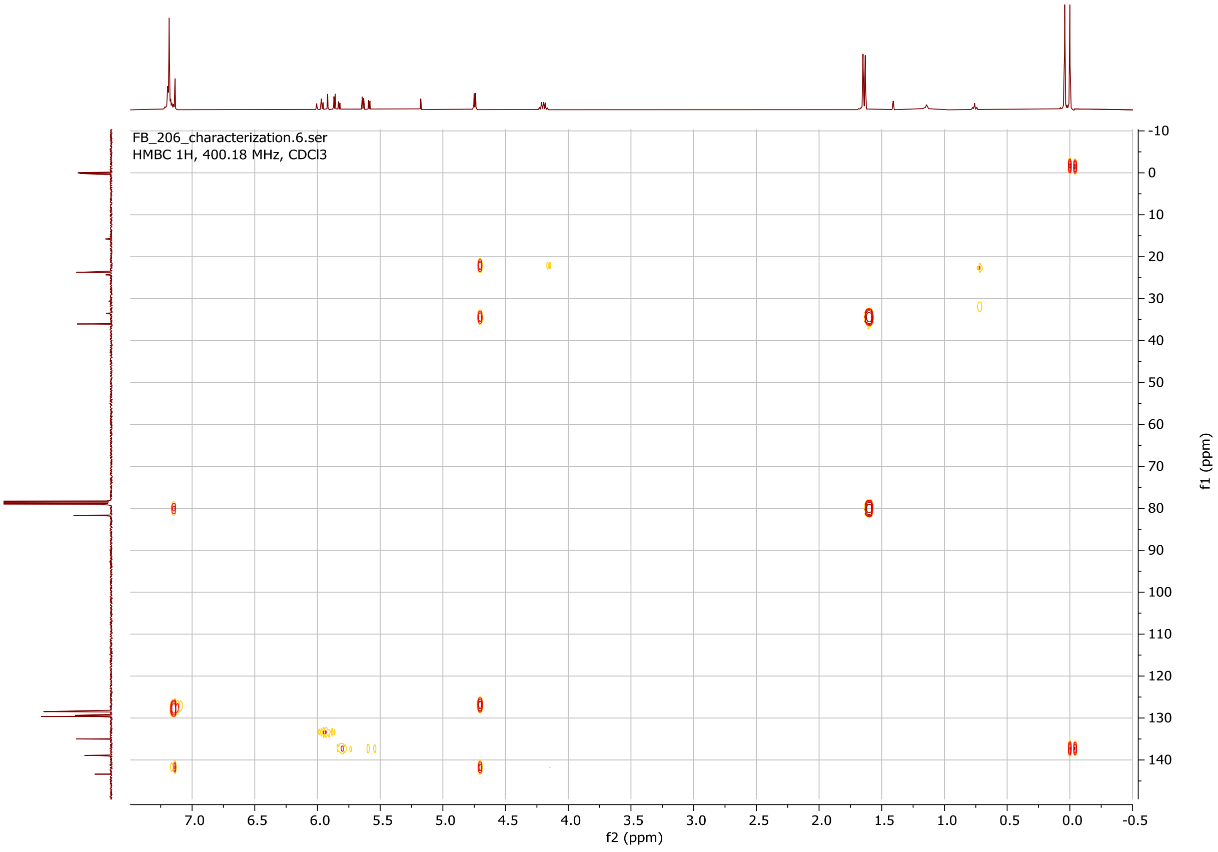

FB_206_characterization.6.ser
HMBC 1H, 400.18 MHz, CDCl3
f2 (ppm)
f1 (ppm)

((5-Iodooctan-4-yl)oxy)dimethylvinylsilane **4g**

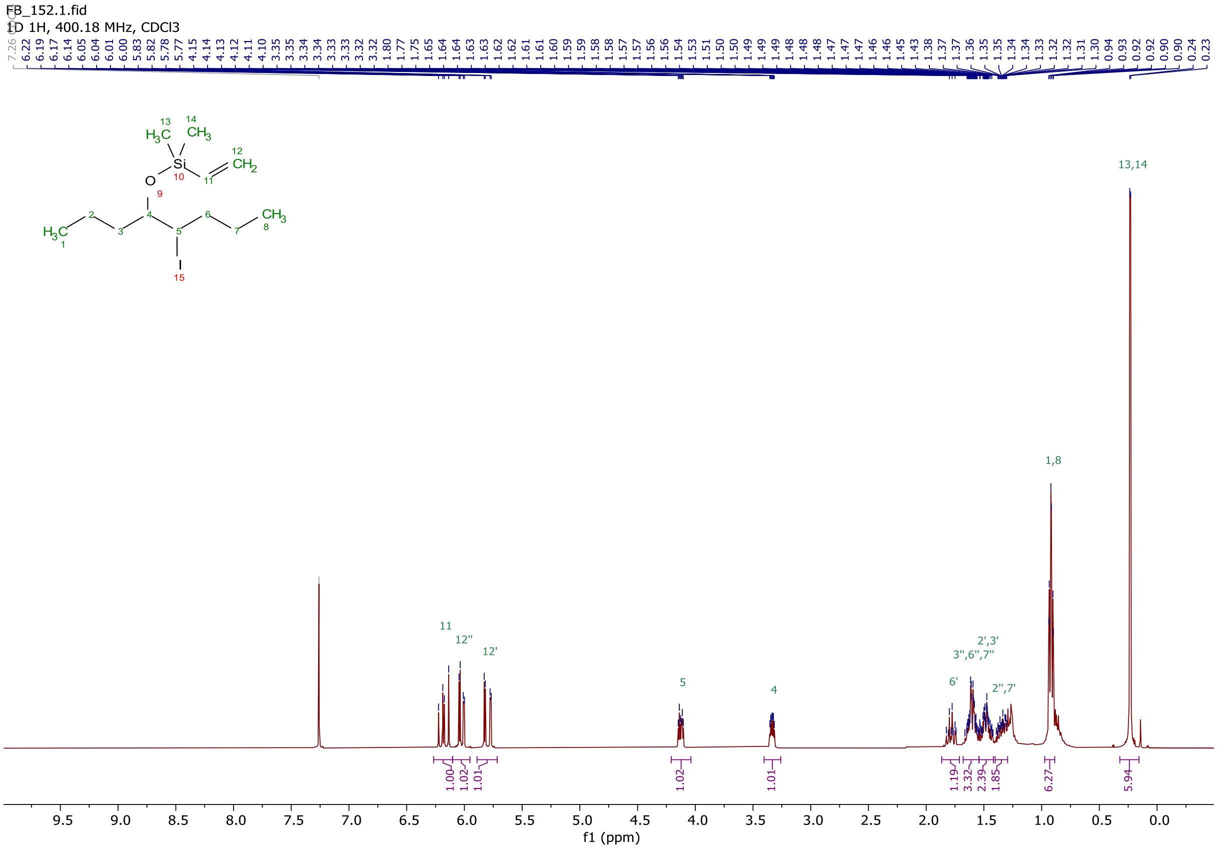

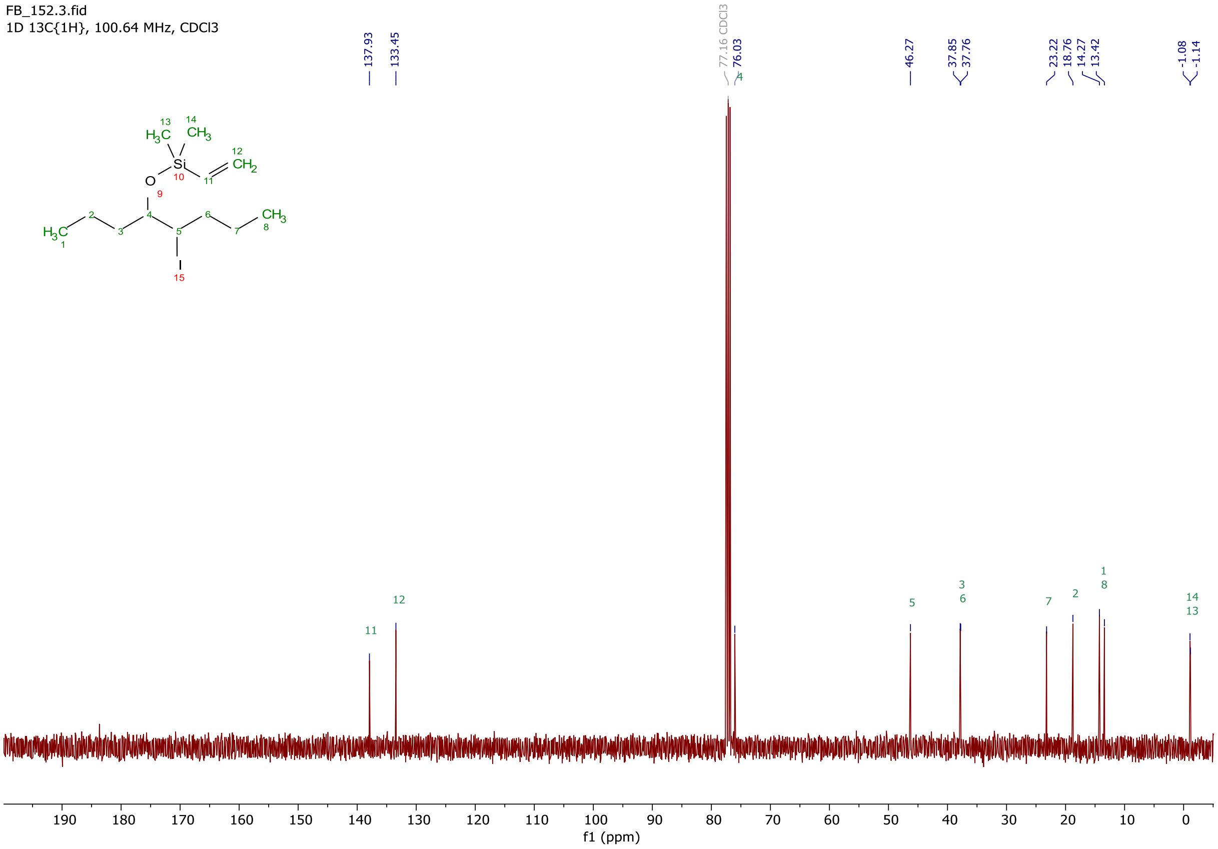

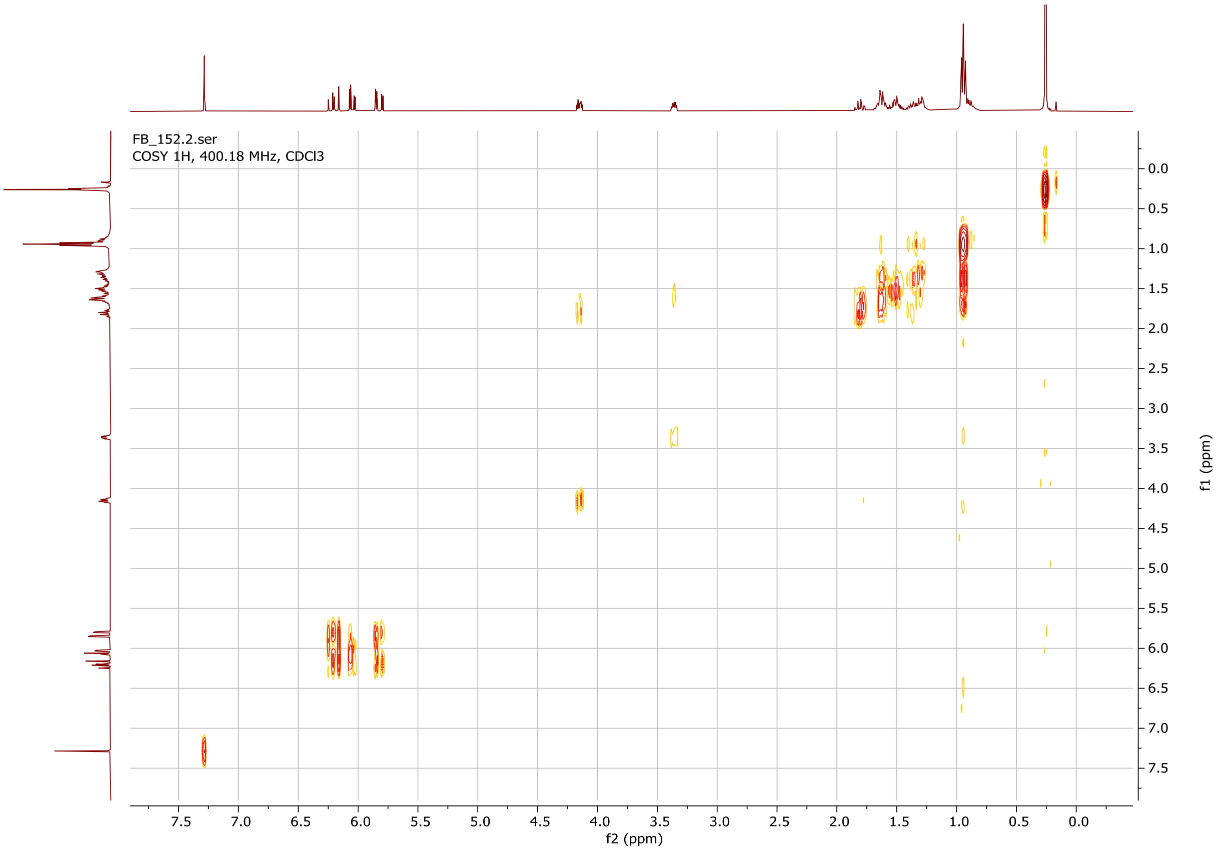

FB_152.2.ser
COSY 1H, 400.18 MHz, CDCl3
f2 (ppm)
f1 (ppm)

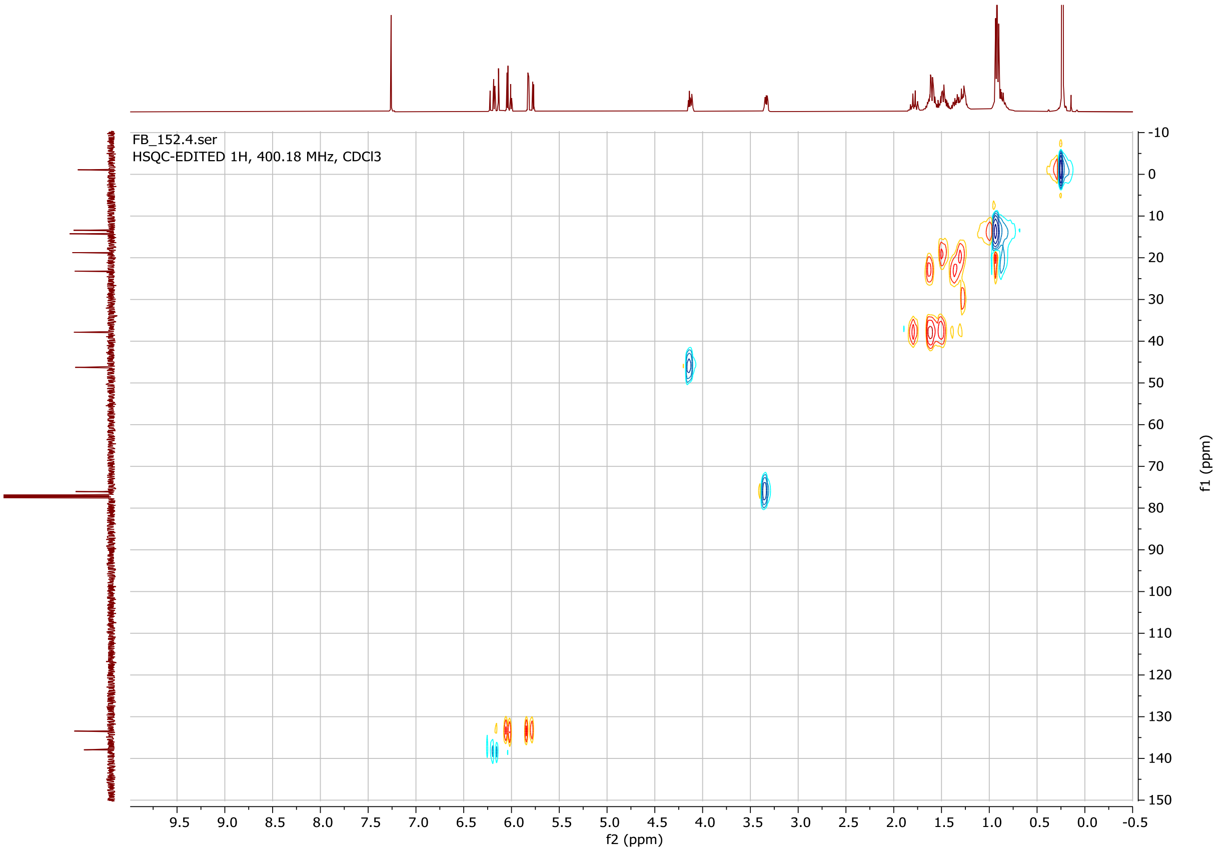

FB_152.4.ser
HSQC-EDITED 1H, 400.18 MHz, CDCl3
f2 (ppm)
f1 (ppm)

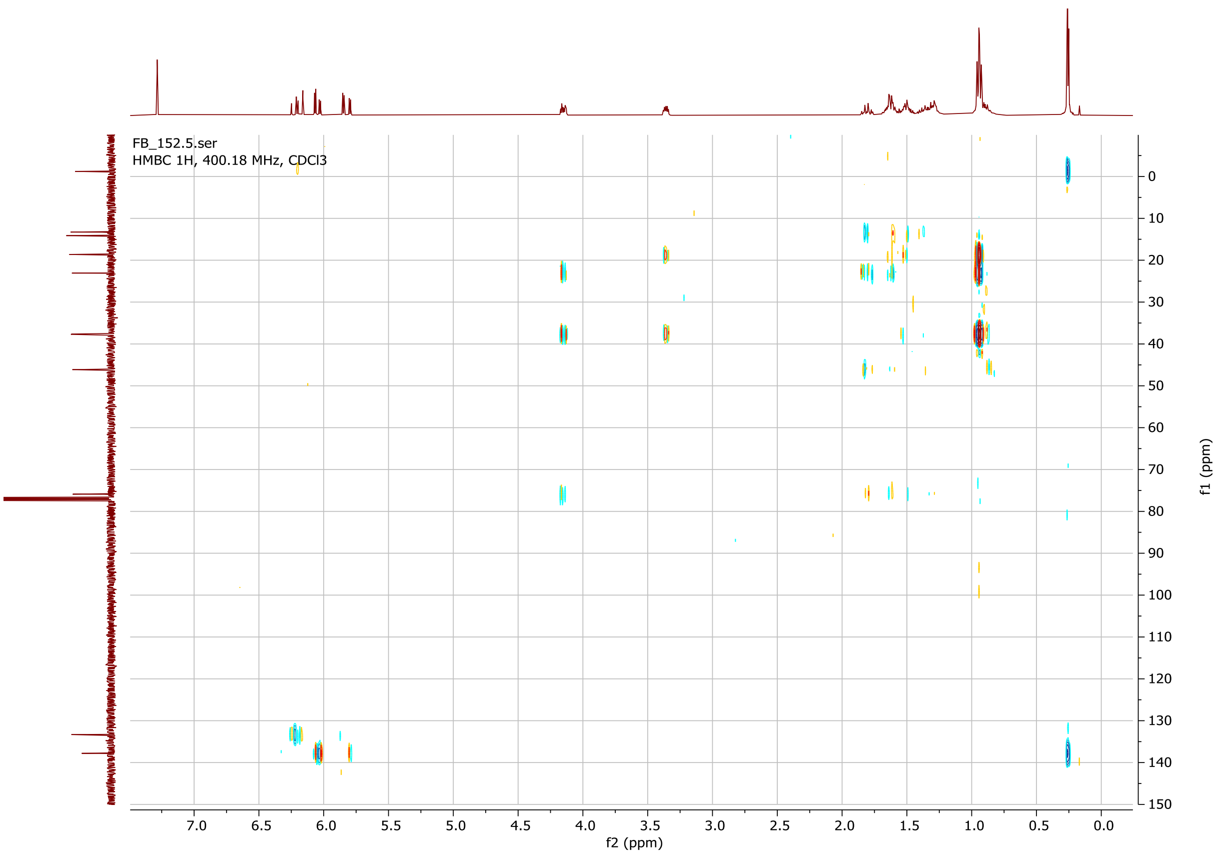

6-Iodo-3,3,9,9-tetramethyl-5-phenyl-4,8-dioxa-3,9-disilaundeca-1,10-diene **4h**

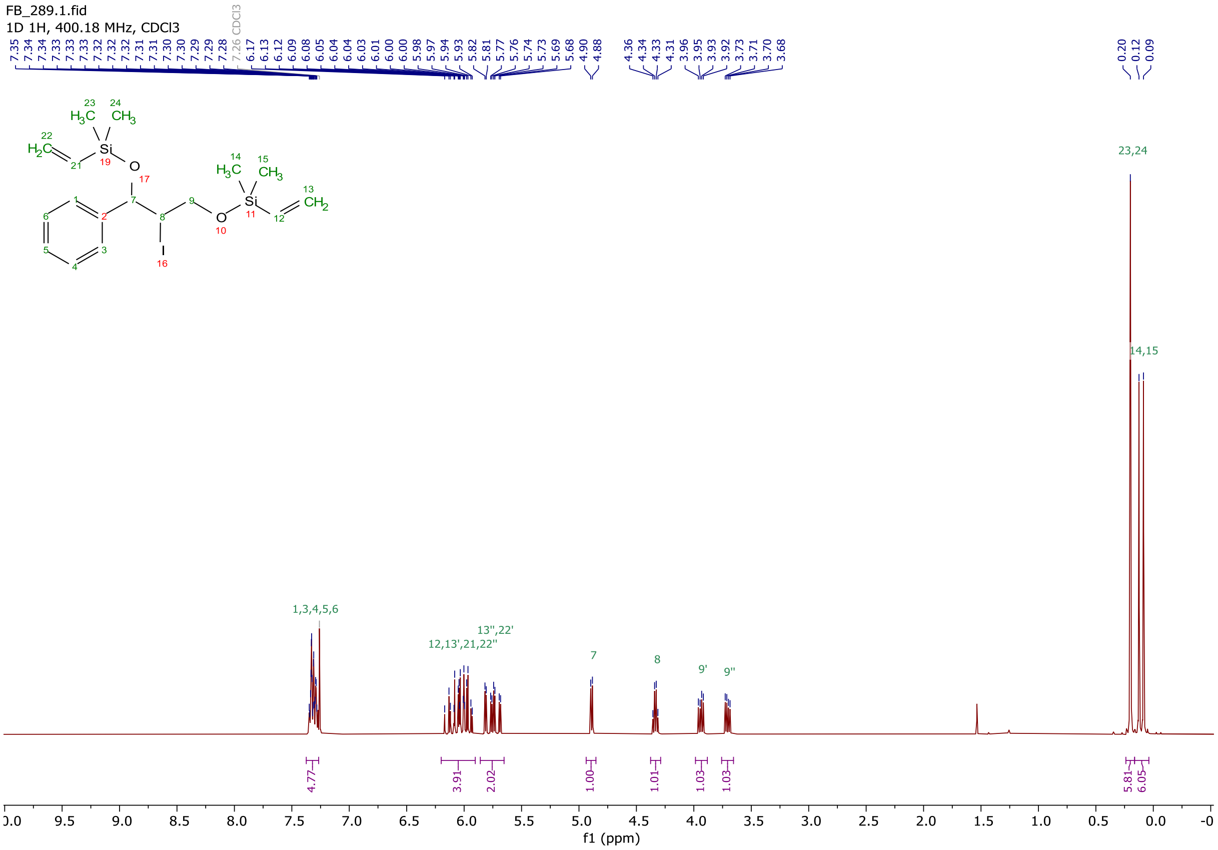

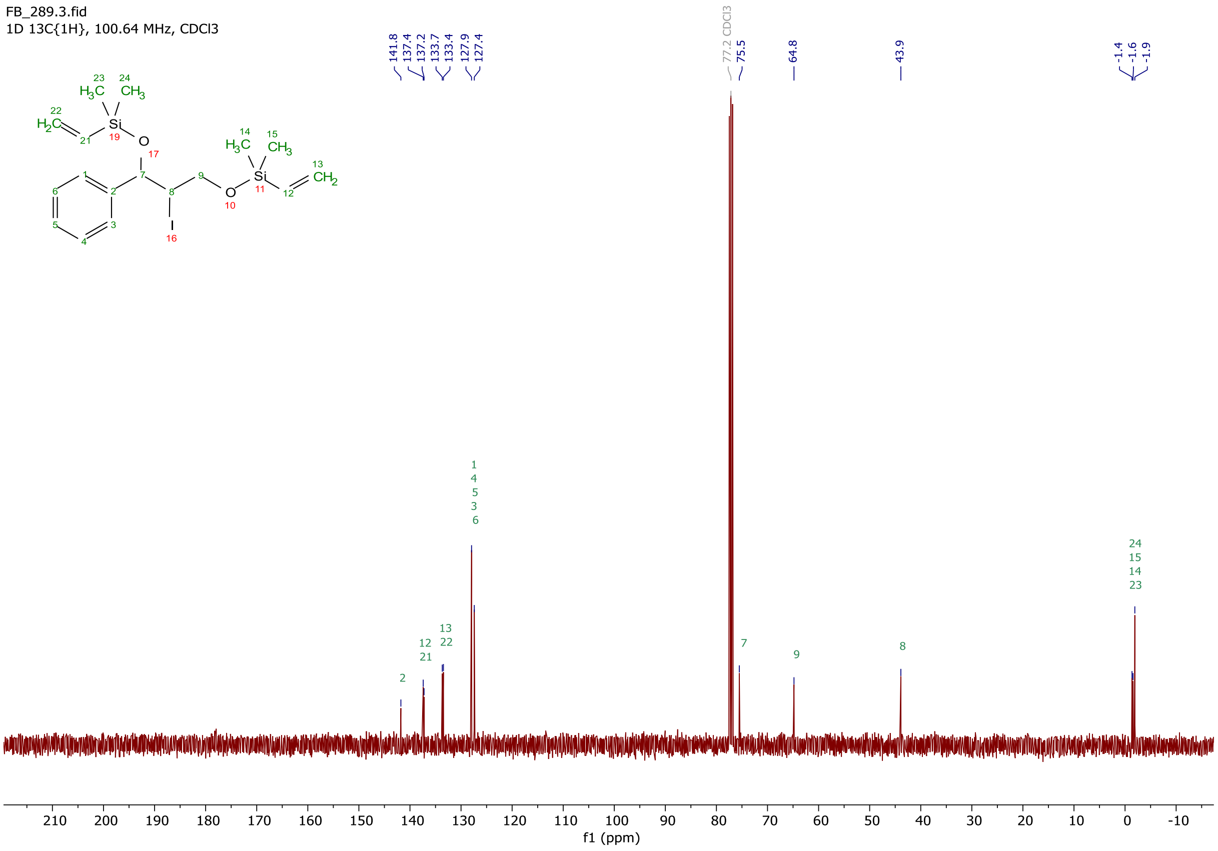
FB_289.3.fid
1D 13C{1H}, 100.64 MHz, CDCl3
141.8
137.4
137.2
133.7
133.4
127.9
127.4
77.2 CDCl3
75.5
64.8
43.9
-1.4
-1.6
-1.9
f1 (ppm)

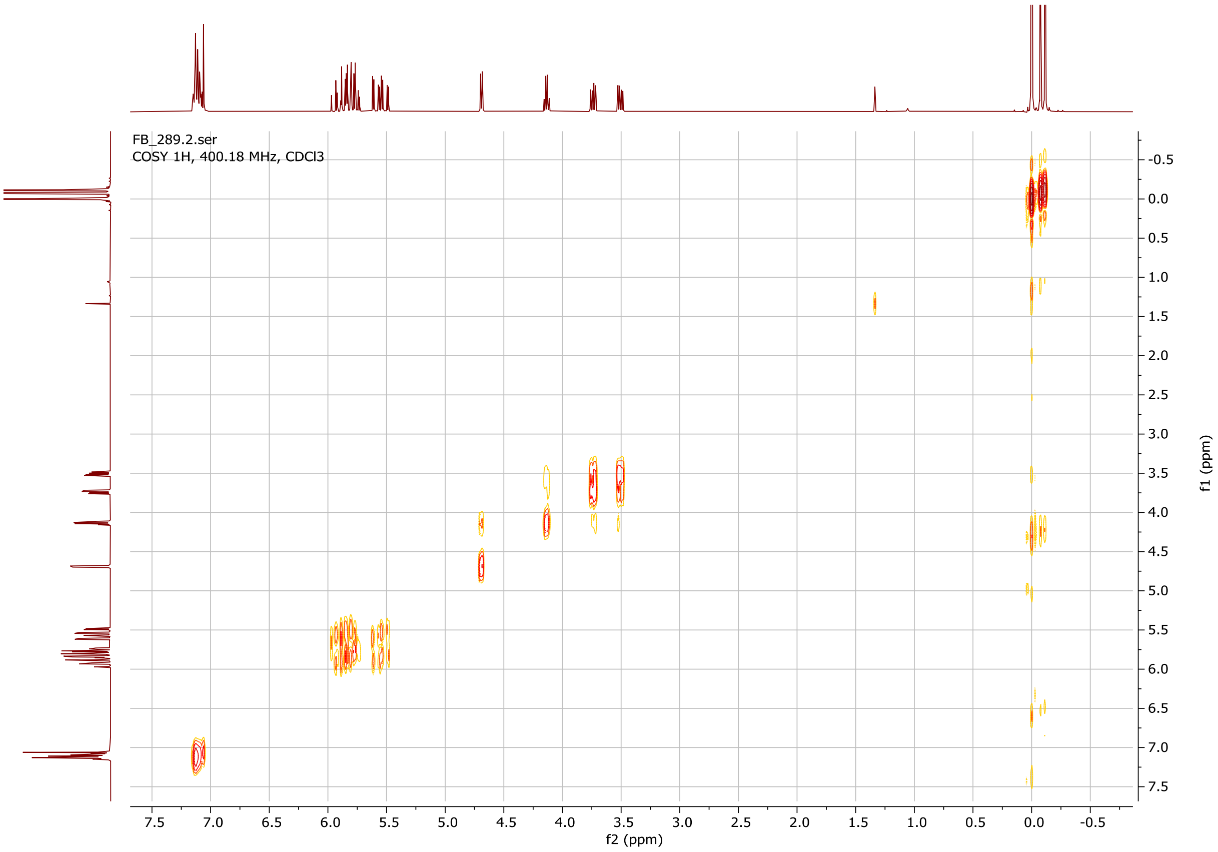
FB_289.2.ser
COSY 1H, 400.18 MHz, CDCl3
f2 (ppm)
f1 (ppm)

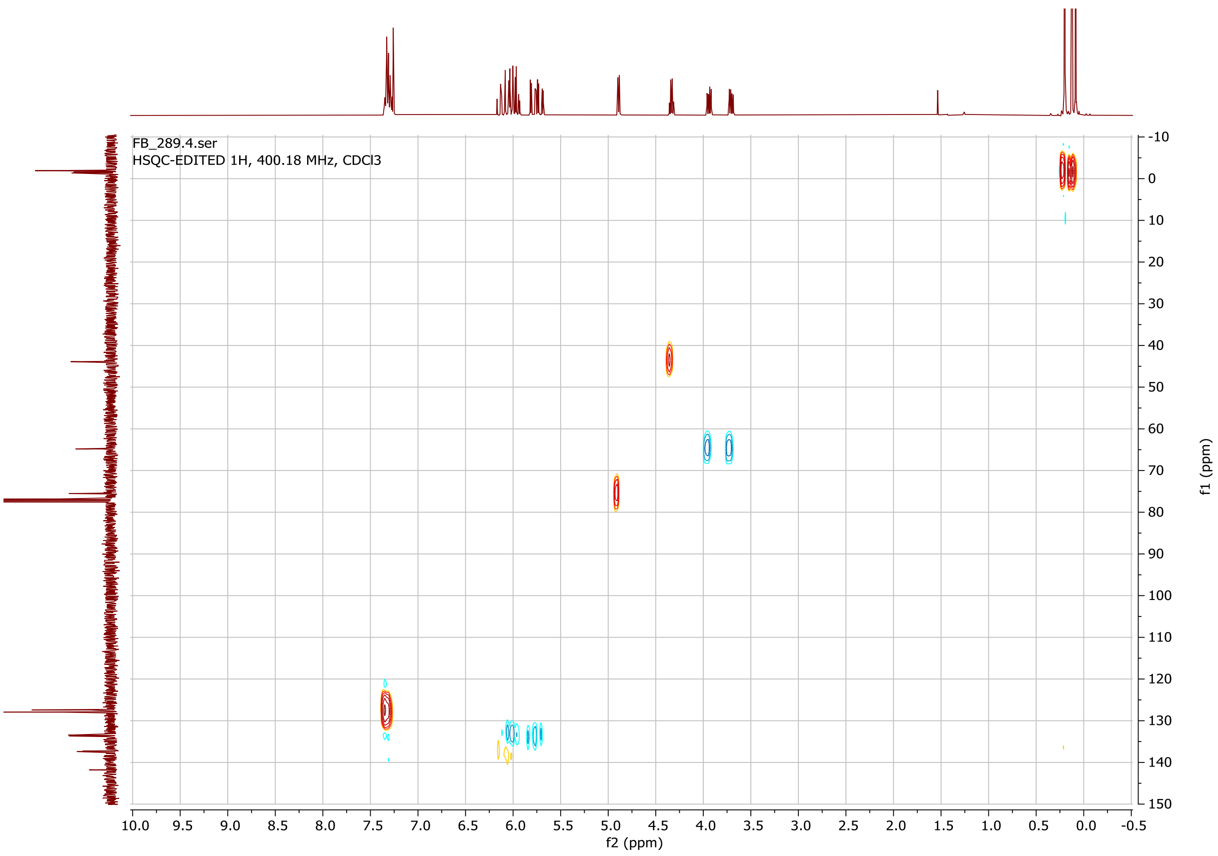
FB_289.4.ser
HSQC-EDITED 1H, 400.18 MHz, CDCl3
f2 (ppm)
f1 (ppm)

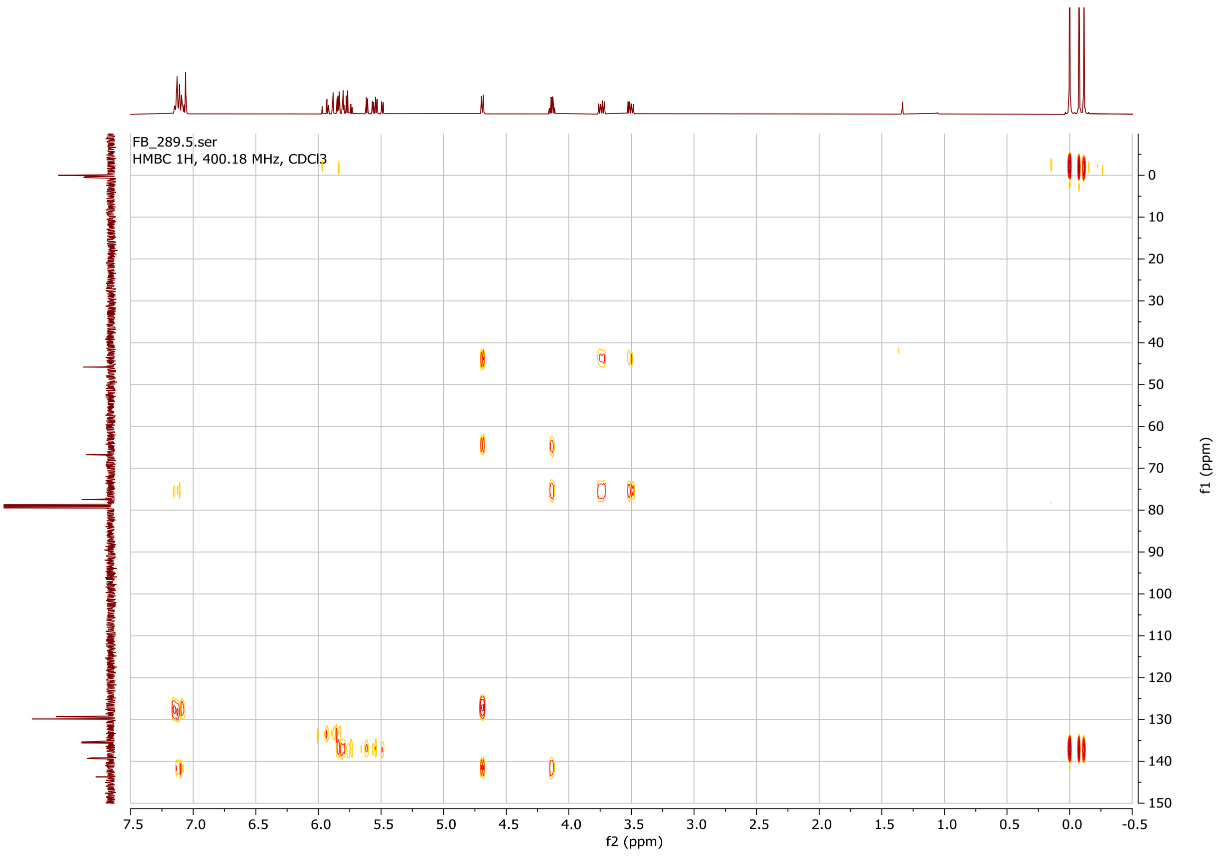
FB_289.5.ser
HMBC 1H, 400.18 MHz, CDCl3
f2 (ppm)
f1 (ppm)

(1-((*tert*-Butyldimethylsilyl)oxy)-2-iodohexan-3-oxy)dimethylvinylsilane **4i**

FB_272.1.fid
1D 1H, 400.18 MHz, CDCl3

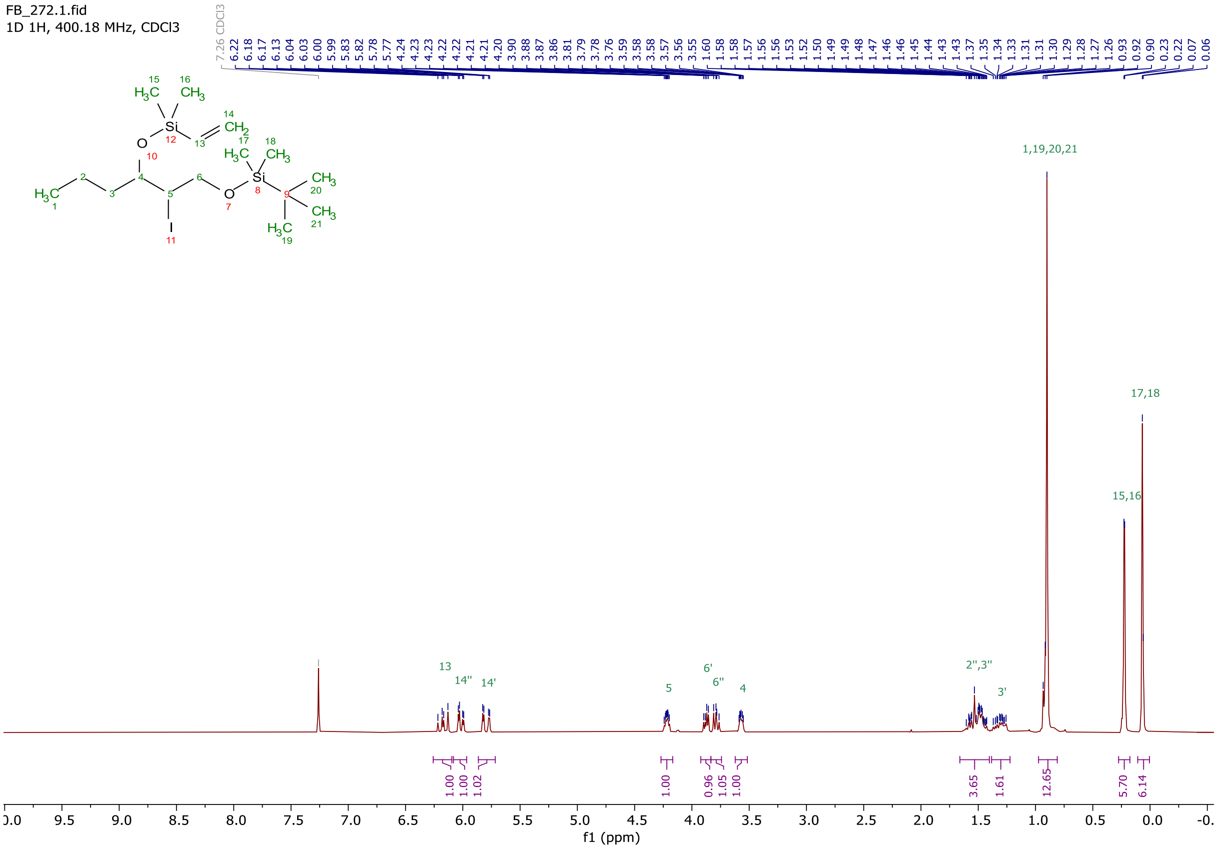

FB_272.3.fid
1D 13C{1H}, 100.64 MHz, CDCl3

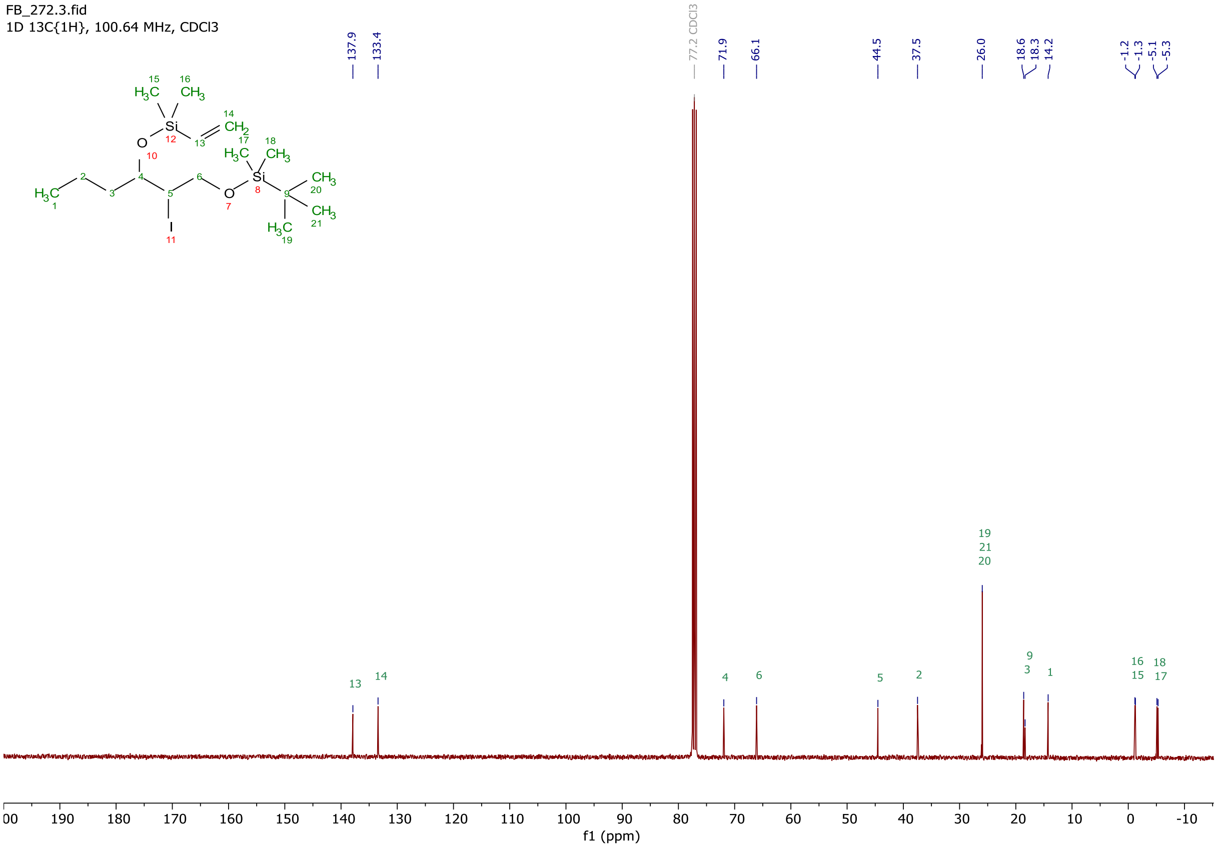

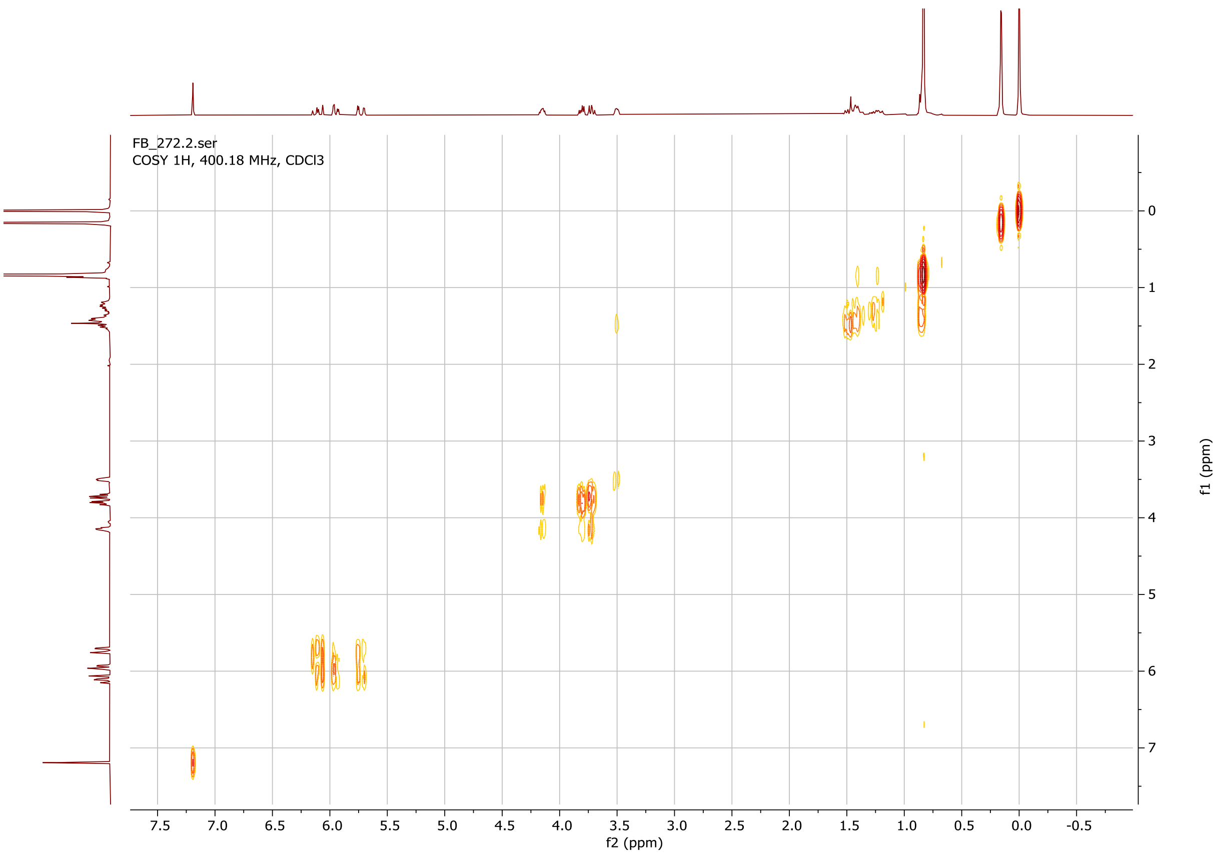
FB_272.2.ser
COSY 1H, 400.18 MHz, CDCl3
f2 (ppm)
f1 (ppm)

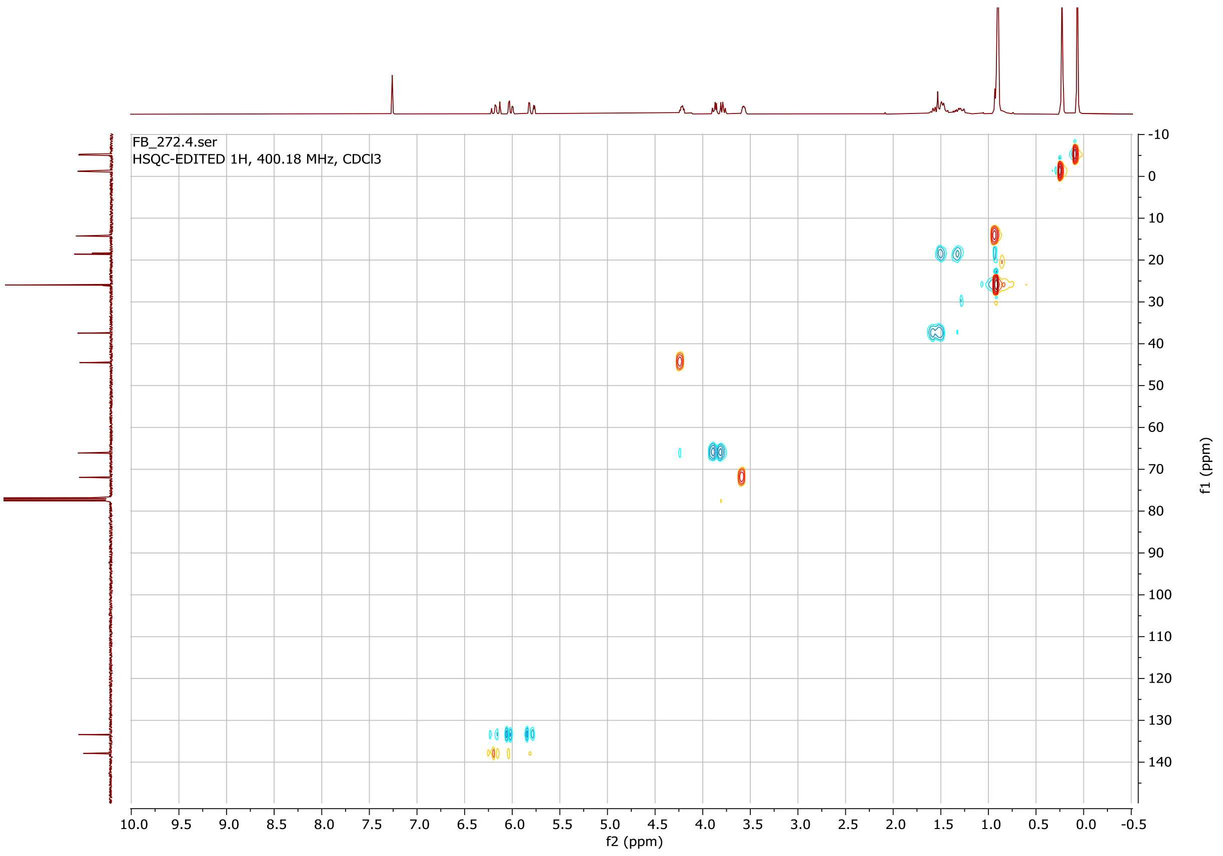
FB_272.4.ser
HSQC-EDITED 1H, 400.18 MHz, CDCl3
f2 (ppm)
f1 (ppm)

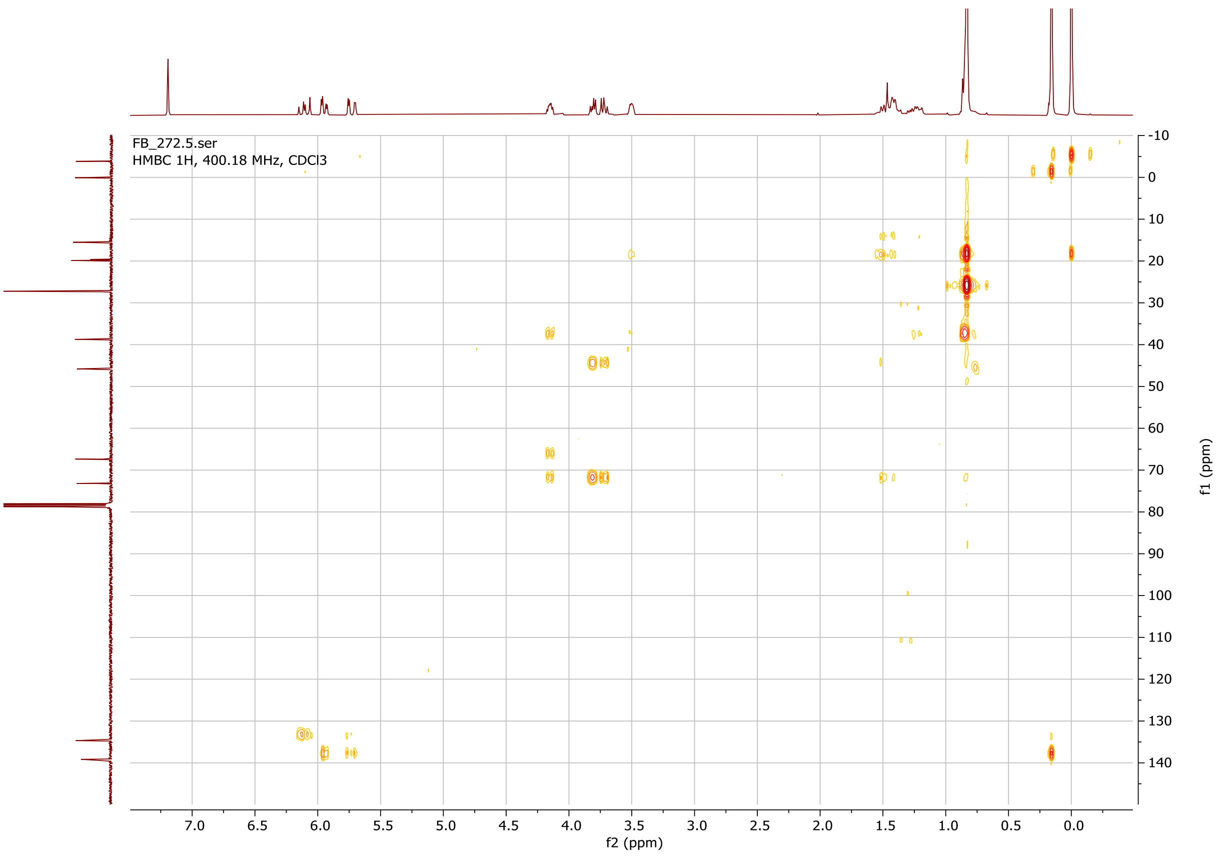

((2-Iodohexyl)oxy)dimethylvinylsilane **4j**

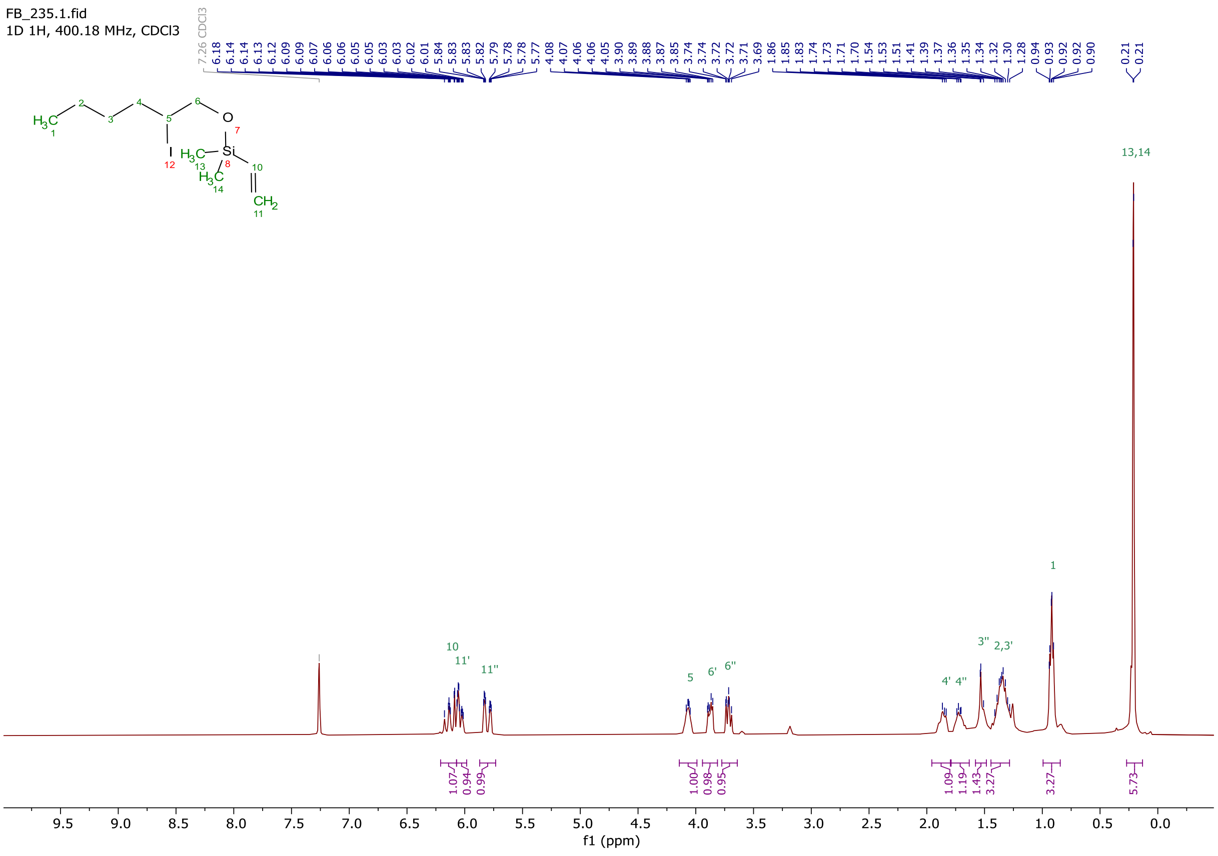

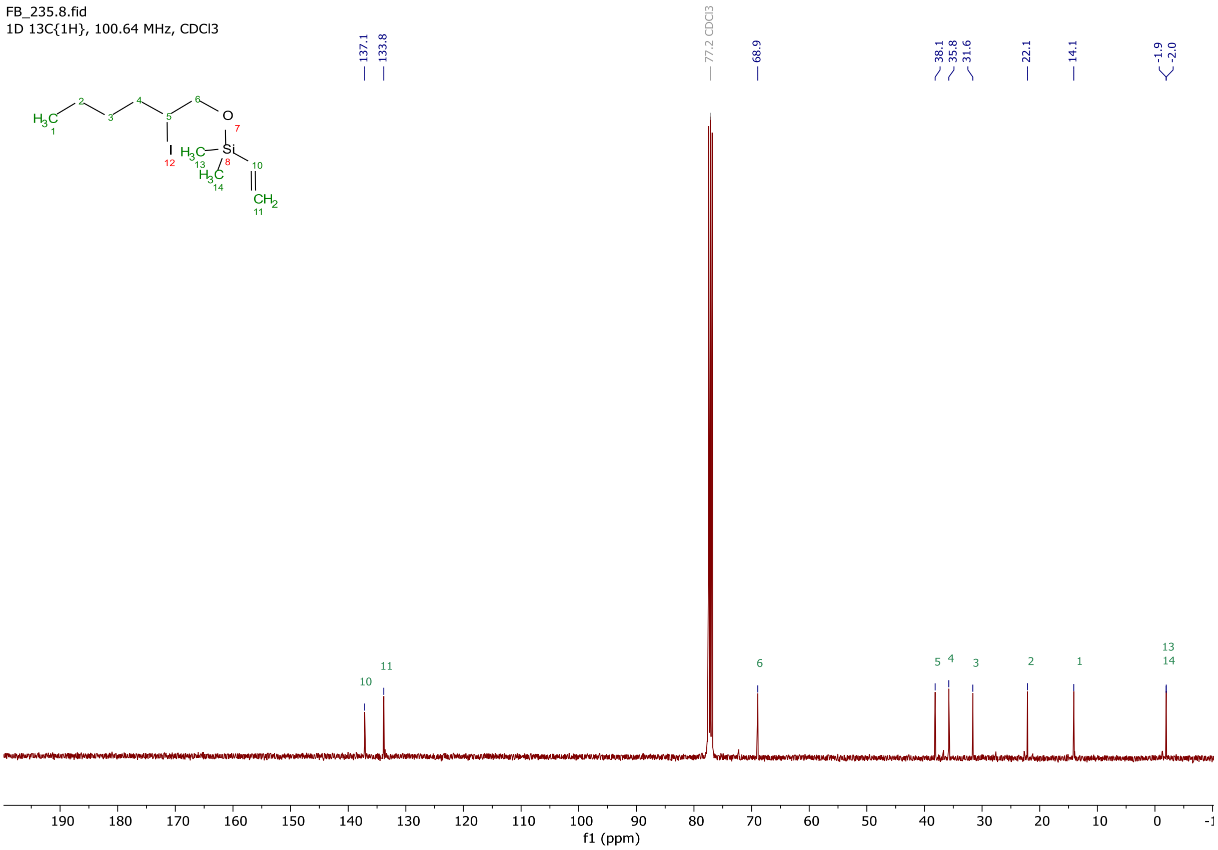
FB_235.8.fid
1D 13C{1H}, 100.64 MHz, CDCl3
137.1
133.8
77.2 CDCl3
68.9
38.1
35.8
31.6
22.1
14.1
-1.9
-2.0
f1 (ppm)

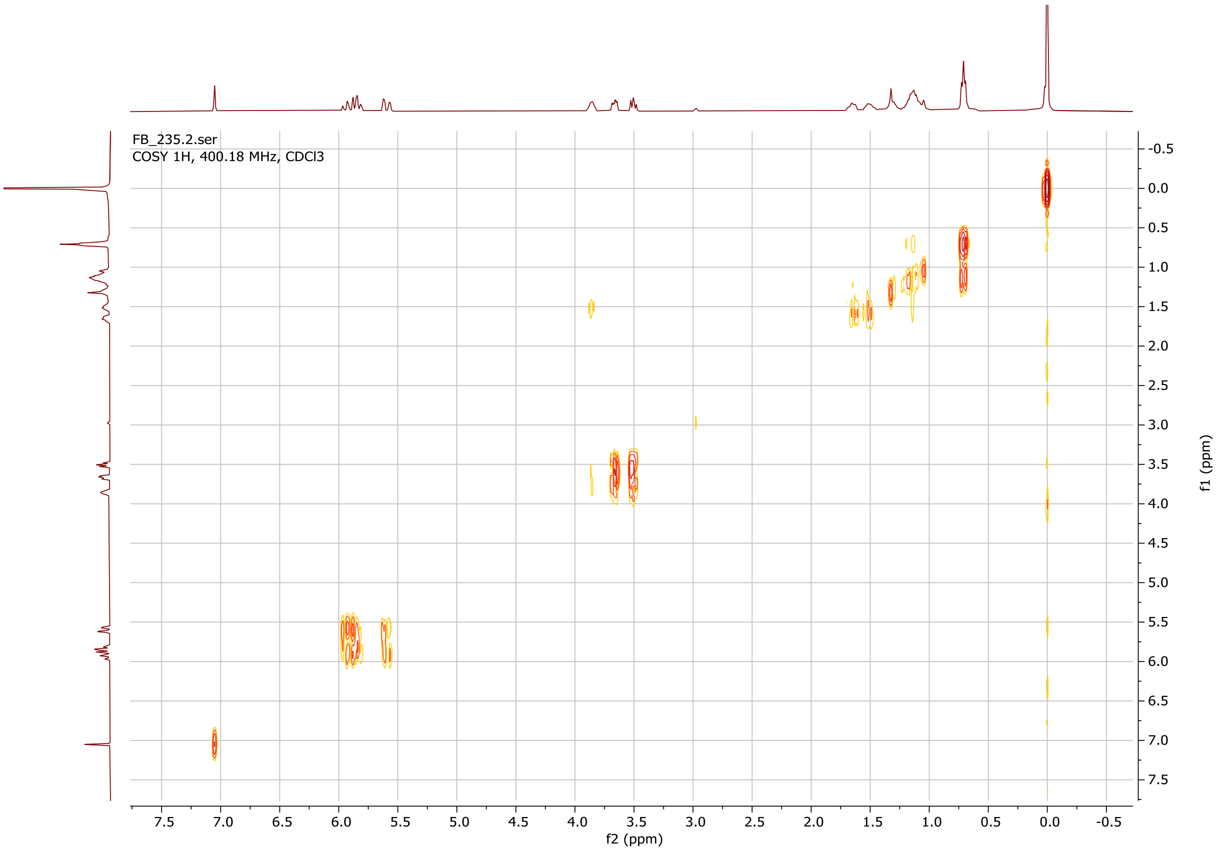
FB_235.2.ser
COSY 1H, 400.18 MHz, CDCl3
f2 (ppm)
f1 (ppm)

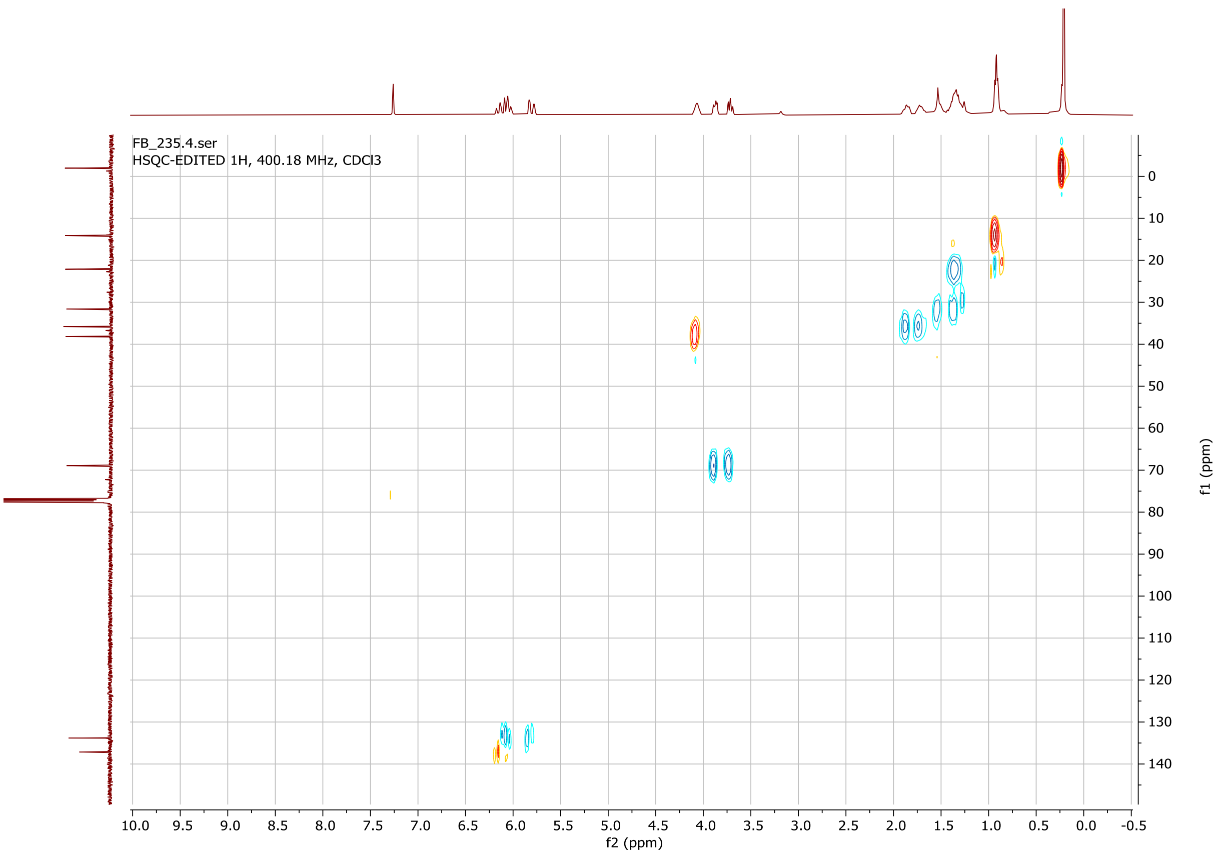
FB_235.4.ser
HSQC-EDITED 1H, 400.18 MHz, CDCl3
f2 (ppm)
f1 (ppm)

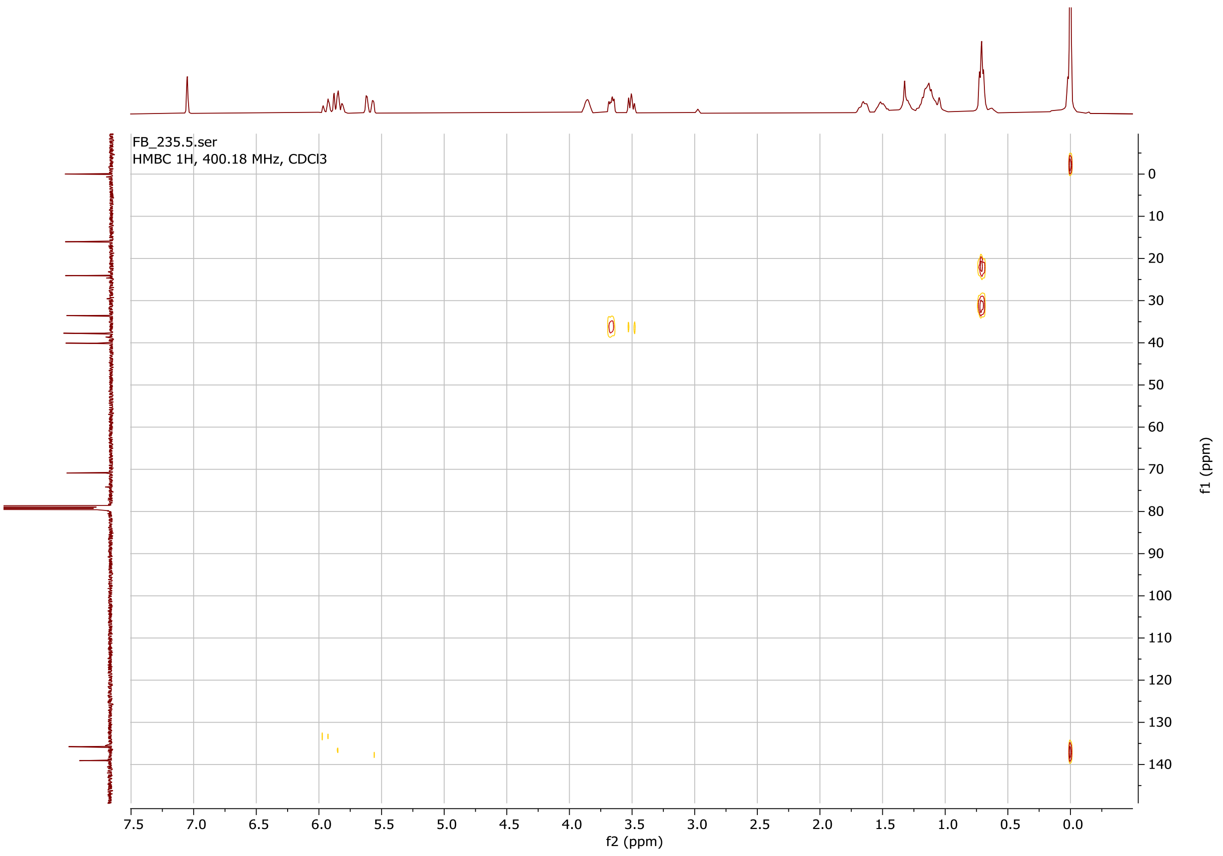
FB_235.5.ser
HMBC 1H, 400.18 MHz, CDCl3
f2 (ppm)
f1 (ppm)

((1-Iodohexan-2-yl)oxy)dimethylvinylsilane **4k**

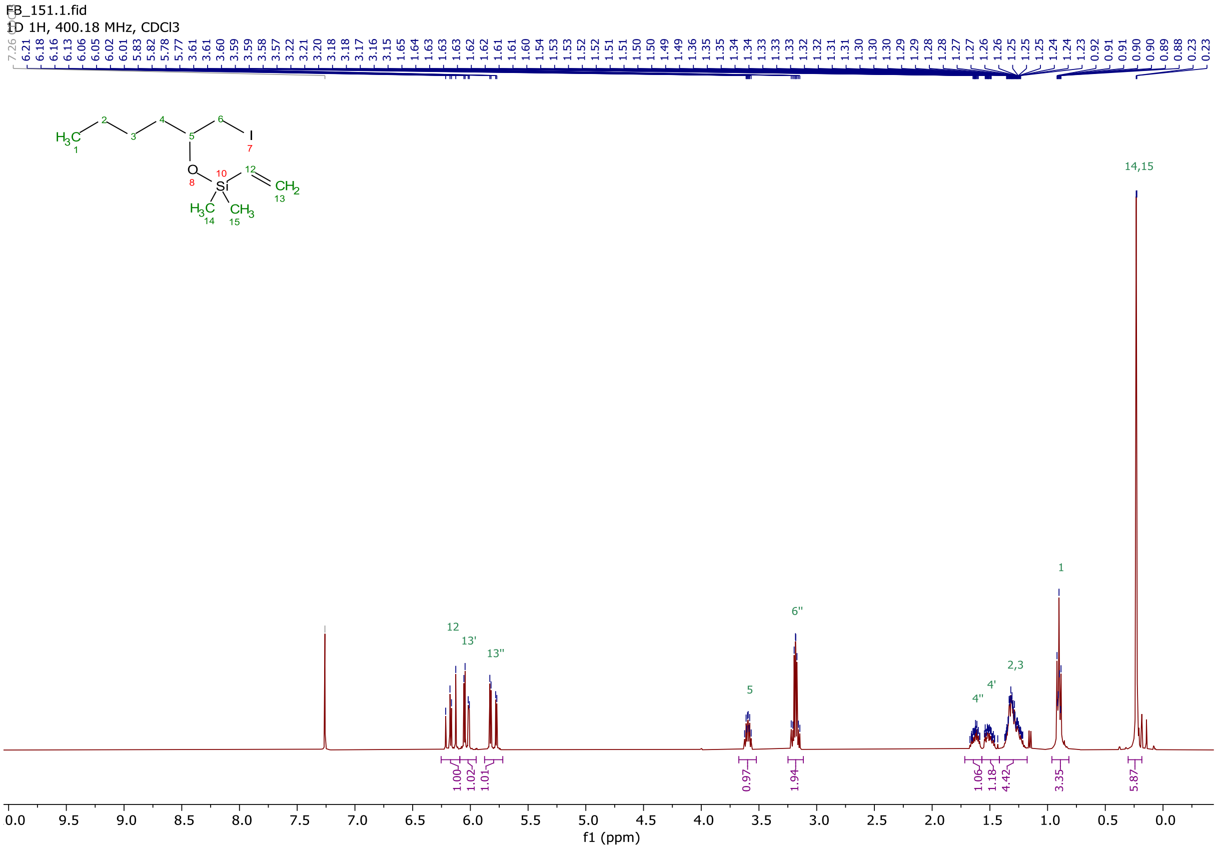

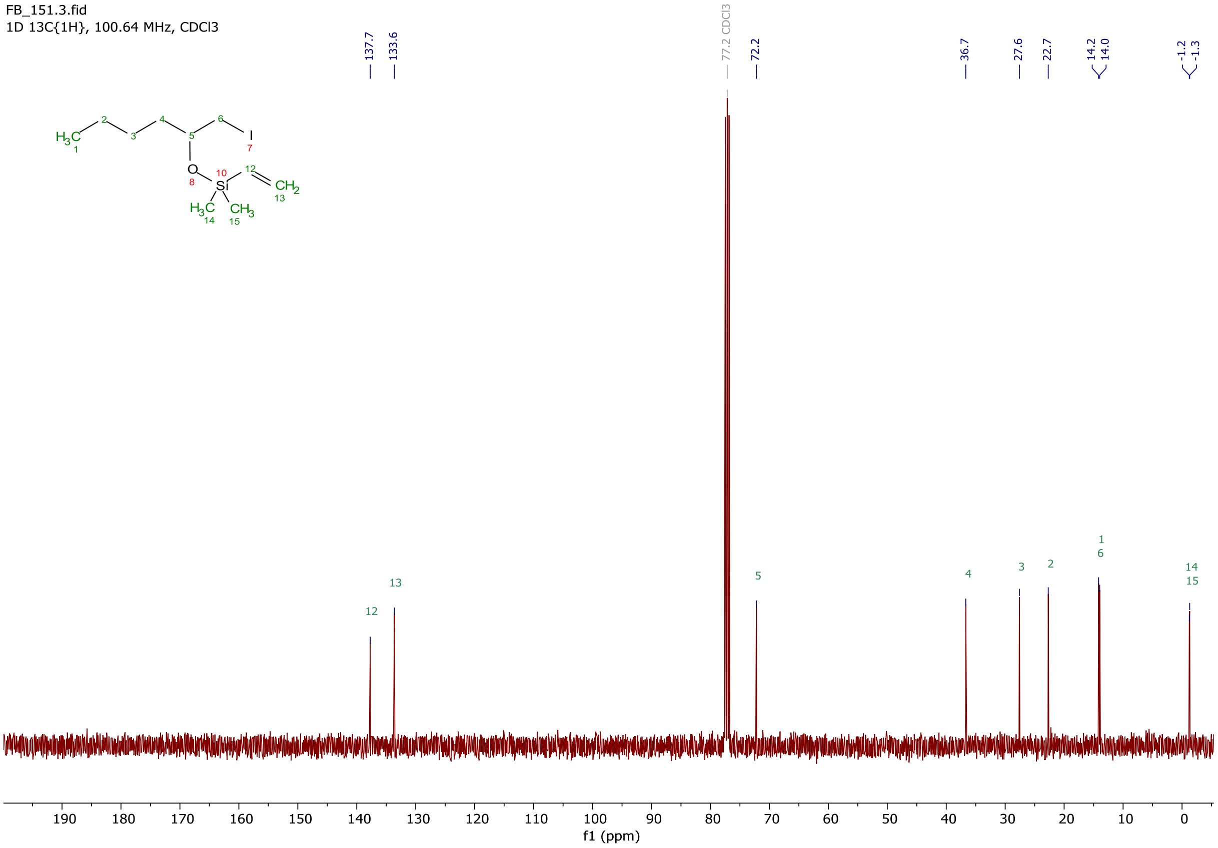

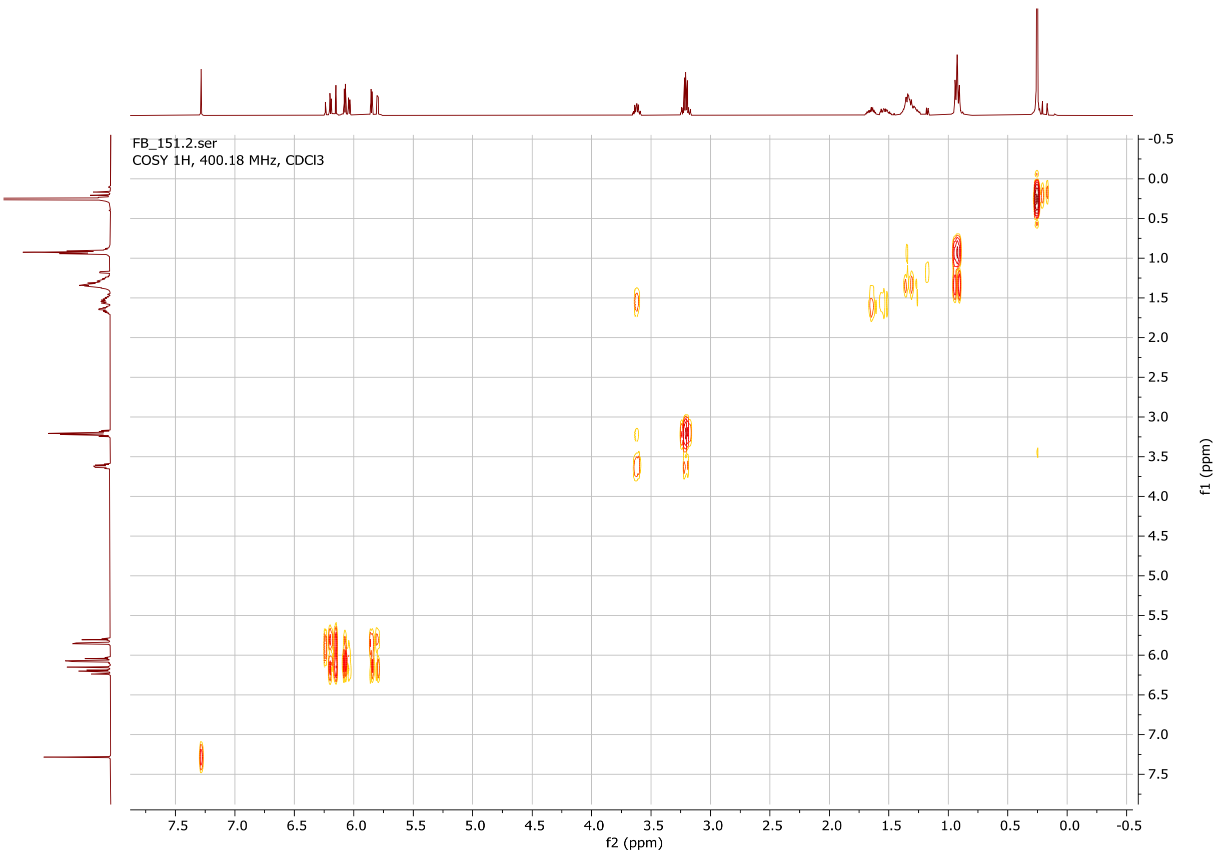
FB_151.2.ser
COSY 1H, 400.18 MHz, CDCl3
f2 (ppm)
f1 (ppm)

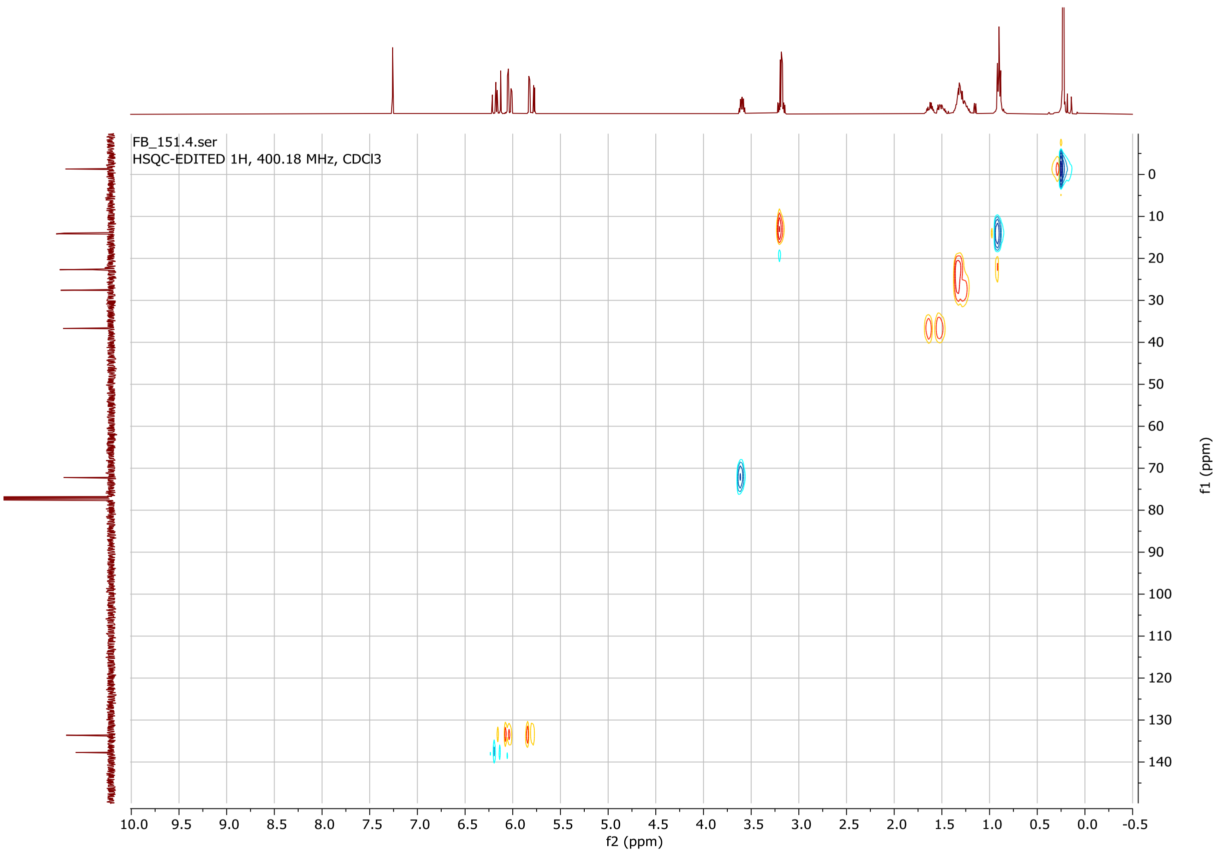
FB_151.4.ser
HSQC-EDITED 1H, 400.18 MHz, CDCl3
f2 (ppm)
f1 (ppm)

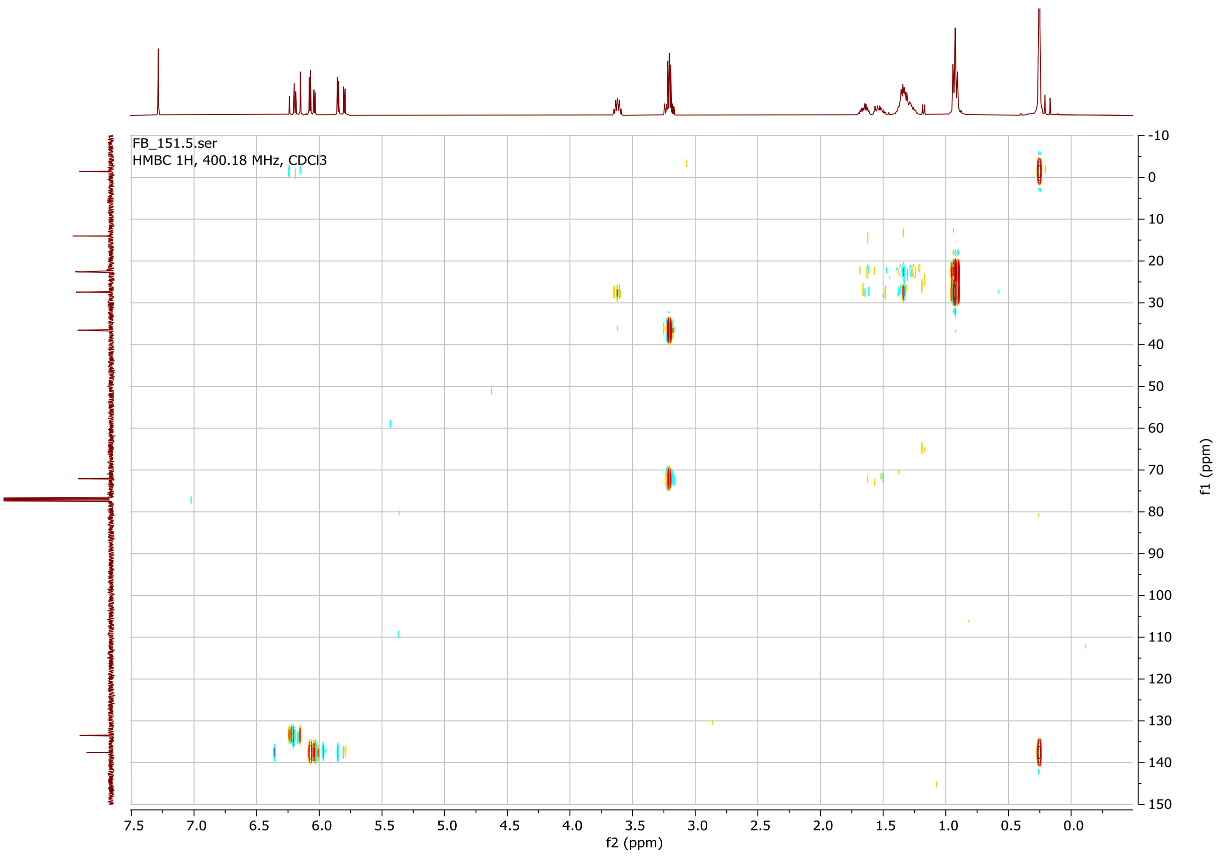

((1-Iodo-4-phenylbutan-2-yl)oxy)dimethylvinylsilane **4l**

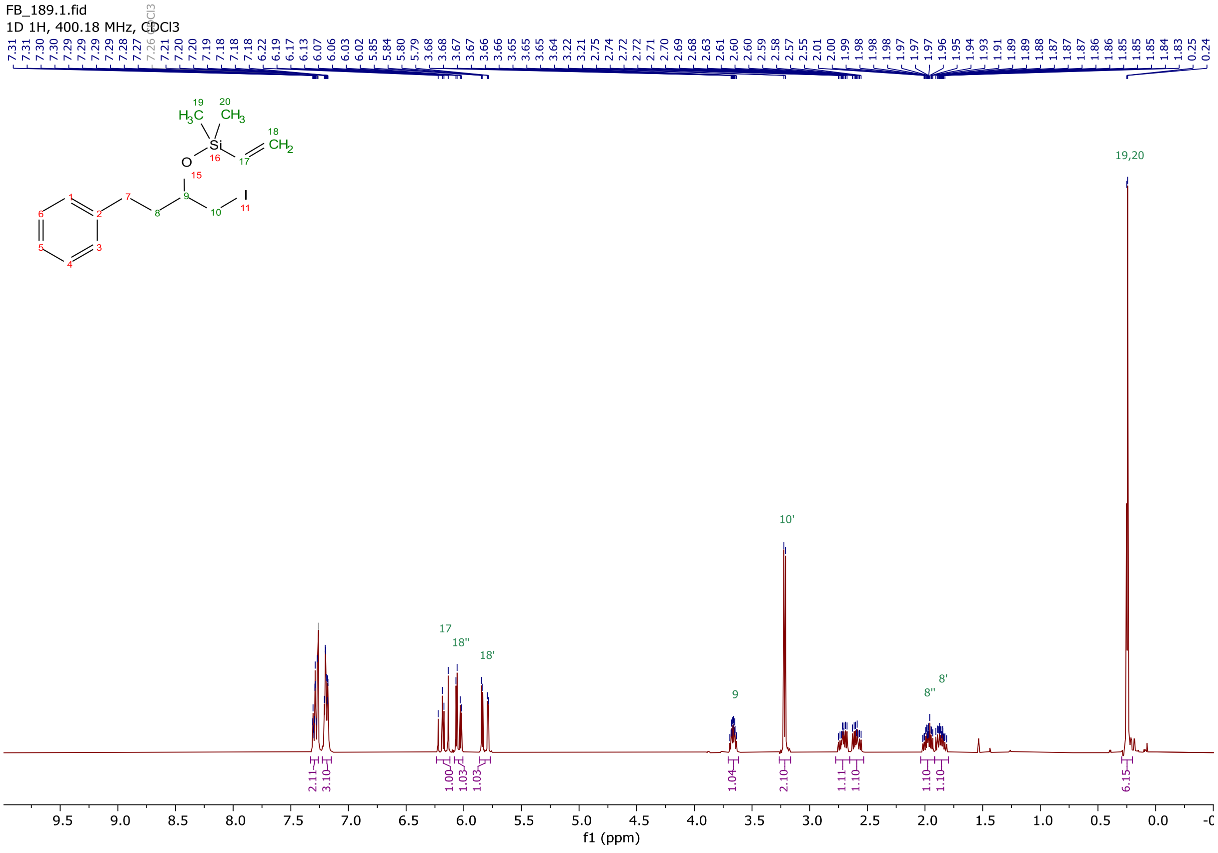

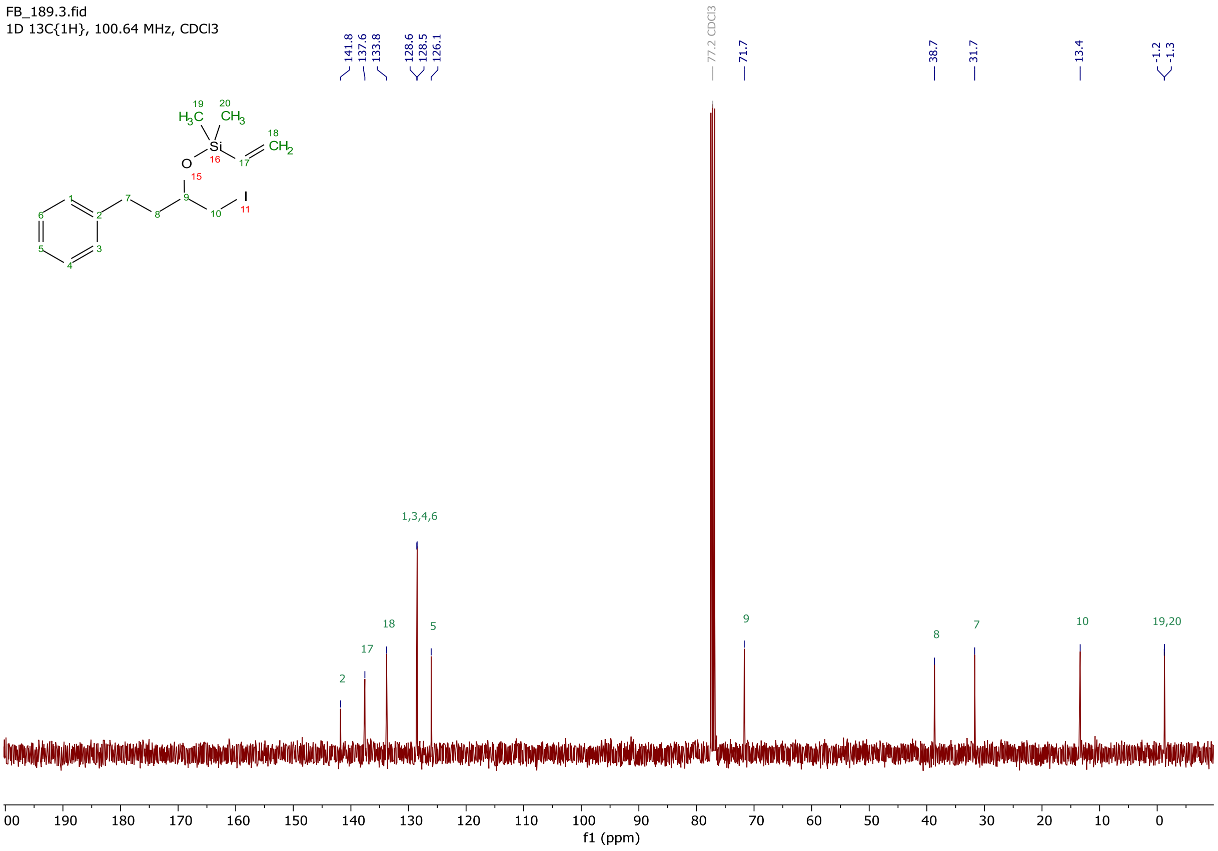
FB_189.3.fid
1D 13C{1H}, 100.64 MHz, CDCl3
141.8
137.6
133.8
128.6
128.5
126.1
77.2 CDCl3
71.7
38.7
31.7
13.4
-1.2
-1.3
1,3,4,6
18
17
2
5
9
8
7
10
19,20
f1 (ppm)

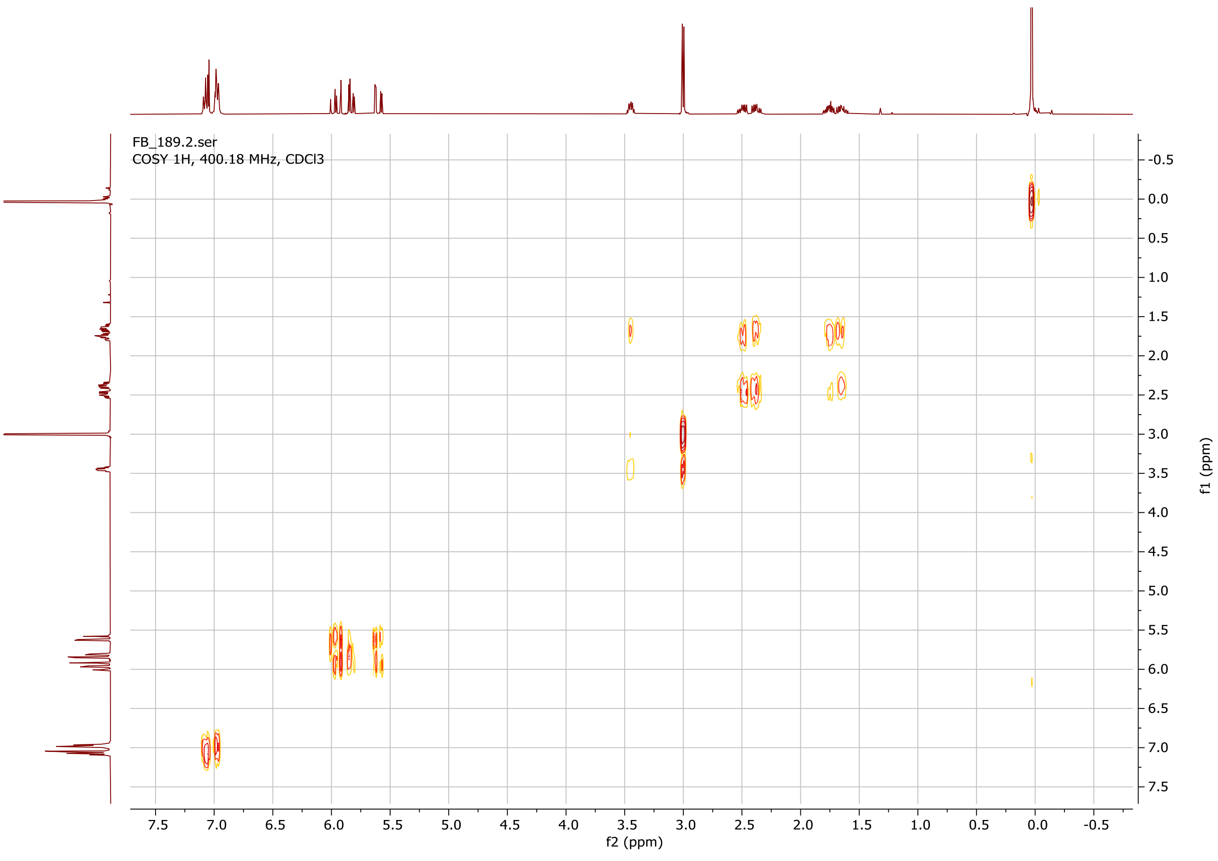
FB_189.2.ser
COSY 1H, 400.18 MHz, CDCl3
f2 (ppm)
f1 (ppm)

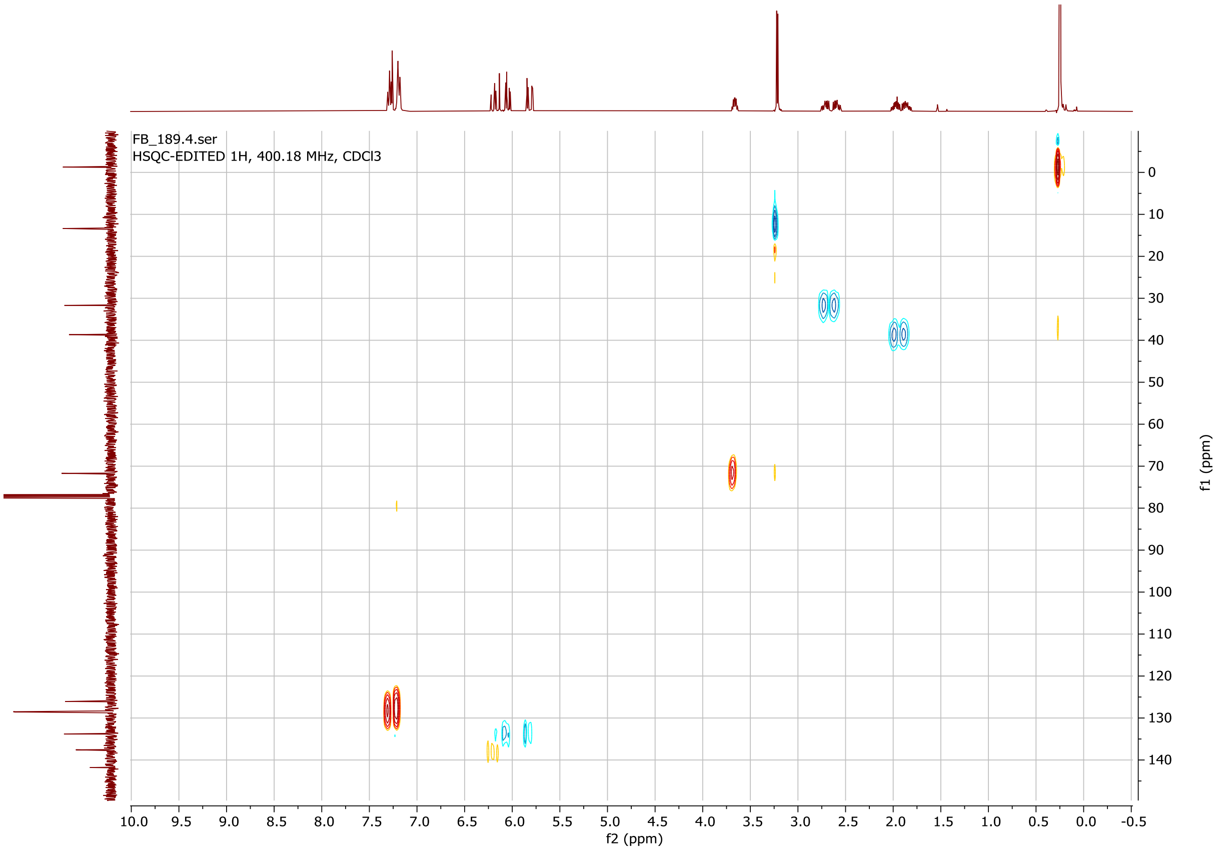
FB_189.4.ser
HSQC-EDITED 1H, 400.18 MHz, CDCl3
f2 (ppm)
f1 (ppm)

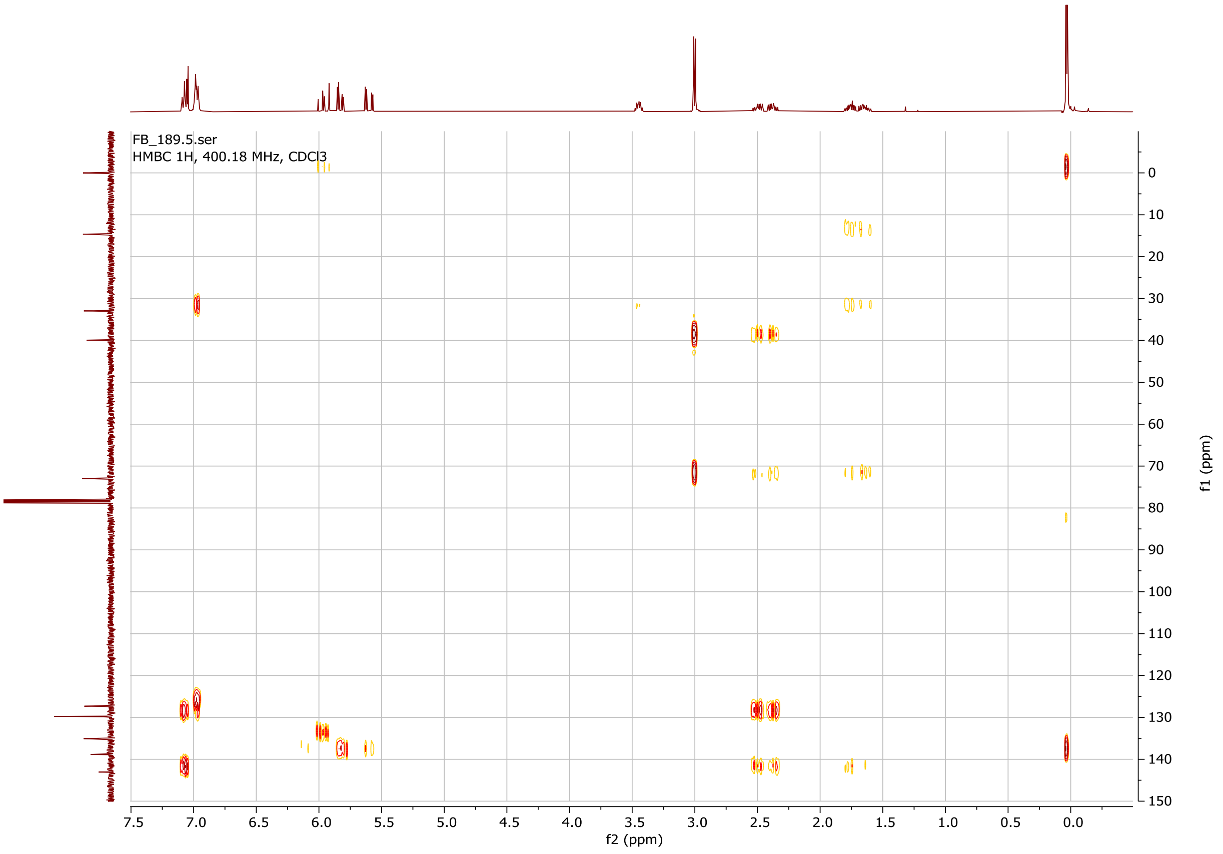
FB_189.5.ser
HMBC 1H, 400.18 MHz, CDCl3
f2 (ppm)
f1 (ppm)

Methyl 4-((dimethylvinylsilyl)oxy)-5-iodopentanoate **4m**

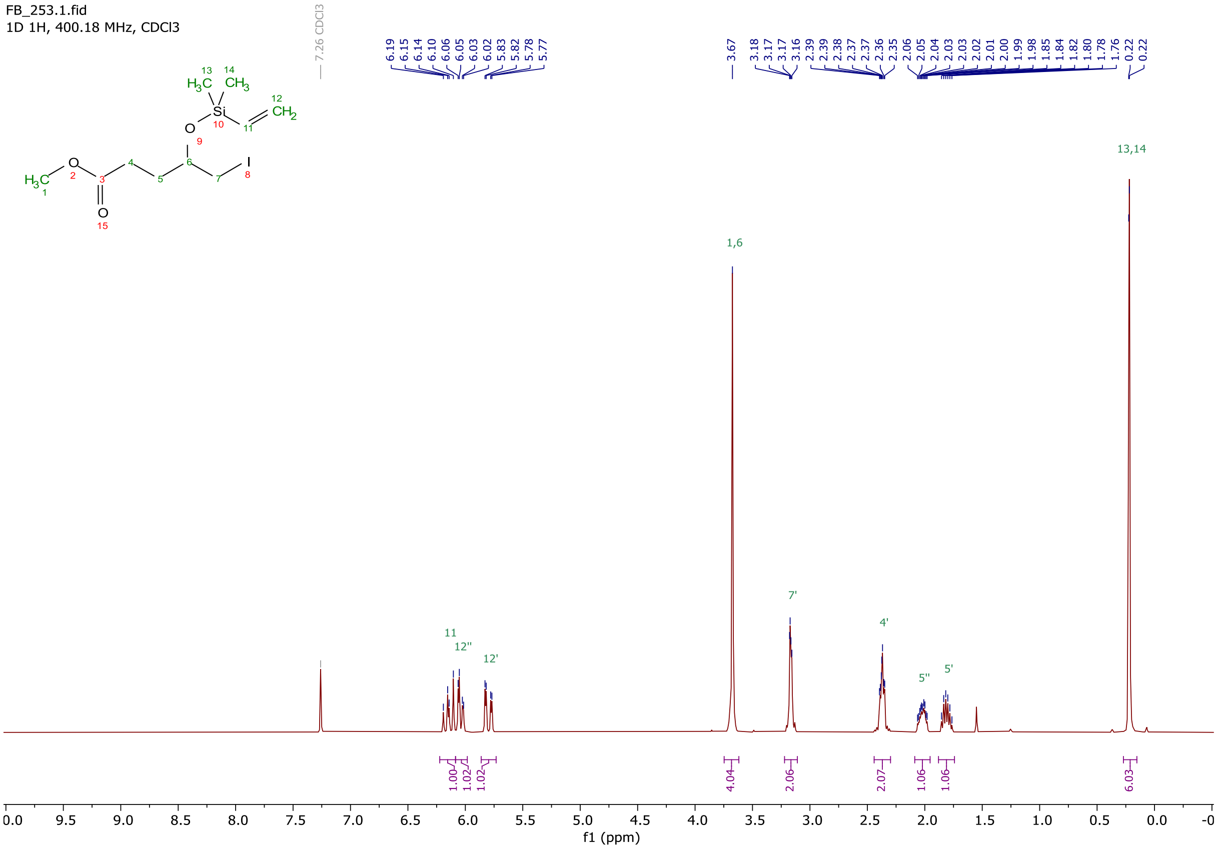

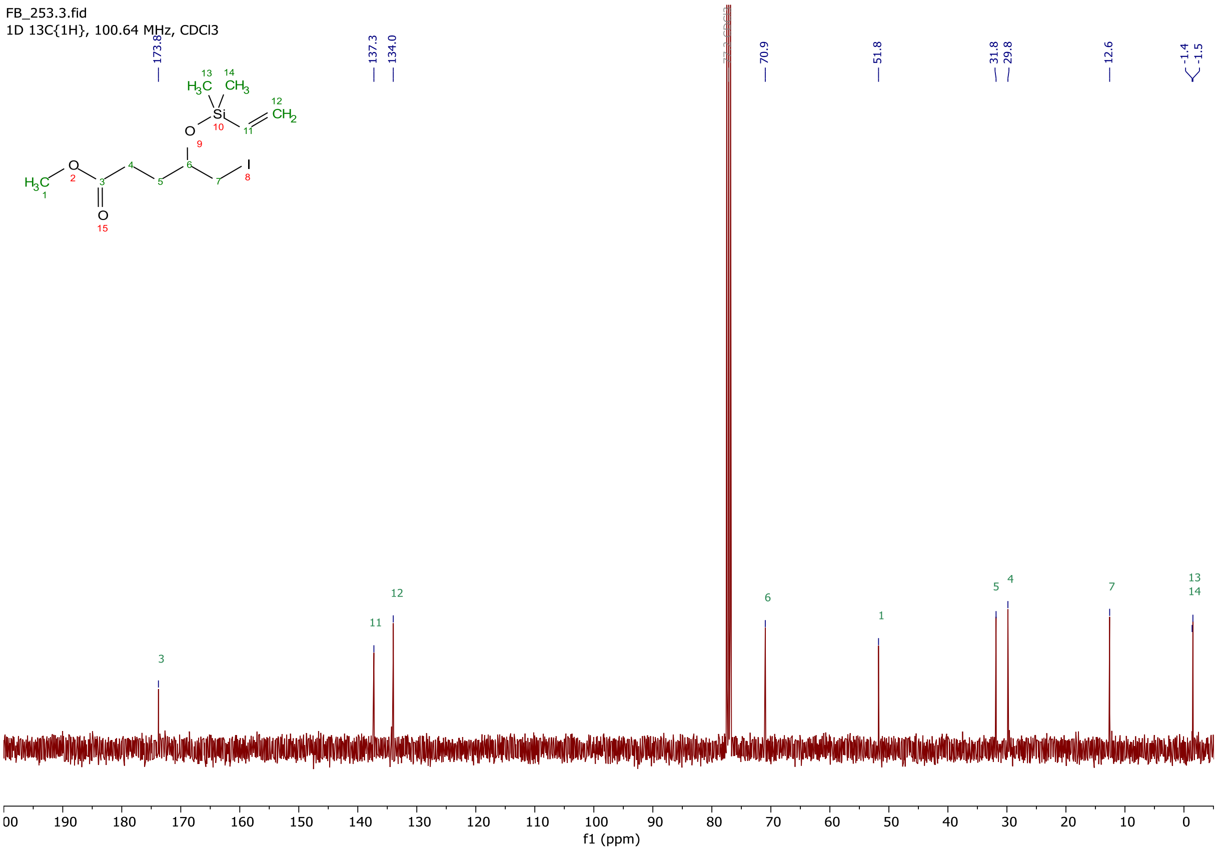

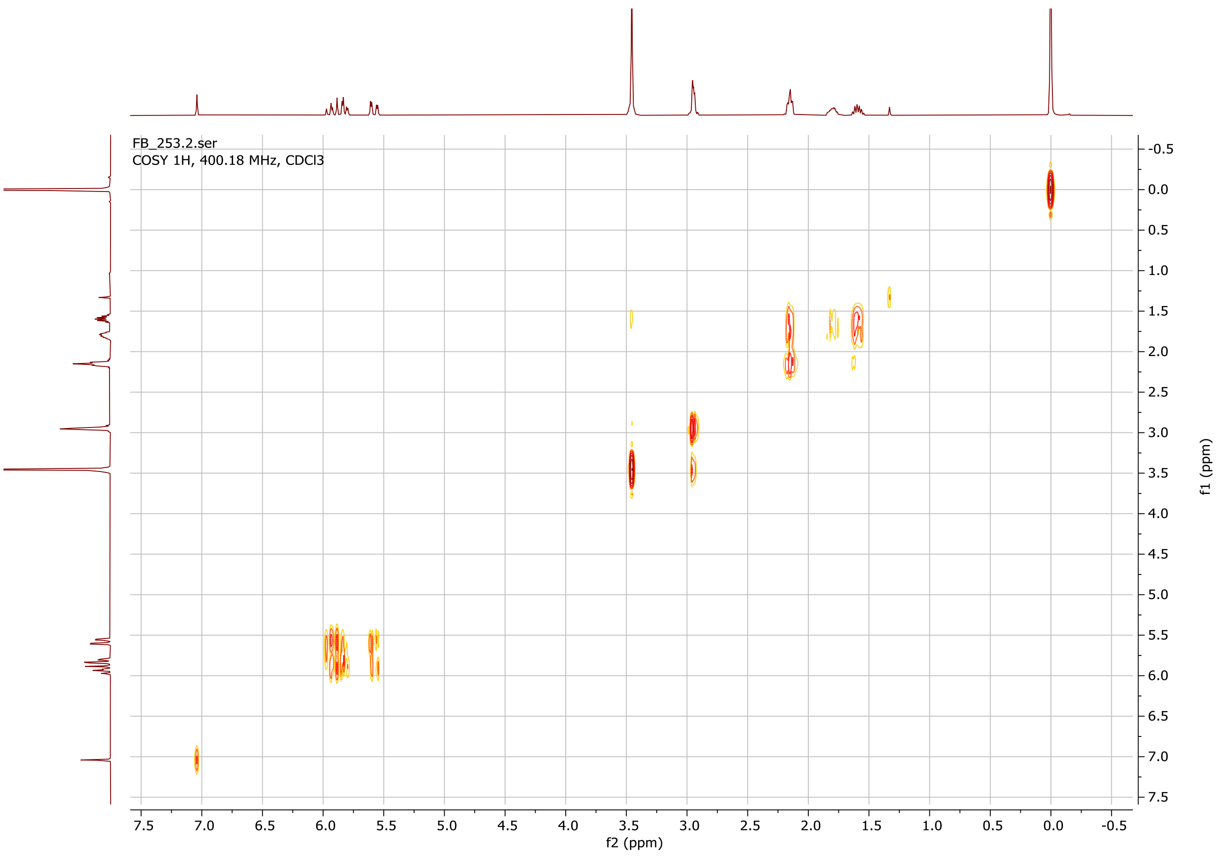
FB_253.2.ser
COSY 1H, 400.18 MHz, CDCl3
f2 (ppm)
f1 (ppm)

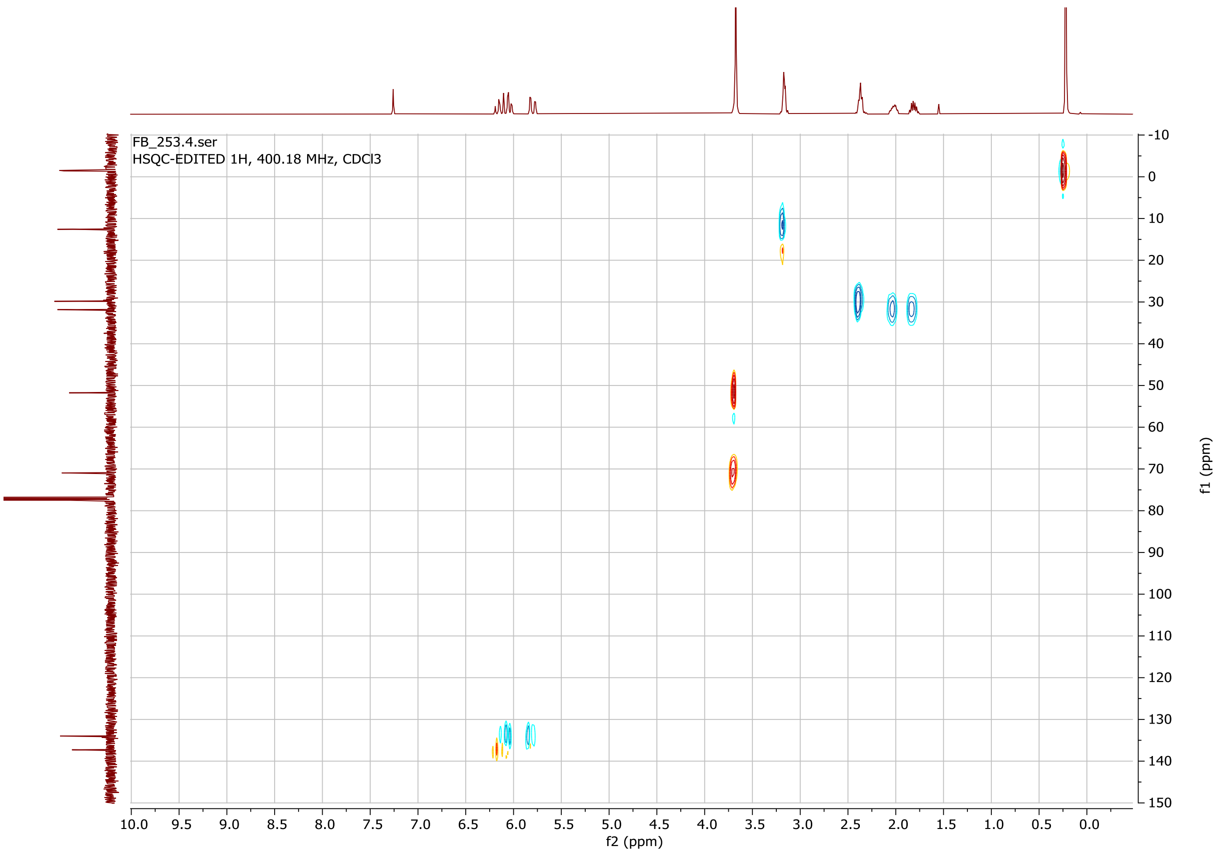
FB_253.4.ser
HSQC-EDITED 1H, 400.18 MHz, CDCl3
f2 (ppm)
f1 (ppm)

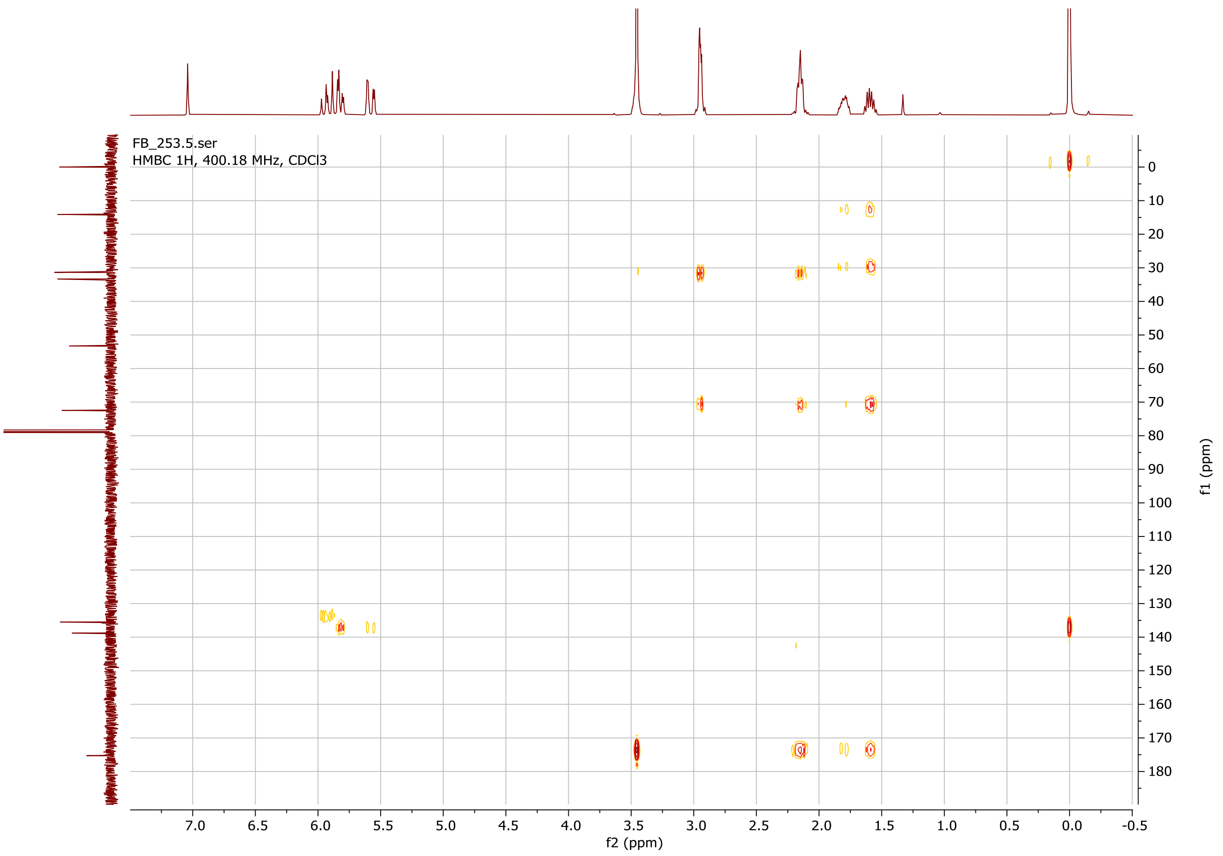

(2-Iodo-1-phenylethoxy)dimethylvinylsilane **4n**

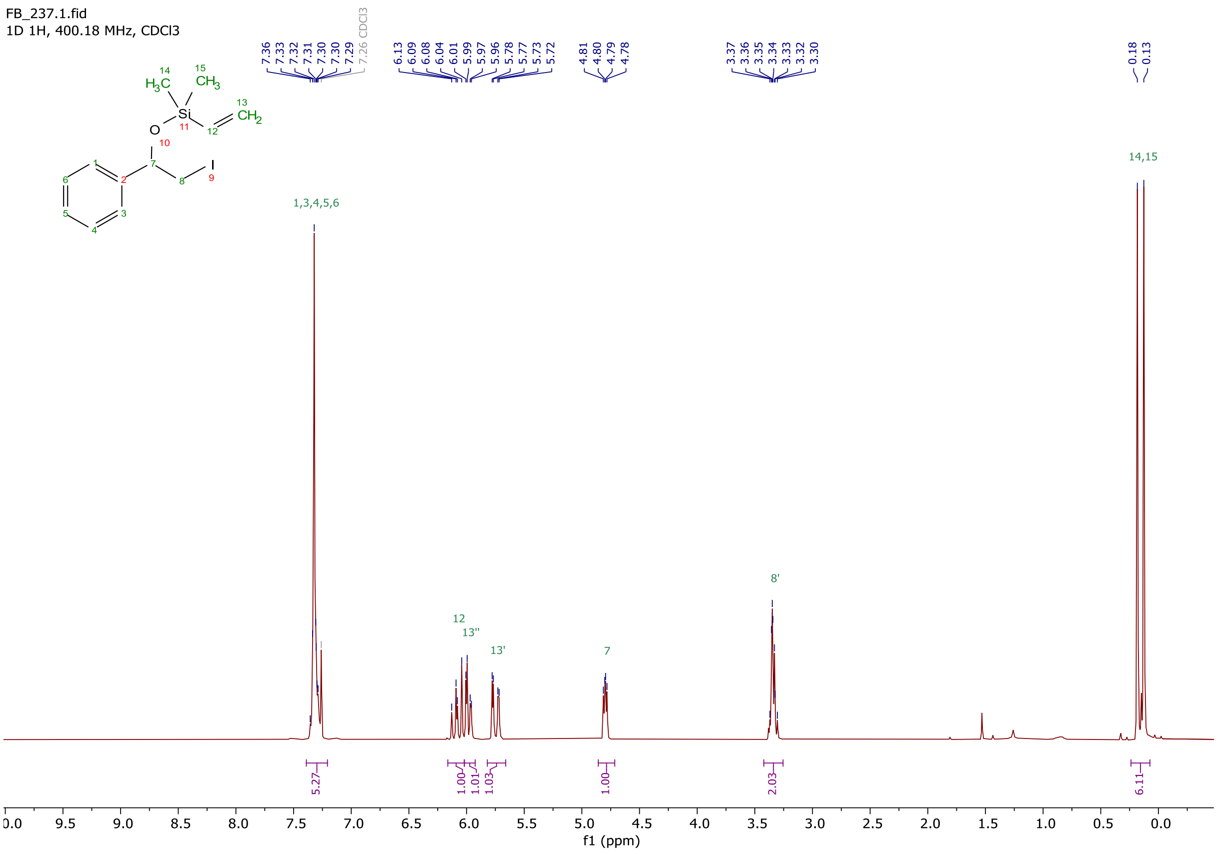

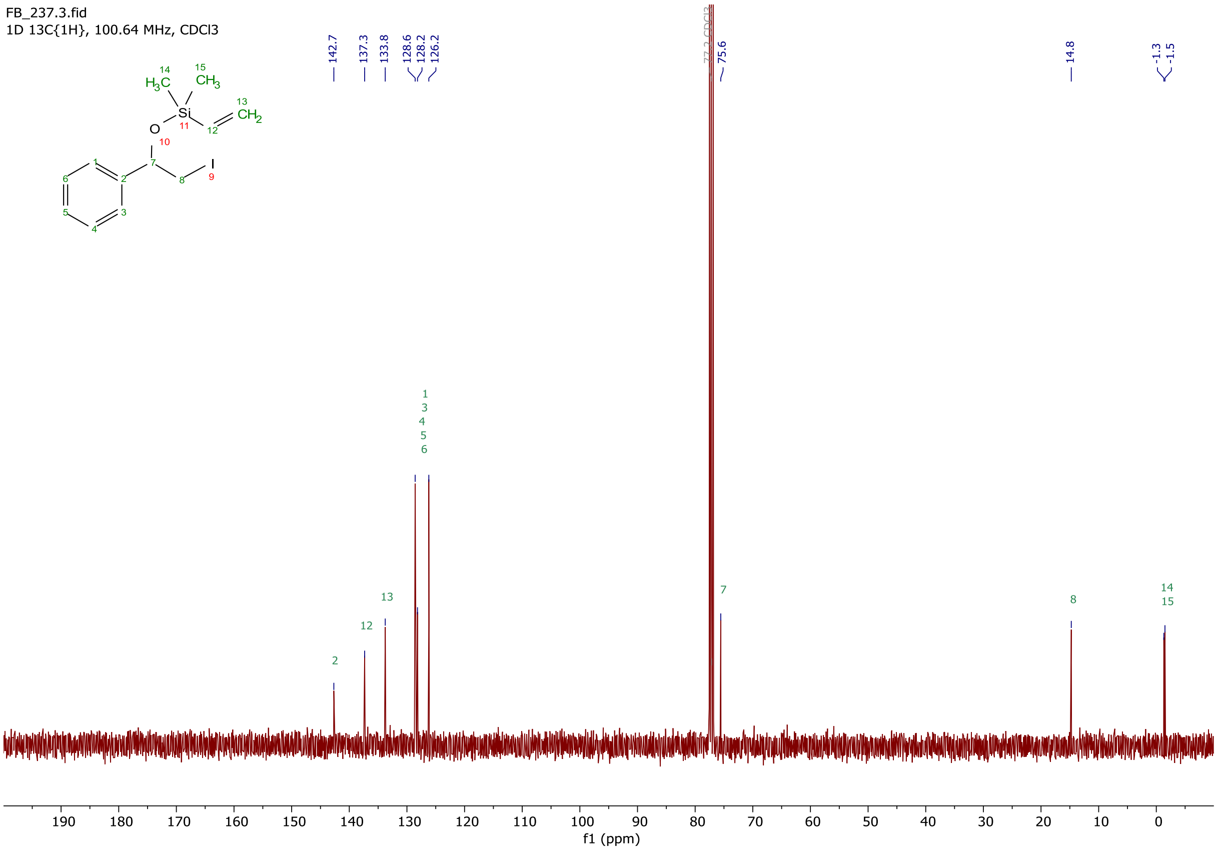
FB_237.3.fid
1D 13C{1H}, 100.64 MHz, CDCl3
142.7
137.3
133.8
128.6
128.2
126.2
77.2 CDCl3
75.6
14.8
-1.3
-1.5
f1 (ppm)

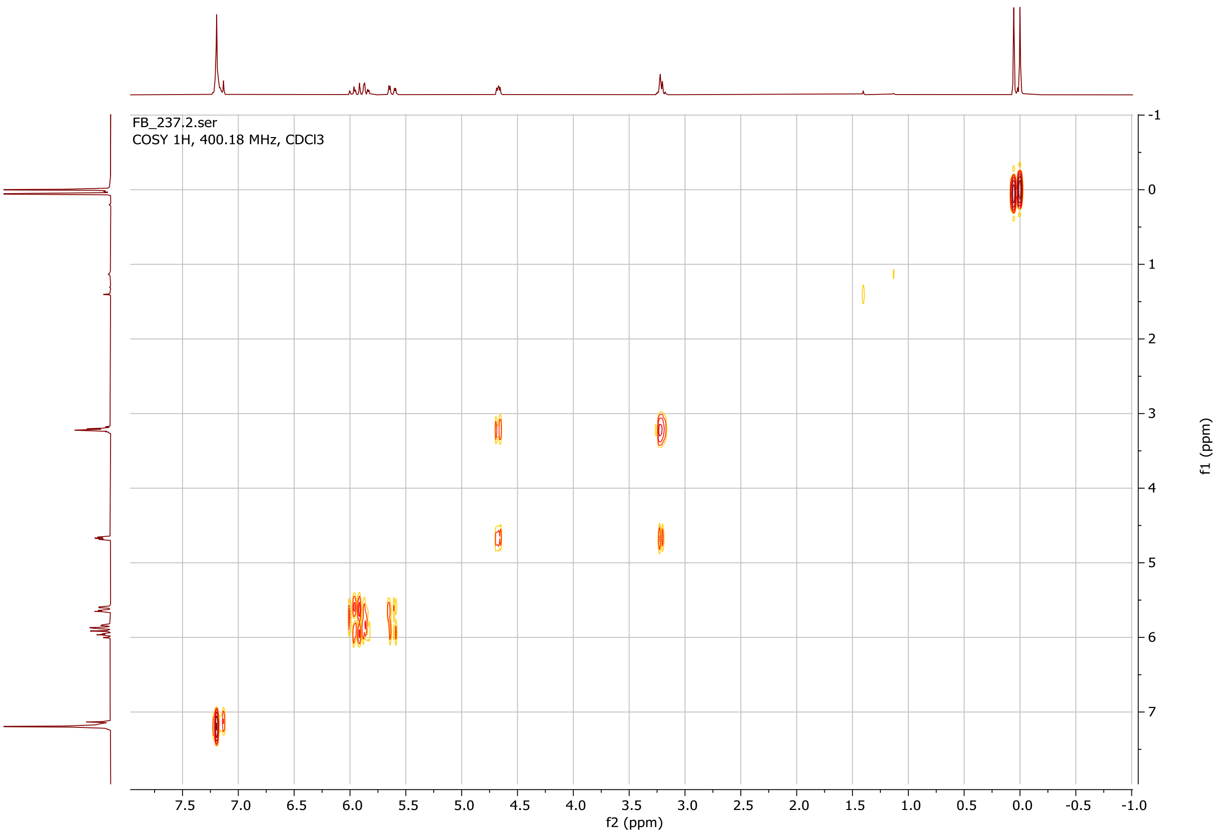
FB_237.2.ser
COSY 1H, 400.18 MHz, CDCl3
f2 (ppm)
f1 (ppm)

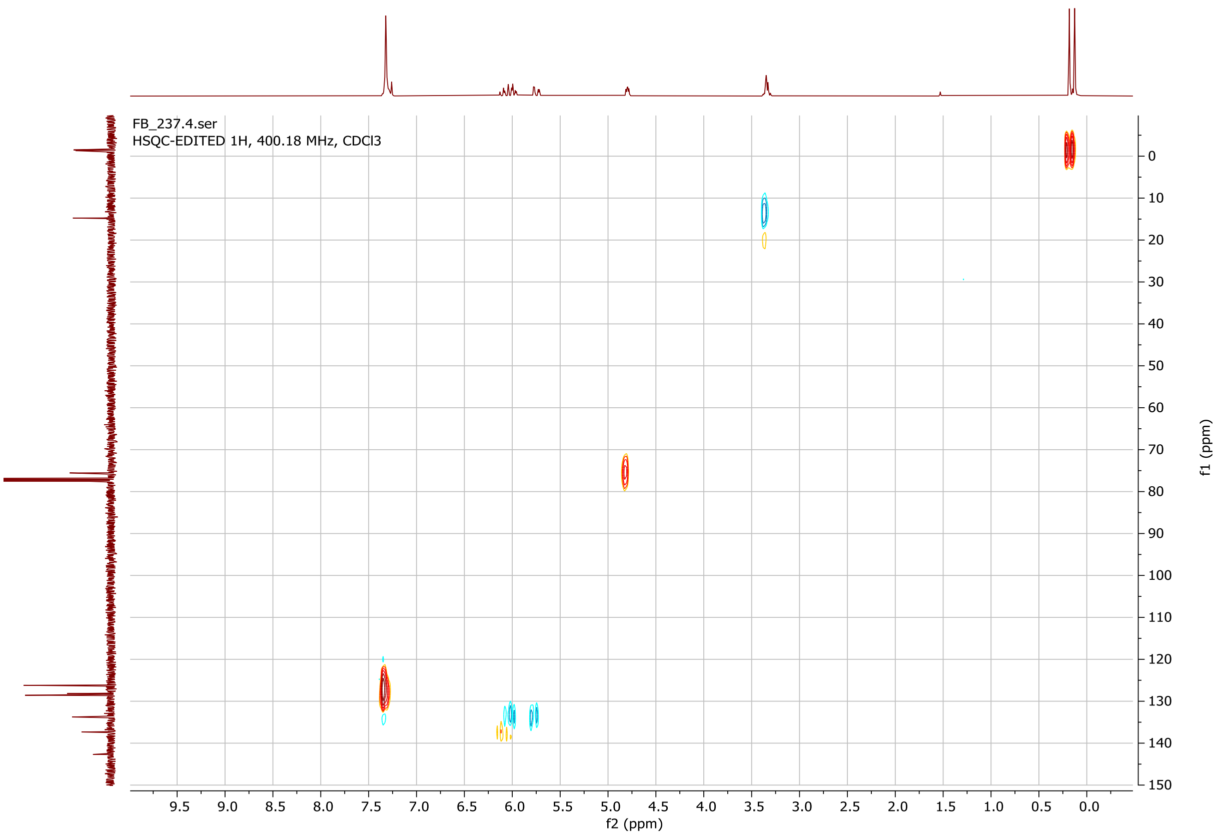
FB_237.4.ser
HSQC-EDITED 1H, 400.18 MHz, CDCl3
f1 (ppm)
f2 (ppm)

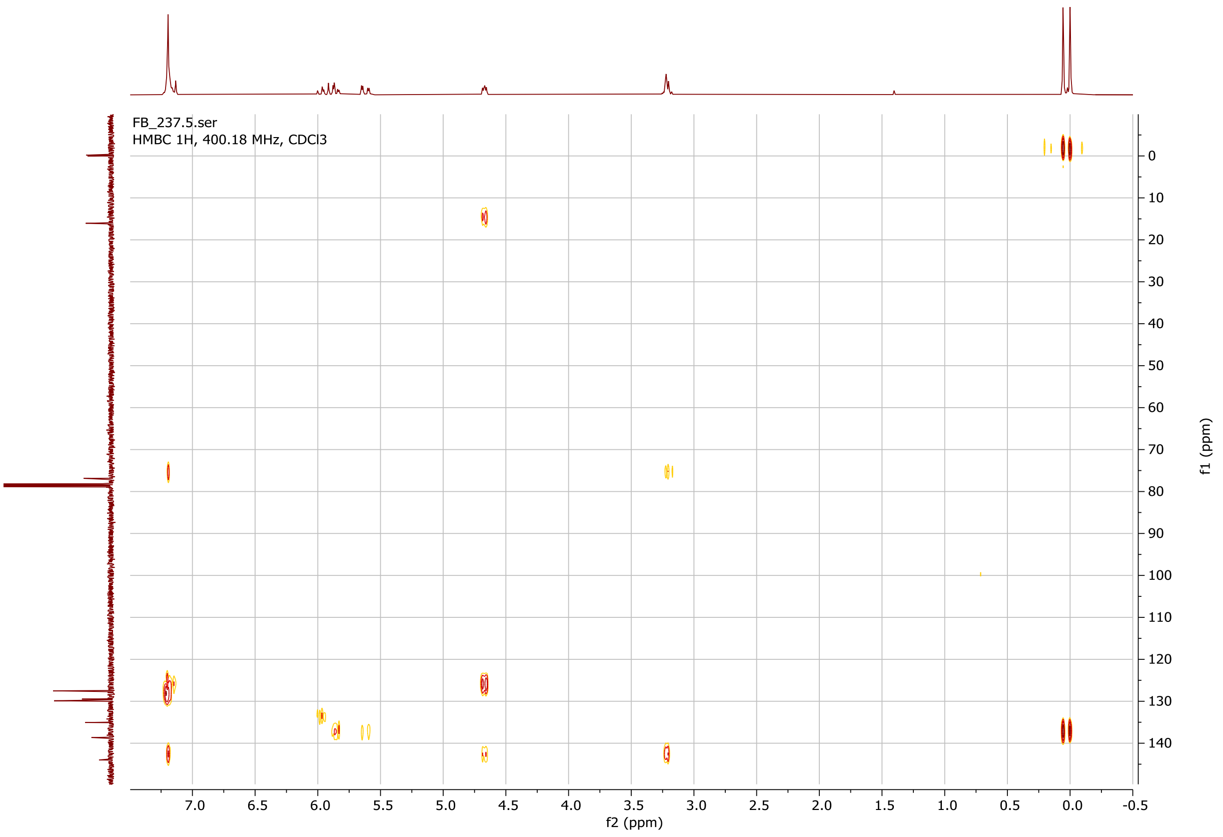
FB_237.5.ser
HMBC 1H, 400.18 MHz, CDCl3
f1 (ppm)
f2 (ppm)

(2-Iodo-1-(4-methoxyphenyl)ethoxy)dimethylvinylsilane **4o**

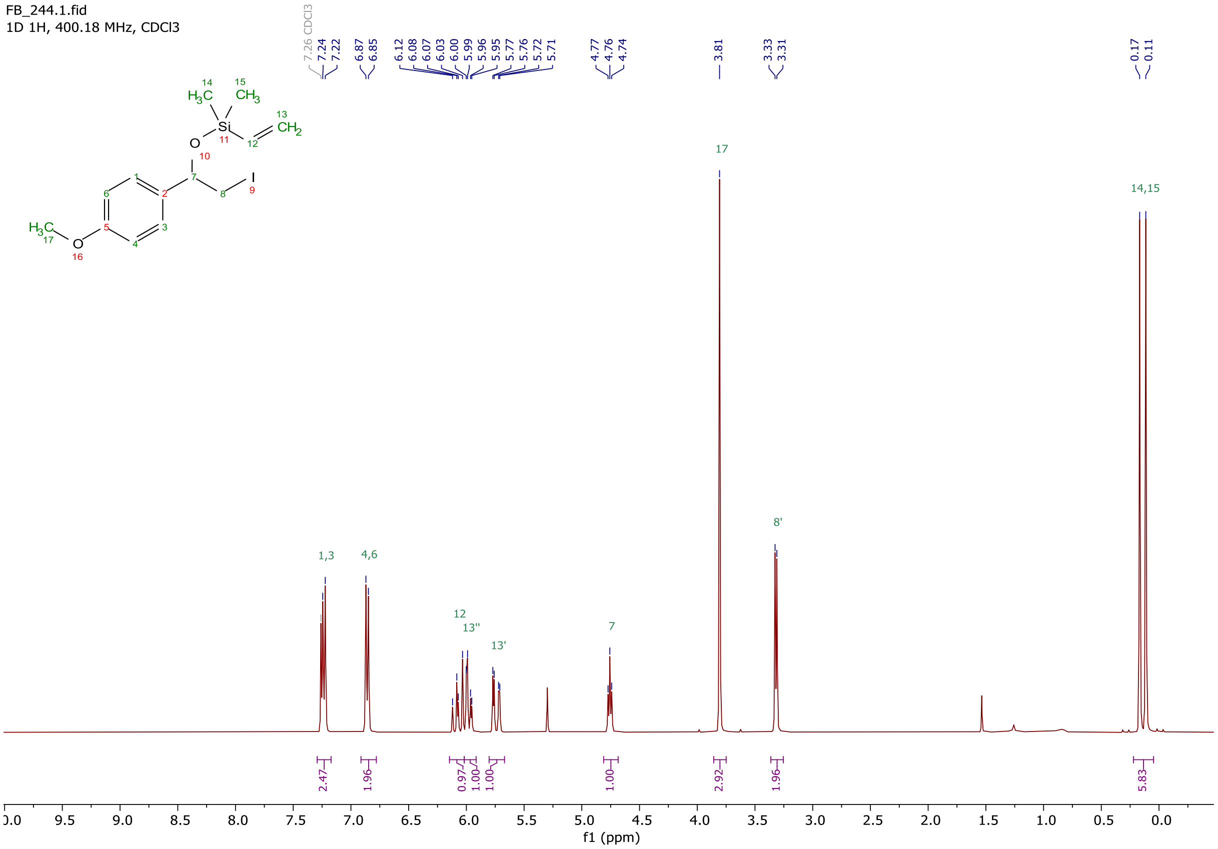

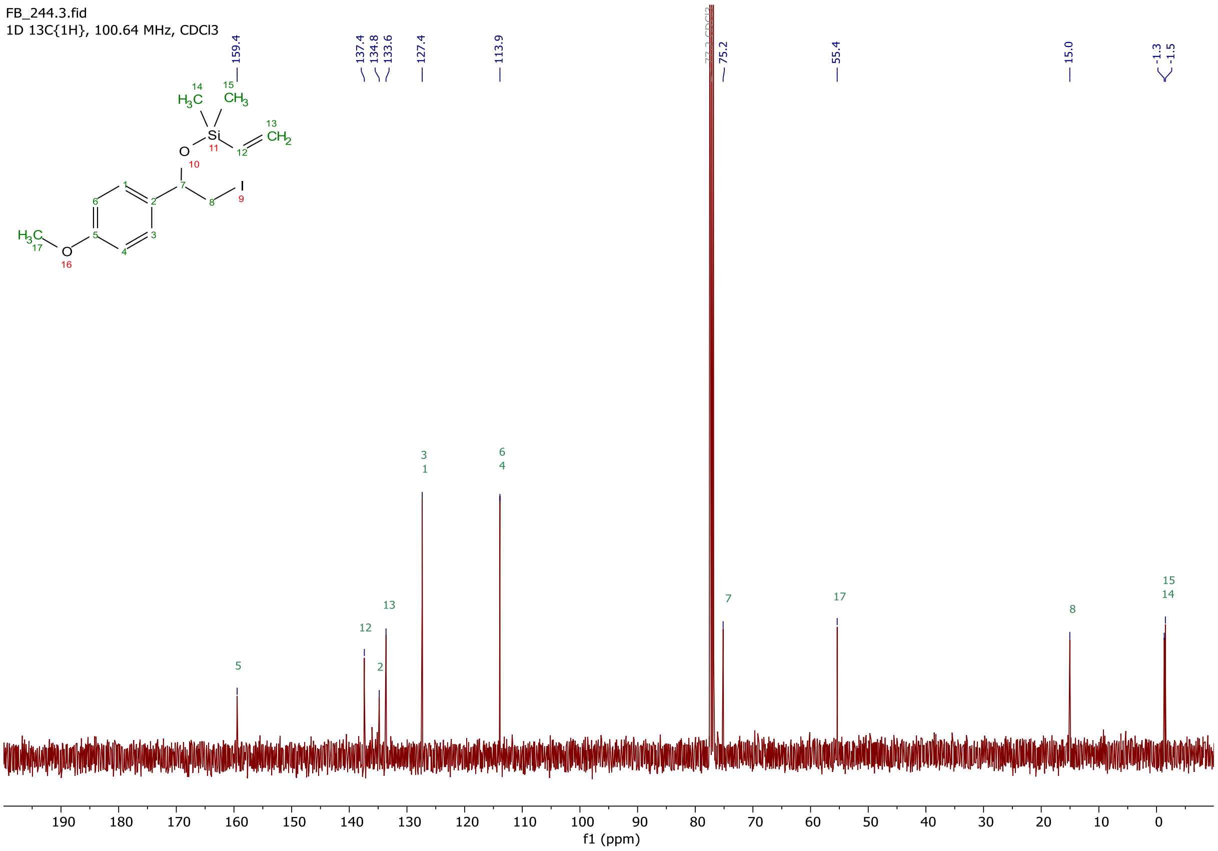

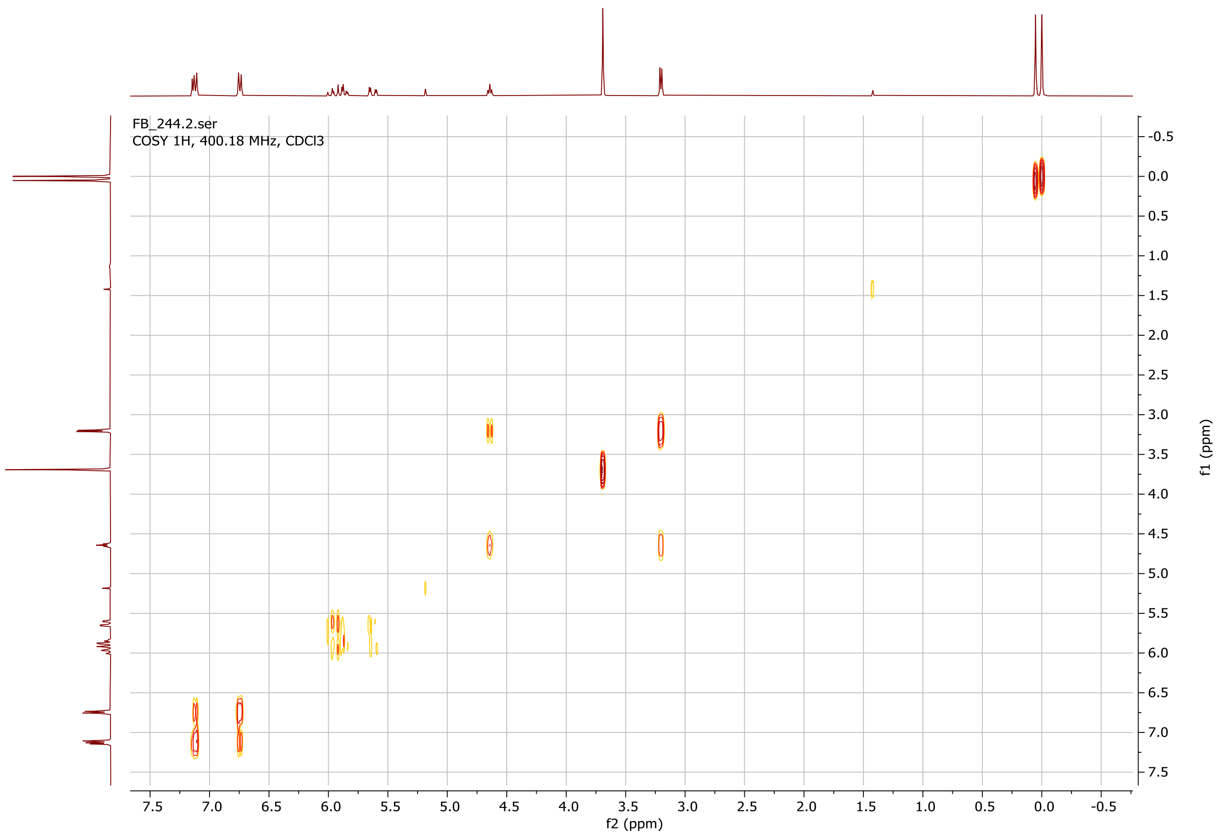
FB_244.2.ser
COSY 1H, 400.18 MHz, CDCl3
f2 (ppm)
f1 (ppm)

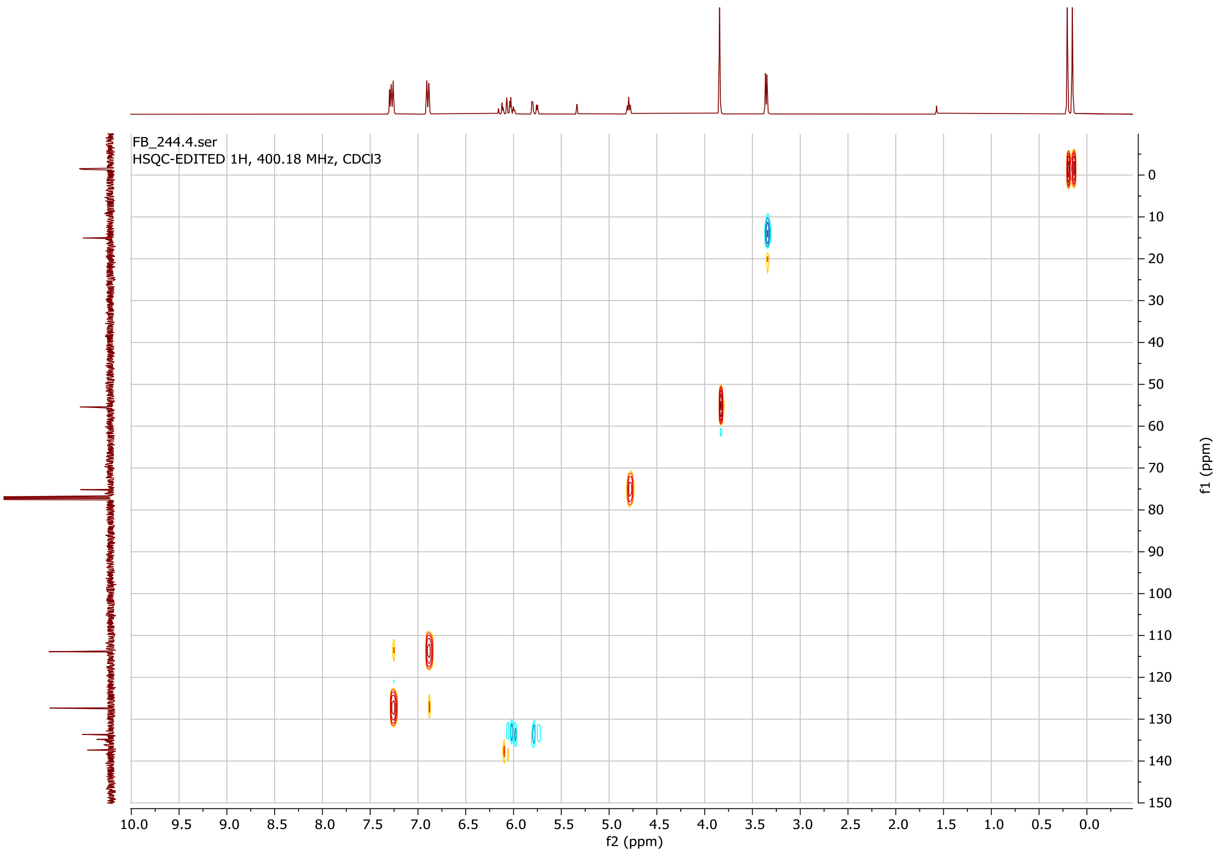
FB_244.4.ser
HSQC-EDITED 1H, 400.18 MHz, CDCl3
f2 (ppm)
f1 (ppm)

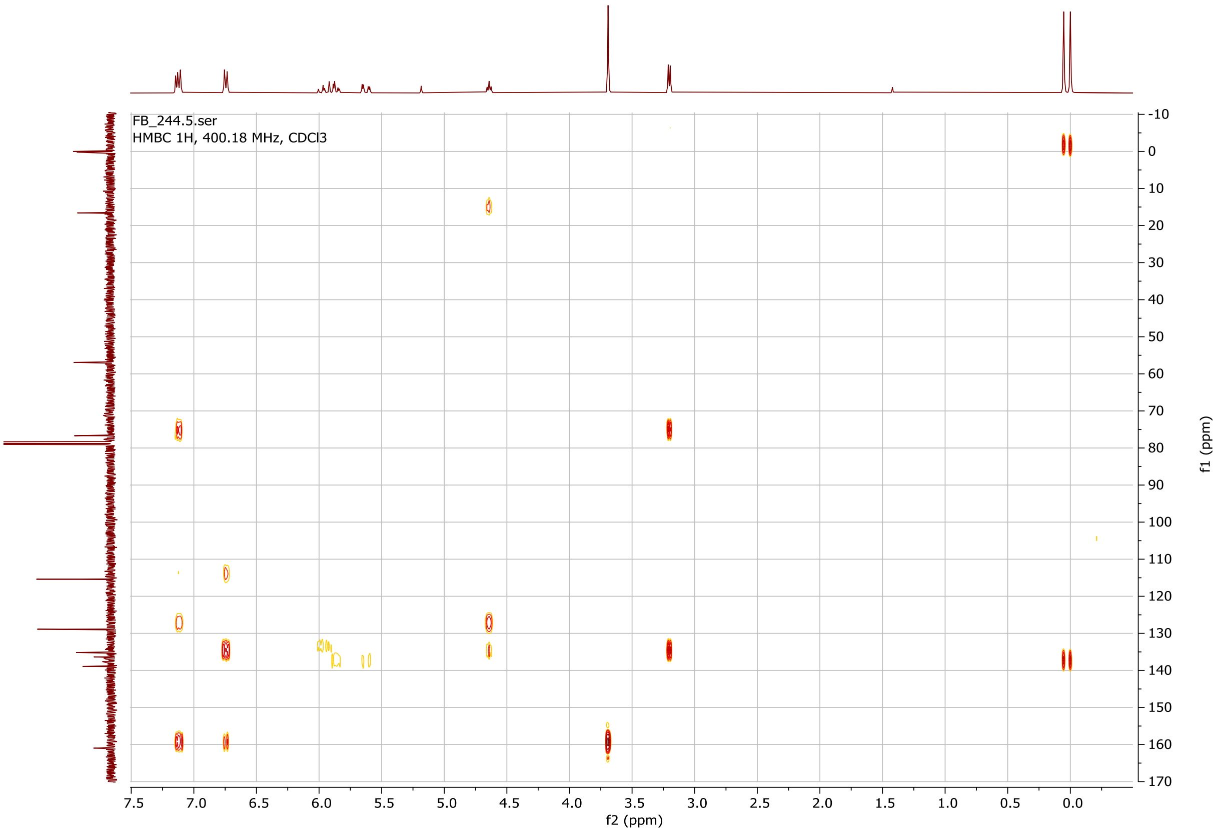

(2-Iodo-1-(4-(trifluoromethyl)phenyl)ethoxy)dimethylvinylsilane **4p**

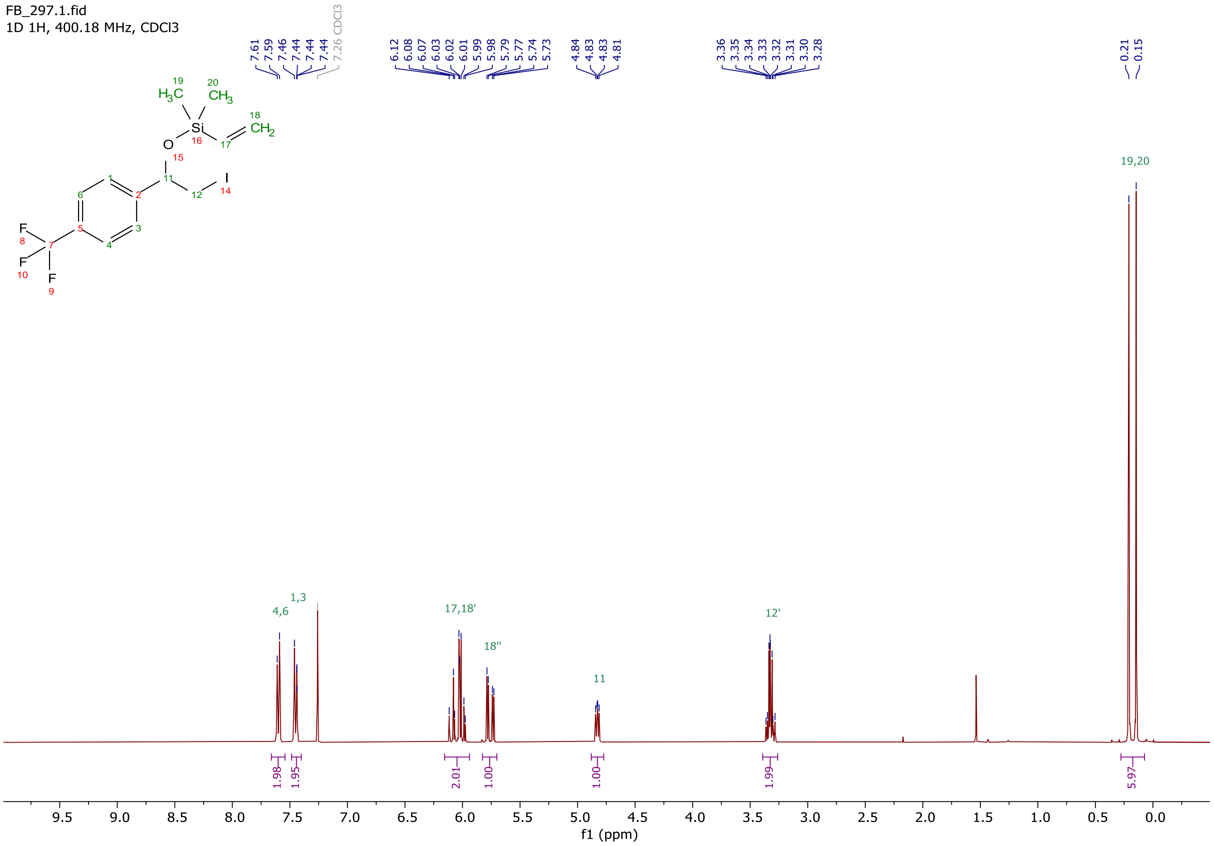

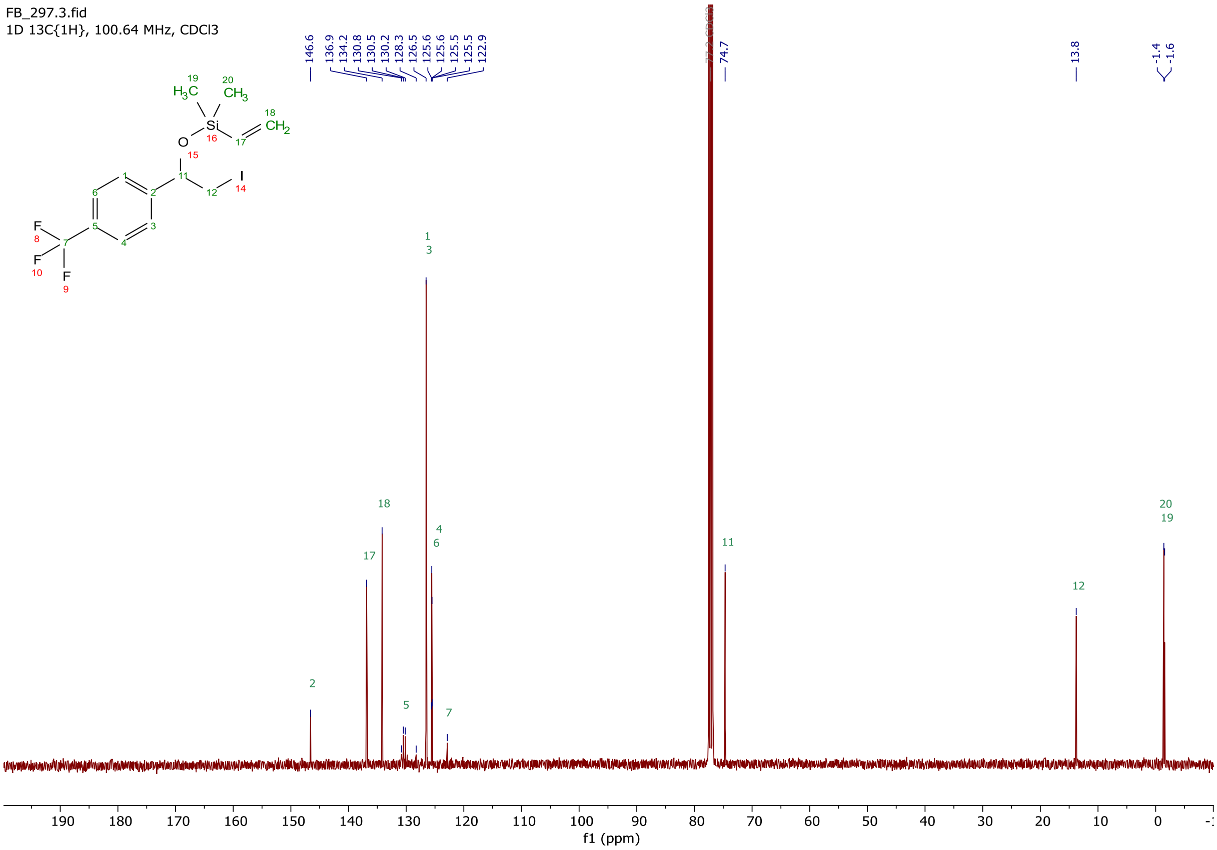

FB_297.3.fid
1D 13C{1H}, 100.64 MHz, CDCl3
146.6
136.9
134.2
130.8
130.5
130.2
128.3
126.5
125.6
125.6
125.5
125.5
122.9
74.7
13.8
-1.4
-1.6
f1 (ppm)

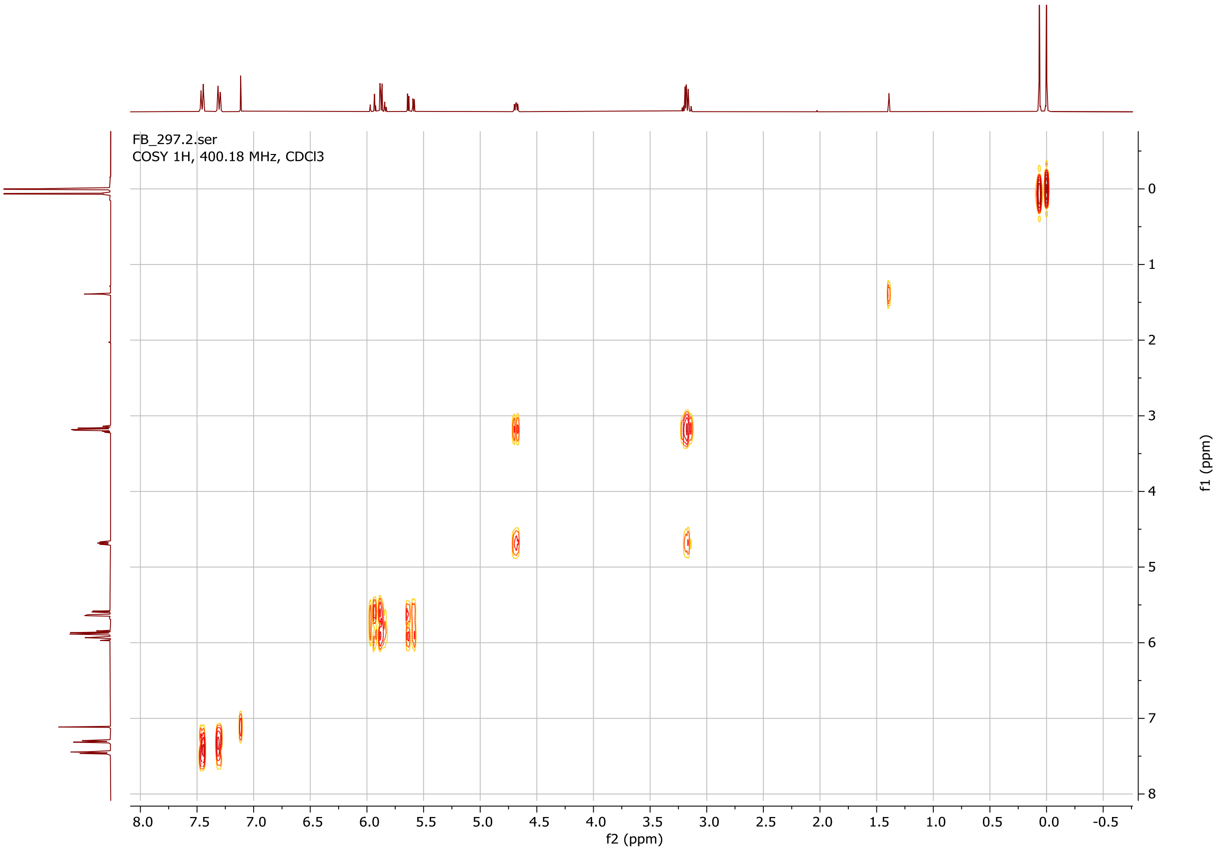

FB_297.2.ser
COSY 1H, 400.18 MHz, CDCl3
f2 (ppm)
f1 (ppm)

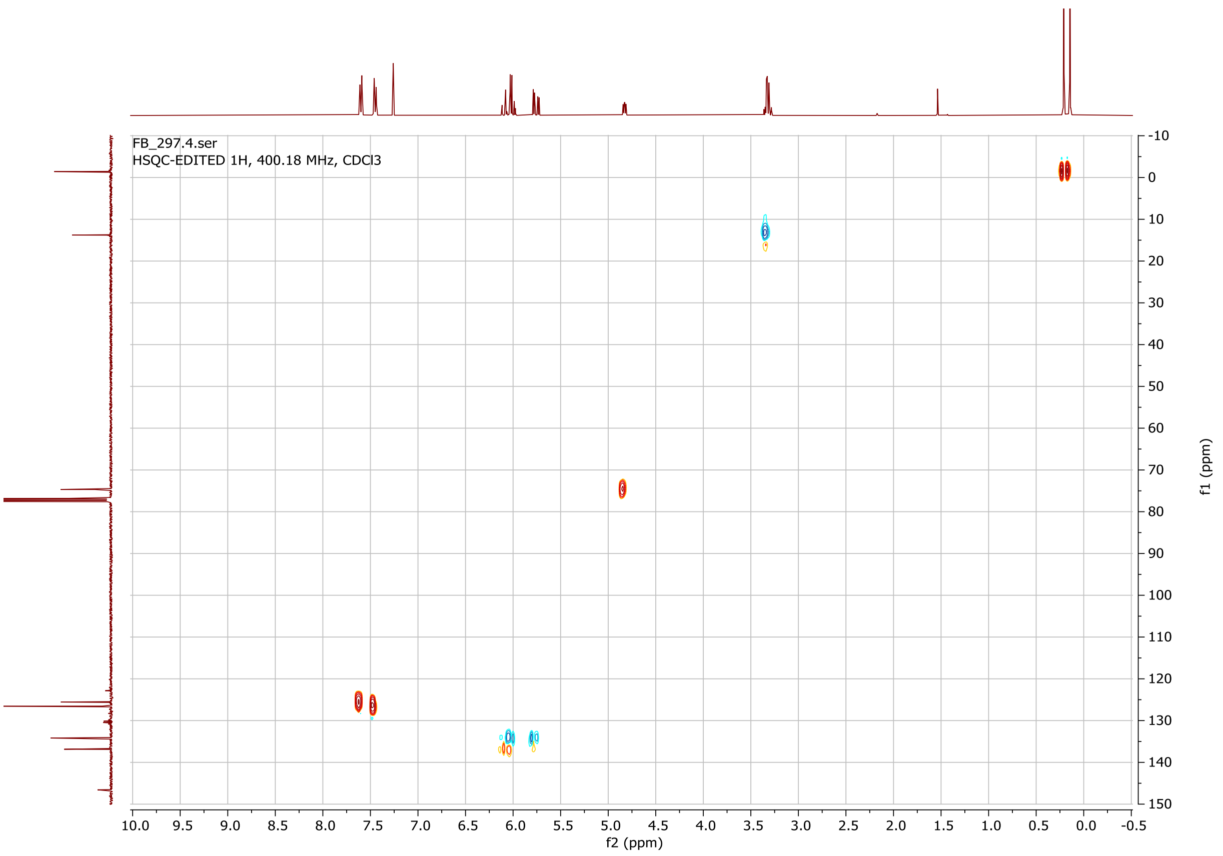
FB_297.4.ser
HSQC-EDITED 1H, 400.18 MHz, CDCl3
f2 (ppm)
f1 (ppm)

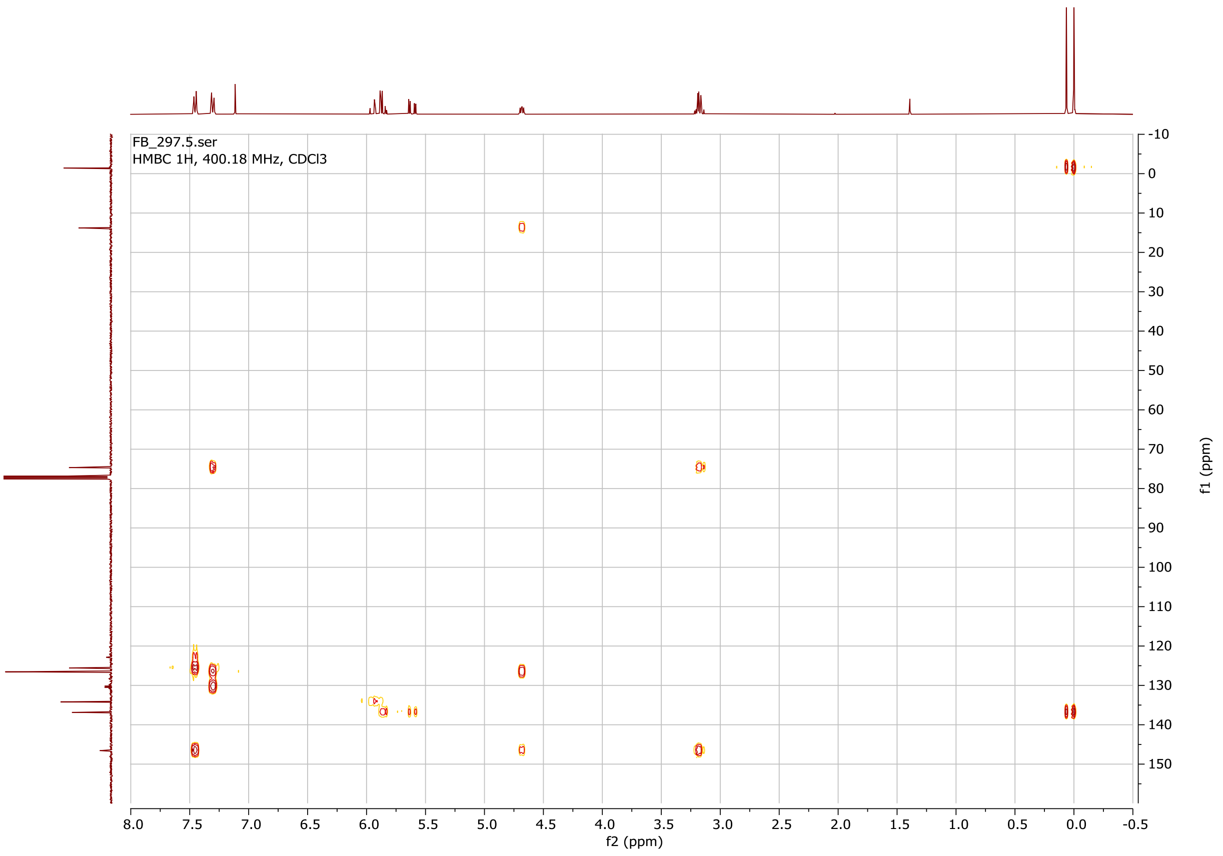
FB_297.5.ser
HMBC 1H, 400.18 MHz, CDCl3
f2 (ppm)
f1 (ppm)

((1-Iodo-1-phenylpropan-2-yl)oxy)dimethylvinylsilane **4q**

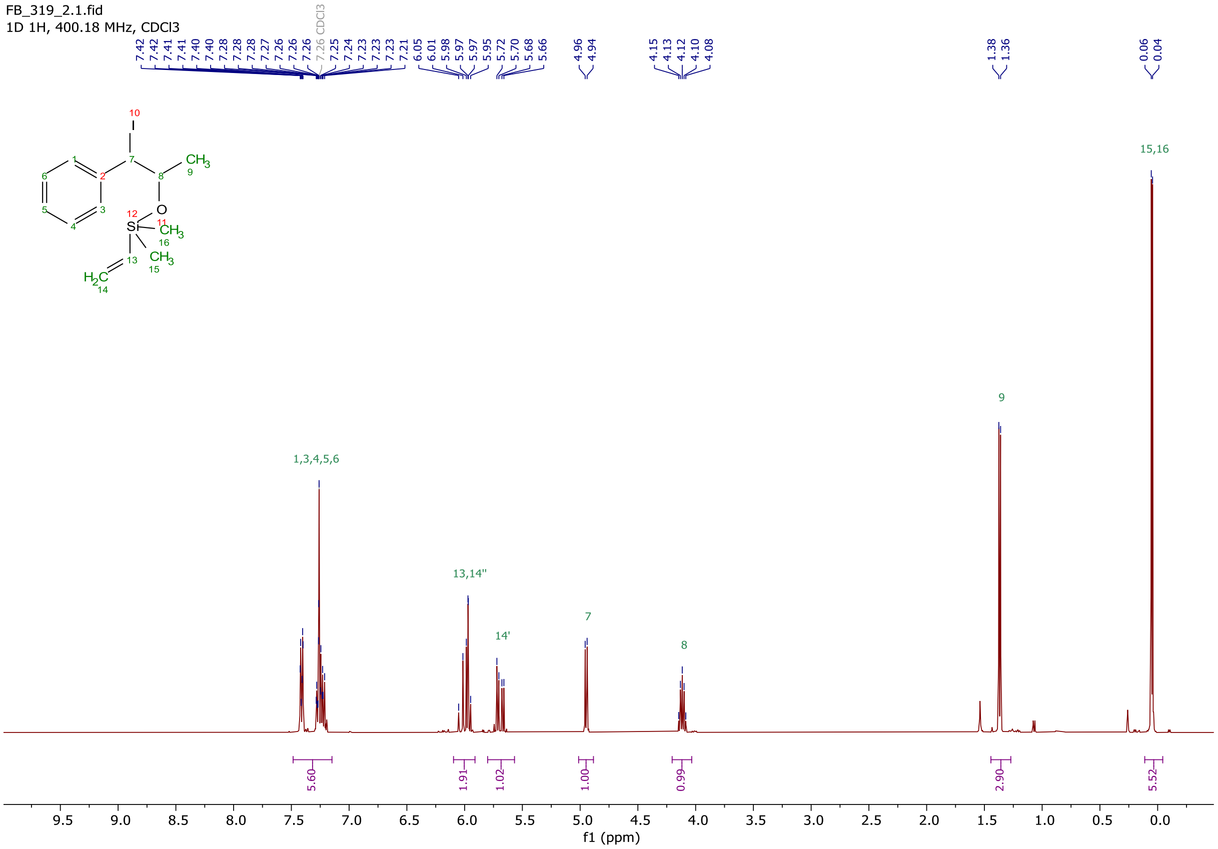

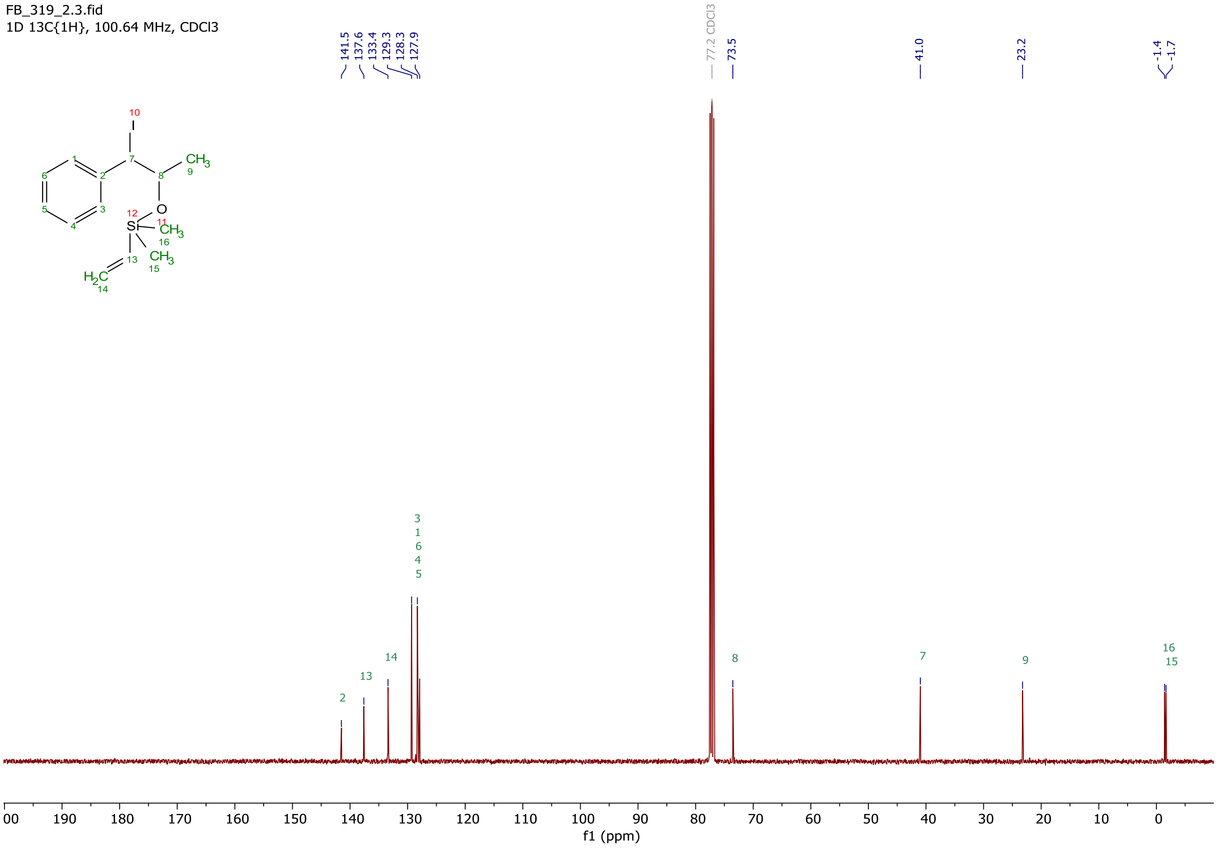

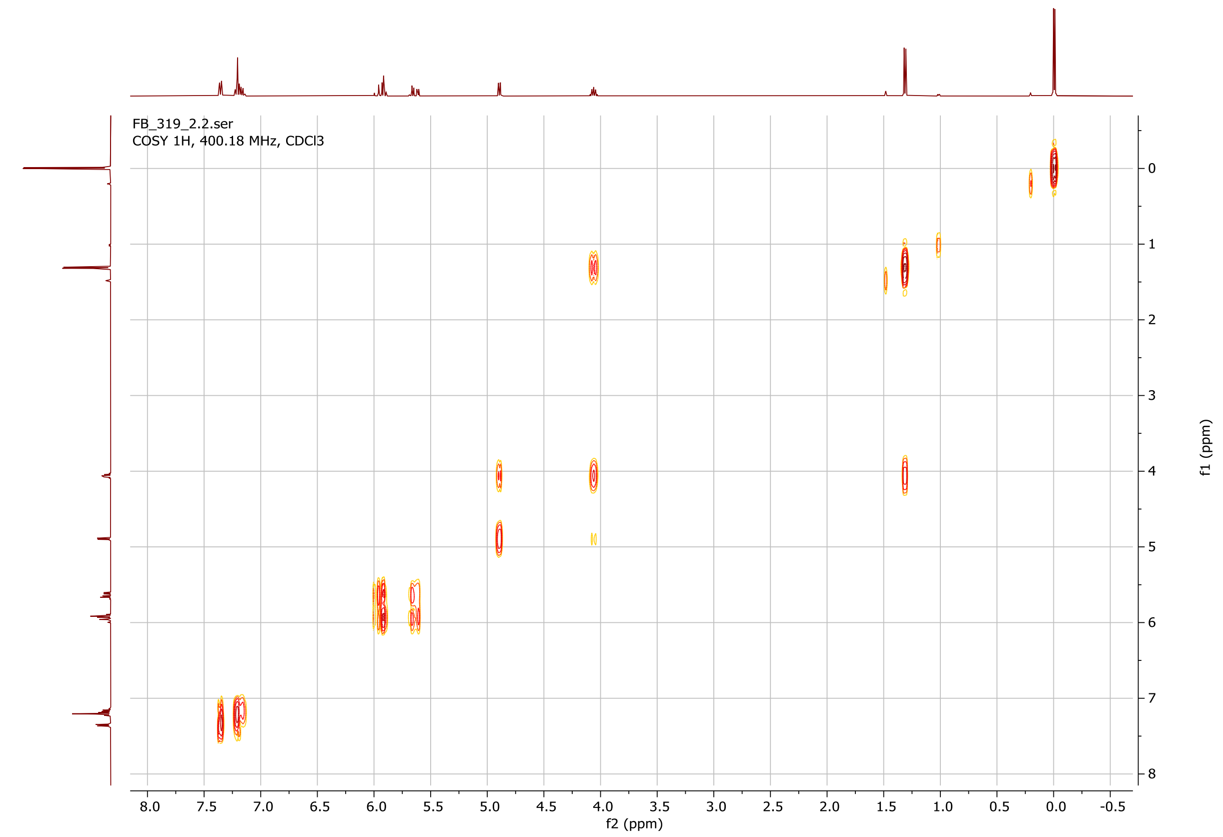
FB_319_2.2.ser
COSY 1H, 400.18 MHz, CDCl3
f2 (ppm)
f1 (ppm)

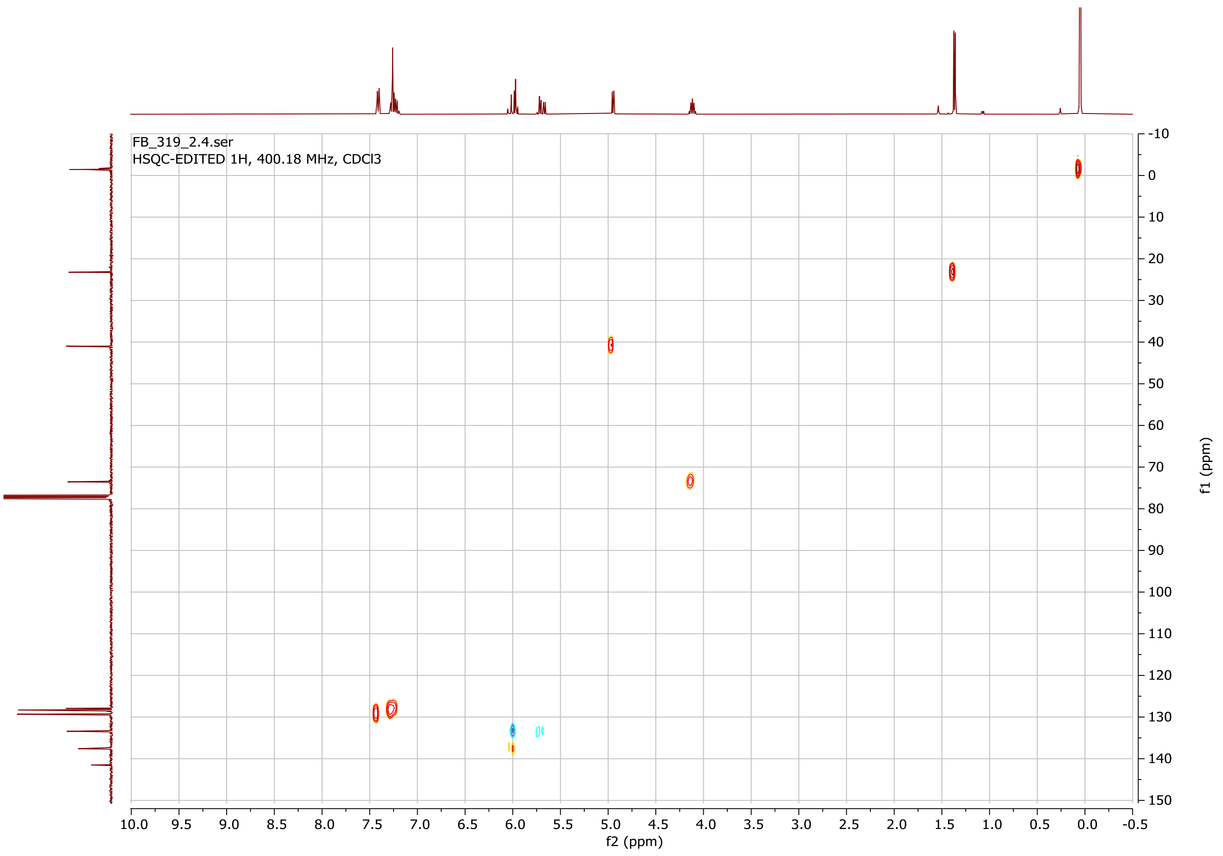
FB_319_2.4.ser
HSQC-EDITED 1H, 400.18 MHz, CDCl3
f2 (ppm)
f1 (ppm)

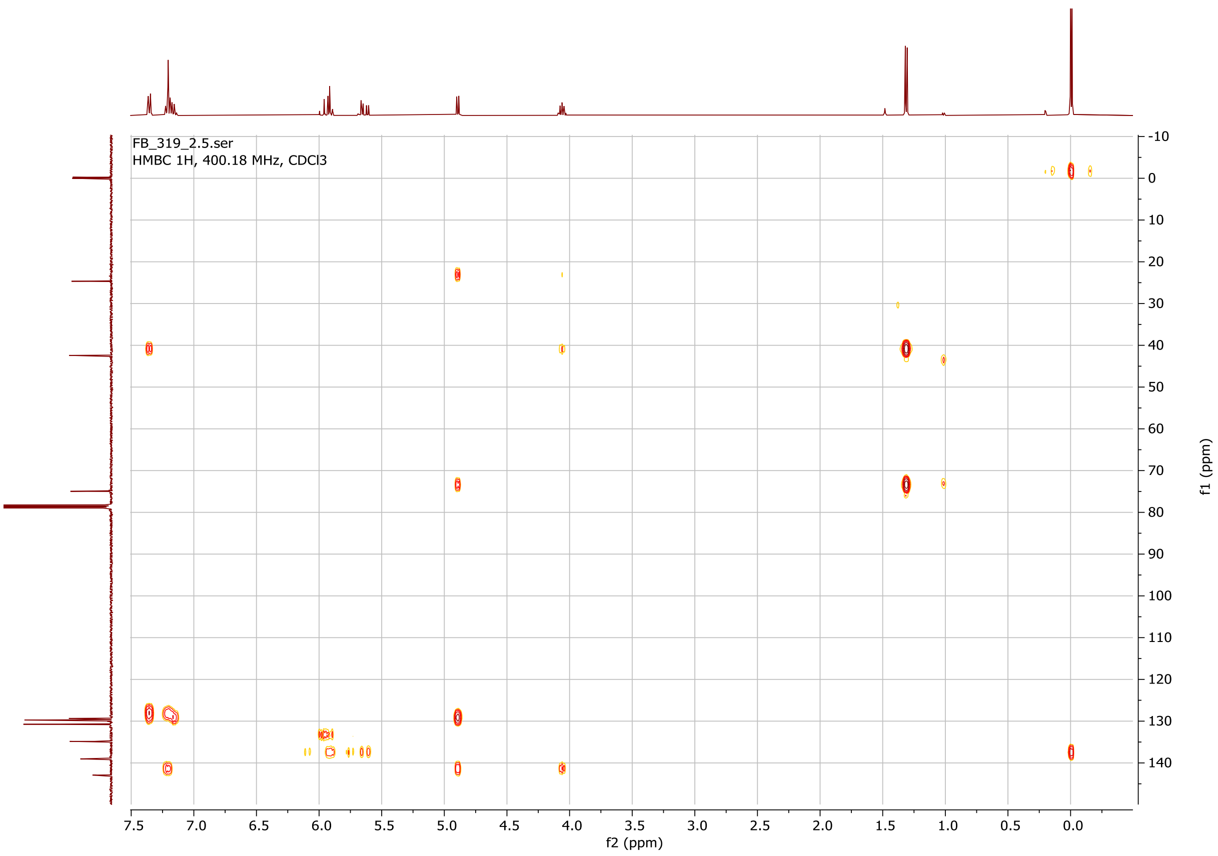

3-((Dimethylvinylsilyl)oxy)-4-iodo-4-phenylbutan-2-one **4r** *(inseparable 87:13 diastereomers mixture)*

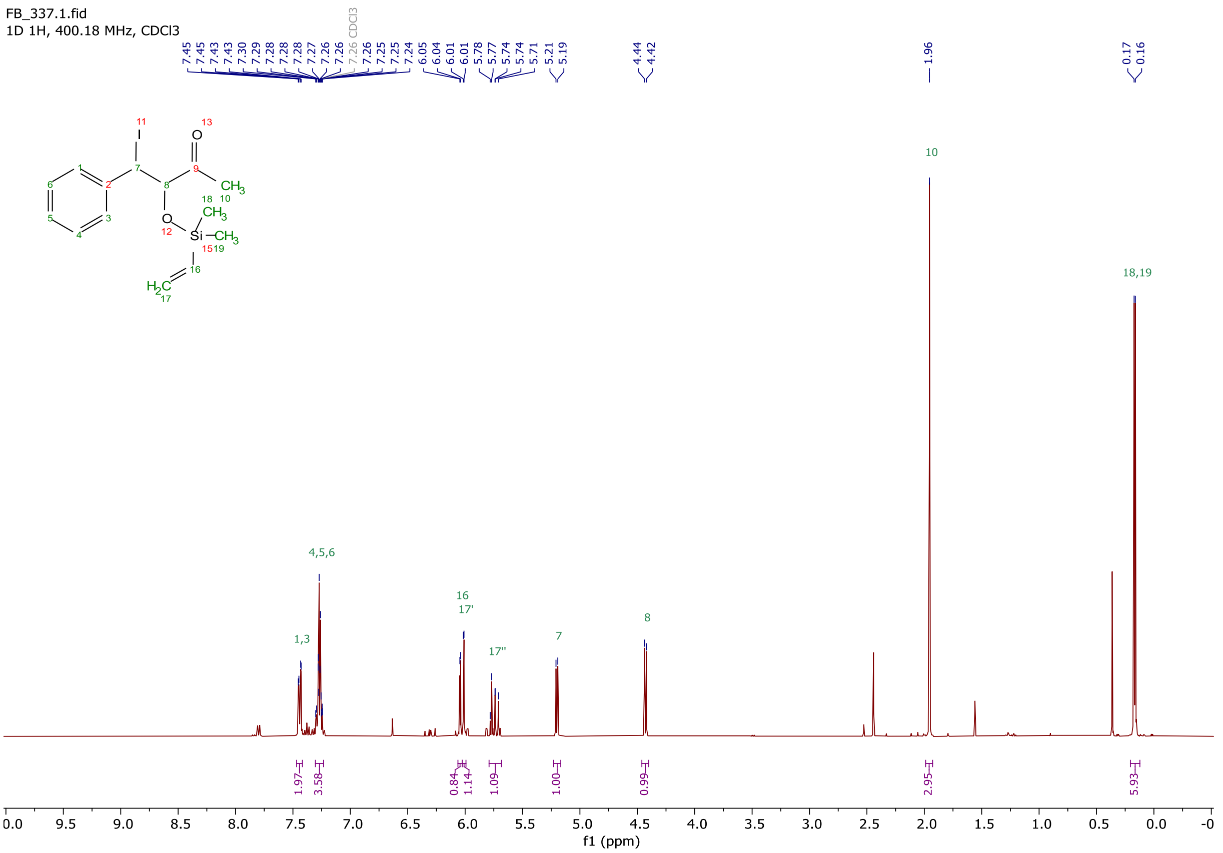

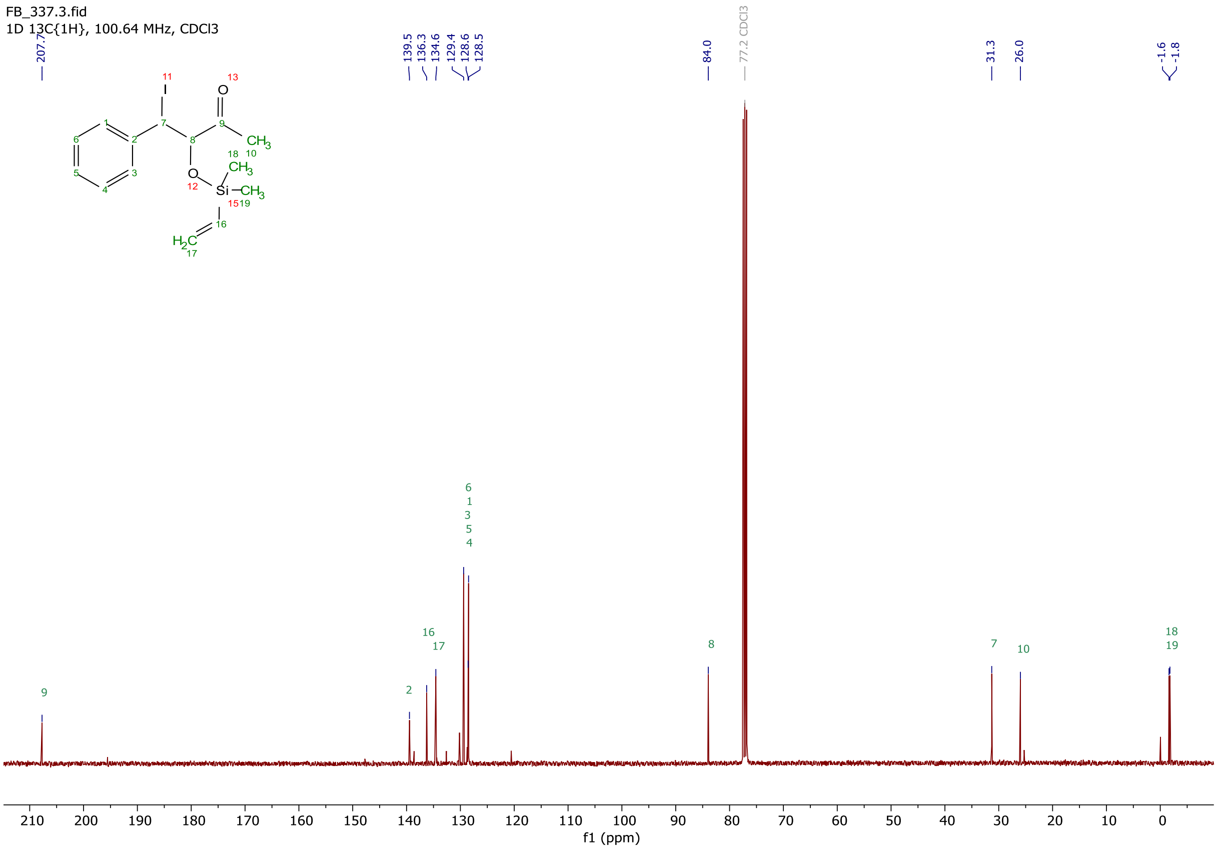
FB_337.3.fid
1D 13C{1H}, 100.64 MHz, CDCl3
207.7
139.5
136.3
134.6
129.4
128.6
128.5
84.0
77.2 CDCl3
31.3
26.0
-1.6
-1.8
f1 (ppm)

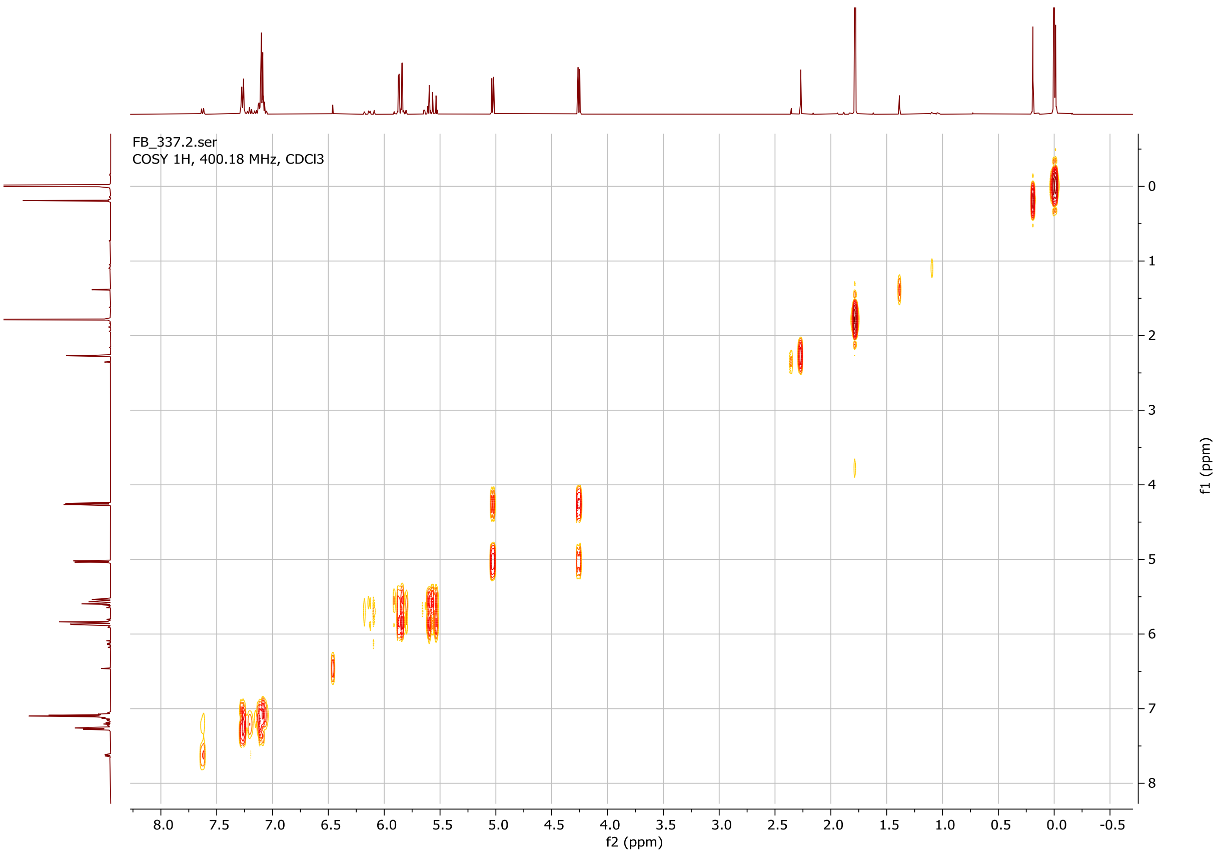
FB_337.2.ser
COSY 1H, 400.18 MHz, CDCl3
f2 (ppm)
f1 (ppm)

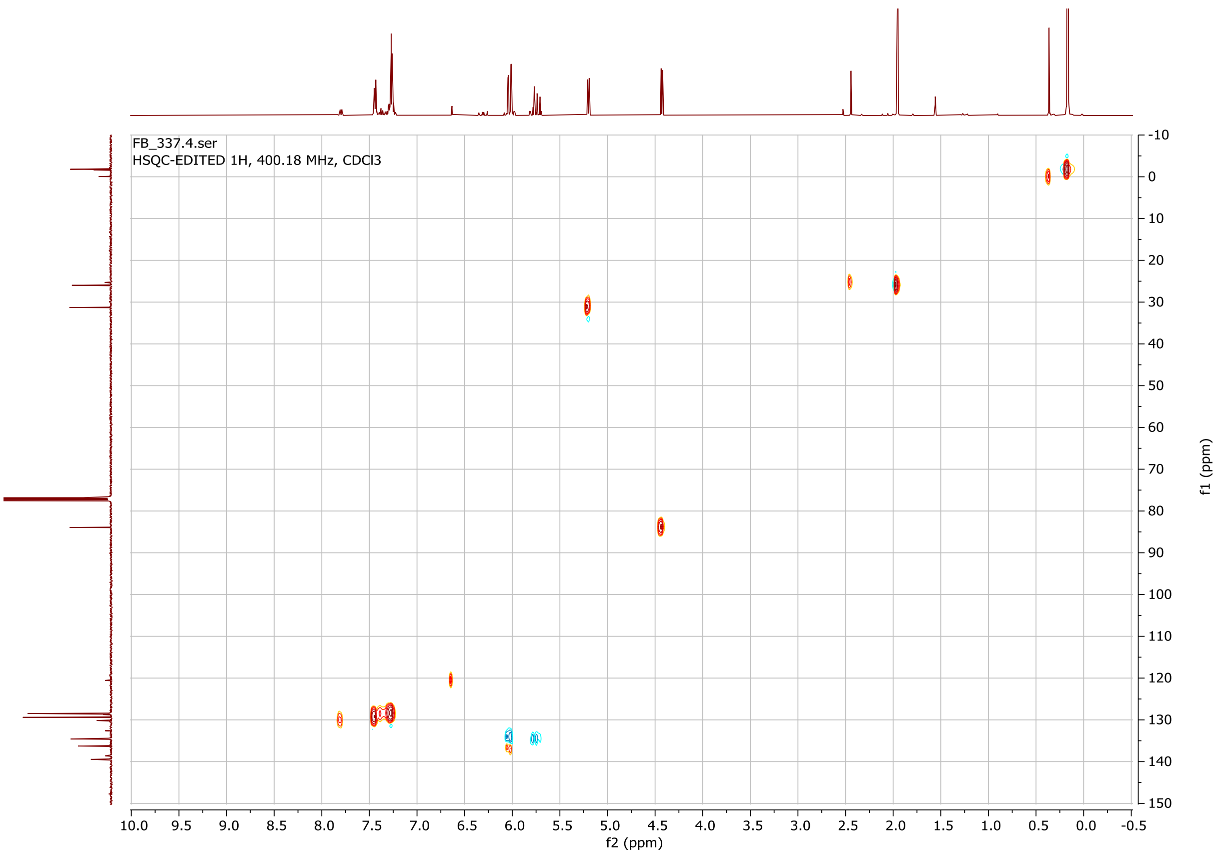
FB_337.4.ser
HSQC-EDITED 1H, 400.18 MHz, CDCl3
f2 (ppm)
f1 (ppm)

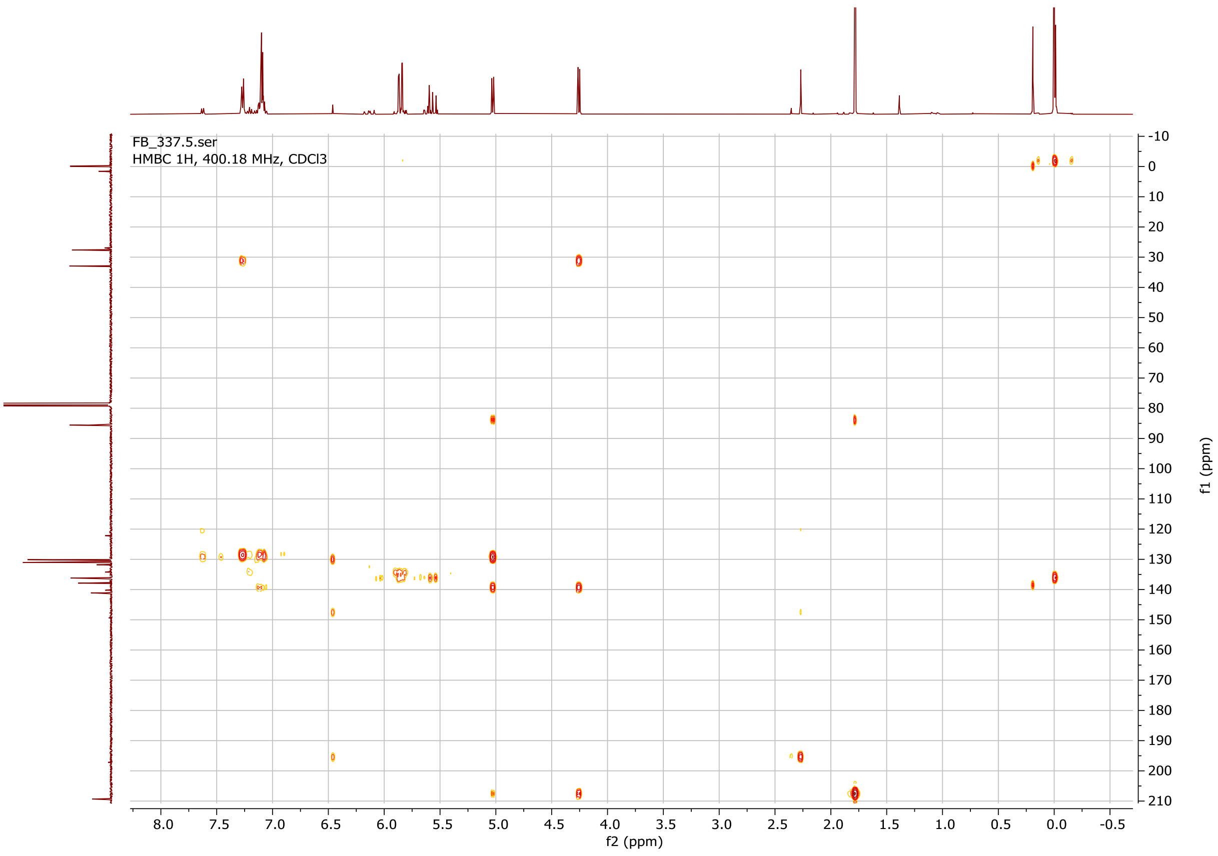
FB_337.5.ser
HMBC 1H, 400.18 MHz, CDCl3
f2 (ppm)
f1 (ppm)

((1-Iodo-3-isopropoxy-1-phenylpropan-2-yl)oxy)dimethylvinylsilane **4s** *(major of separable 80:20 diastereomers mixture)*

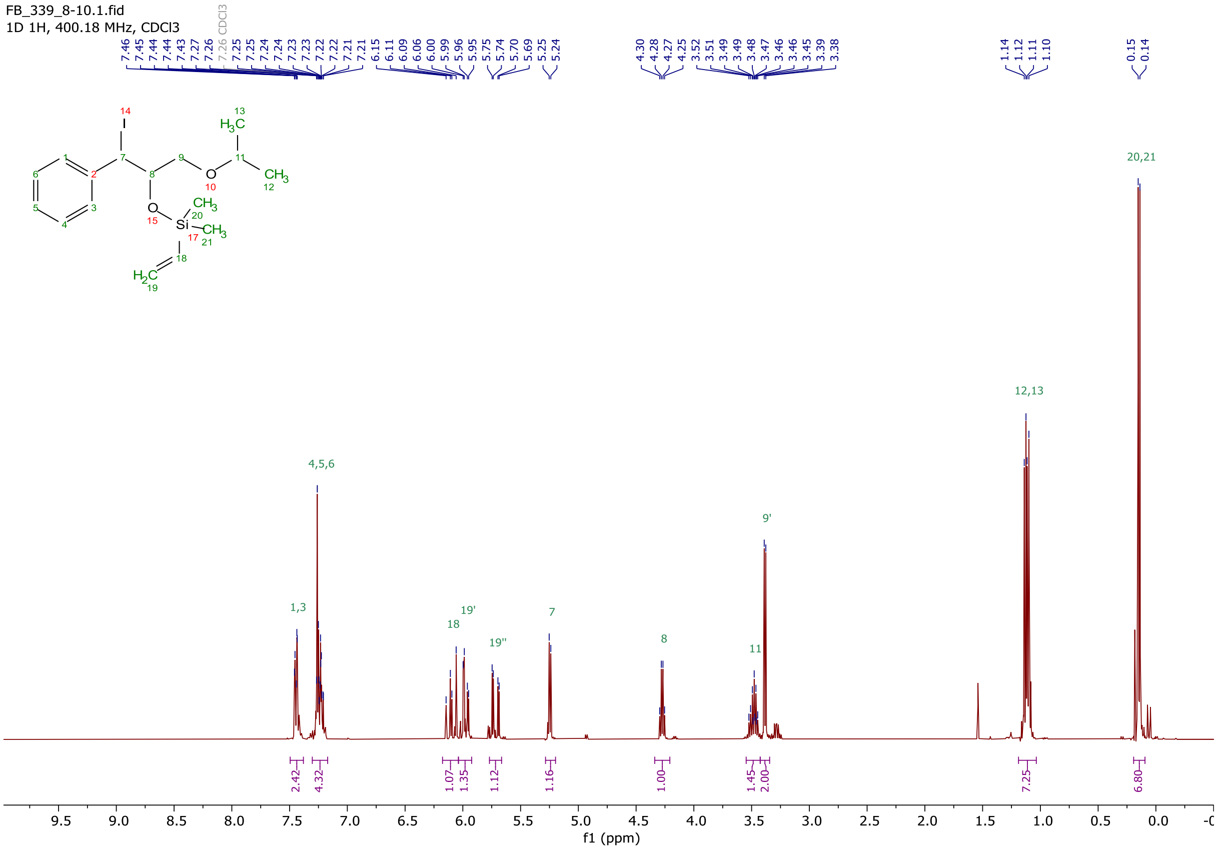

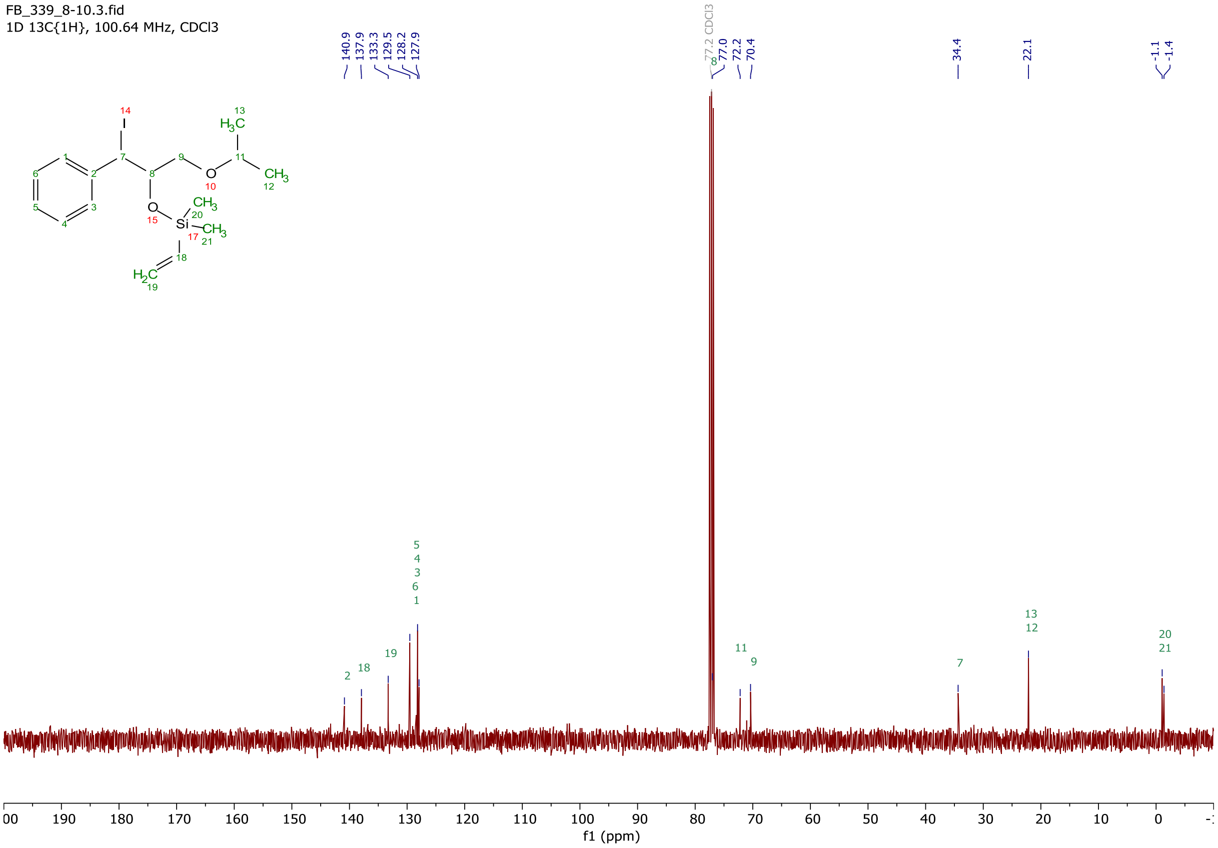

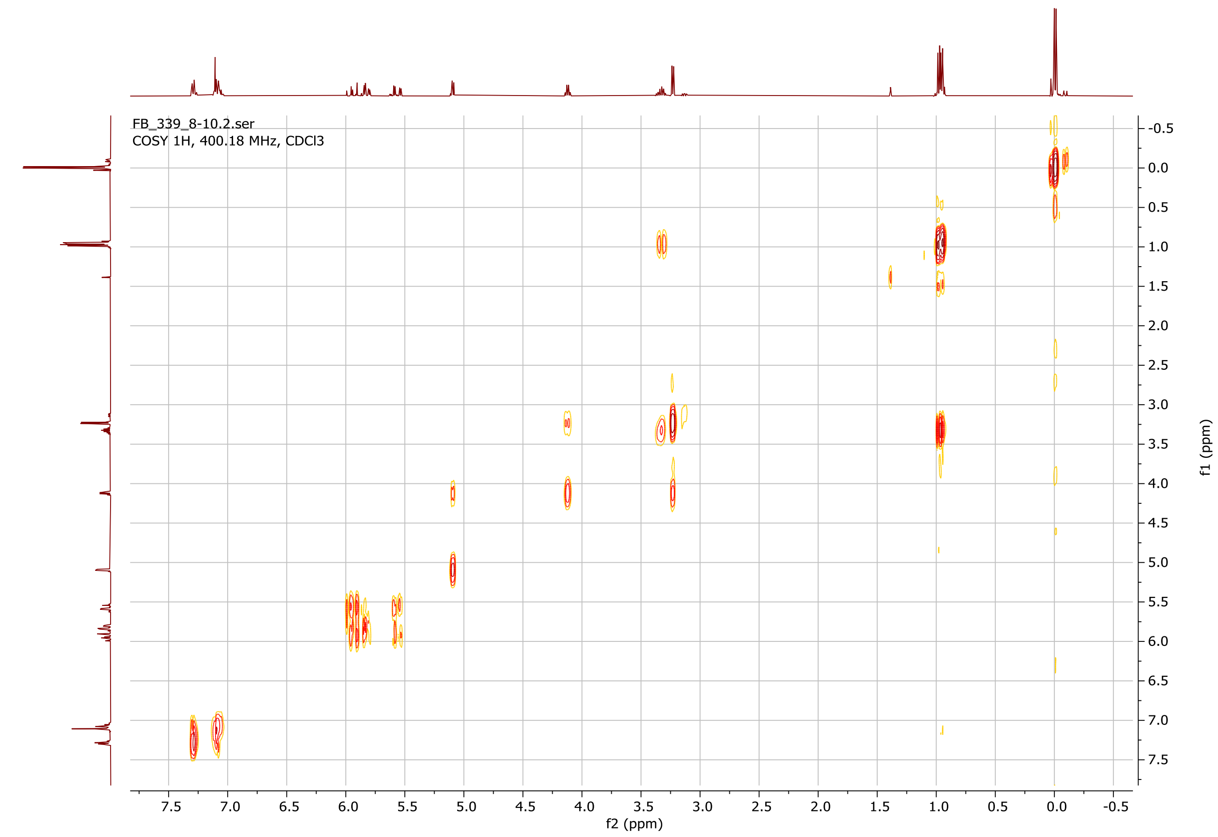
FB_339_8-10.2.ser
COSY 1H, 400.18 MHz, CDCl3
f2 (ppm)
f1 (ppm)

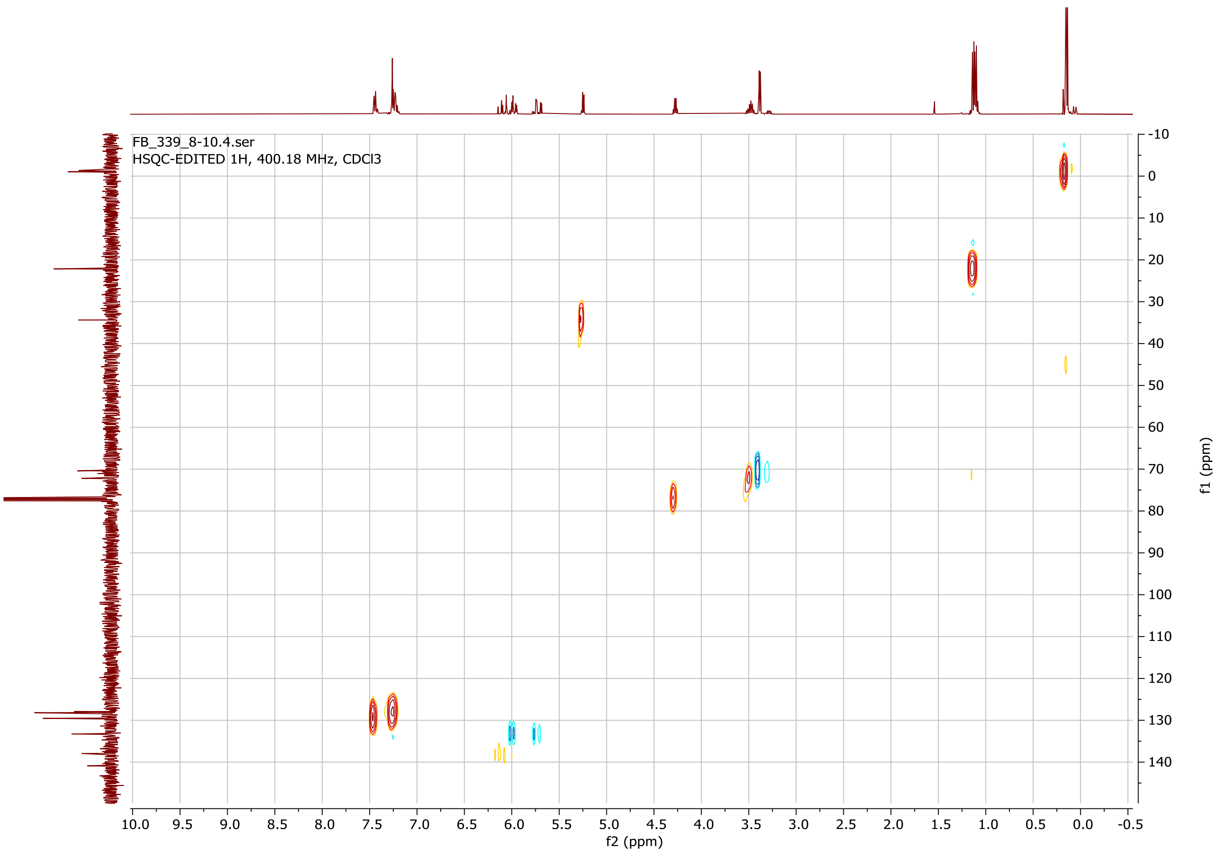
FB_339_8-10.4.ser
HSQC-EDITED 1H, 400.18 MHz, CDCl3
f2 (ppm)
f1 (ppm)

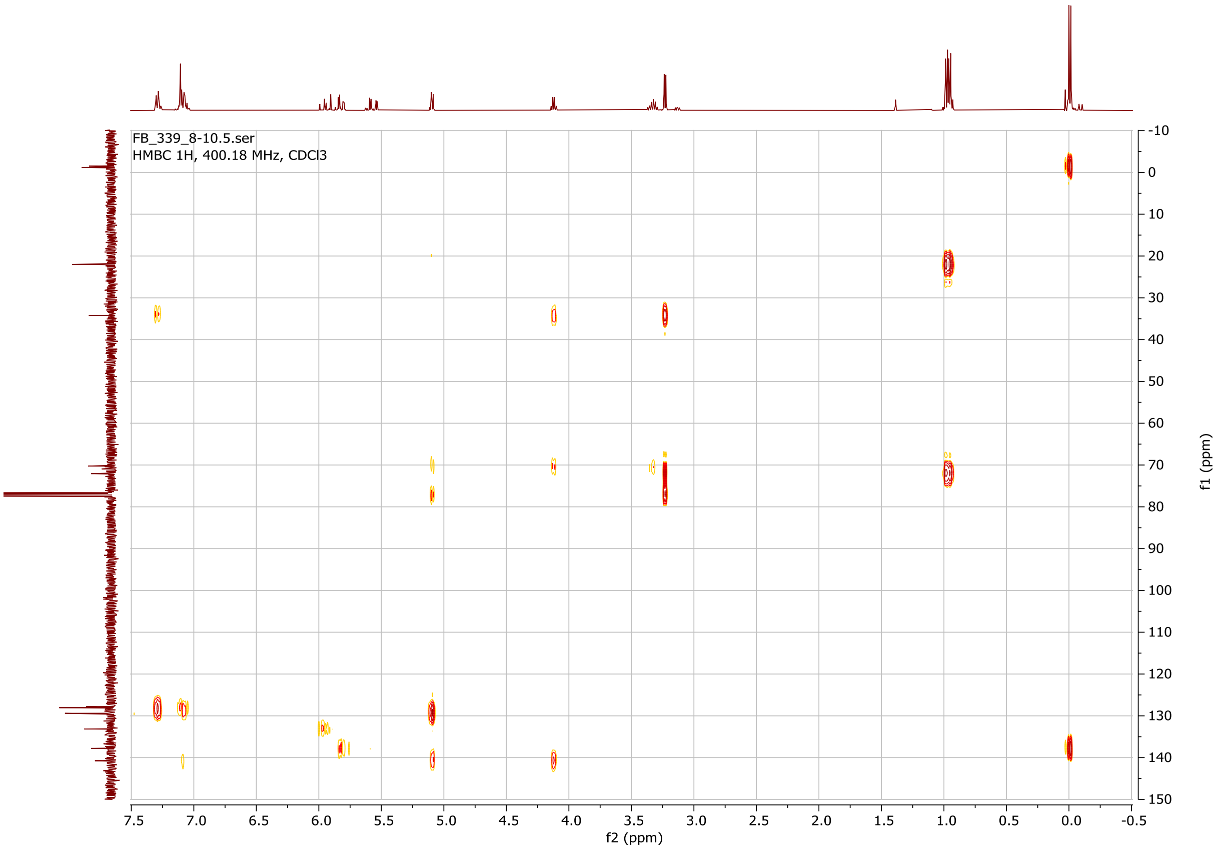

((1-Iodo-3-isopropoxy-1-phenylpropan-2-yl)oxy)dimethylvinylsilane **4s** *(minor of separable 80:20 diastereomers mixture)*

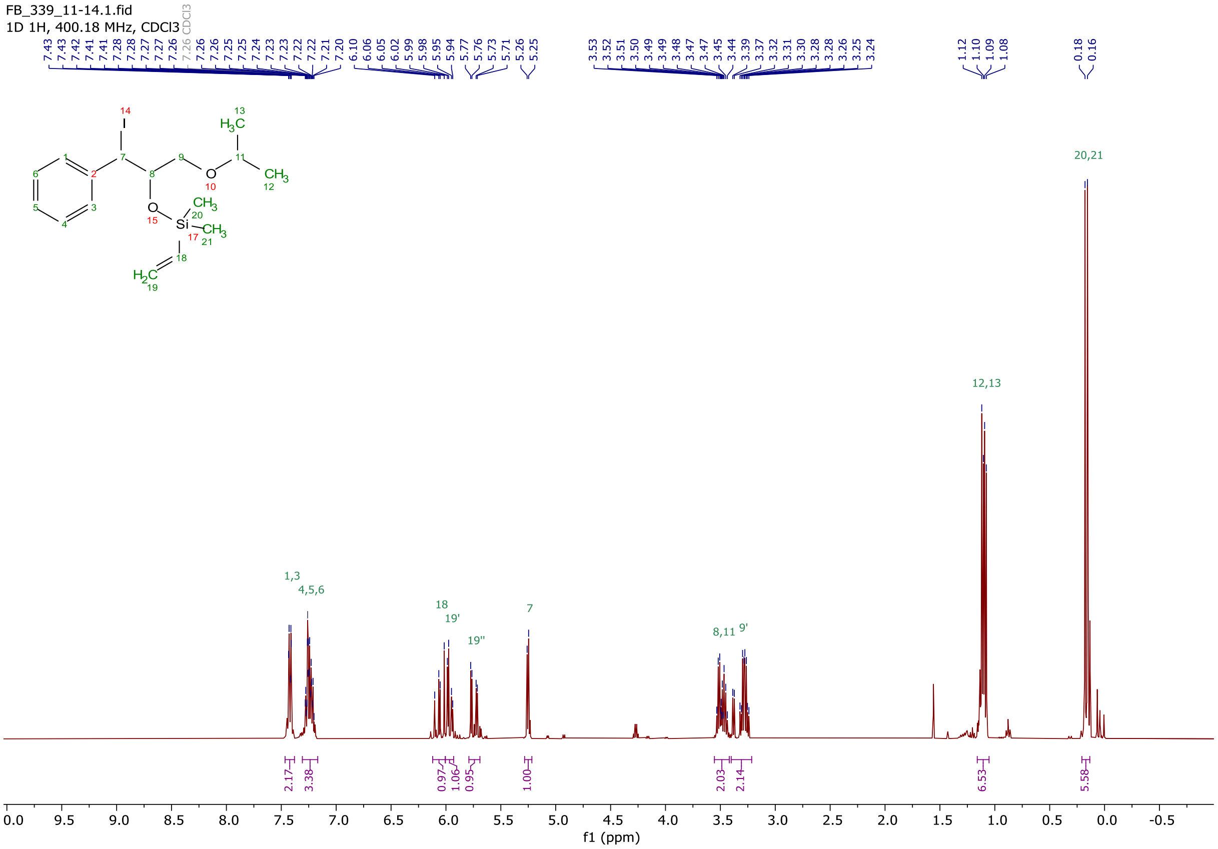

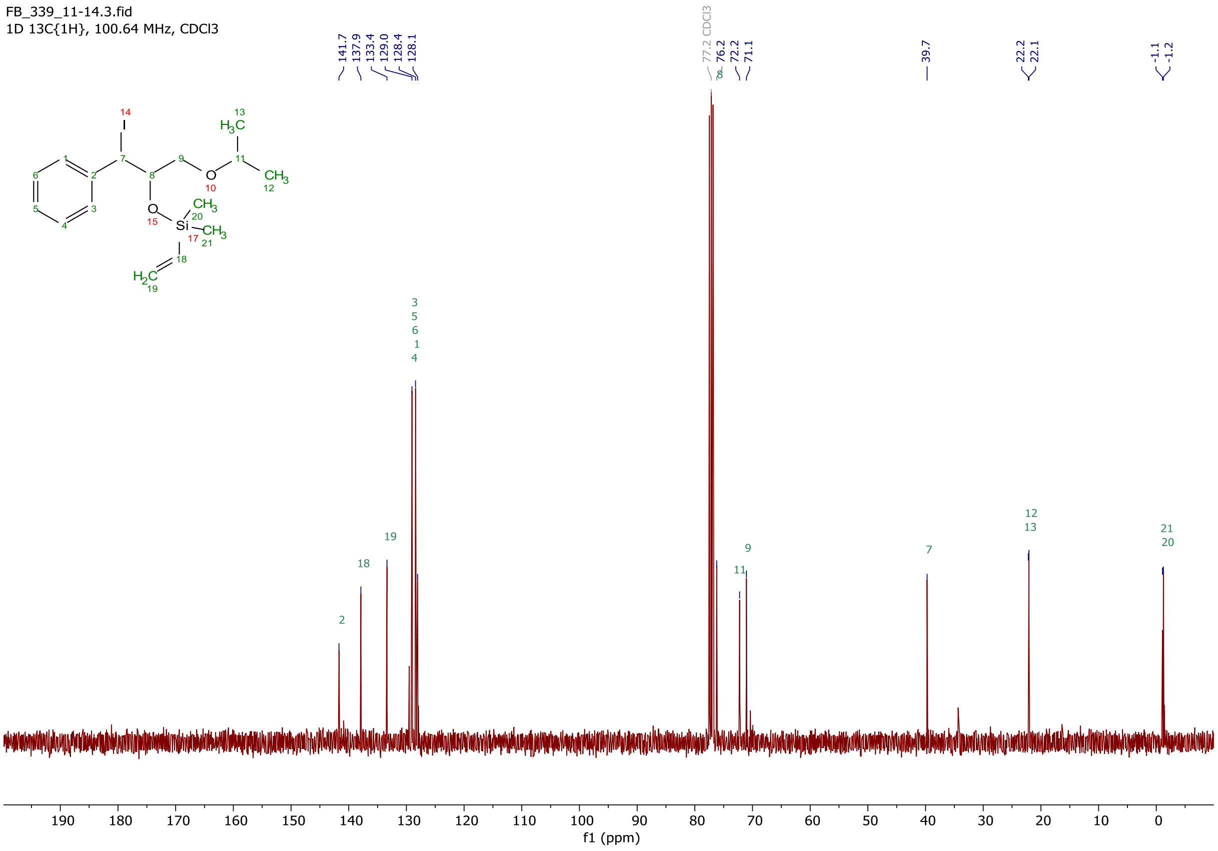
FB_339_11-14.3.fid
1D 13C{1H}, 100.64 MHz, CDCl3
141.7
137.9
133.4
129.0
128.4
128.1
77.2 CDCl3
76.2
72.2
71.1
39.7
22.2
22.1
-1.1
-1.2
f1 (ppm)

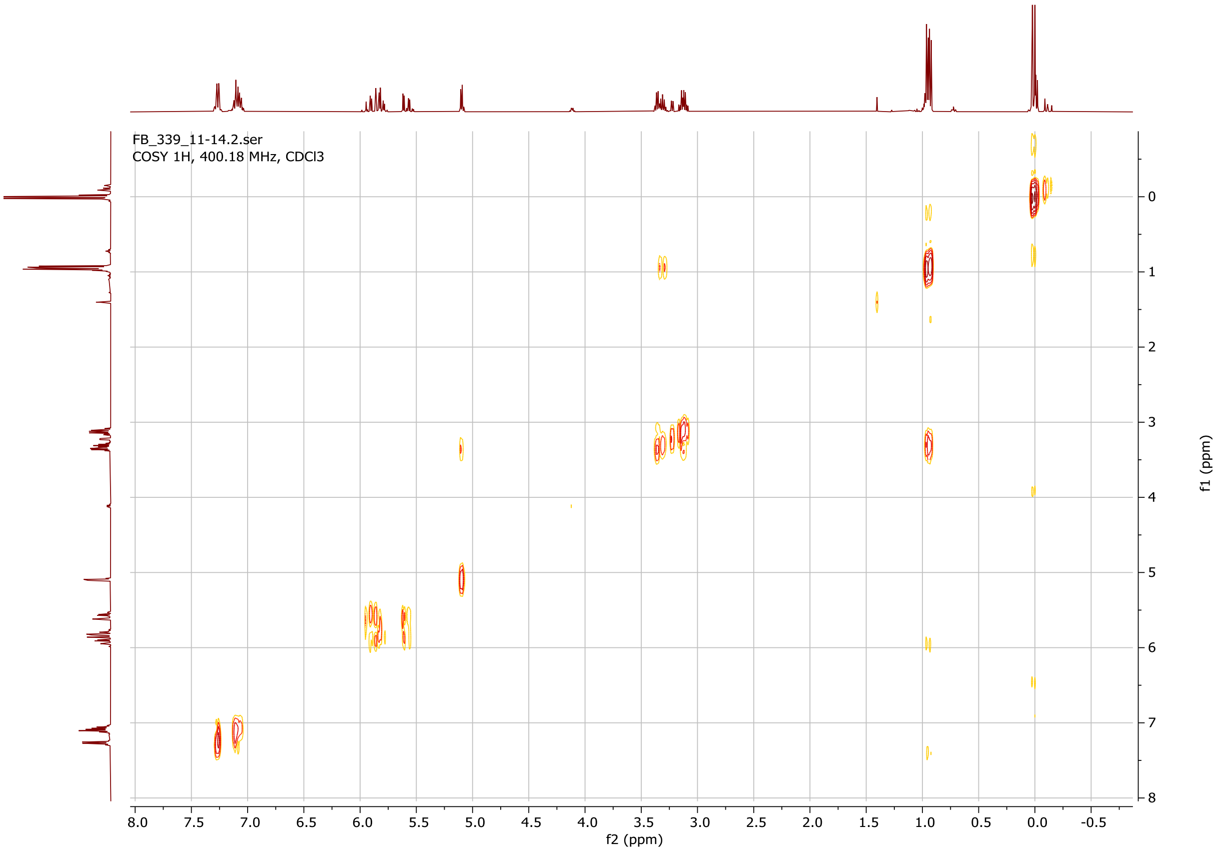
FB_339_11-14.2.ser
COSY 1H, 400.18 MHz, CDCl3
f2 (ppm)
f1 (ppm)

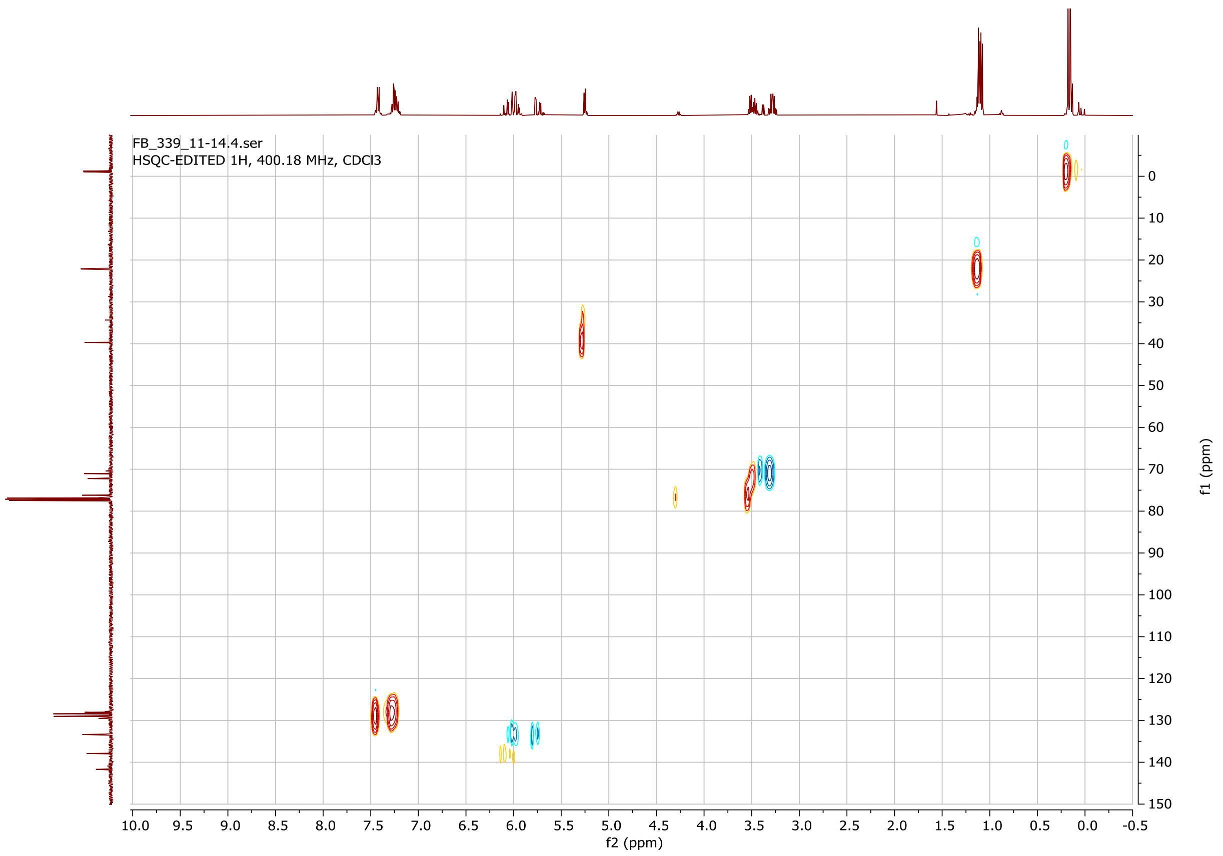
FB_339_11-14.4.ser
HSQC-EDITED 1H, 400.18 MHz, CDCl3
f2 (ppm)
f1 (ppm)

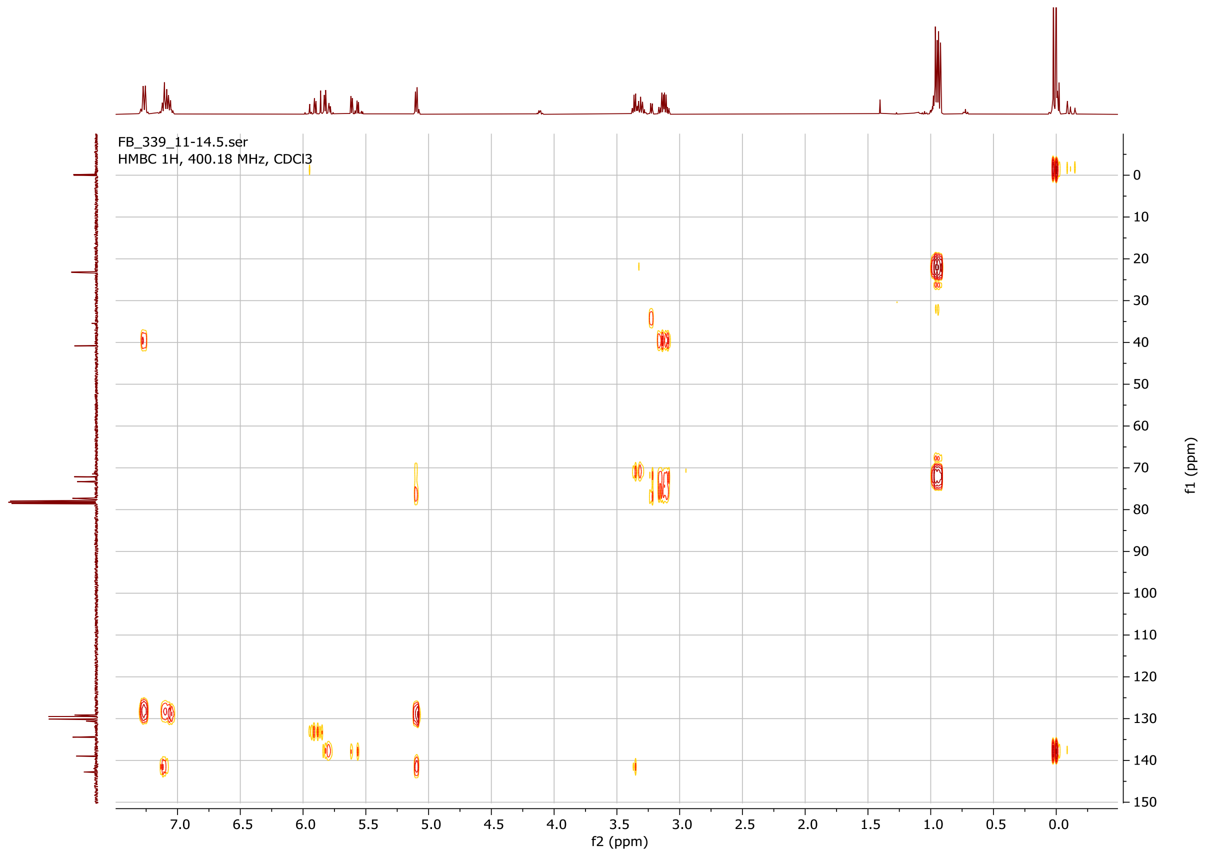
FB_339_11-14.5.ser
HMBC 1H, 400.18 MHz, CDCl3
f2 (ppm)
f1 (ppm)

Methyl 3-((dimethylvinylsilyl)oxy)-2-iodo-3-phenylpropanoate **4t**

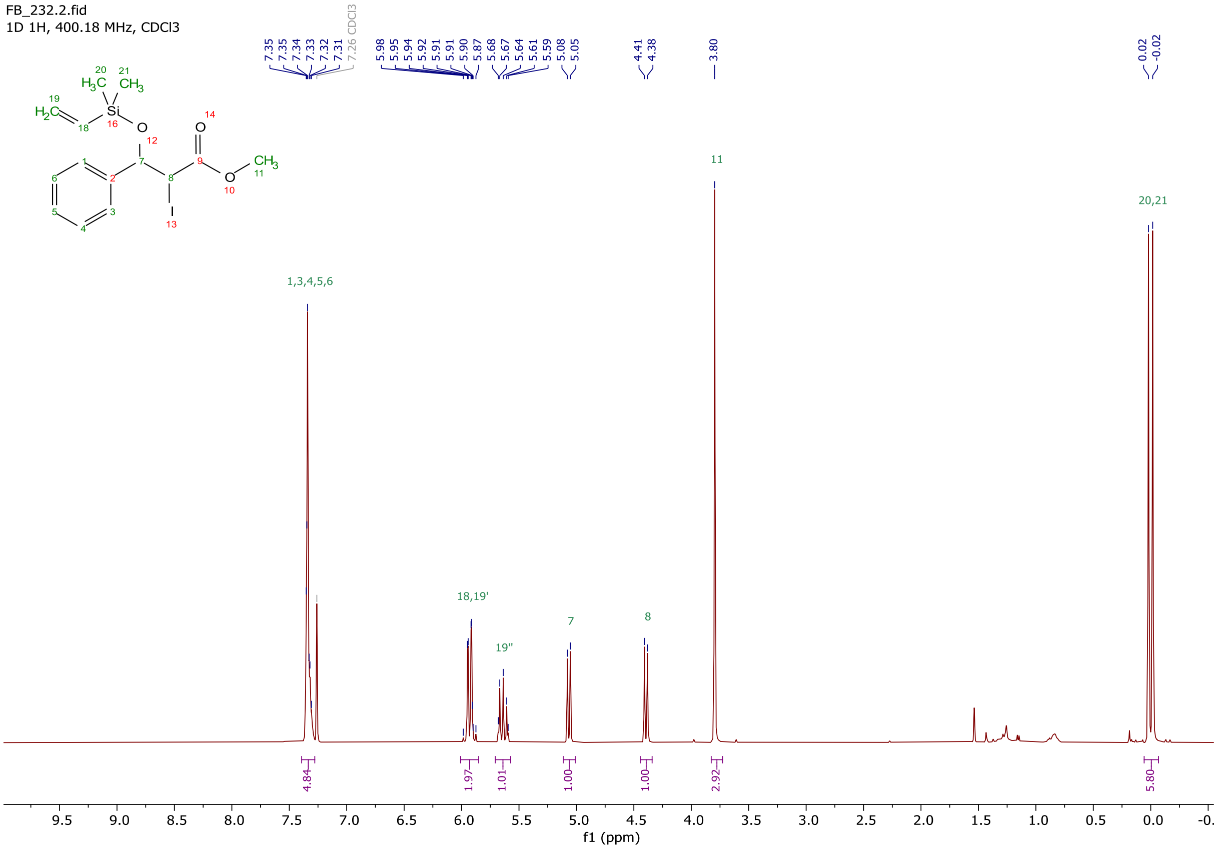

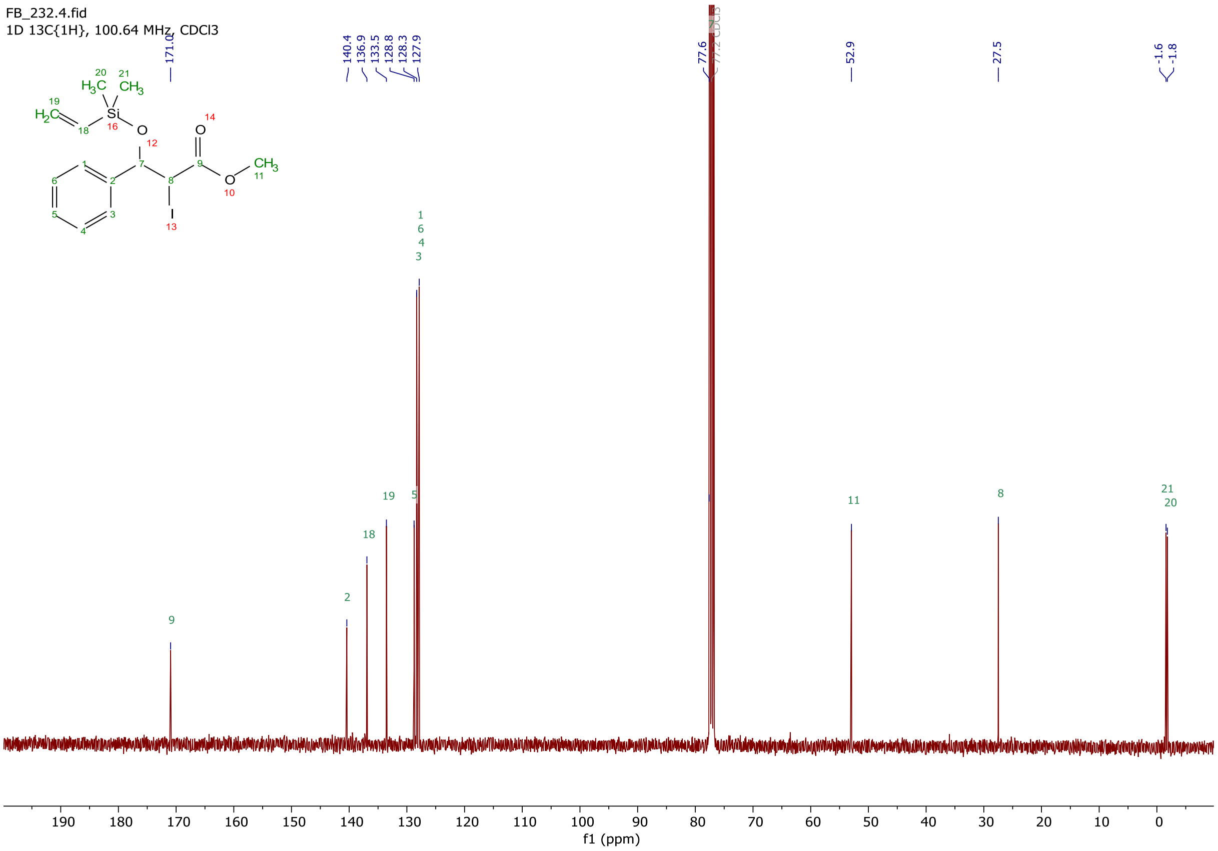

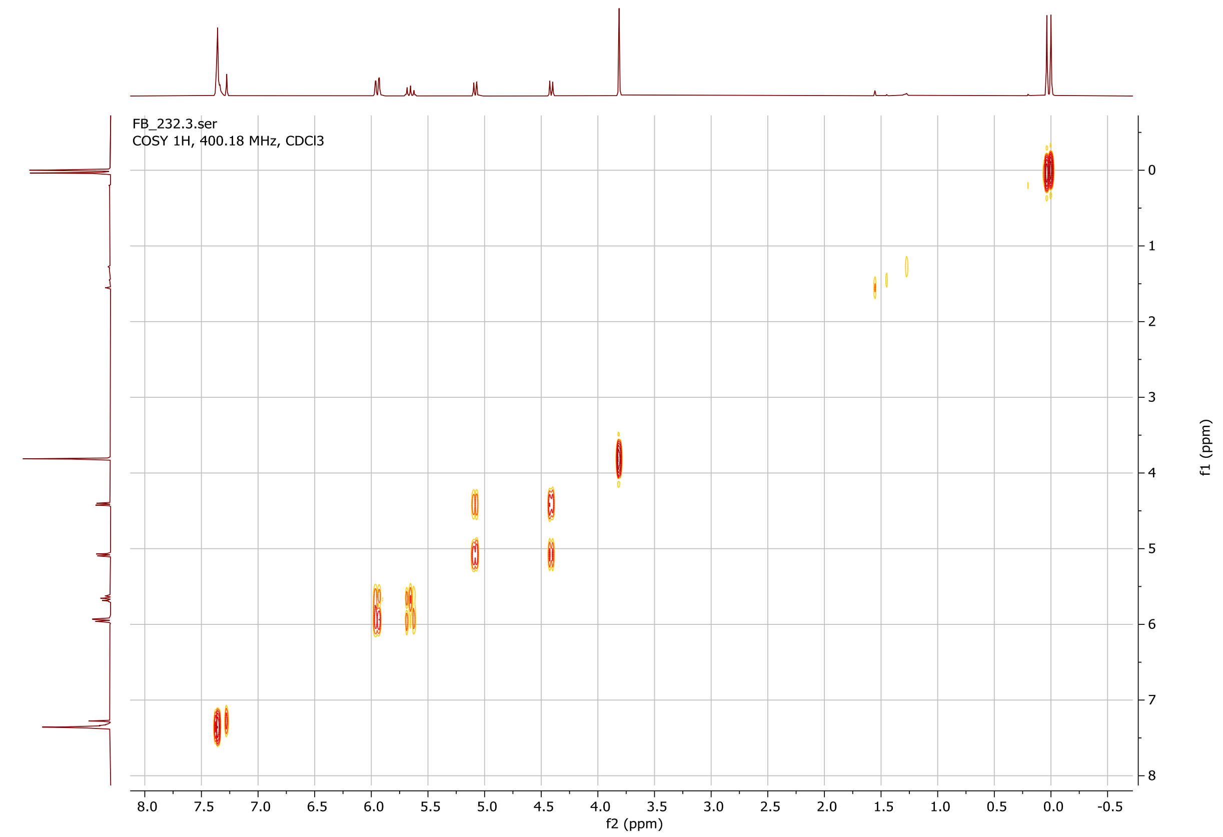
FB_232.3.ser
COSY 1H, 400.18 MHz, CDCl3
f2 (ppm)
f1 (ppm)

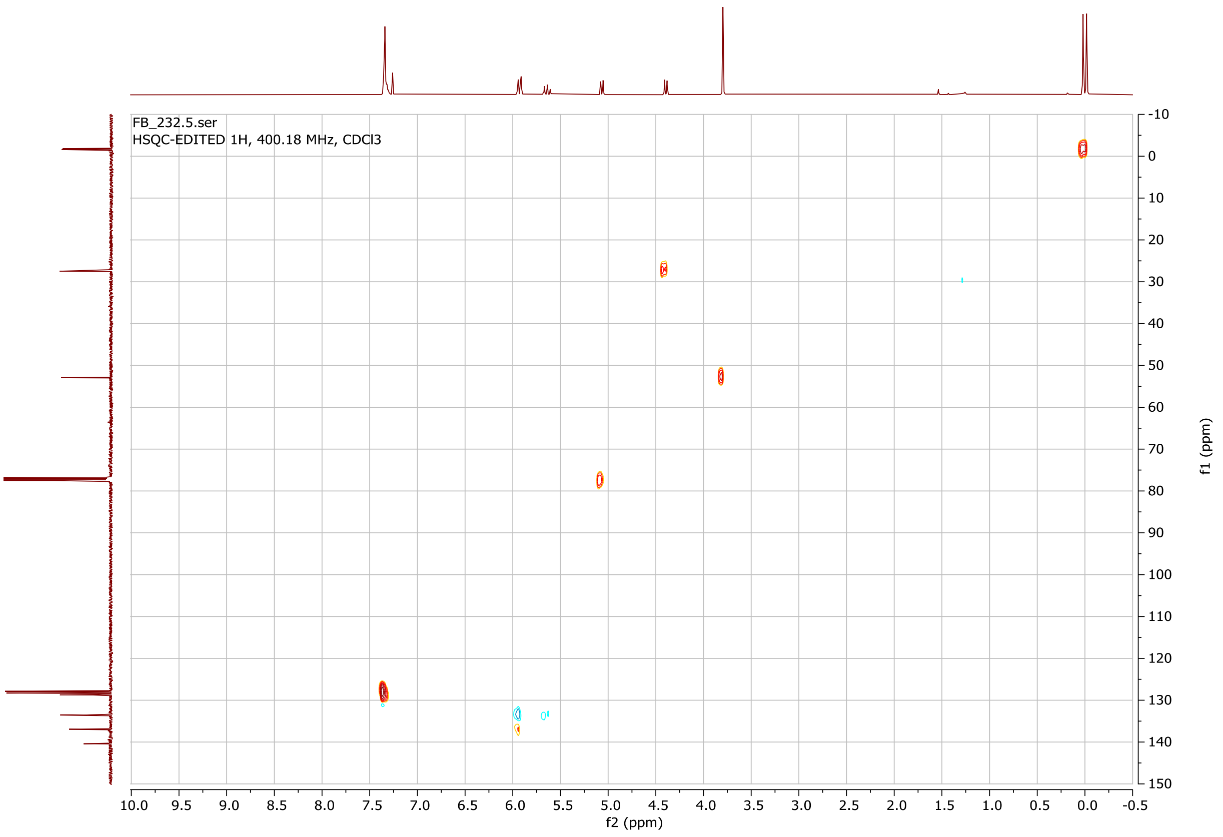
FB_232.5.ser
HSQC-EDITED 1H, 400.18 MHz, CDCl3
f2 (ppm)
f1 (ppm)

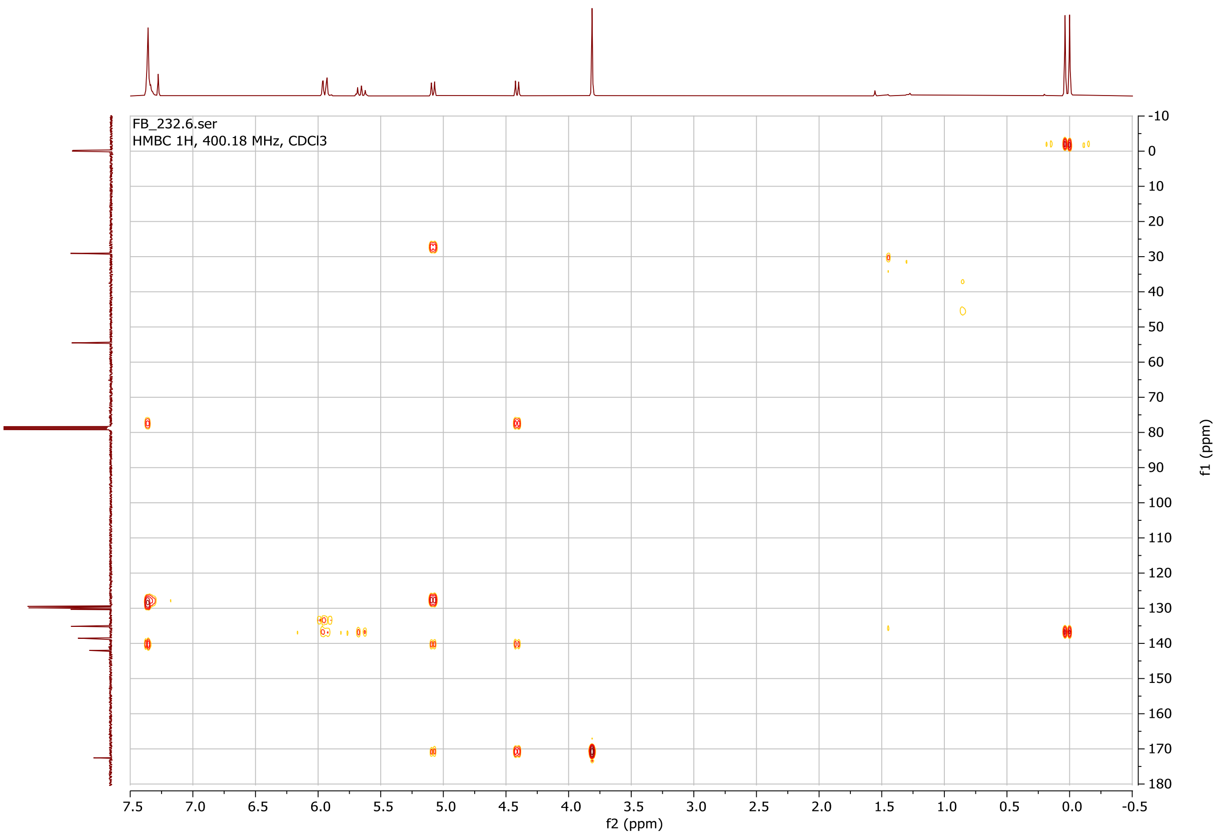

3-((Dimethylvinylsilyl)oxy)-2-iodo-*N*,*N*-dimethyl-3-phenylpropanamide **4u**

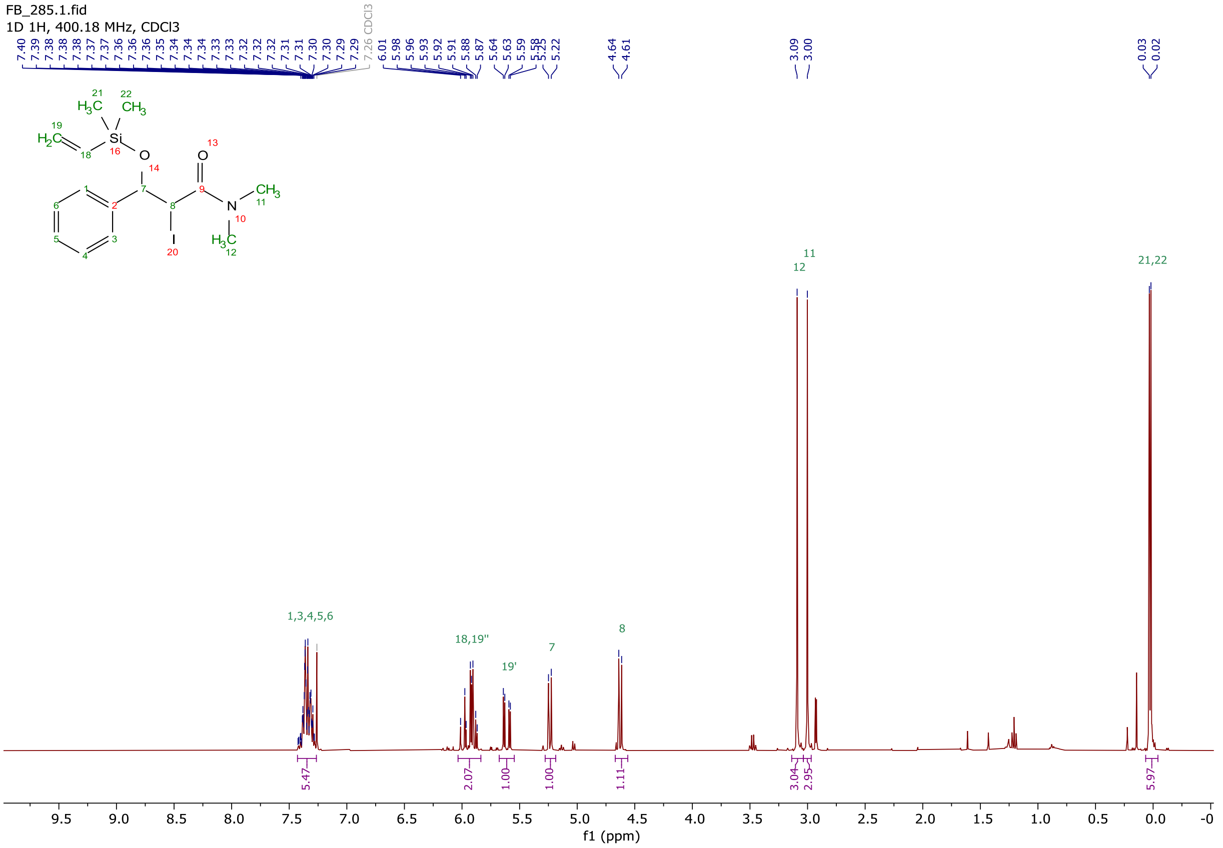

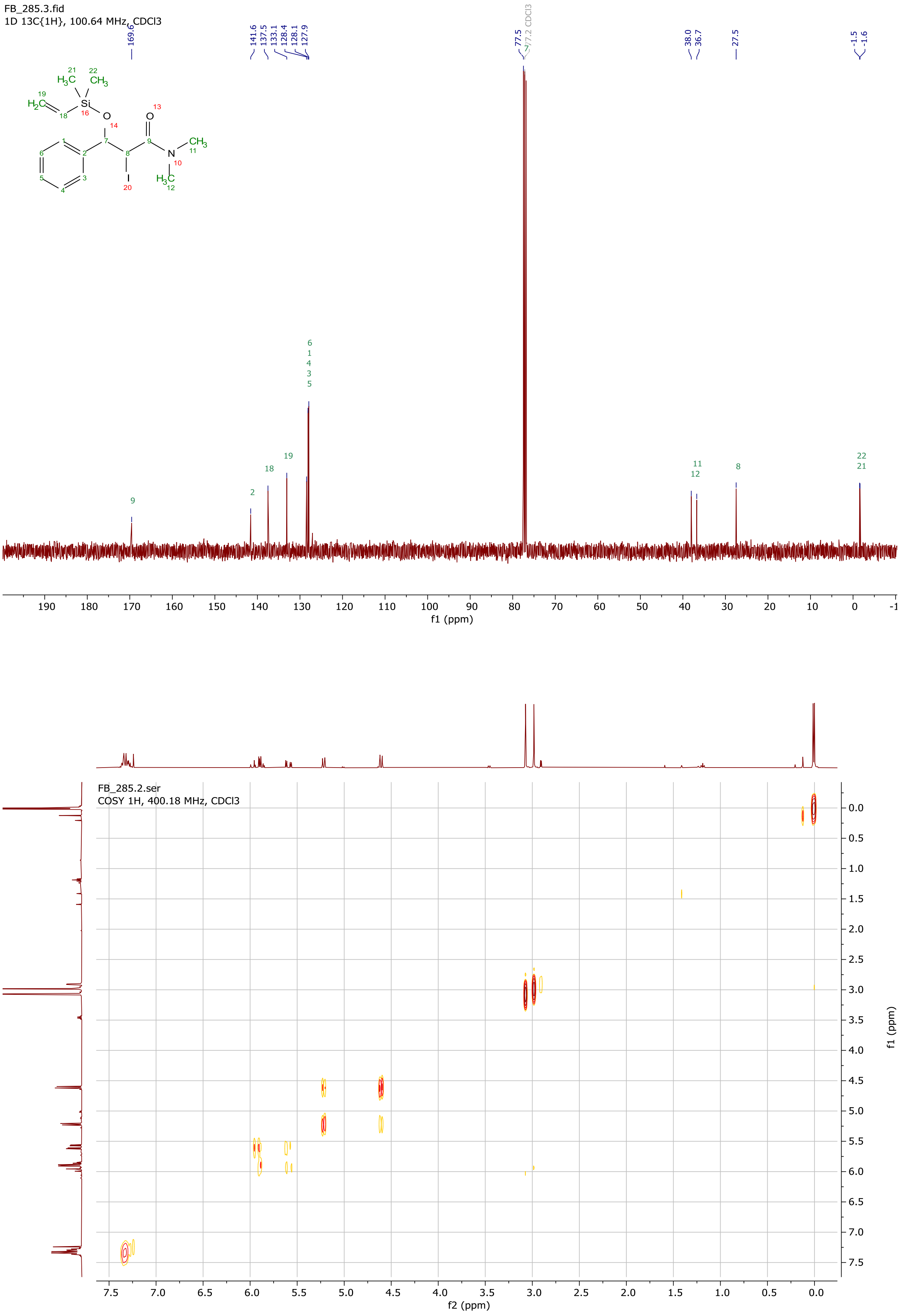

FB_285.3.fid
1D 13C{1H}, 100.64 MHz, CDCl3
169.6
141.6
137.5
133.1
128.4
128.1
127.9
77.5
77.2 CDCl3
38.0
36.7
27.5
-1.5
-1.6
f1 (ppm)
FB_285.2.ser
COSY 1H, 400.18 MHz, CDCl3
f2 (ppm)
f1 (ppm)

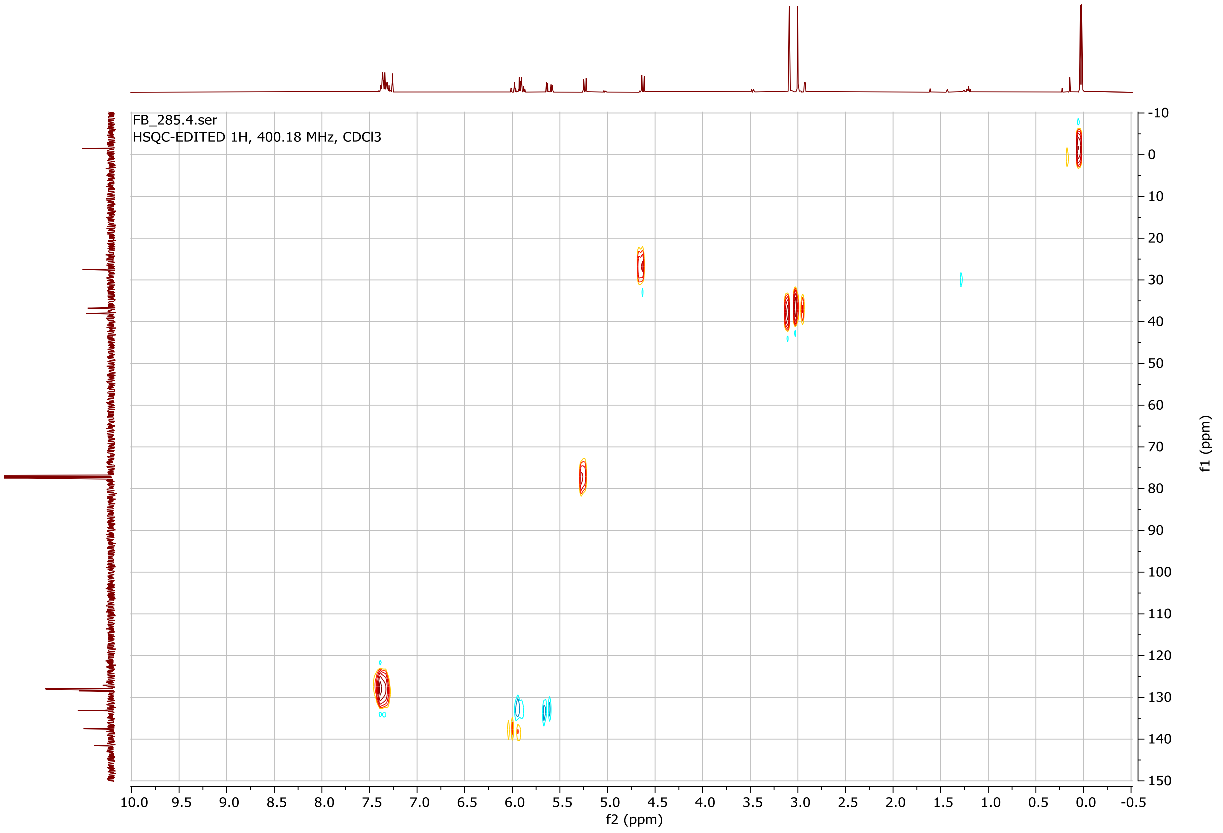
FB_285.4.ser
HSQC-EDITED 1H, 400.18 MHz, CDCl3
f2 (ppm)
f1 (ppm)

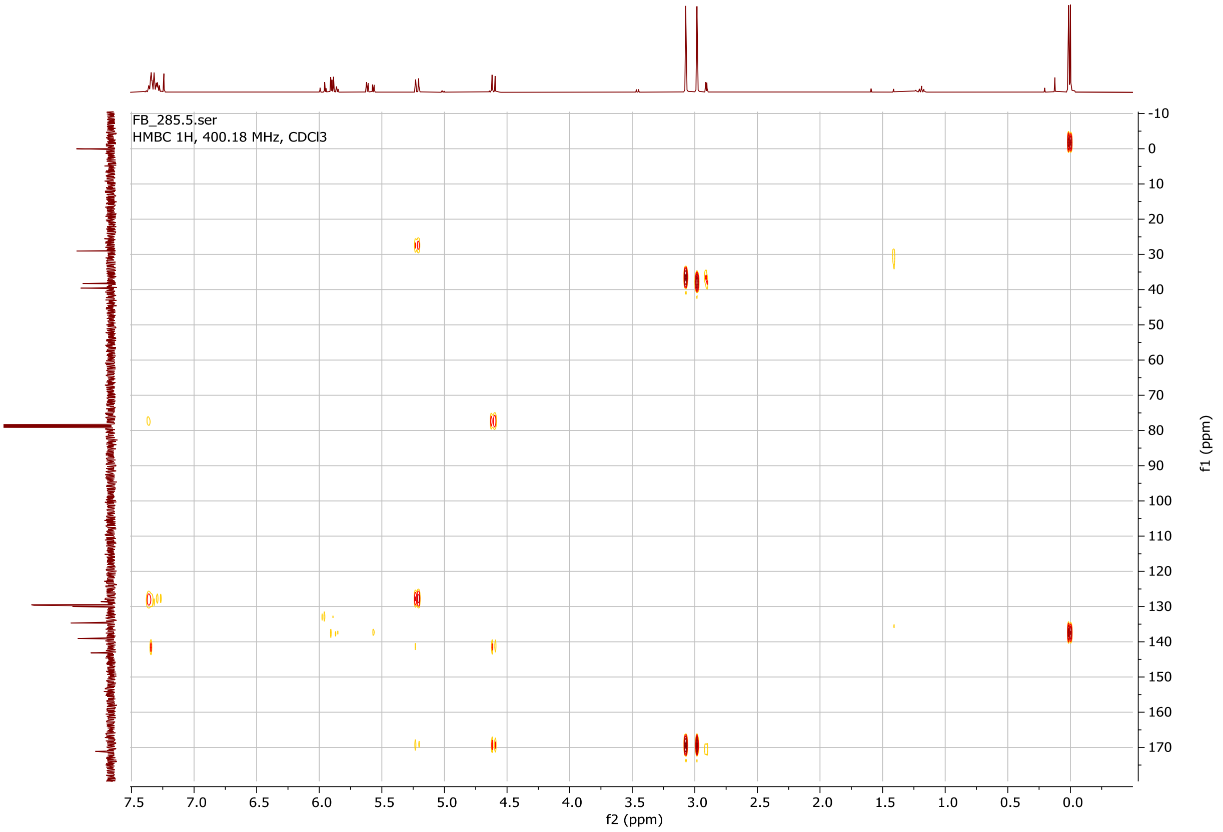
FB_285.5.ser
HMBC 1H, 400.18 MHz, CDCl3
f2 (ppm)
f1 (ppm)

3-((Dimethylvinylsilyl)oxy)-2-iodocyclohexan-1-one **4v** *(major of separable 71:29 diastereomers mixture)*

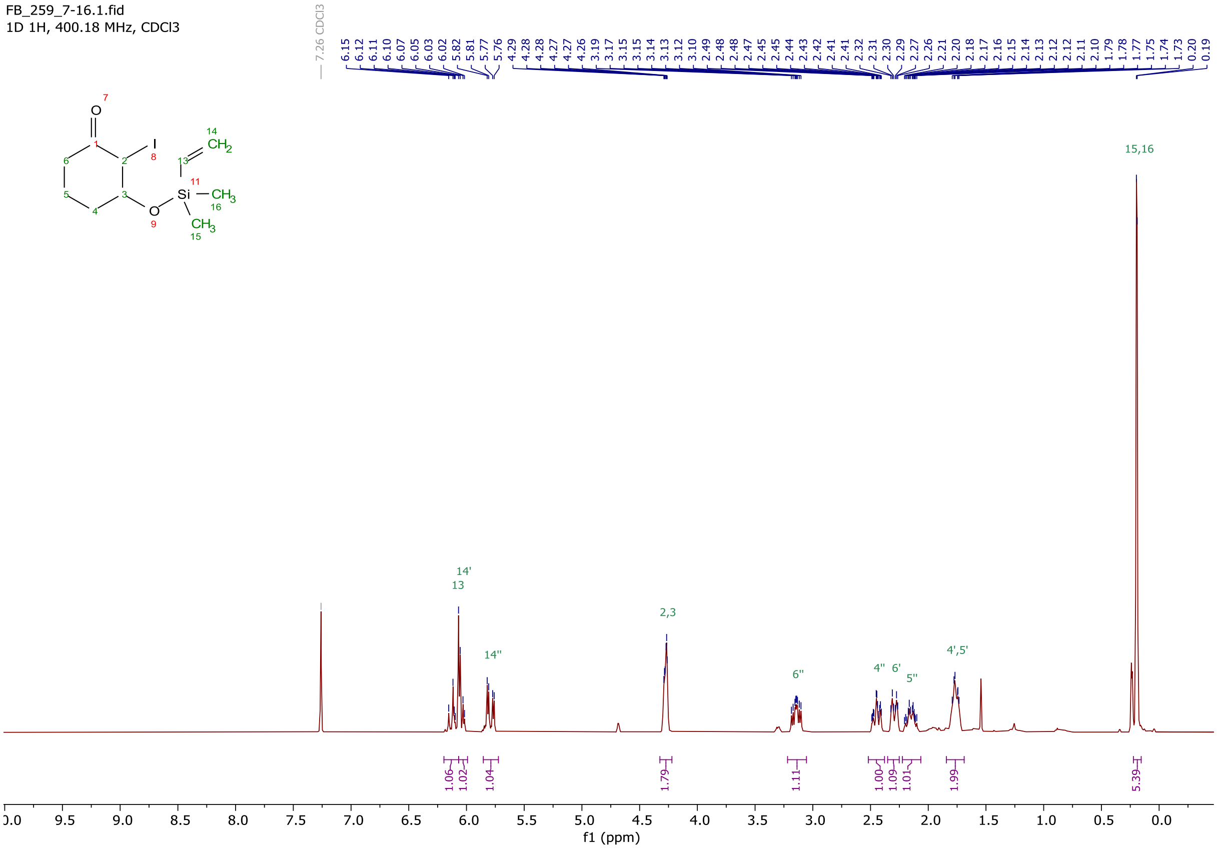

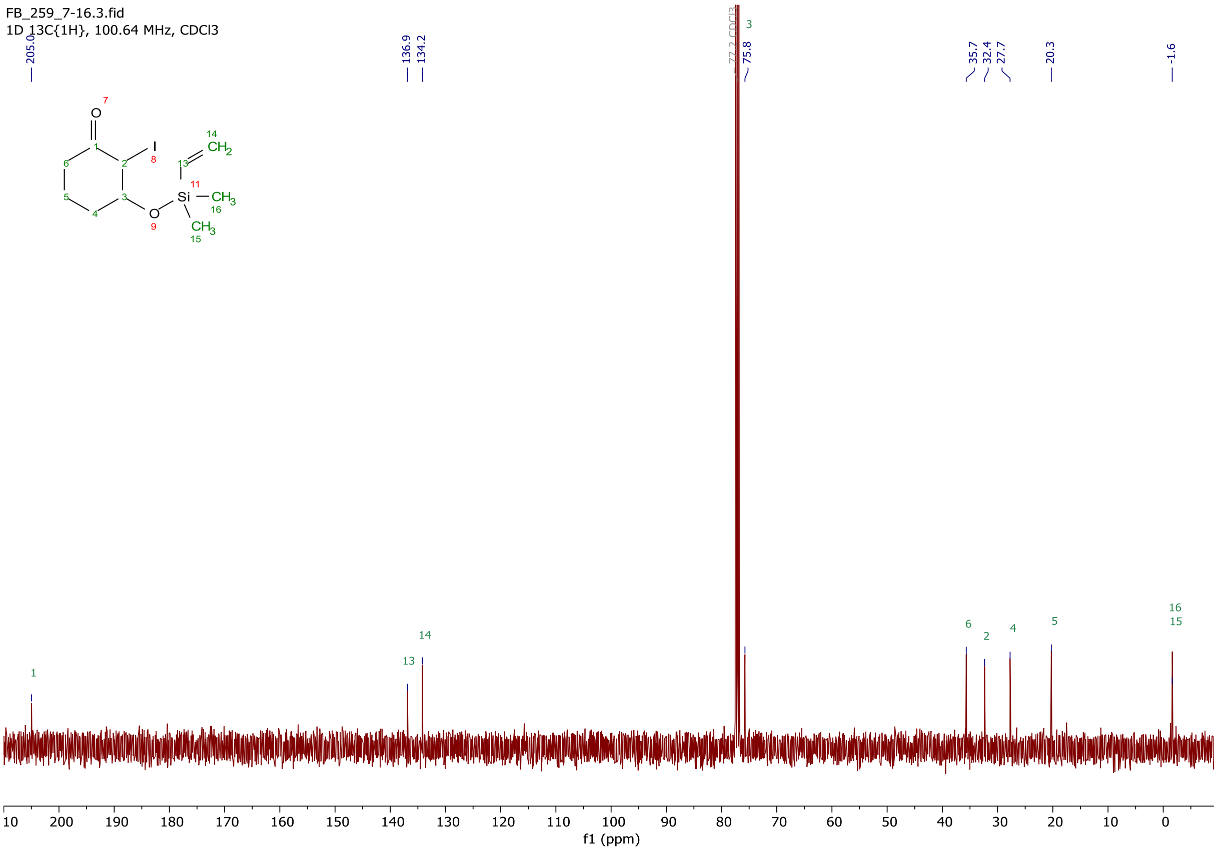

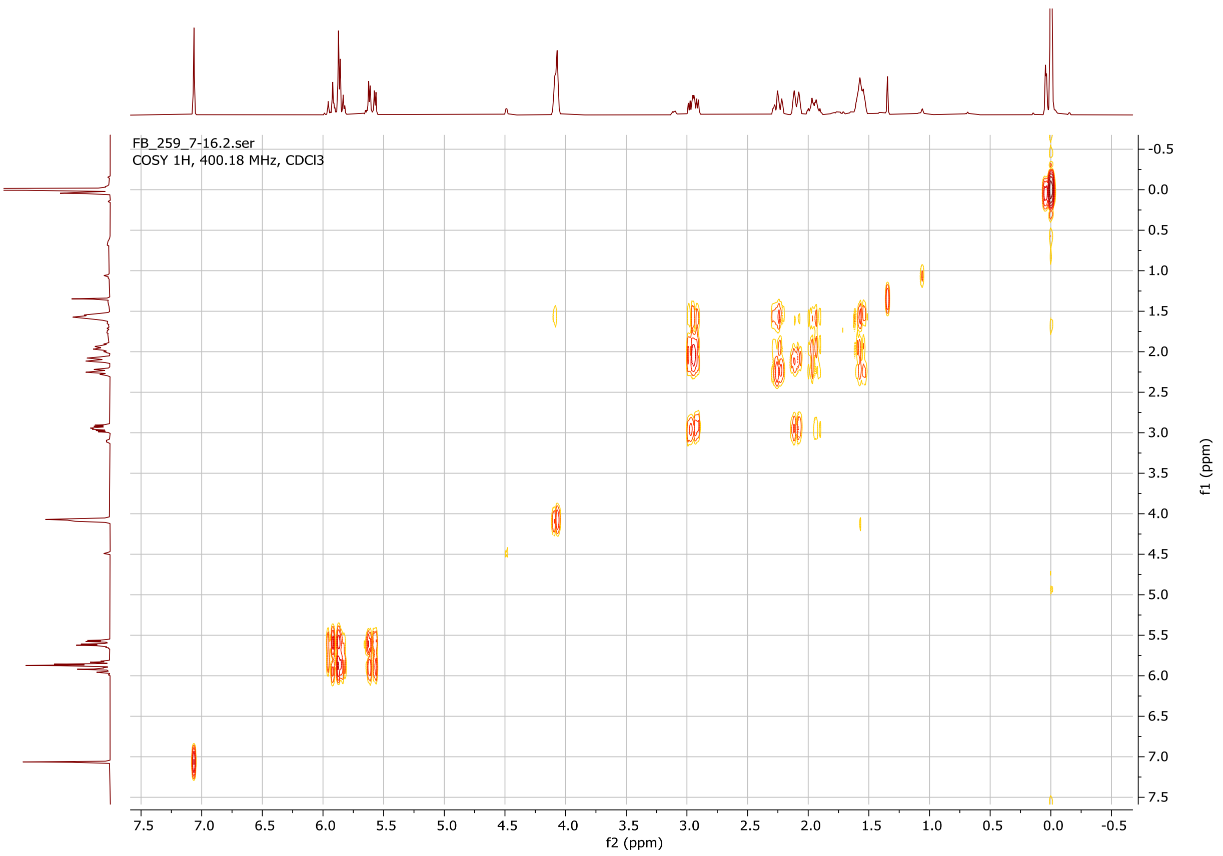
FB_259_7-16.2.ser
COSY 1H, 400.18 MHz, CDCl3
f2 (ppm)
f1 (ppm)

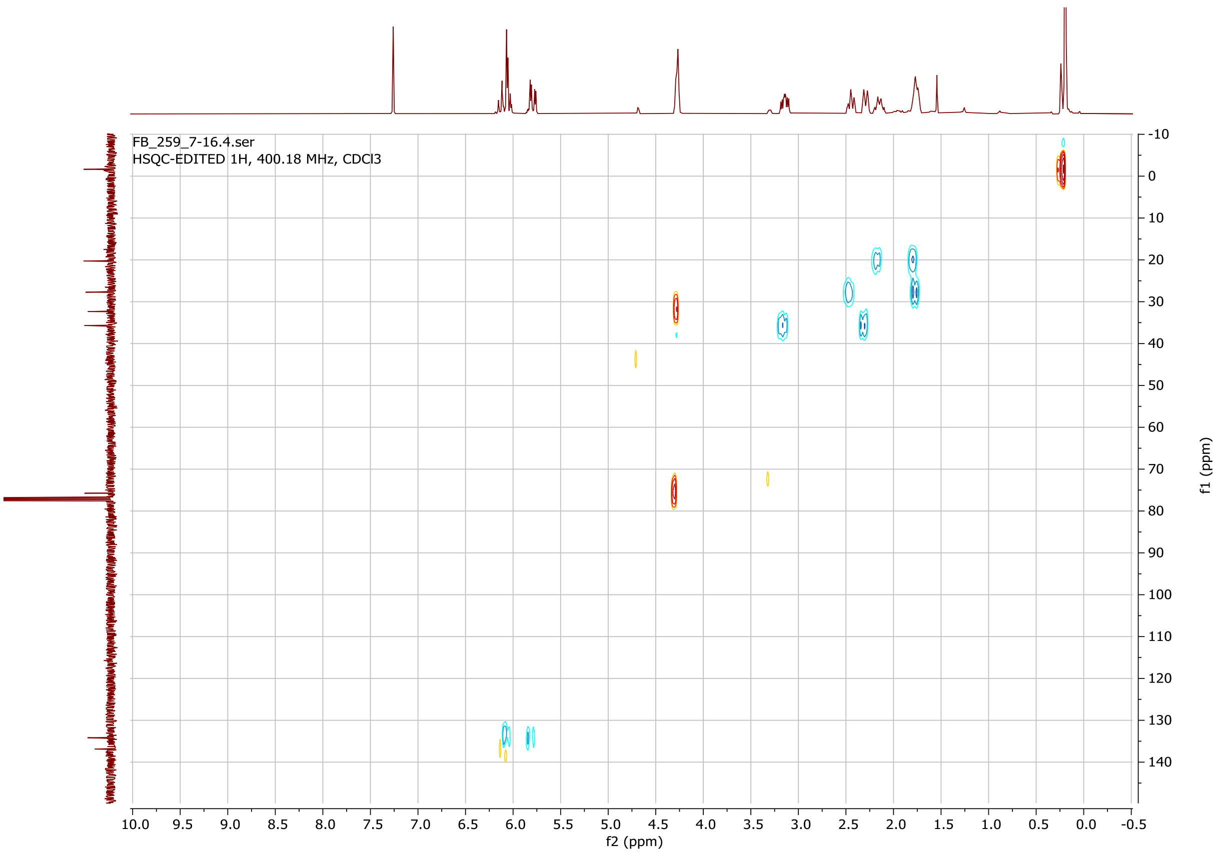
FB_259_7-16.4.ser
HSQC-EDITED 1H, 400.18 MHz, CDCl3
f2 (ppm)
f1 (ppm)

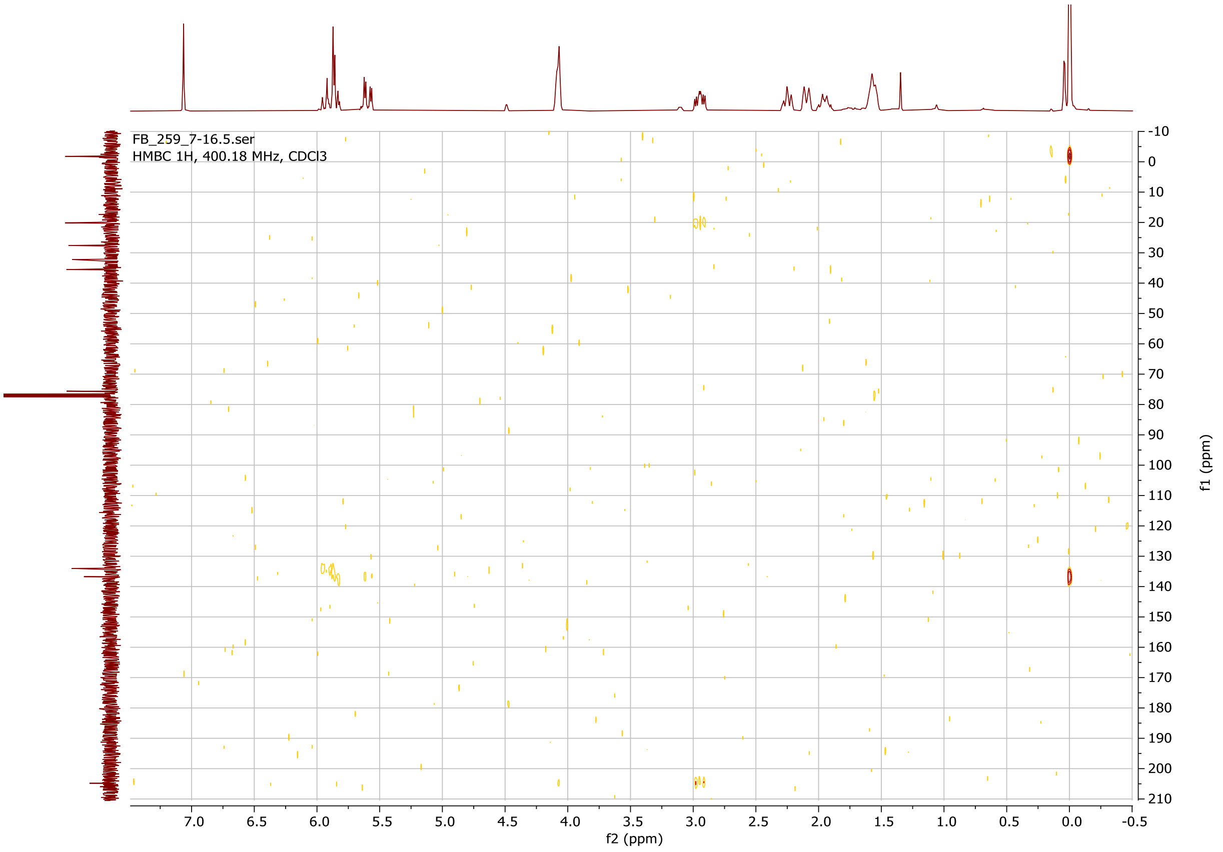

3-((Dimethylvinylsilyl)oxy)-2-iodocyclohexan-1-one **4v** *(minor of separable 71:29 diastereomers mixture)*

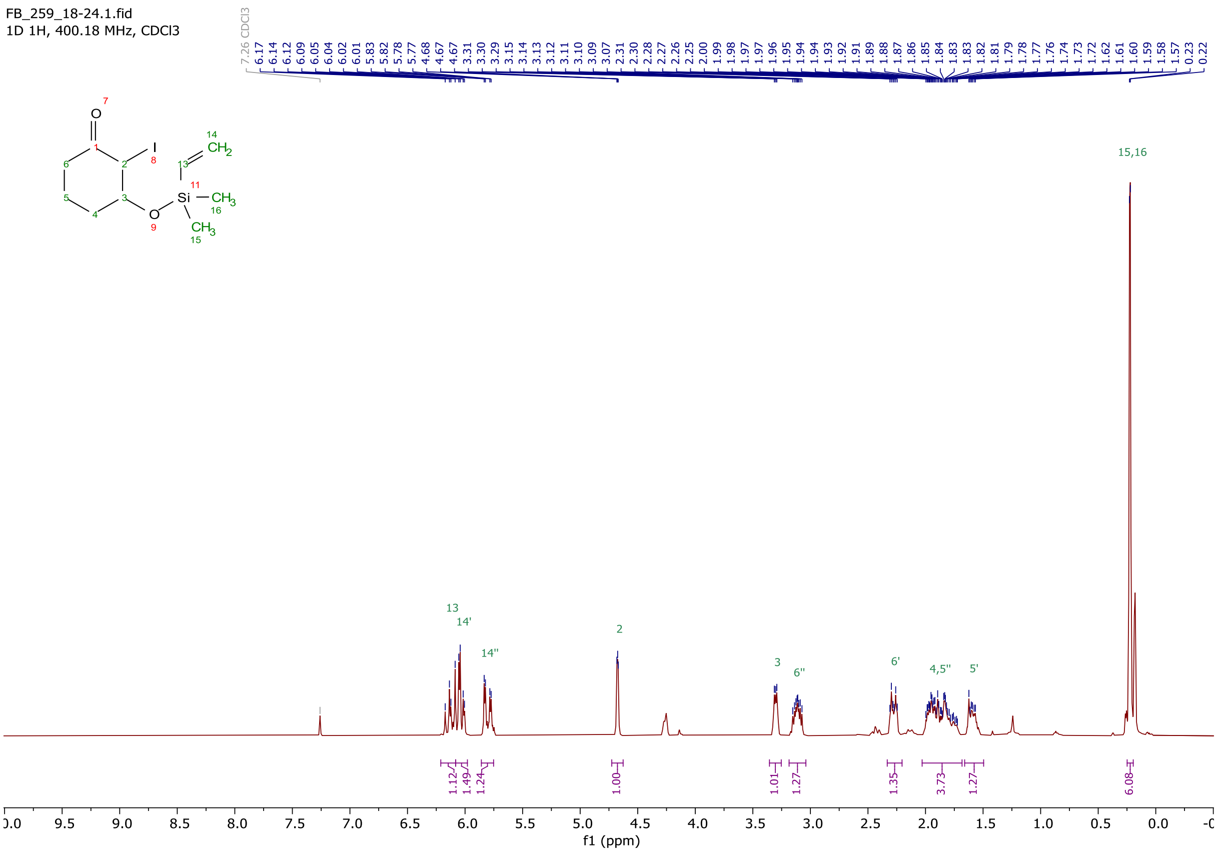

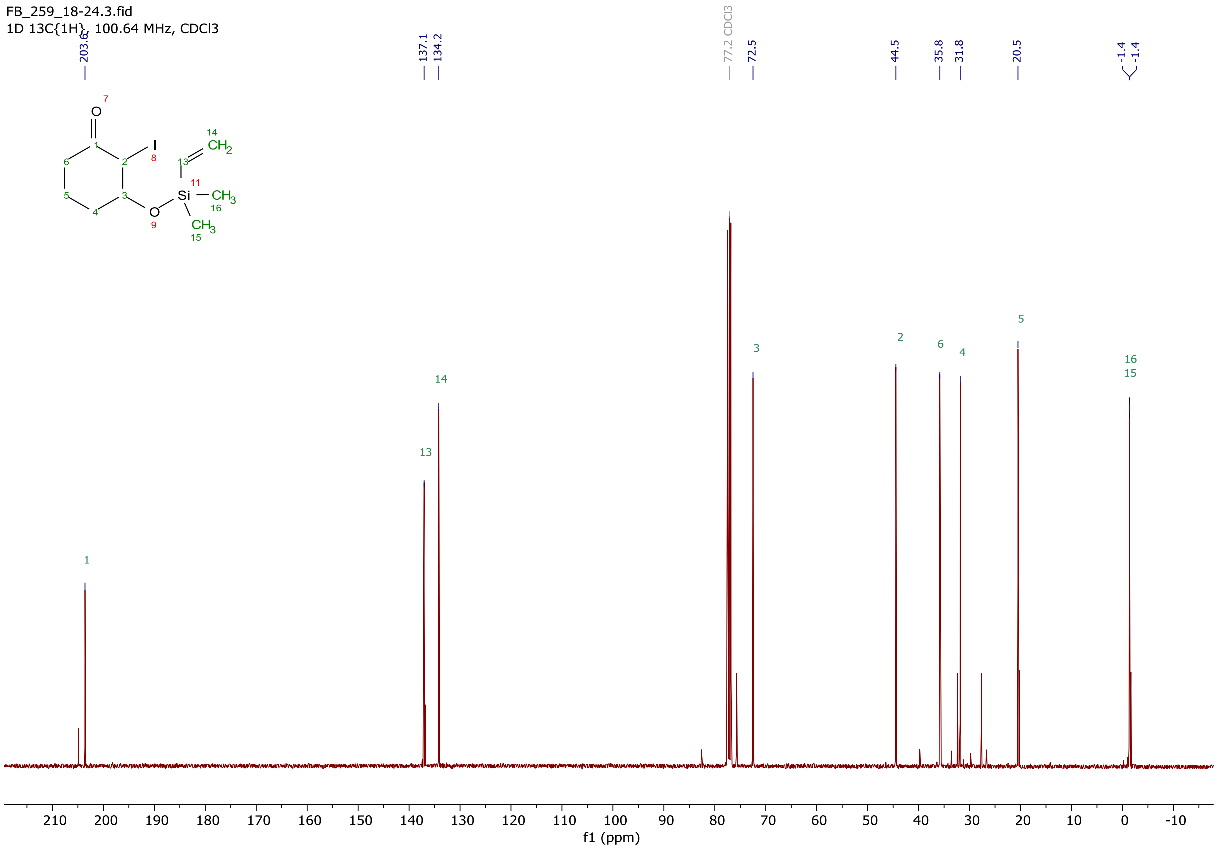
FB_259_18-24.3.fid
1D 13C{1H}, 100.64 MHz, CDCl3
203.6
137.1
134.2
77.2 CDCl3
72.5
44.5
35.8
31.8
20.5
-1.4
-1.4
f1 (ppm)

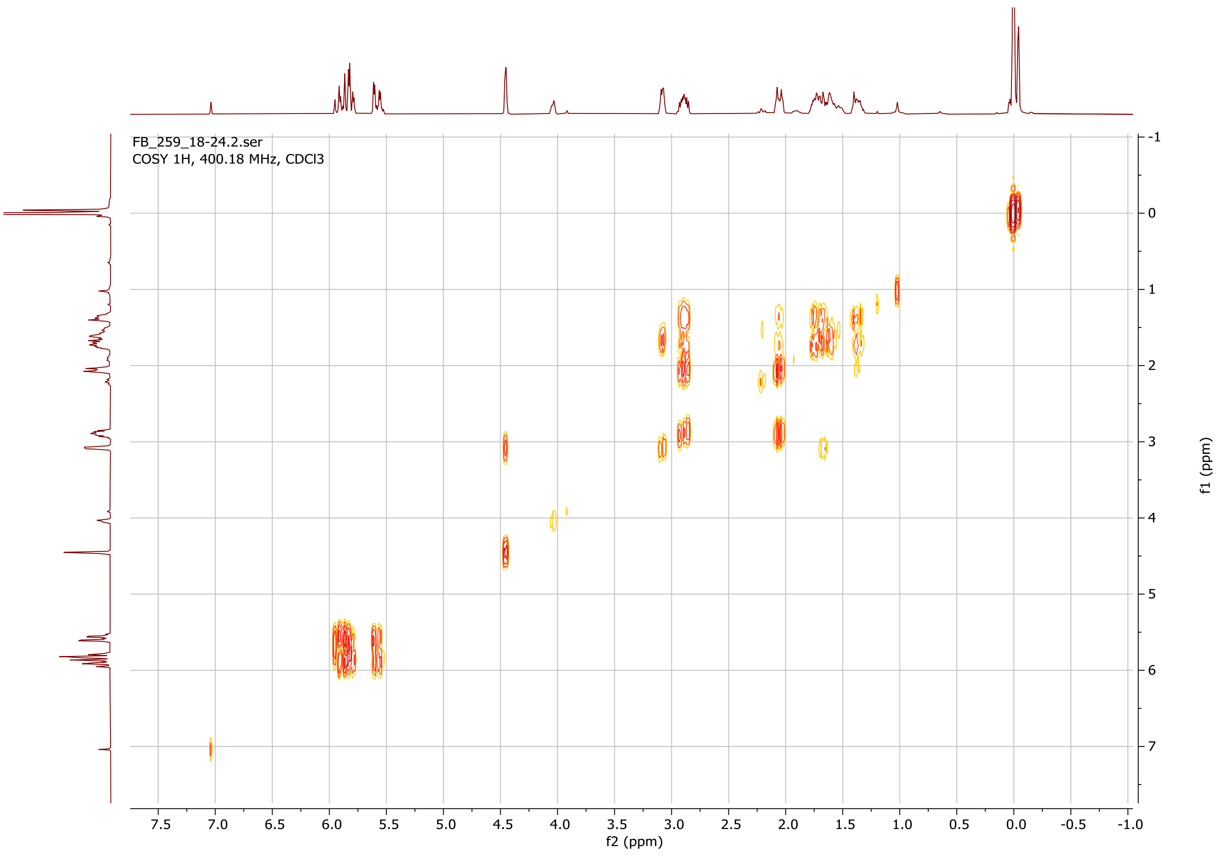
FB_259_18-24.2.ser
COSY 1H, 400.18 MHz, CDCl3
f2 (ppm)
f1 (ppm)

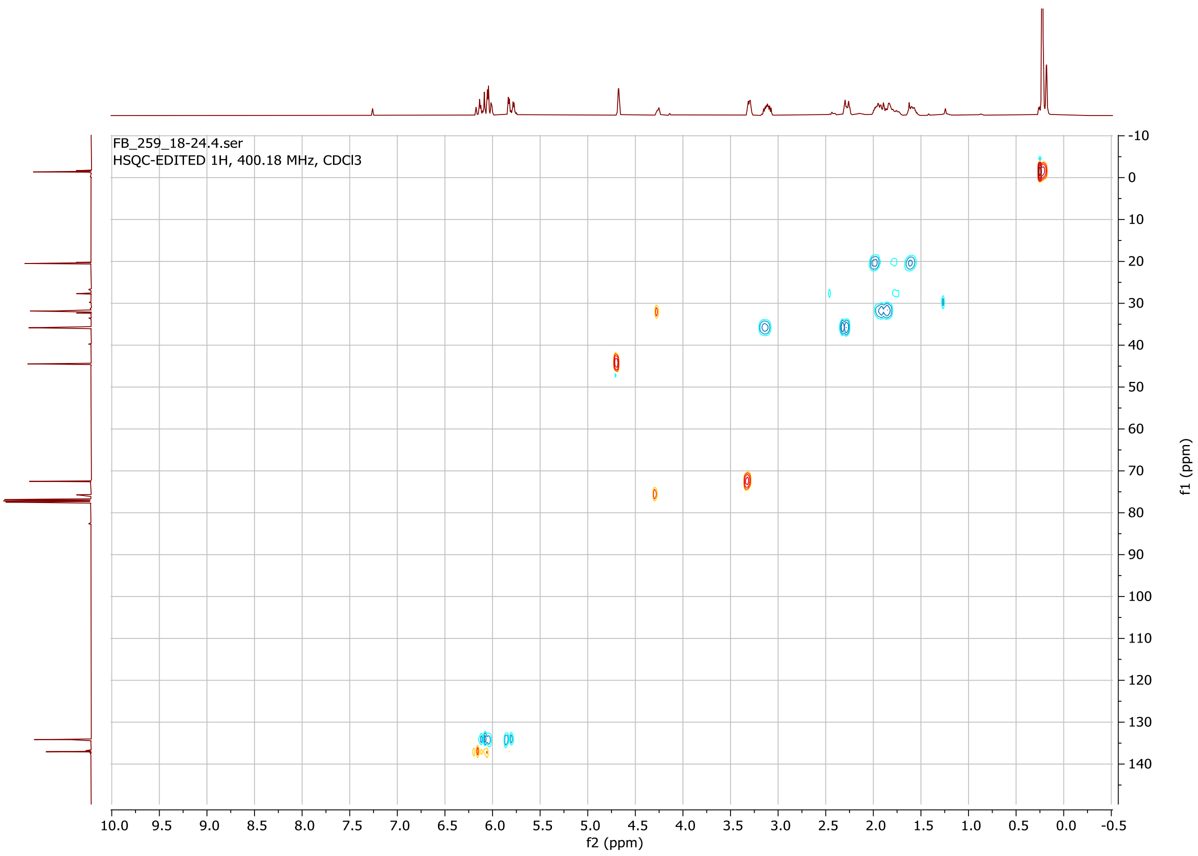
FB_259_18-24.4.ser
HSQC-EDITED 1H, 400.18 MHz, CDCl3
f2 (ppm)
f1 (ppm)

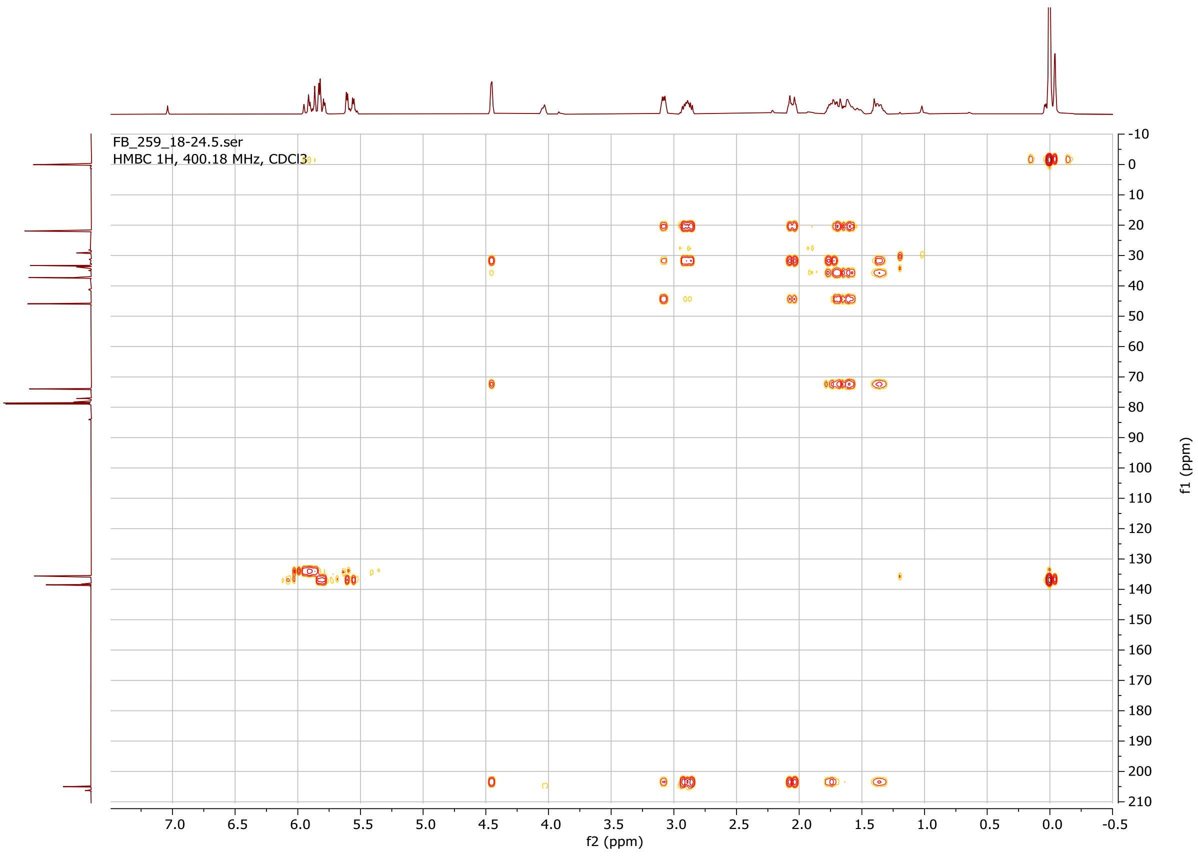
FB_259_18-24.5.ser
HMBC 1H, 400.18 MHz, CDCl3
f2 (ppm)
f1 (ppm)

Metoxydimethyl((2-vinyl-2,3-dihydro-1*H*-inden-1-yl)oxy)silane **5a**

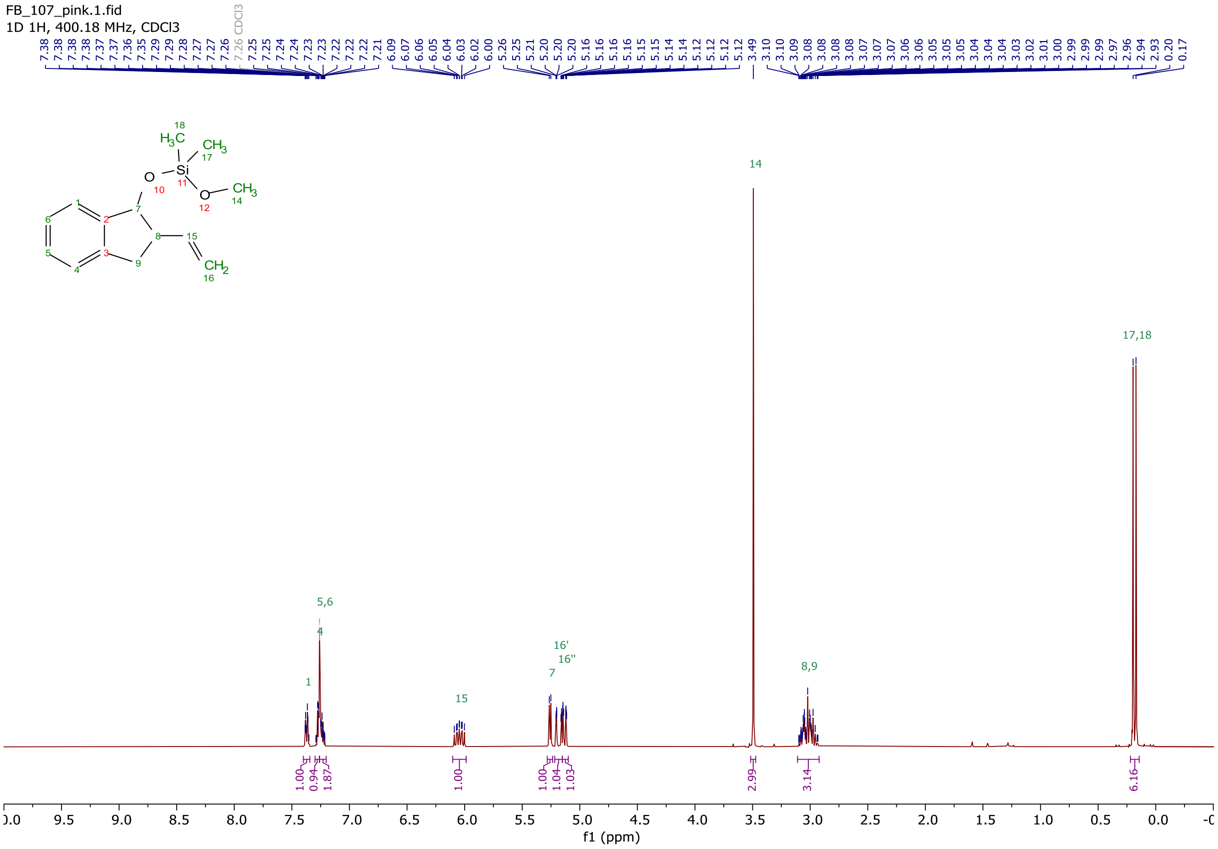

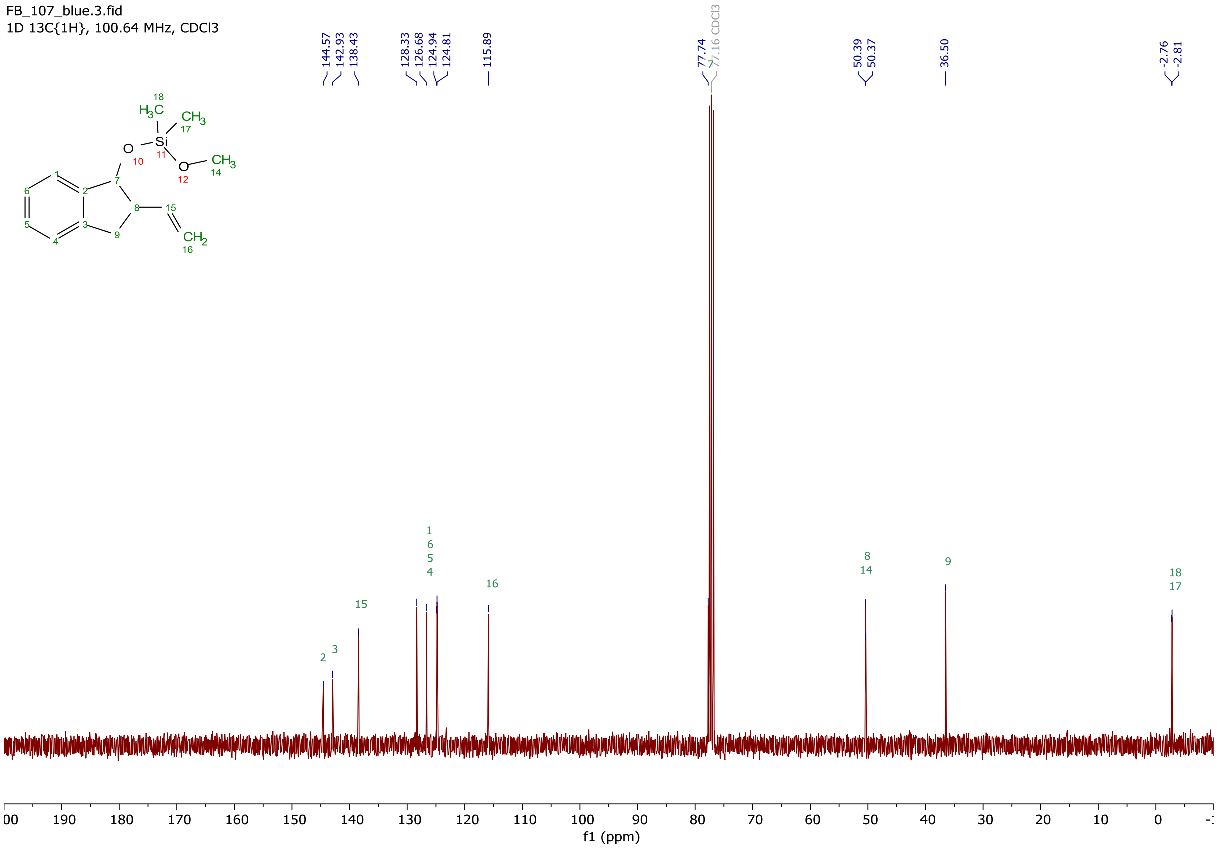

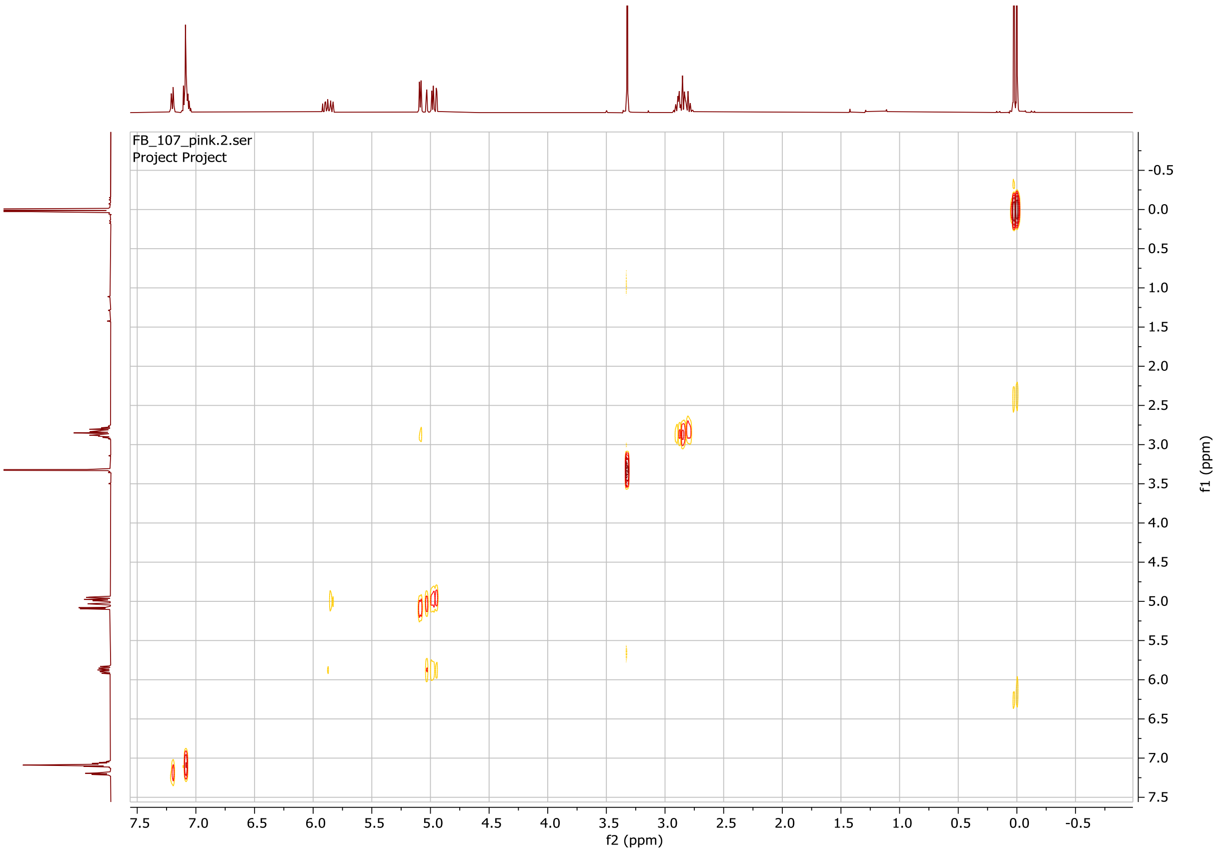
FB_107_pink.2.ser
Project Project
f2 (ppm)
f1 (ppm)

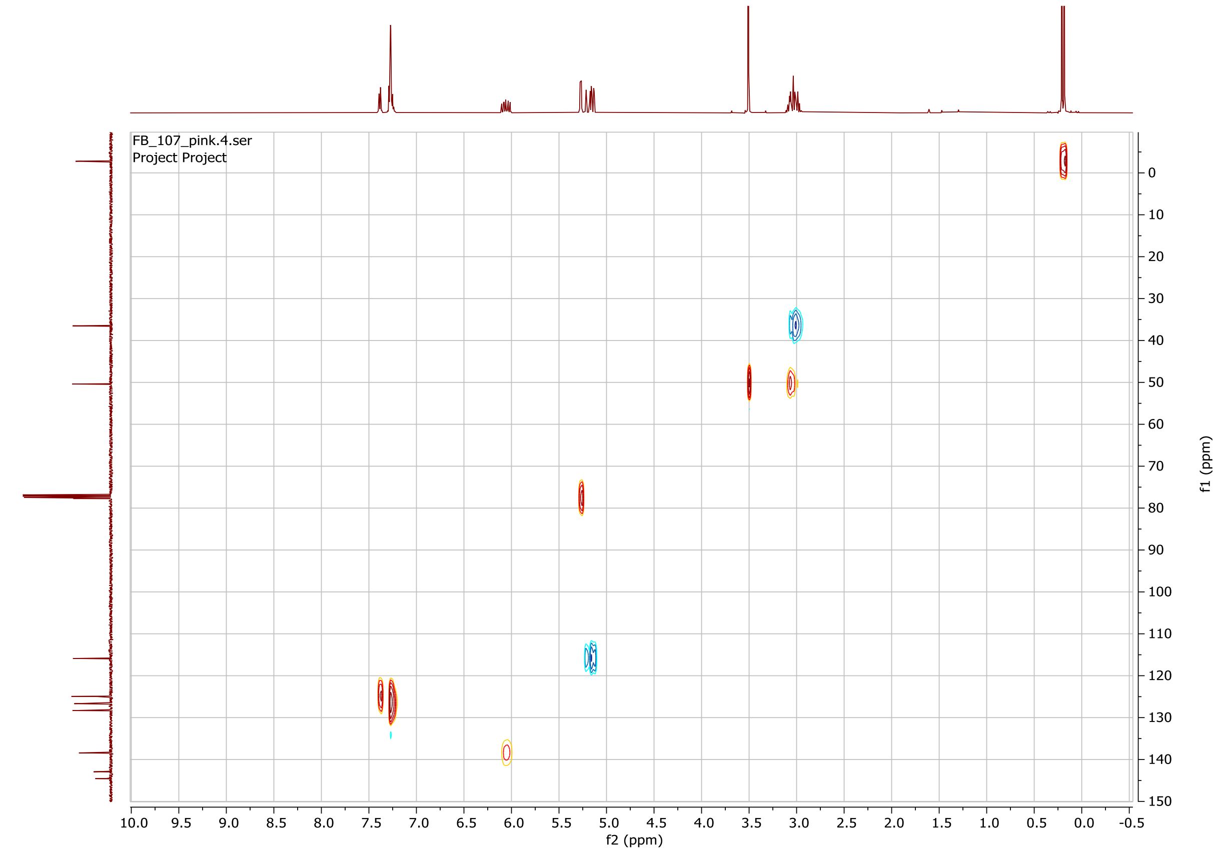
FB_107_pink.4.ser
Project Project
f2 (ppm)
f1 (ppm)

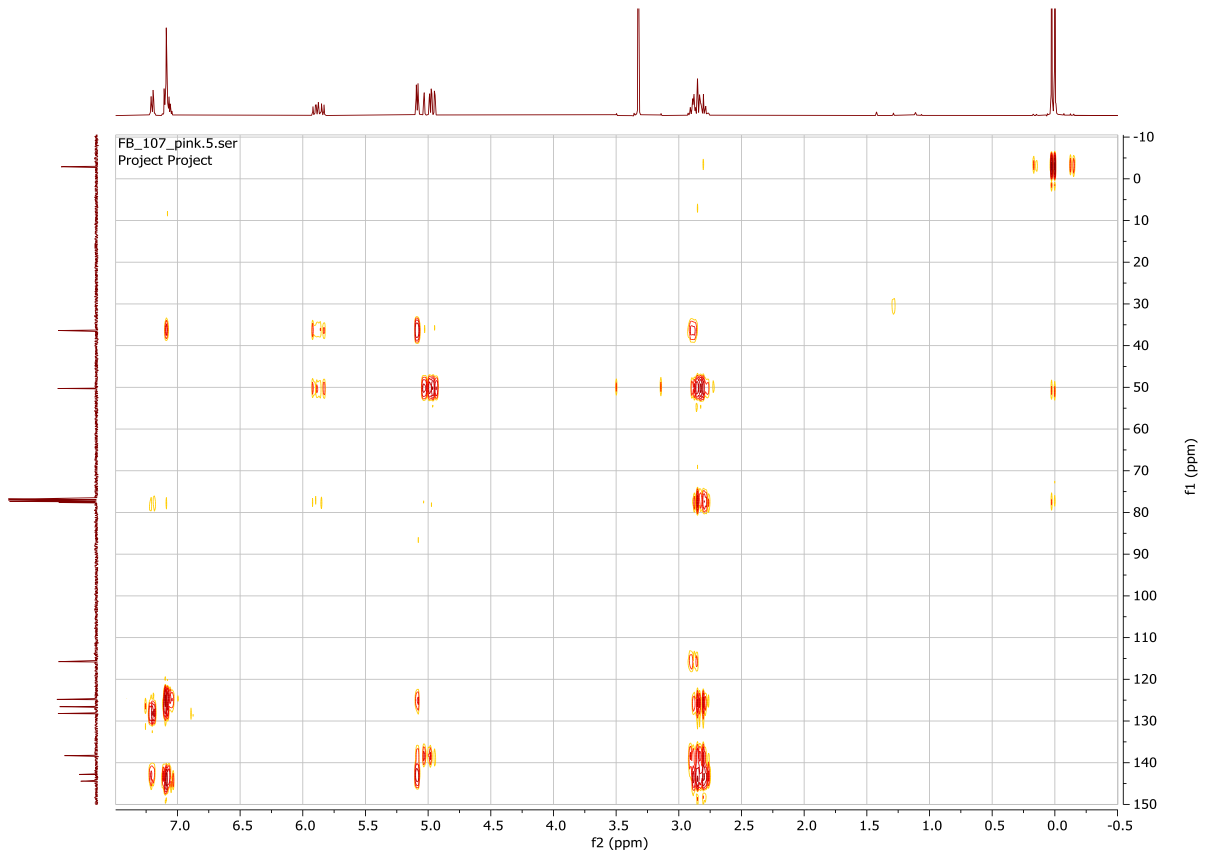

Inseparable 1:2 mixture of substrate **4k** and methoxydimethyl(oct-1-en-4-yloxy)silane **5k**

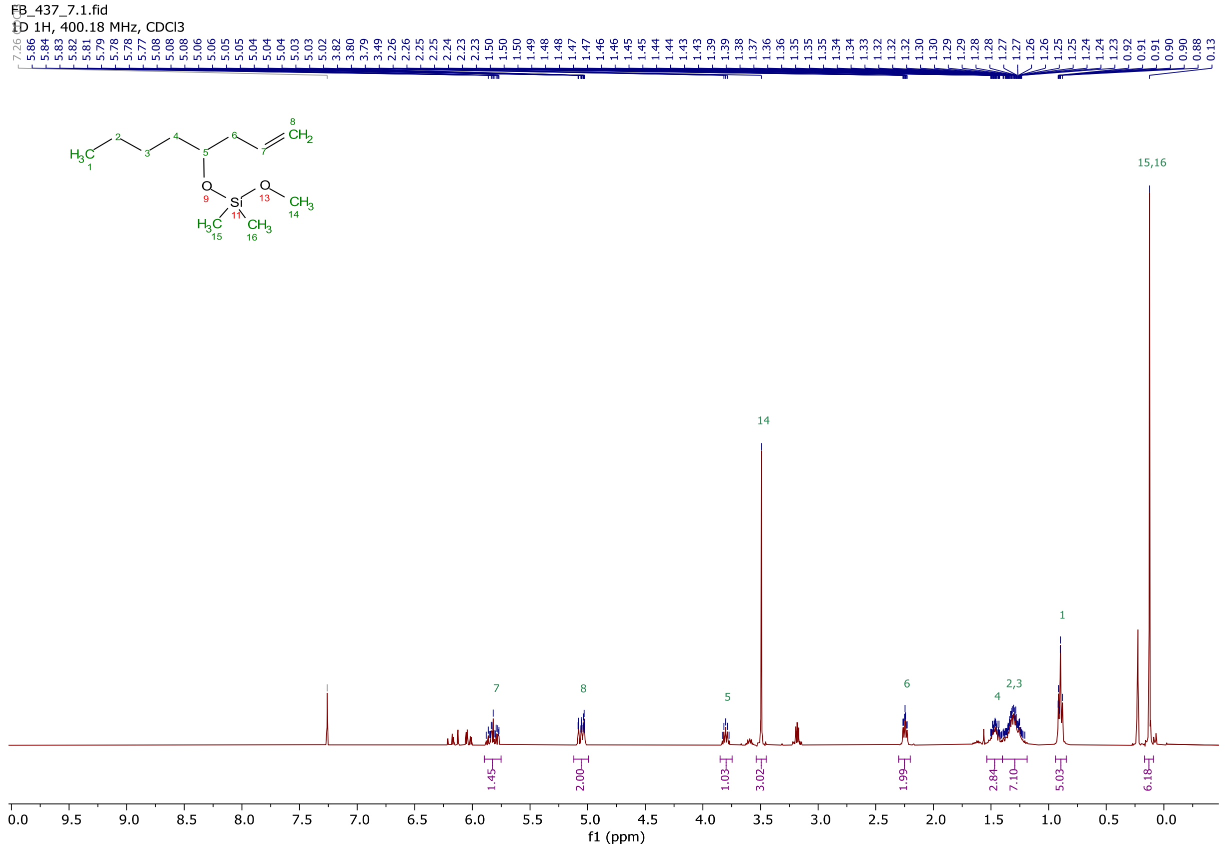

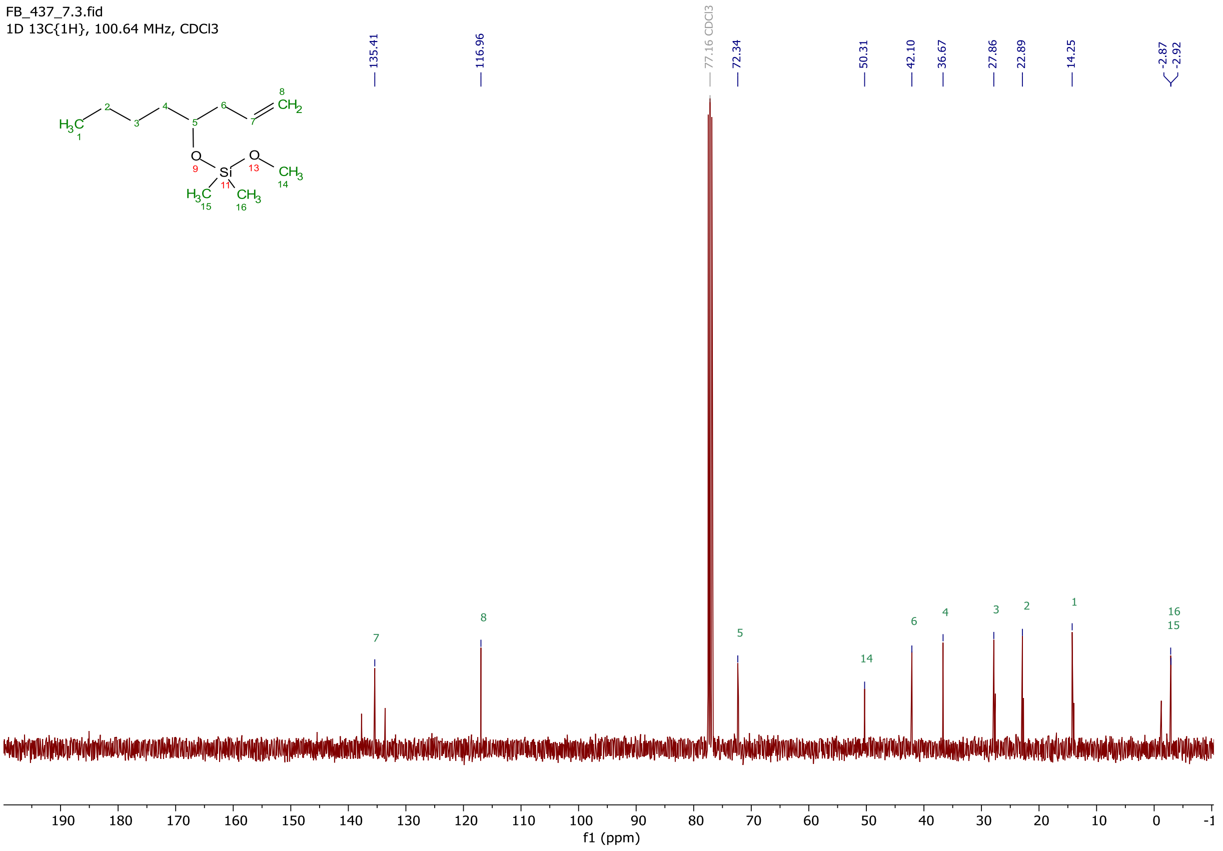

FB_437_7.3.fid
1D 13C{1H}, 100.64 MHz, CDCl3
135.41
116.96
77.16 CDCl3
72.34
50.31
42.10
36.67
27.86
22.89
14.25
-2.87
-2.92
f1 (ppm)

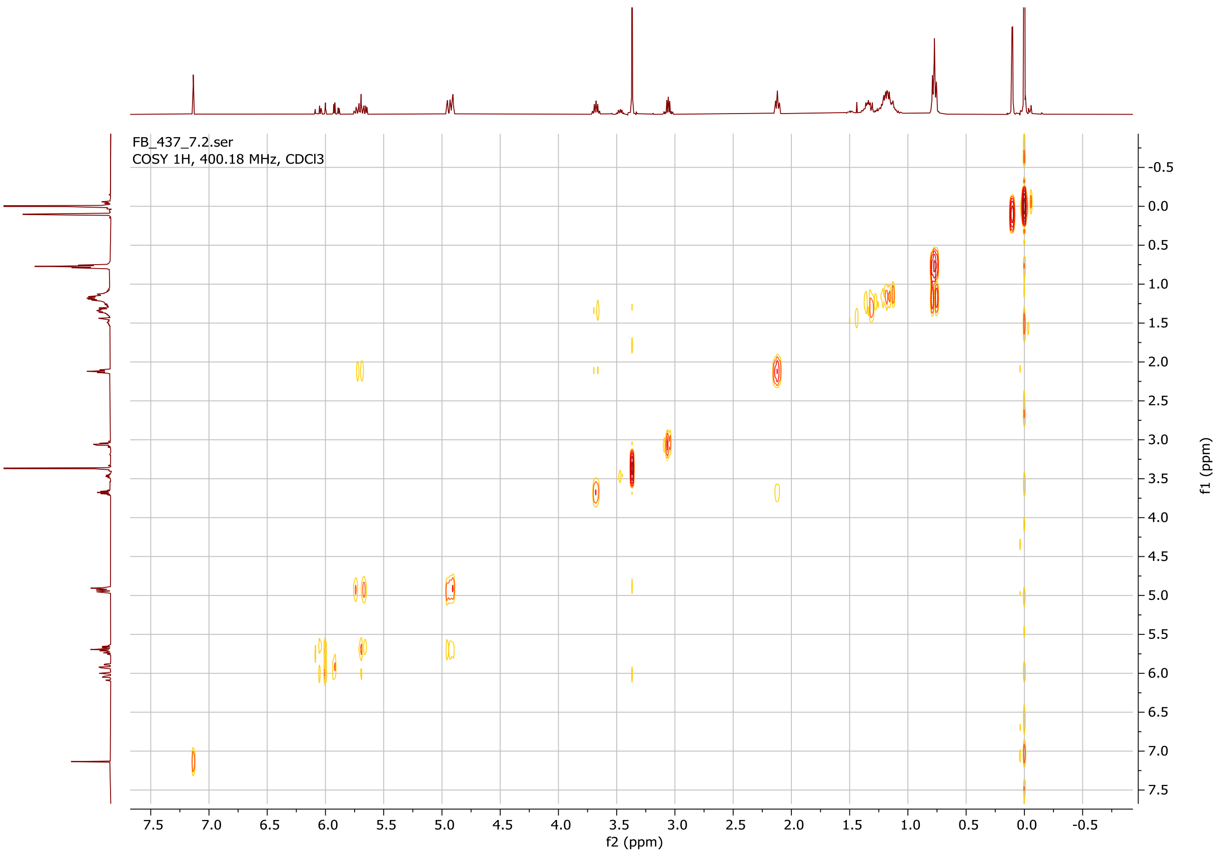

FB_437_7.2.ser
COSY 1H, 400.18 MHz, CDCl3
f2 (ppm)
f1 (ppm)

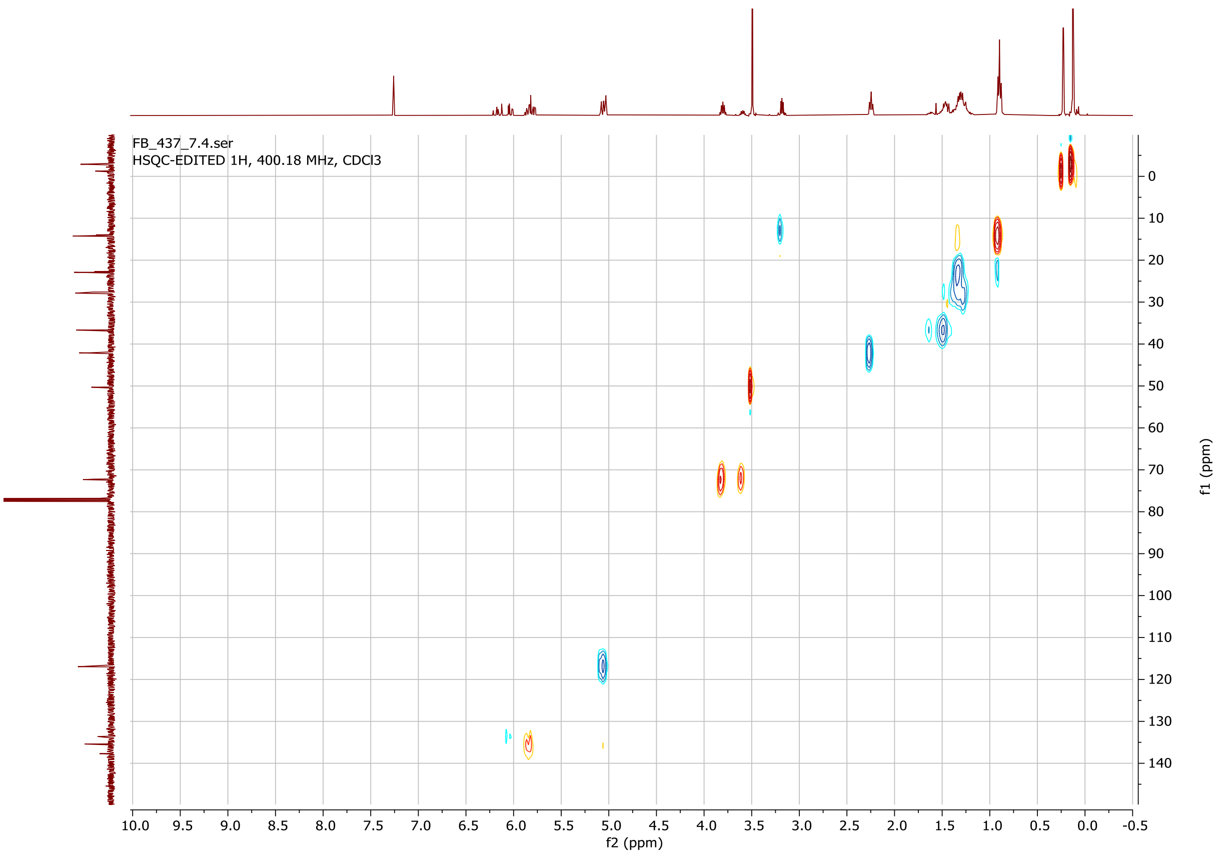
FB_437_7.4.ser
HSQC-EDITED 1H, 400.18 MHz, CDCl3
f1 (ppm)
f2 (ppm)

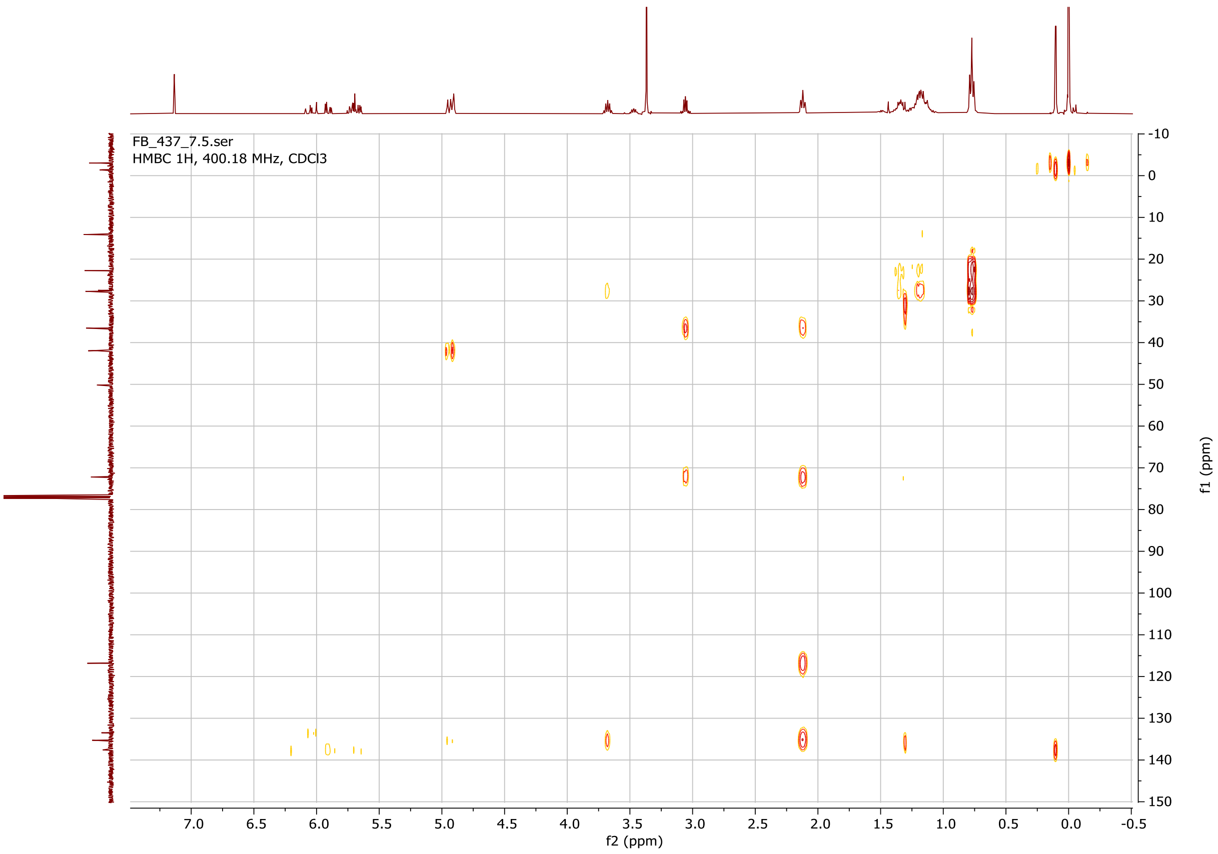
FB_437_7.5.ser
HMBC 1H, 400.18 MHz, CDCl3
f1 (ppm)
f2 (ppm)

Methoxydimethyl((1-phenylbut-3-en-1-yl)oxy)silane **5n**

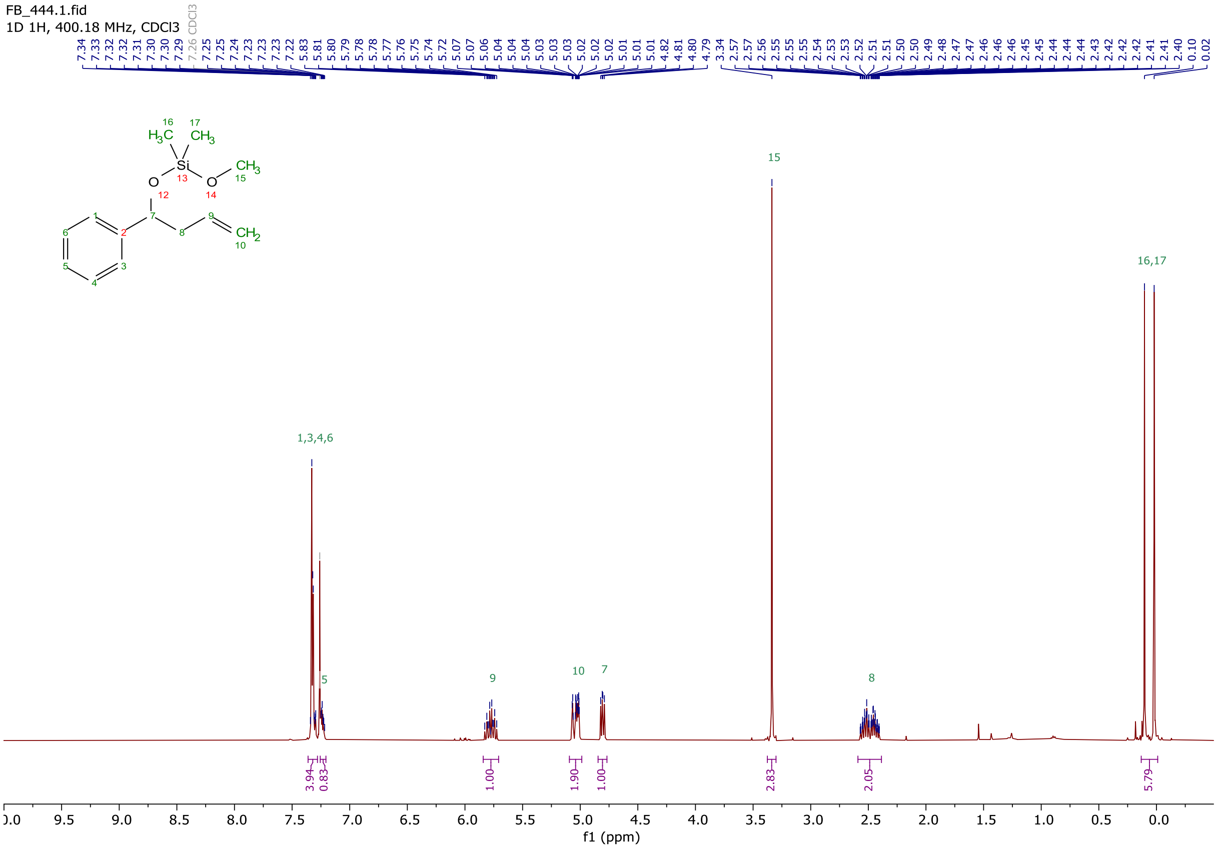

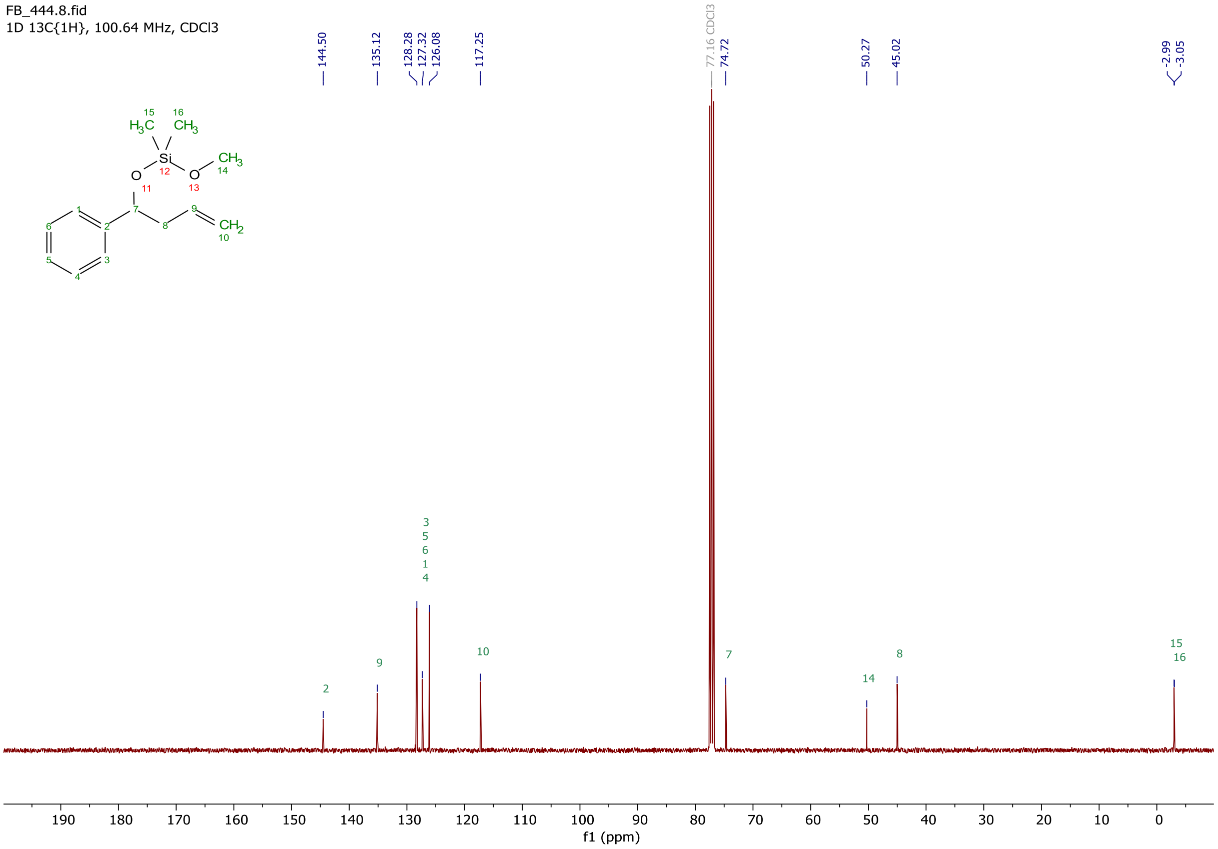

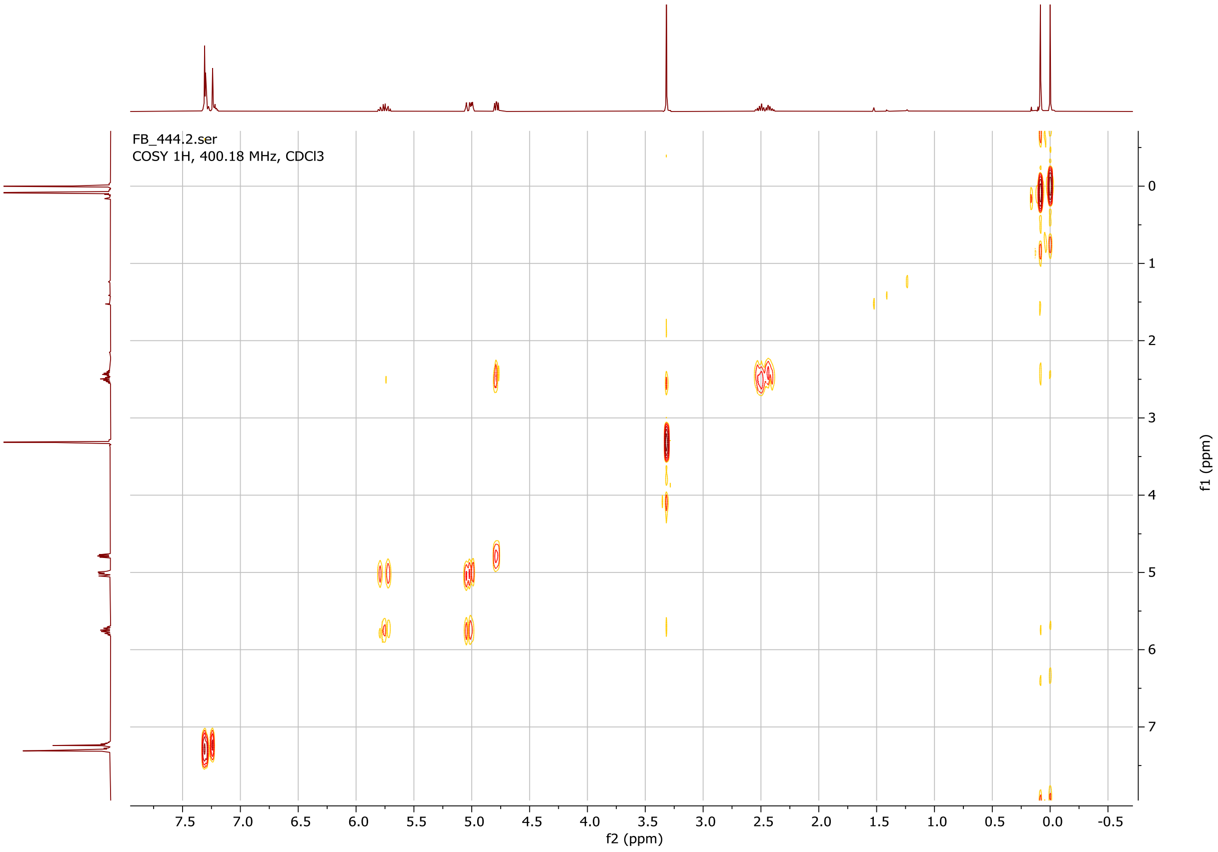
FB_444.2.ser
COSY 1H, 400.18 MHz, CDCl3
f2 (ppm)
f1 (ppm)

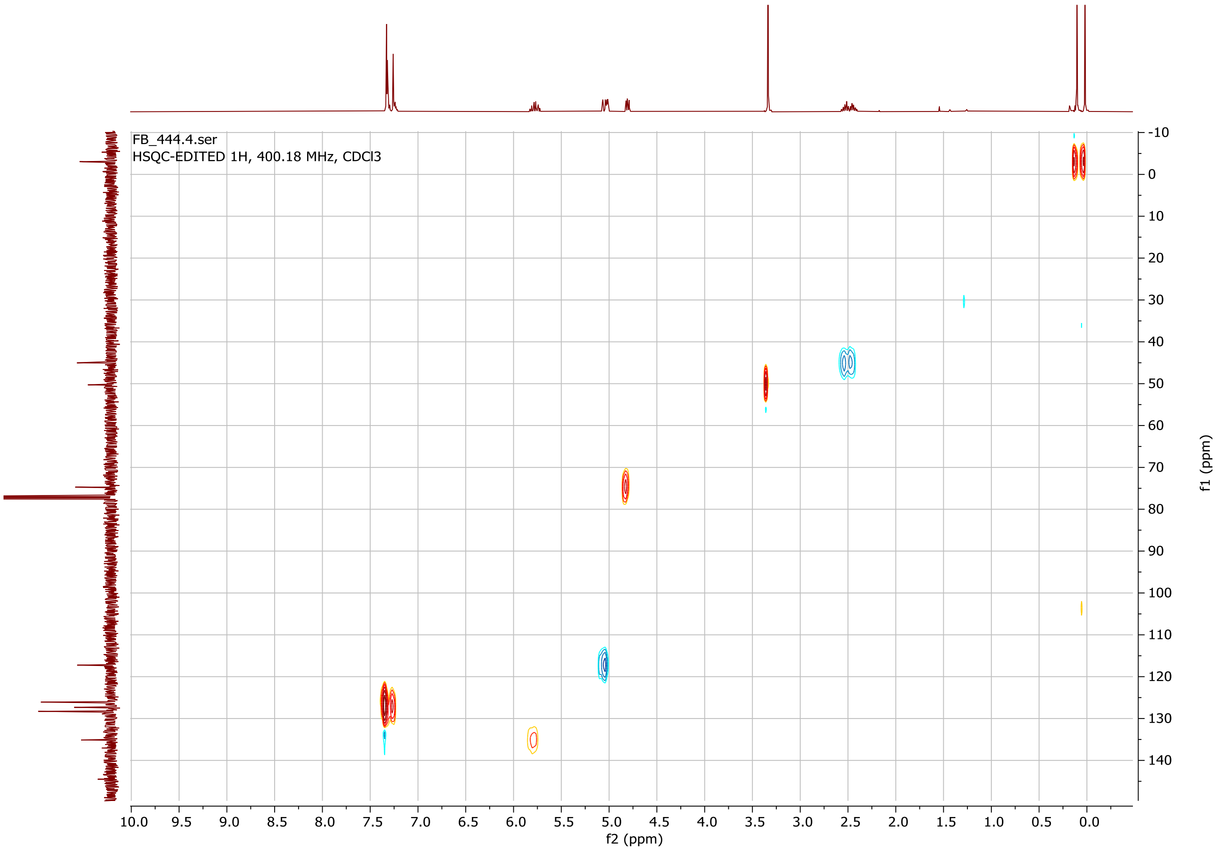
FB_444.4.ser
HSQC-EDITED 1H, 400.18 MHz, CDCl3
f2 (ppm)
f1 (ppm)

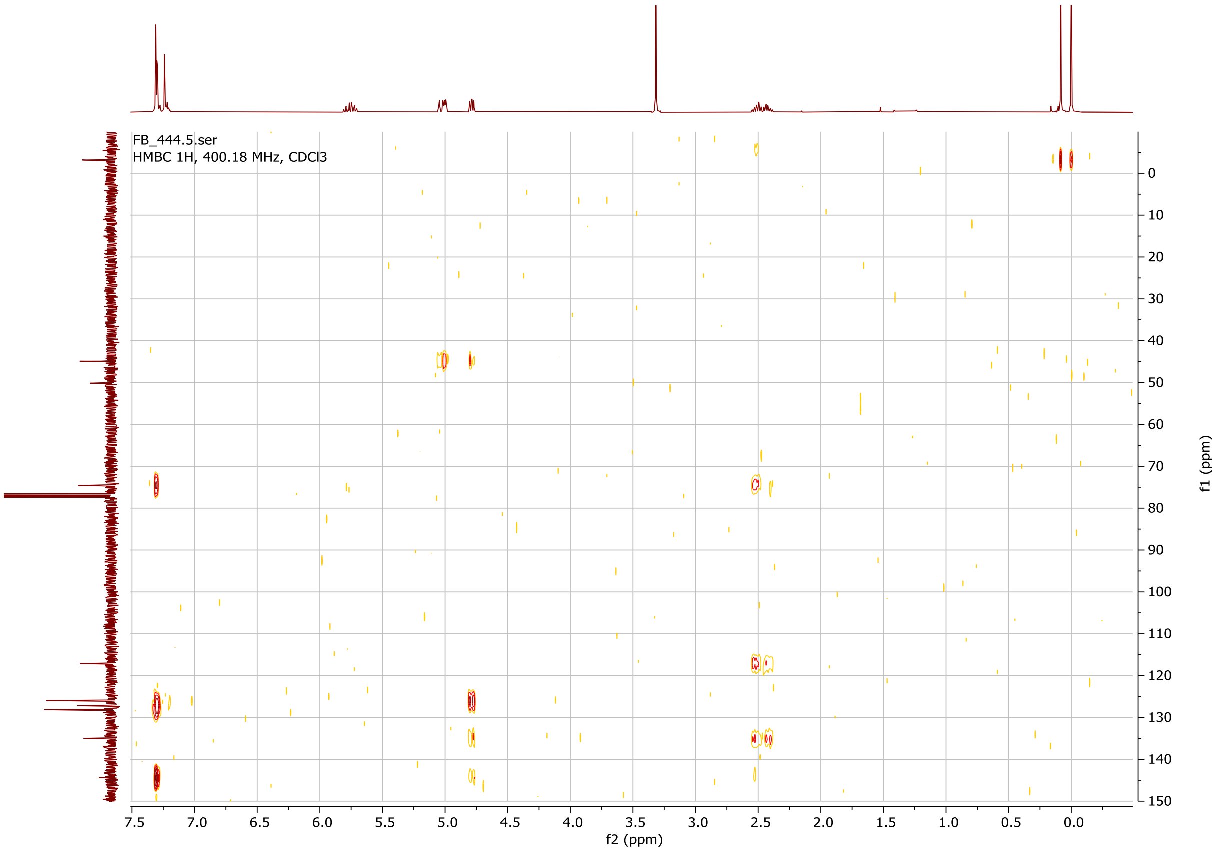

2-Vinyl-2,3-dihydro-1*H*-inden-1-ol **6a**

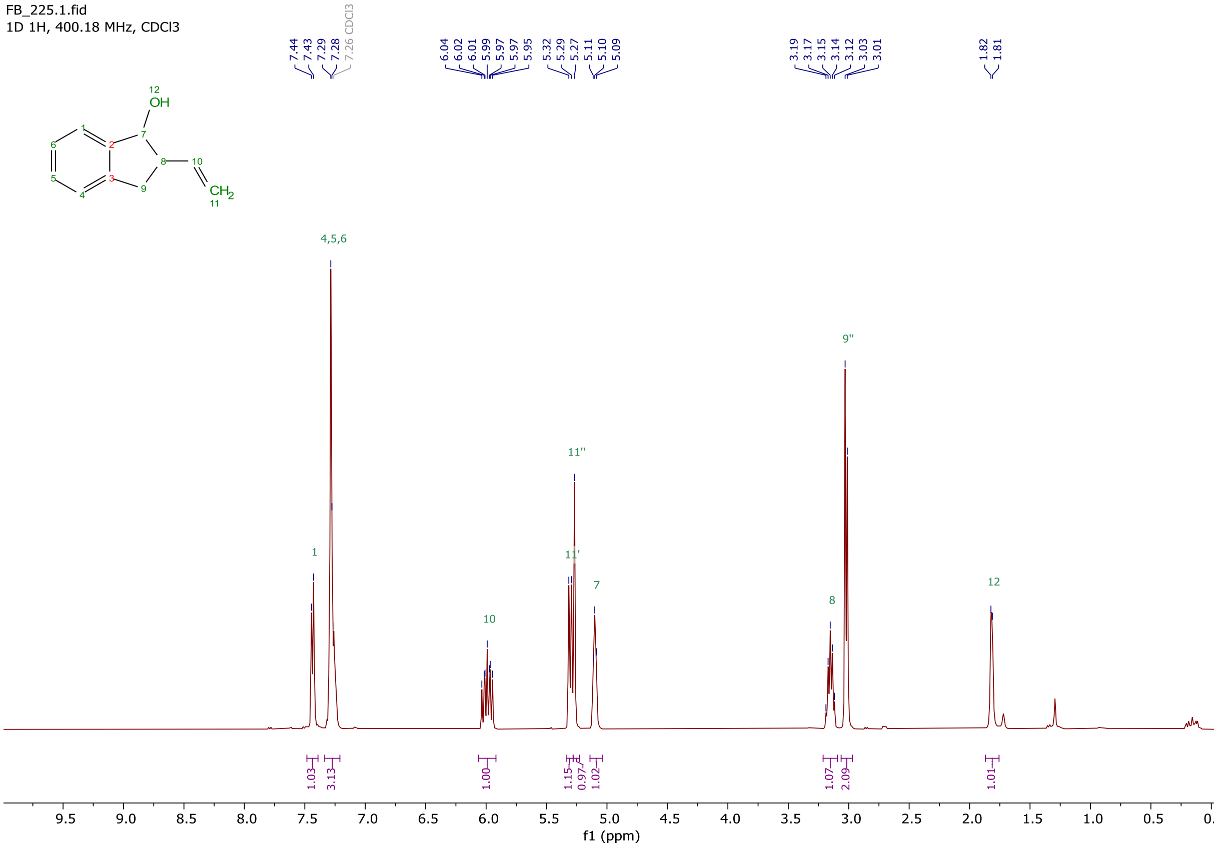

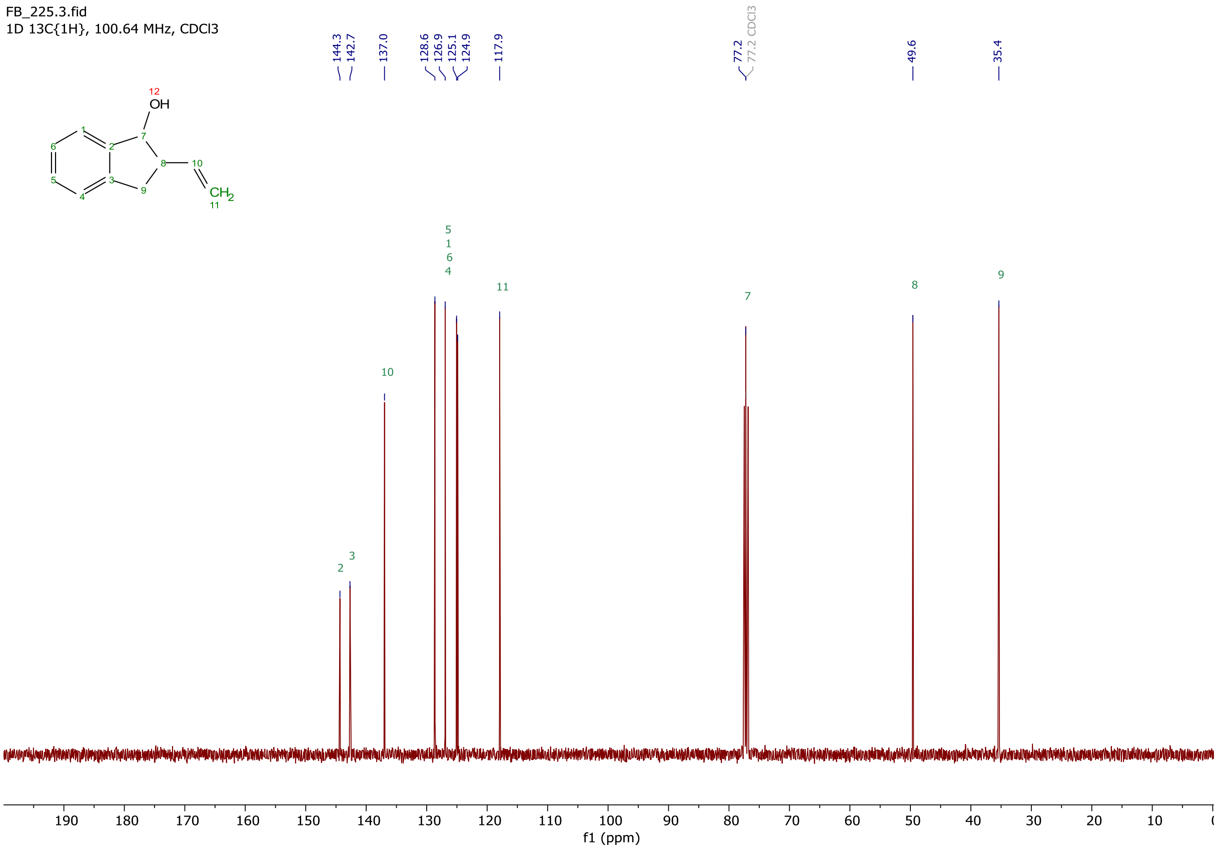
FB_225.3.fid
1D 13C{1H}, 100.64 MHz, CDCl3
144.3
142.7
137.0
128.6
126.9
125.1
124.9
117.9
77.2
77.2 CDCl3
49.6
35.4
OH
CH2
f1 (ppm)

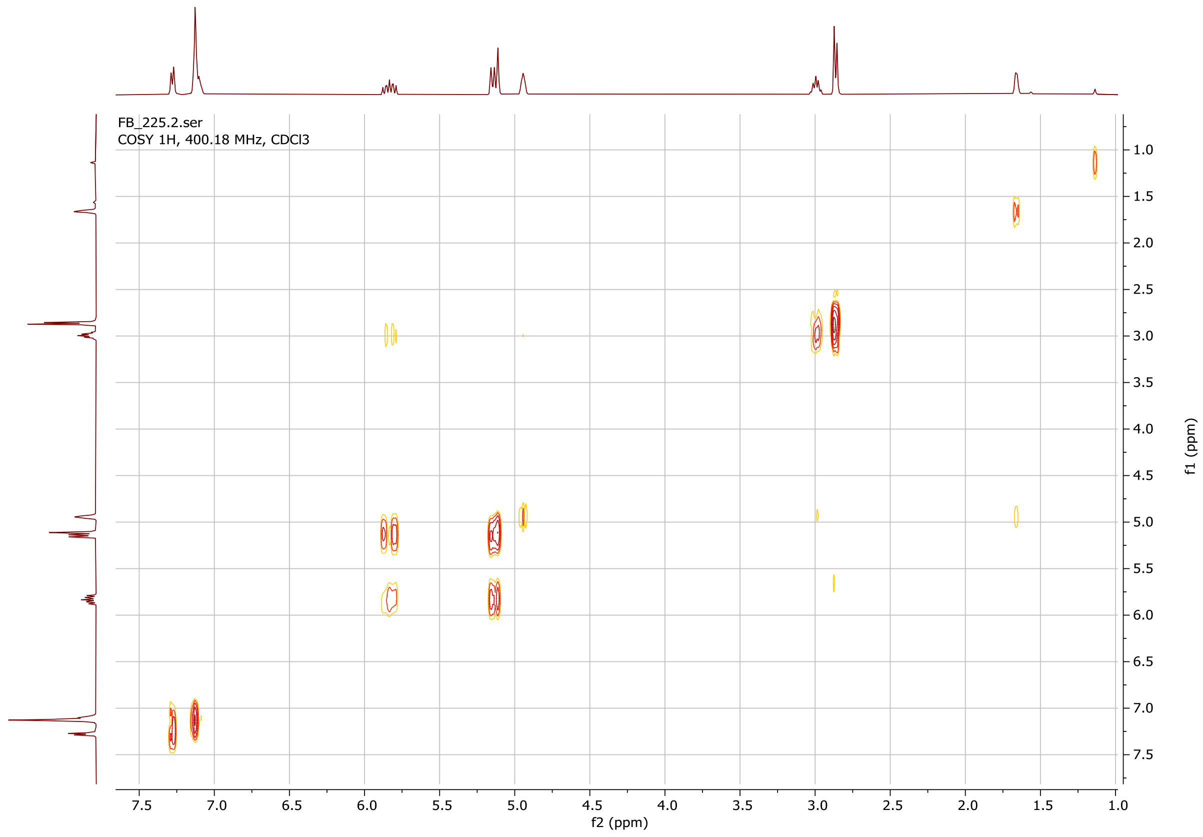
FB_225.2.ser
COSY 1H, 400.18 MHz, CDCl3
f2 (ppm)
f1 (ppm)

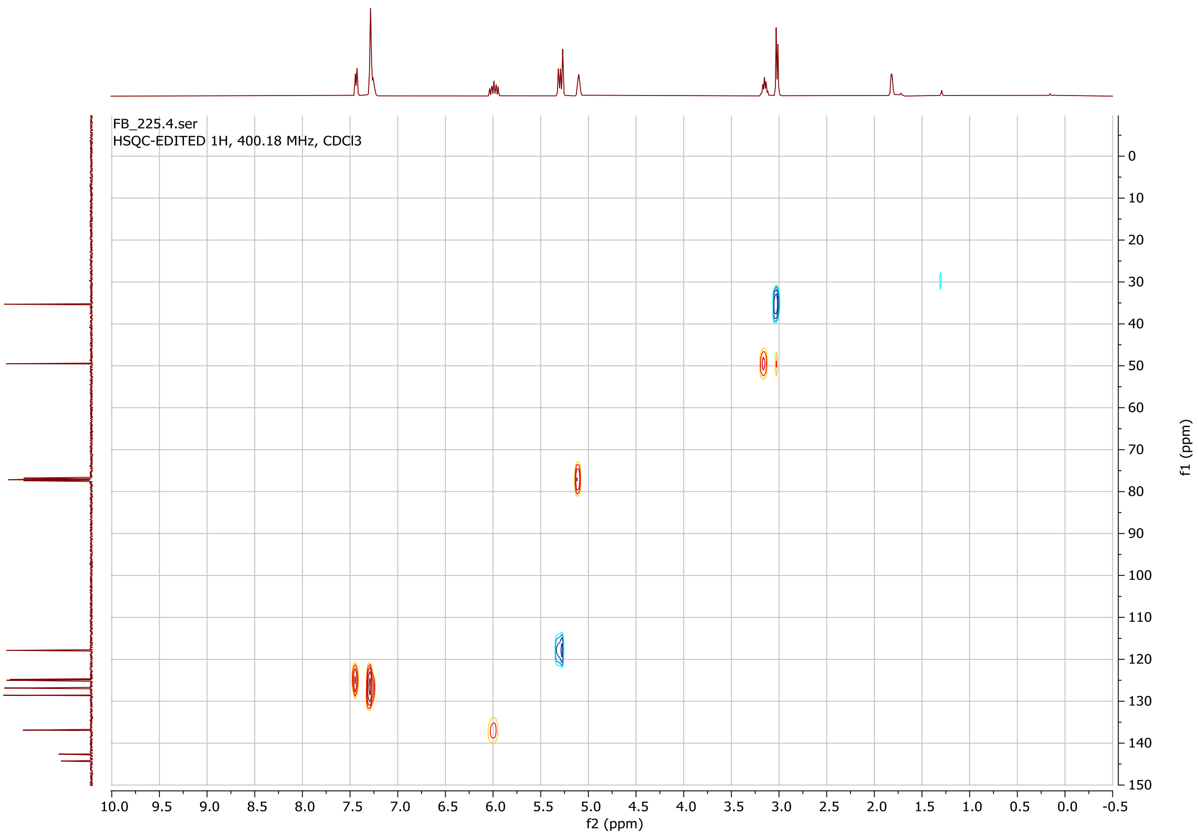

FB_225.4.ser
HSQC-EDITED 1H, 400.18 MHz, CDCl3
f2 (ppm)
f1 (ppm)

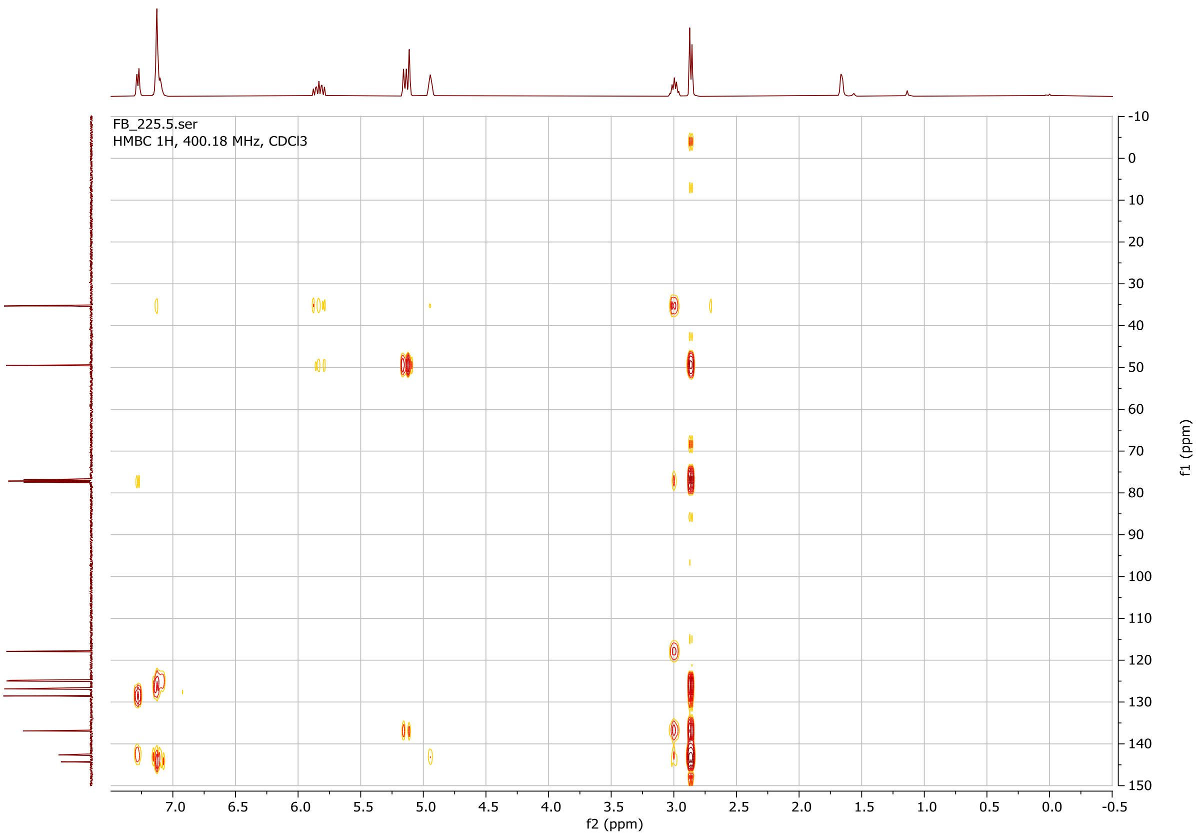

FB_225.5.ser
HMBC 1H, 400.18 MHz, CDCl3
f2 (ppm)
f1 (ppm)

2-Ethynylcyclopentanol **6b**

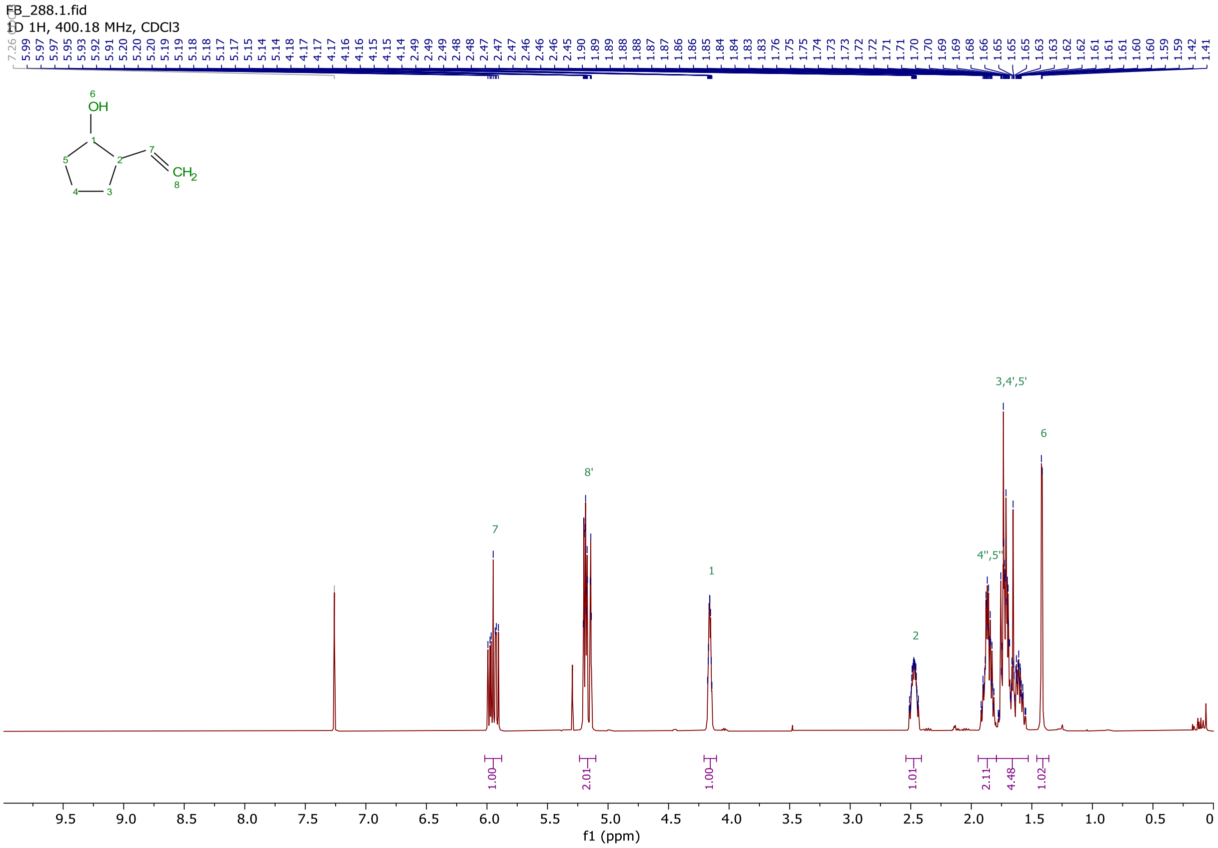

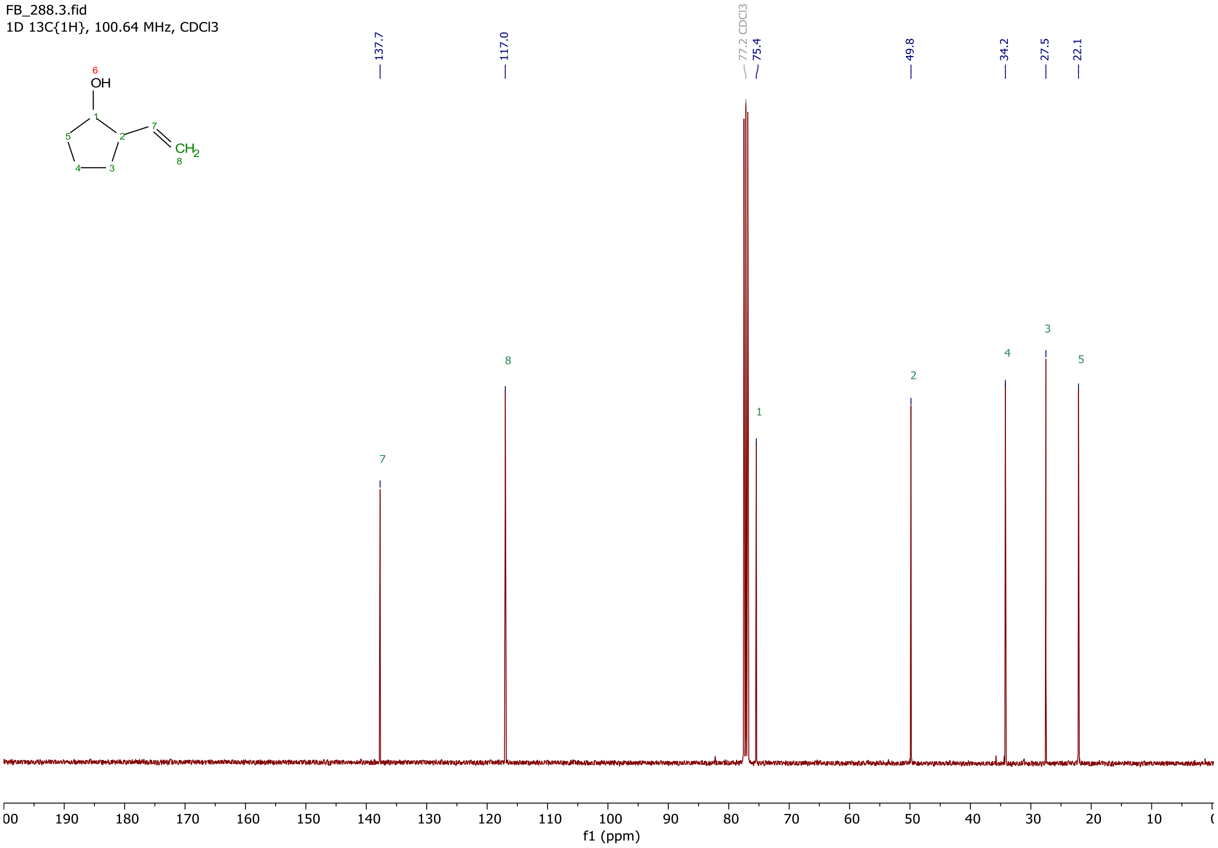

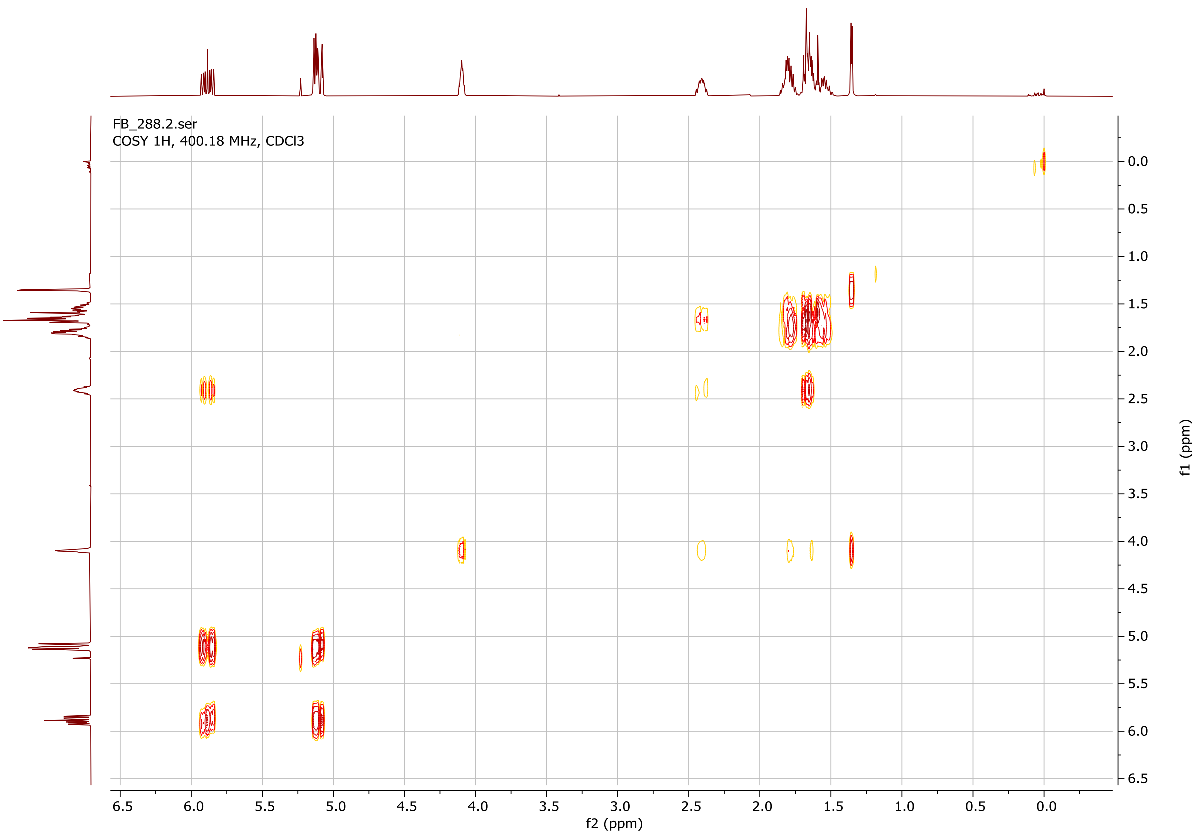
FB_288.2.ser
COSY 1H, 400.18 MHz, CDCl3
f2 (ppm)
f1 (ppm)

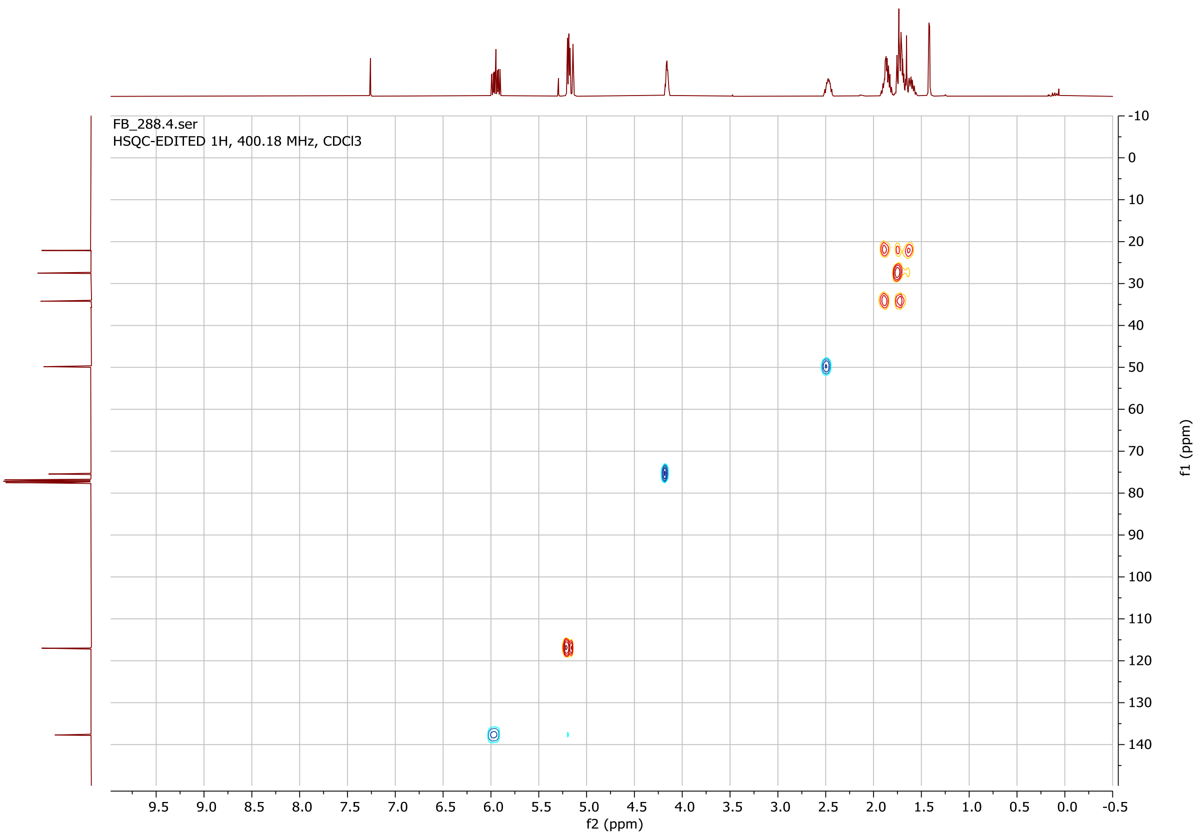
FB_288.4.ser
HSQC-EDITED 1H, 400.18 MHz, CDCl3
f2 (ppm)
f1 (ppm)

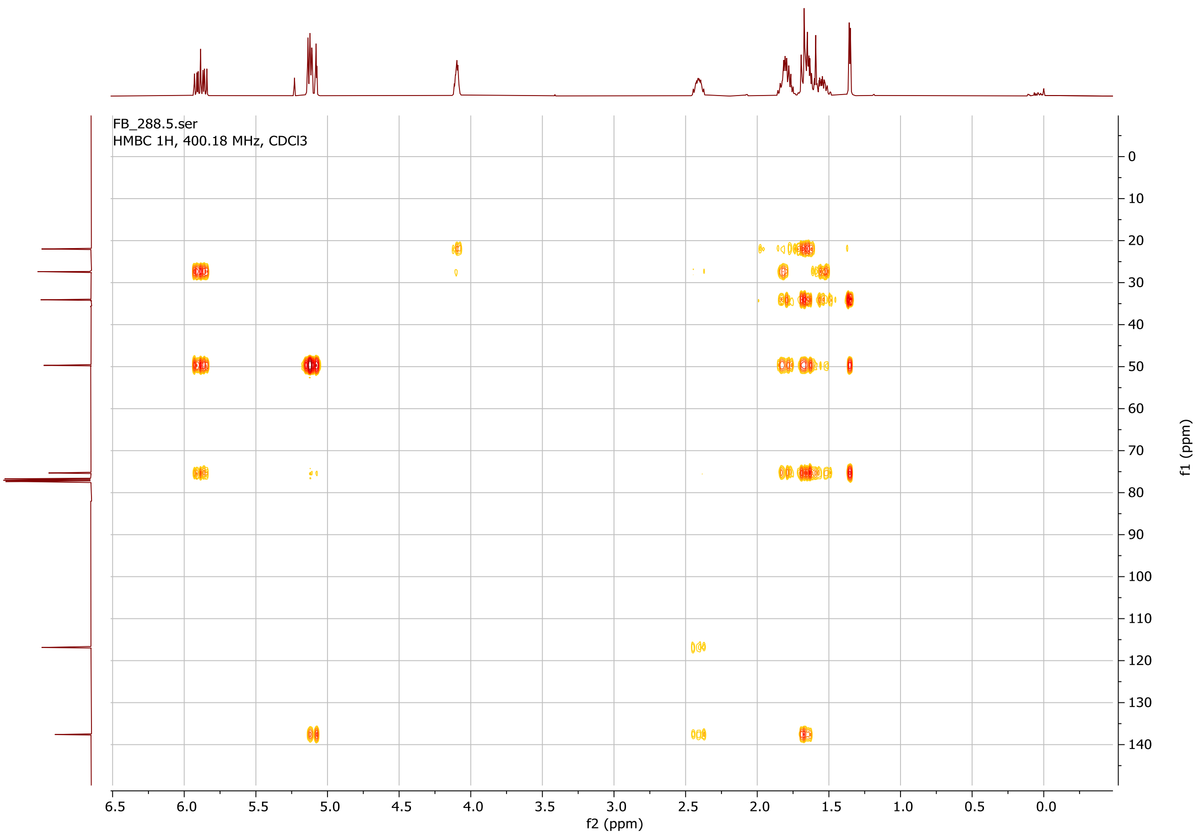

2-Vinylcyclohexan-1-ol **6c**

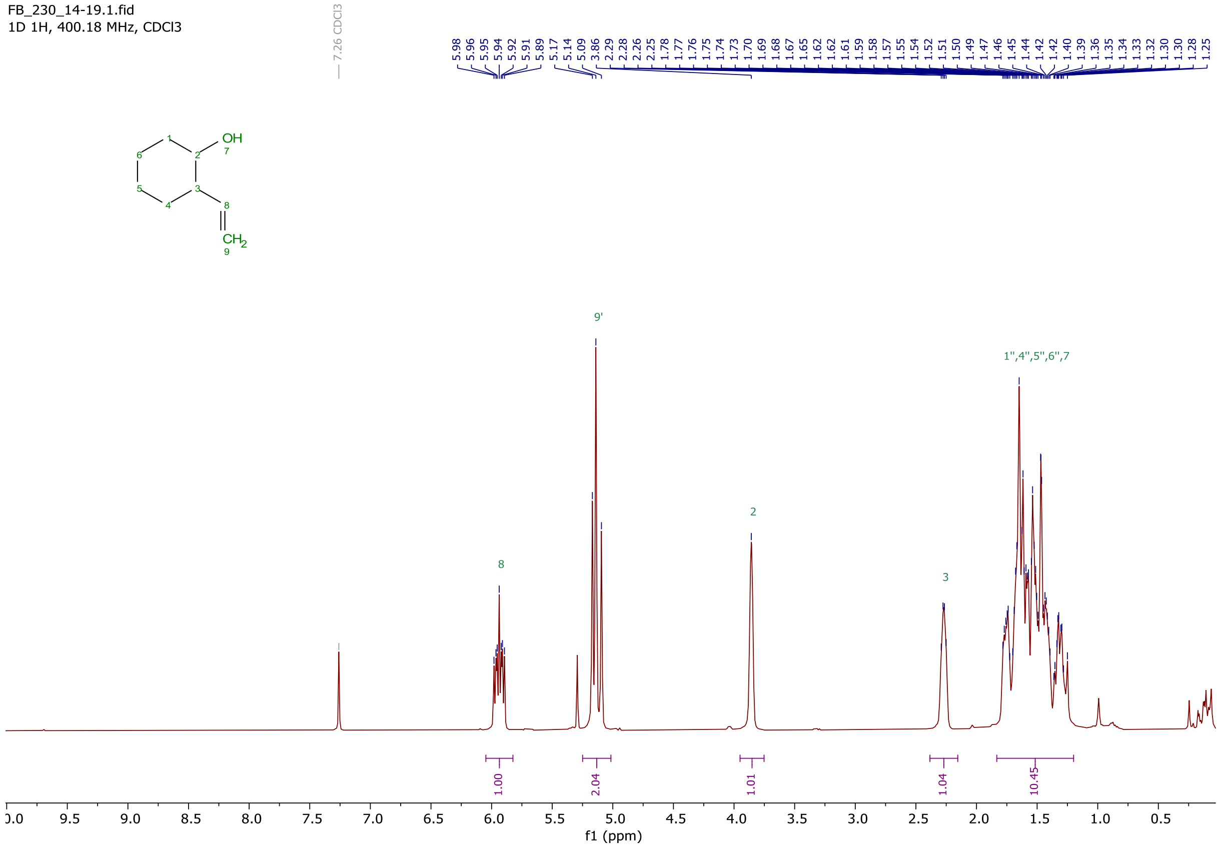

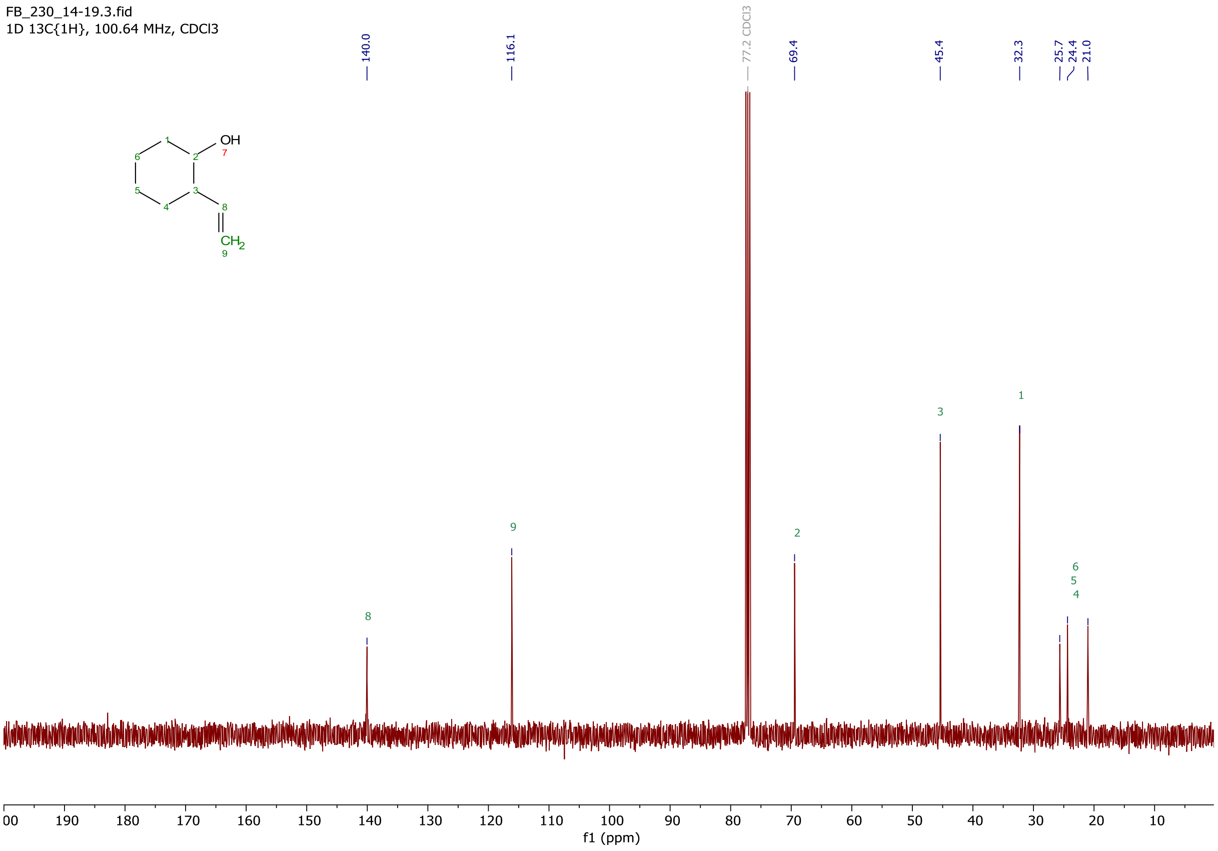
FB_230_14-19.3.fid
1D 13C{1H}, 100.64 MHz, CDCl3
140.0
116.1
77.2 CDCl3
69.4
45.4
32.3
25.7
24.4
21.0
OH
CH2
f1 (ppm)

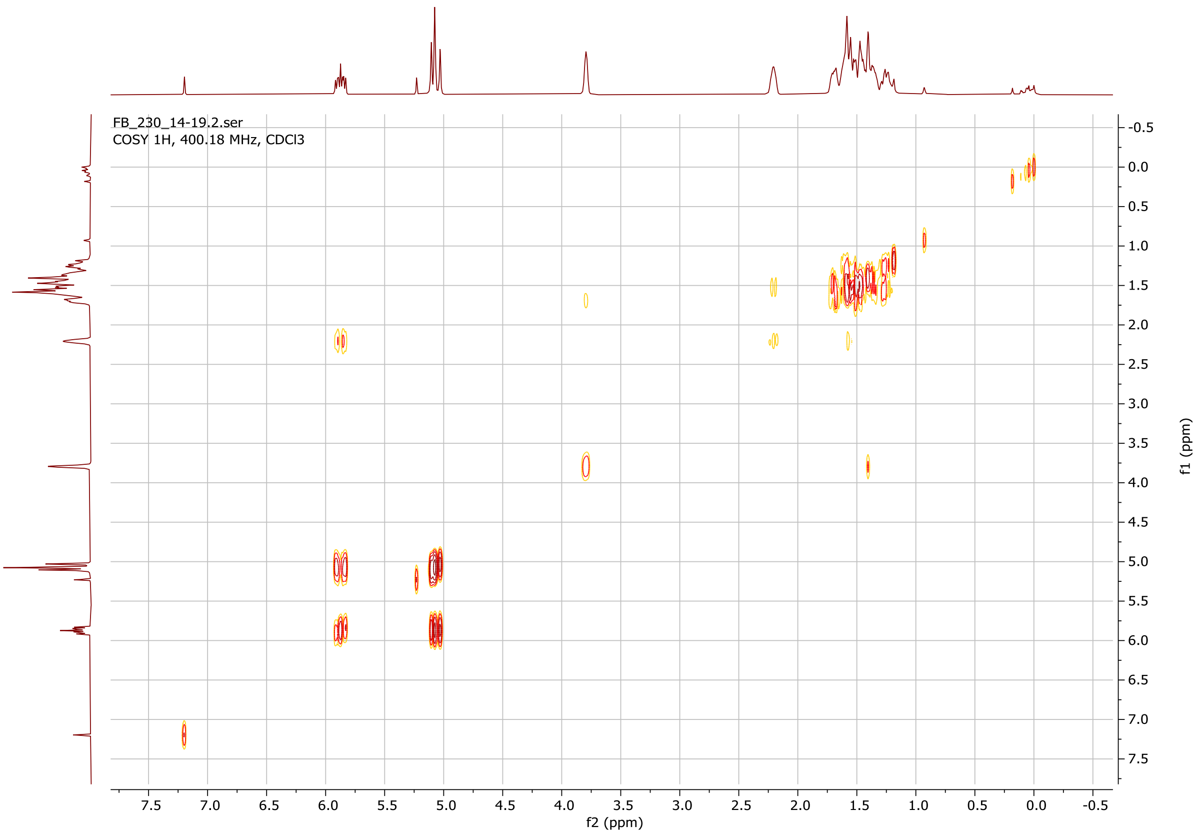
FB_230_14-19.2.ser
COSY 1H, 400.18 MHz, CDCl3
f2 (ppm)
f1 (ppm)

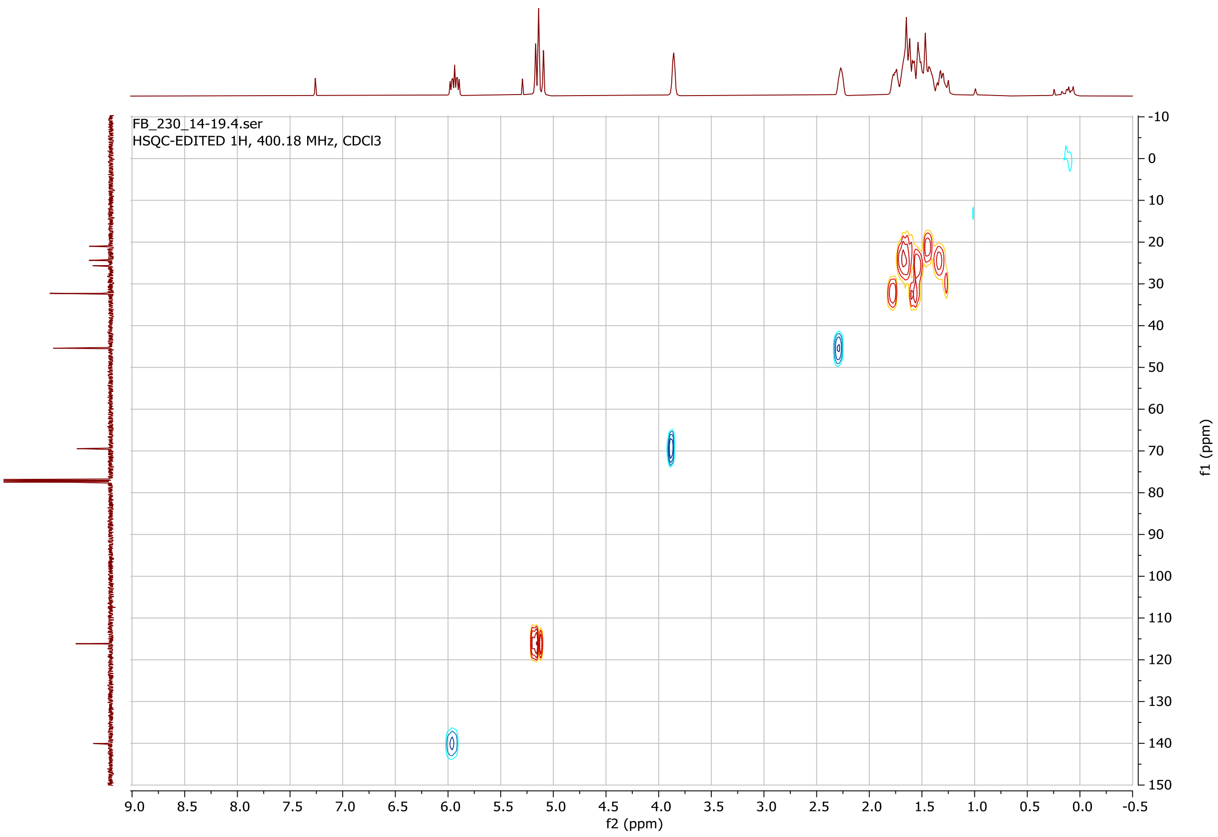
FB_230_14-19.4.ser
HSQC-EDITED 1H, 400.18 MHz, CDCl3
f2 (ppm)
f1 (ppm)

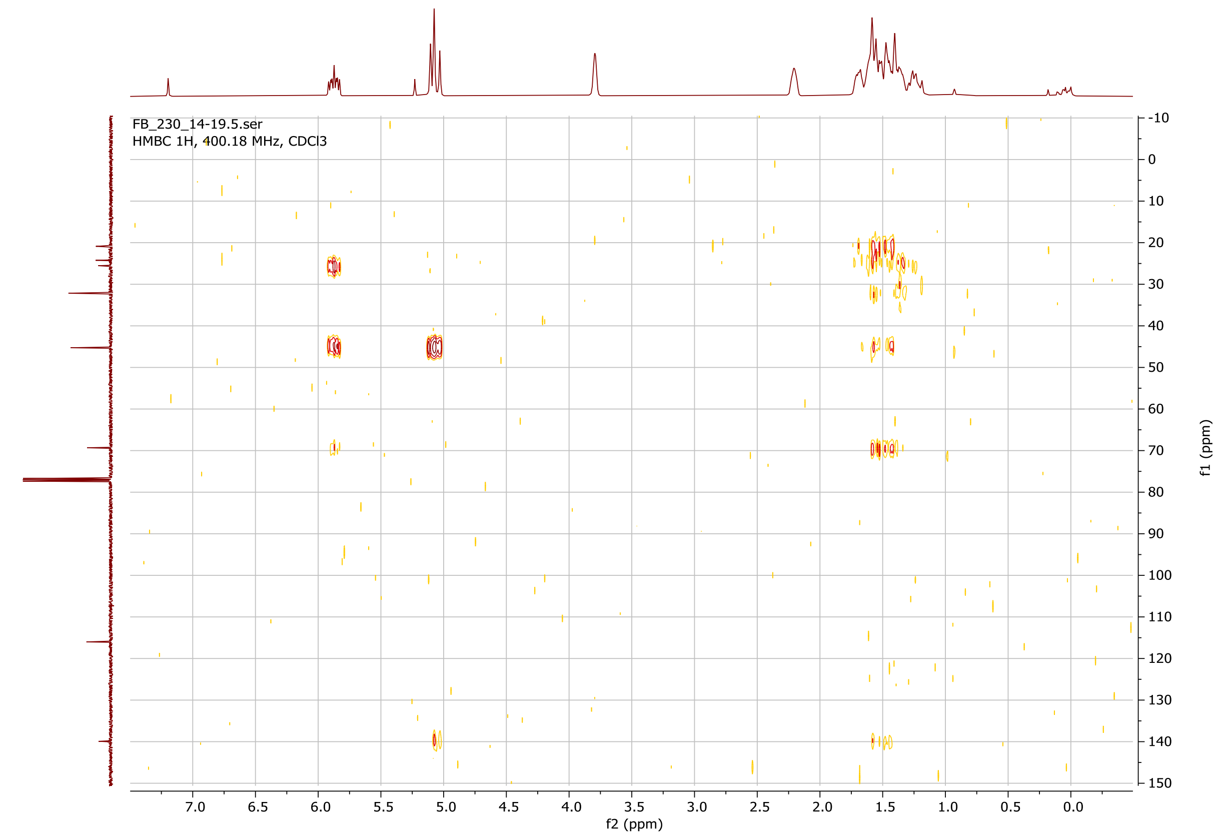
FB_230_14-19.5.ser
HMBC 1H, 400.18 MHz, CDCl3
f2 (ppm)
f1 (ppm)

tert-Butyl 3-hydroxy-4-vinylpyrrolidine-1-carboxylate **6d** *(mixture of rotamers)*

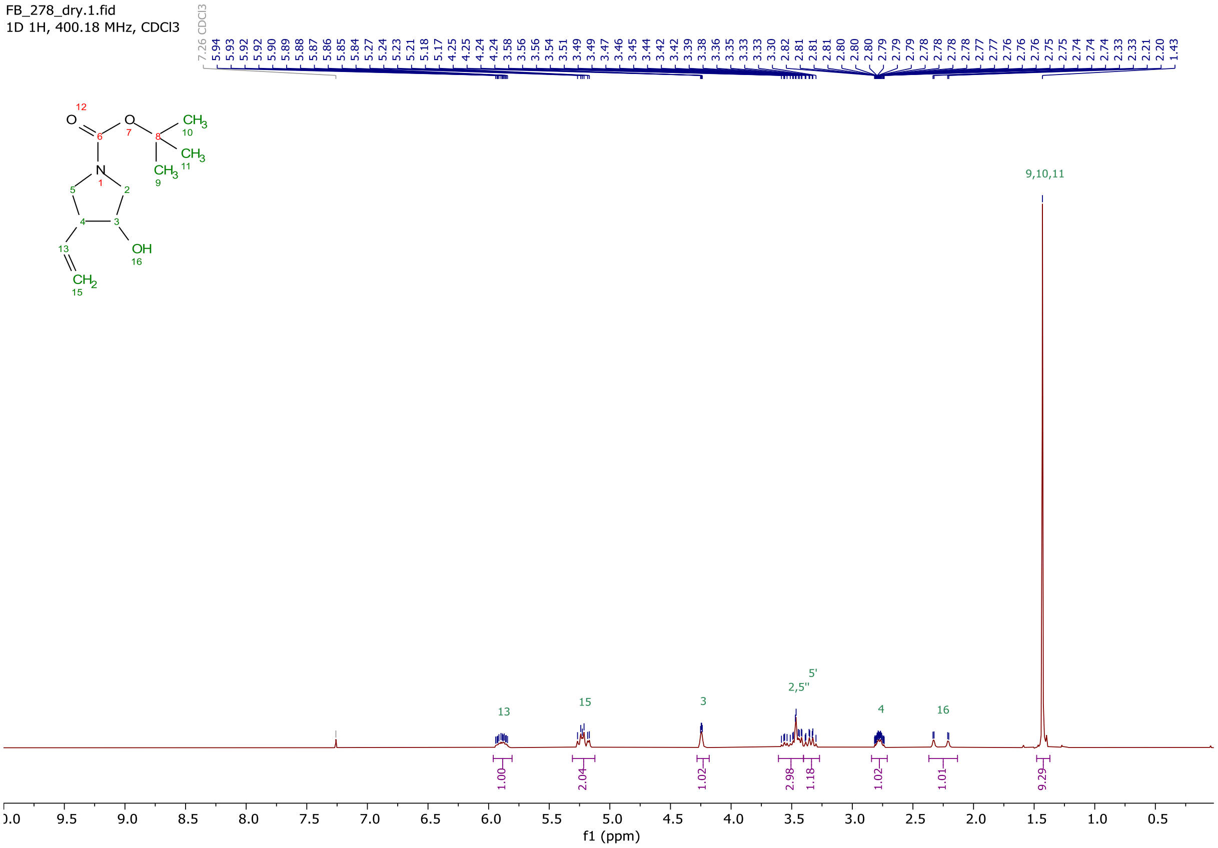

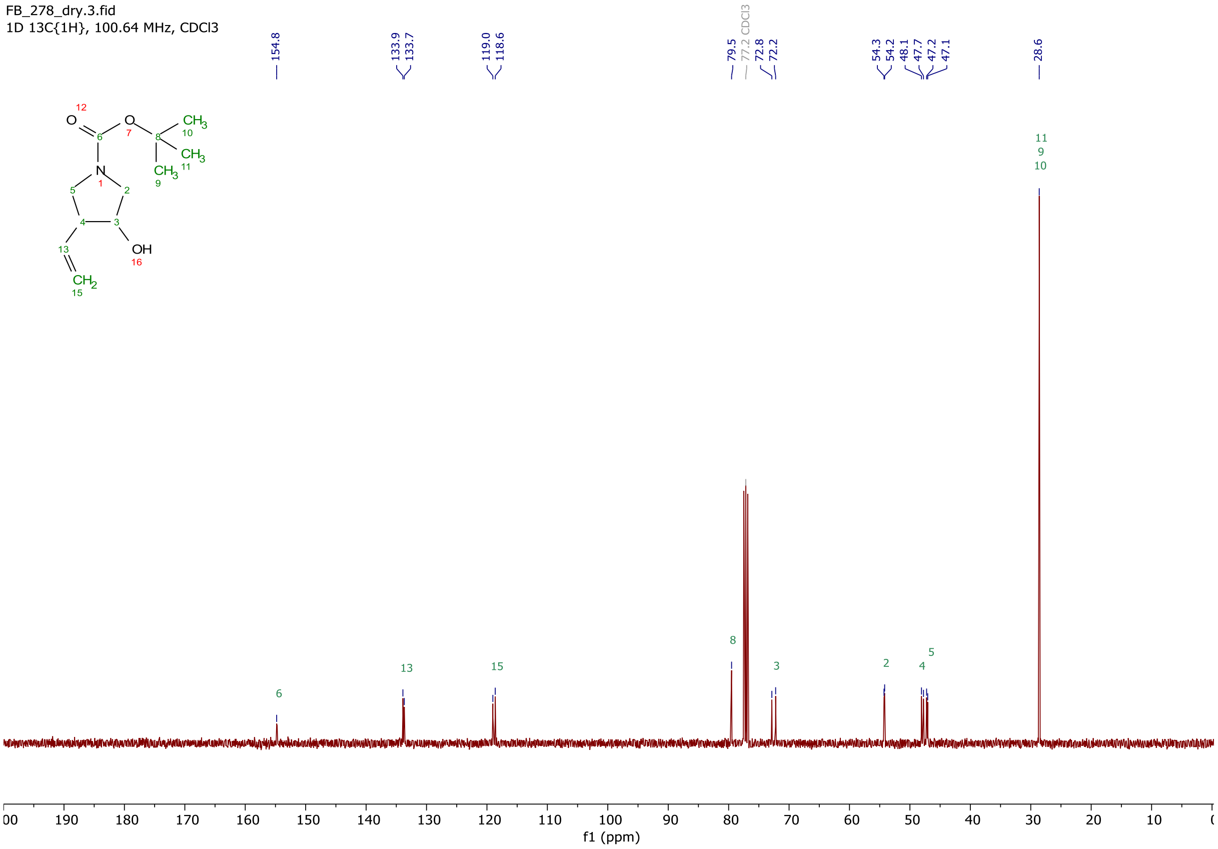

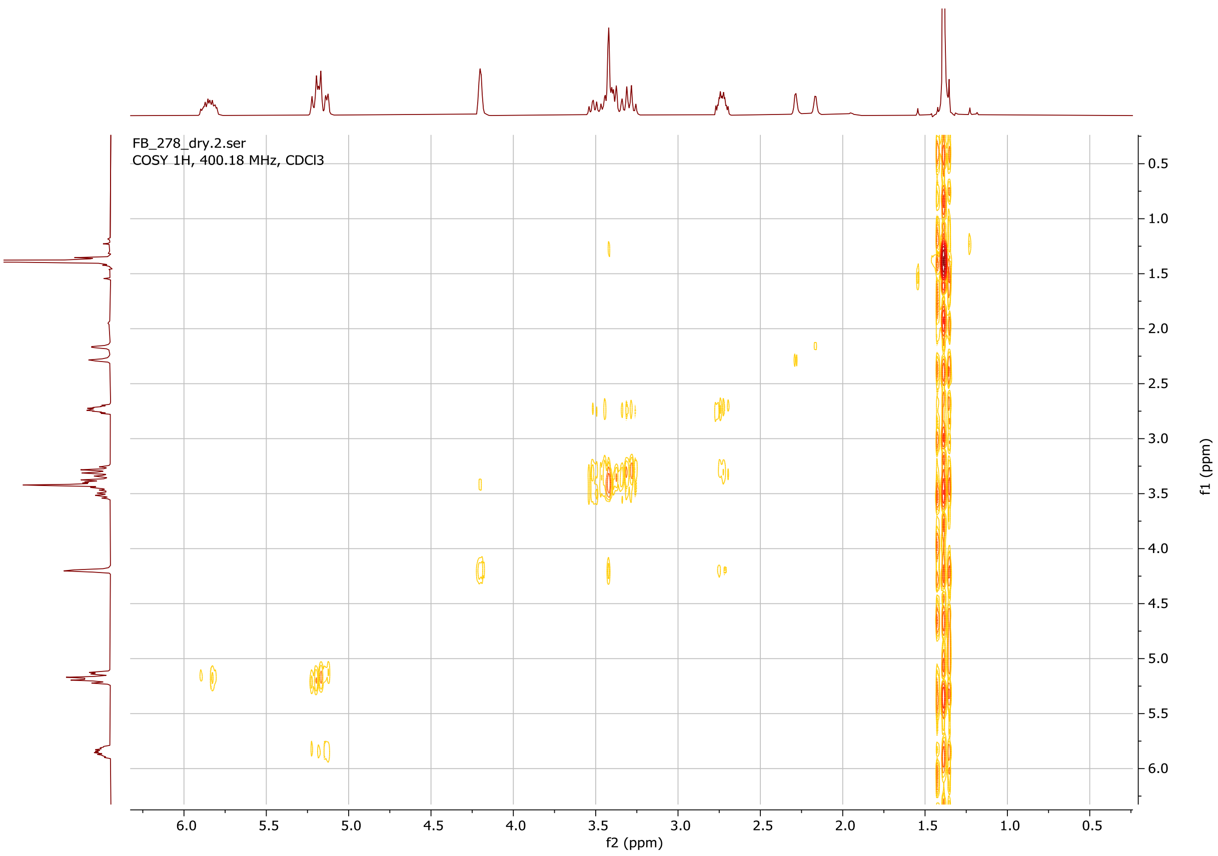
FB_278_dry.2.ser
COSY 1H, 400.18 MHz, CDCl3
f2 (ppm)
f1 (ppm)

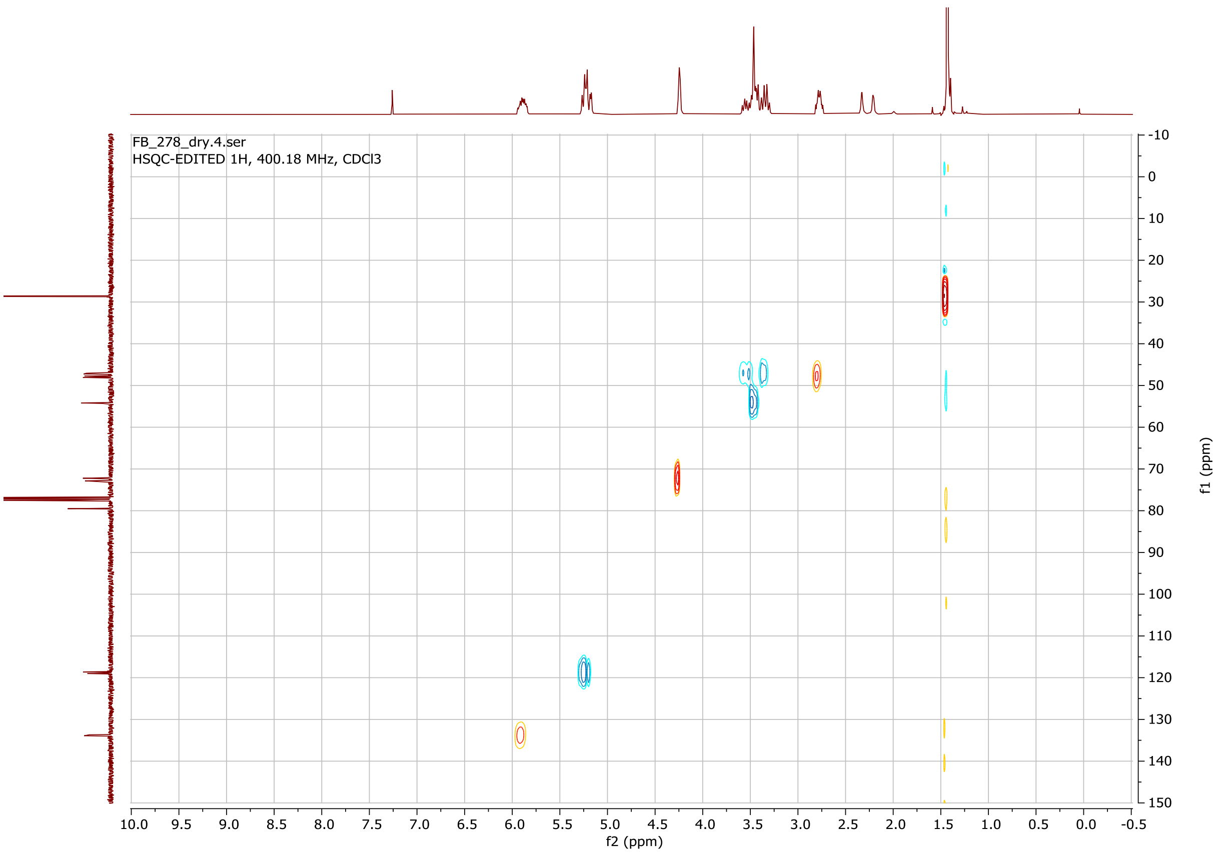
FB_278_dry.4.ser
HSQC-EDITED 1H, 400.18 MHz, CDCl3
f2 (ppm)
f1 (ppm)

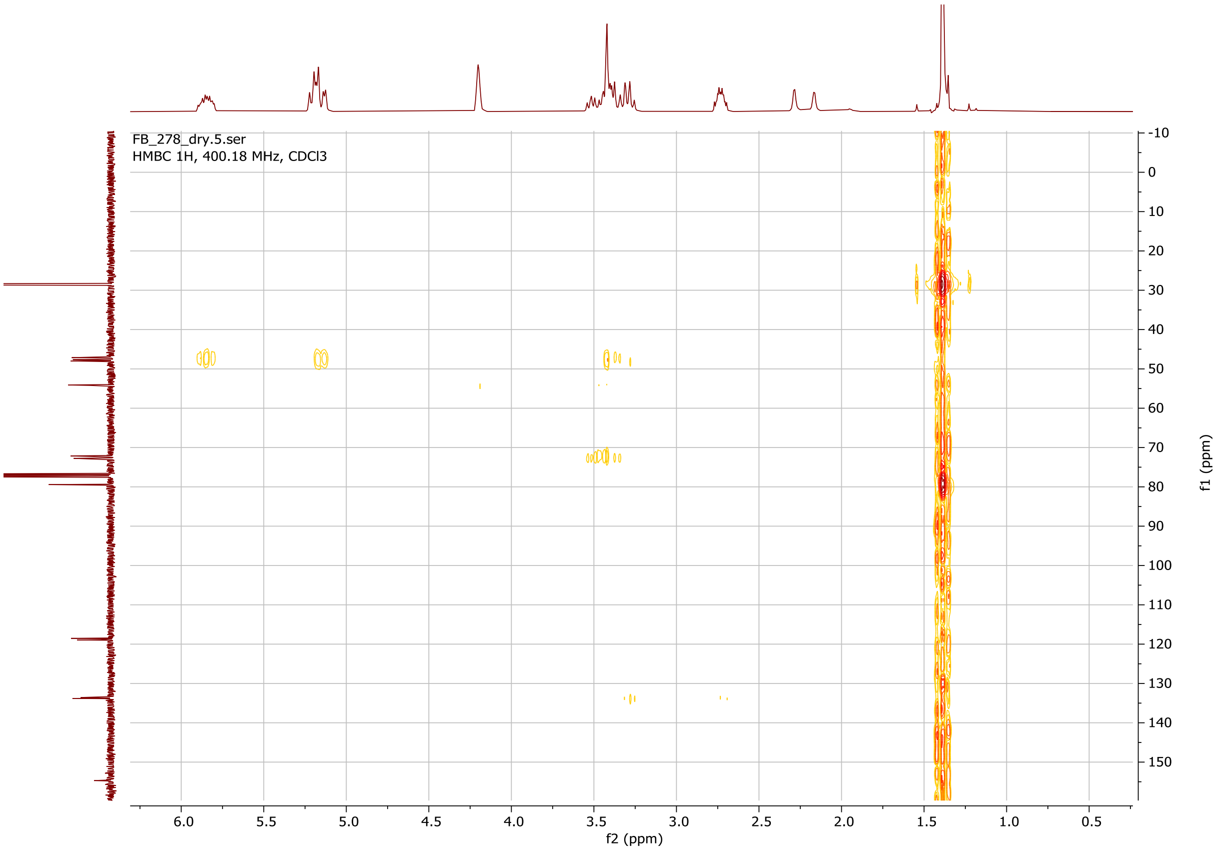

4-(2-Benzyloxy)propan-2-yl)-1-methyl-2-vinylcyclohexan-1-ol **6e**

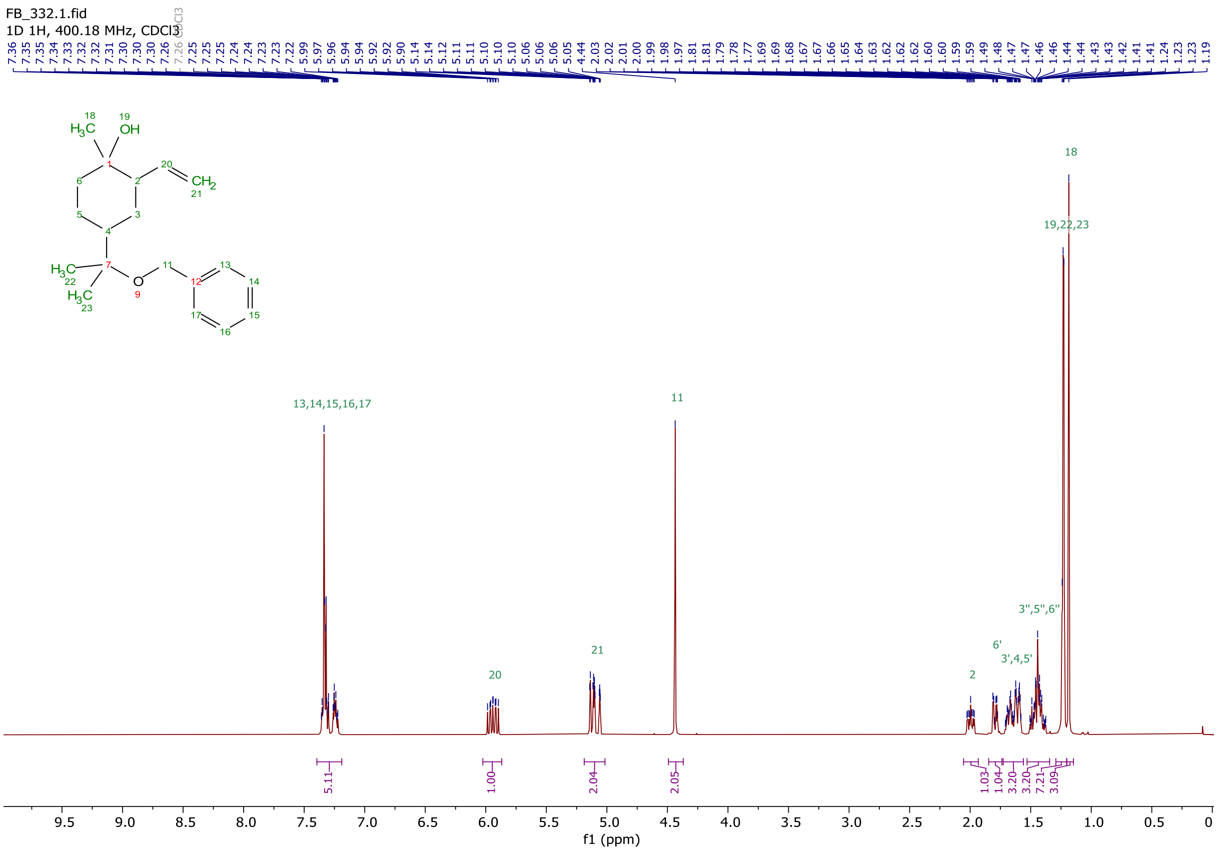

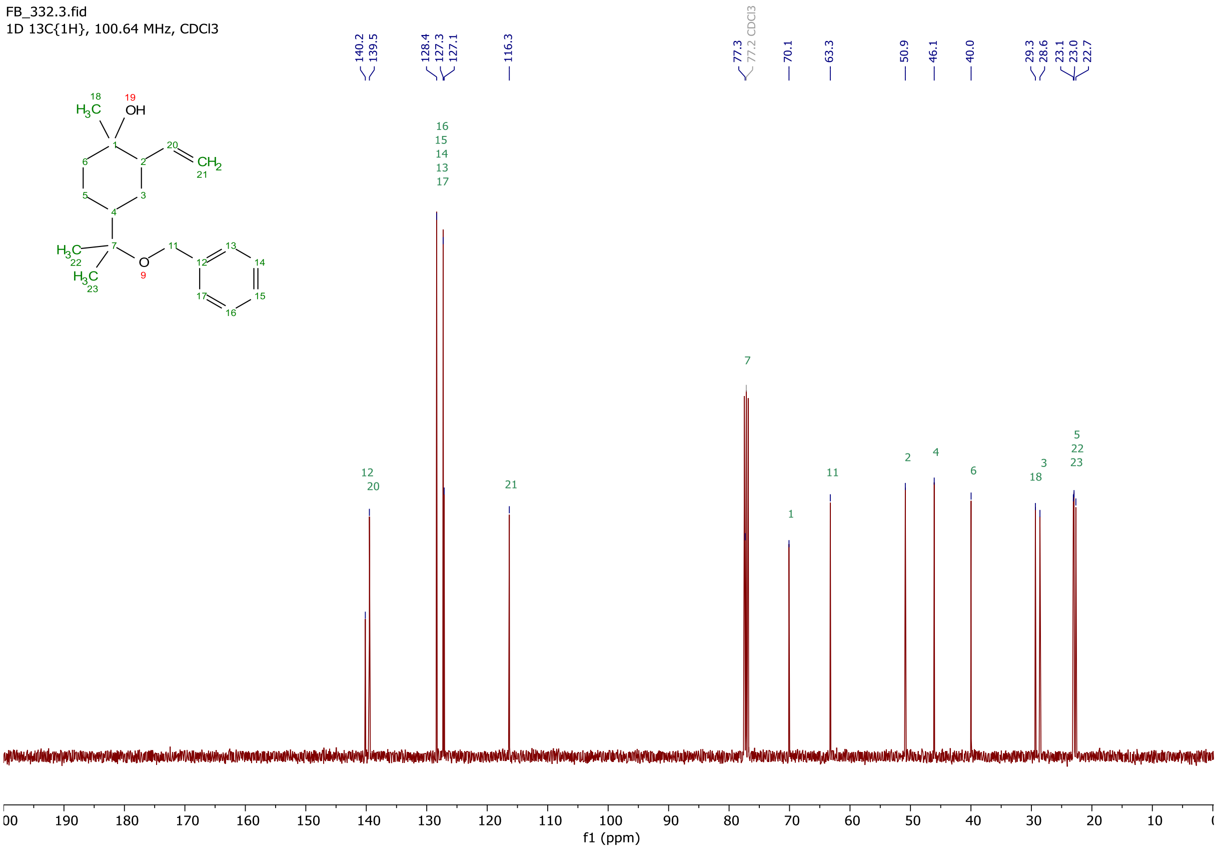
FB_332.3.fid
1D 13C{1H}, 100.64 MHz, CDCl3
140.2
139.5
128.4
127.3
127.1
116.3
77.3
77.2 CDCl3
70.1
63.3
50.9
46.1
40.0
29.3
28.6
23.1
23.0
22.7
f1 (ppm)

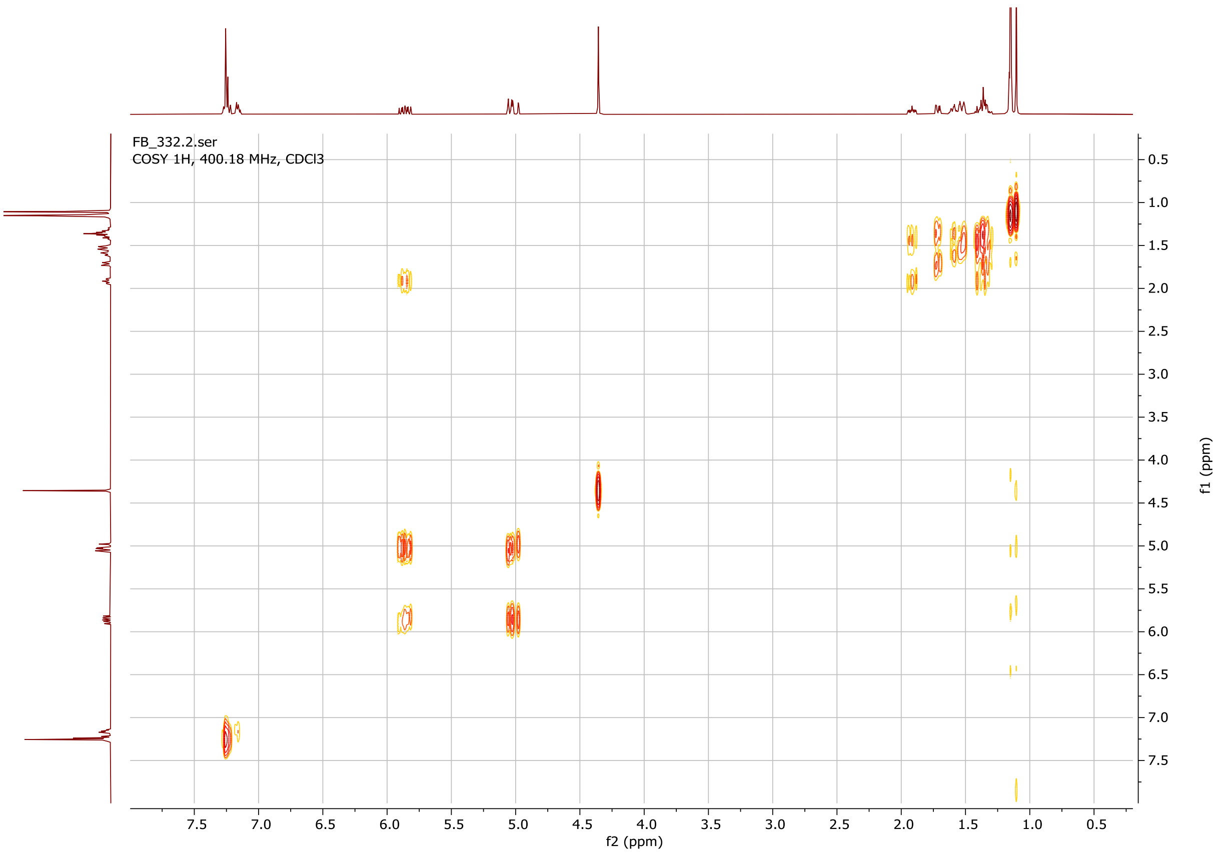
FB_332.2.ser
COSY 1H, 400.18 MHz, CDCl3
f2 (ppm)
f1 (ppm)

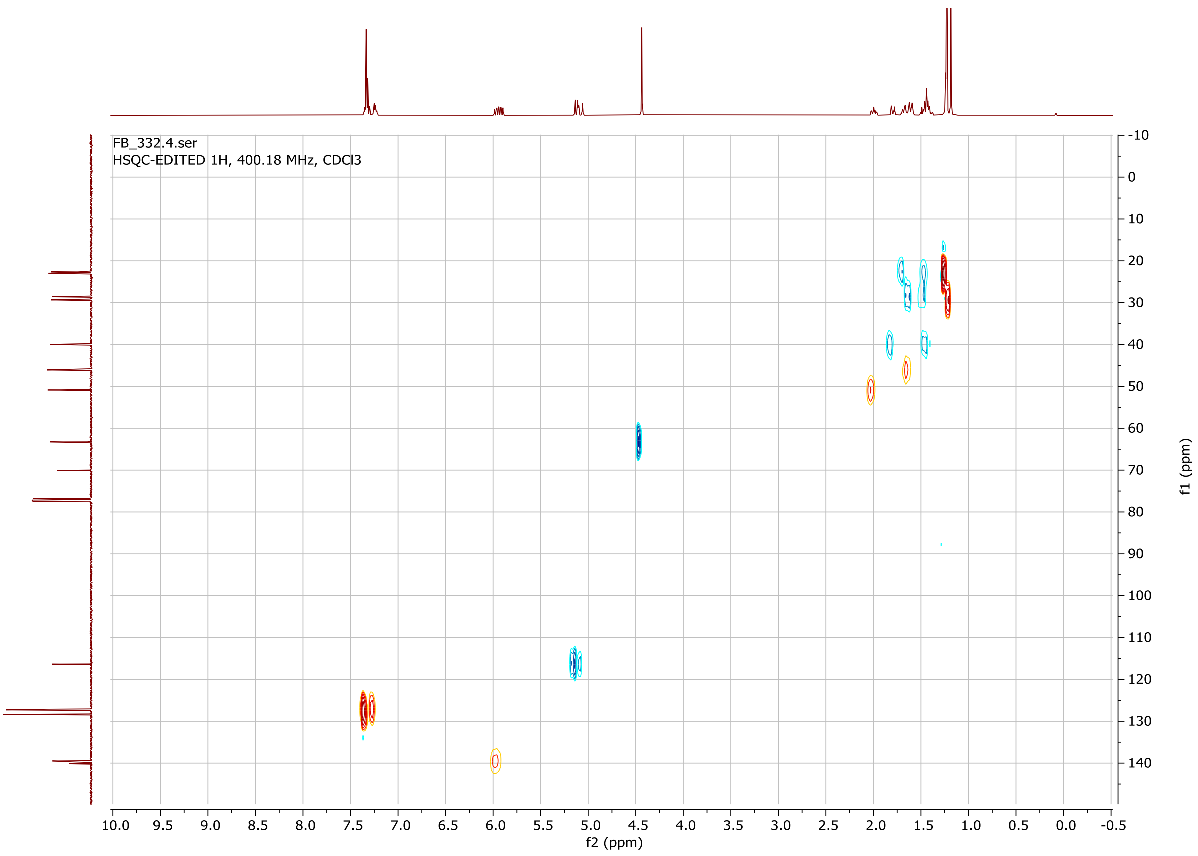
FB_332.4.ser
HSQC-EDITED 1H, 400.18 MHz, CDCl3
f2 (ppm)
f1 (ppm)

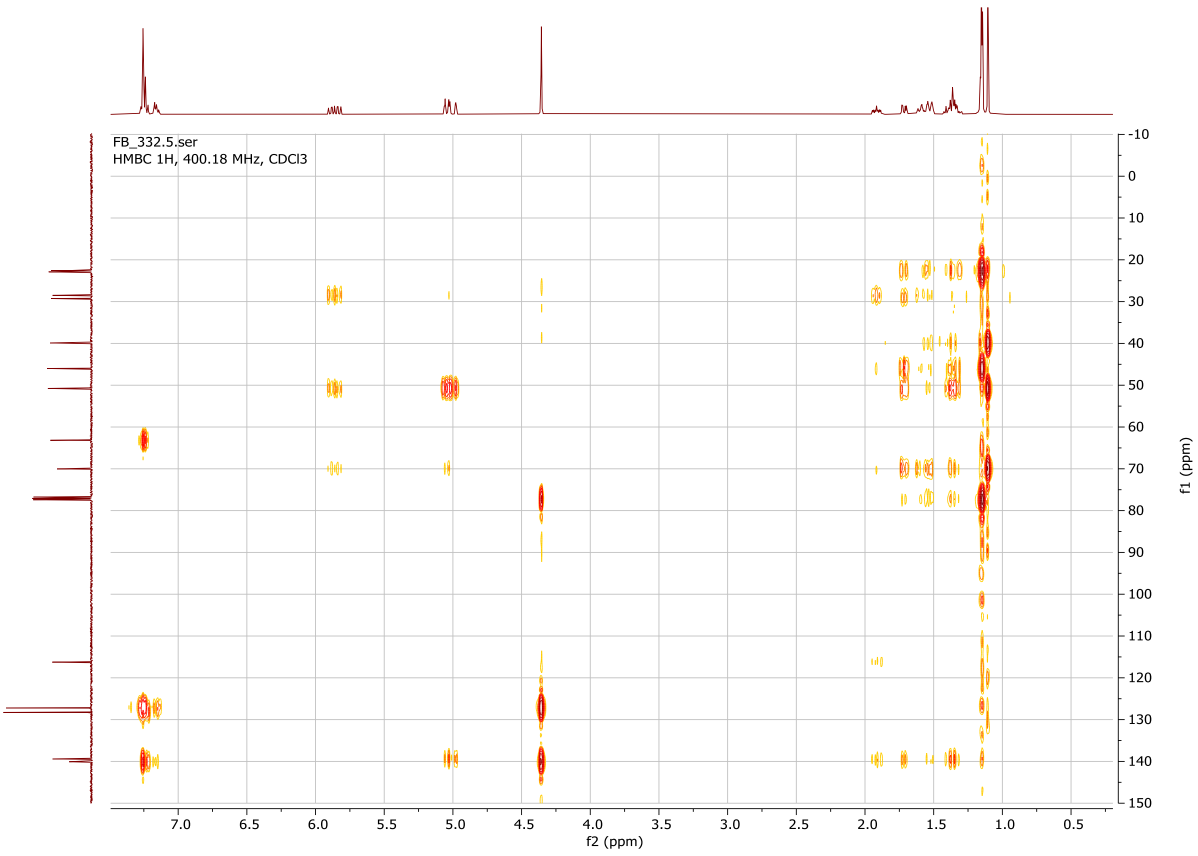
FB_332.5.ser
HMBC 1H, 400.18 MHz, CDCl3
f2 (ppm)
f1 (ppm)

2-Methyl-1-phenylbut-3-en-1-ol **6f** (*inseparable 85:15 diastereomers mixture*)

FB_233.1.fid
1D 1H, 400.18 MHz, CDCl3

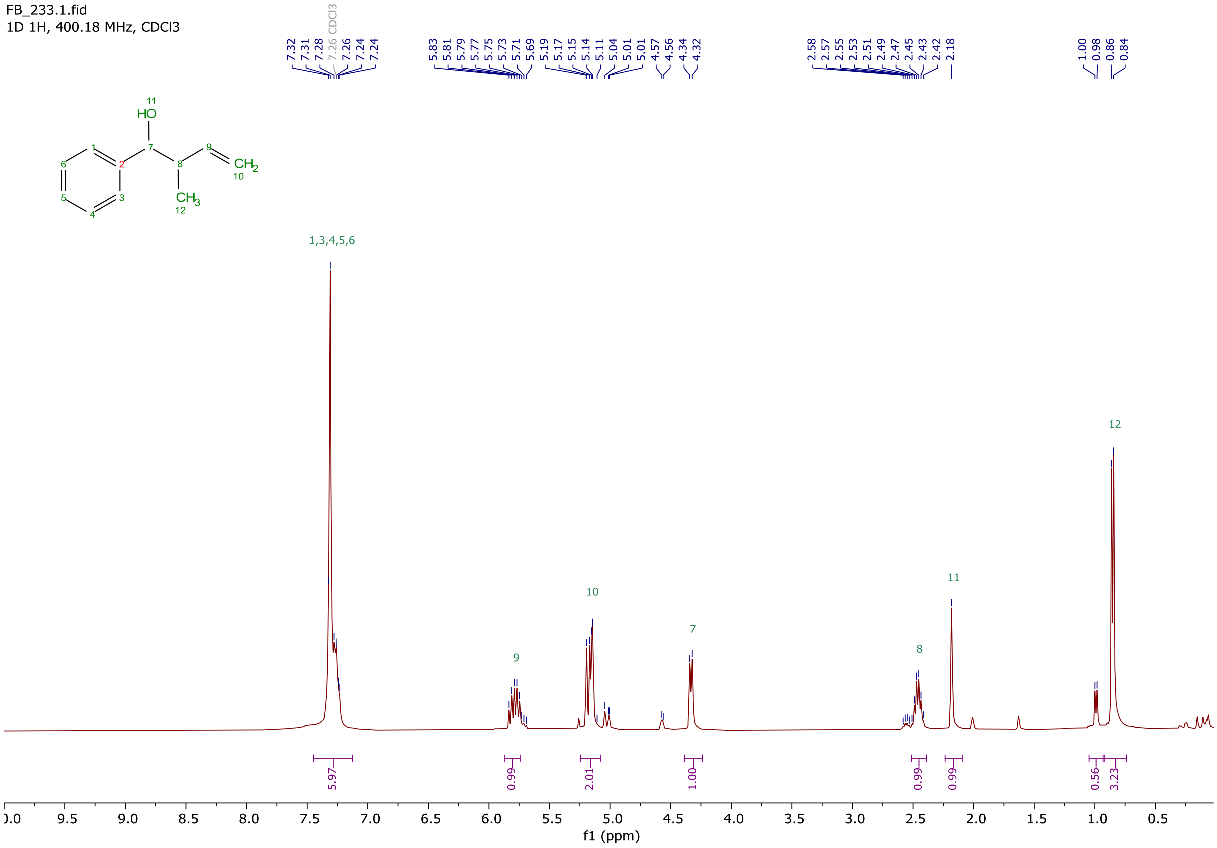

FB_233.3.fid
1D 13C{1H}, 100.64 MHz, CDCl3

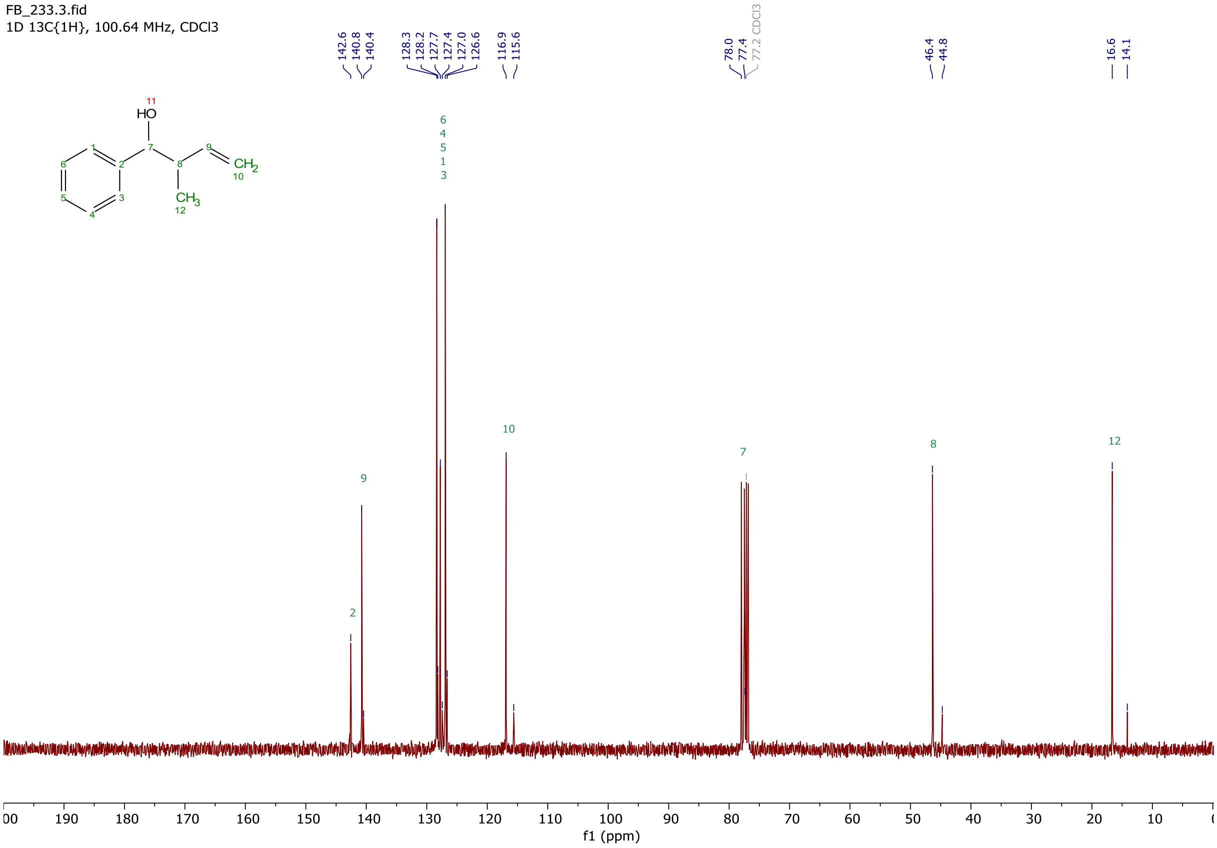

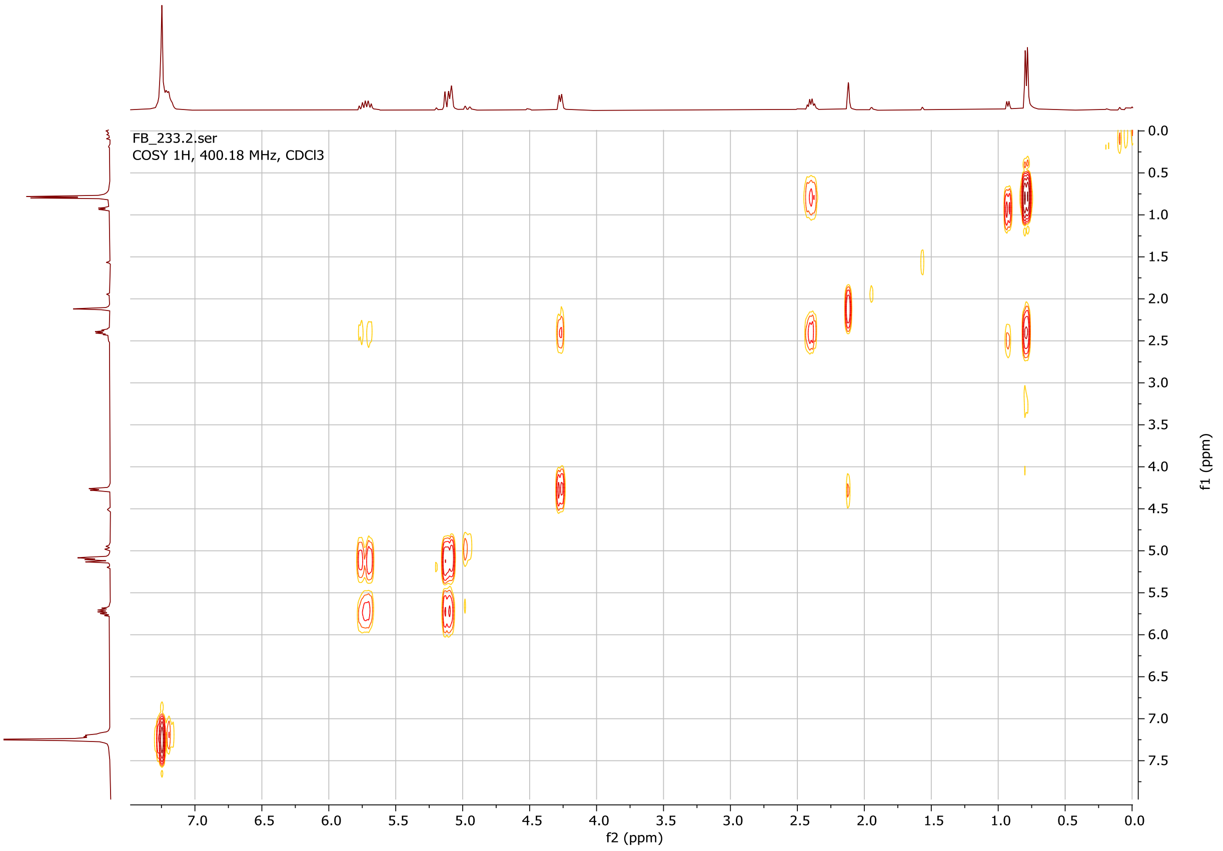
FB_233.2.ser
COSY 1H, 400.18 MHz, CDCl3
f2 (ppm)
f1 (ppm)

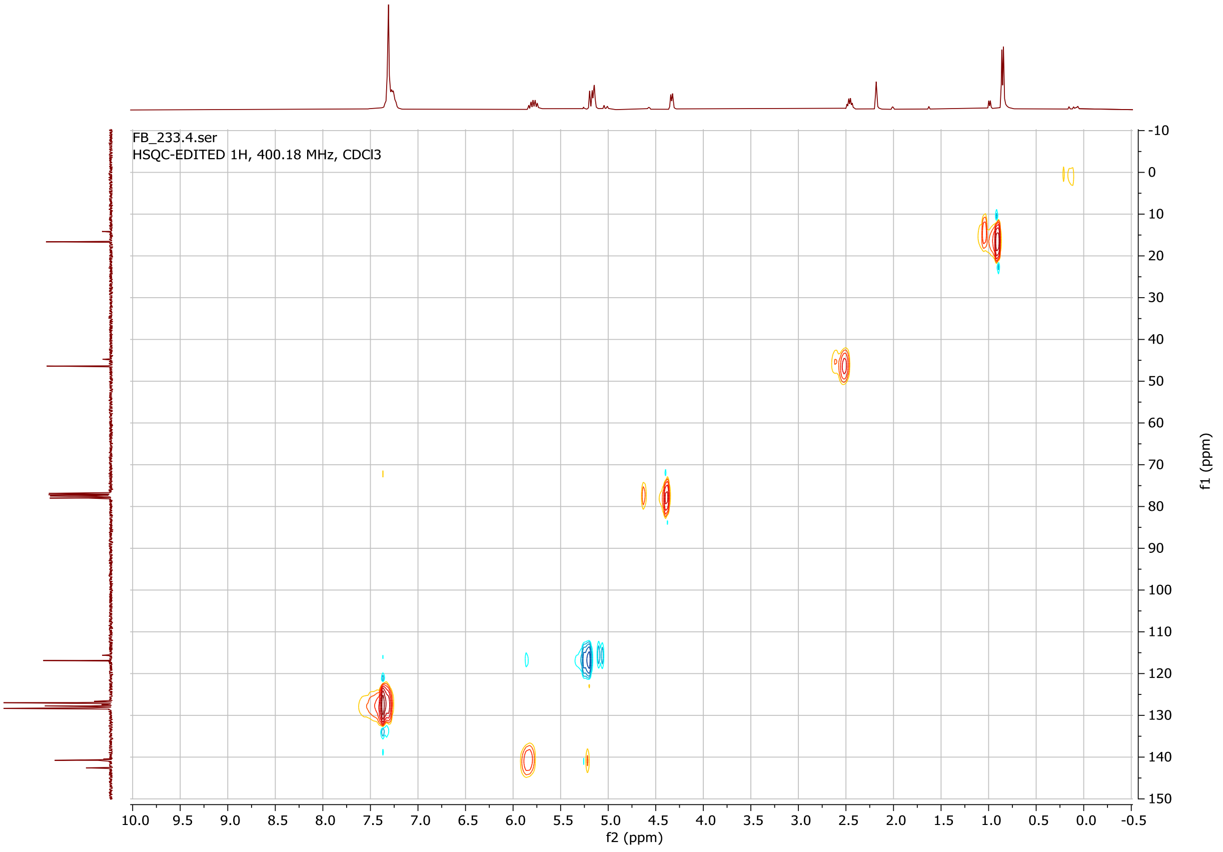
FB_233.4.ser
HSQC-EDITED 1H, 400.18 MHz, CDCl3
f2 (ppm)
f1 (ppm)

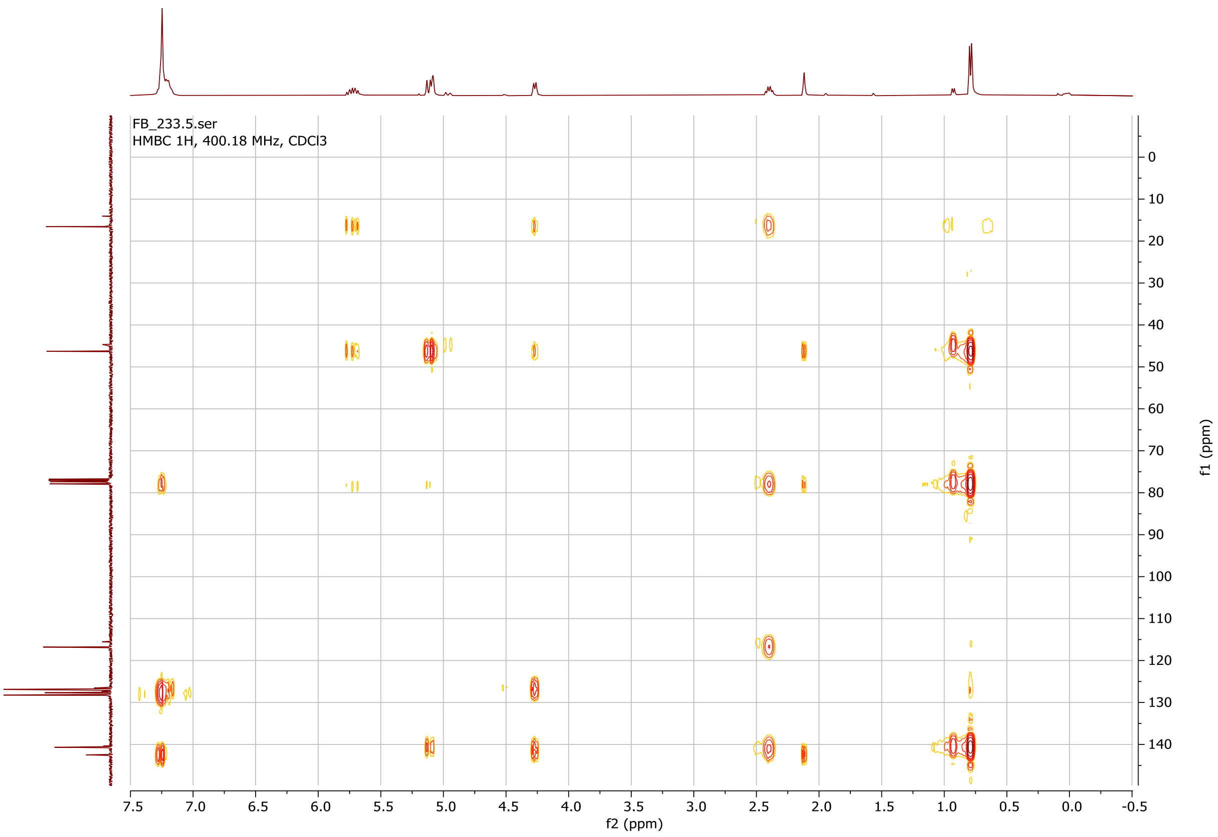

5-Vinyloctan-4-ol **6g** *(inseparable 86:14 diastereomers mixture)*

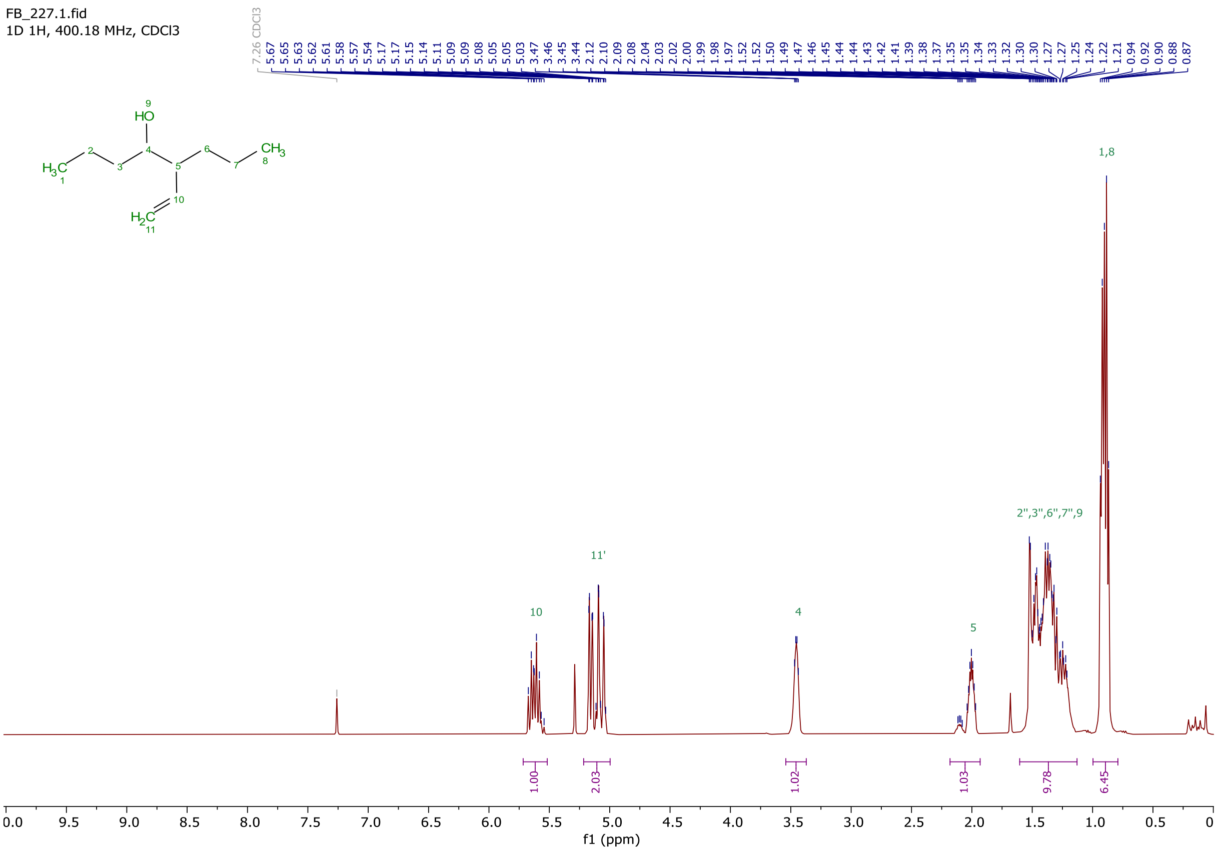

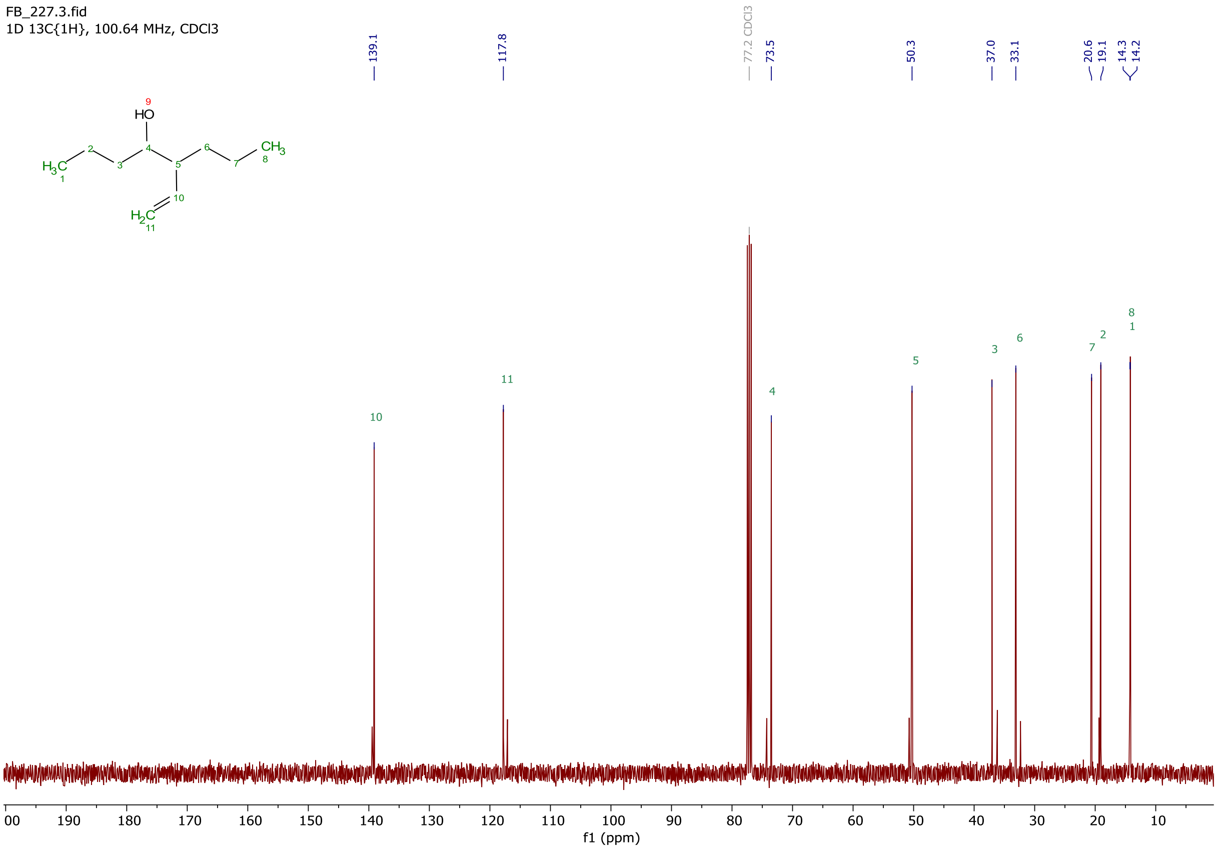
FB_227.3.fid
1D 13C{1H}, 100.64 MHz, CDCl3
139.1
117.8
77.2 CDCl3
73.5
50.3
37.0
33.1
20.6
19.1
14.3
14.2
f1 (ppm)

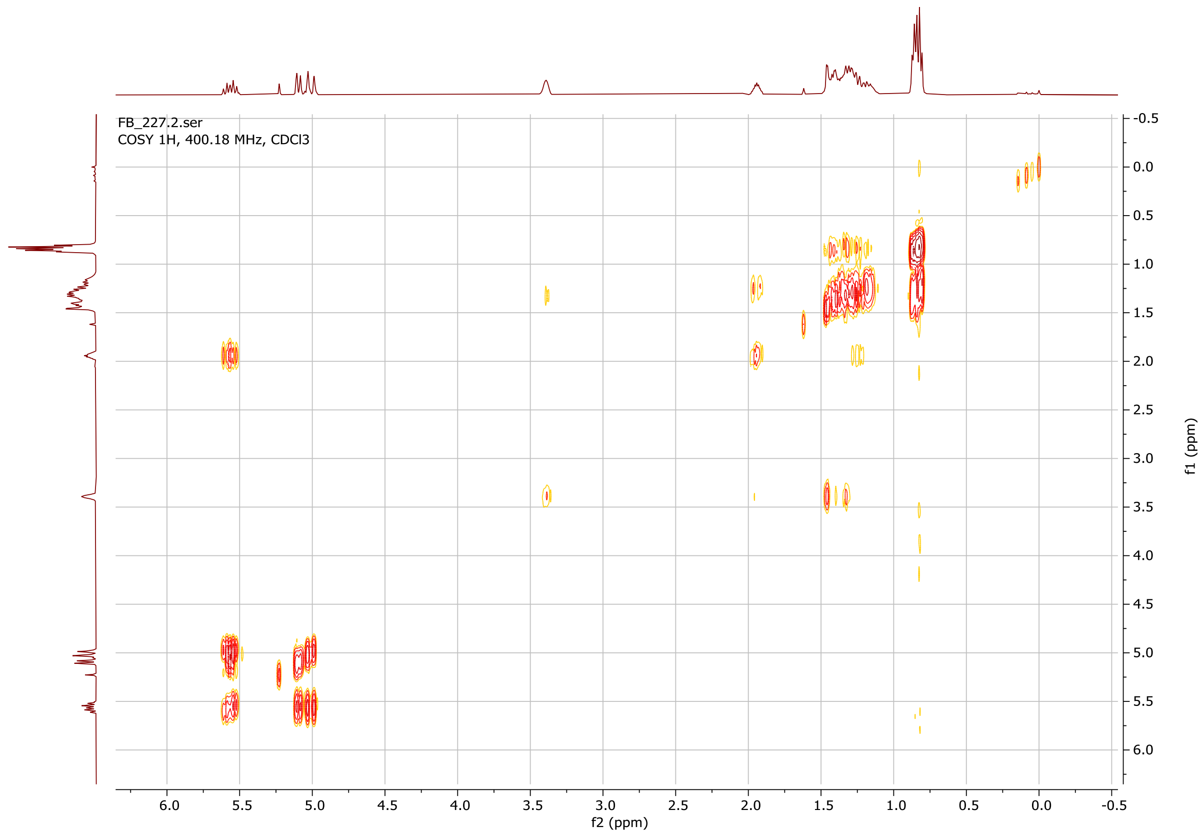
FB_227.2.ser
COSY 1H, 400.18 MHz, CDCl3
f2 (ppm)
f1 (ppm)

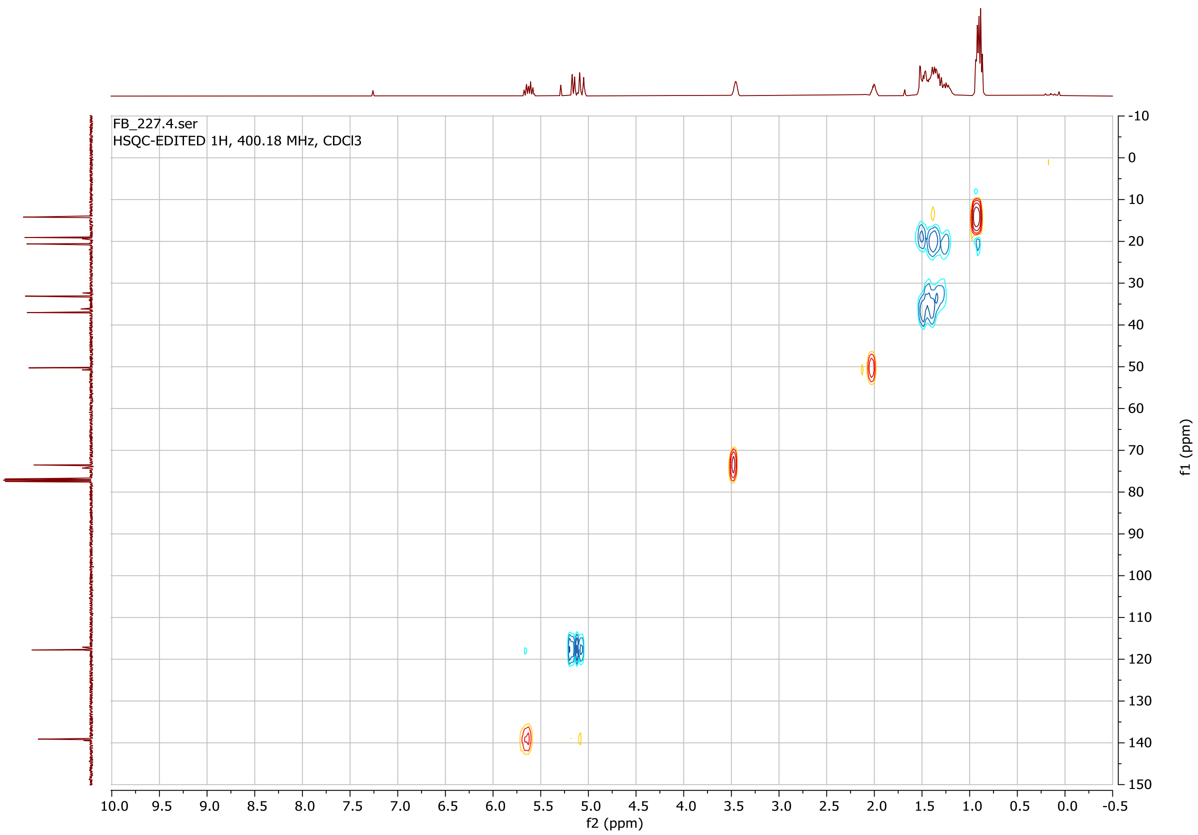
FB_227.4.ser
HSQC-EDITED 1H, 400.18 MHz, CDCl3
f2 (ppm)
f1 (ppm)

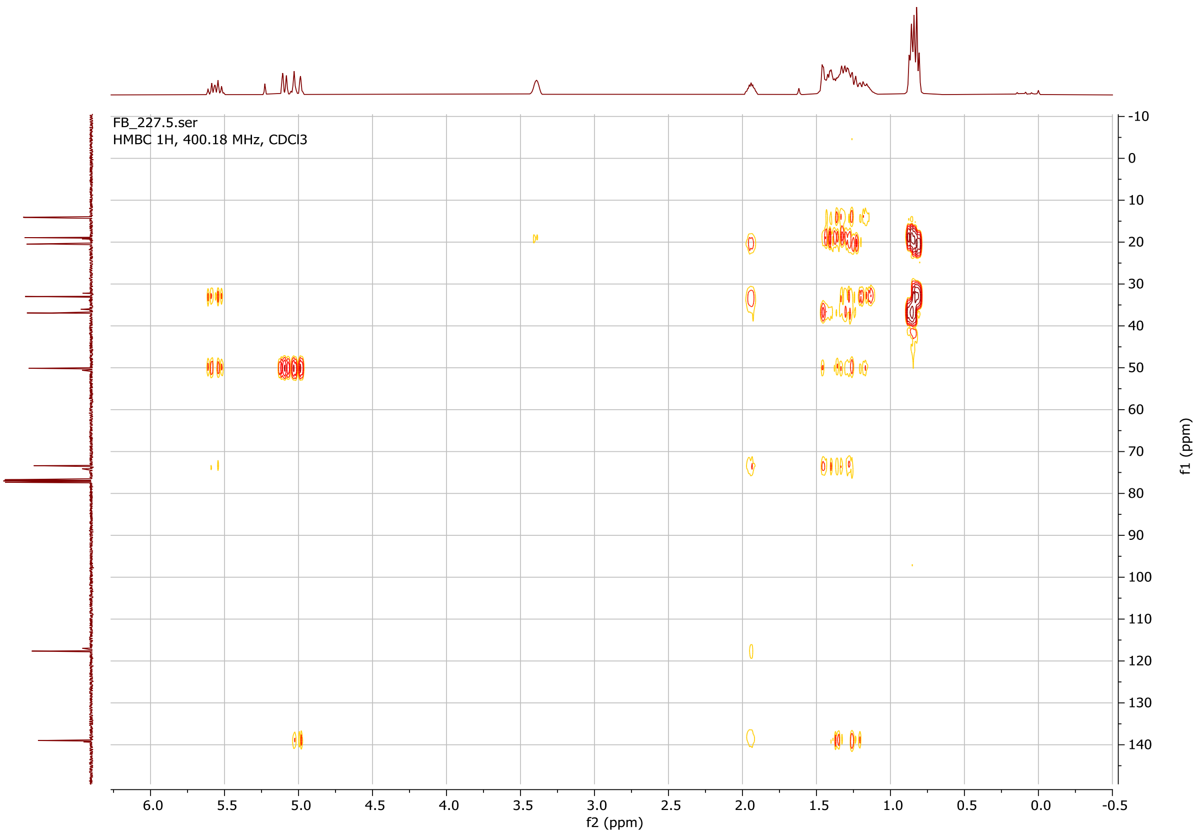
FB_227.5.ser
HMBC 1H, 400.18 MHz, CDCl3
f2 (ppm)
f1 (ppm)

1-Phenyl-2-vinylpropane-1,3-diol **6h** *(inseparable 87:13 diastereomers mixture)*

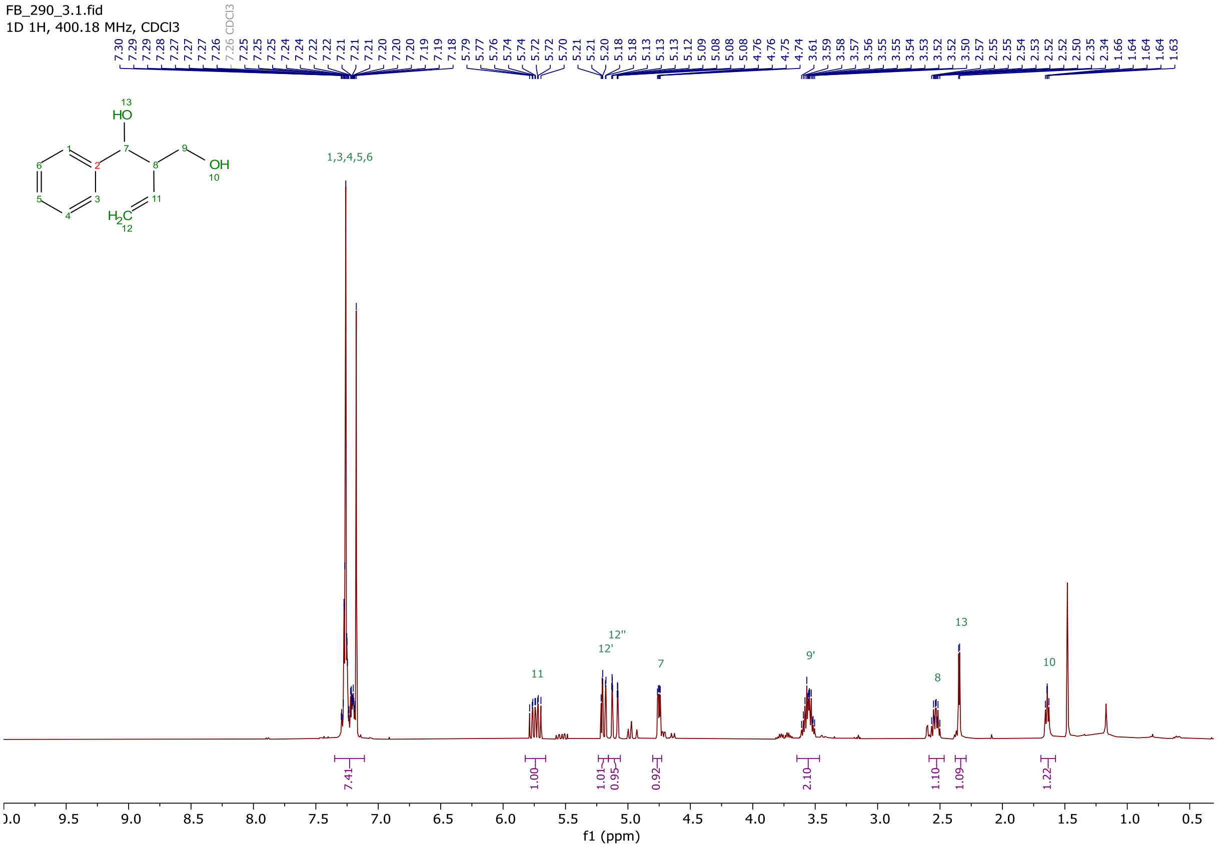

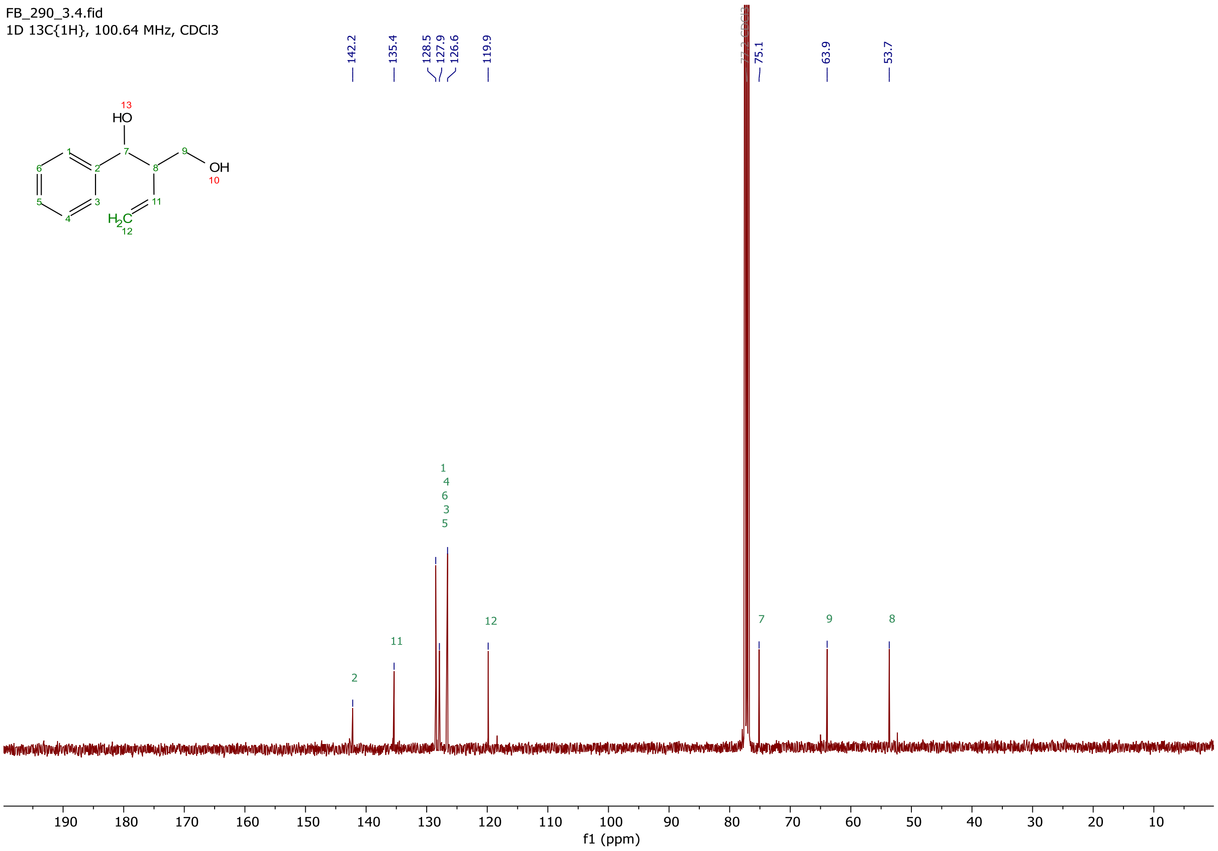

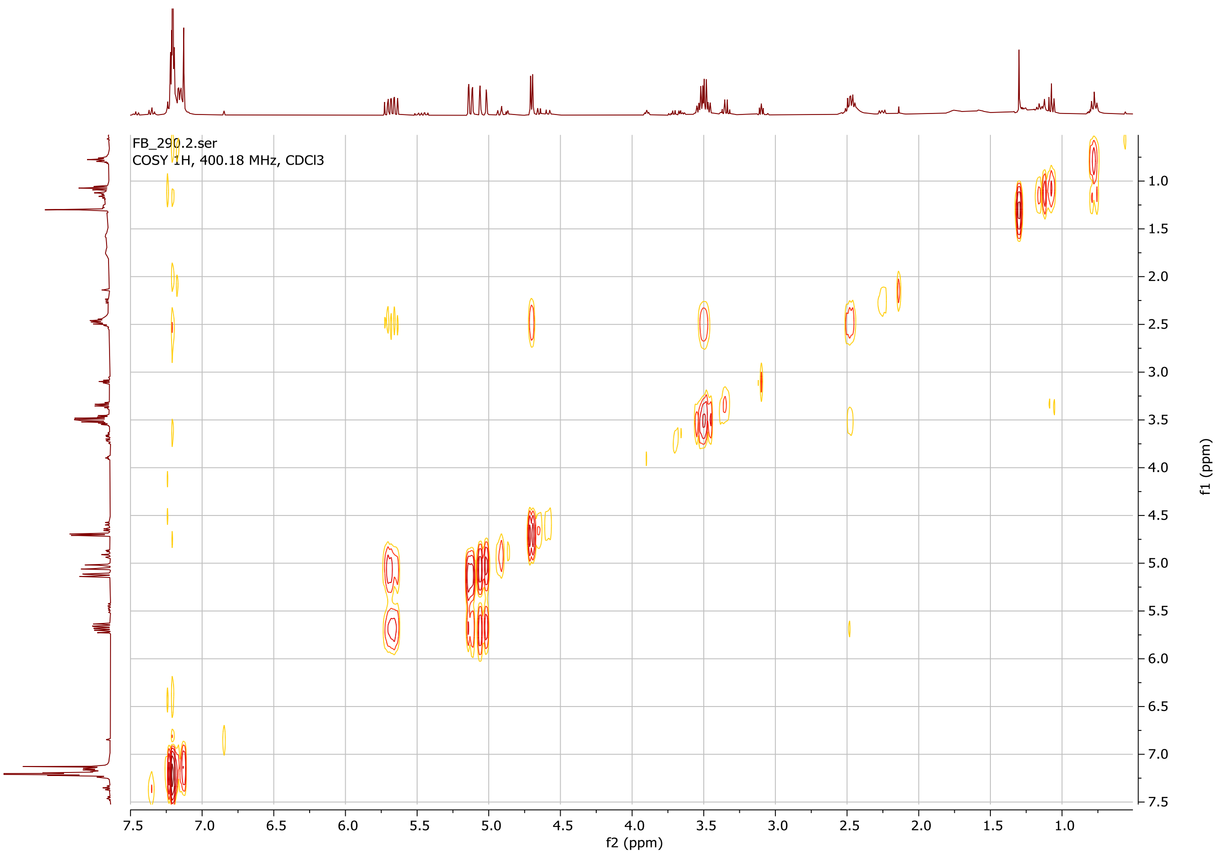
FB_290.2.ser
COSY 1H, 400.18 MHz, CDCl3
f1 (ppm)
f2 (ppm)

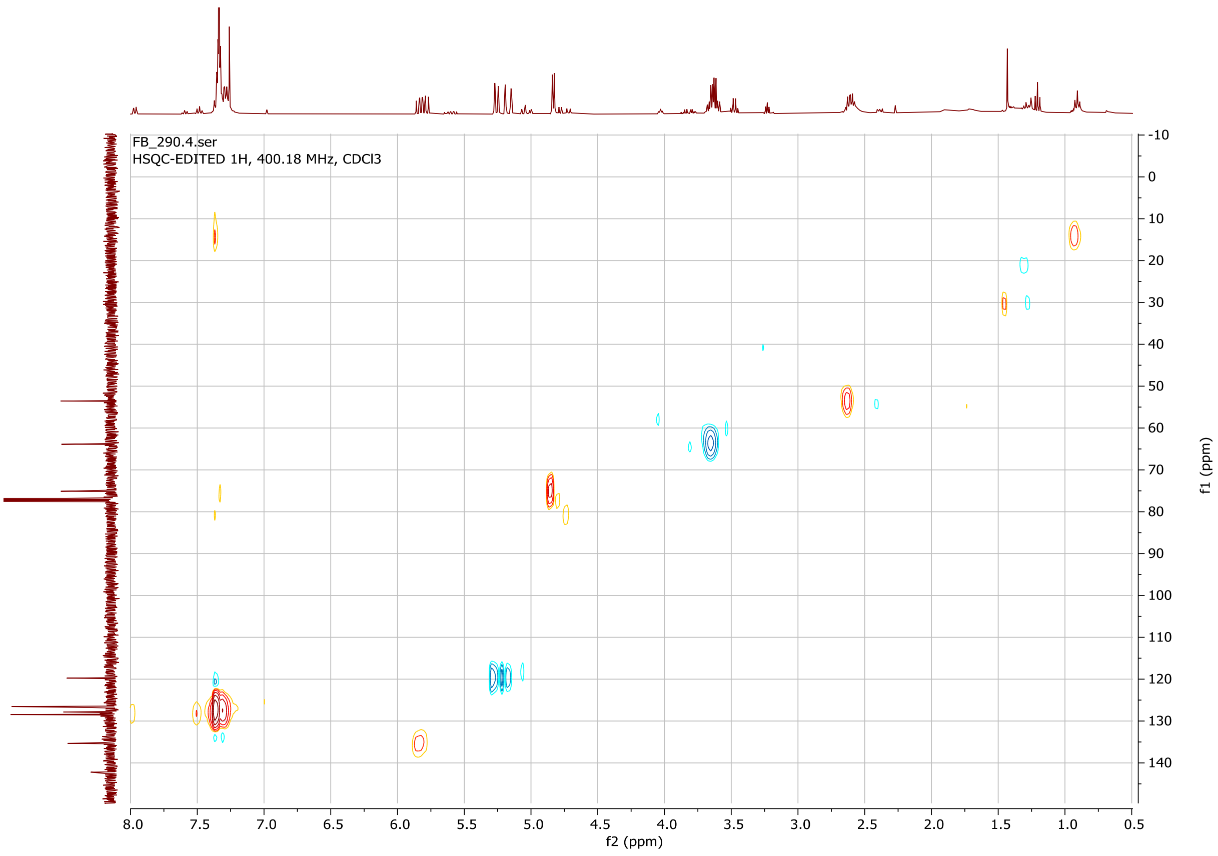
FB_290.4.ser
HSQC-EDITED 1H, 400.18 MHz, CDCl3
f1 (ppm)
f2 (ppm)

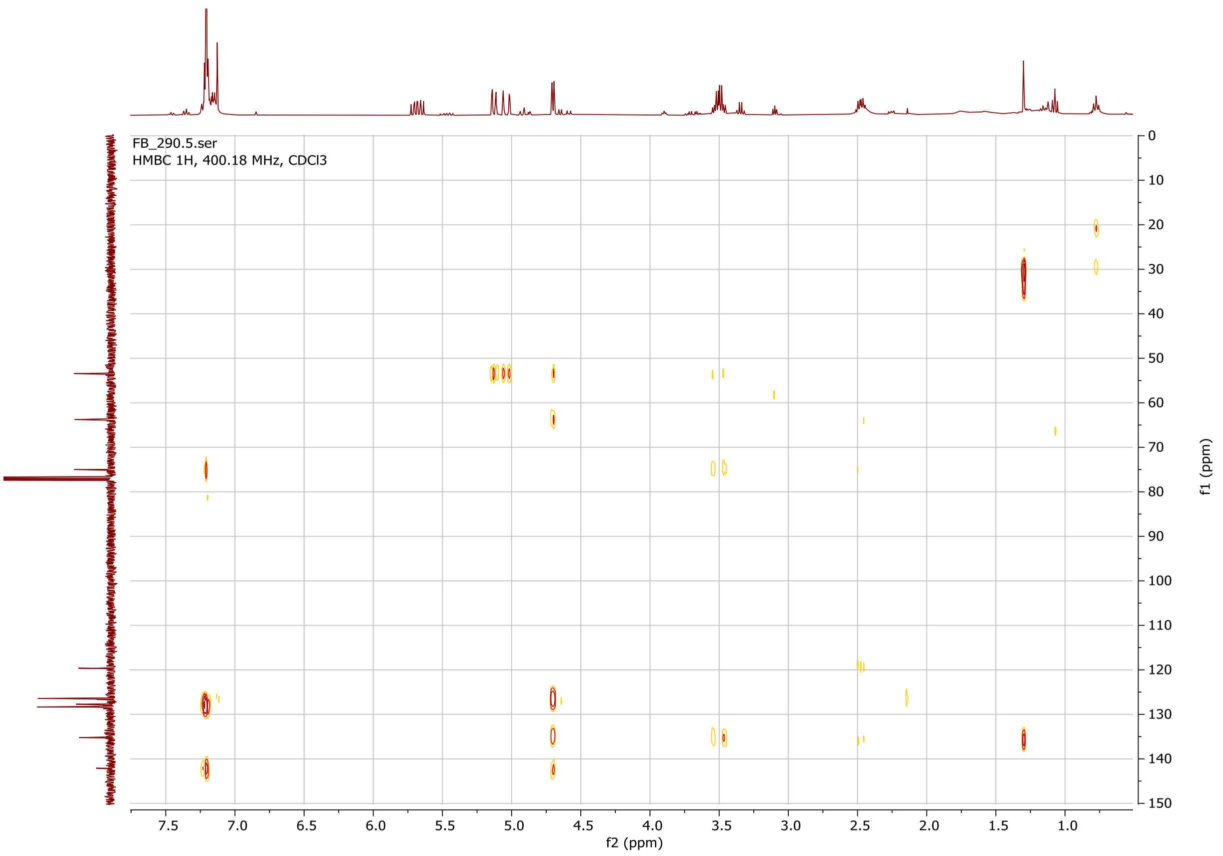

2-Vinylhexan-1,3-diol **6i** *(inseparable 88:12 diastereomers mixture)*

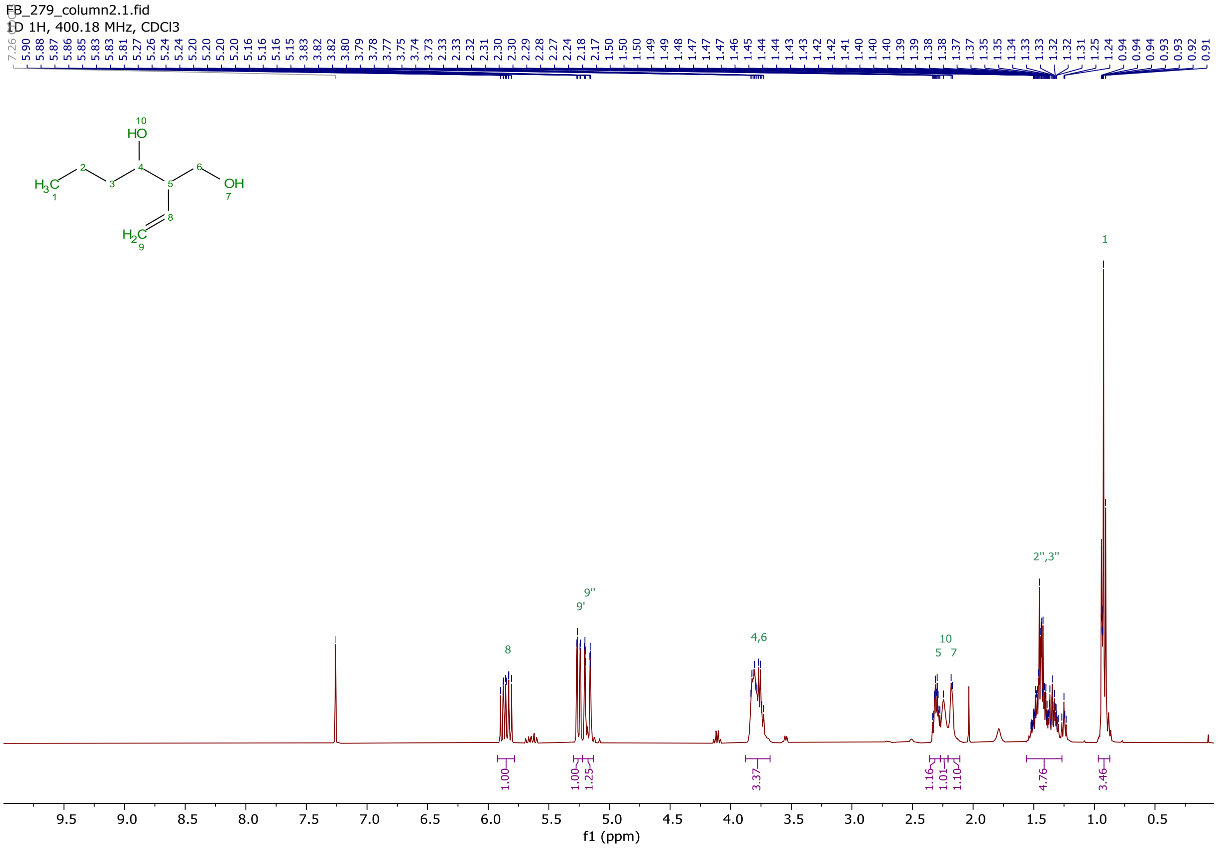

FB_279_column2.3.fid
1D 13C{1H}, 100.64 MHz, CDCl3

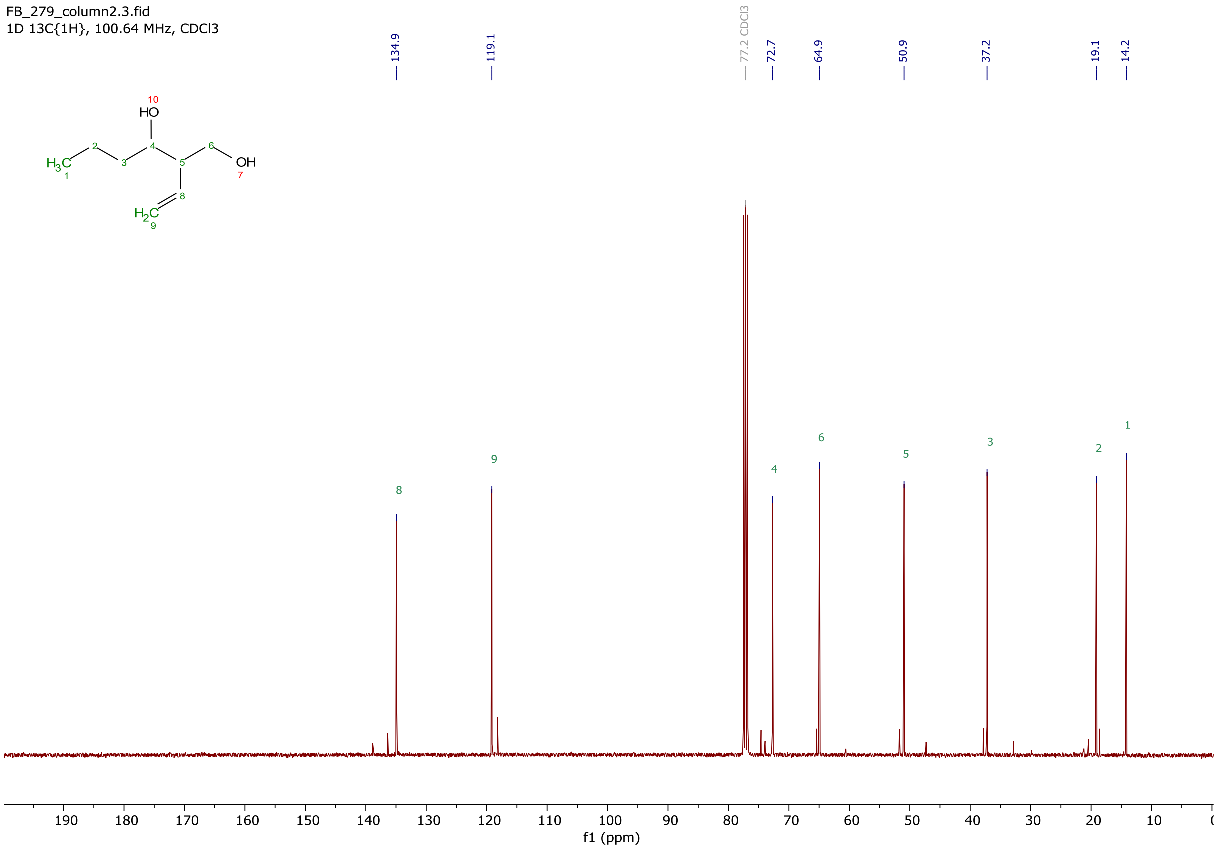

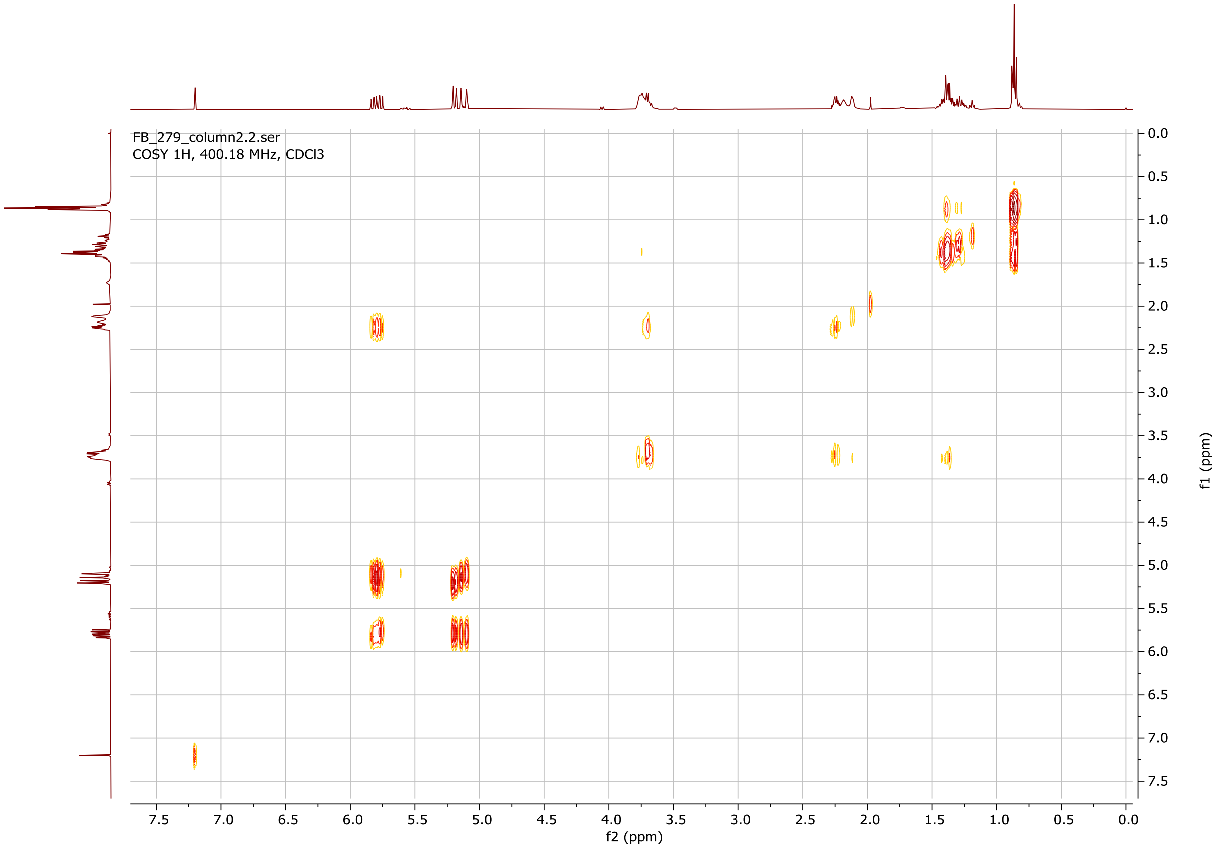

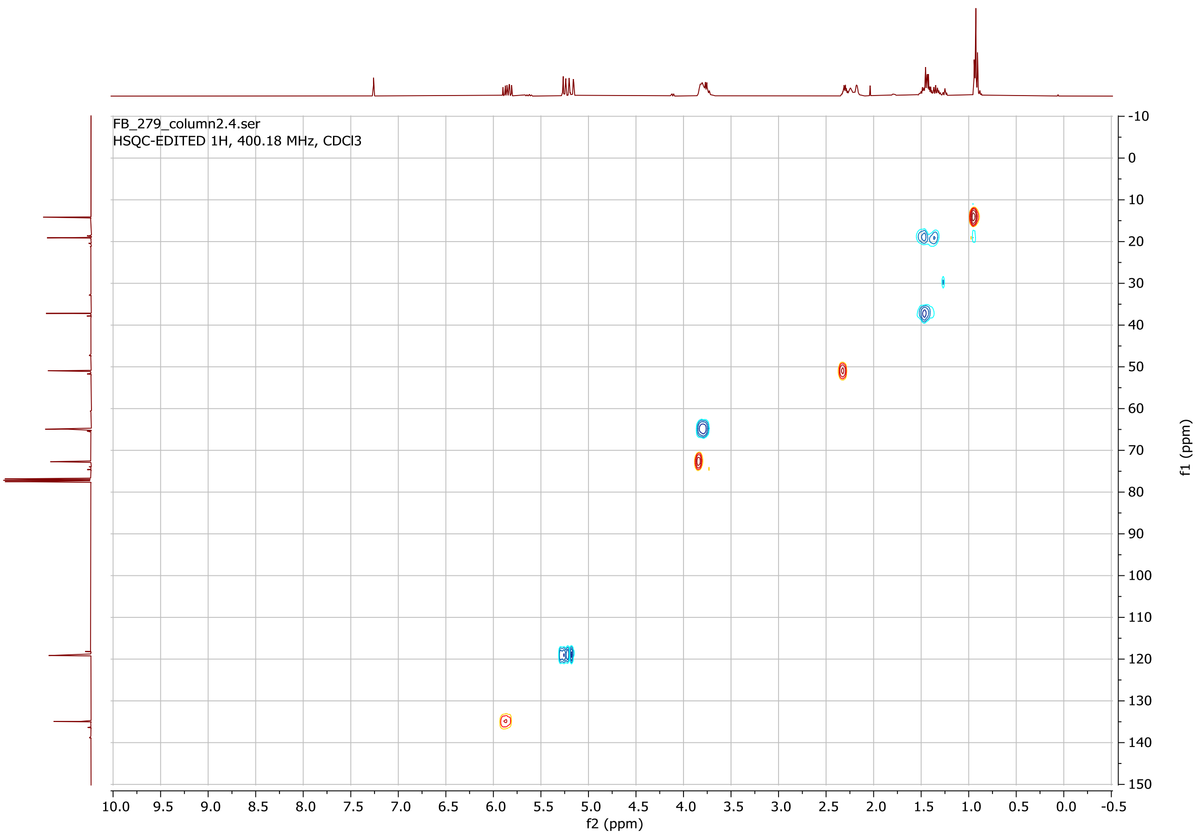
FB_279_column2.4.ser
HSQC-EDITED 1H, 400.18 MHz, CDCl3
f2 (ppm)
f1 (ppm)

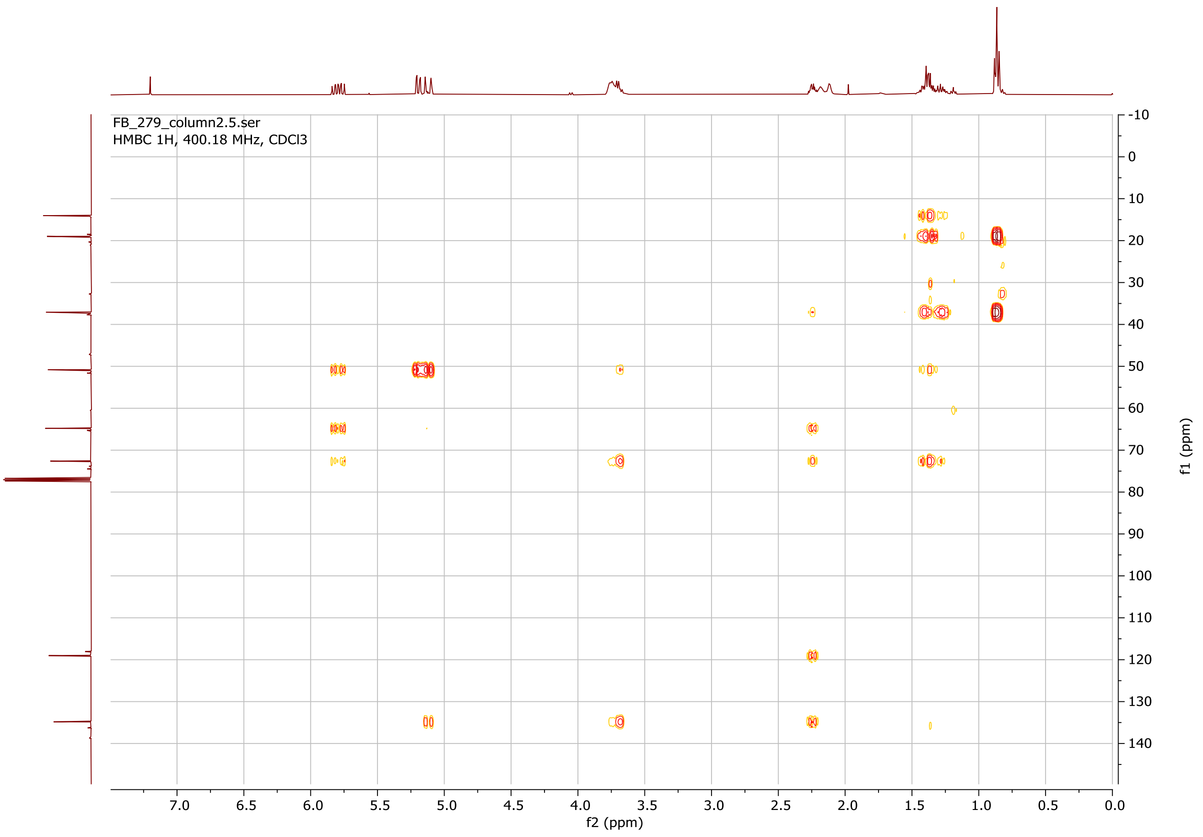
FB_279_column2.5.ser
HMBC 1H, 400.18 MHz, CDCl3
f2 (ppm)
f1 (ppm)

2-Vinylhexan-1-ol **6j**

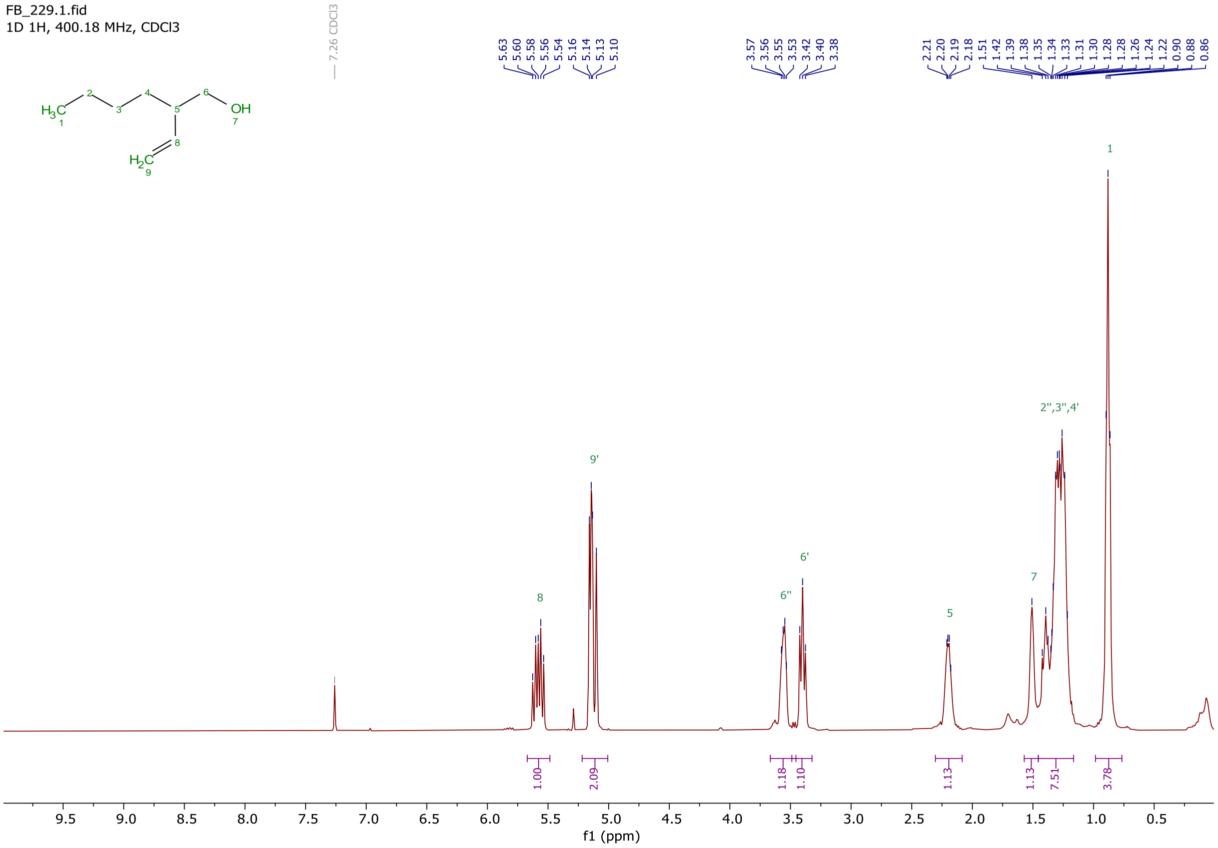

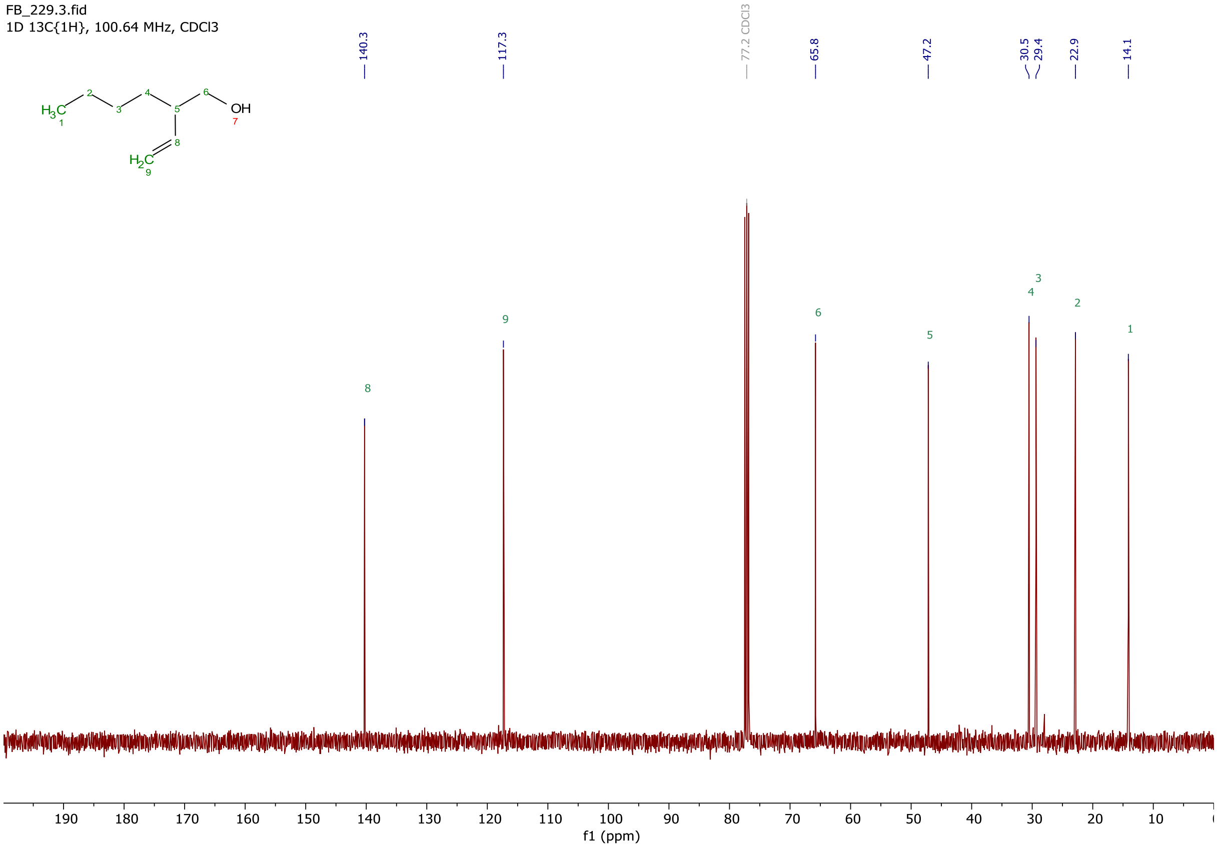

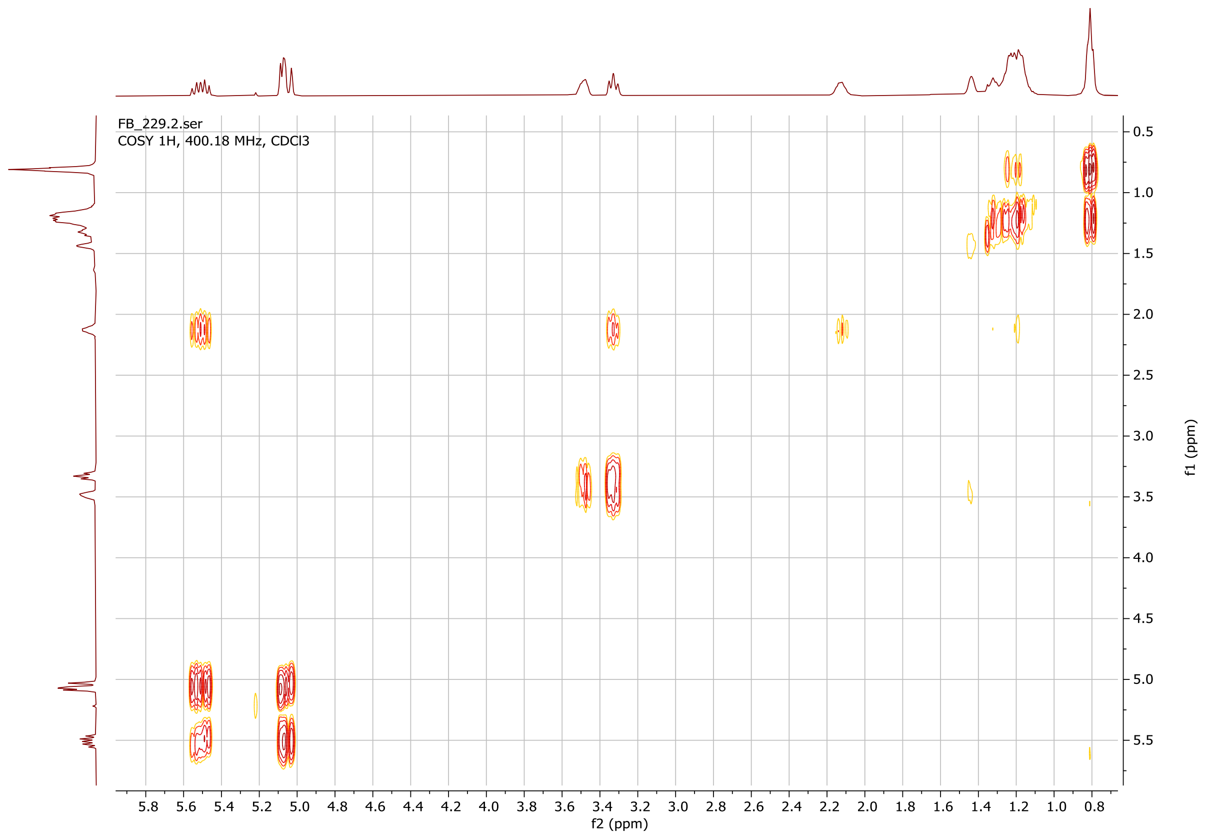
FB_229.2.ser
COSY 1H, 400.18 MHz, CDCl3
f2 (ppm)
f1 (ppm)

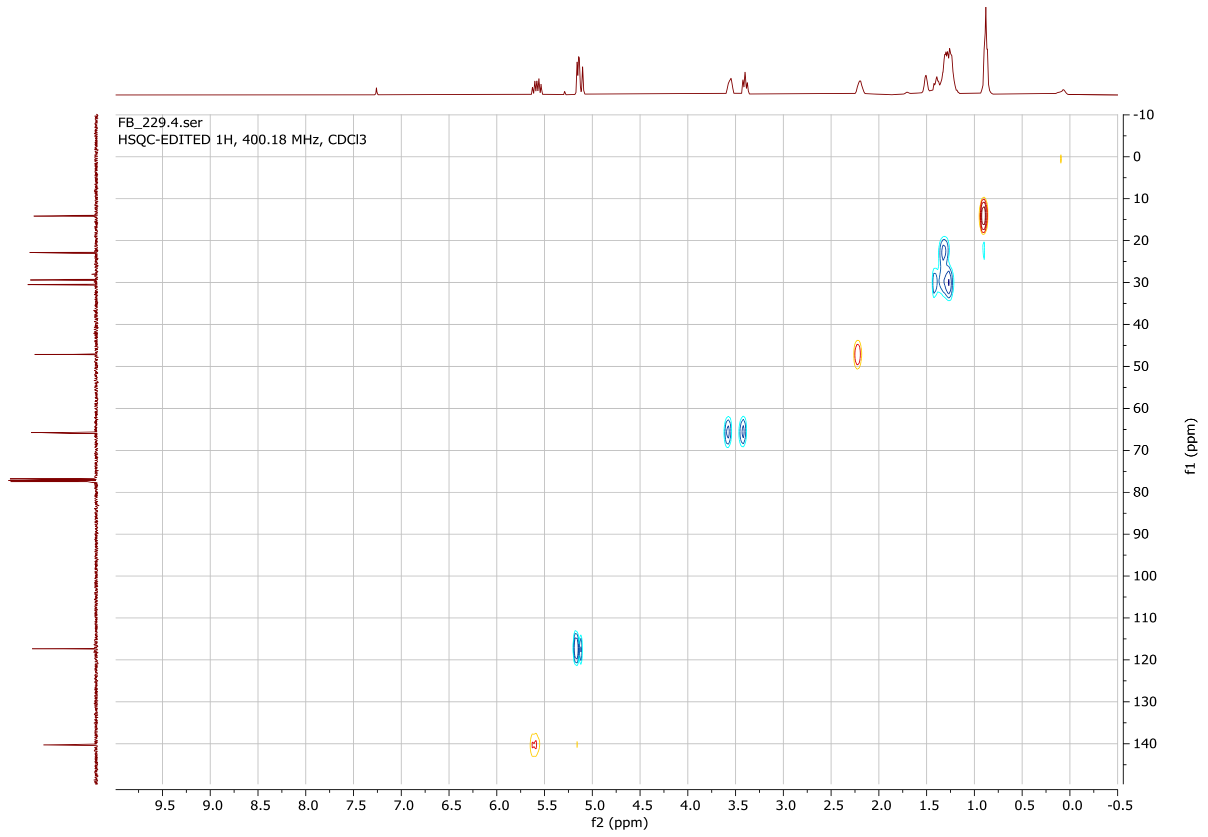
FB_229.4.ser
HSQC-EDITED 1H, 400.18 MHz, CDCl3
f2 (ppm)
f1 (ppm)

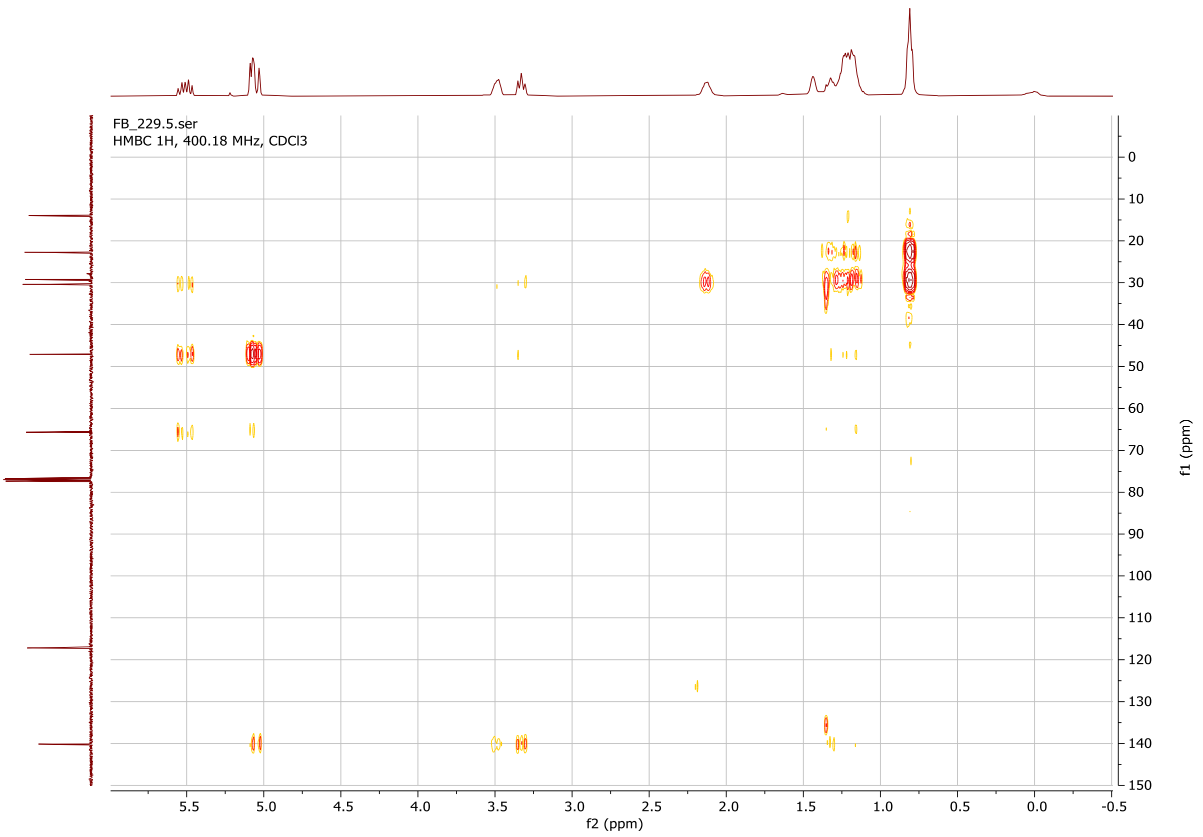

Oct-1-en-4-ol **6k**

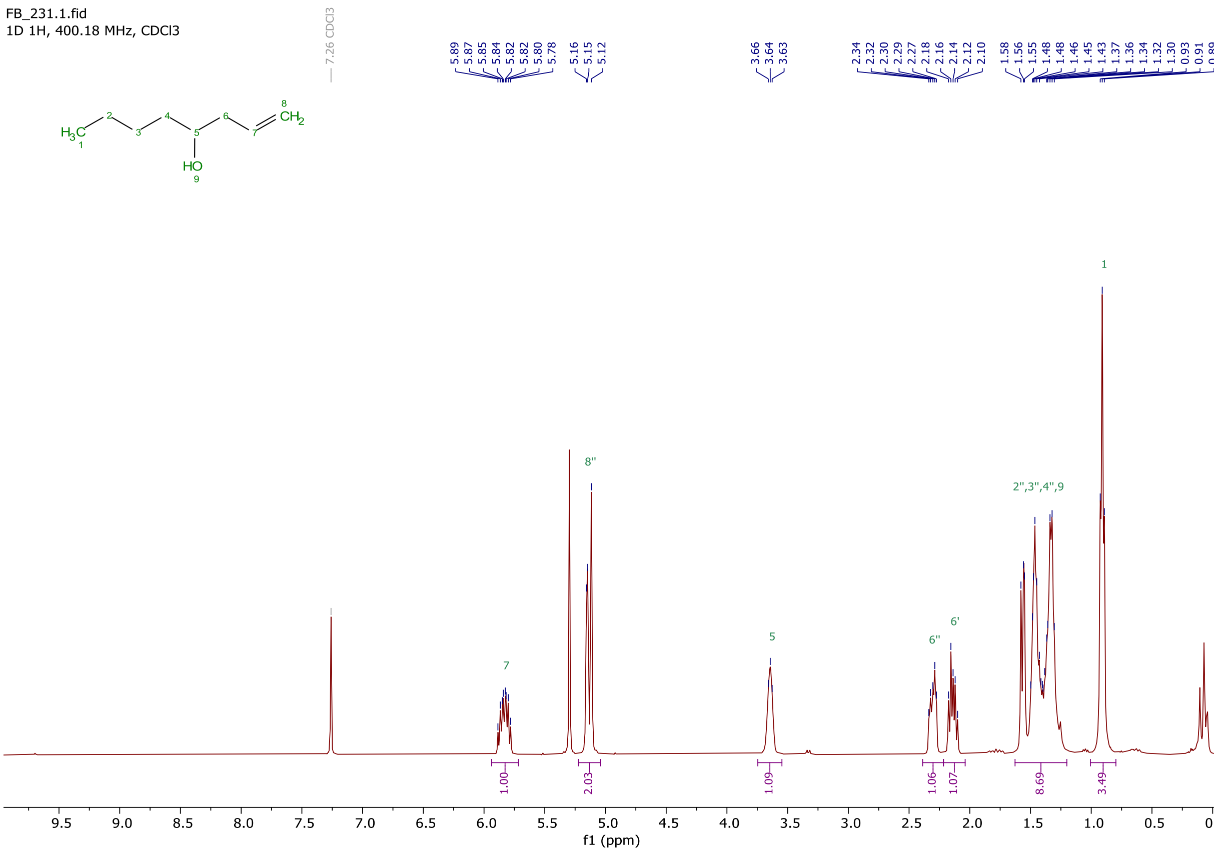

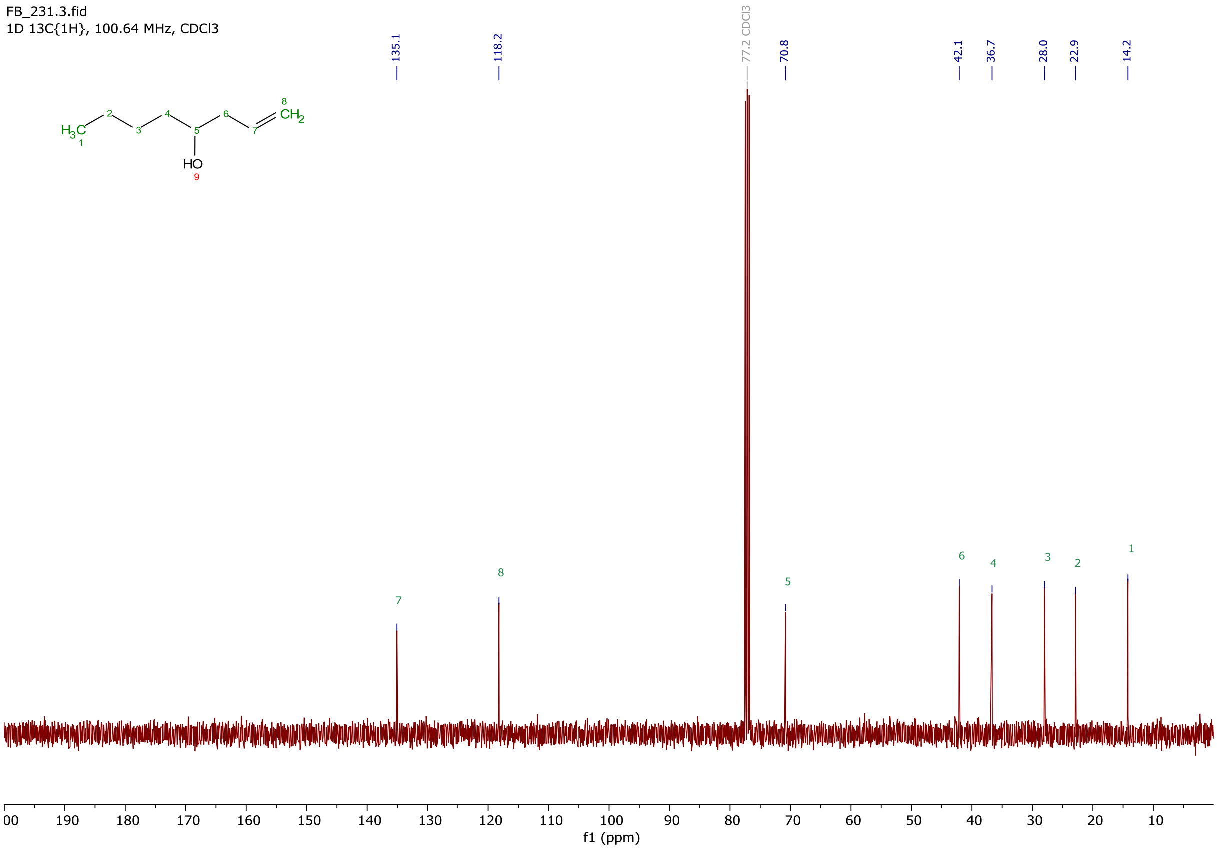
FB_231.3.fid
1D 13C{1H}, 100.64 MHz, CDCl3
135.1
118.2
77.2 CDCl3
70.8
42.1
36.7
28.0
22.9
14.2
H3C
HO
f1 (ppm)

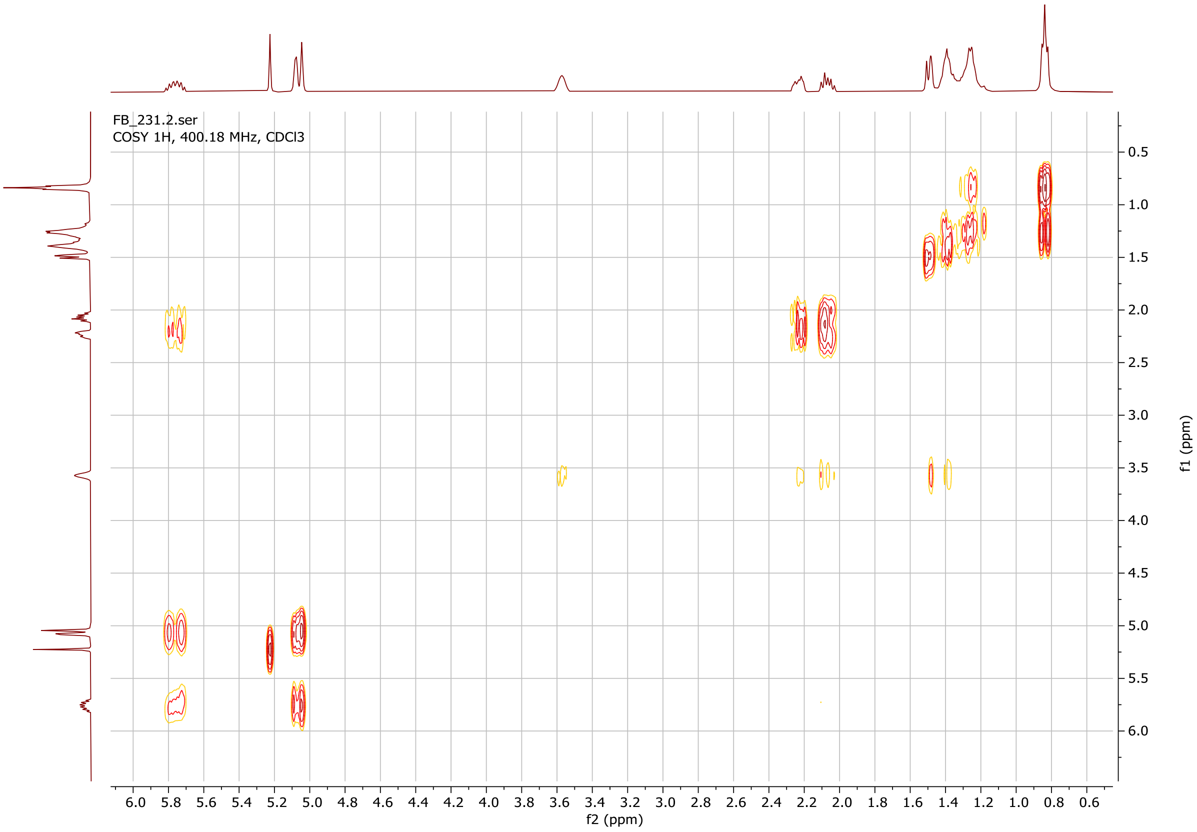
FB_231.2.ser
COSY 1H, 400.18 MHz, CDCl3
f2 (ppm)
f1 (ppm)

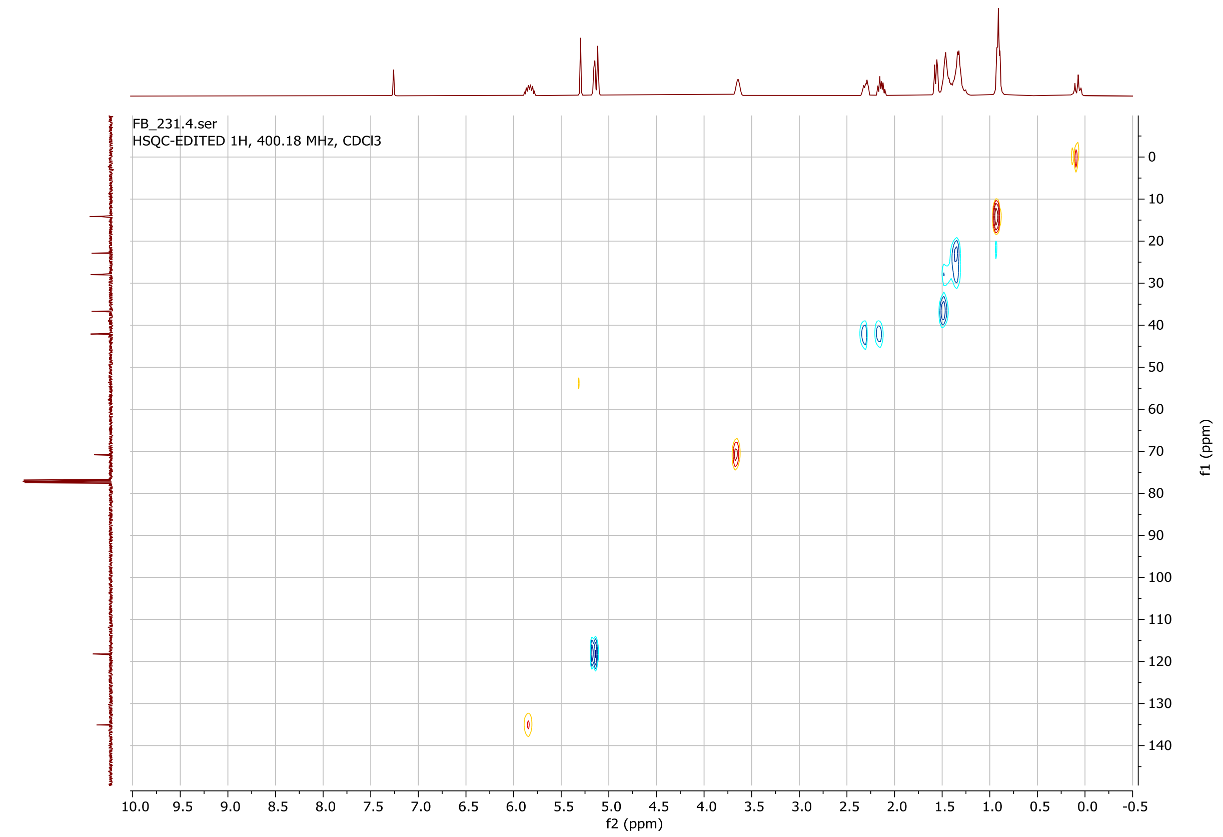
FB_231.4.ser
HSQC-EDITED 1H, 400.18 MHz, CDCl3
f2 (ppm)
f1 (ppm)

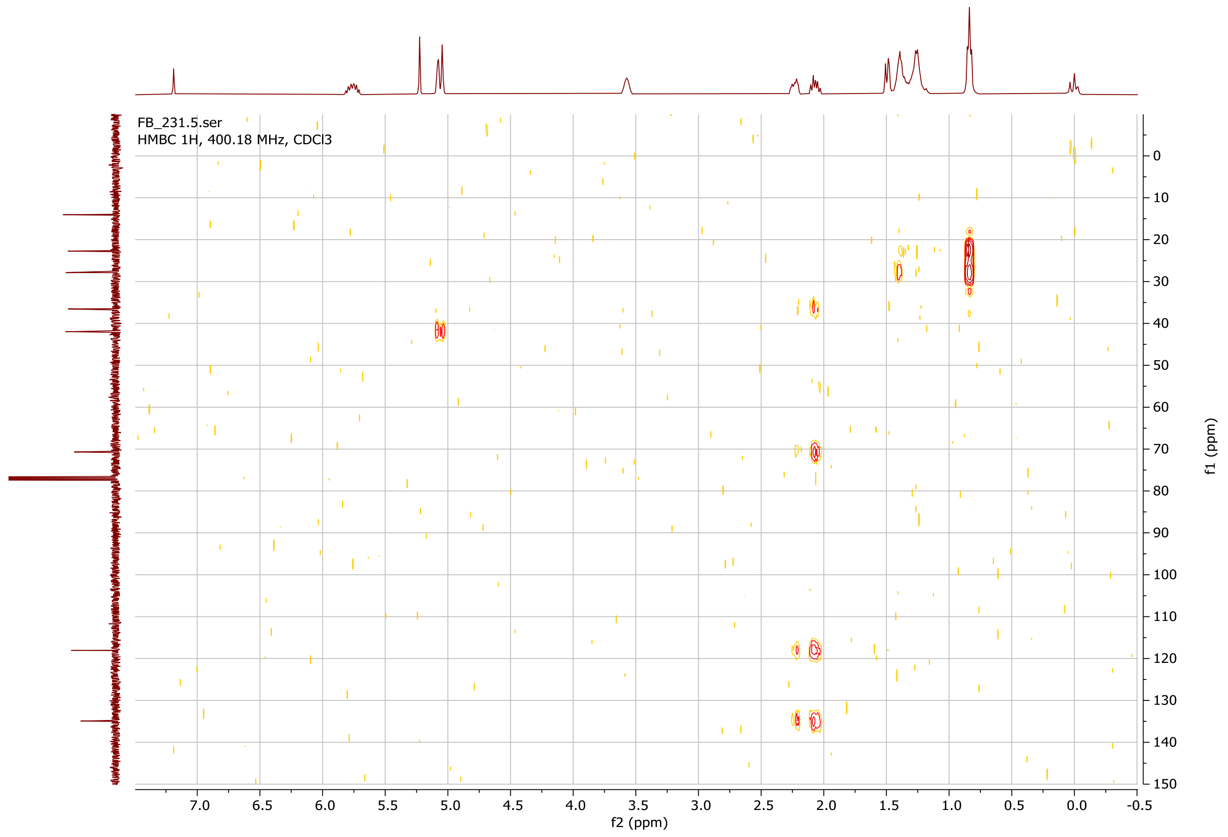
FB_231.5.ser
HMBC 1H, 400.18 MHz, CDCl3
f2 (ppm)
f1 (ppm)

1-Phenylhex-5-en-3-ol **6l**

FB_228.1.fid
1D 1H, 400.18 MHz, CDCl3

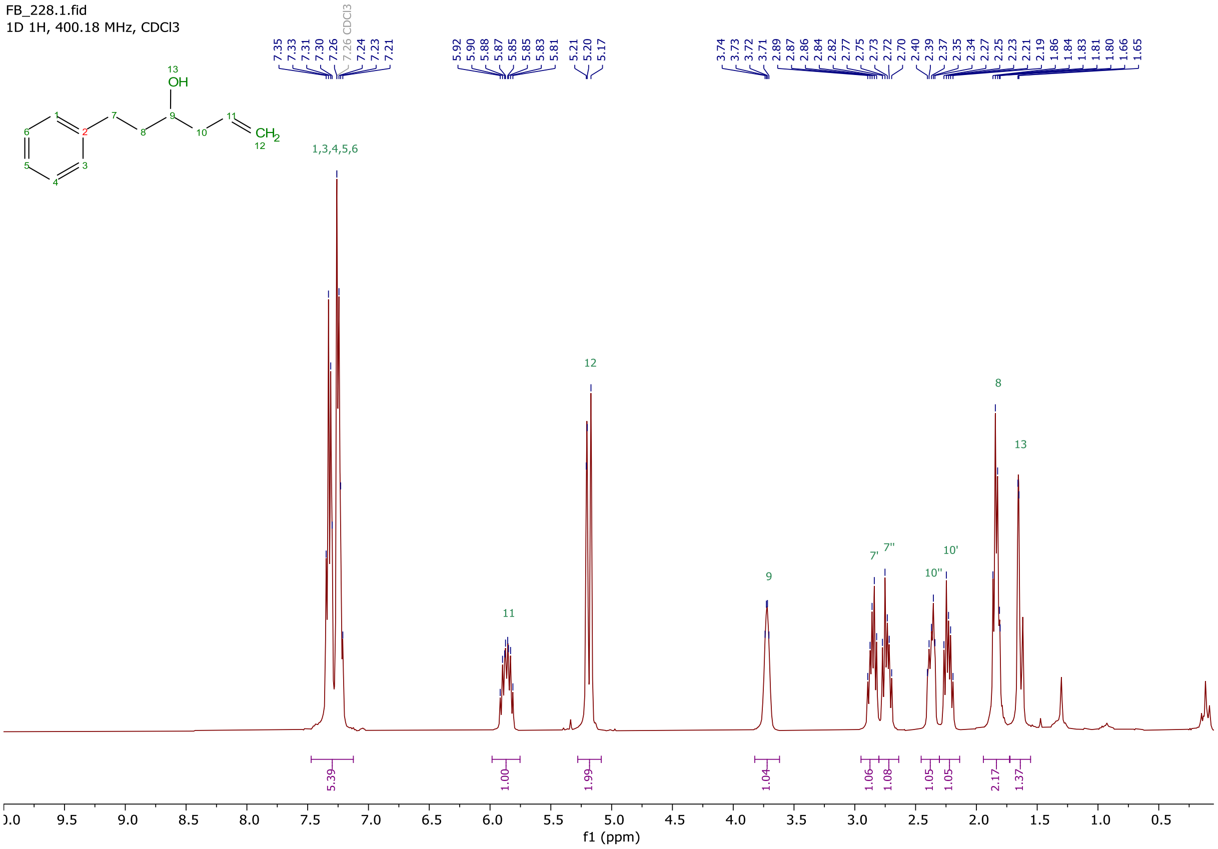

FB_228.3.fid
1D 13C{1H}, 100.64 MHz, CDCl3

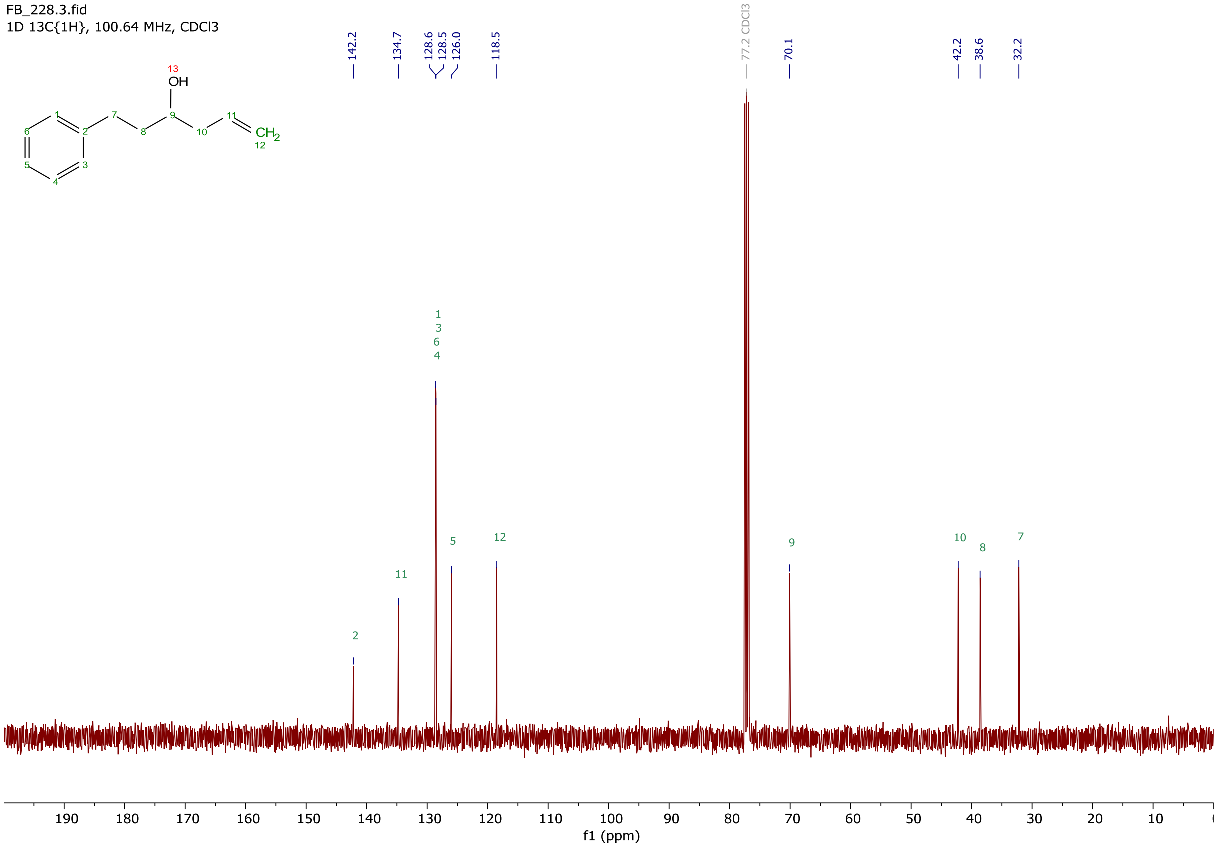

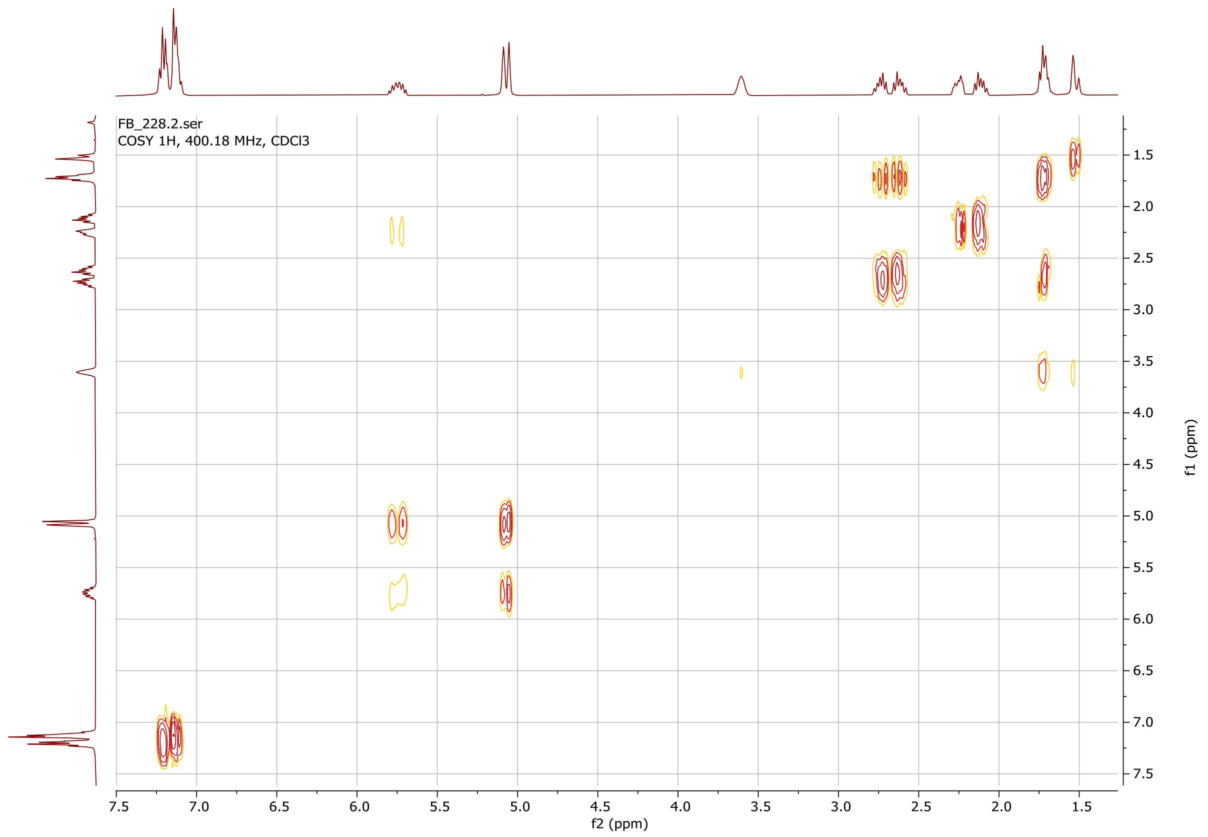
FB_228.2.ser
COSY 1H, 400.18 MHz, CDCl3
f2 (ppm)
f1 (ppm)

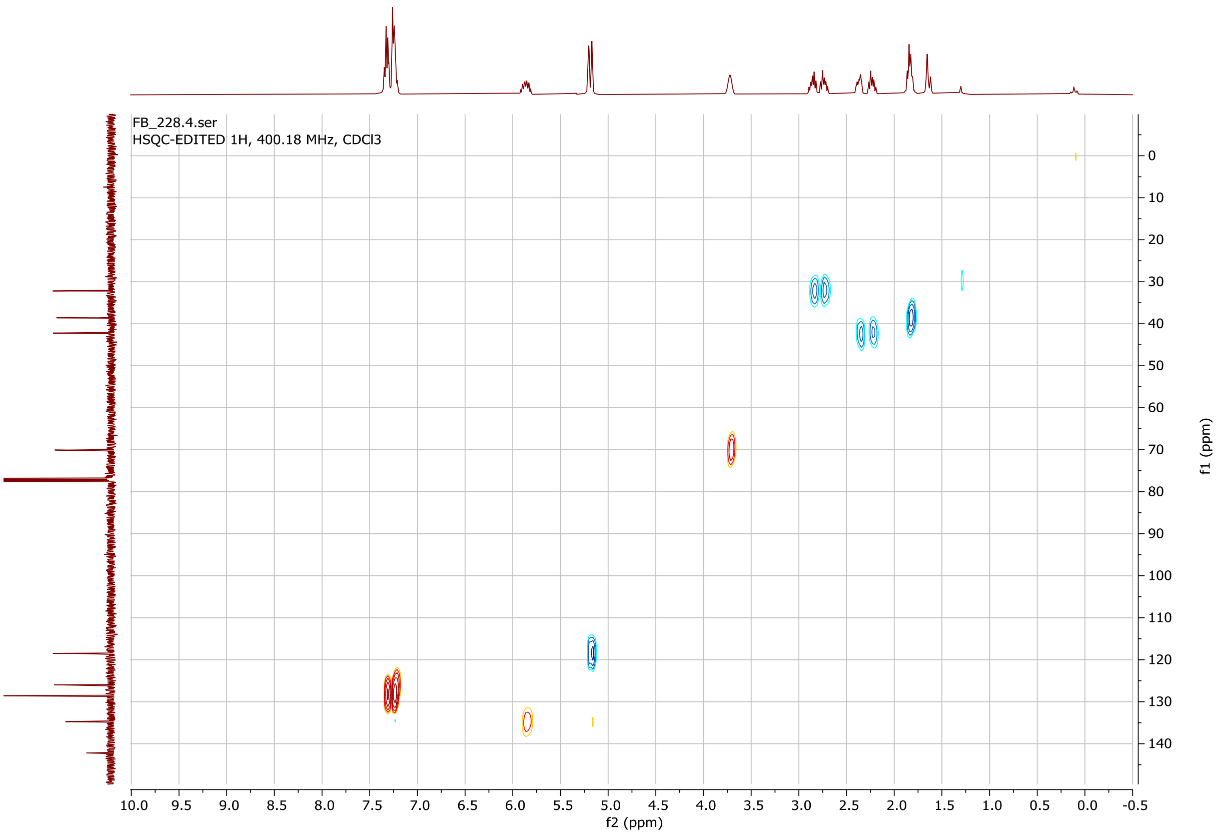
FB_228.4.ser
HSQC-EDITED 1H, 400.18 MHz, CDCl3
f2 (ppm)
f1 (ppm)

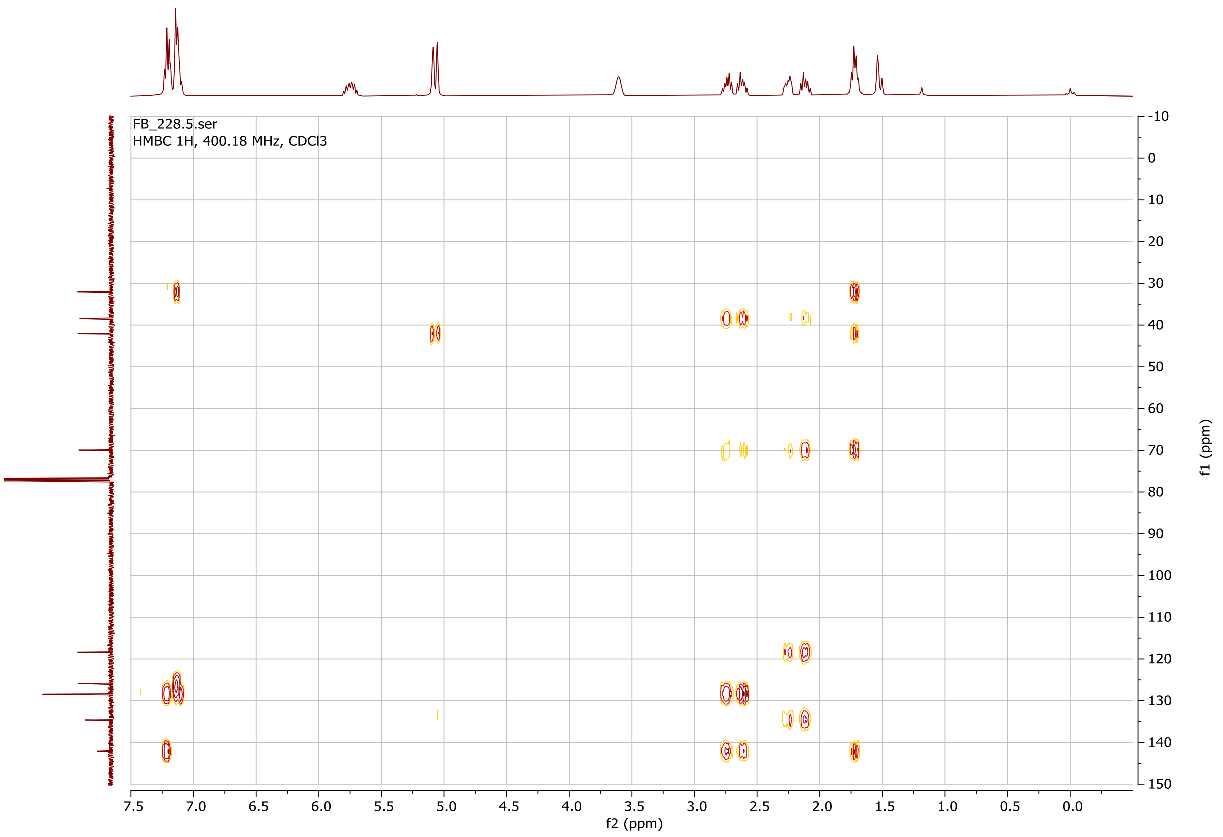

Methyl 4-hydroxyhept-6-enoate **6m**

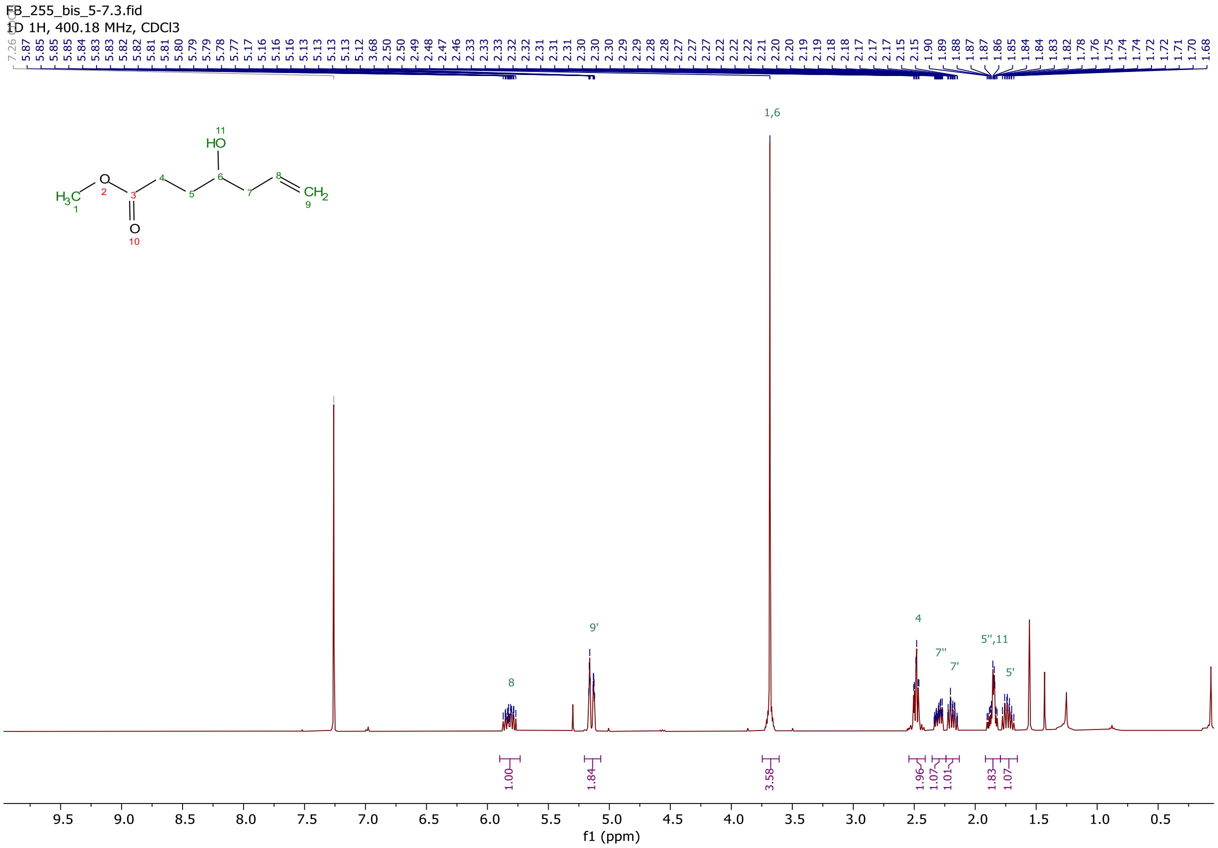

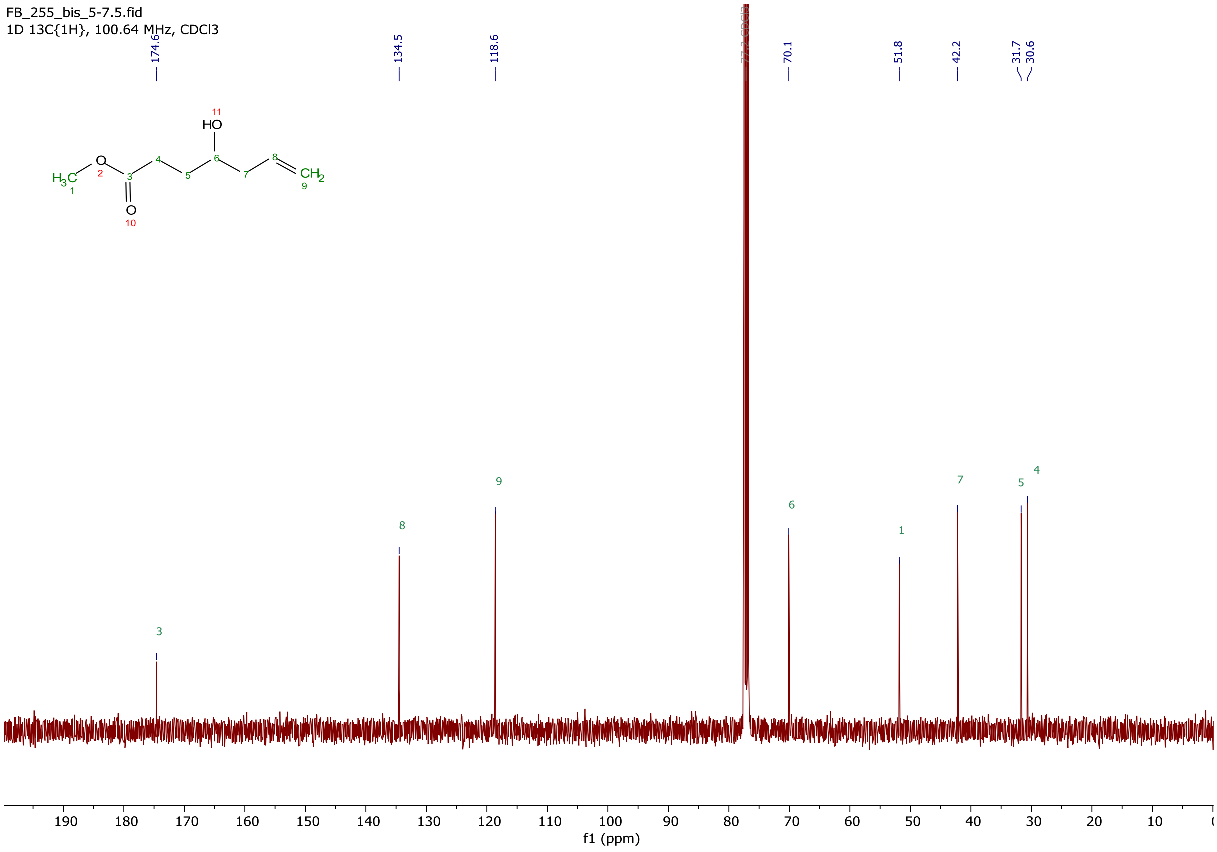

FB_255_bis_5-7.5.fid
1D 13C{1H}, 100.64 MHz, CDCl3
174.6
134.5
118.6
70.1
51.8
42.2
31.7
30.6
HO
O
O
3
8
9
6
1
7
5
4
f1 (ppm)

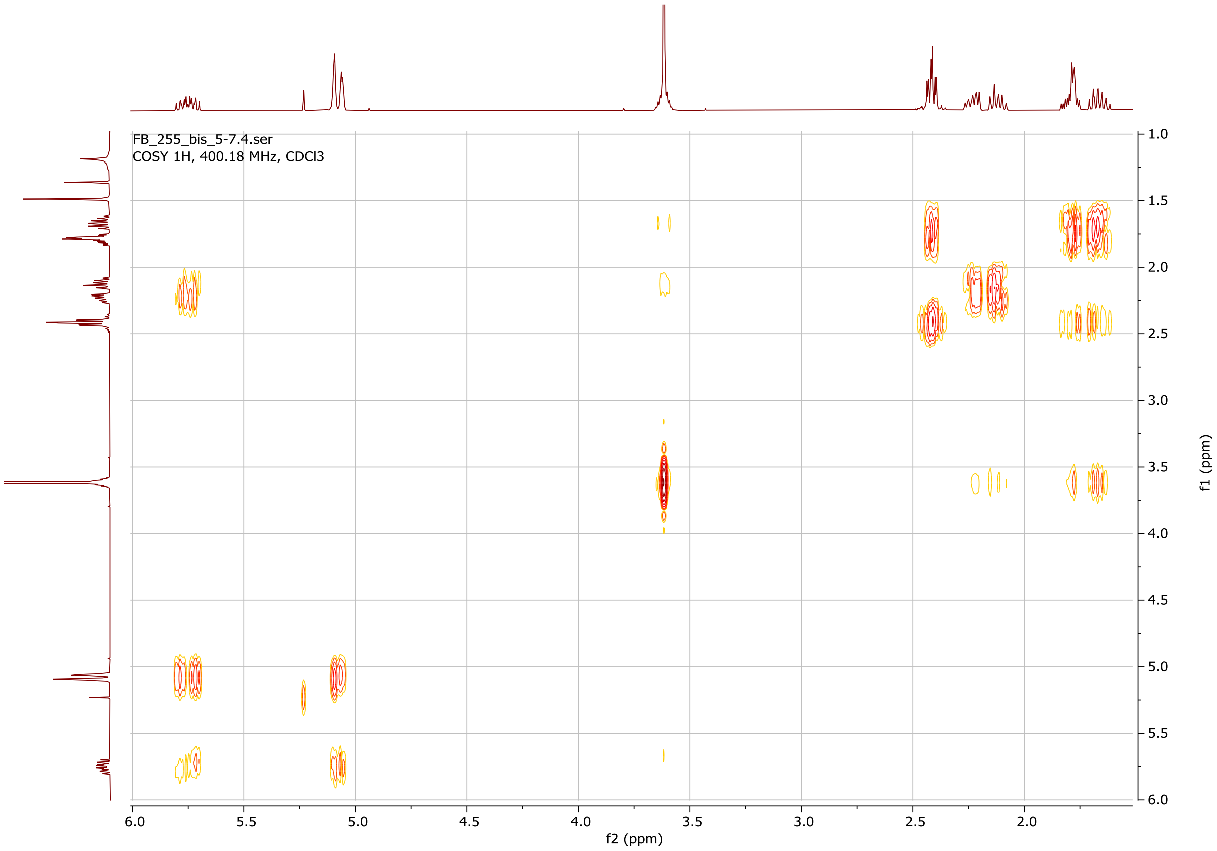

FB_255_bis_5-7.4.ser
COSY 1H, 400.18 MHz, CDCl3
f2 (ppm)
f1 (ppm)

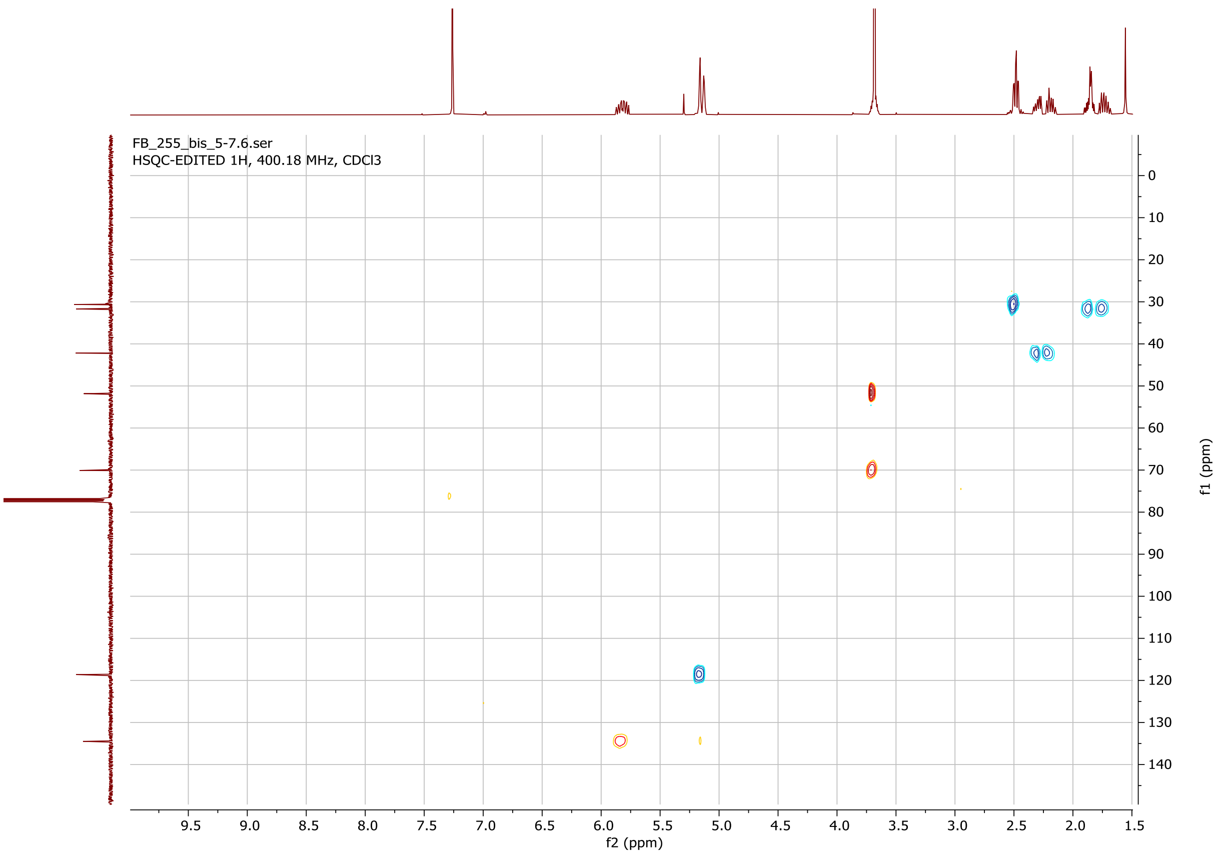
FB_255_bis_5-7.6.ser
HSQC-EDITED 1H, 400.18 MHz, CDCl3
f2 (ppm)
f1 (ppm)

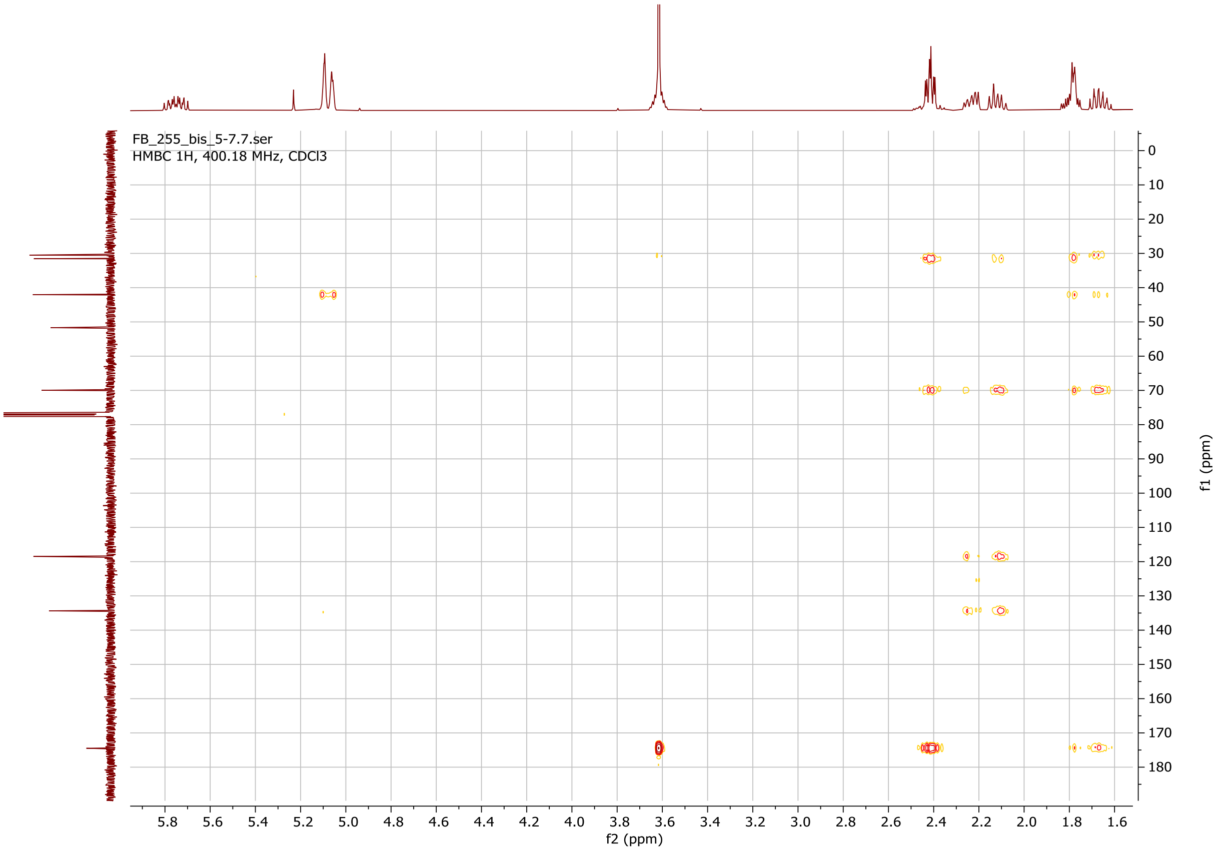
FB_255_bis_5-7.7.ser
HMBC 1H, 400.18 MHz, CDCl3
f2 (ppm)
f1 (ppm)

1-Phenylbut-3-en-1-ol **6n**

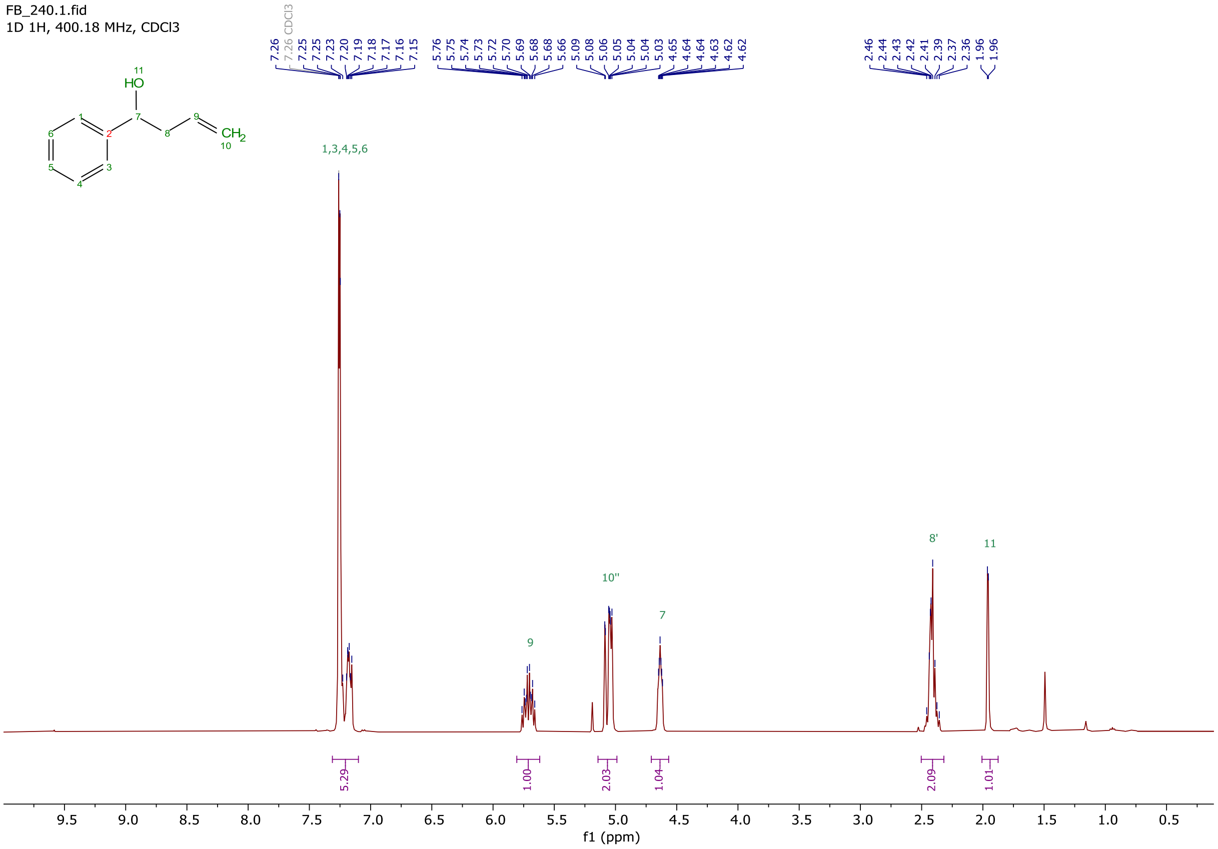

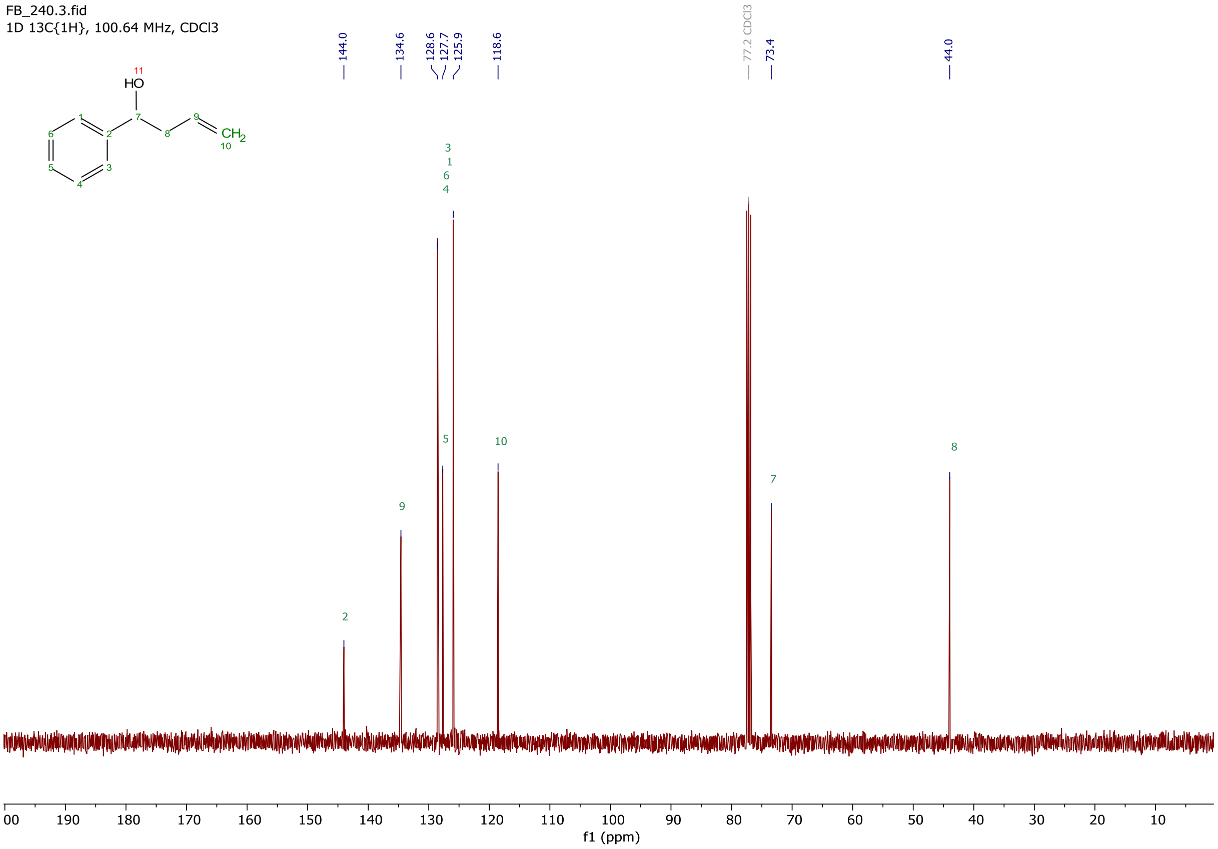

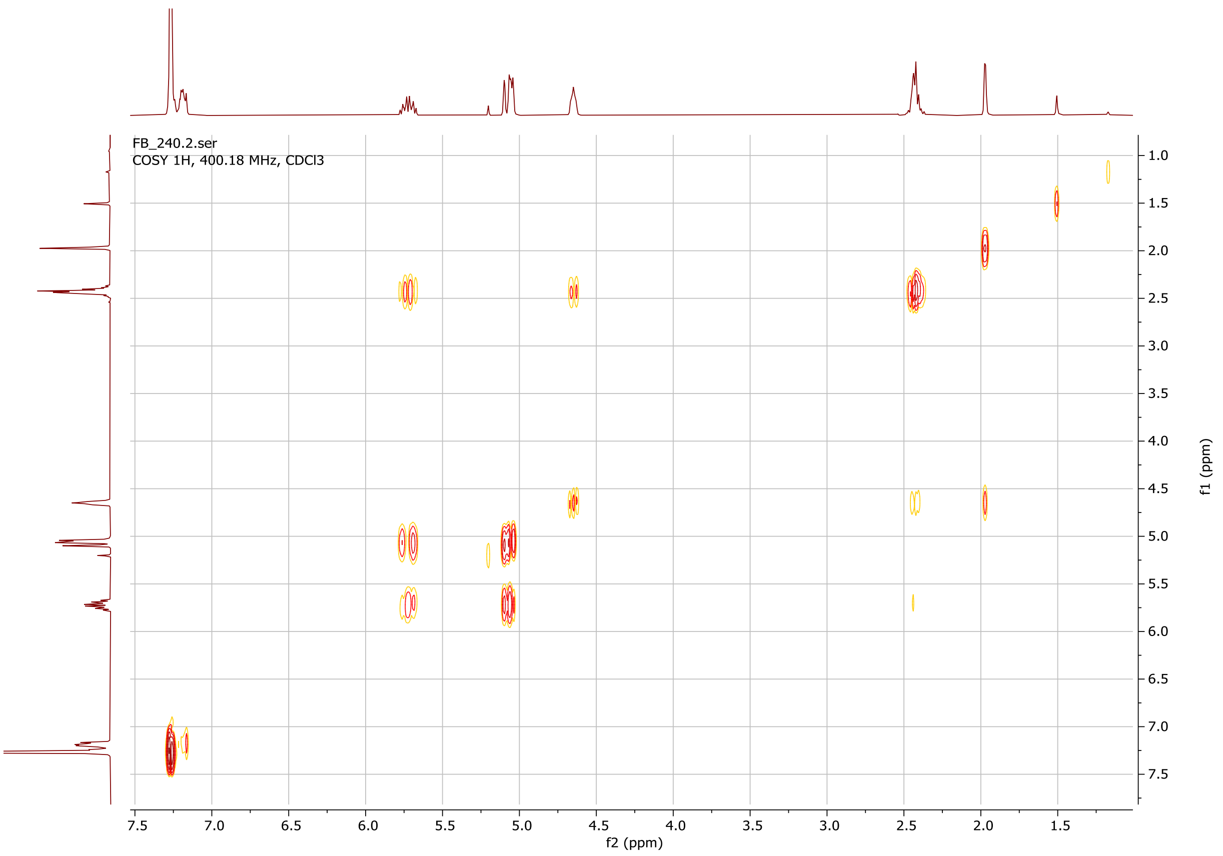
FB_240.2.ser
COSY 1H, 400.18 MHz, CDCl3
f2 (ppm)
f1 (ppm)

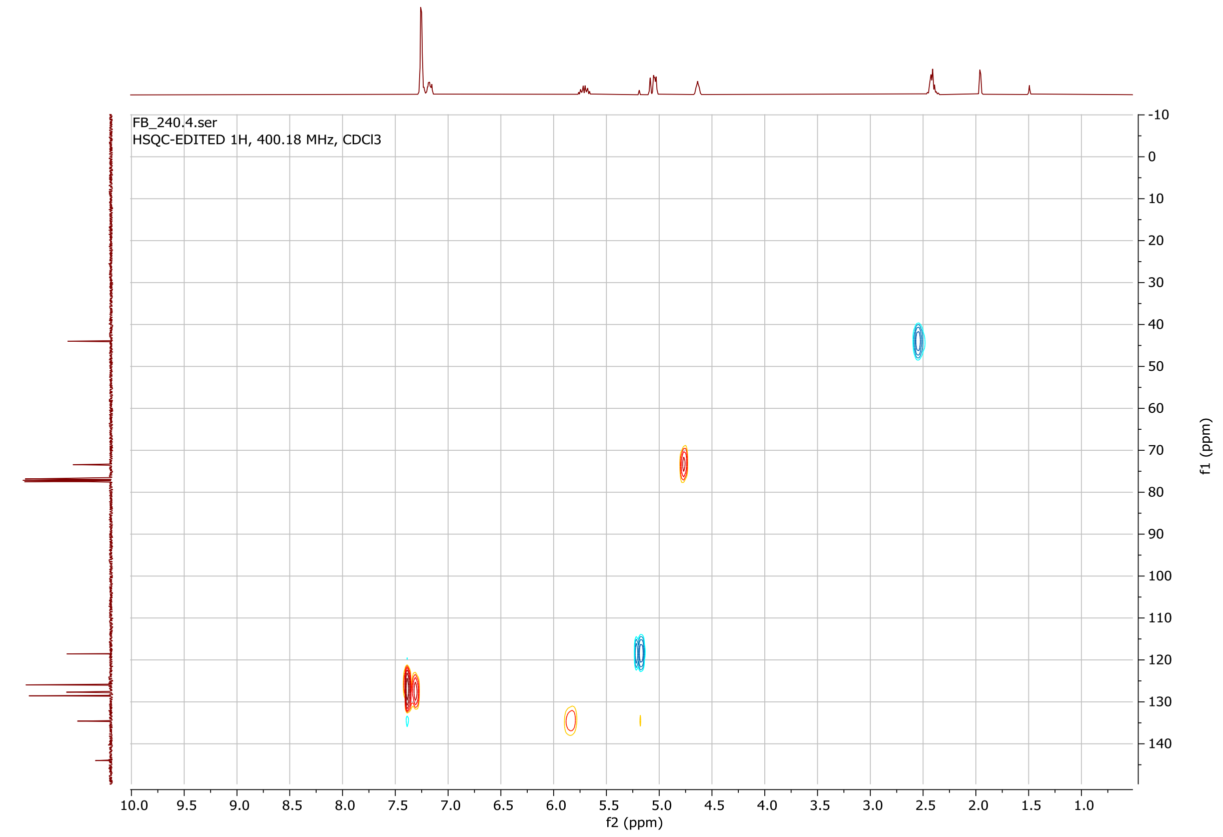
FB_240.4.ser
HSQC-EDITED 1H, 400.18 MHz, CDCl3
f2 (ppm)
f1 (ppm)

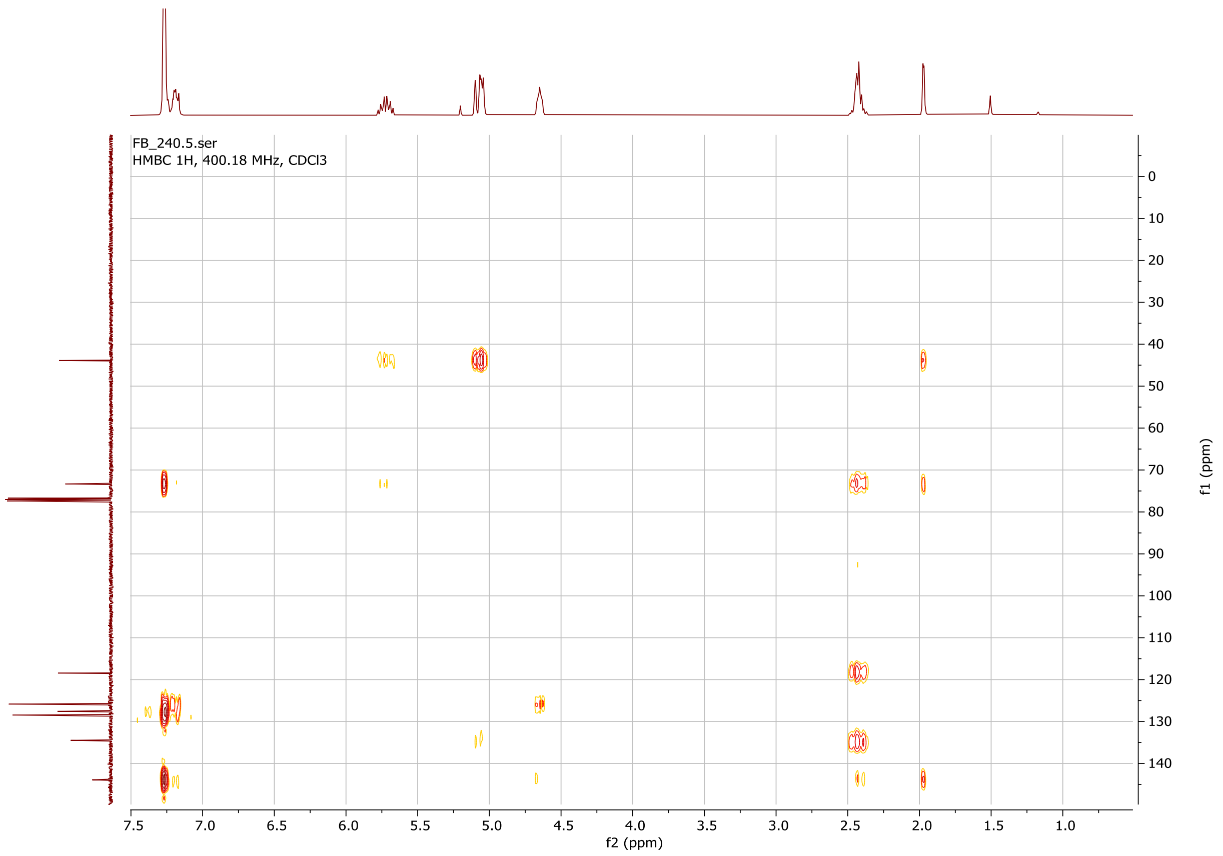

1-(4-Methoxyphenyl)but-3-en-1-ol **6o**

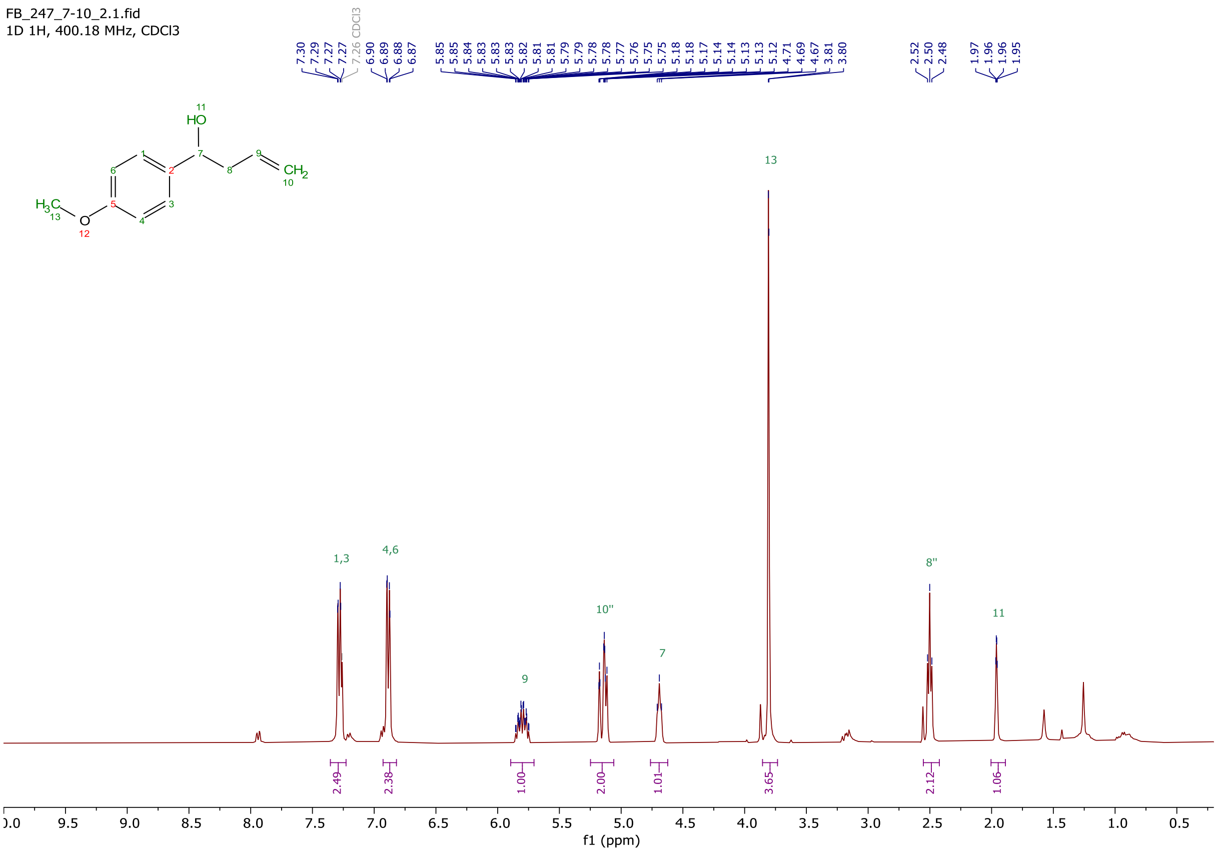

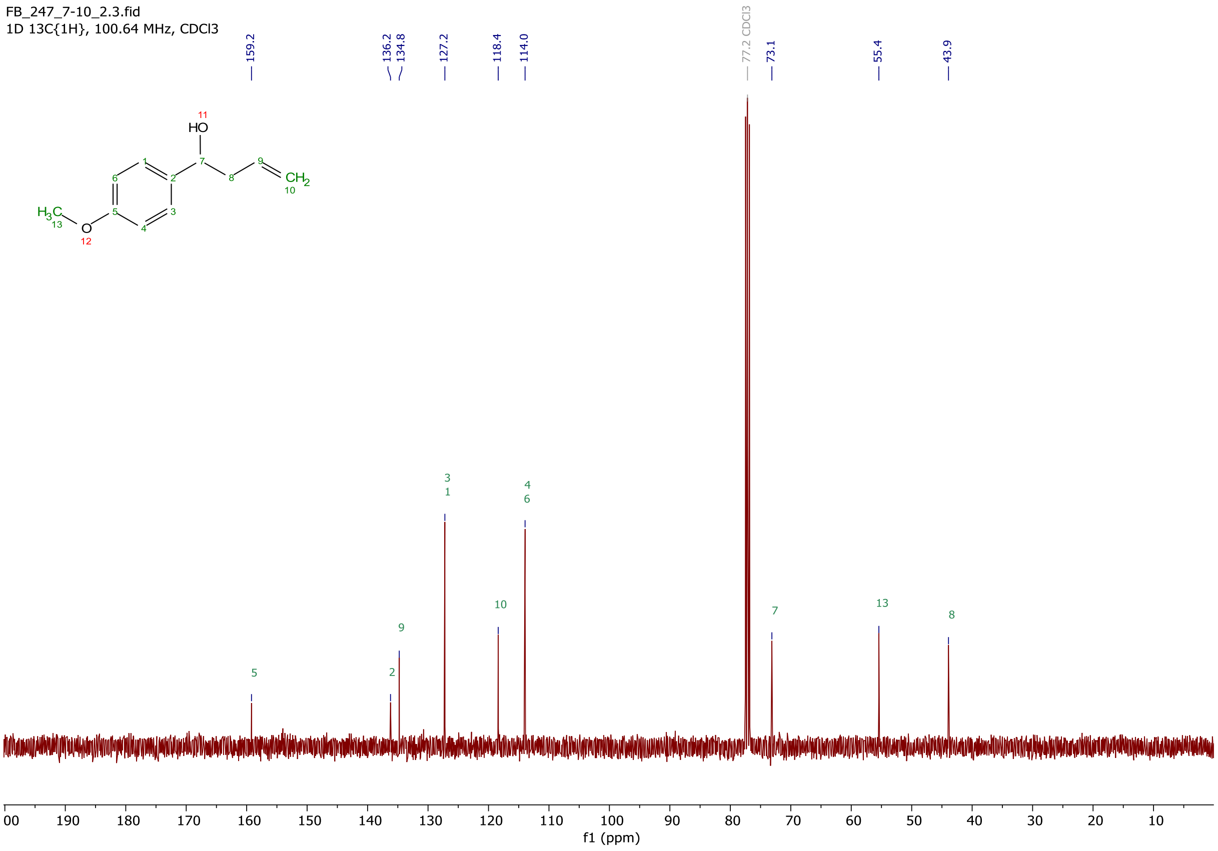
FB_247_7-10_2.3.fid
1D 13C{1H}, 100.64 MHz, CDCl3
159.2
136.2
134.8
127.2
118.4
114.0
77.2 CDCl3
73.1
55.4
43.9
f1 (ppm)

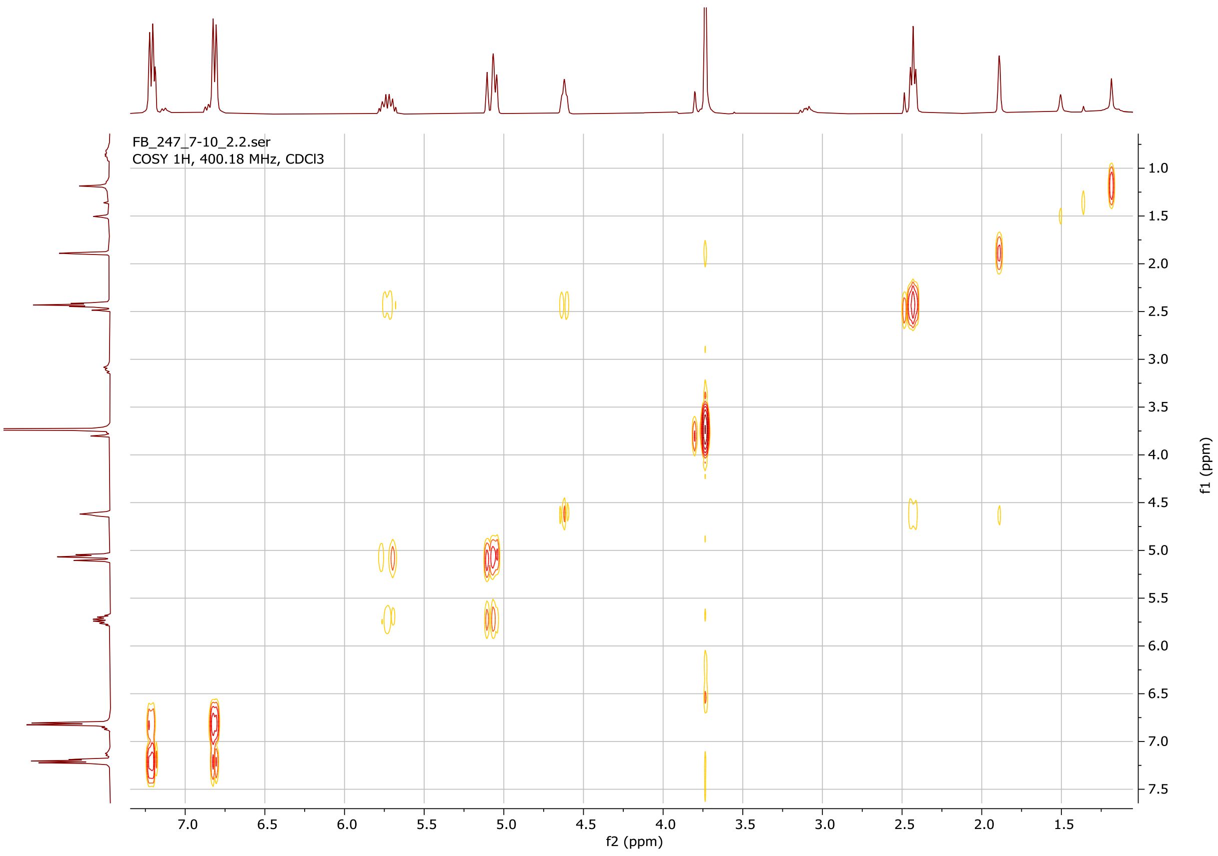
FB_247_7-10_2.2.ser
COSY 1H, 400.18 MHz, CDCl3
f2 (ppm)
f1 (ppm)

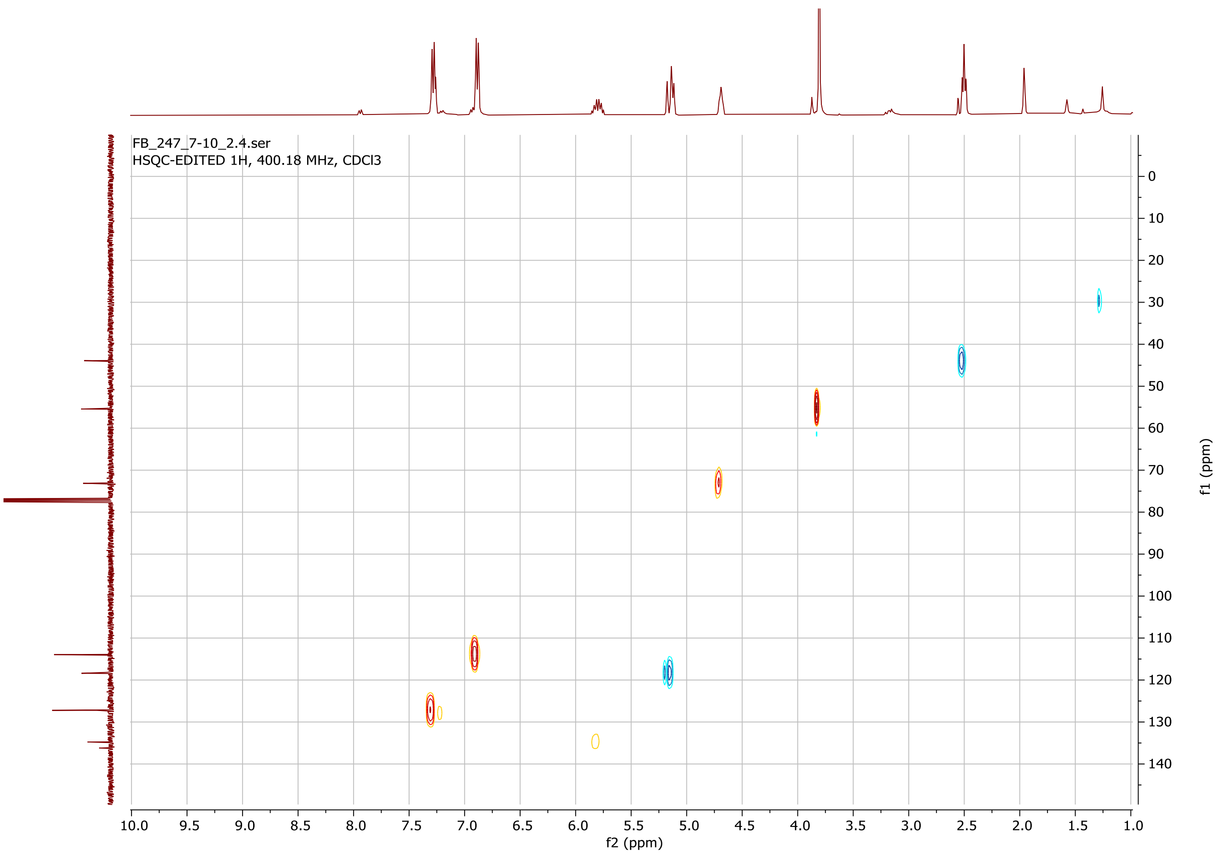

FB_247_7-10_2.4.ser
HSQC-EDITED 1H, 400.18 MHz, CDCl3
f2 (ppm)
f1 (ppm)

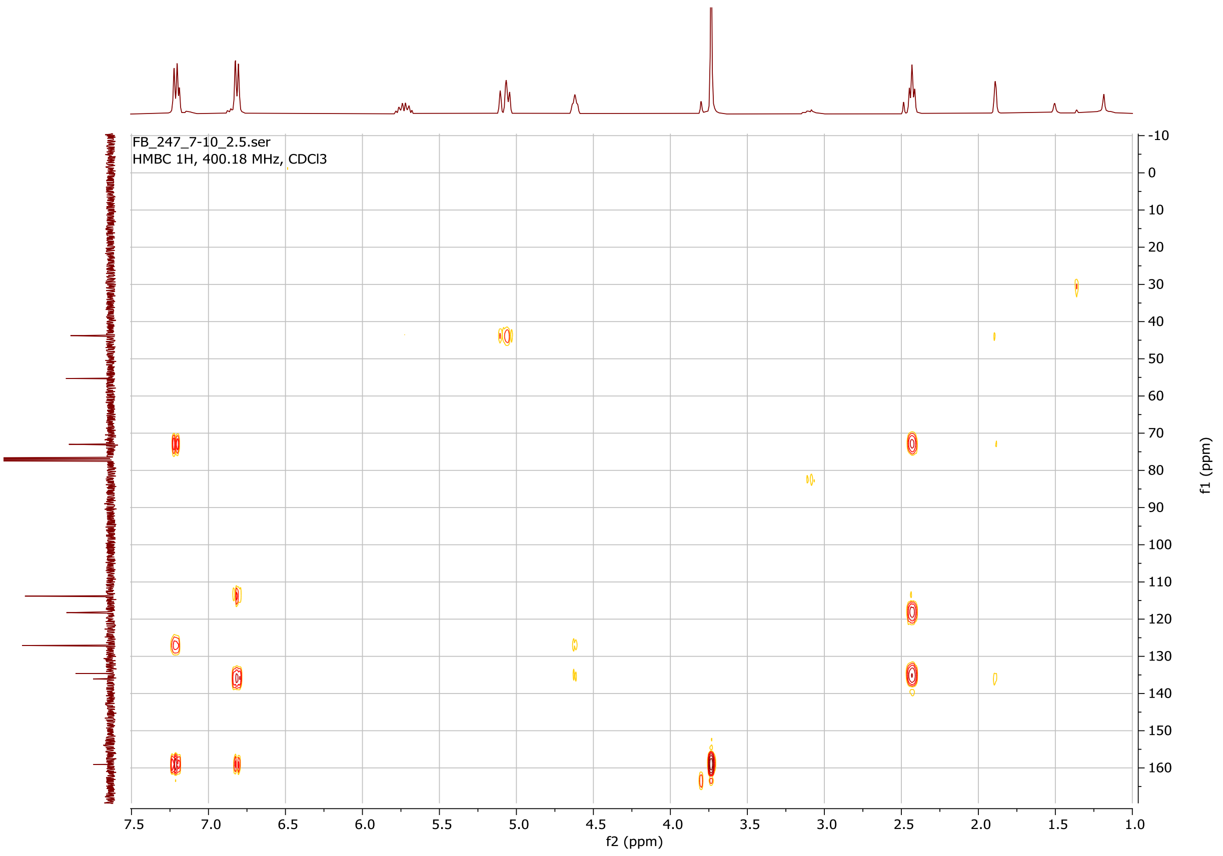

FB_247_7-10_2.5.ser
HMBC 1H, 400.18 MHz, CDCl3
f2 (ppm)
f1 (ppm)

1-(4-(Trifluoromethyl)phenyl)but-3-en-1-ol **6p**

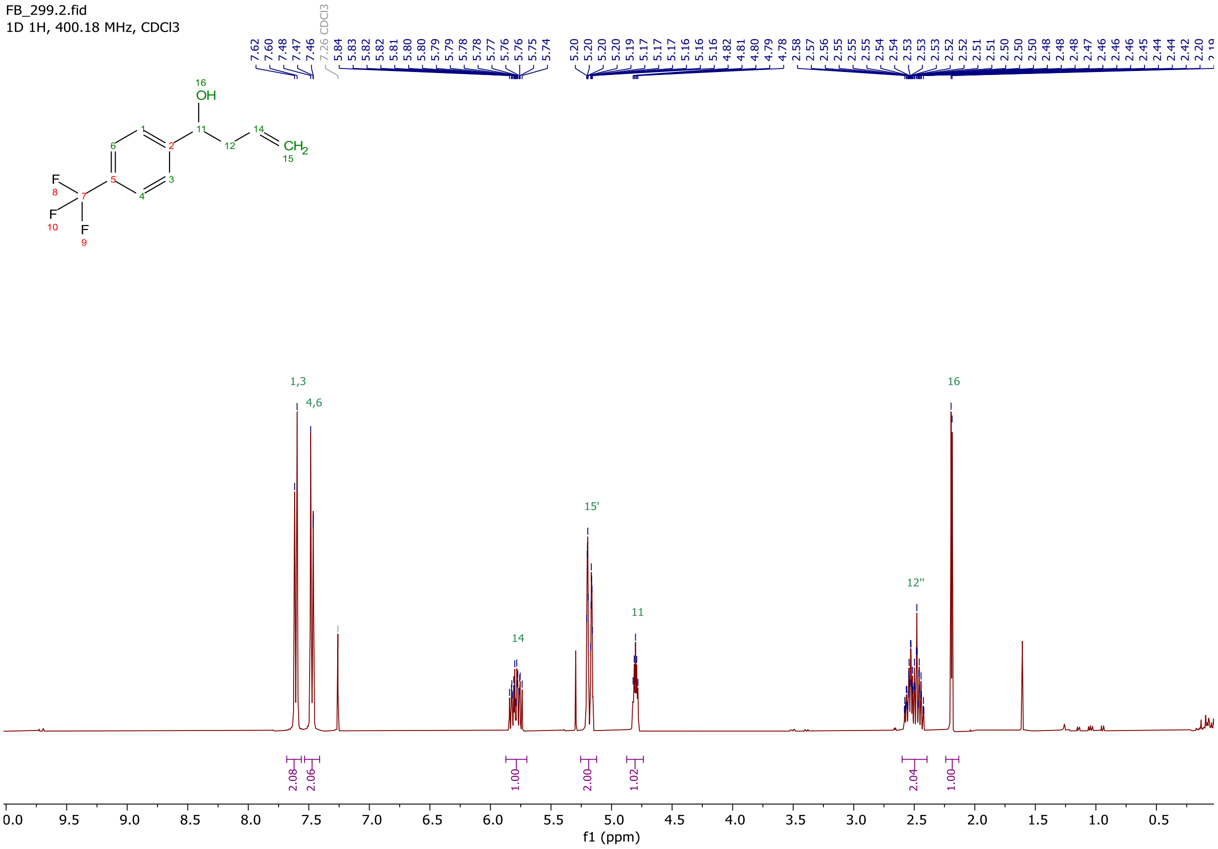

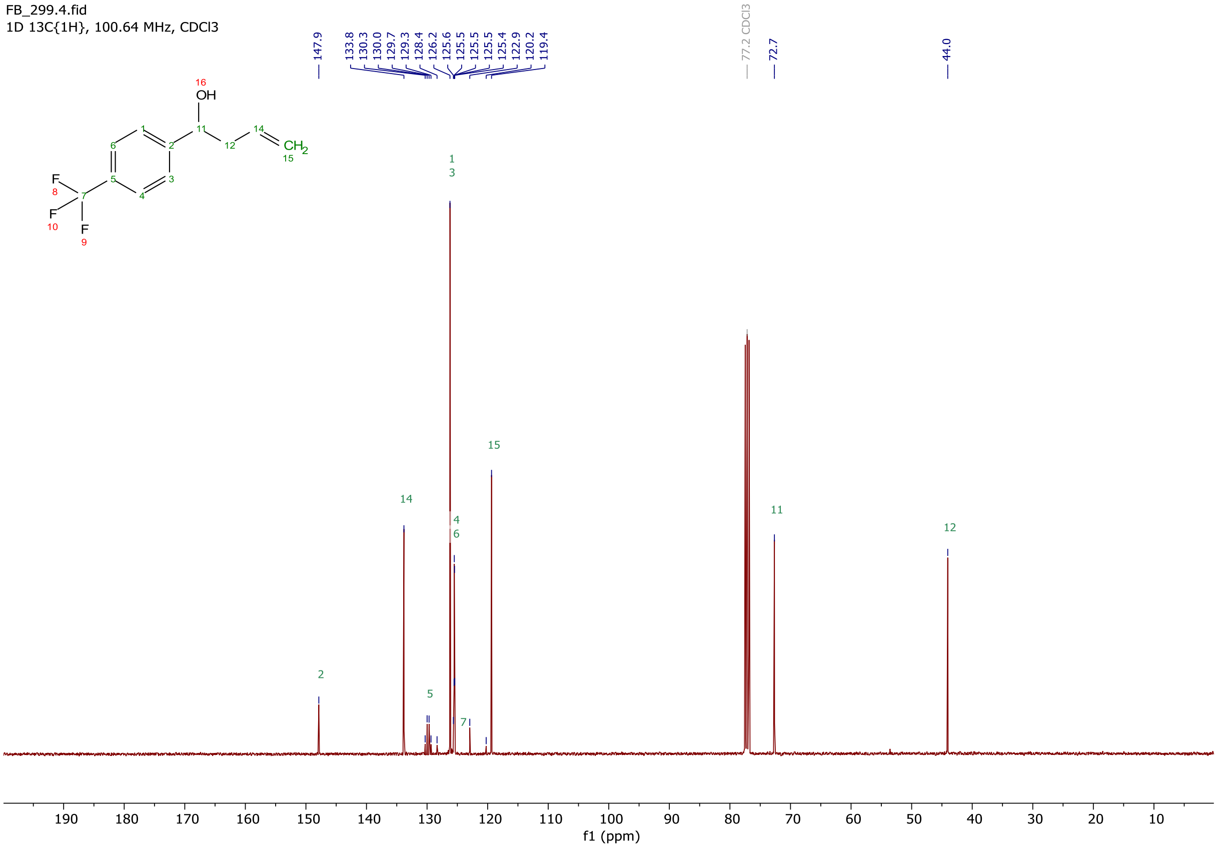

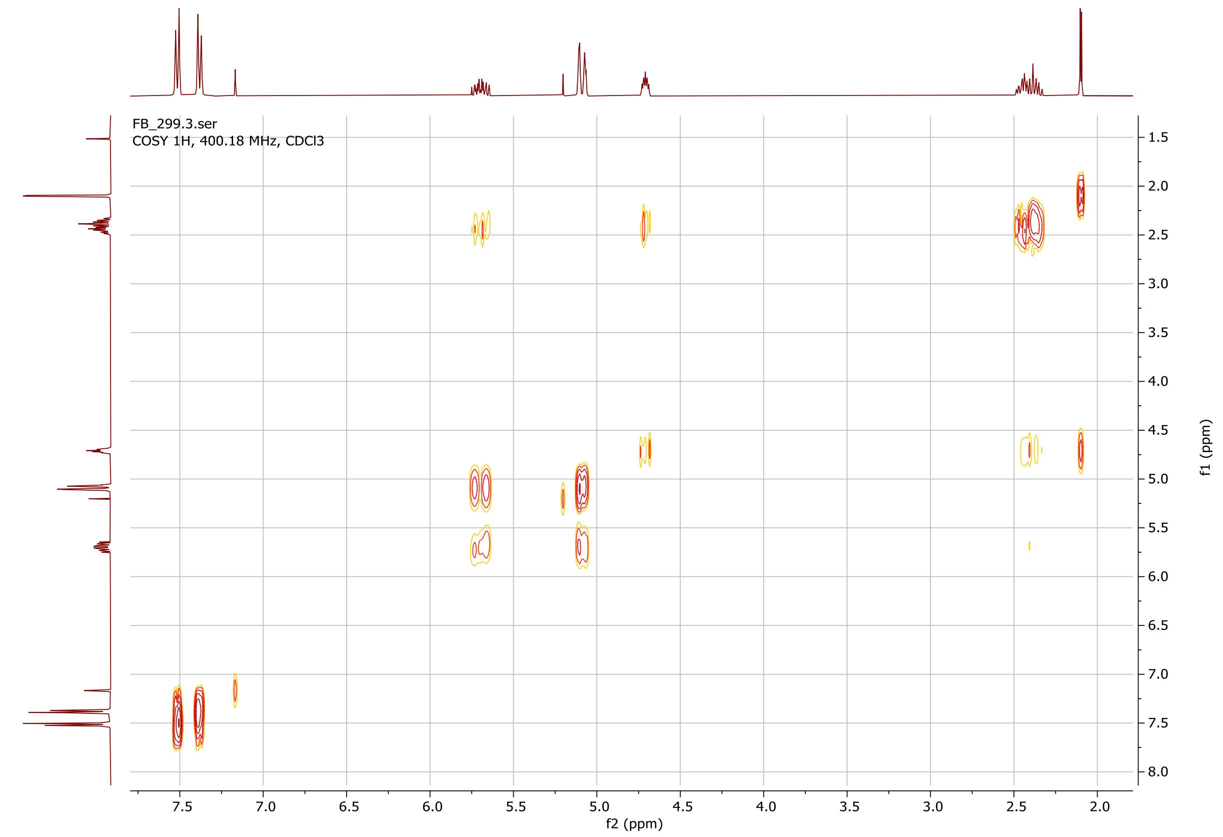

FB_299.3.ser
COSY 1H, 400.18 MHz, CDCl3
f2 (ppm)
f1 (ppm)

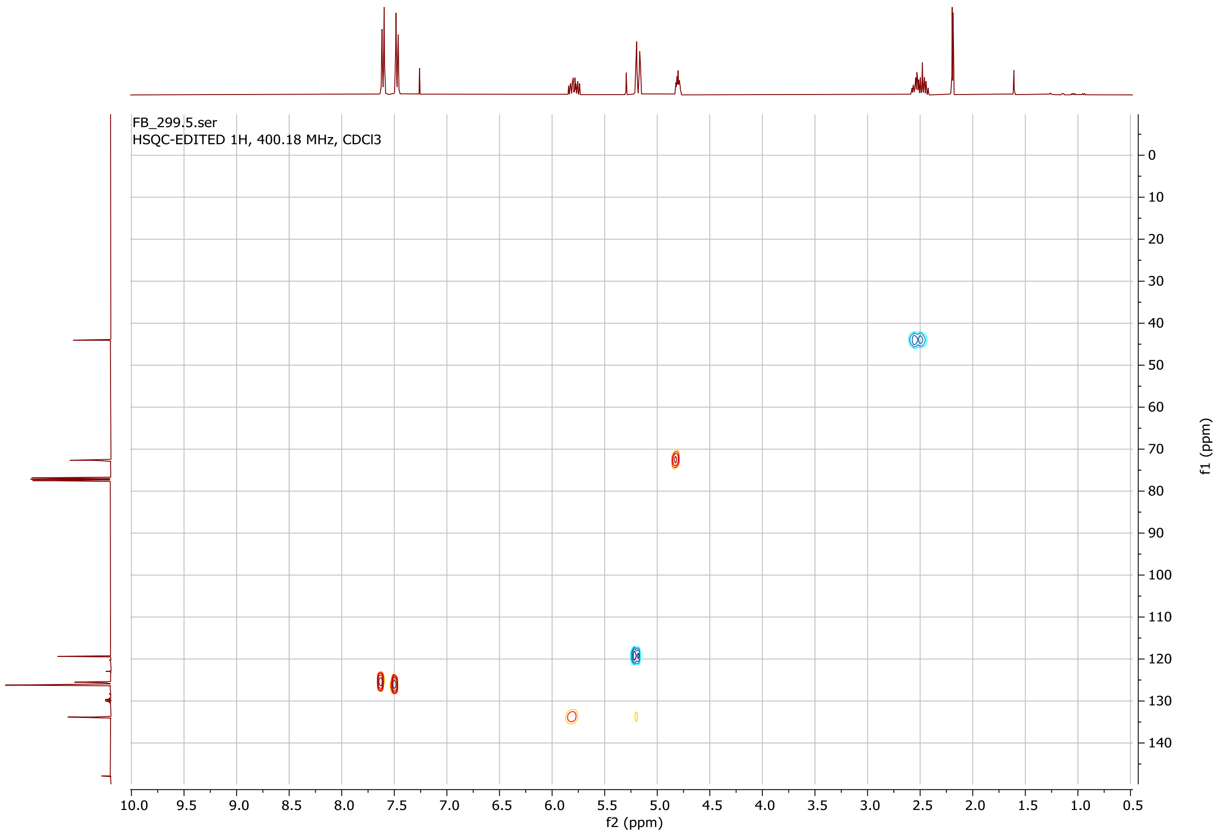

FB_299.5.ser
HSQC-EDITED 1H, 400.18 MHz, CDCl3
f2 (ppm)
f1 (ppm)

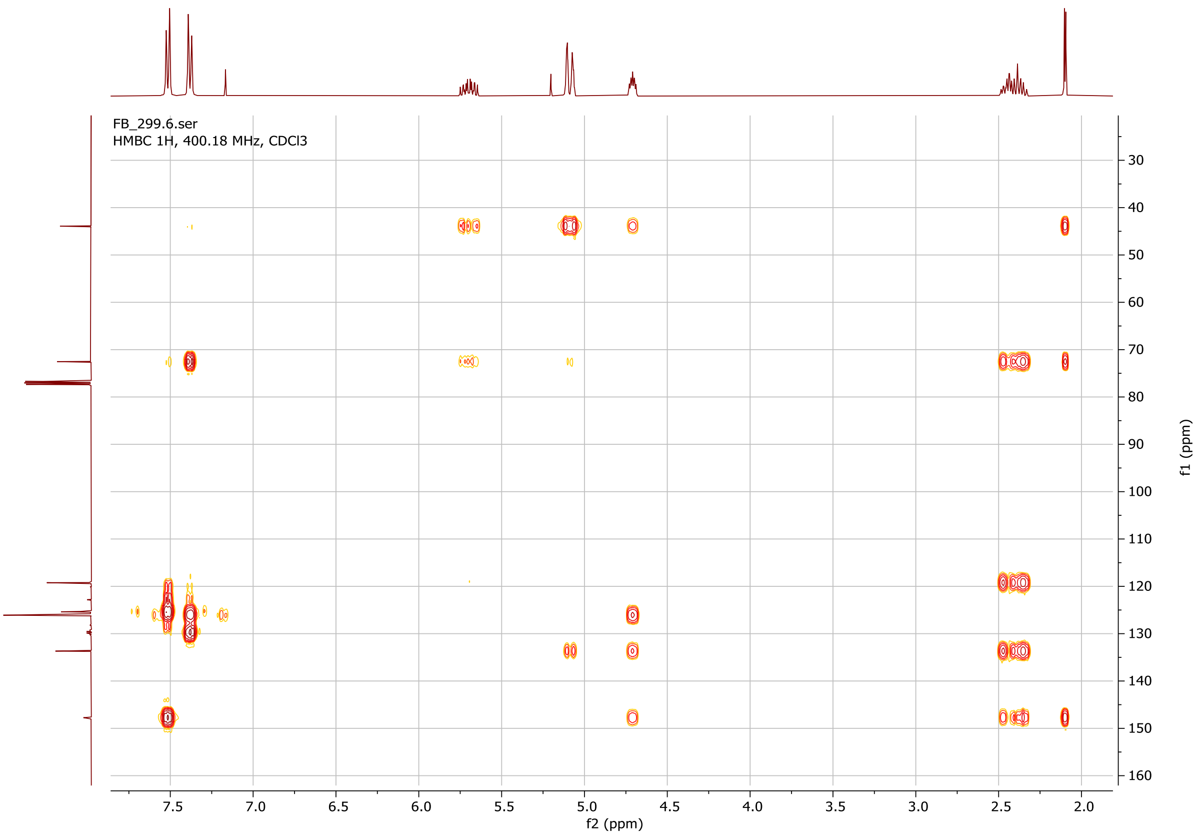

3-Phenylpent-4-en-2-ol **6q**

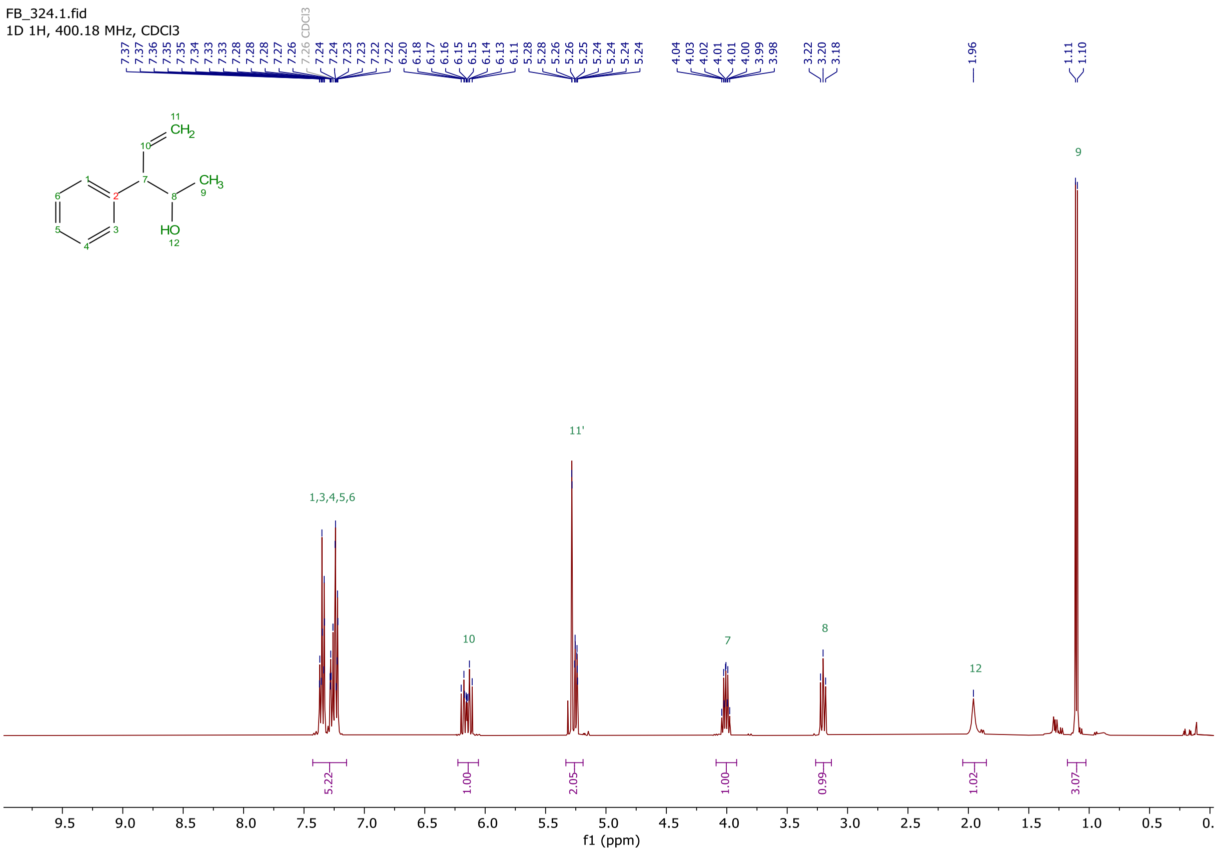

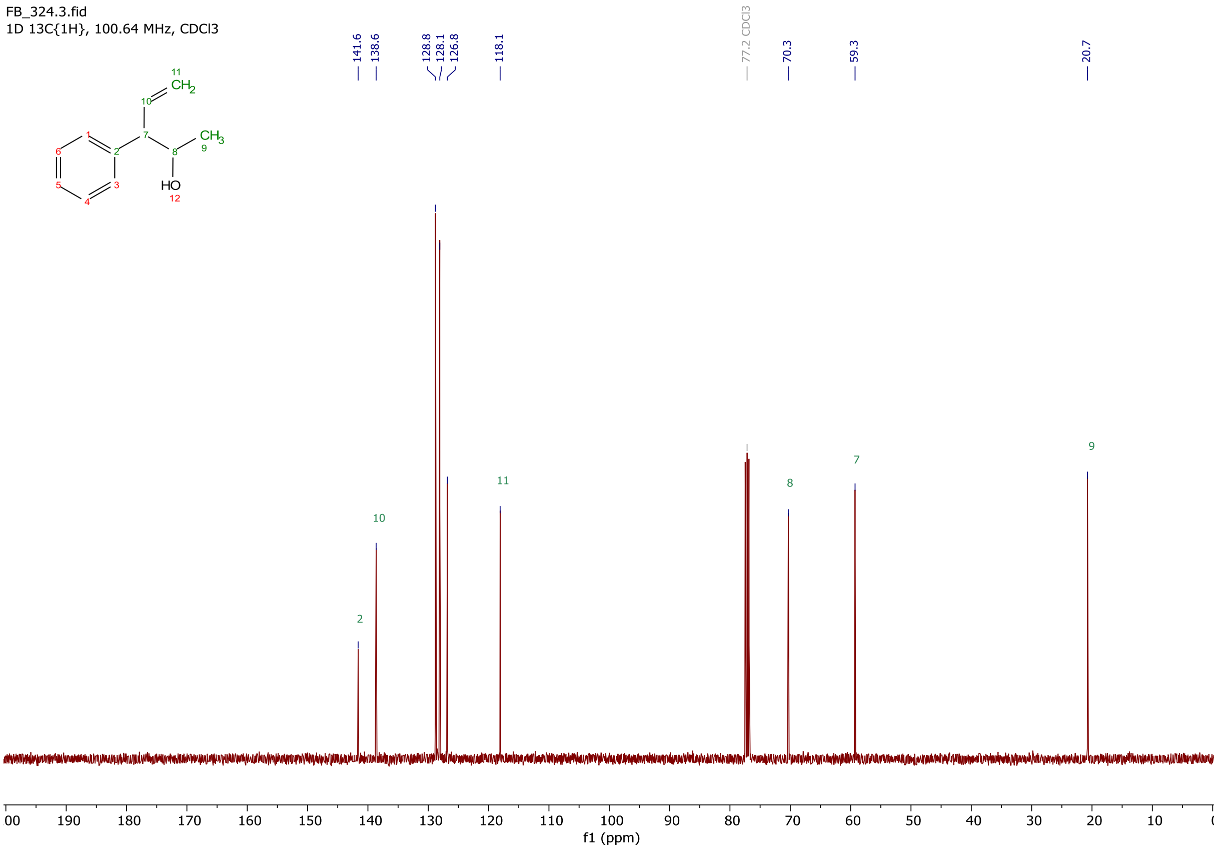

FB_324.3.fid
1D 13C{1H}, 100.64 MHz, CDCl3
141.6
138.6
128.8
128.1
126.8
118.1
77.2 CDCl3
70.3
59.3
20.7
CH2
CH3
HO
f1 (ppm)

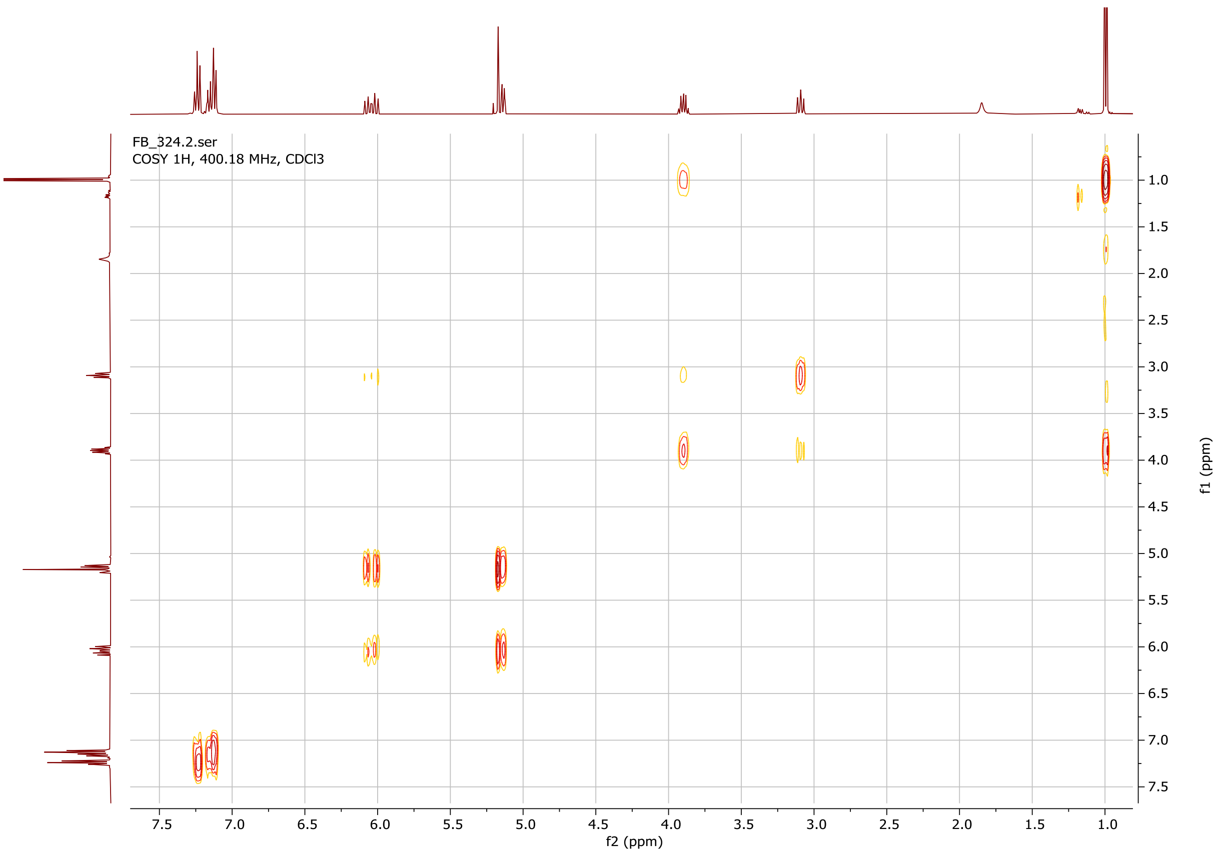

FB_324.2.ser
COSY 1H, 400.18 MHz, CDCl3
f2 (ppm)
f1 (ppm)

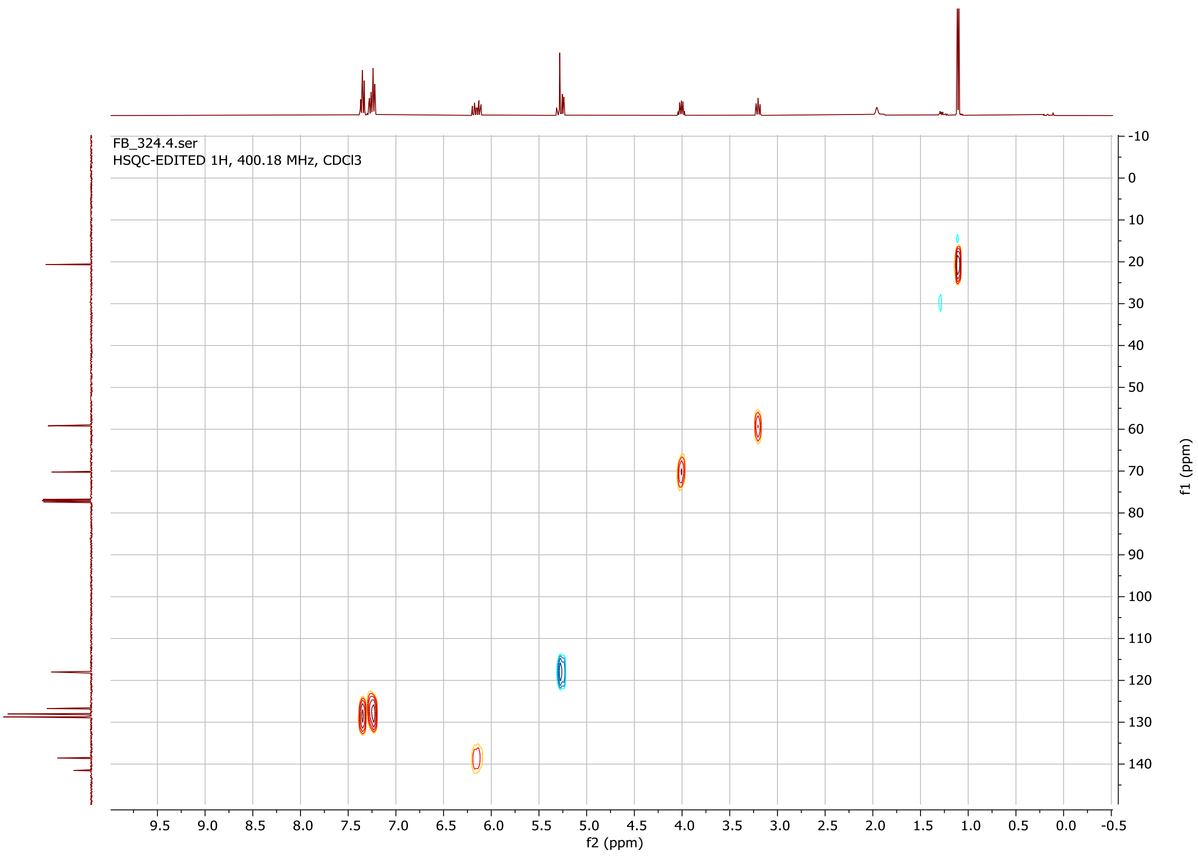
FB_324.4.ser
HSQC-EDITED 1H, 400.18 MHz, CDCl3
f2 (ppm)
f1 (ppm)

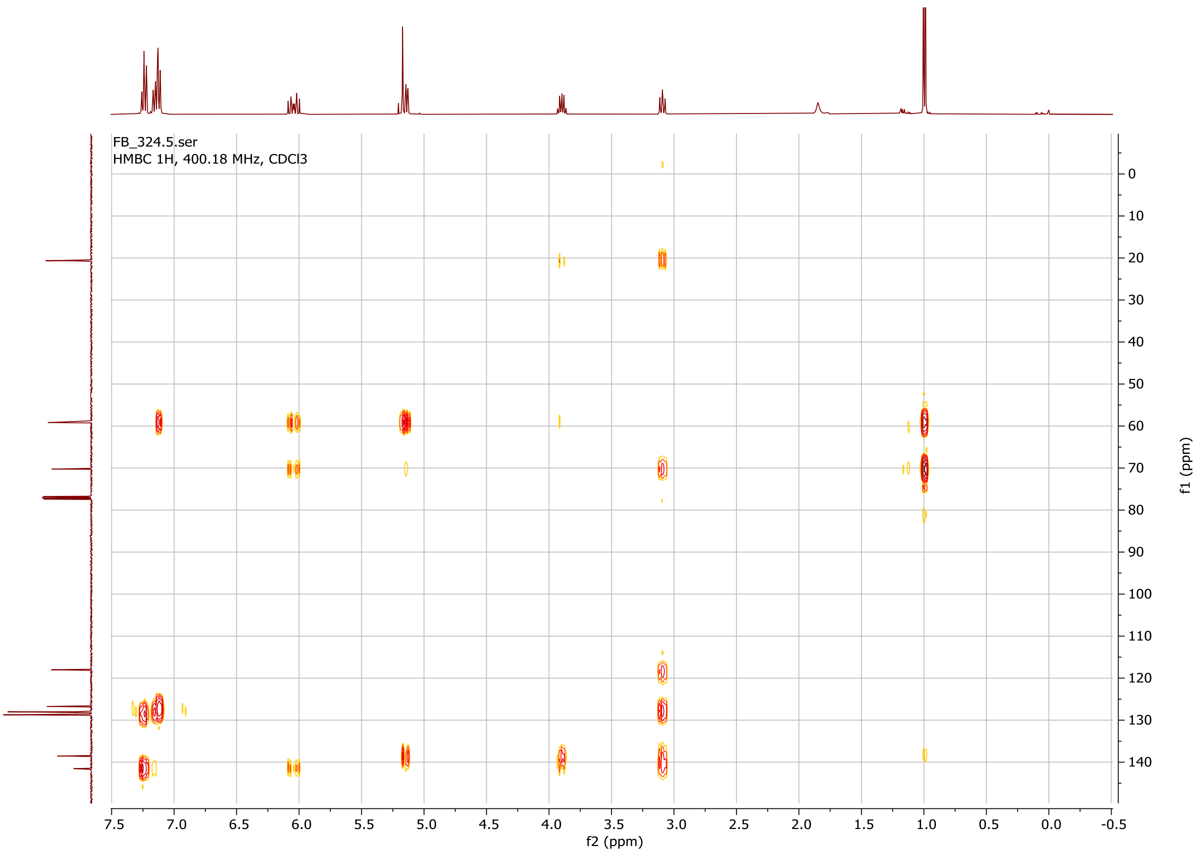
FB_324.5.ser
HMBC 1H, 400.18 MHz, CDCl3
f2 (ppm)
f1 (ppm)

3-Hydroxy-4-phenylhex-5-en-2-one **6r**

FB_340.1.fid
1D 1H, 400.18 MHz, CDCl3

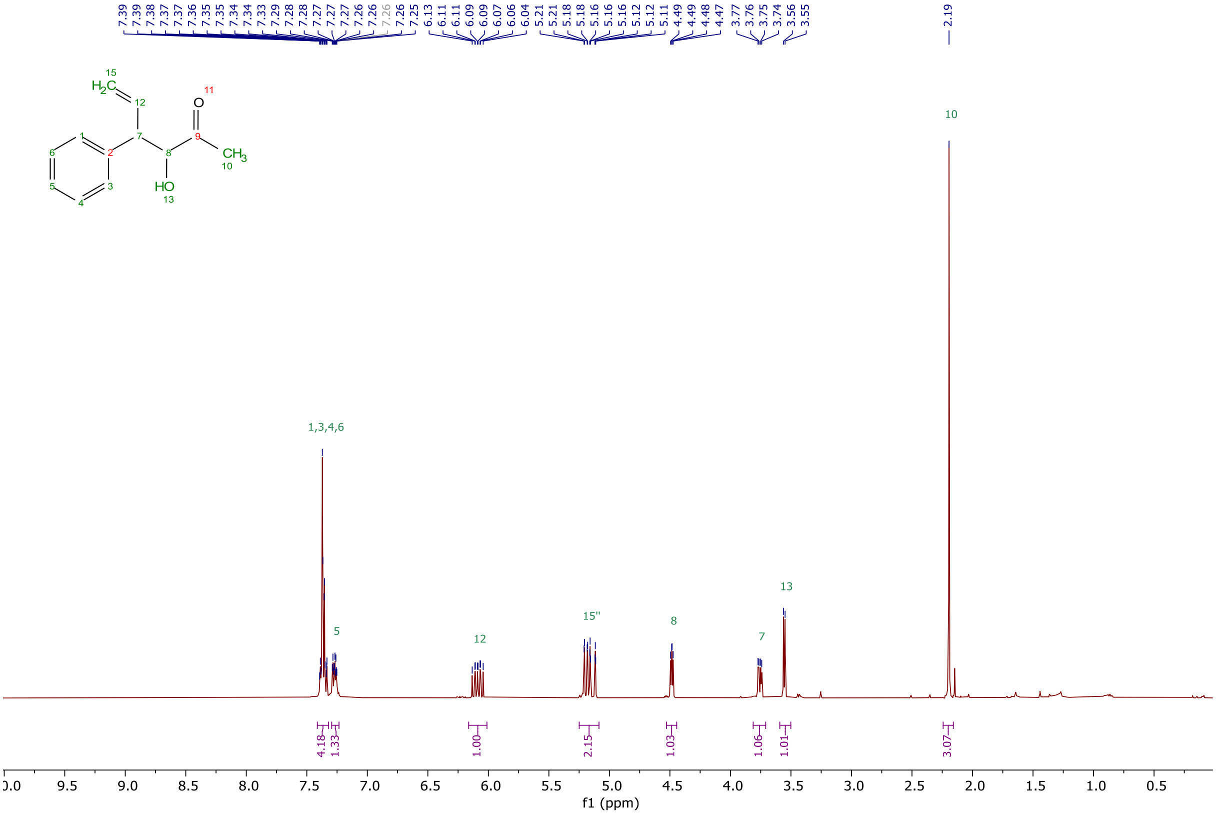

FB_340.3.fid
1D 13C{1H}, 100.64 MHz, CDCl3

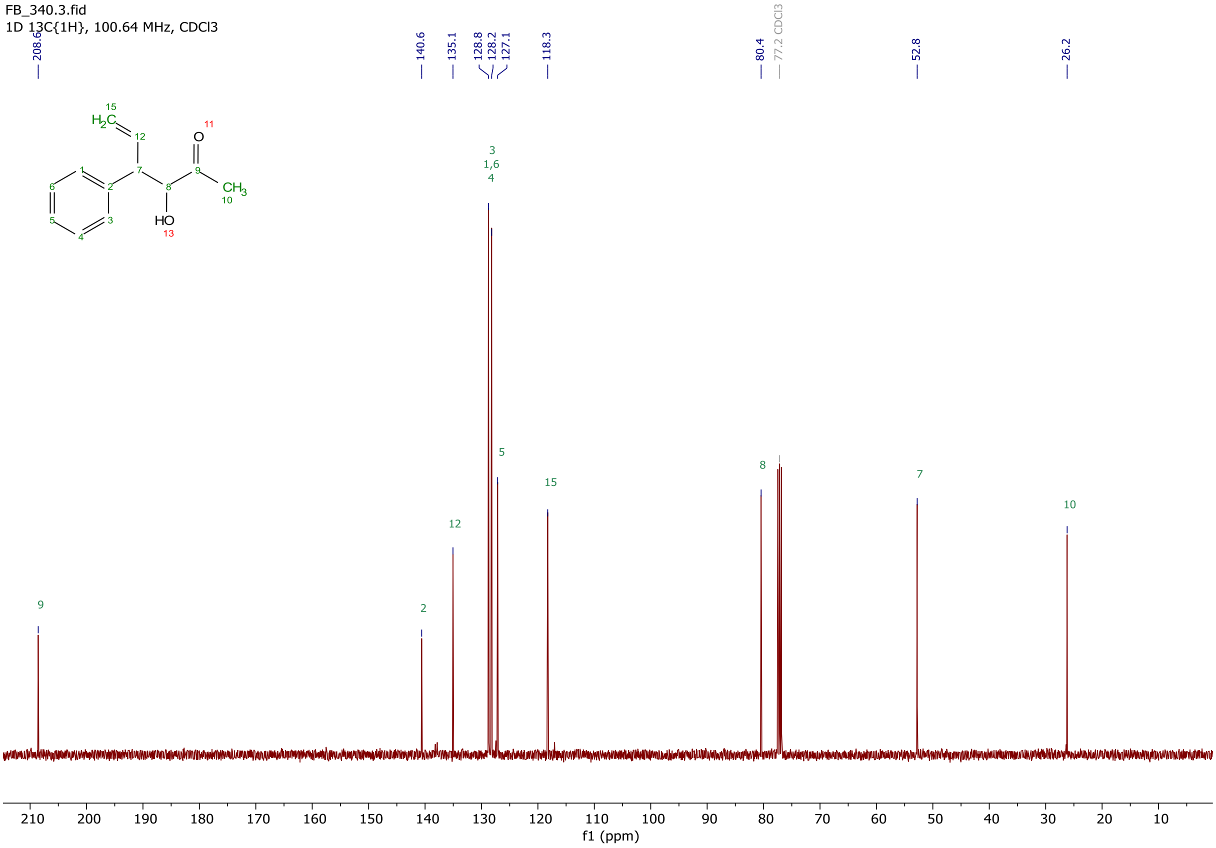

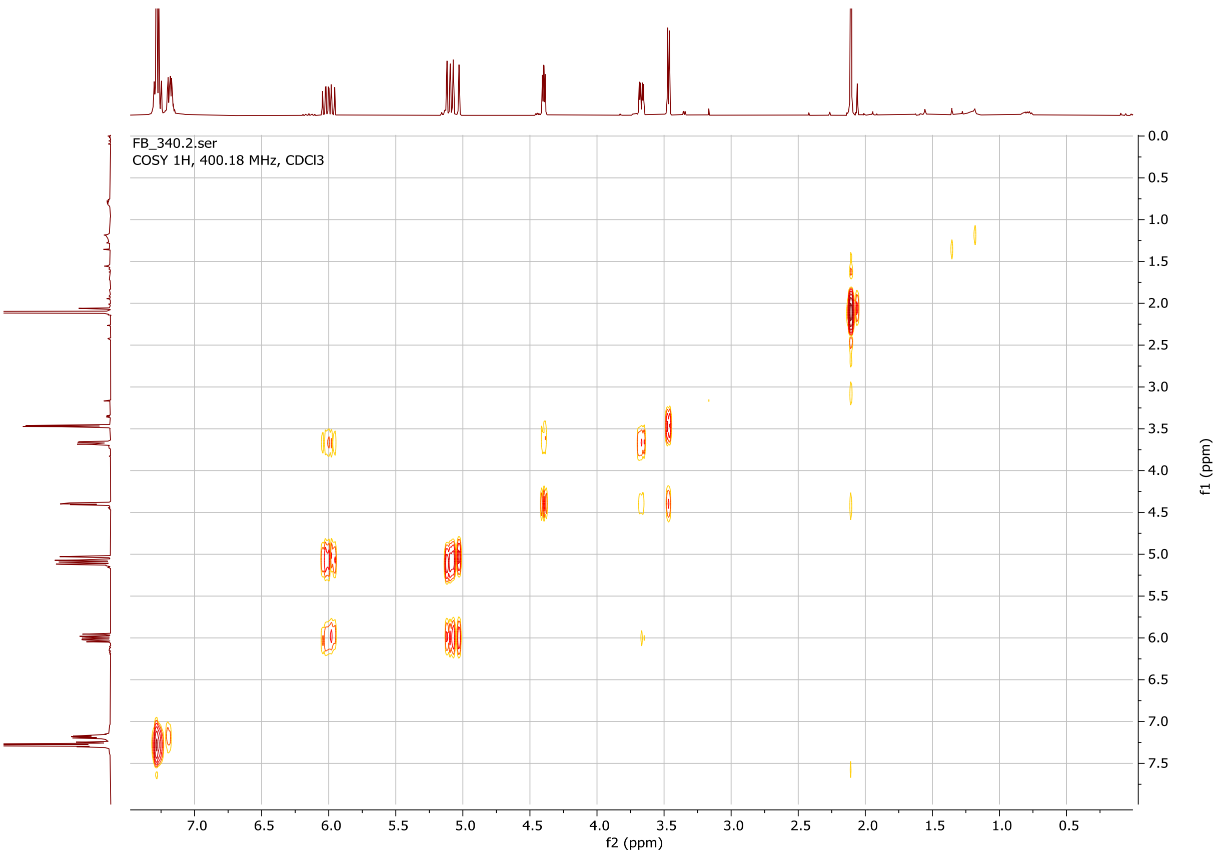
FB_340.2.ser
COSY 1H, 400.18 MHz, CDCl3
f2 (ppm)
f1 (ppm)

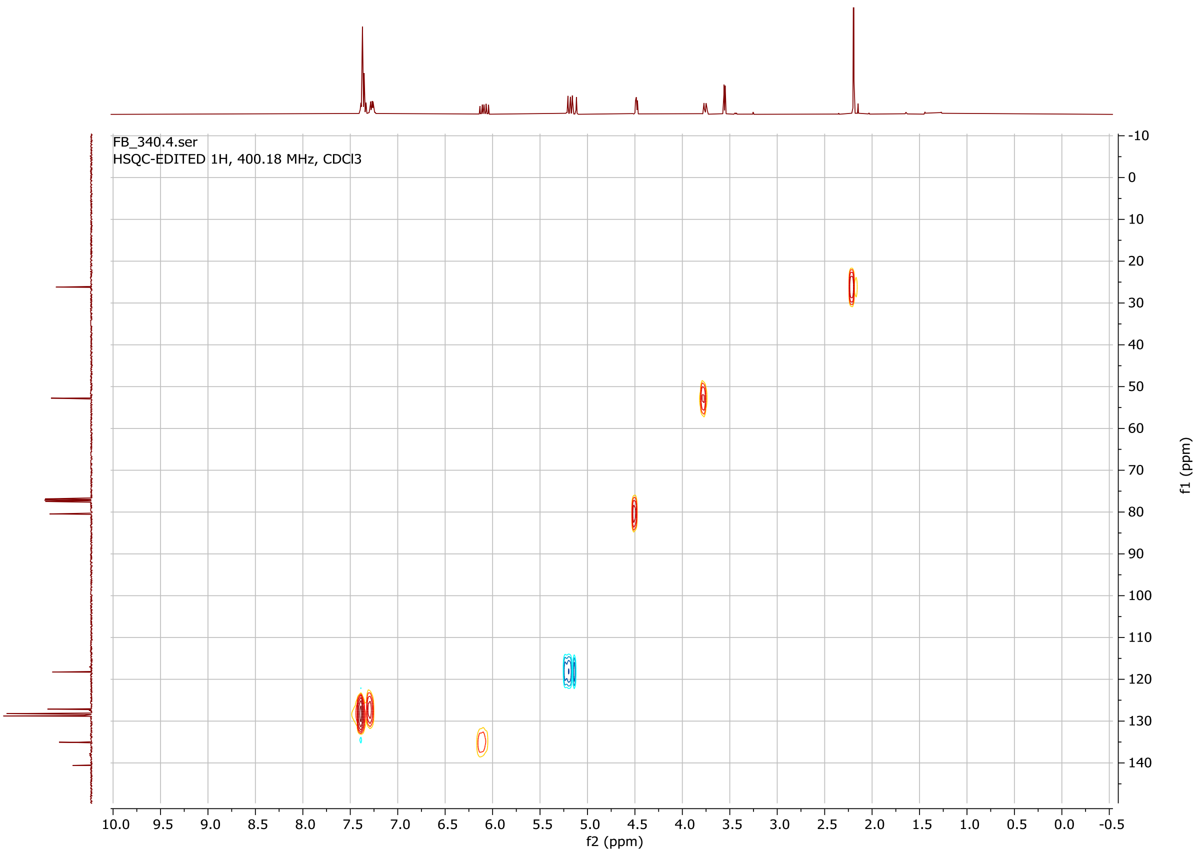
FB_340.4.ser
HSQC-EDITED 1H, 400.18 MHz, CDCl3
f2 (ppm)
f1 (ppm)

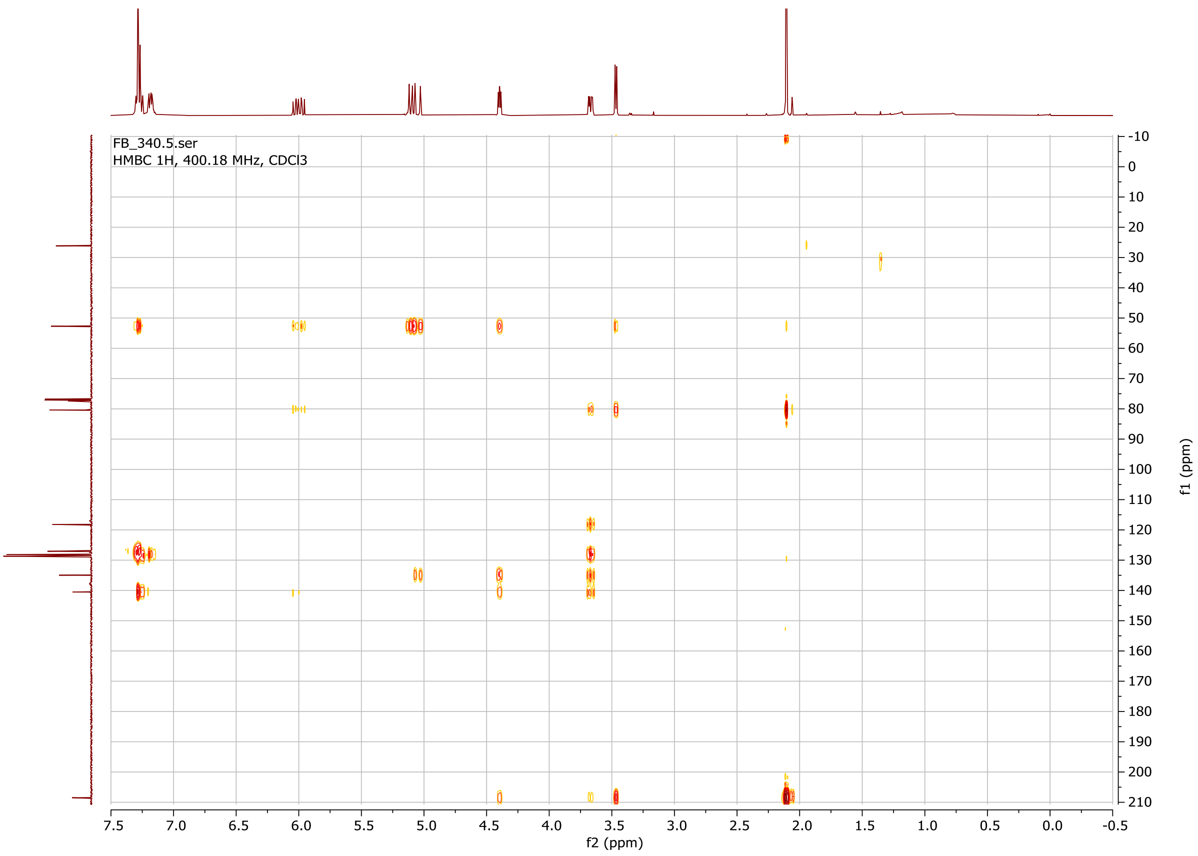

1-Isopropoxy-3-phenylpent-4-en-2-ol **6s**

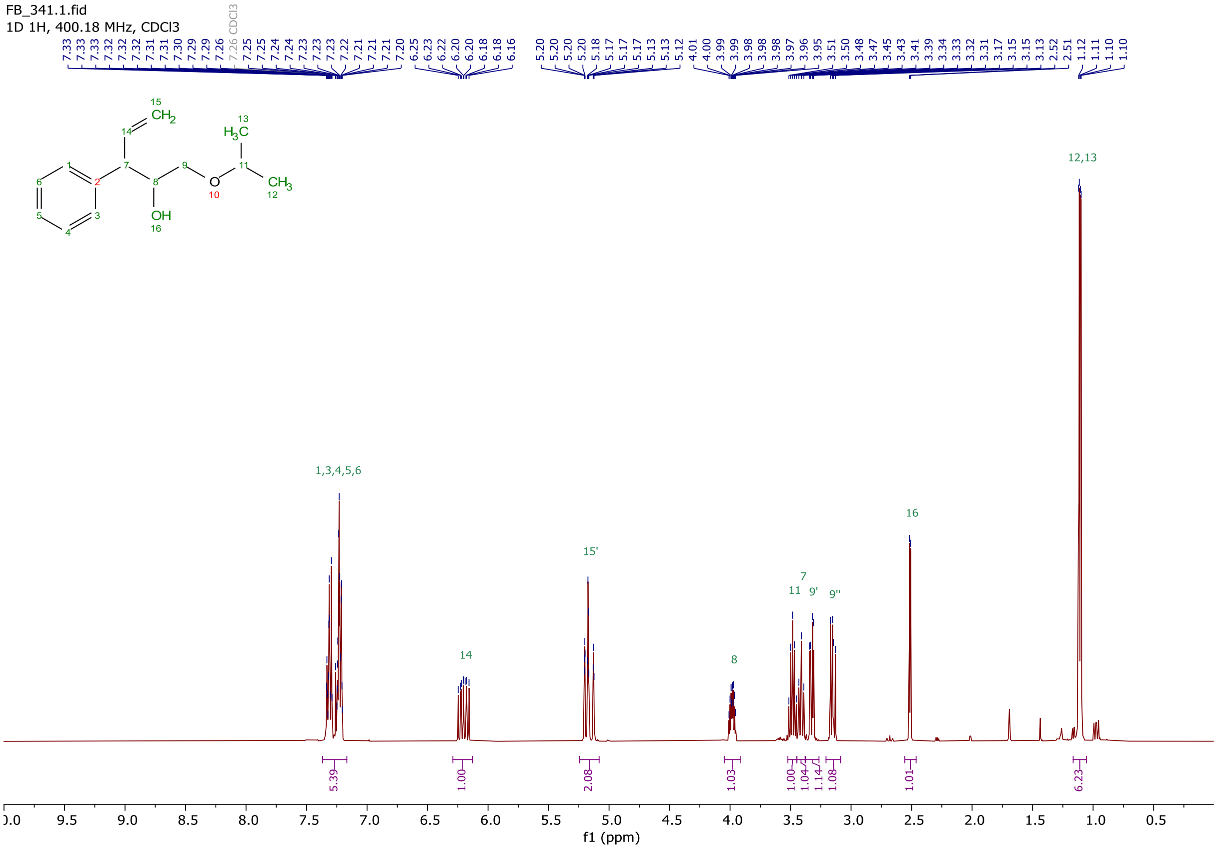

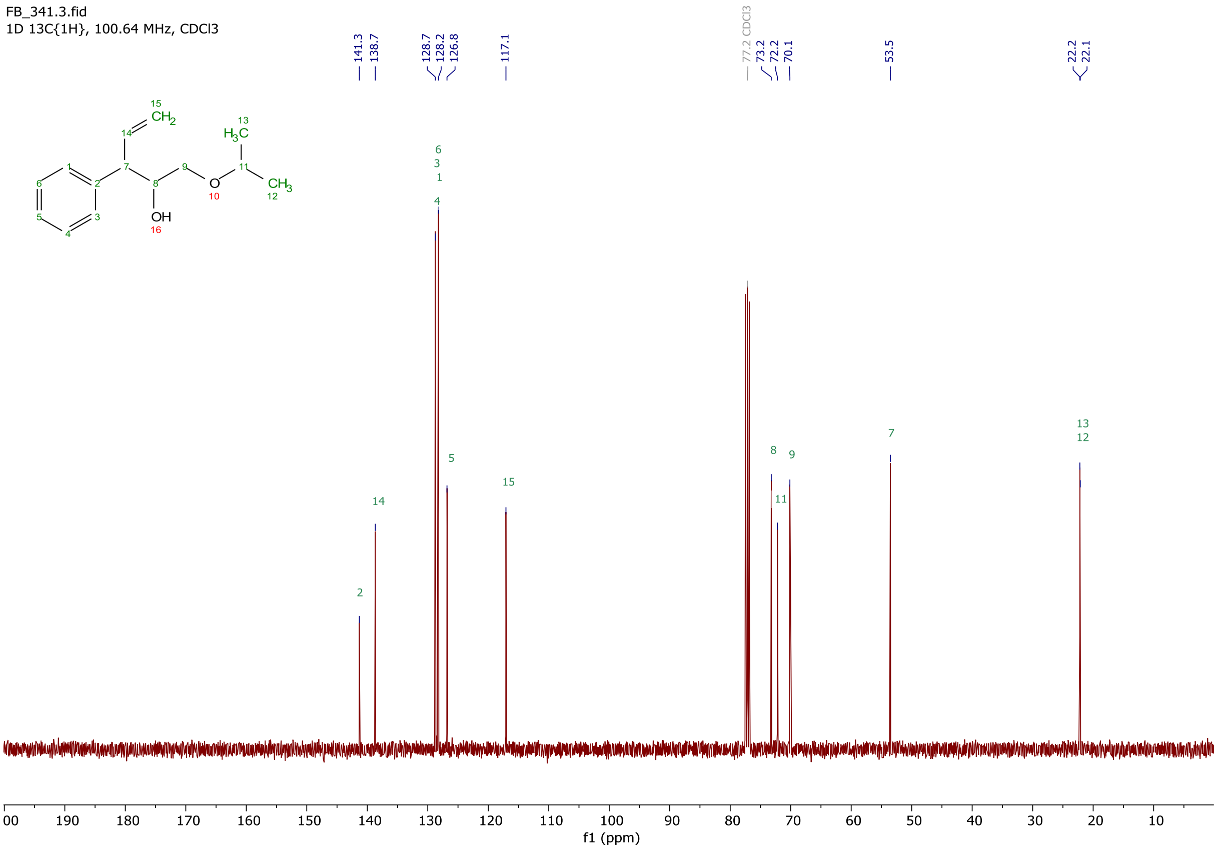
FB_341.3.fid
1D 13C{1H}, 100.64 MHz, CDCl3
141.3
138.7
128.7
128.2
126.8
117.1
77.2 CDCl3
73.2
72.2
70.1
53.5
22.2
22.1
OH
f1 (ppm)

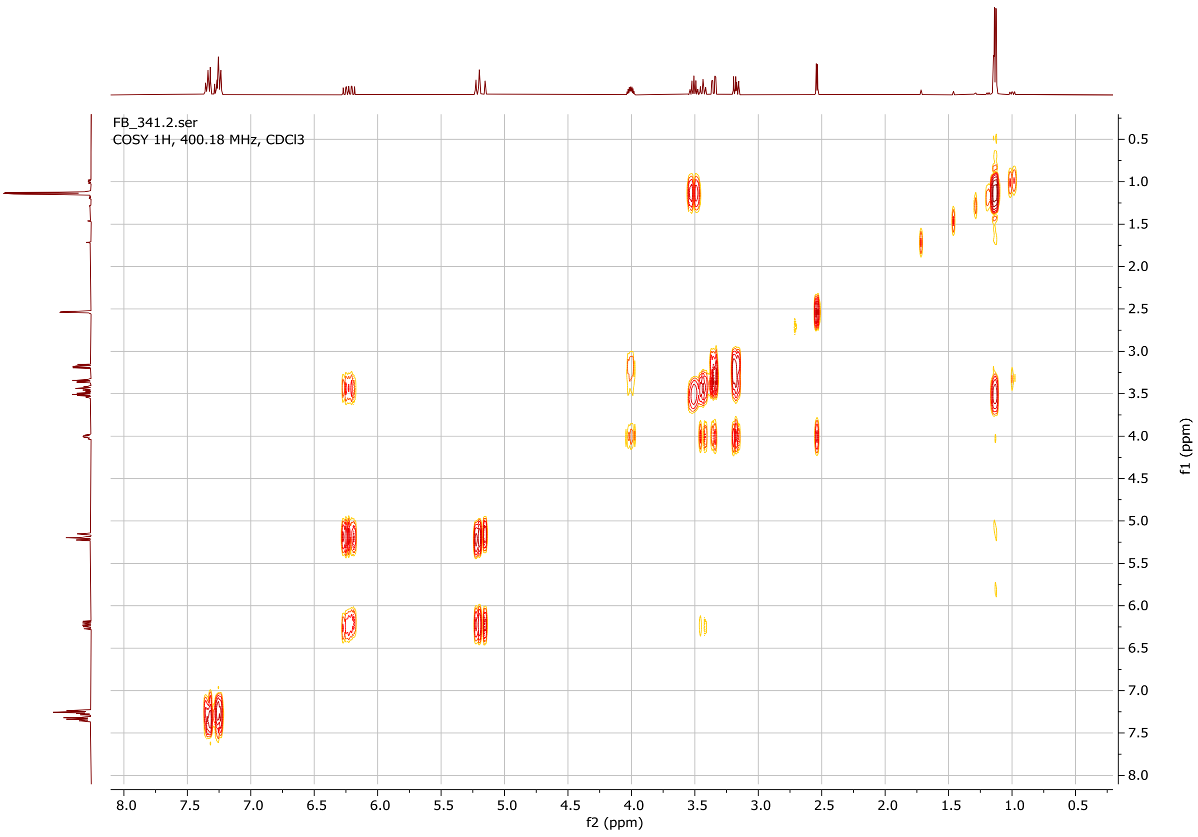
FB_341.2.ser
COSY 1H, 400.18 MHz, CDCl3
f2 (ppm)
f1 (ppm)

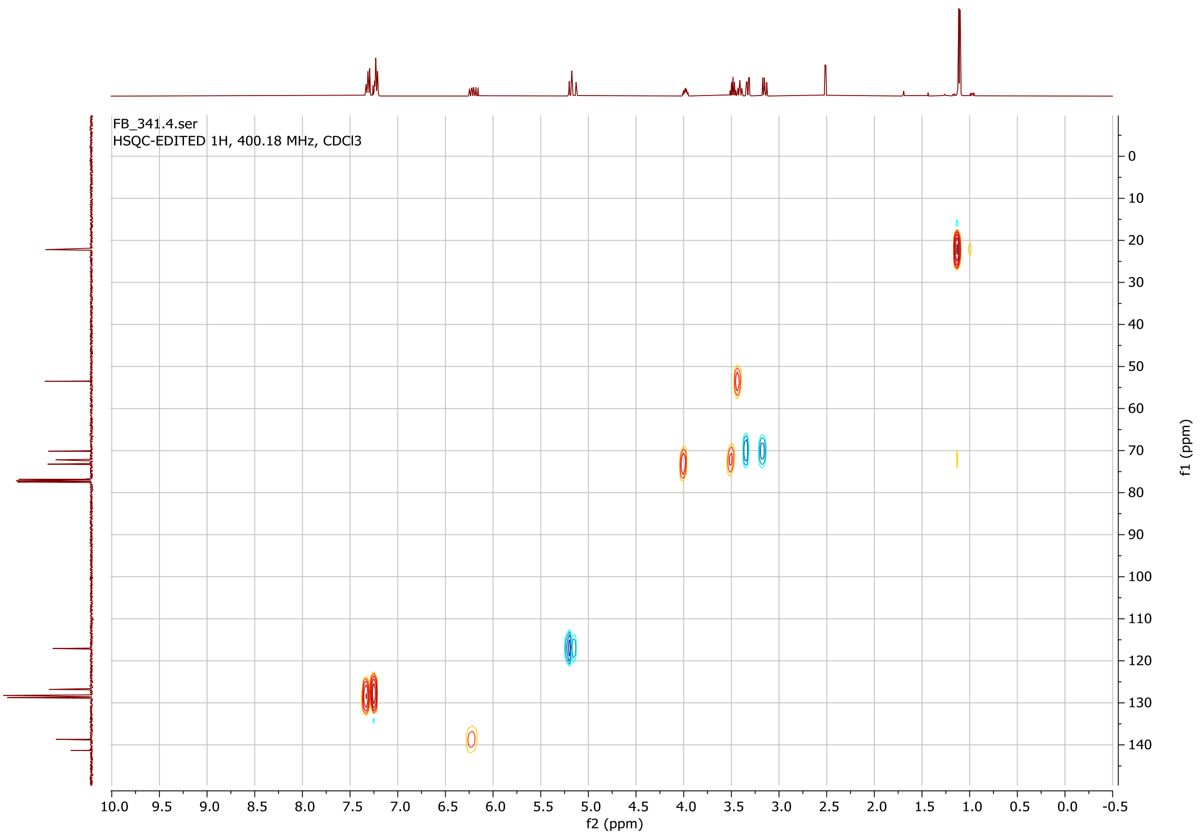

FB_341.4.ser
HSQC-EDITED 1H, 400.18 MHz, CDCl3
f2 (ppm)
f1 (ppm)

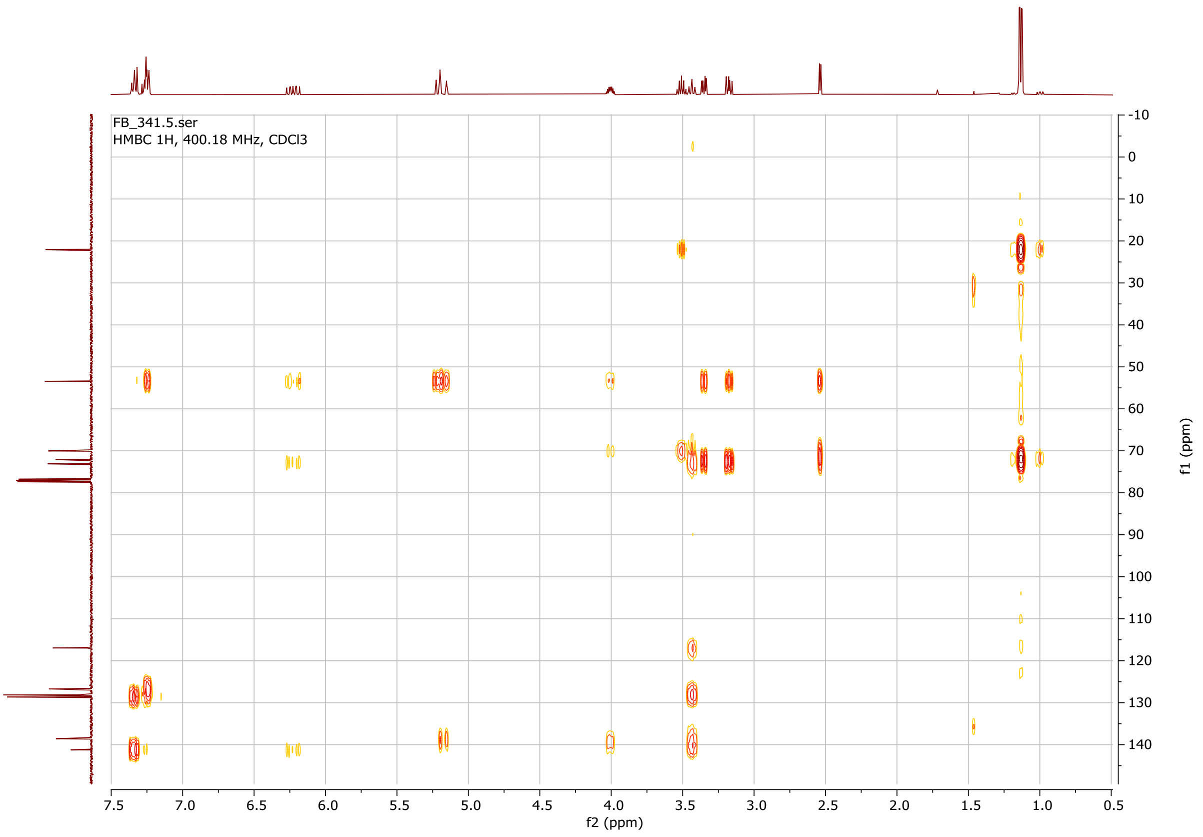

FB_341.5.ser
HMBC 1H, 400.18 MHz, CDCl3
f2 (ppm)
f1 (ppm)

Methyl 2-(hydroxy(phenyl)methyl)but-3-enoate **6t**

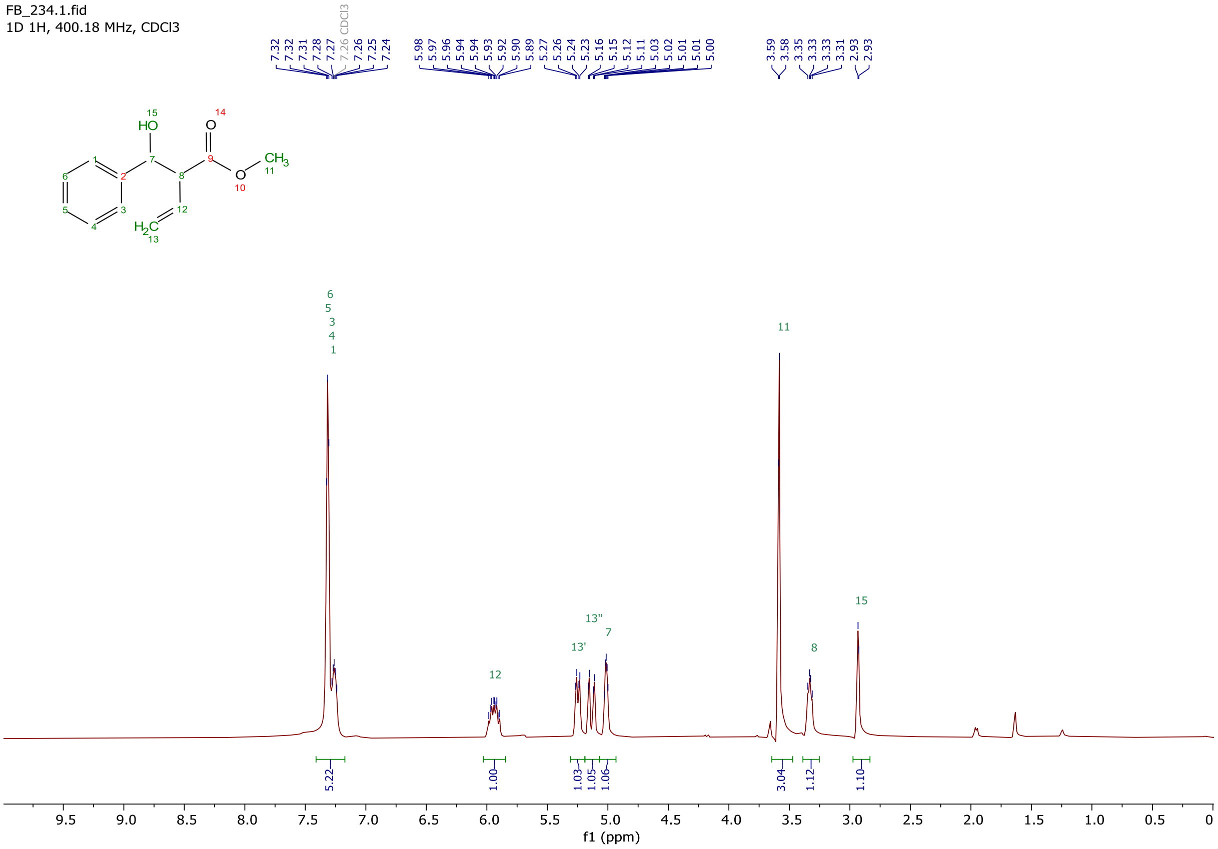

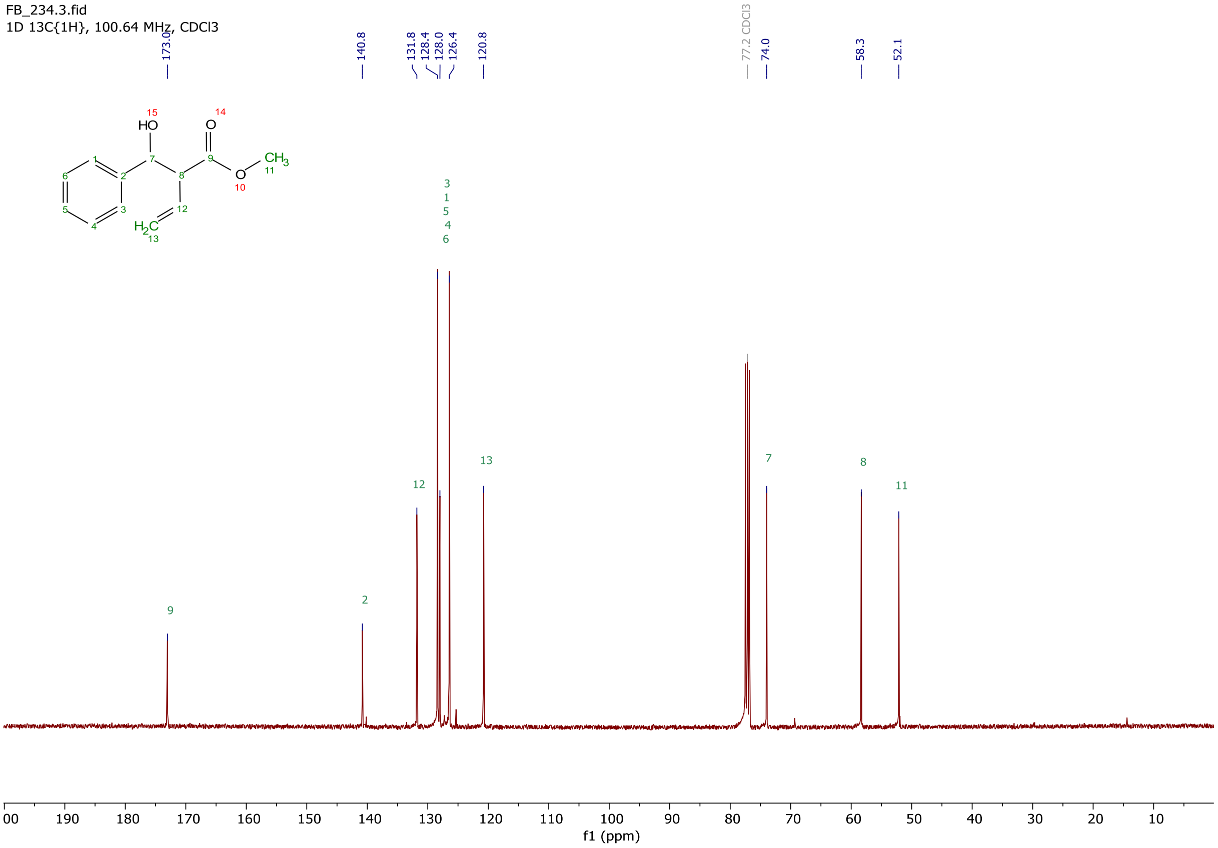

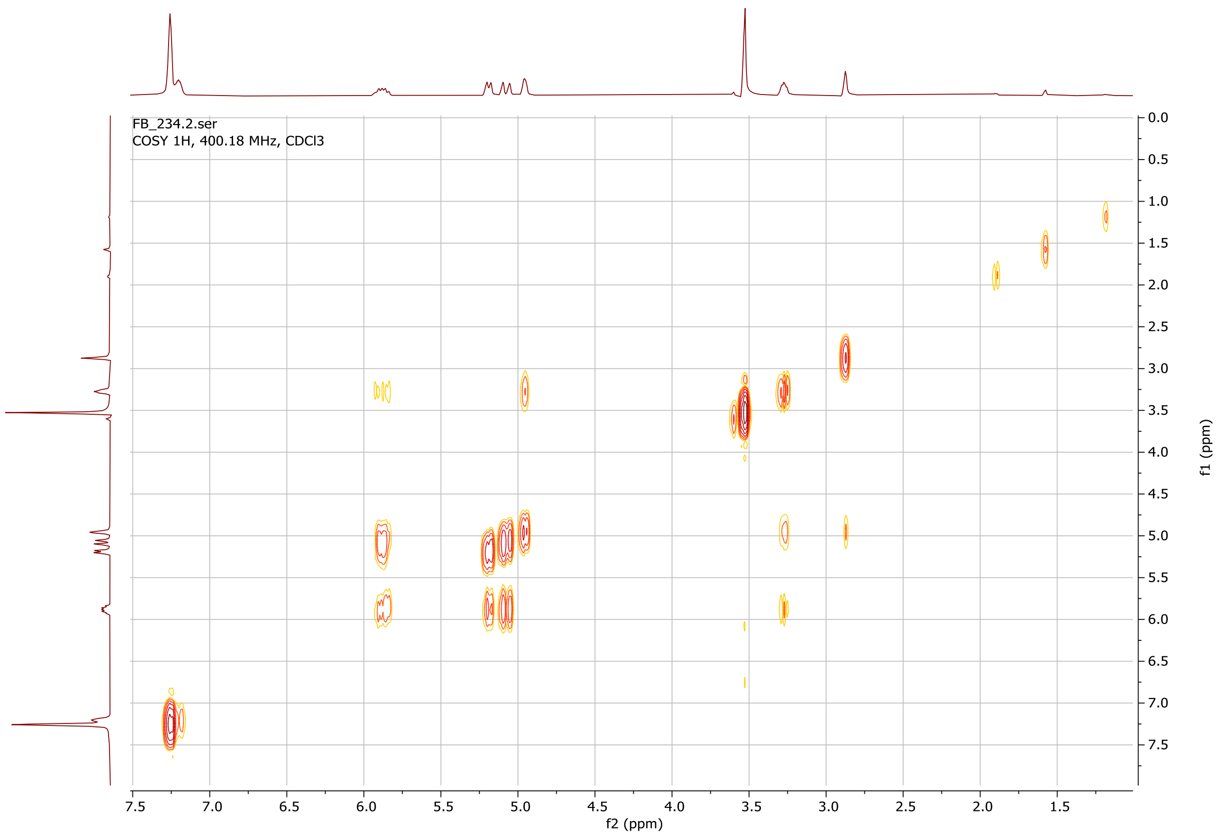
FB_234.2.ser
COSY 1H, 400.18 MHz, CDCl3
f2 (ppm)
f1 (ppm)

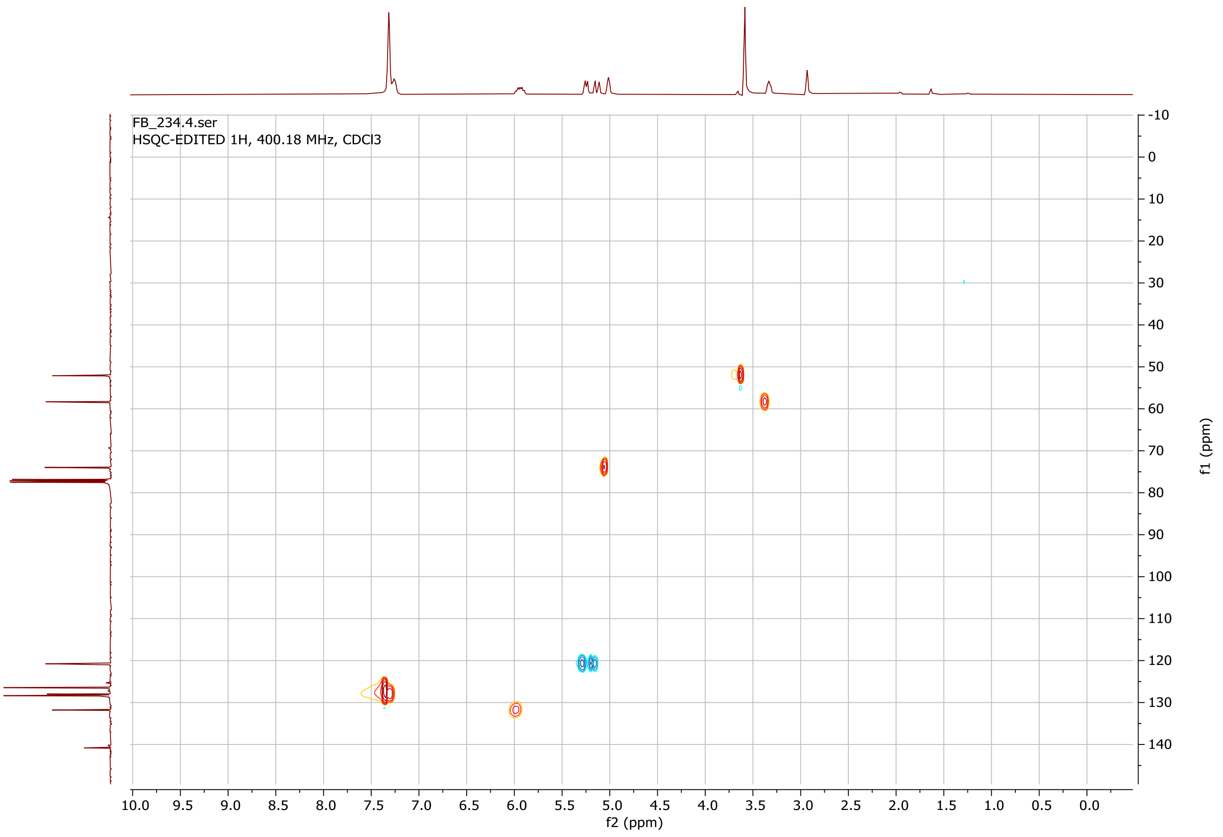
FB_234.4.ser
HSQC-EDITED 1H, 400.18 MHz, CDCl3
f2 (ppm)
f1 (ppm)

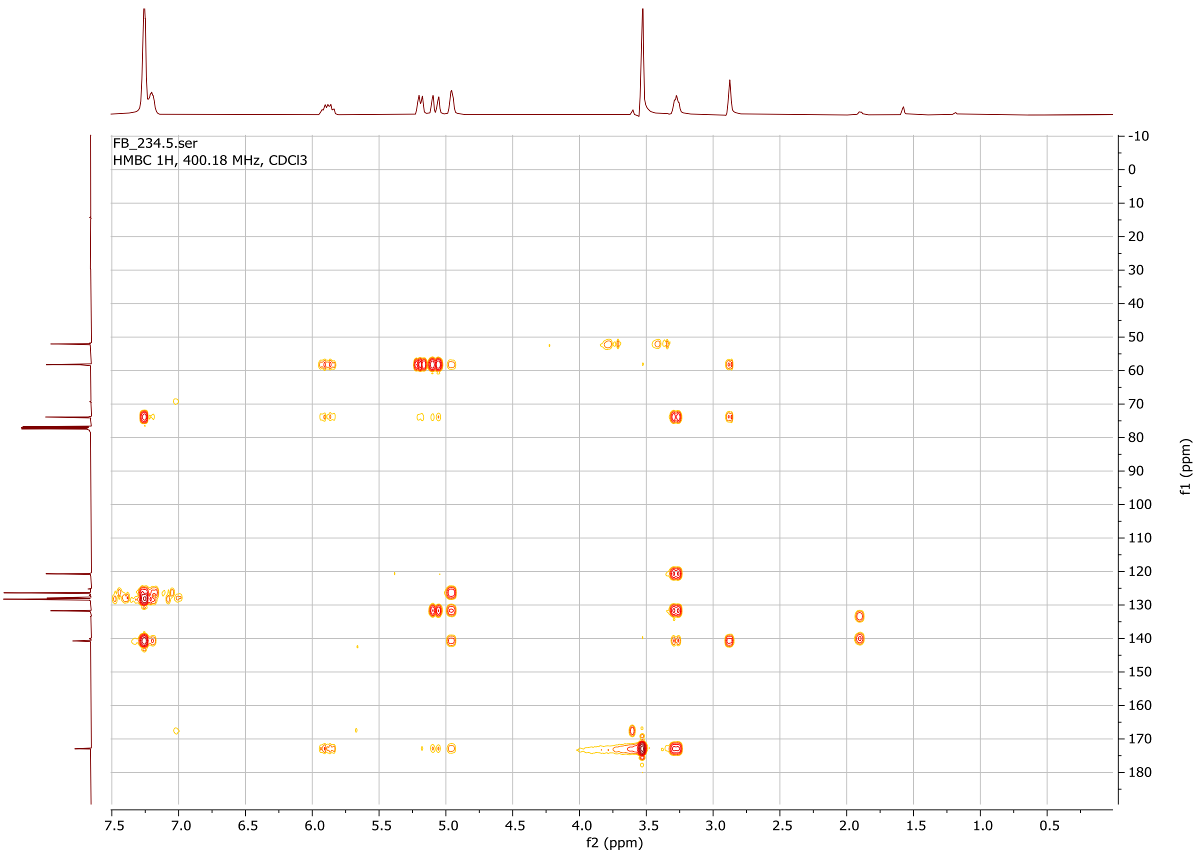

2-(Hydroxy(phenyl)methyl)-*N*,*N*-dimethylbut-3-enamide **6u**

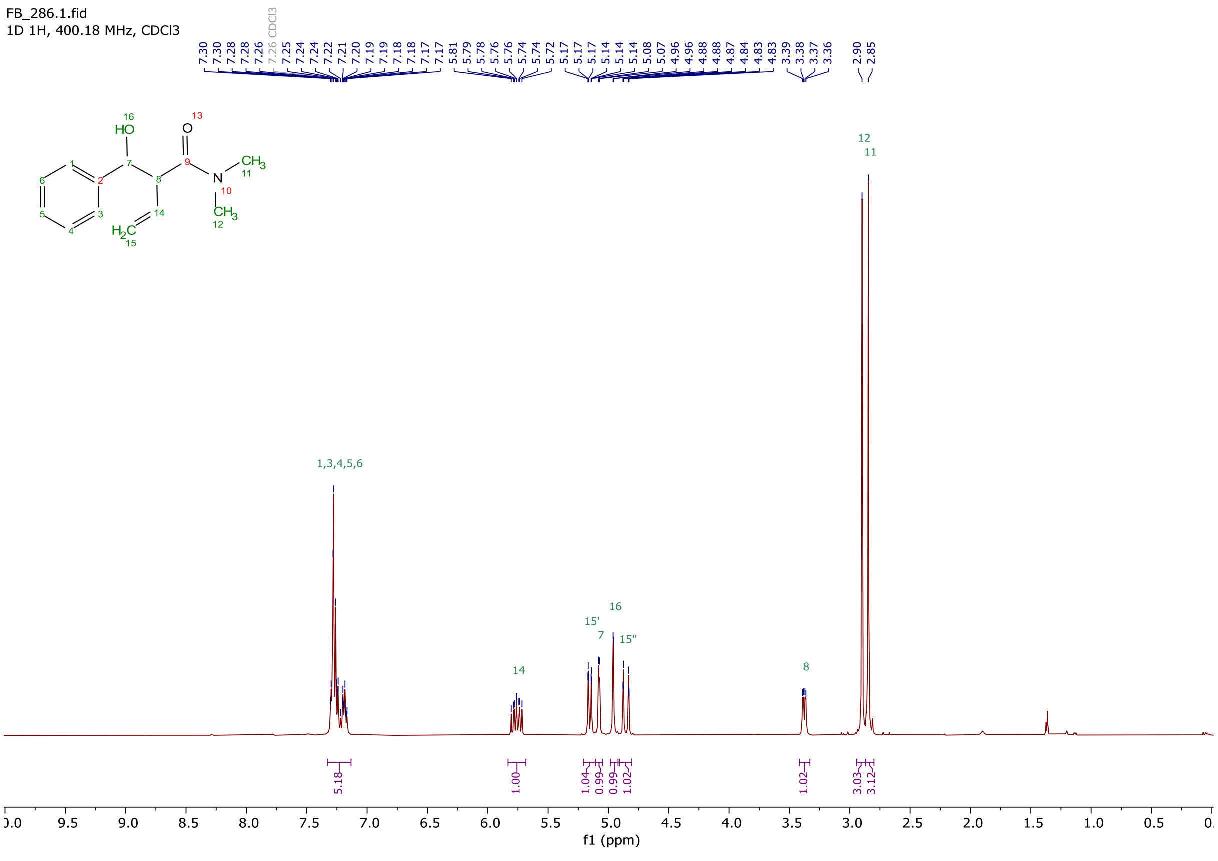

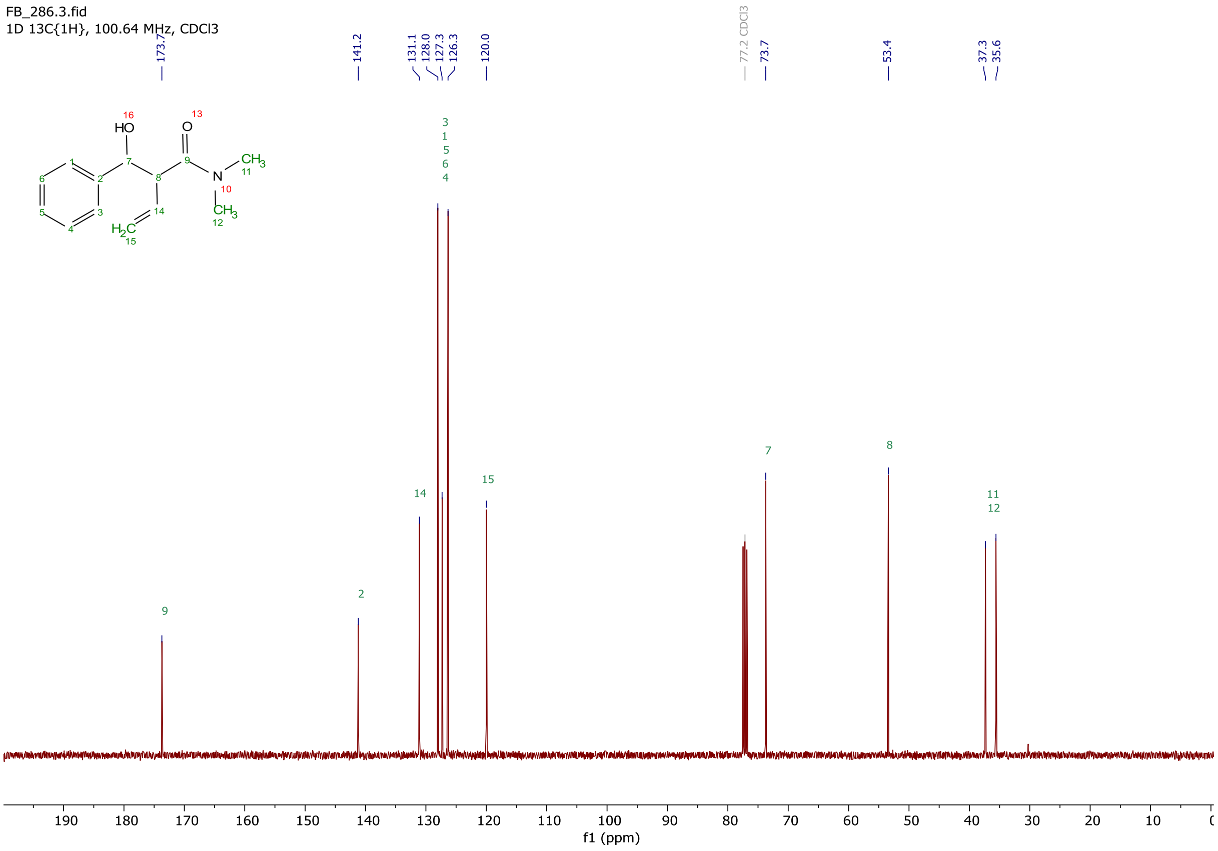
FB_286.3.fid
1D 13C{1H}, 100.64 MHz, CDCl3
173.7
141.2
131.1
128.0
127.3
126.3
120.0
77.2 CDCl3
73.7
53.4
37.3
35.6
f1 (ppm)

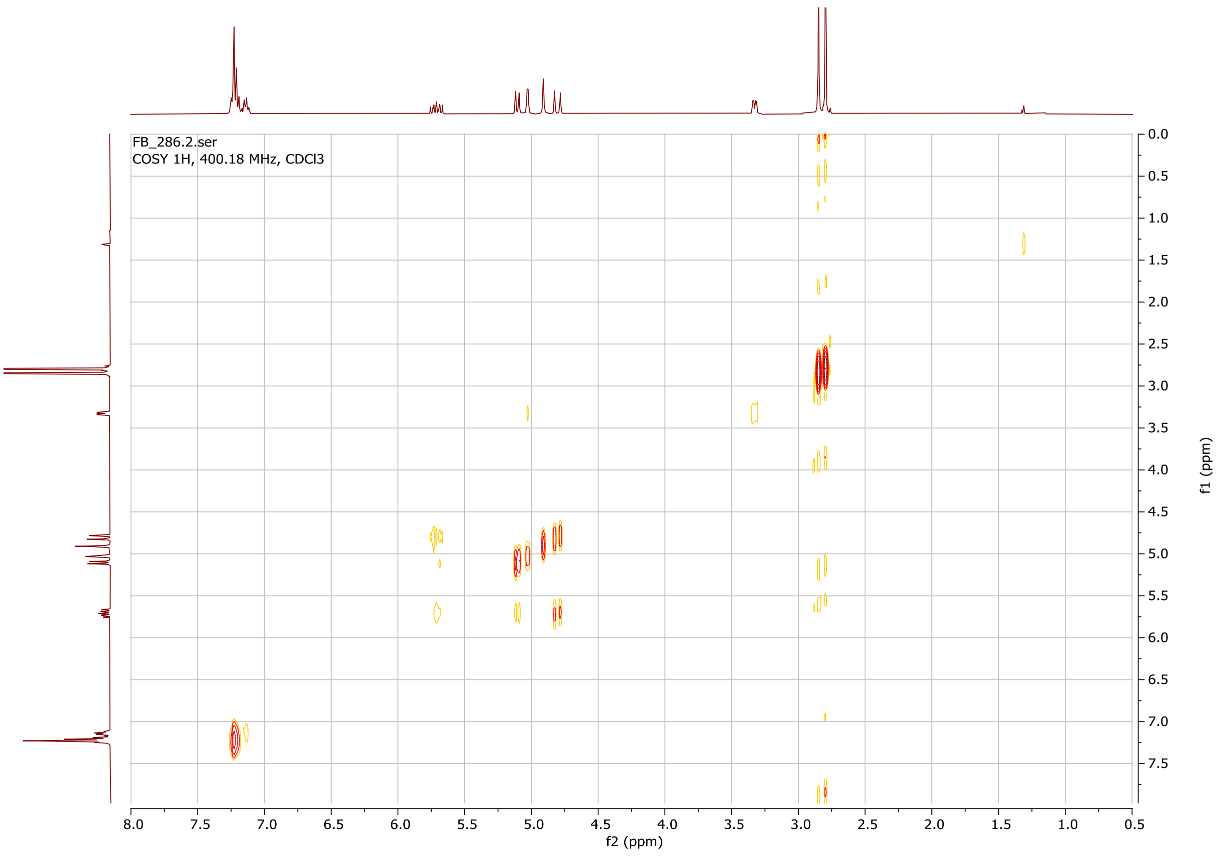
FB_286.2.ser
COSY 1H, 400.18 MHz, CDCl3
f2 (ppm)
f1 (ppm)

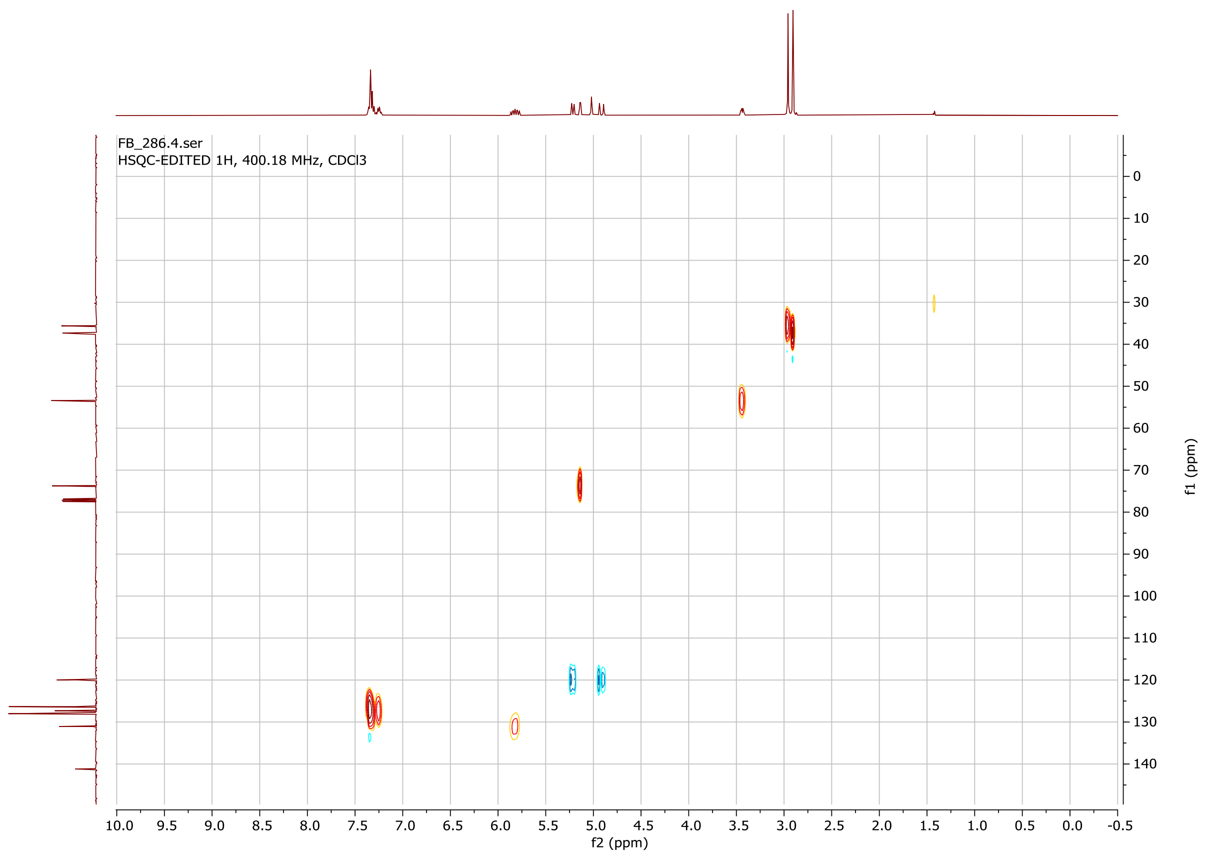
FB_286.4.ser
HSQC-EDITED 1H, 400.18 MHz, CDCl3
f1 (ppm)
f2 (ppm)

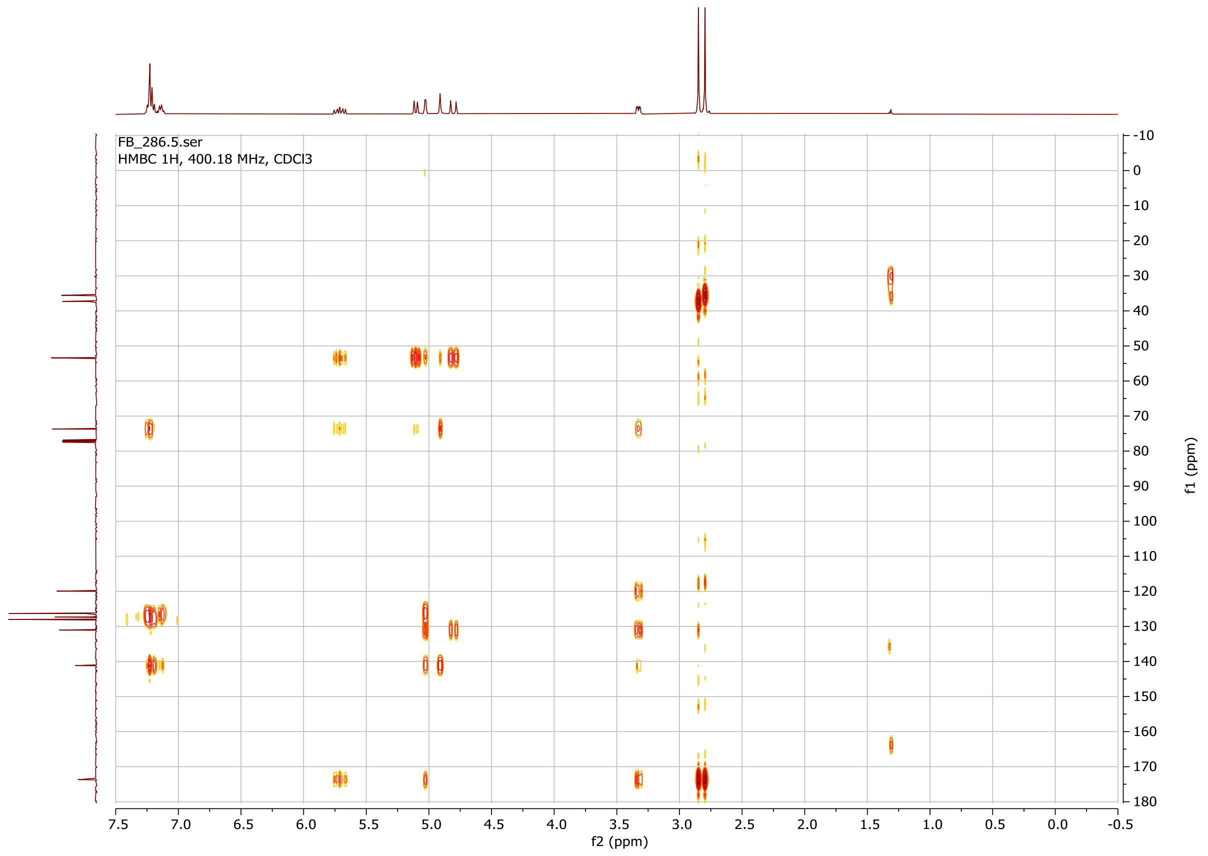
FB_286.5.ser
HMBC 1H, 400.18 MHz, CDCl3
f1 (ppm)
f2 (ppm)

2-Vinyl-2-cyclohexenone **6v**

FB_260_7-17.1.fid
1D 1H, 400.18 MHz, CDCl3

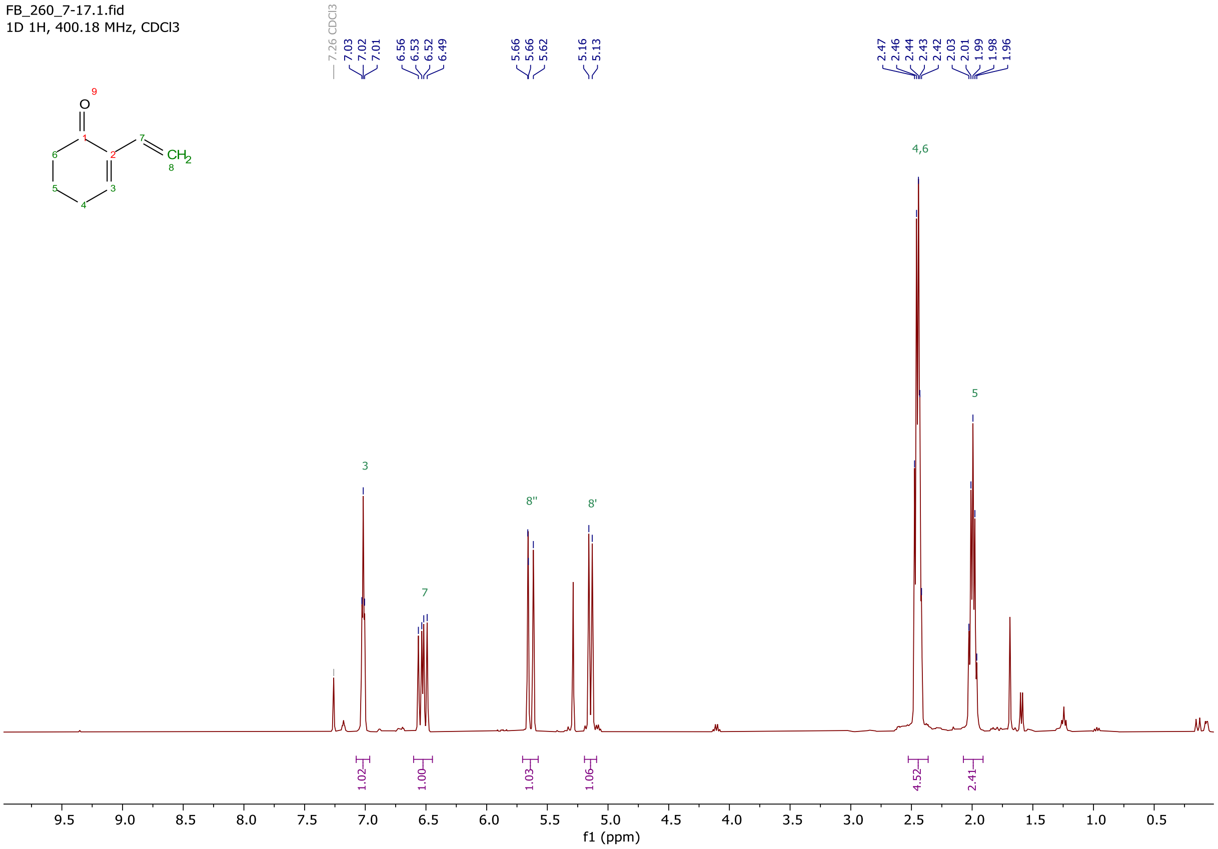

FB_260_7-17.3.fid
1D 13C{1H}, 100.64 MHz, CDCl3

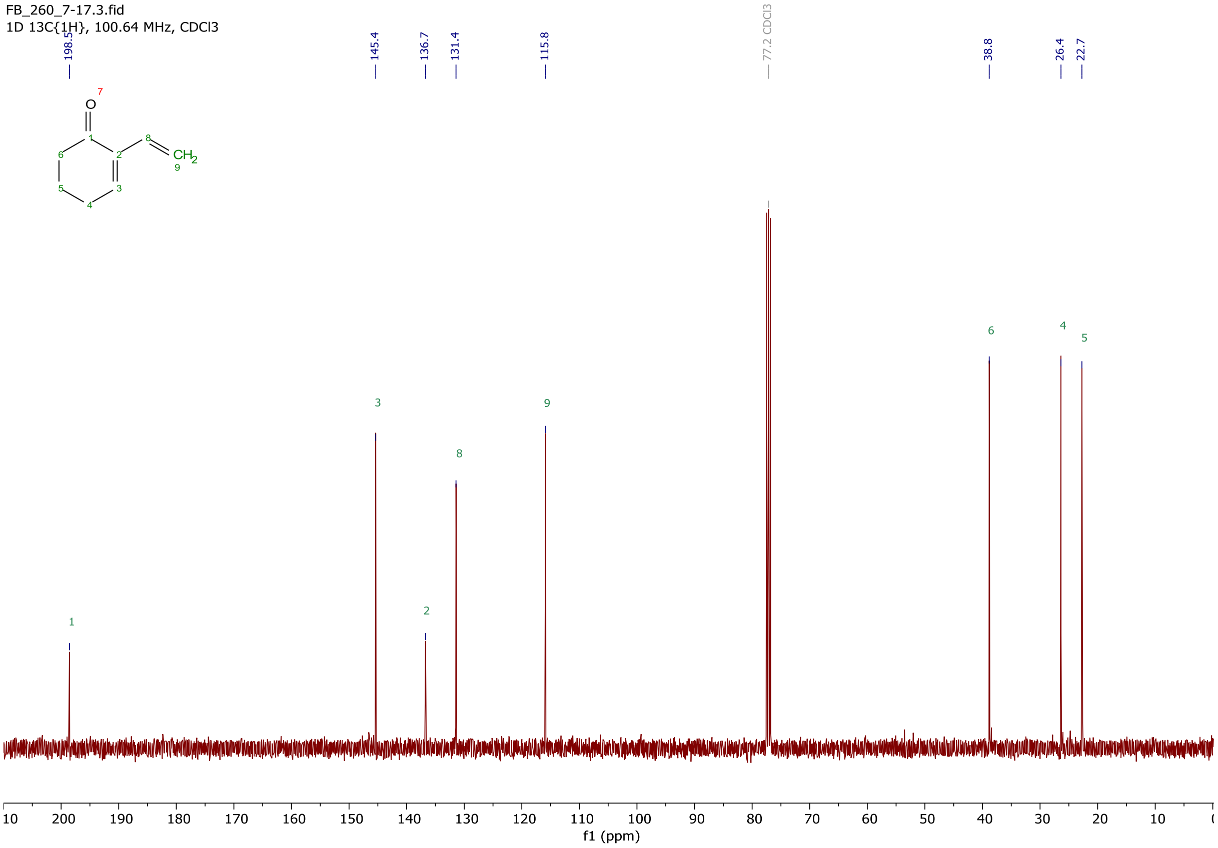

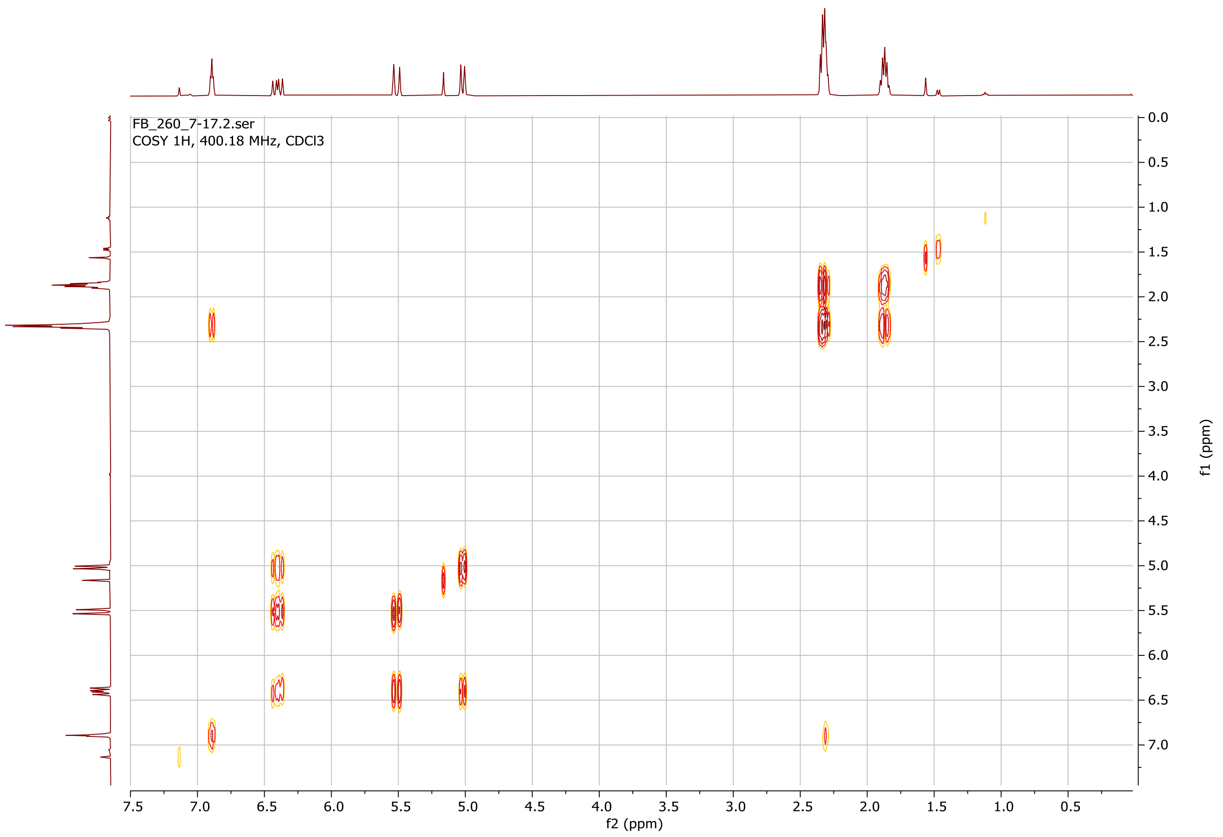

FB_260_7-17.2.ser
COSY 1H, 400.18 MHz, CDCl3
f2 (ppm)
f1 (ppm)

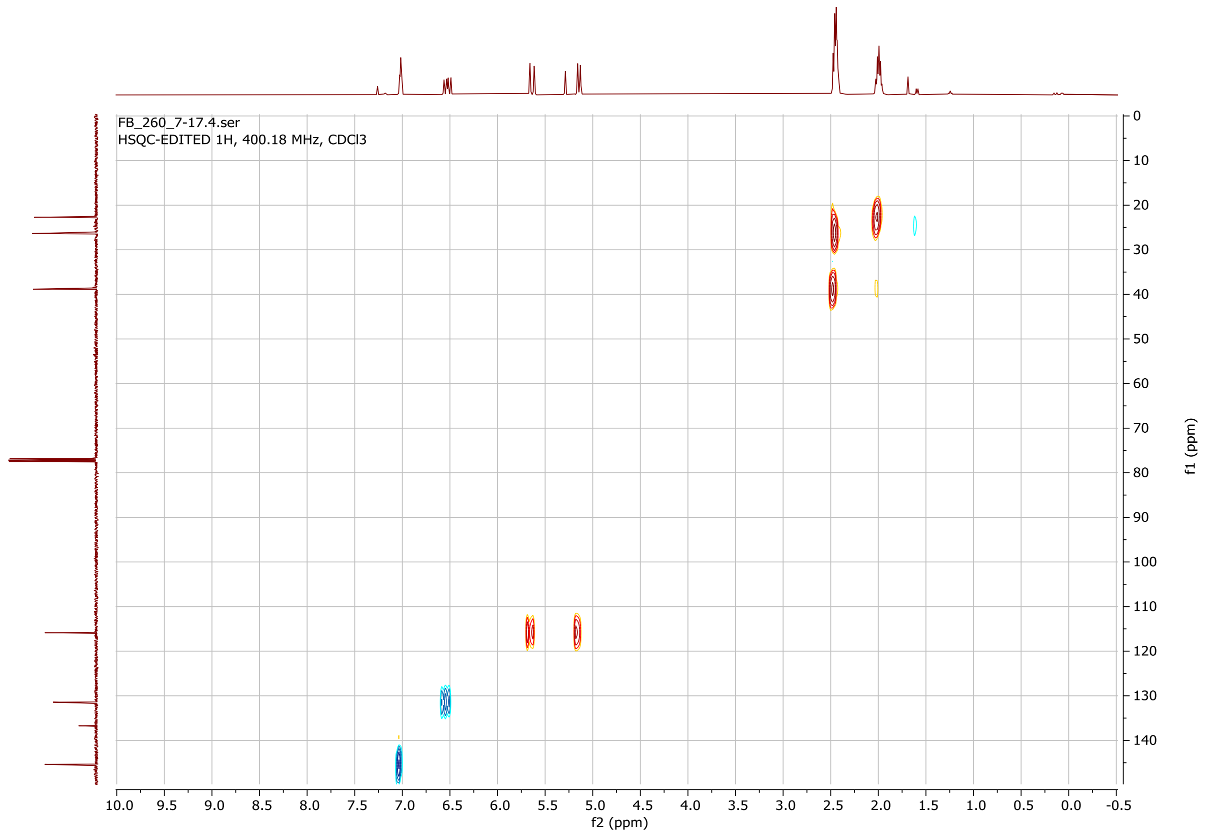

FB_260_7-17.4.ser
HSQC-EDITED 1H, 400.18 MHz, CDCl3
f2 (ppm)
f1 (ppm)

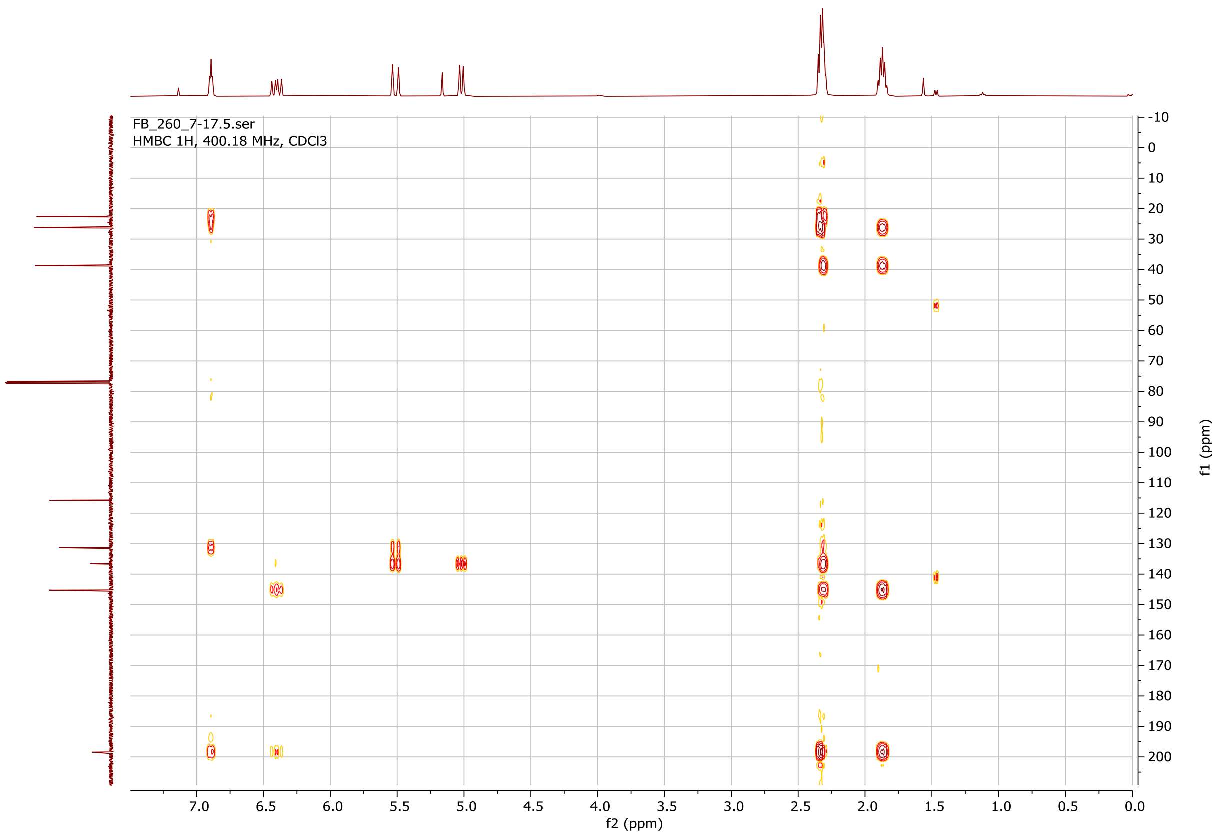

(2-Iodo-1-phenylpropoxy)dimethyl(trimethylsilyl)ethylsilane **7f**

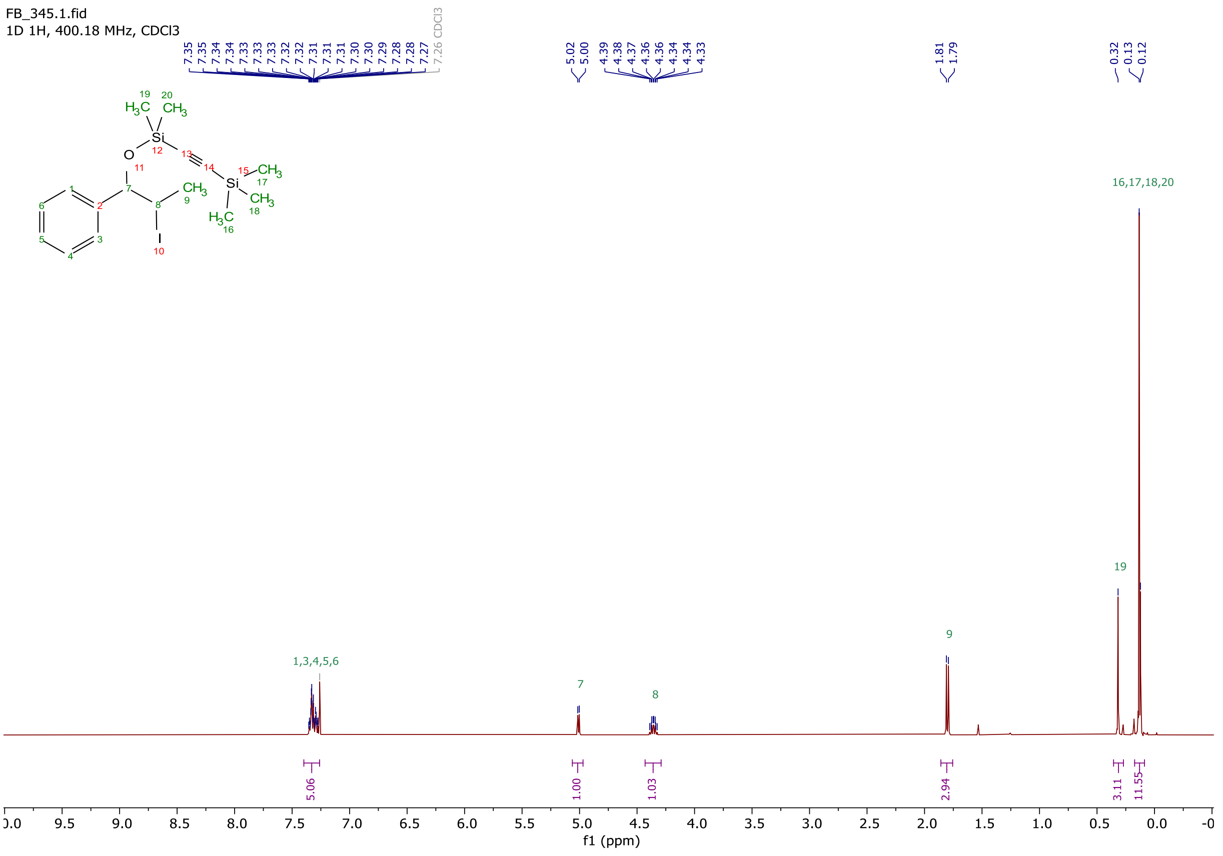

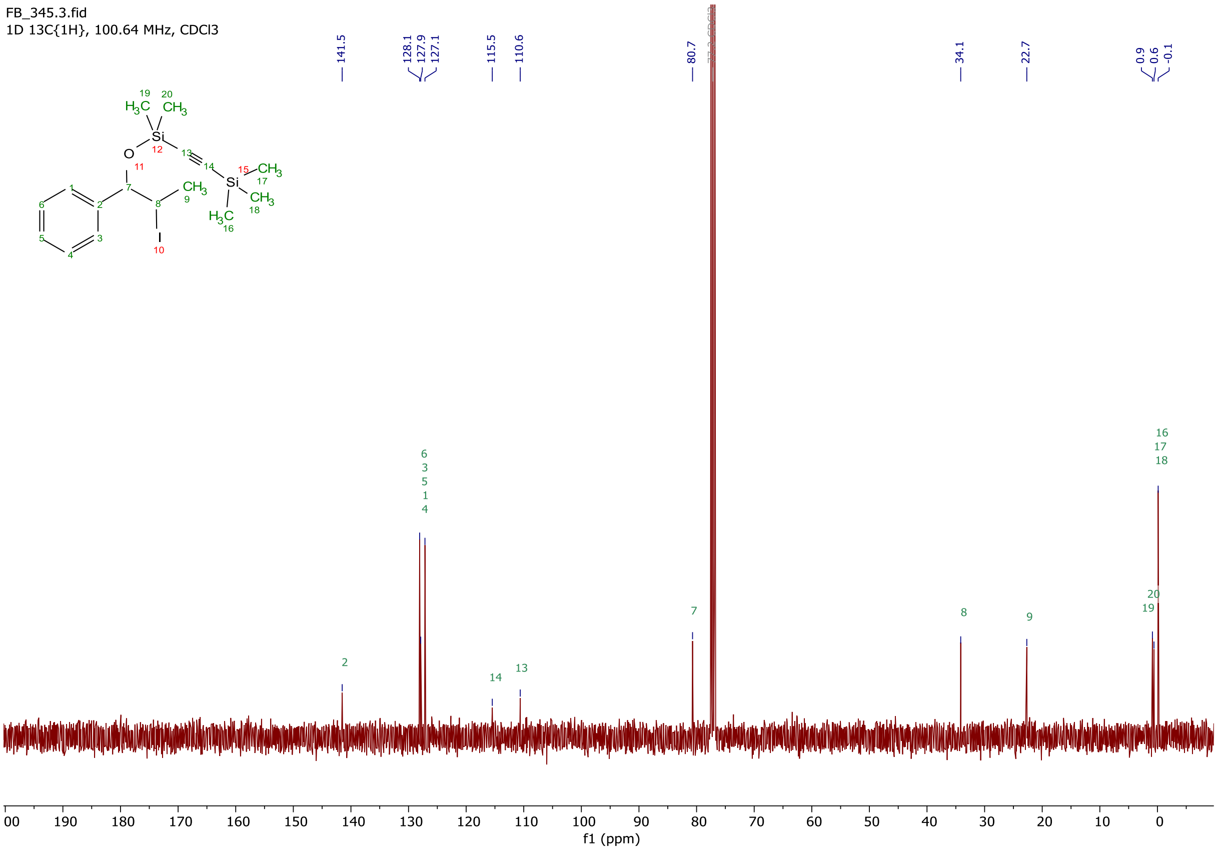
FB_345.3.fid
1D 13C{1H}, 100.64 MHz, CDCl3
141.5
128.1
127.9
127.1
115.5
110.6
80.7
34.1
22.7
0.9
0.6
-0.1
f1 (ppm)

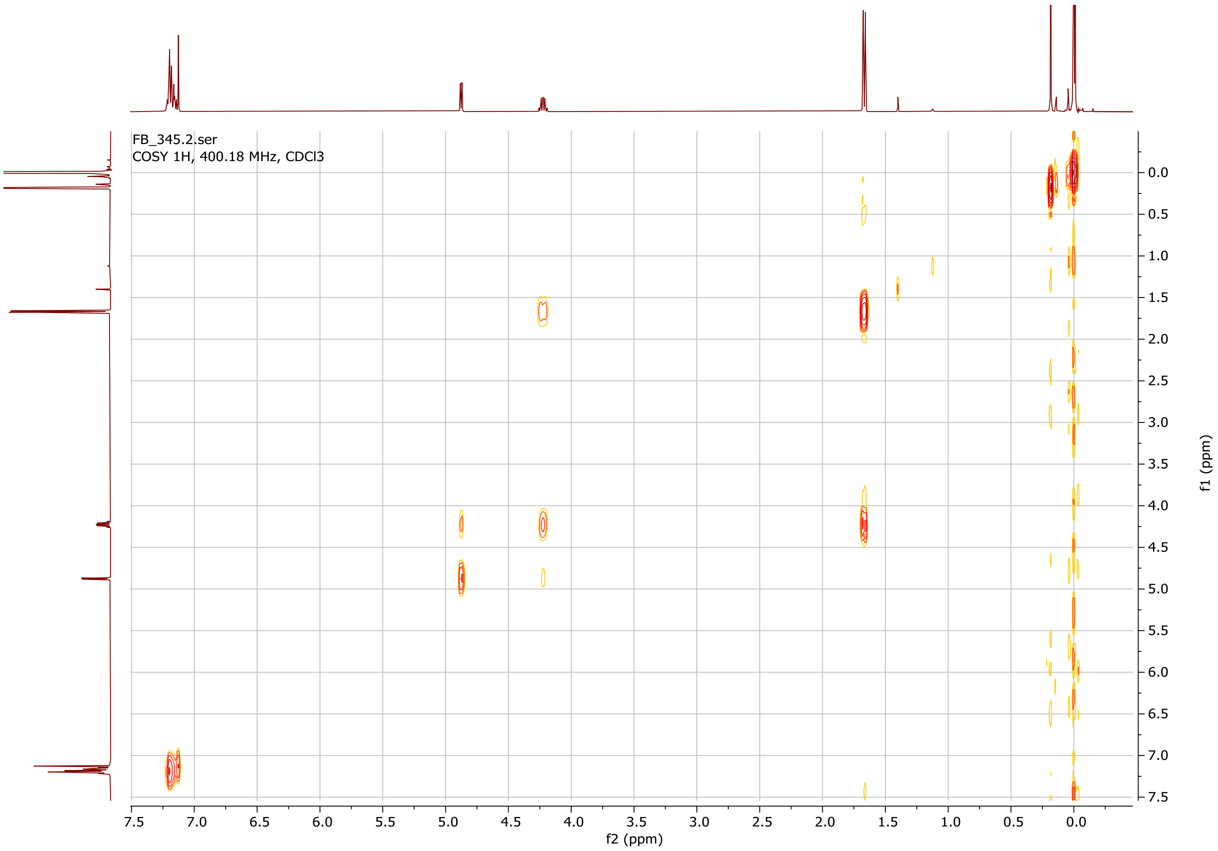
FB_345.2.ser
COSY 1H, 400.18 MHz, CDCl3
f2 (ppm)
f1 (ppm)

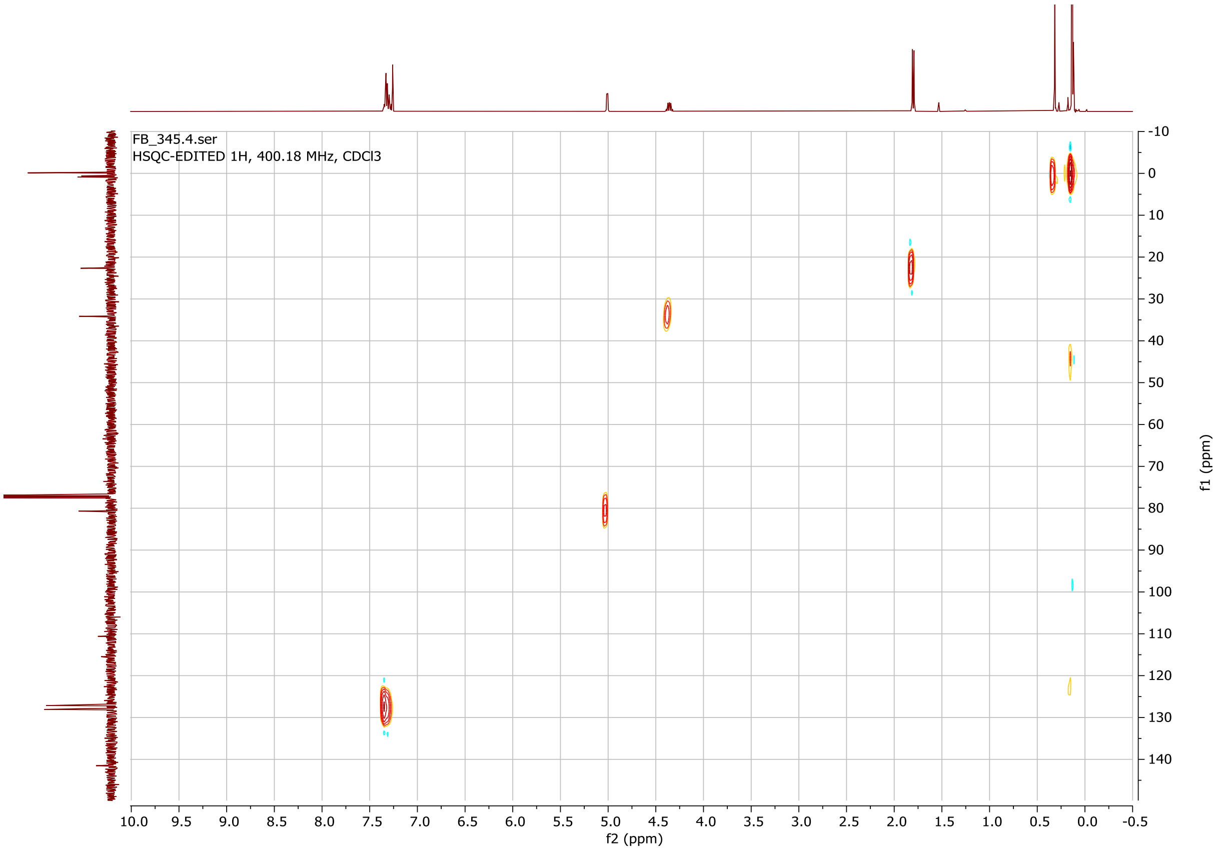
FB_345.4.ser
HSQC-EDITED 1H, 400.18 MHz, CDCl3
f2 (ppm)
f1 (ppm)

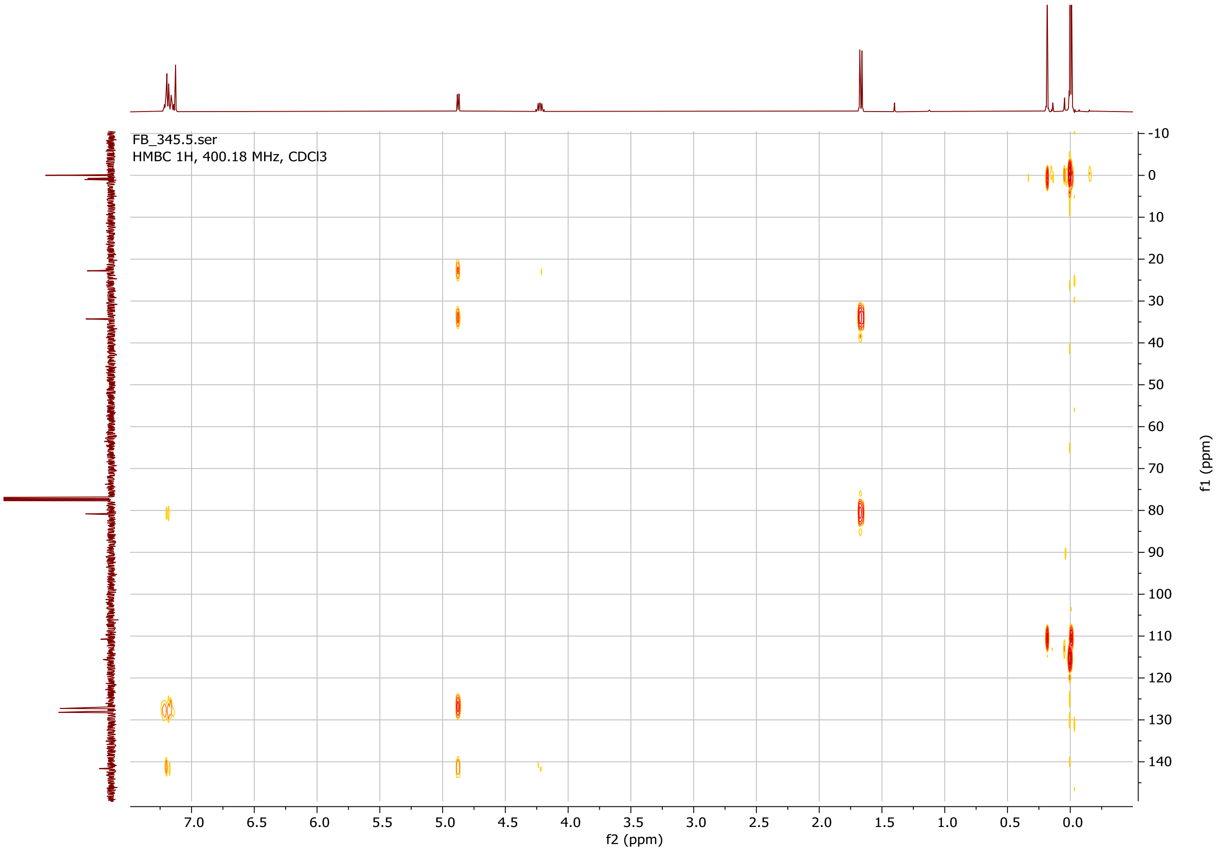
FB_345.5.ser
HMBC 1H, 400.18 MHz, CDCl3
f2 (ppm)
f1 (ppm)

((1-Iodohexan-2-yl)oxy)dimethyl(trimethylsilyl)ethynylsilane **7k**

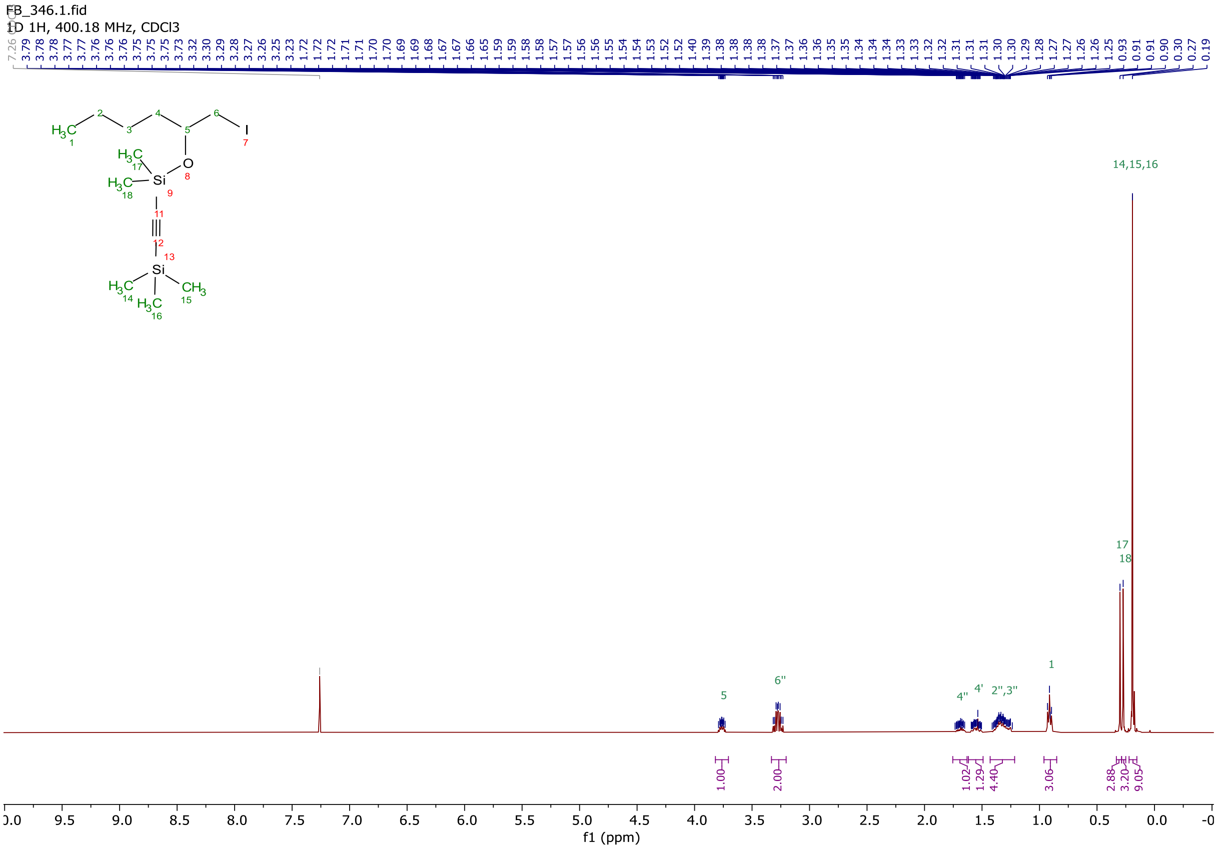

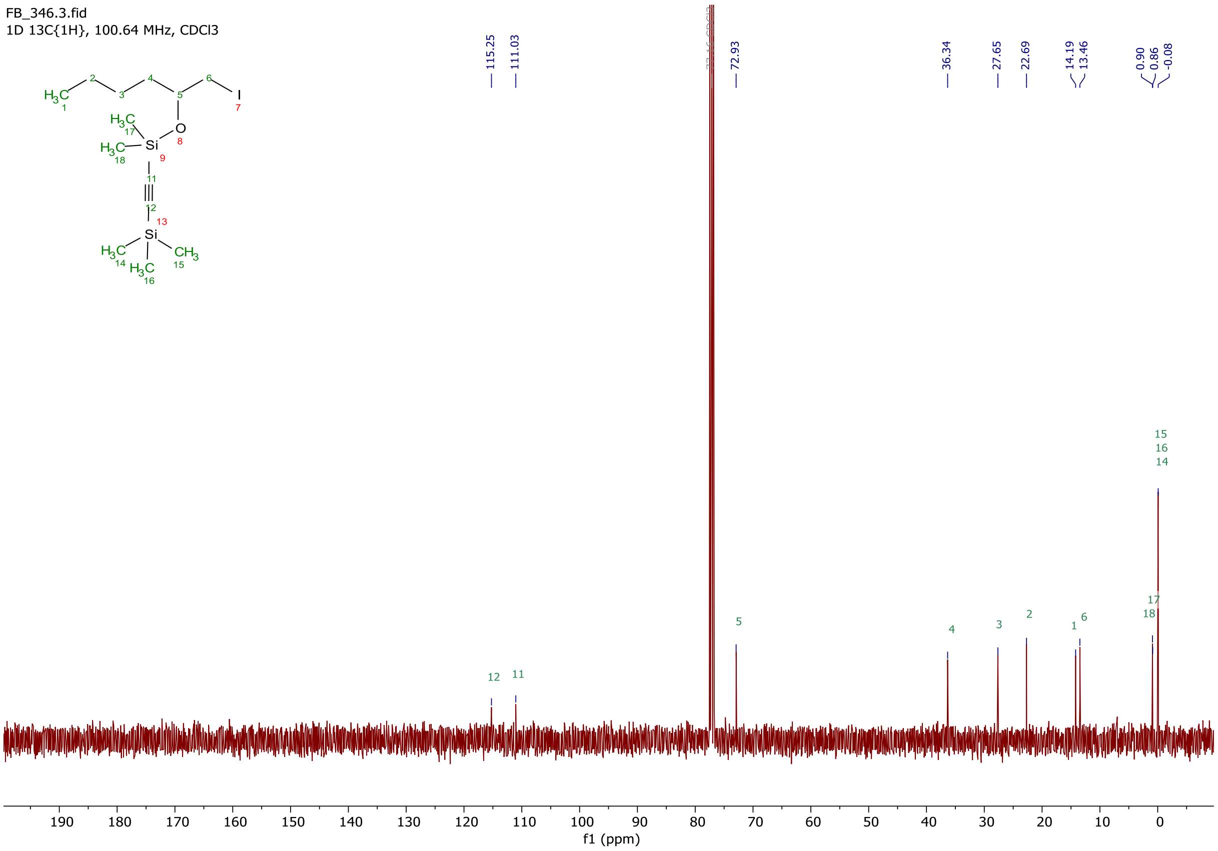

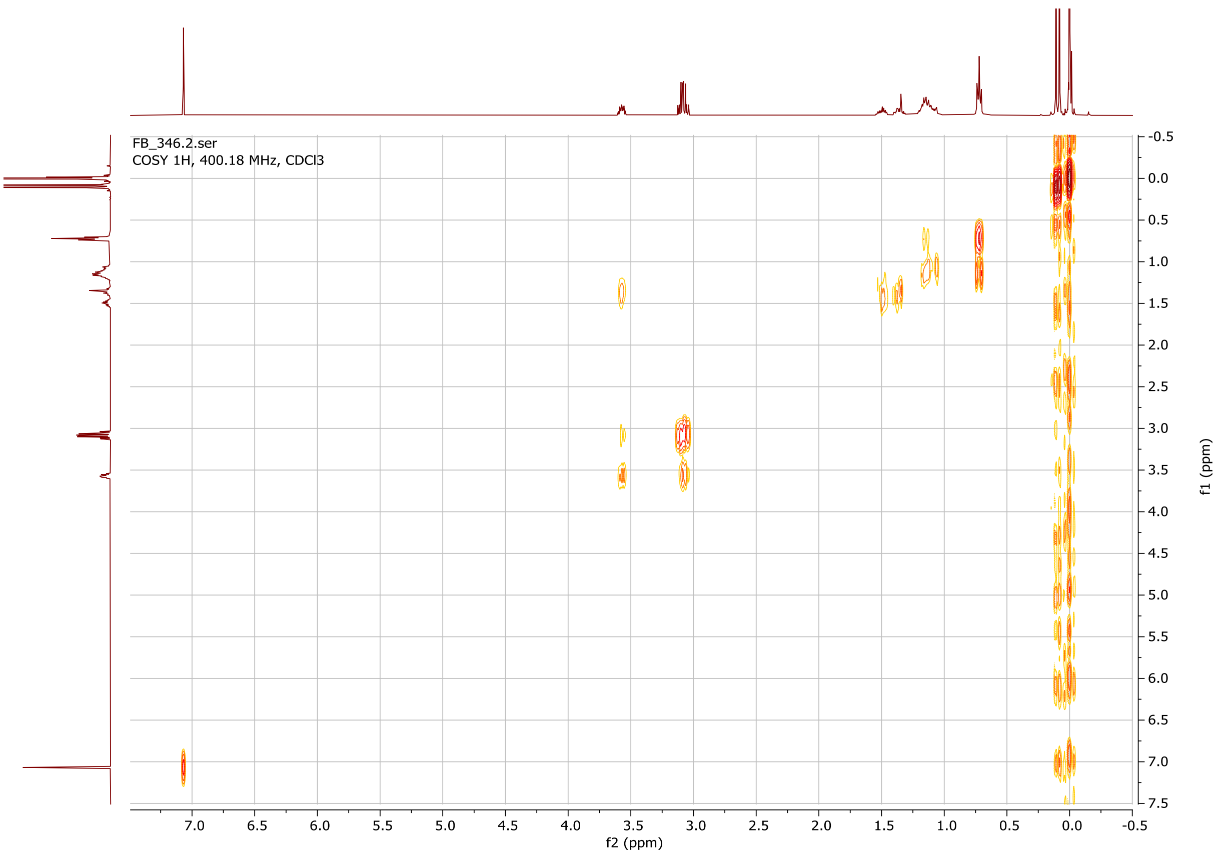
FB_346.2.ser
COSY 1H, 400.18 MHz, CDCl3
f2 (ppm)
f1 (ppm)

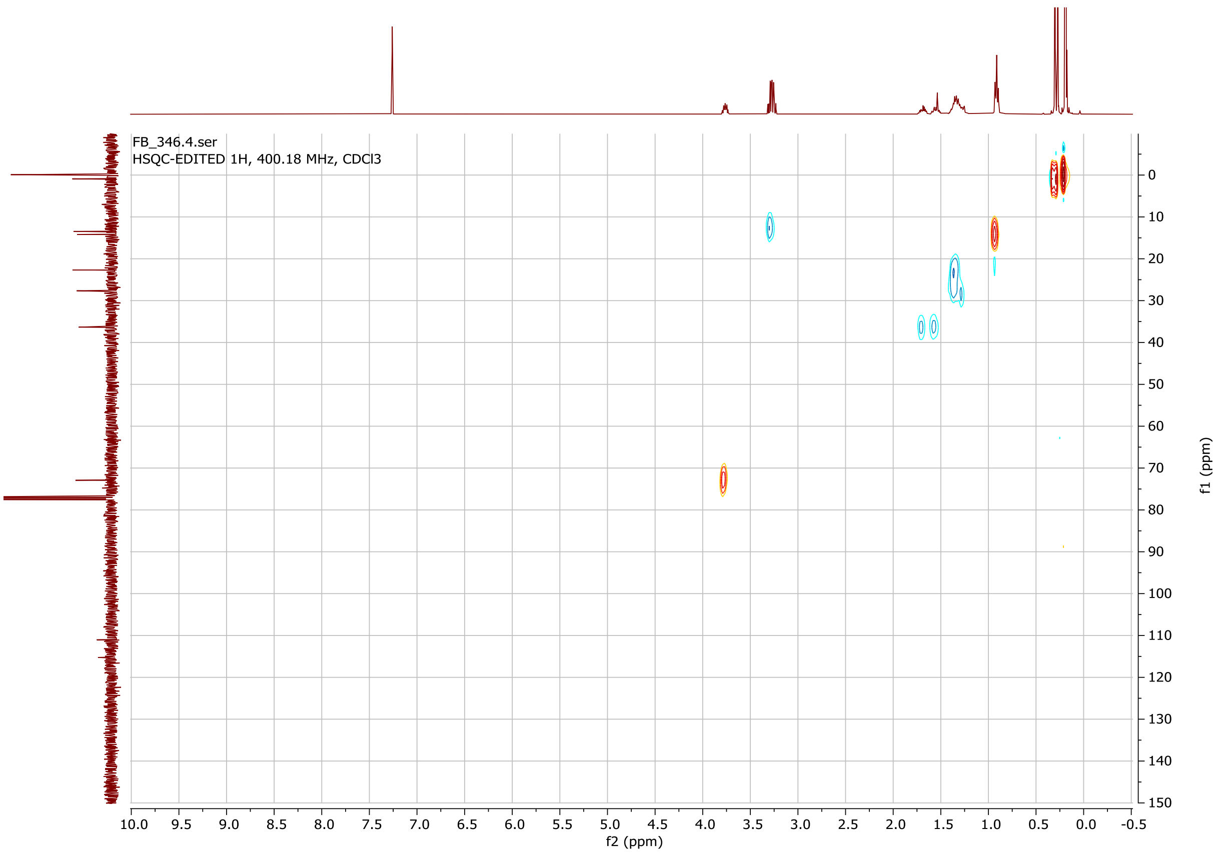
FB_346.4.ser
HSQC-EDITED 1H, 400.18 MHz, CDCl3
f2 (ppm)
f1 (ppm)

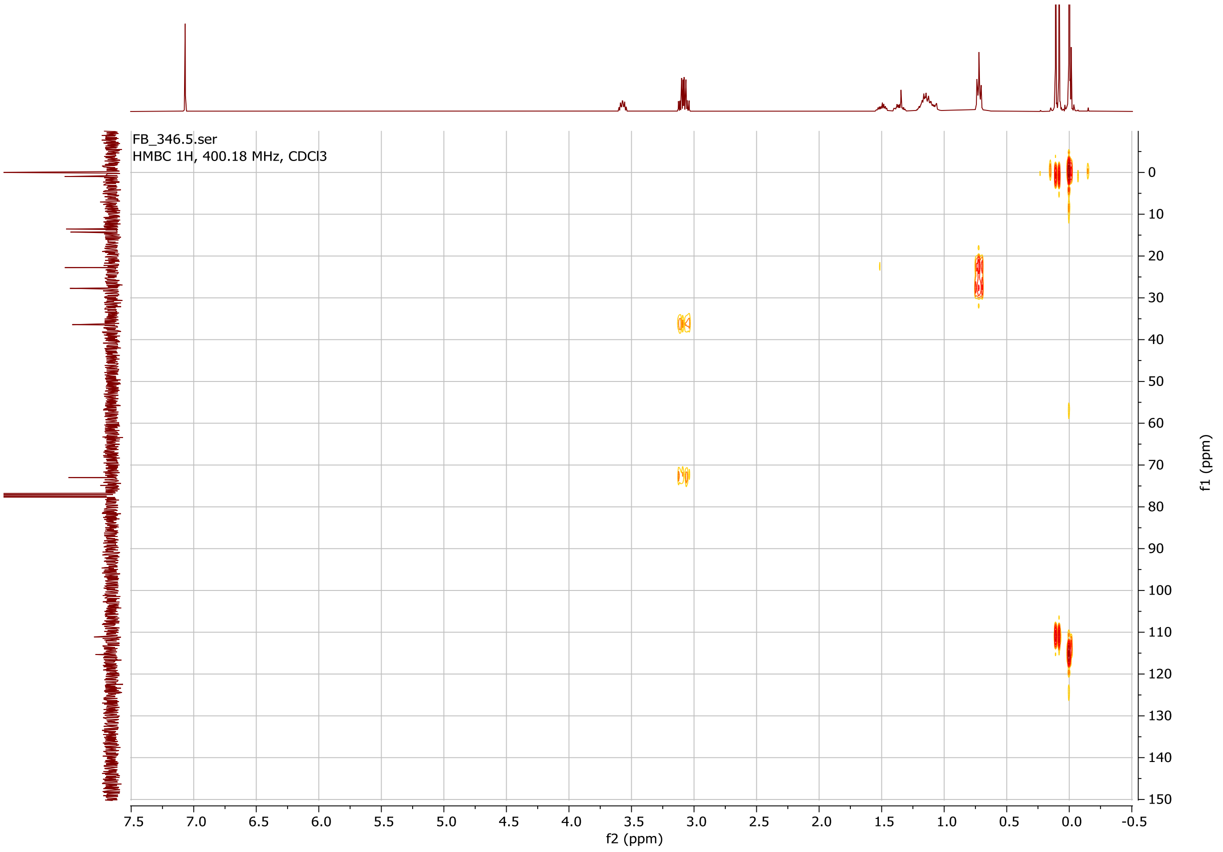

((1-Iodo-3-isopropoxy-1-phenylpropan-2-yl)oxy)dimethyl(trimethylsilyl)ethynylsilane **7s** *(inseparable 62:38 diastereomers mixture)*

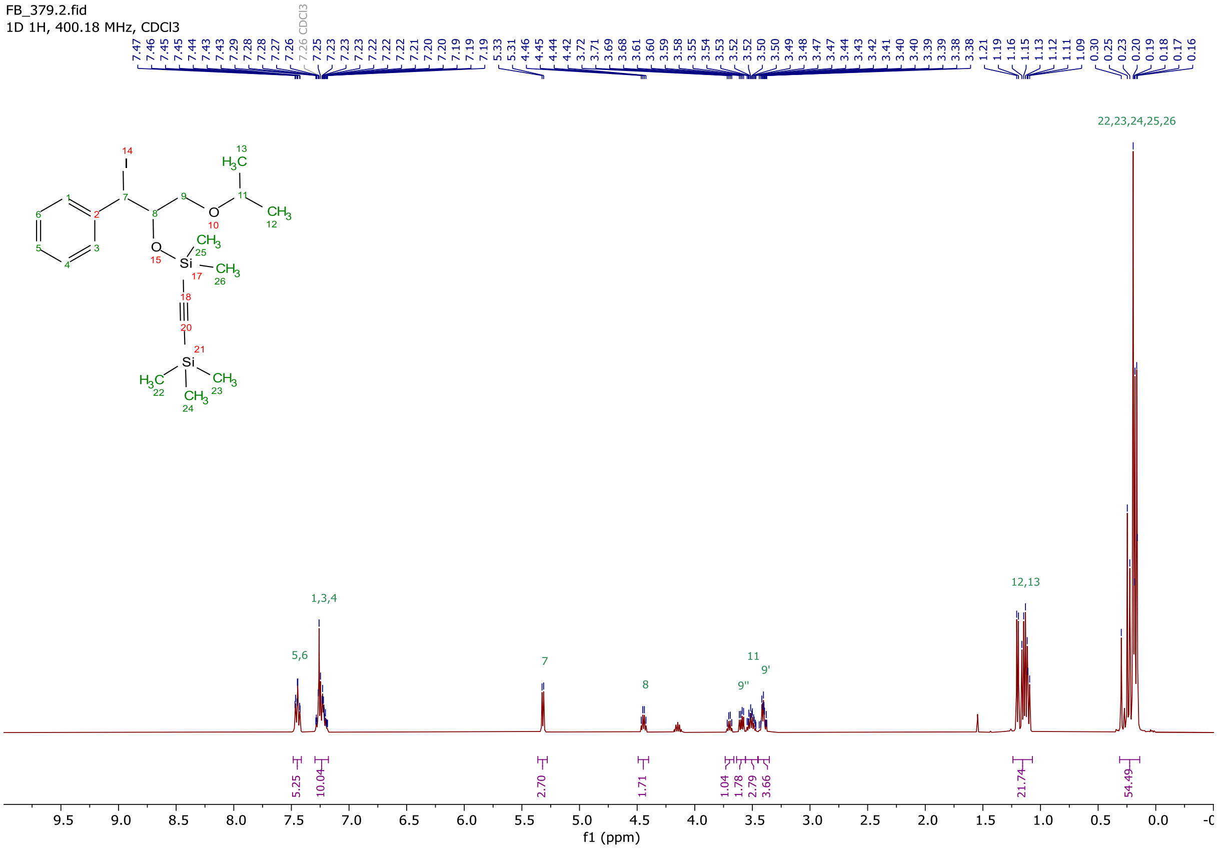

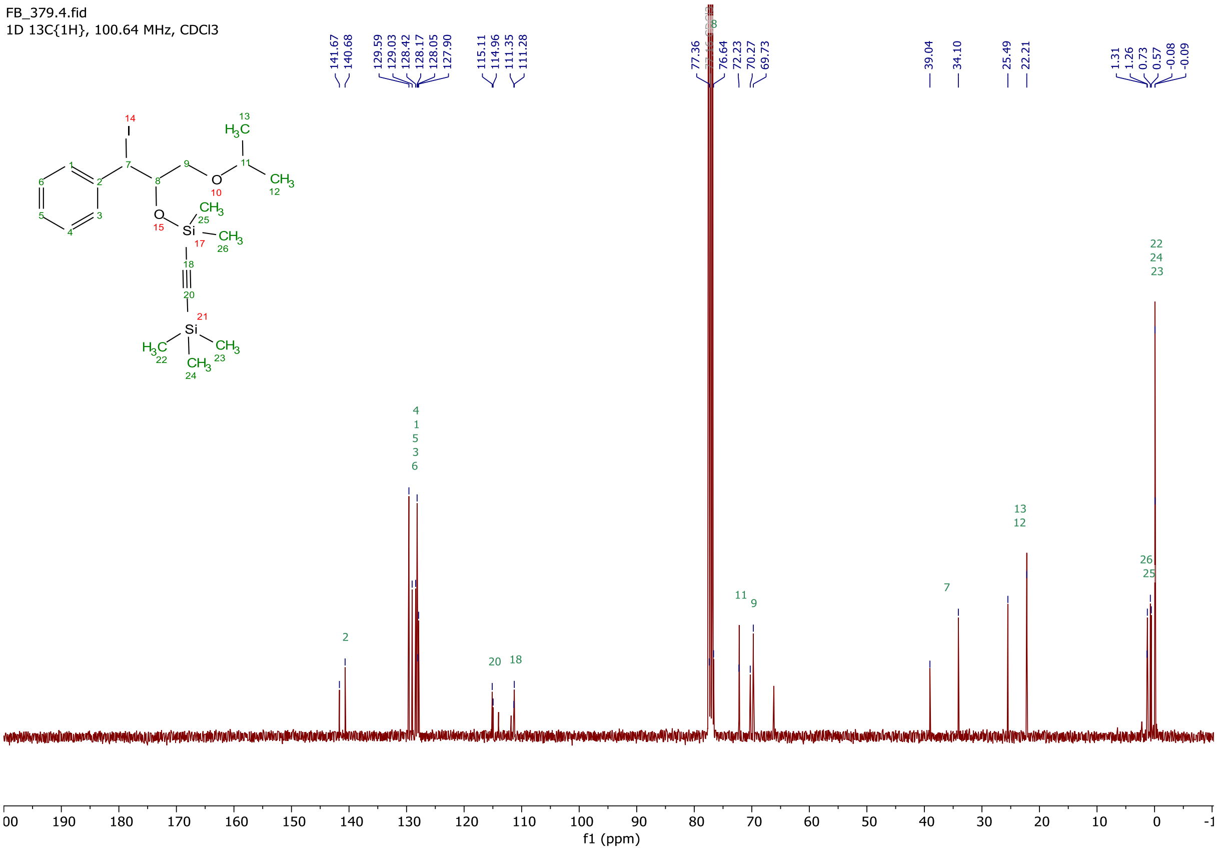
FB_379.4.fid
1D 13C{1H}, 100.64 MHz, CDCl3
141.67
140.68
129.59
129.03
128.42
128.17
128.05
127.90
115.11
114.96
111.35
111.28
77.36
76.64
72.23
70.27
69.73
39.04
34.10
25.49
22.21
1.31
1.26
0.73
0.57
-0.08
-0.09
f1 (ppm)

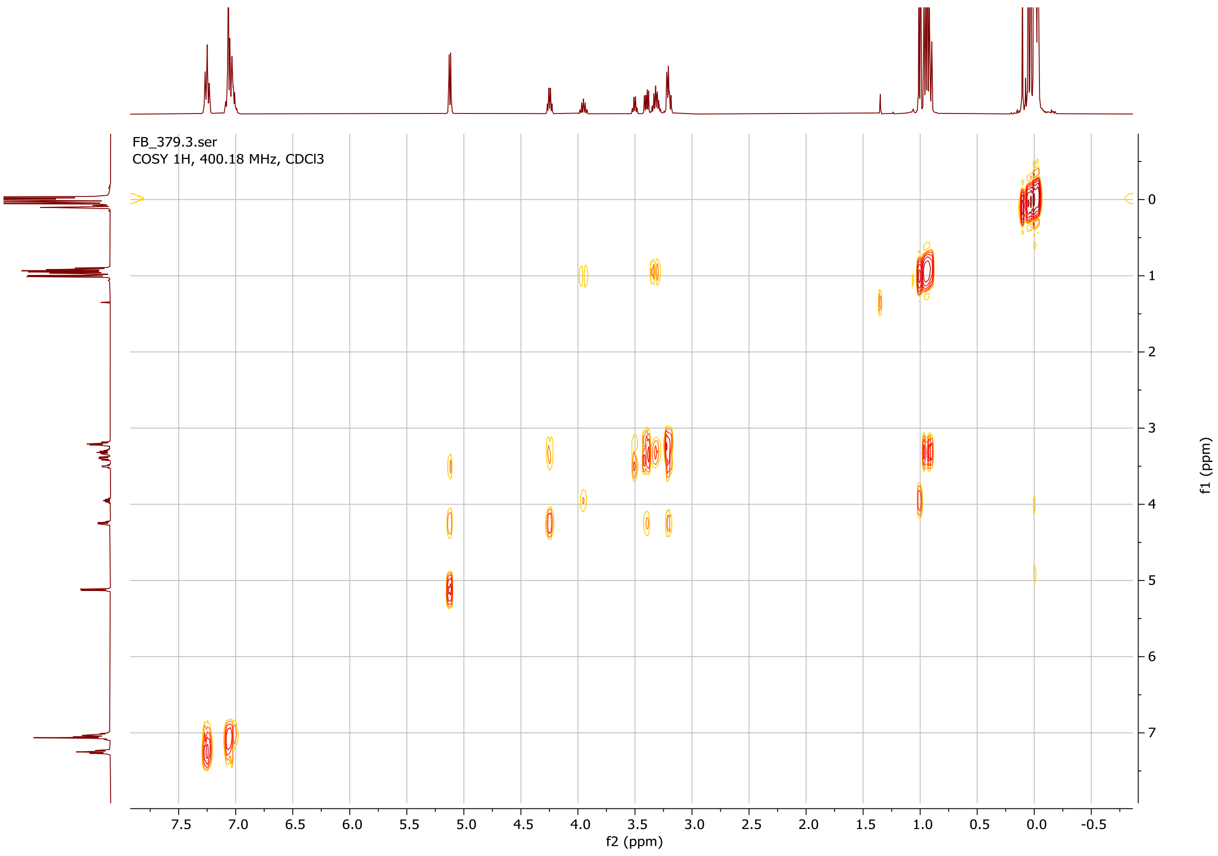
FB_379.3.ser
COSY 1H, 400.18 MHz, CDCl3
f2 (ppm)
f1 (ppm)

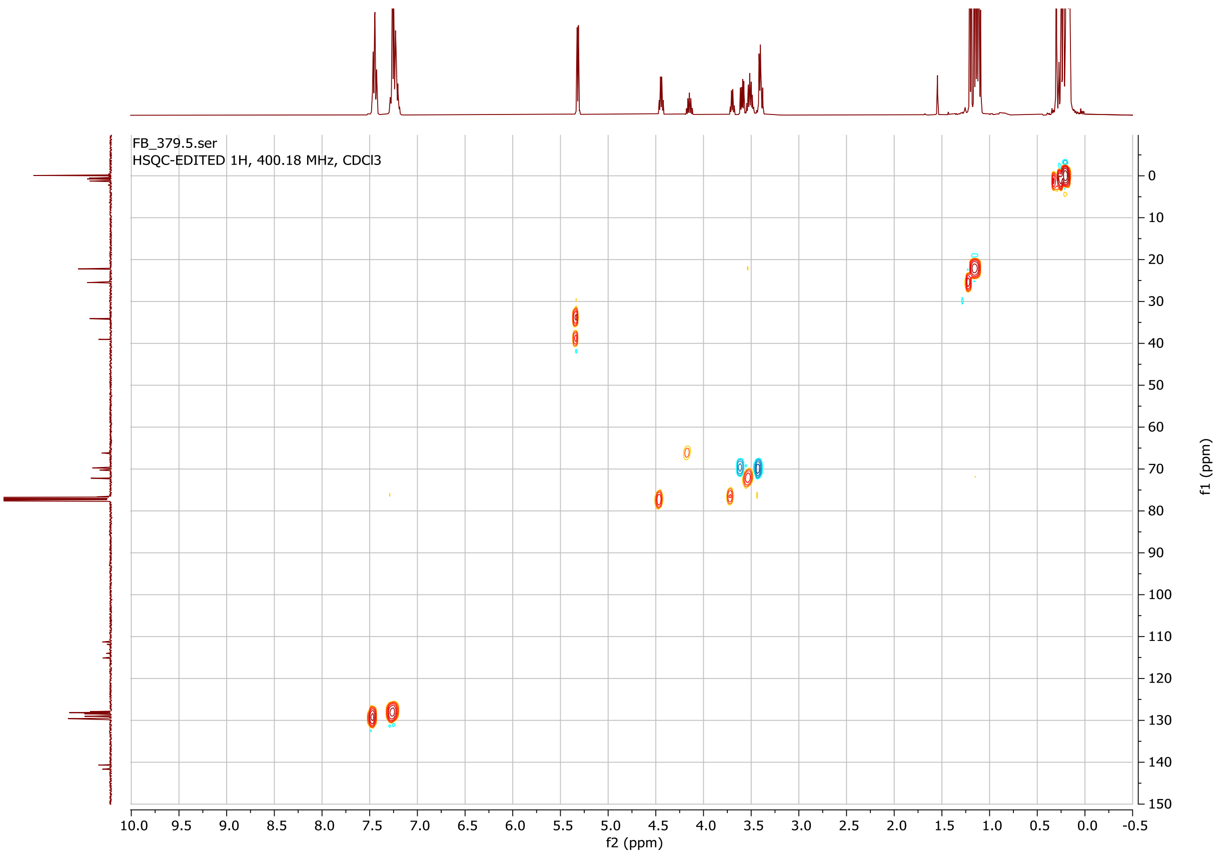
FB_379.5.ser
HSQC-EDITED 1H, 400.18 MHz, CDCl3
f2 (ppm)
f1 (ppm)

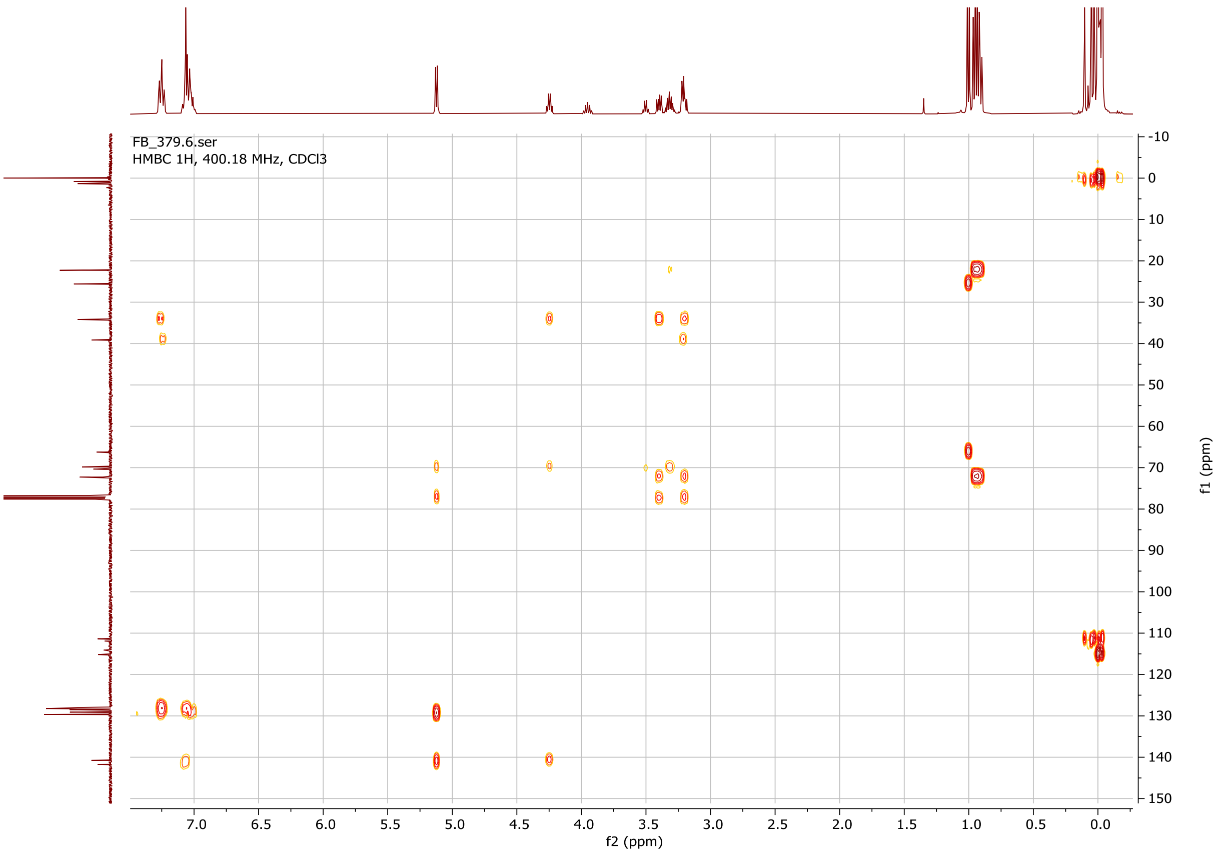
FB_379.6.ser
HMBC 1H, 400.18 MHz, CDCl3
f2 (ppm)
f1 (ppm)

2-Methyl-1-phenyl-4-(trimethylsilyl)but-3-yn-1-ol **8f**

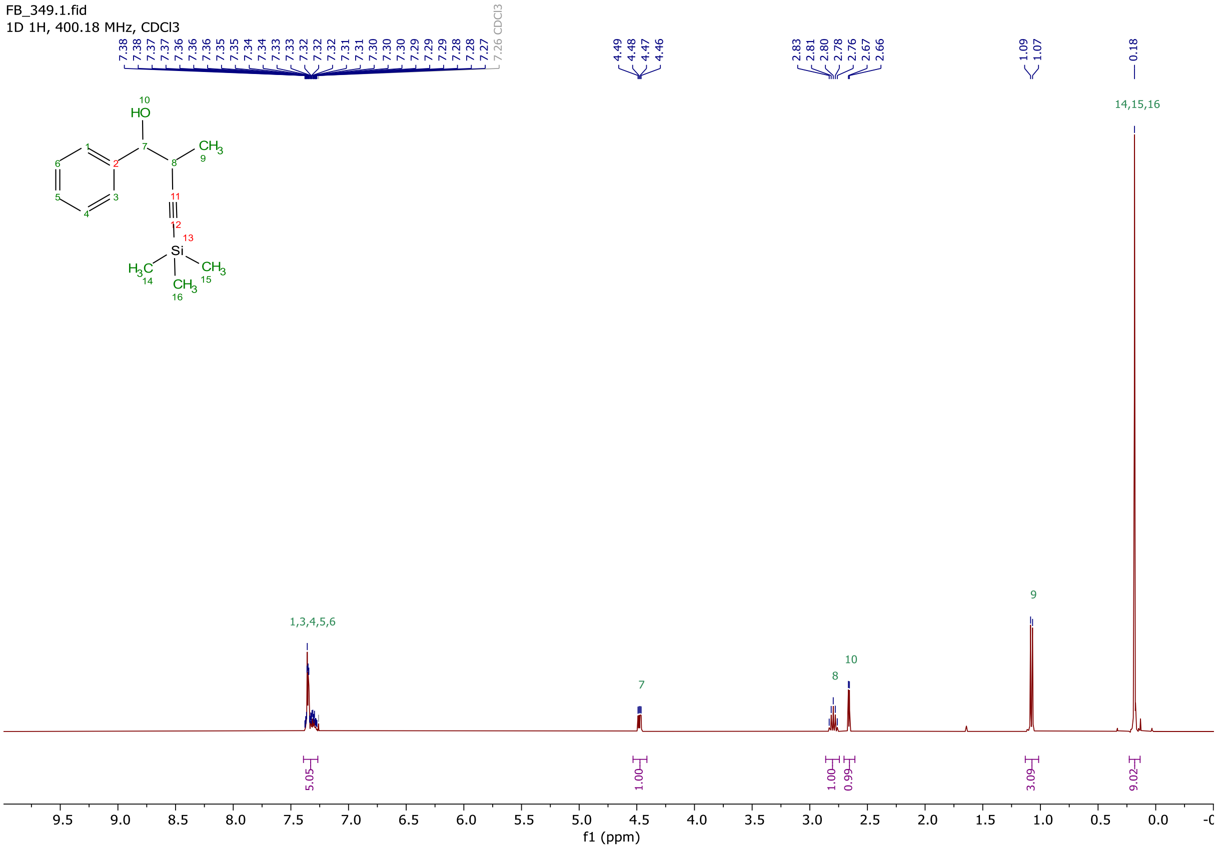

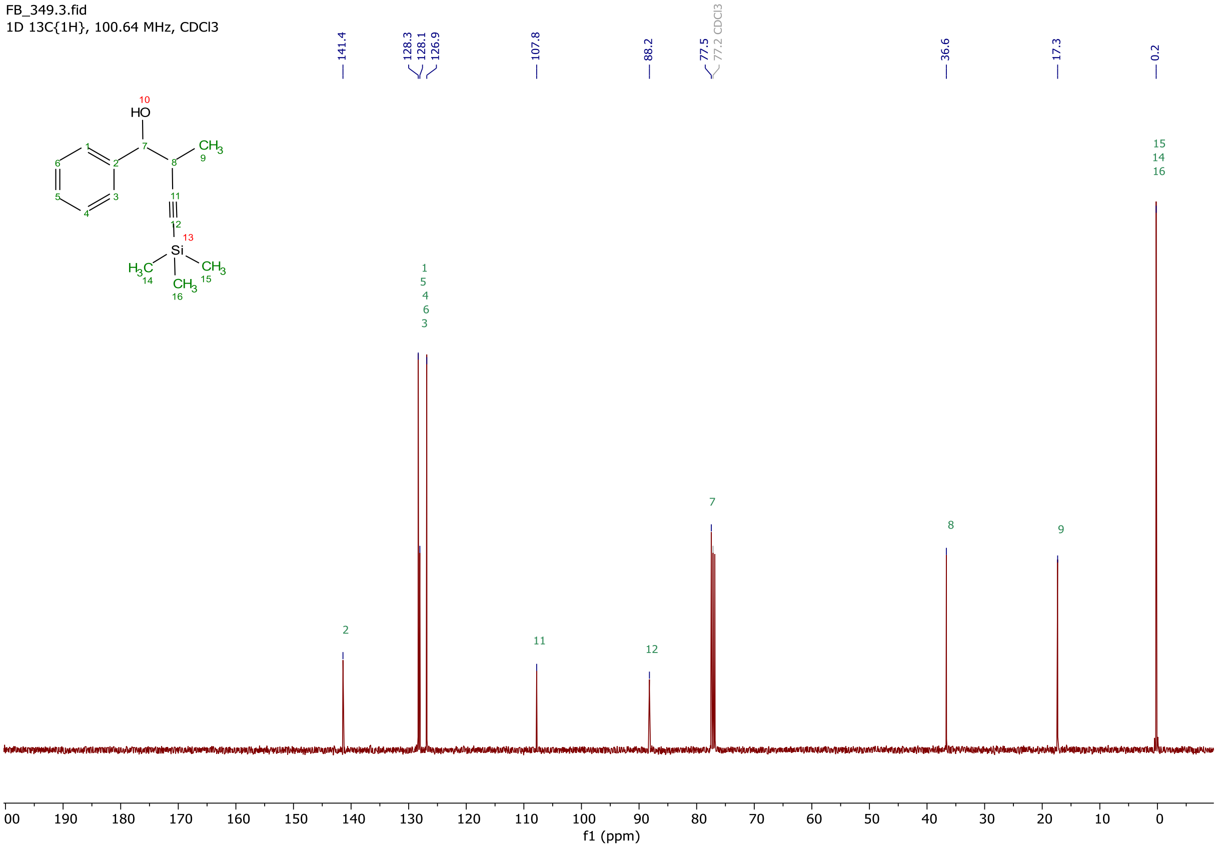

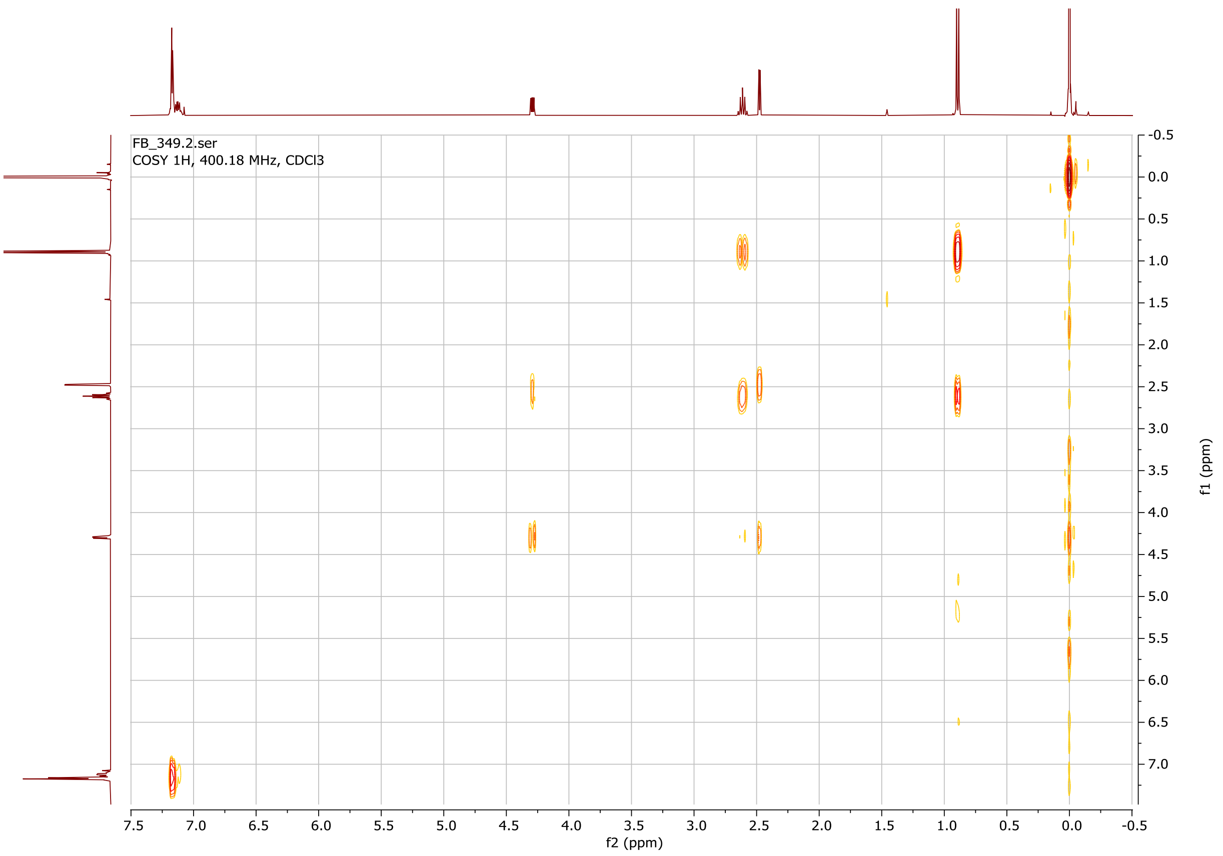
FB_349.2.ser
COSY 1H, 400.18 MHz, CDCl3
f2 (ppm)
f1 (ppm)

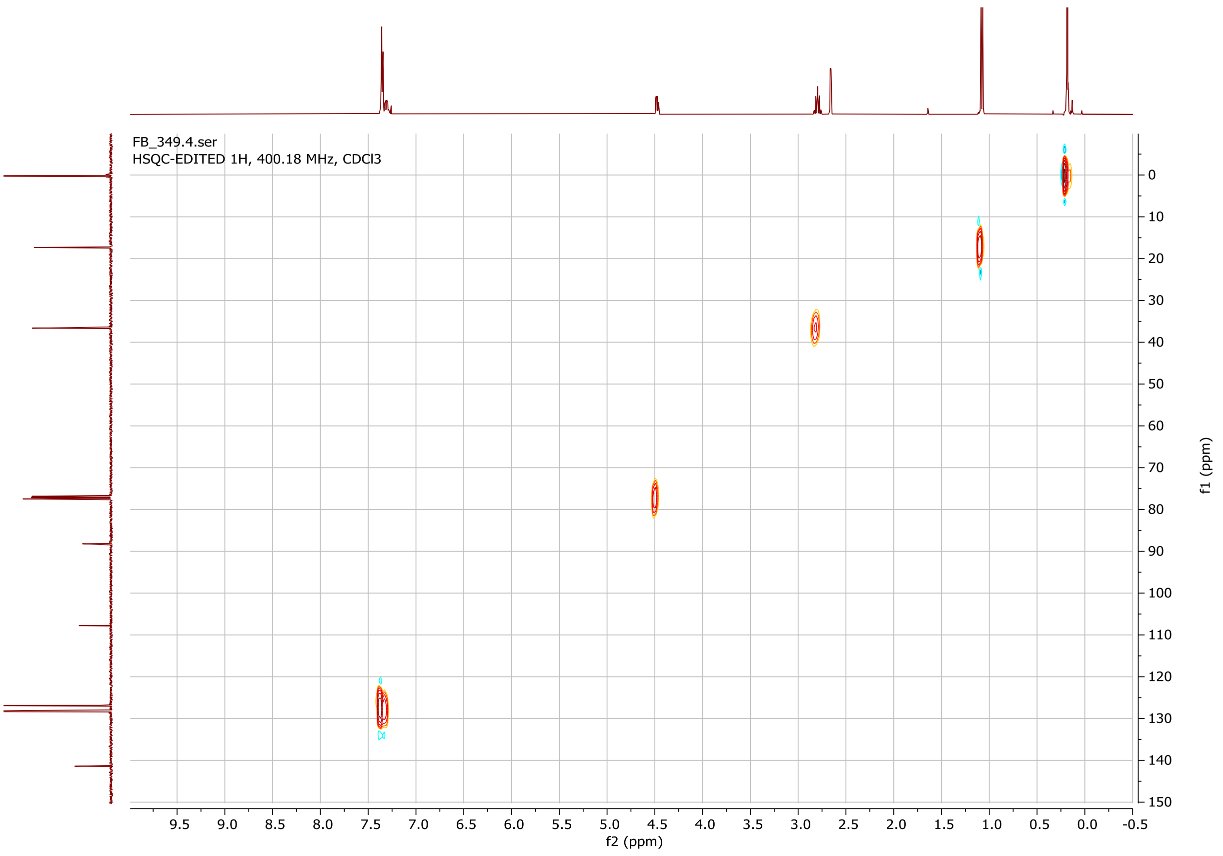
FB_349.4.ser
HSQC-EDITED 1H, 400.18 MHz, CDCl3
f2 (ppm)
f1 (ppm)

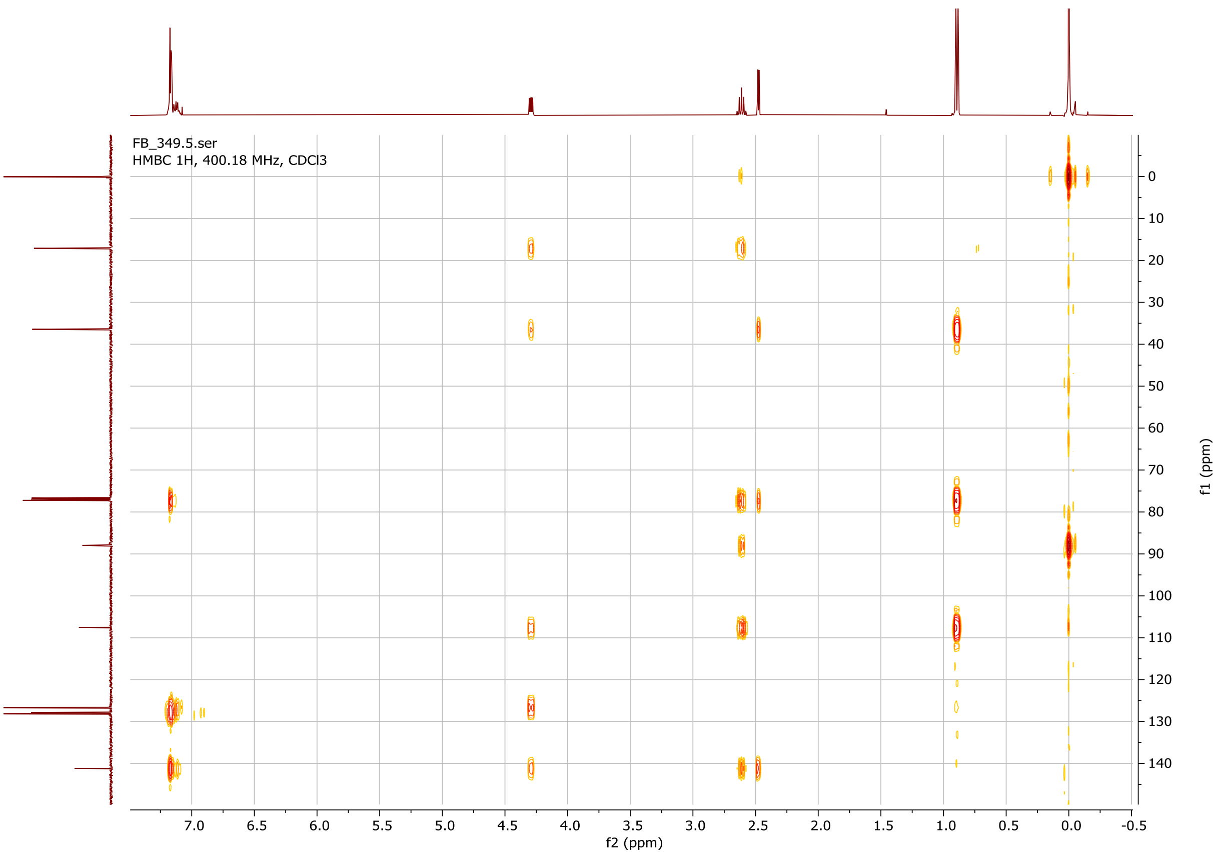

1-(Trimethylsilyl)oct-1-yn-4-ol **8k**

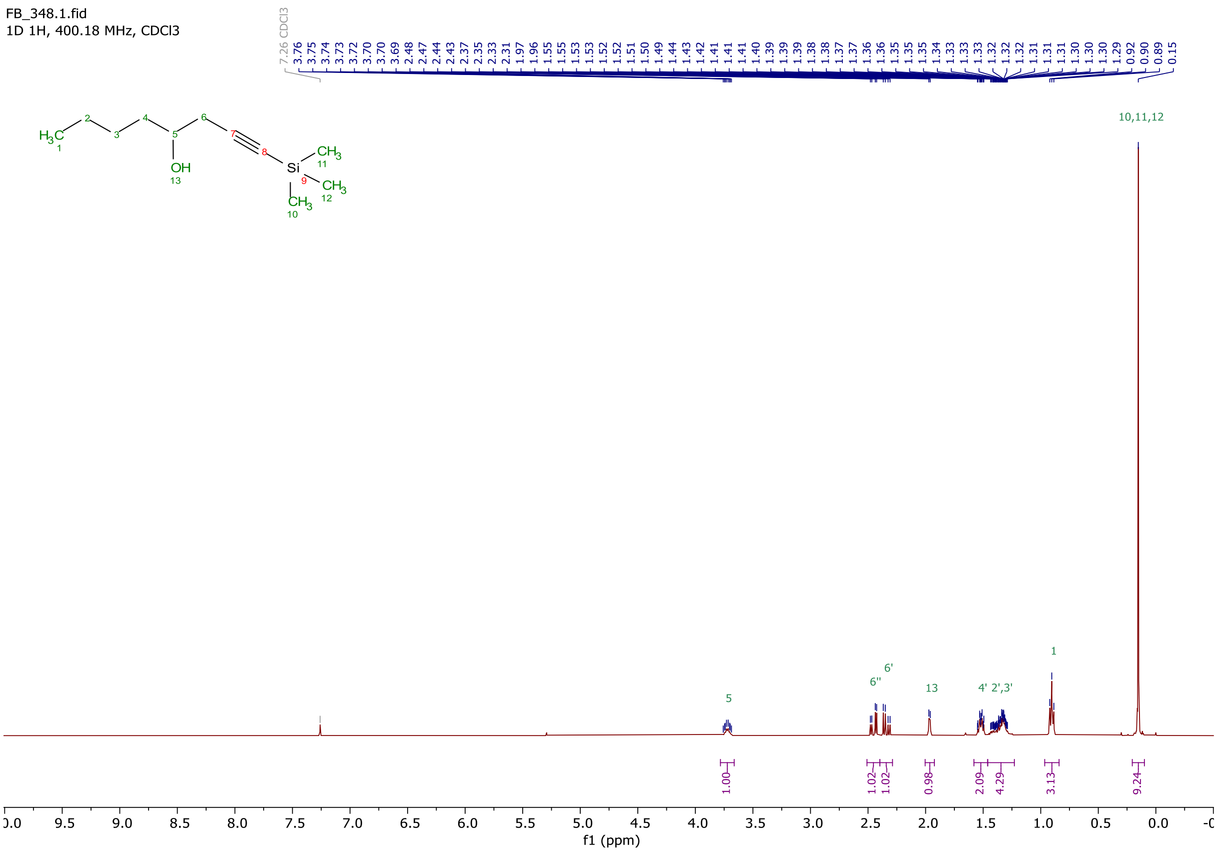

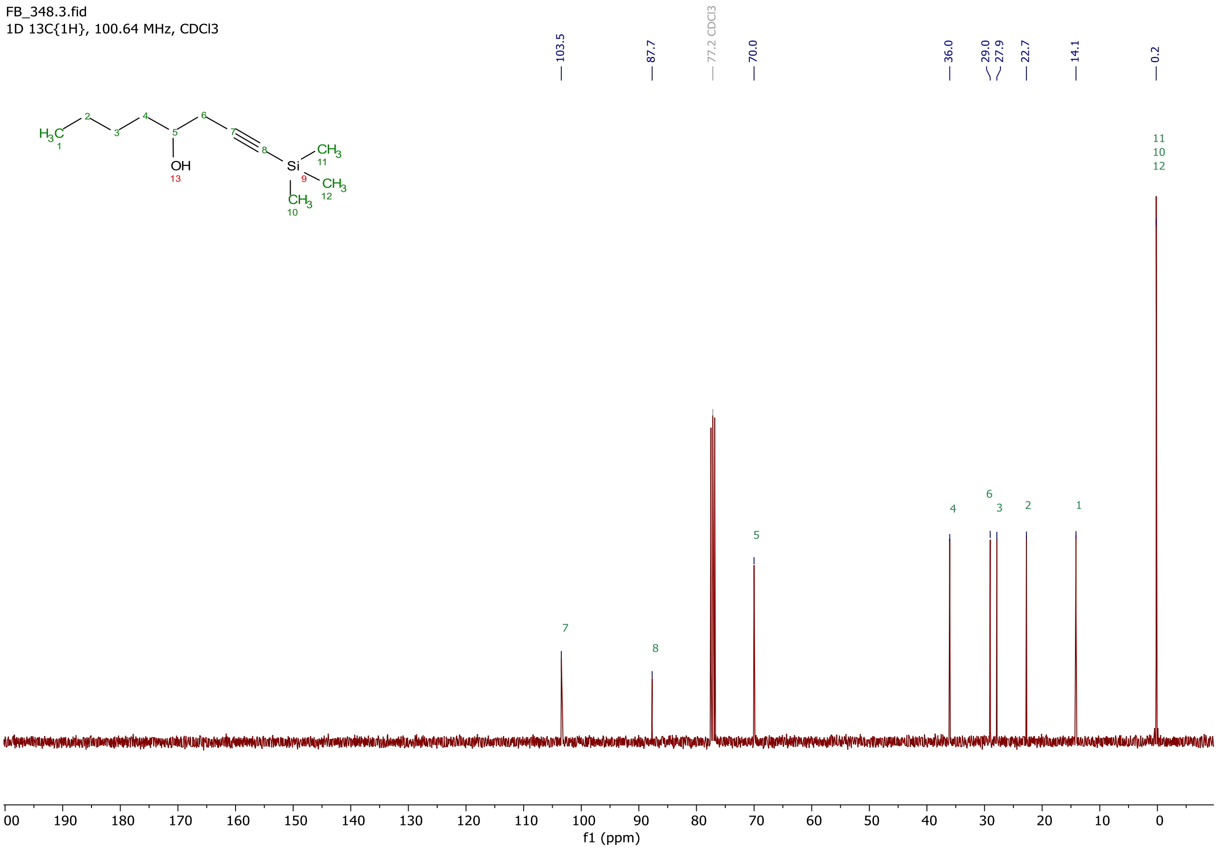

FB_348.3.fid
1D 13C{1H}, 100.64 MHz, CDCl3
103.5
87.7
77.2 CDCl3
70.0
36.0
29.0
27.9
22.7
14.1
0.2
H3C
OH
Si
CH3
f1 (ppm)

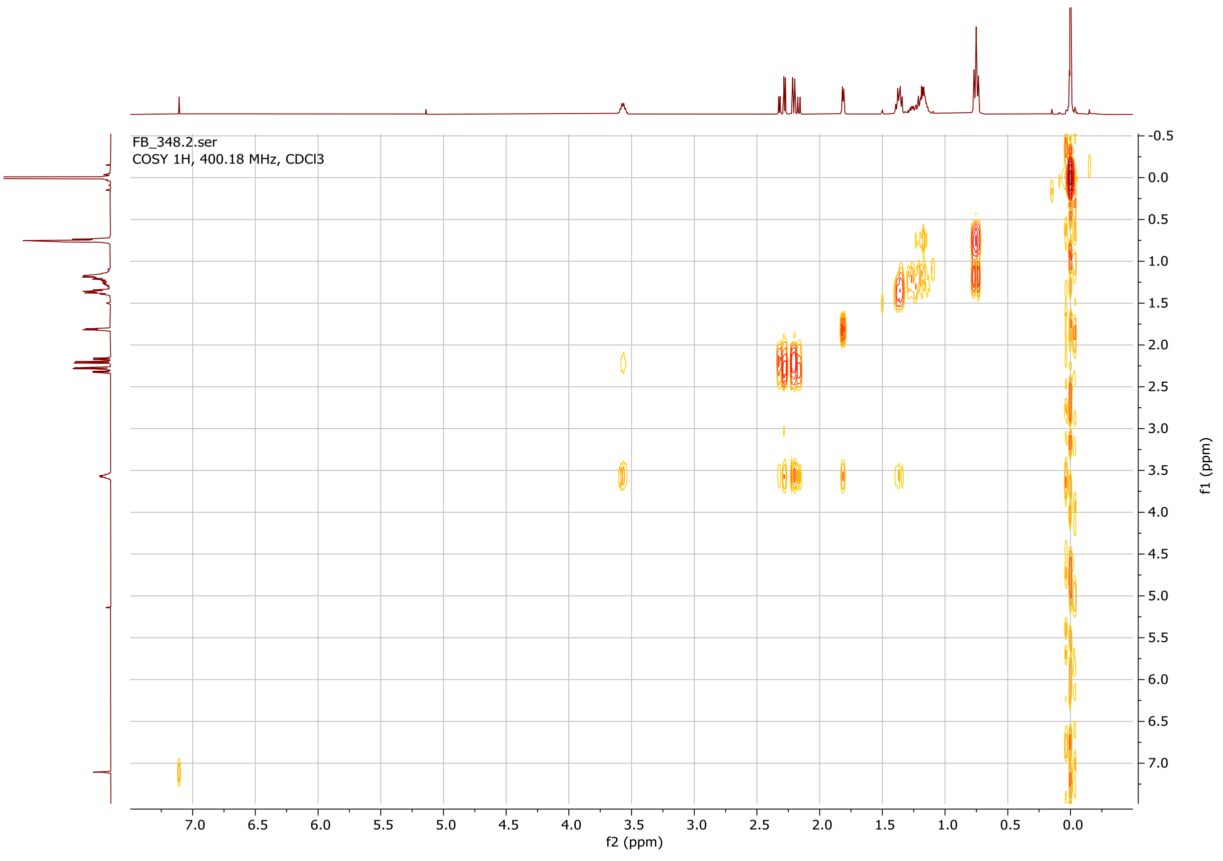

FB_348.2.ser
COSY 1H, 400.18 MHz, CDCl3
f2 (ppm)
f1 (ppm)

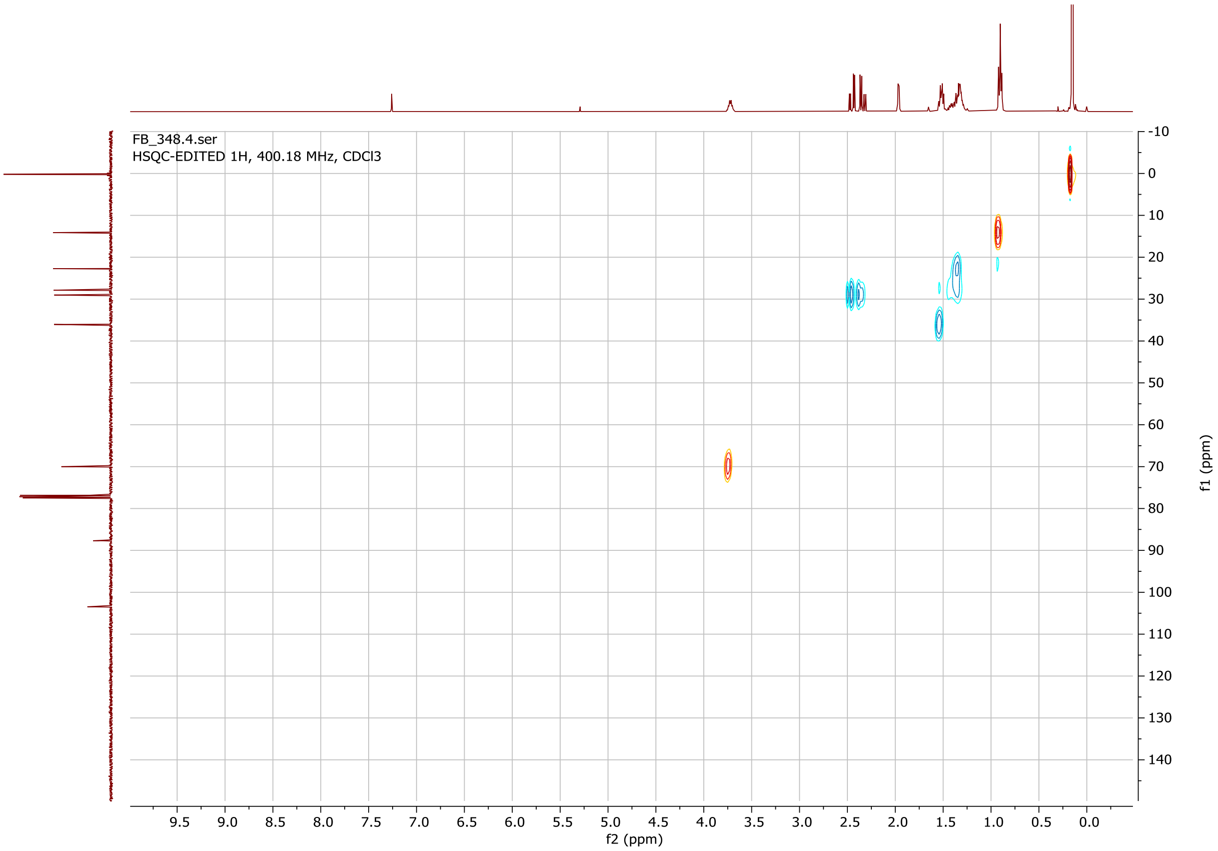

FB_348.4.ser
HSQC-EDITED 1H, 400.18 MHz, CDCl3
f2 (ppm)
f1 (ppm)

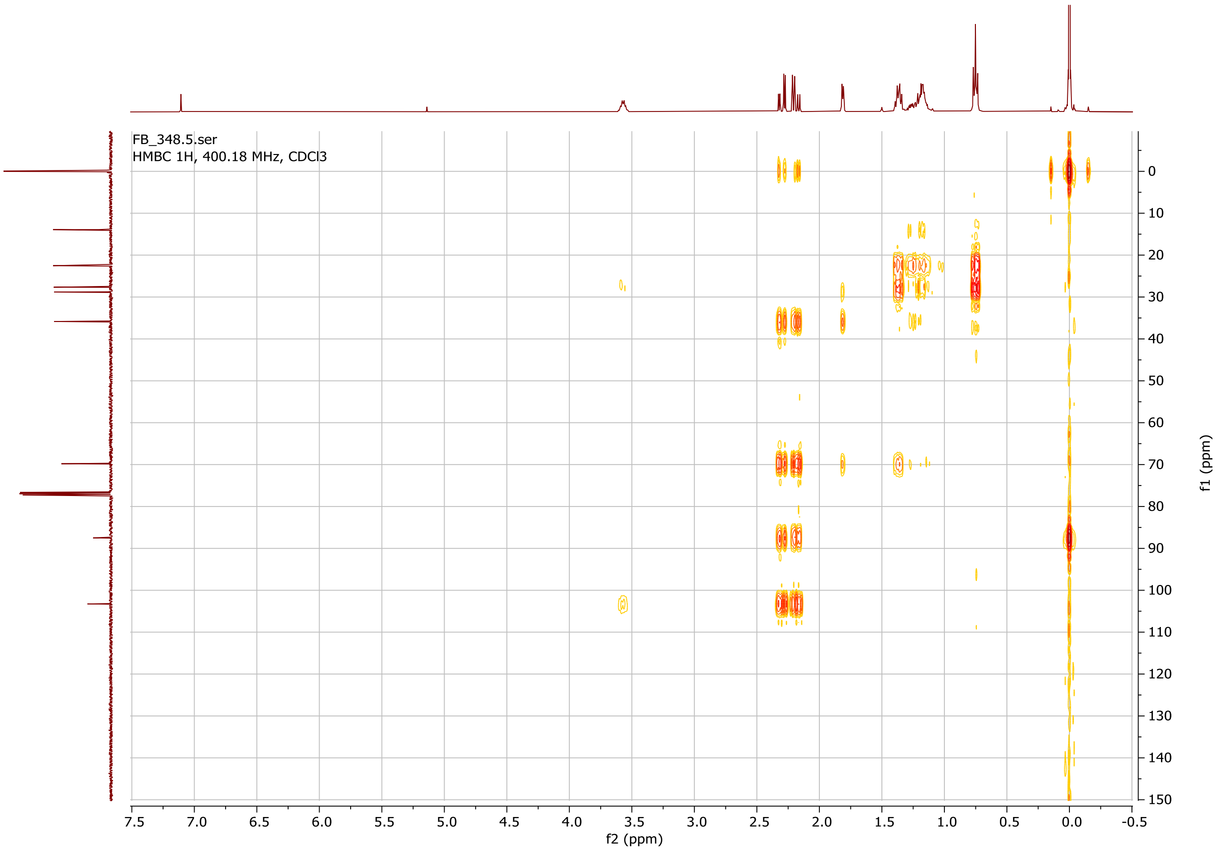

FB_348.5.ser
HMBC 1H, 400.18 MHz, CDCl3
f2 (ppm)
f1 (ppm)

1-Isopropoxy-3-phenyl-5-(trimethylsilylpent-4-yn-2-ol **8s**

FB_280_2-3.1.fid
1D 1H, 400.18 MHz, CDCl3

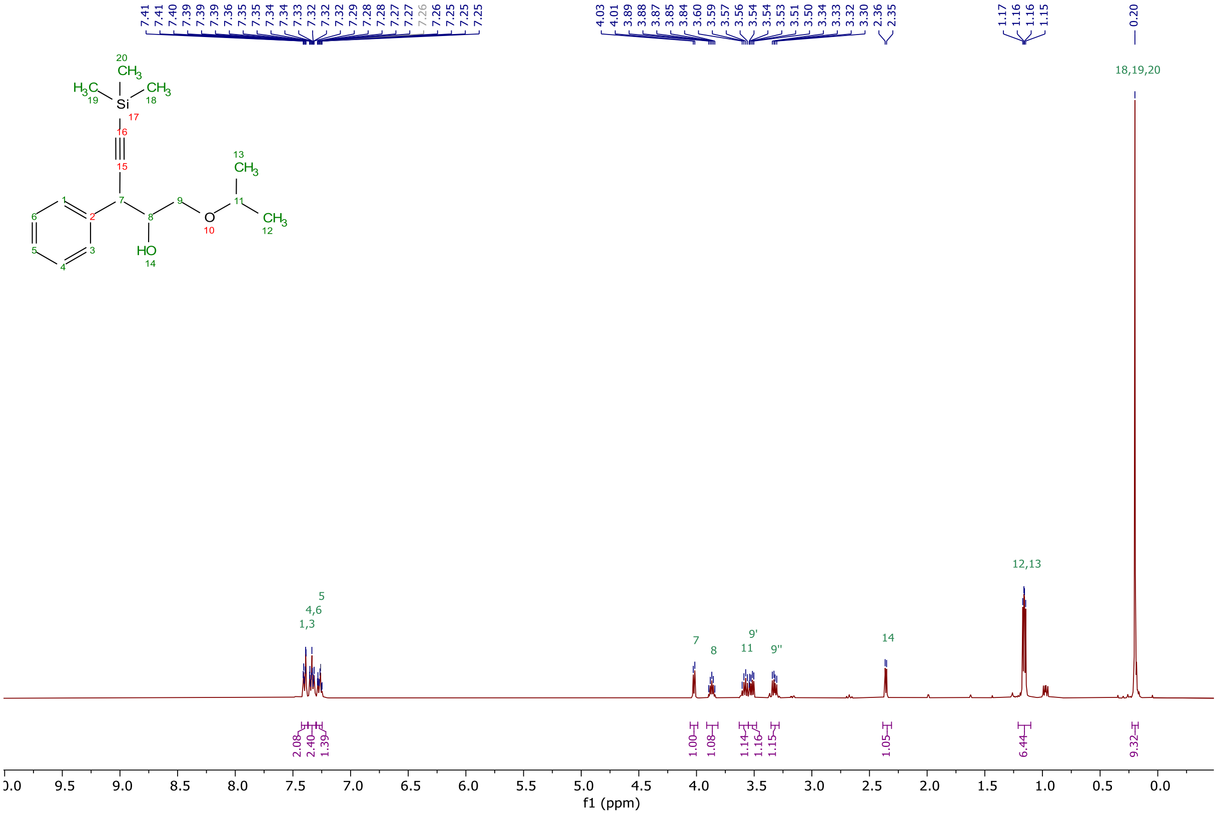

FB_280_2-3.3.fid
1D 13C{1H}, 100.64 MHz, CDCl3

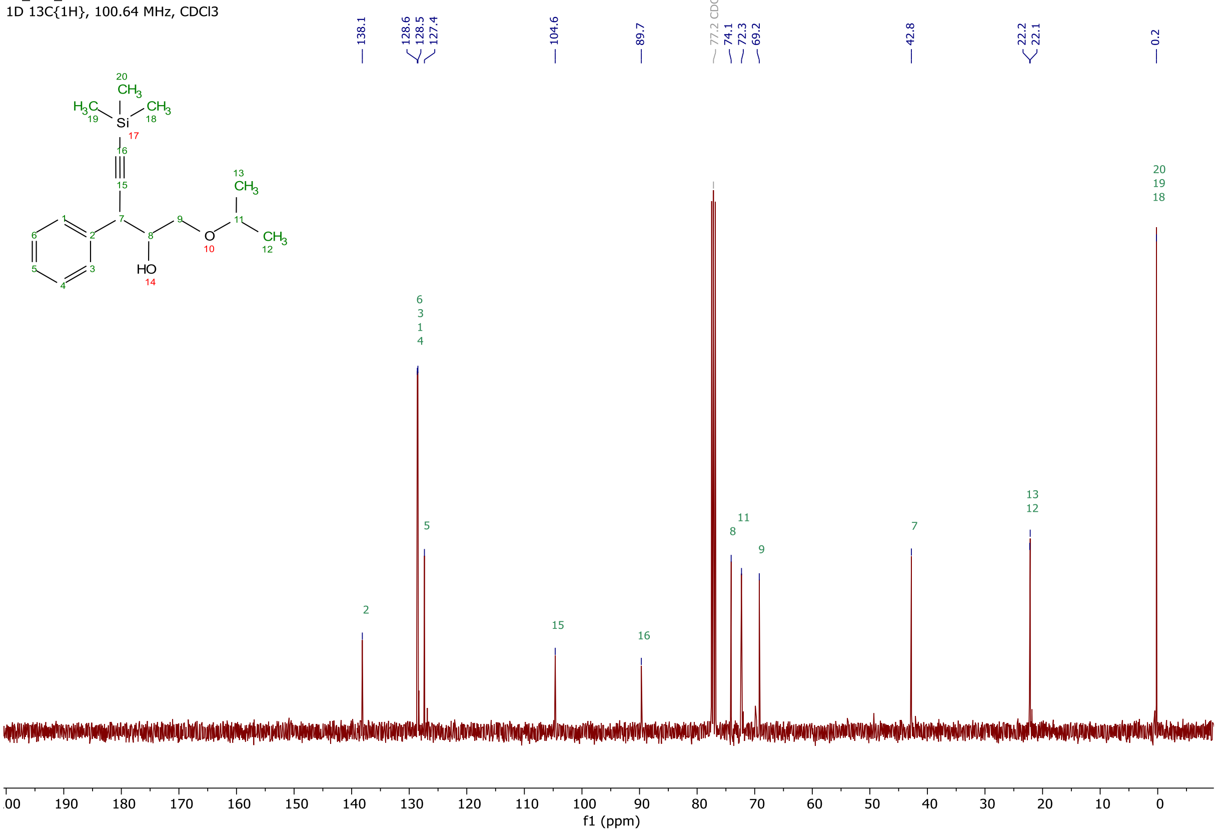

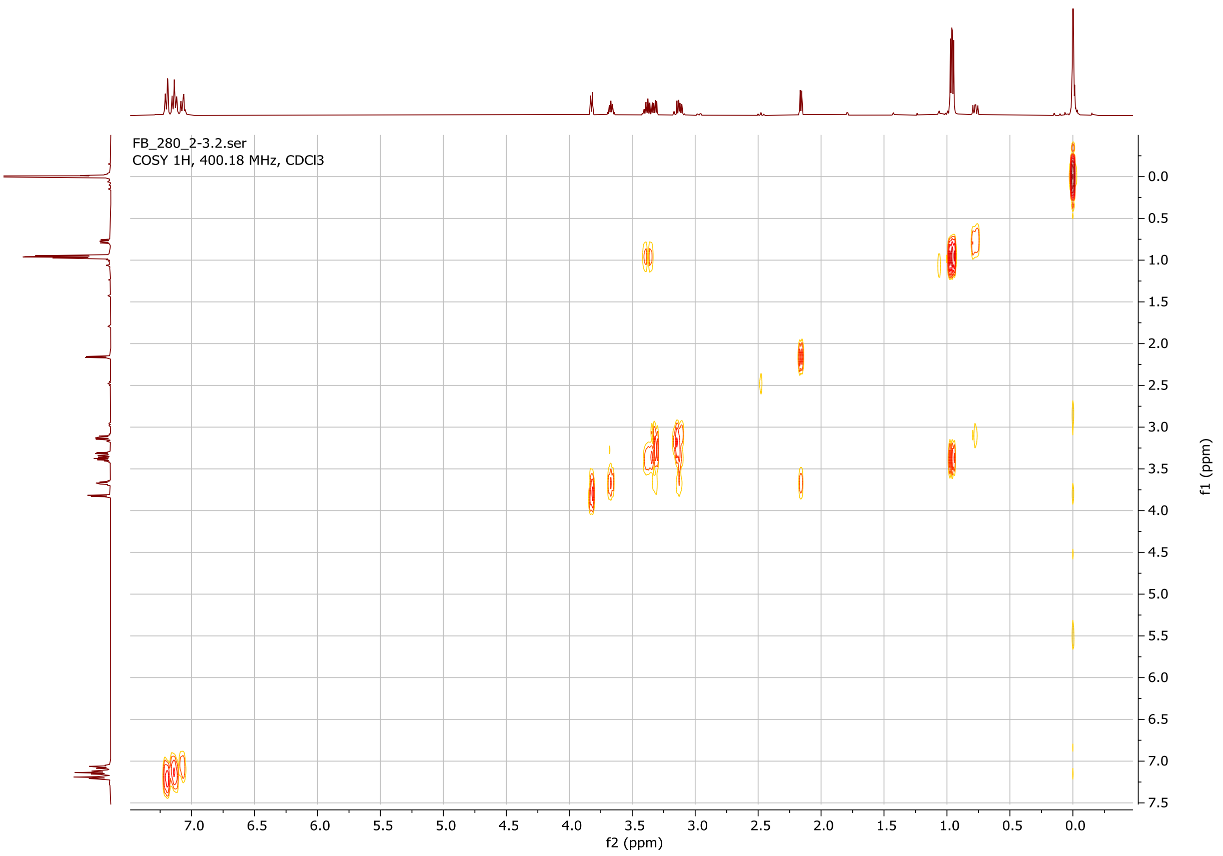
FB_280_2-3.2.ser
COSY 1H, 400.18 MHz, CDCl3
f2 (ppm)
f1 (ppm)

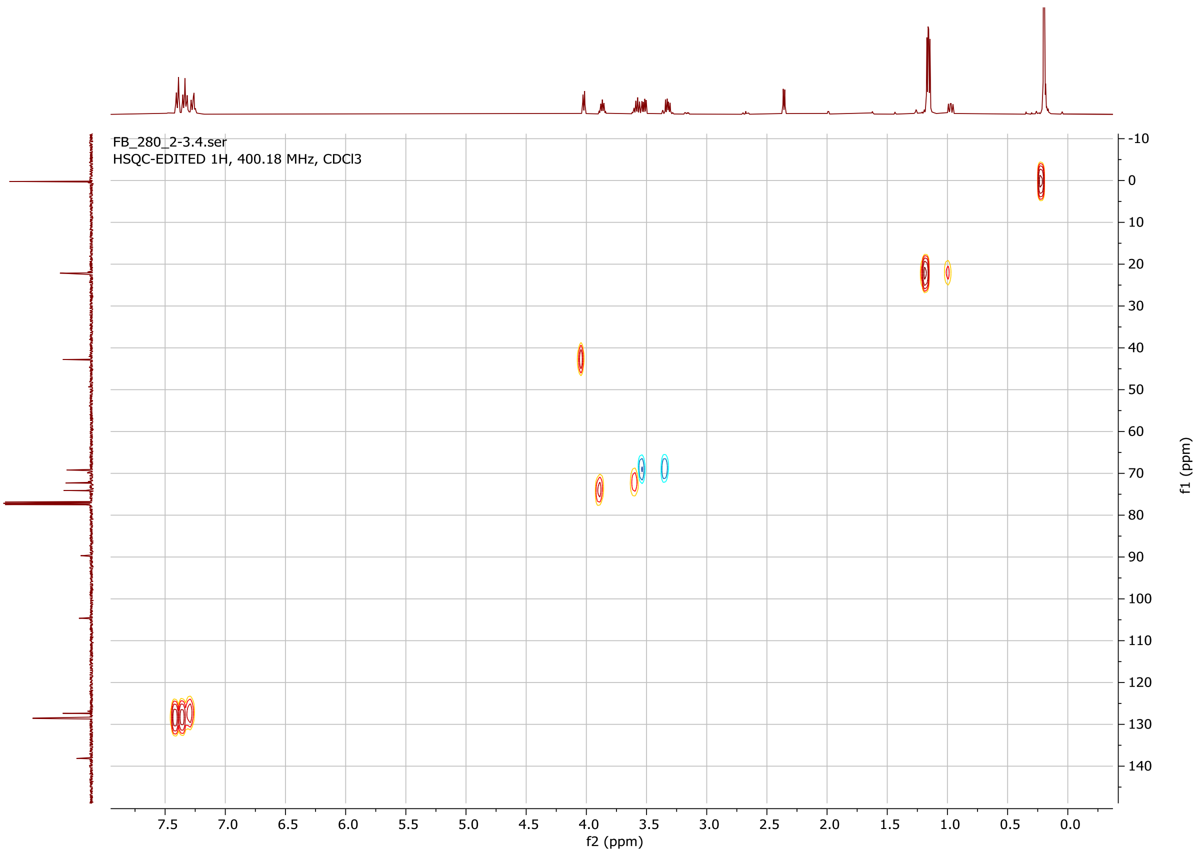
FB_280_2-3.4.ser
HSQC-EDITED 1H, 400.18 MHz, CDCl3
f2 (ppm)
f1 (ppm)

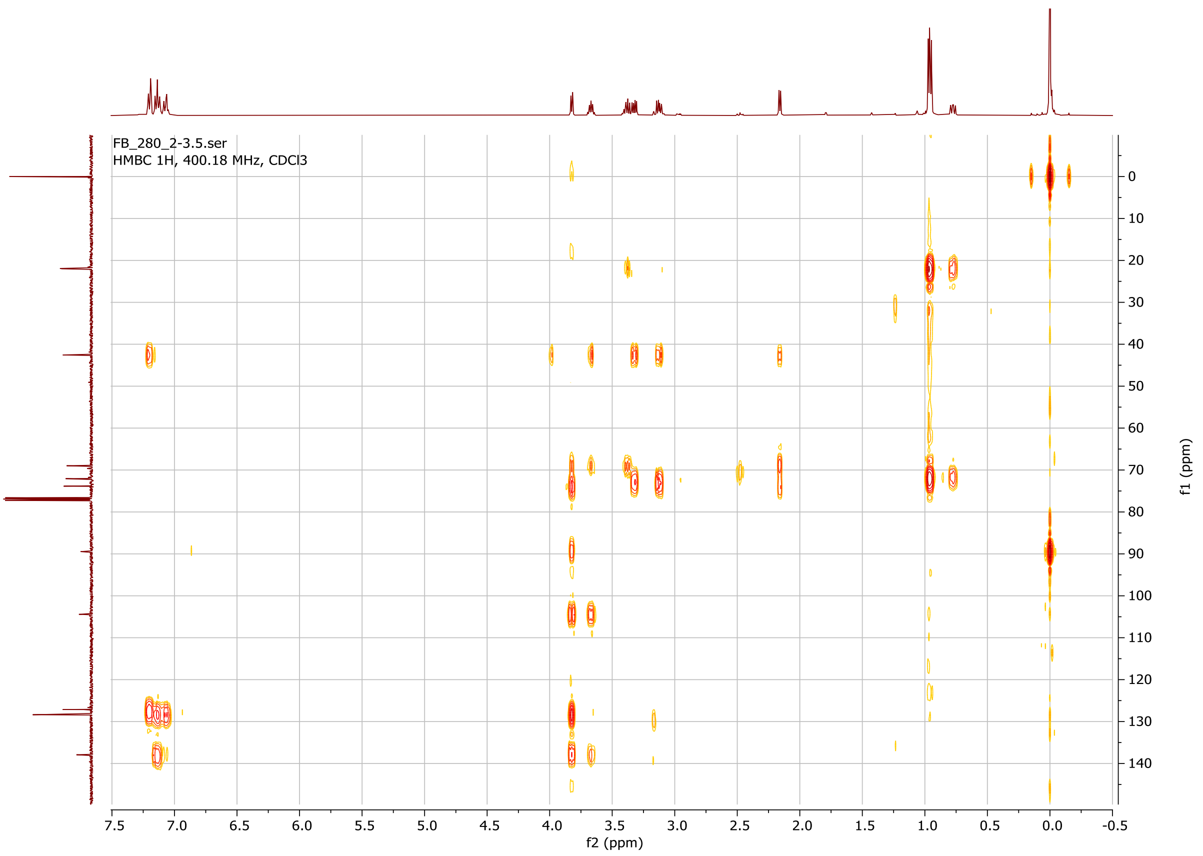

1-(Allyloxy)-2-iodo-2,3-dihydro-1*H*-indene **9**

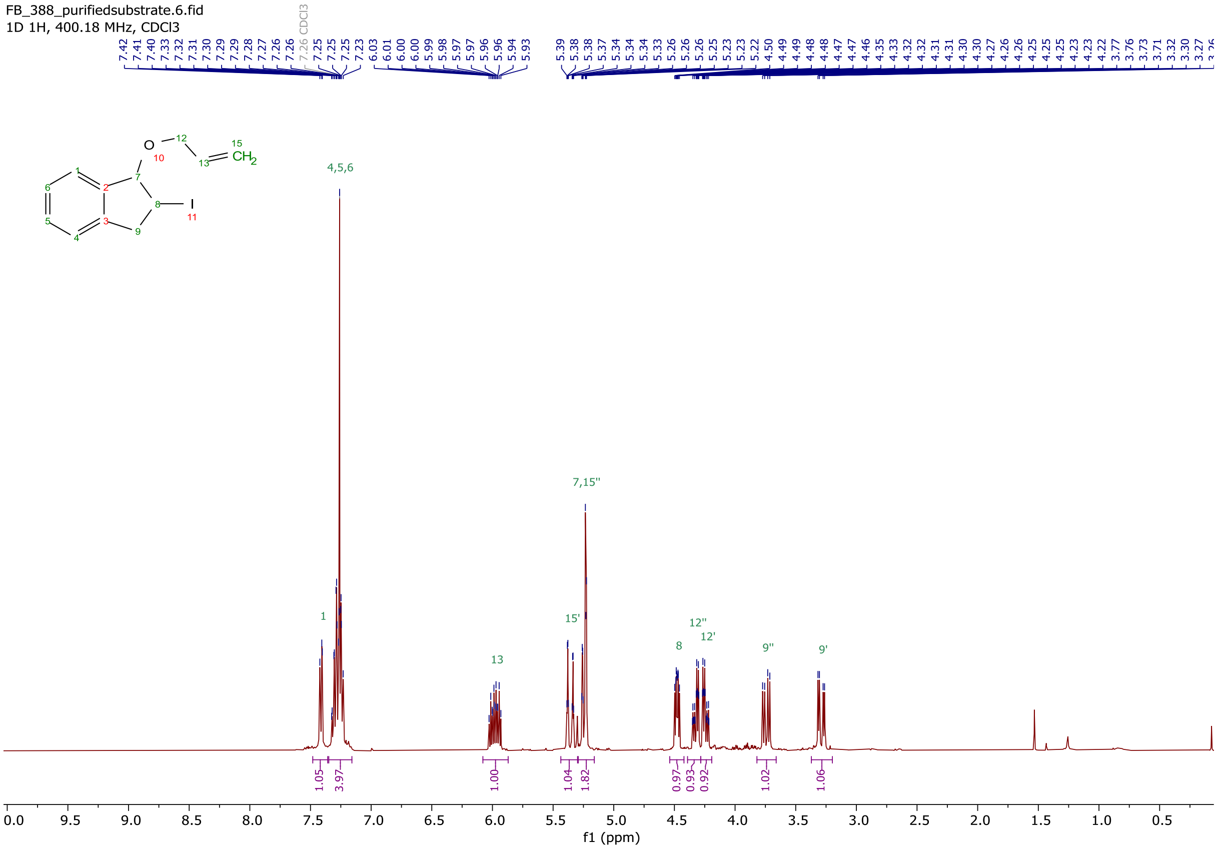

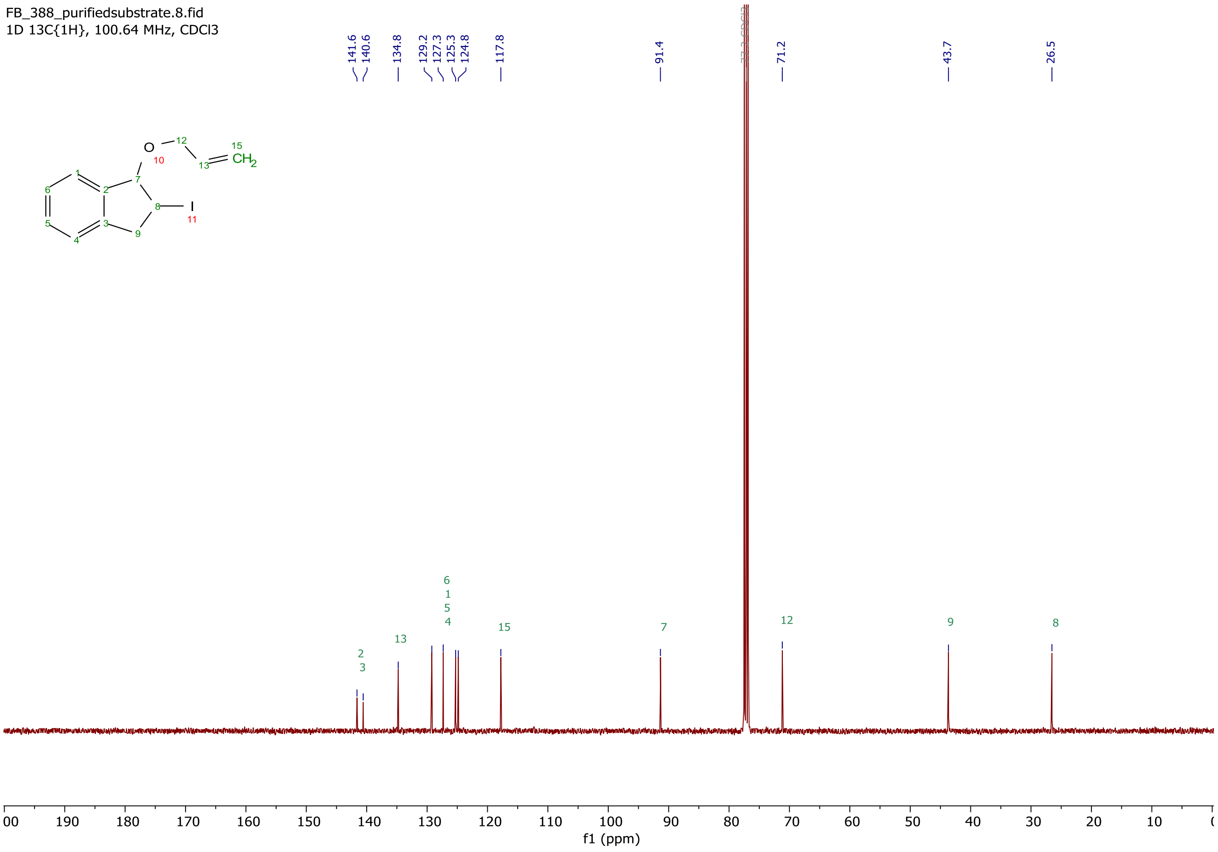
FB_388_purifiedsubstrate.8.fid
1D 13C{1H}, 100.64 MHz, CDCl3
141.6
140.6
134.8
129.2
127.3
125.3
124.8
117.8
91.4
71.2
43.7
26.5
f1 (ppm)

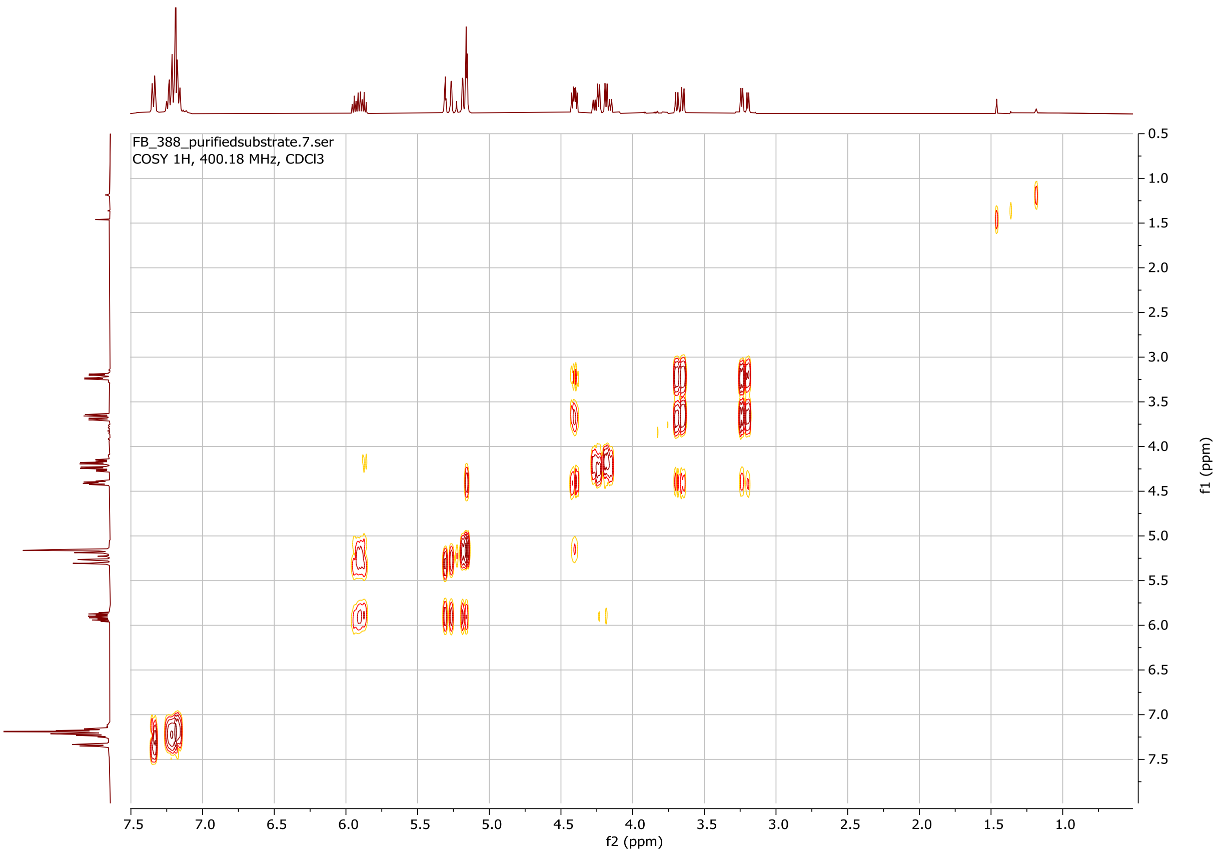
FB_388_purifiedsubstrate.7.ser
COSY 1H, 400.18 MHz, CDCl3
f2 (ppm)
f1 (ppm)

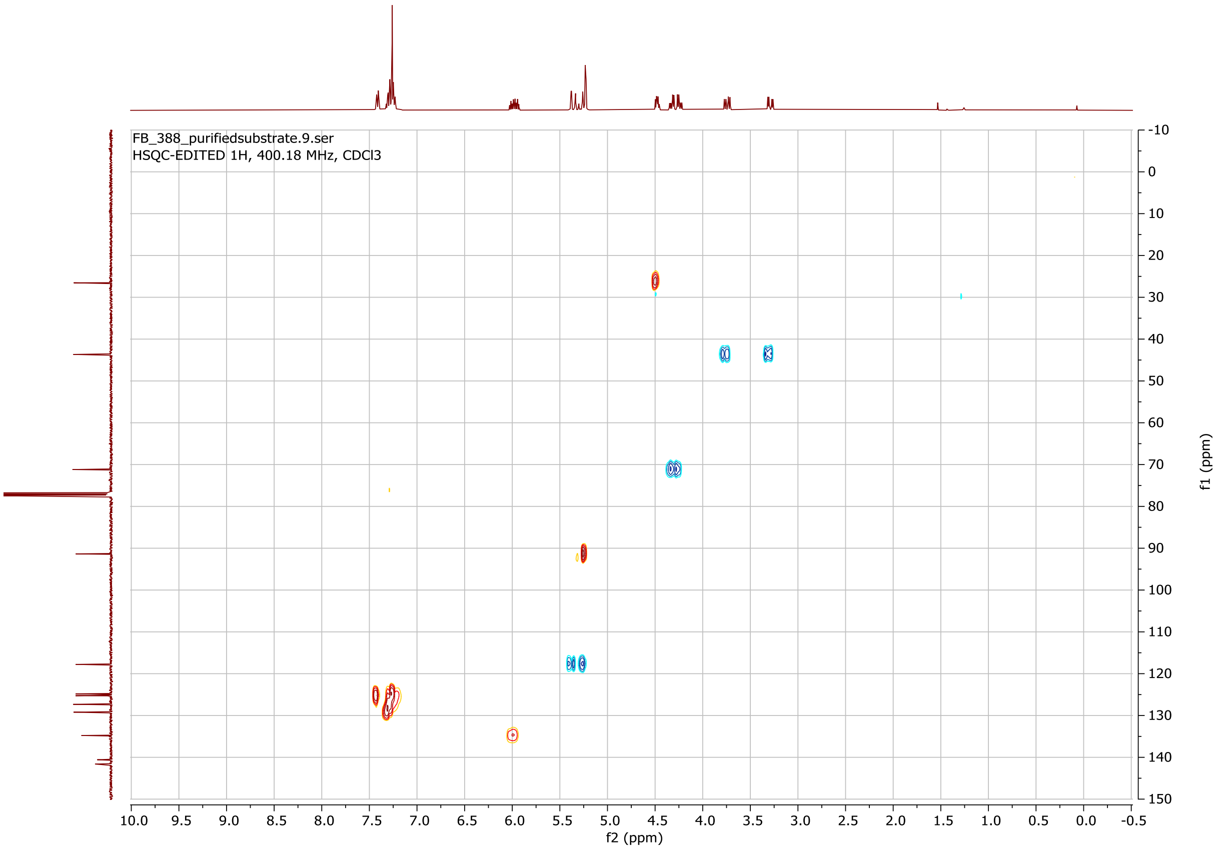
FB_388_purifiedsubstrate.9.ser
HSQC-EDITED 1H, 400.18 MHz, CDCl3
f2 (ppm)
f1 (ppm)

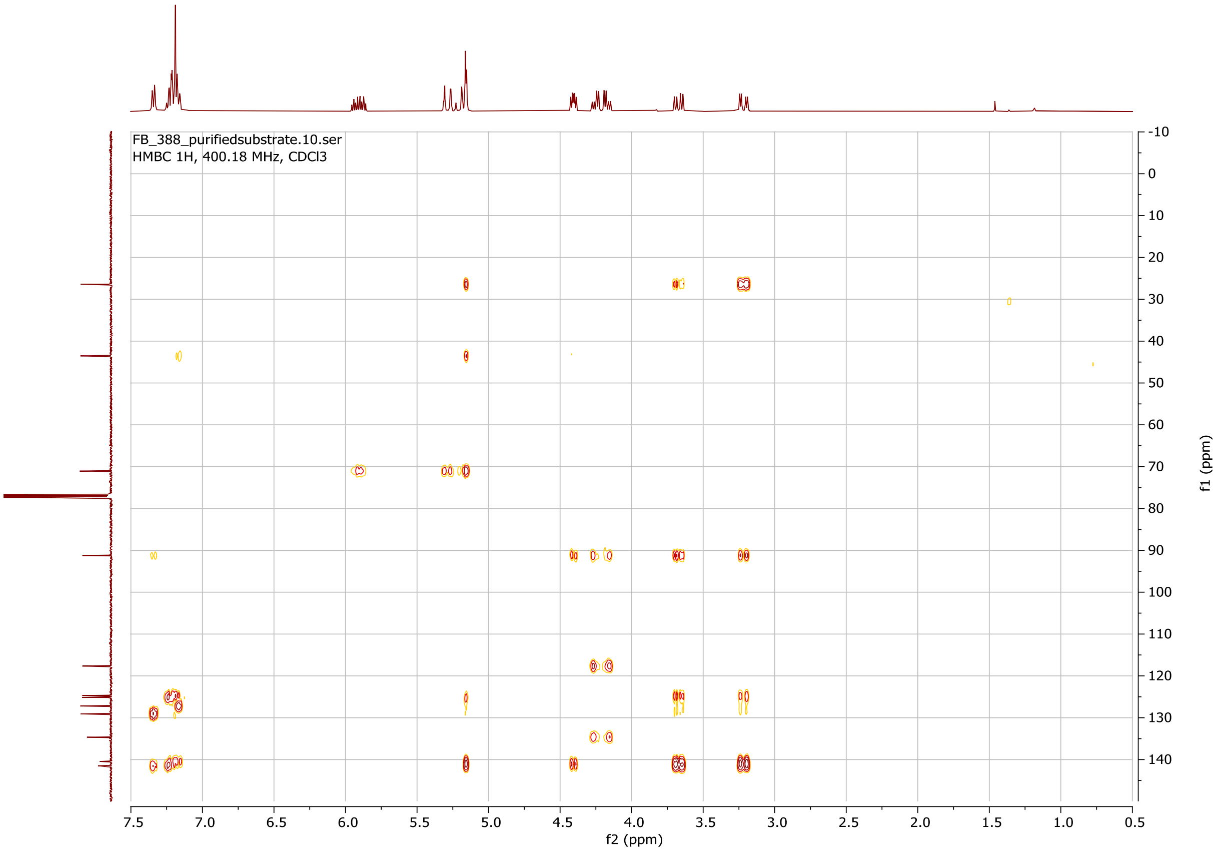
FB_388_purifiedsubstrate.10.ser
HMBC 1H, 400.18 MHz, CDCl3
f2 (ppm)
f1 (ppm)

Inseparable 80:20 mixture of 3-(iodomethyl)-3,3a,4,8b-tetrahydro-2*H*-indeno[1,2-*b*]furan **10** and 3-methyl-3,3a,4,8b-tetrahydro-2*H*-indeno[1,2-*b*]furan **11**

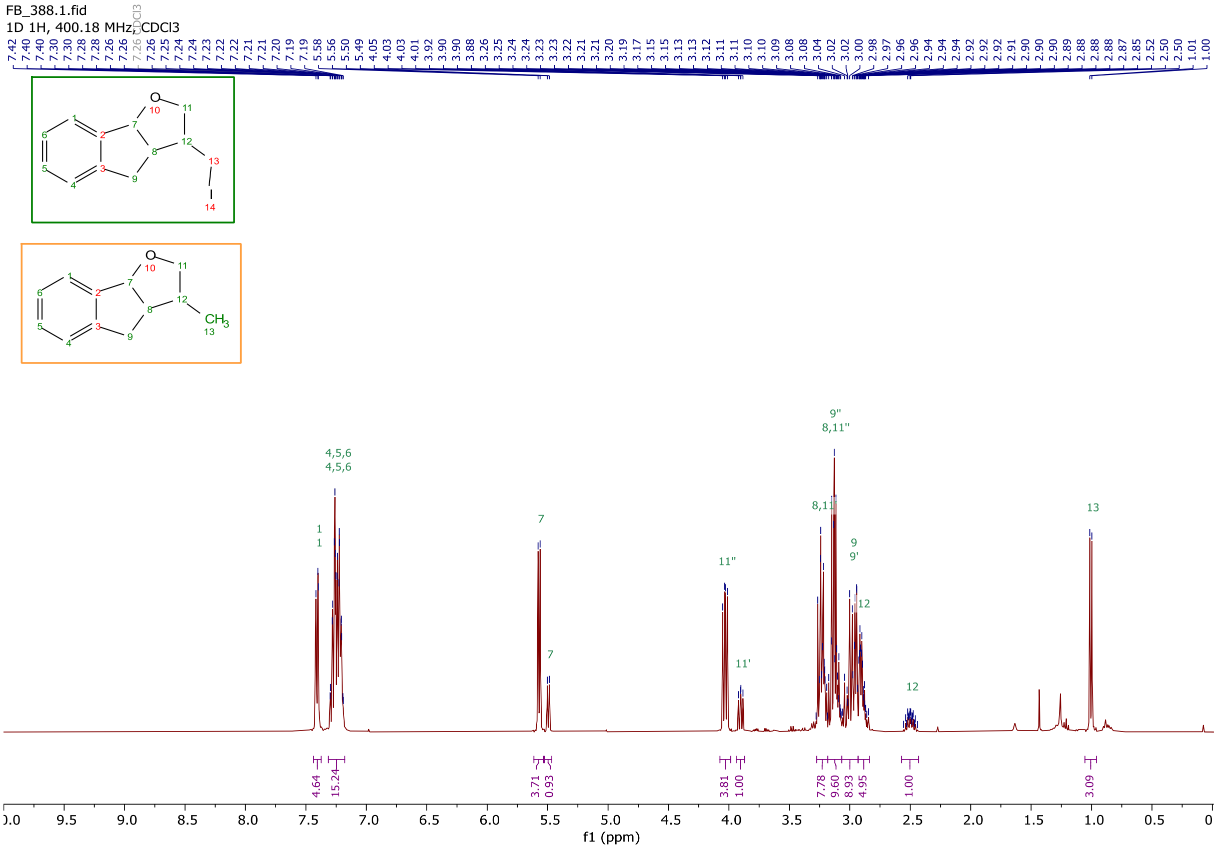

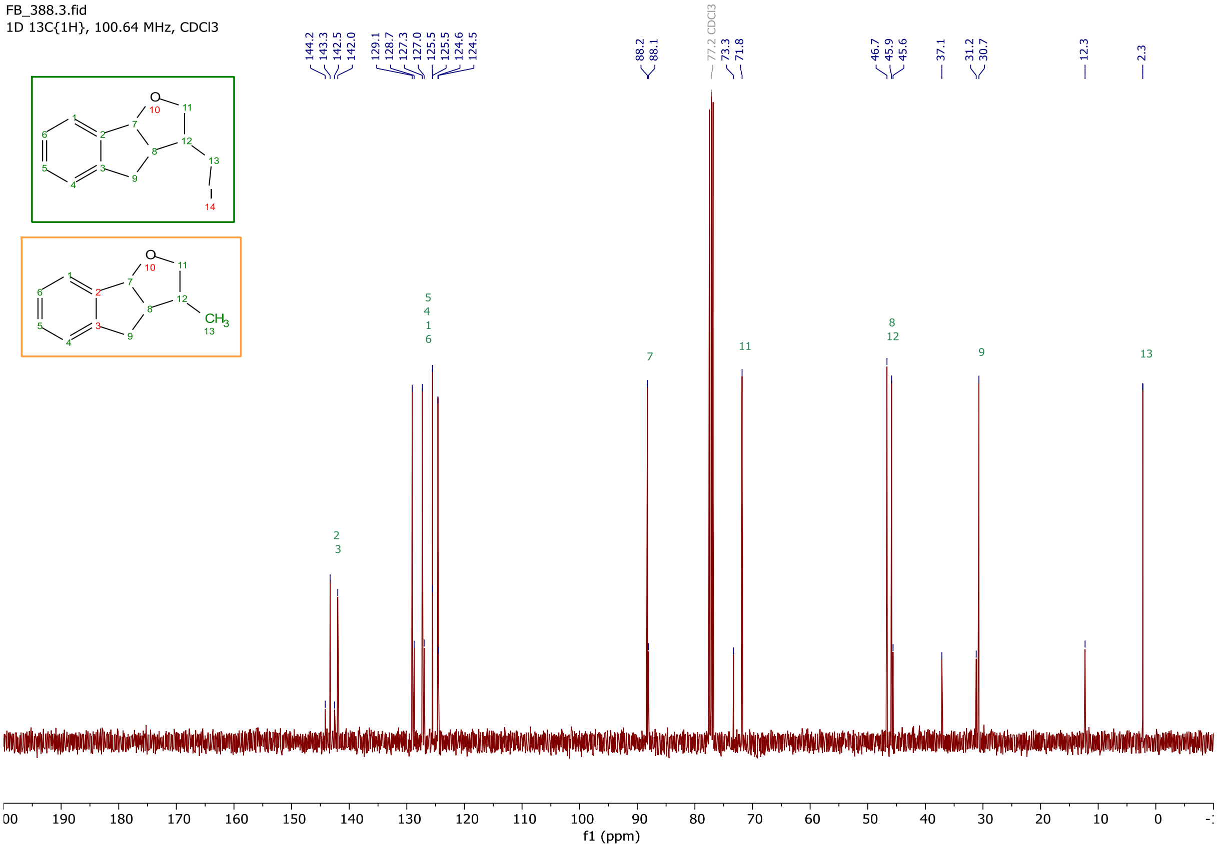

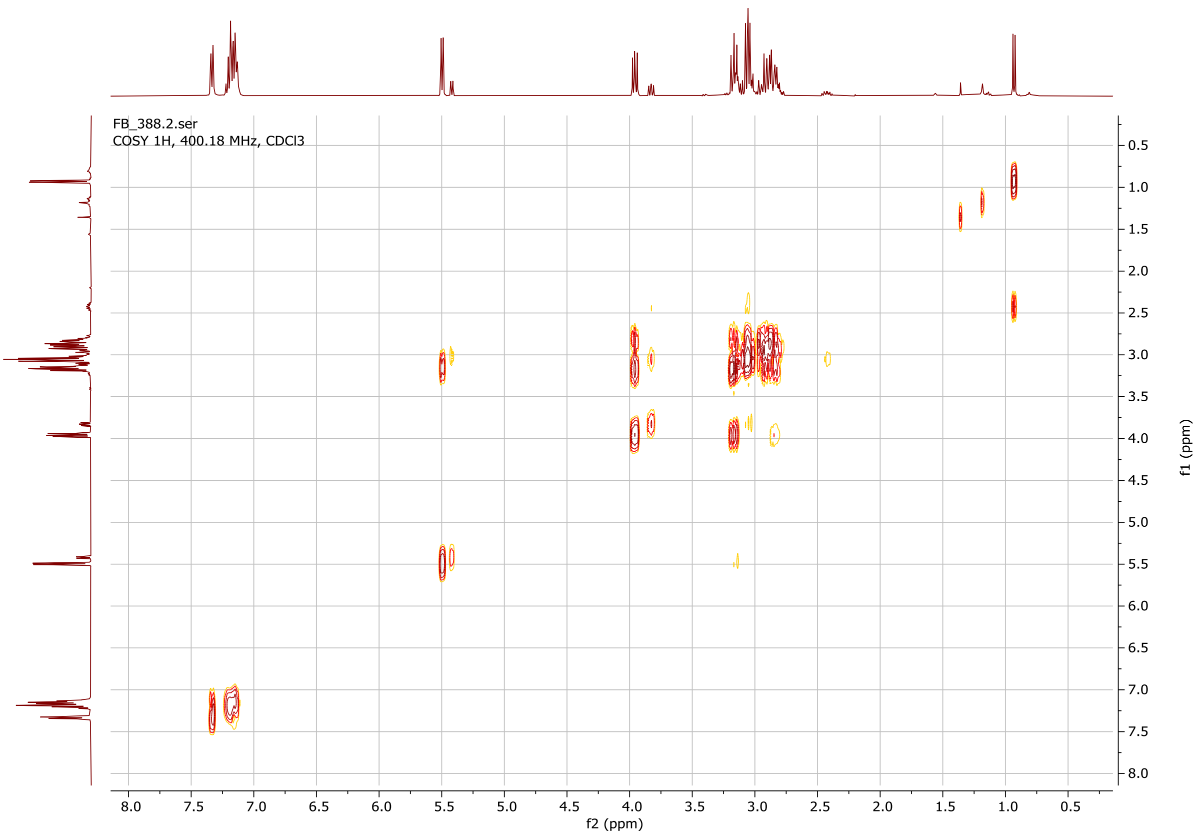
FB_388.2.ser
COSY 1H, 400.18 MHz, CDCl3
f2 (ppm)
f1 (ppm)

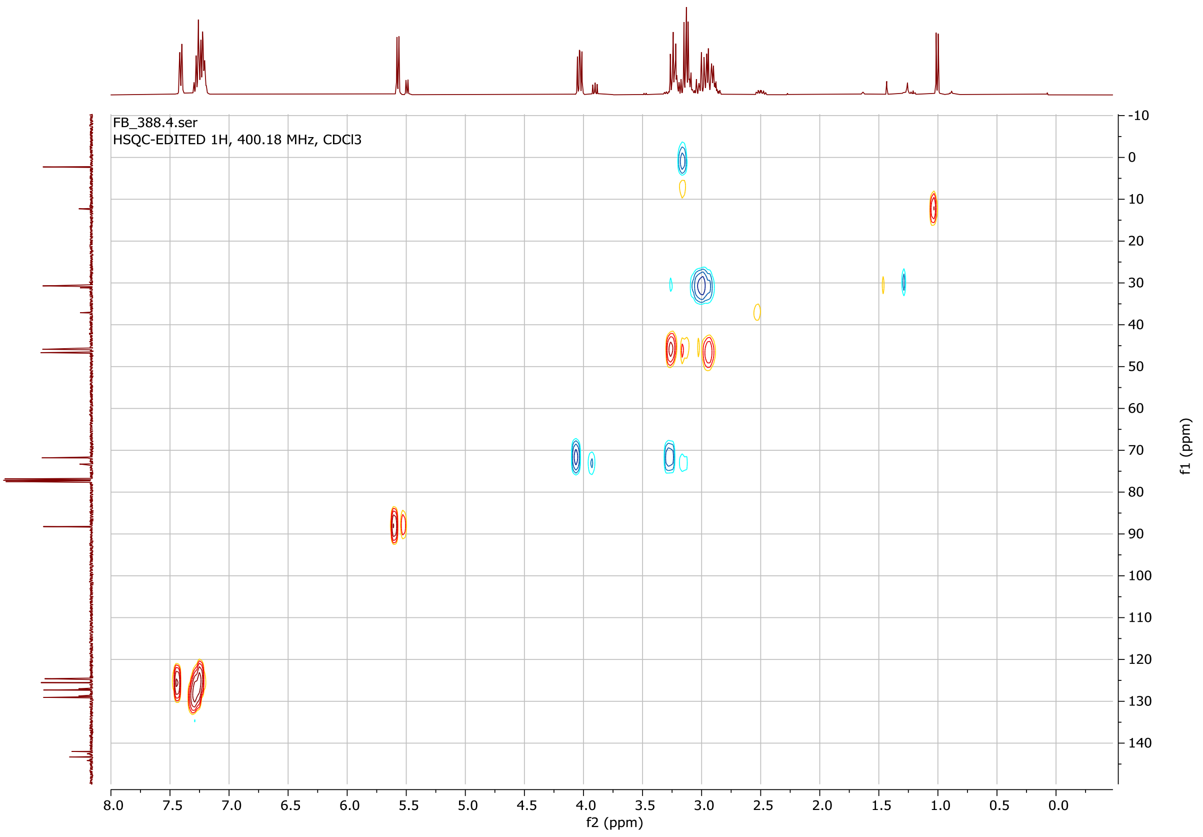
FB_388.4.ser
HSQC-EDITED 1H, 400.18 MHz, CDCl3
f2 (ppm)
f1 (ppm)

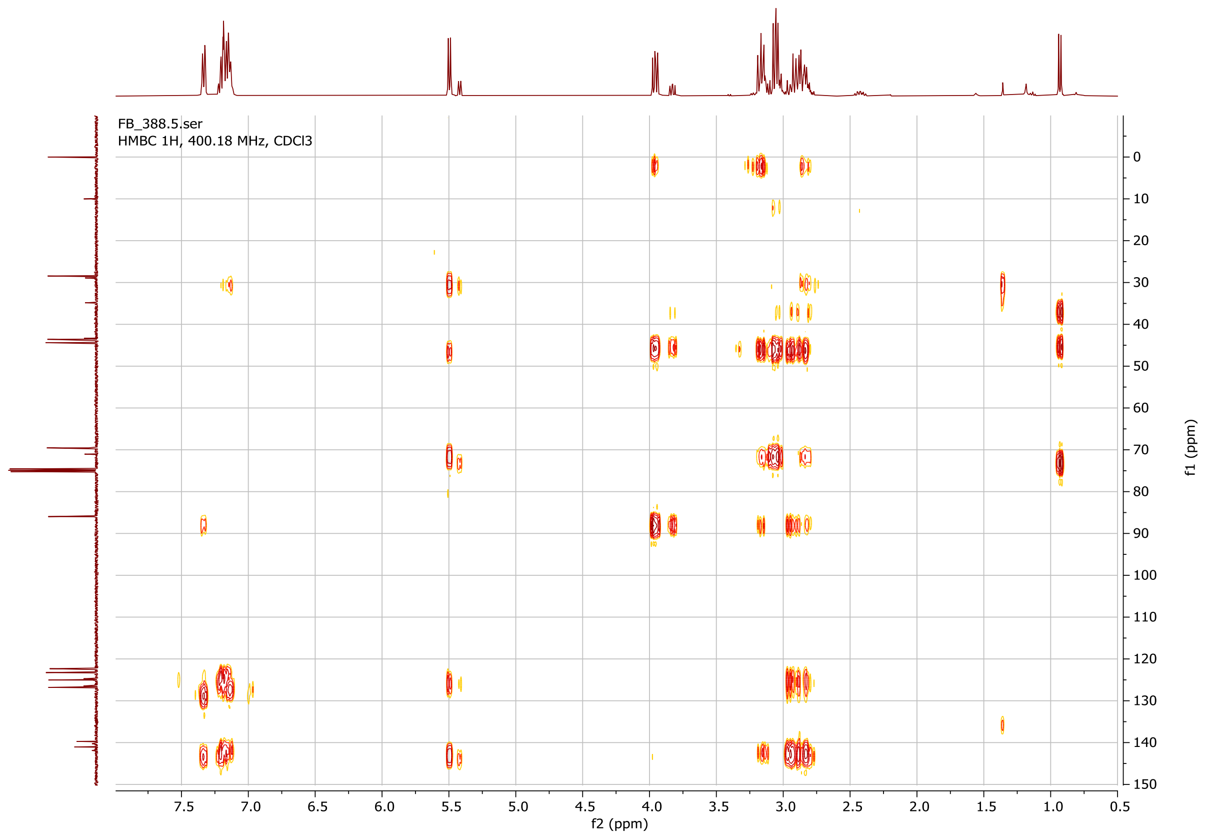

1,1,3,3-Tetramethyl-1,3-bis((2-vinyl-2,3-dihydro-1*H*-inden-1-yl)oxy)disiloxane **13a**

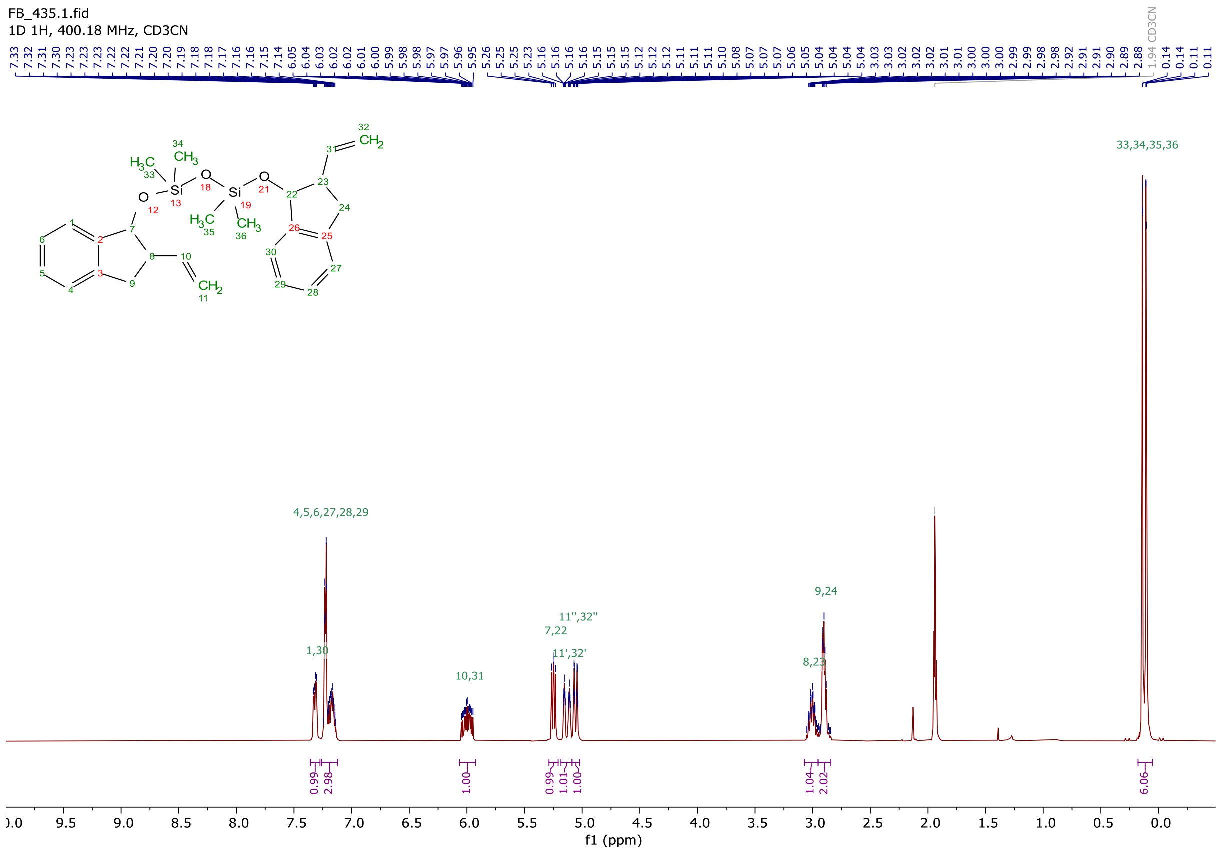

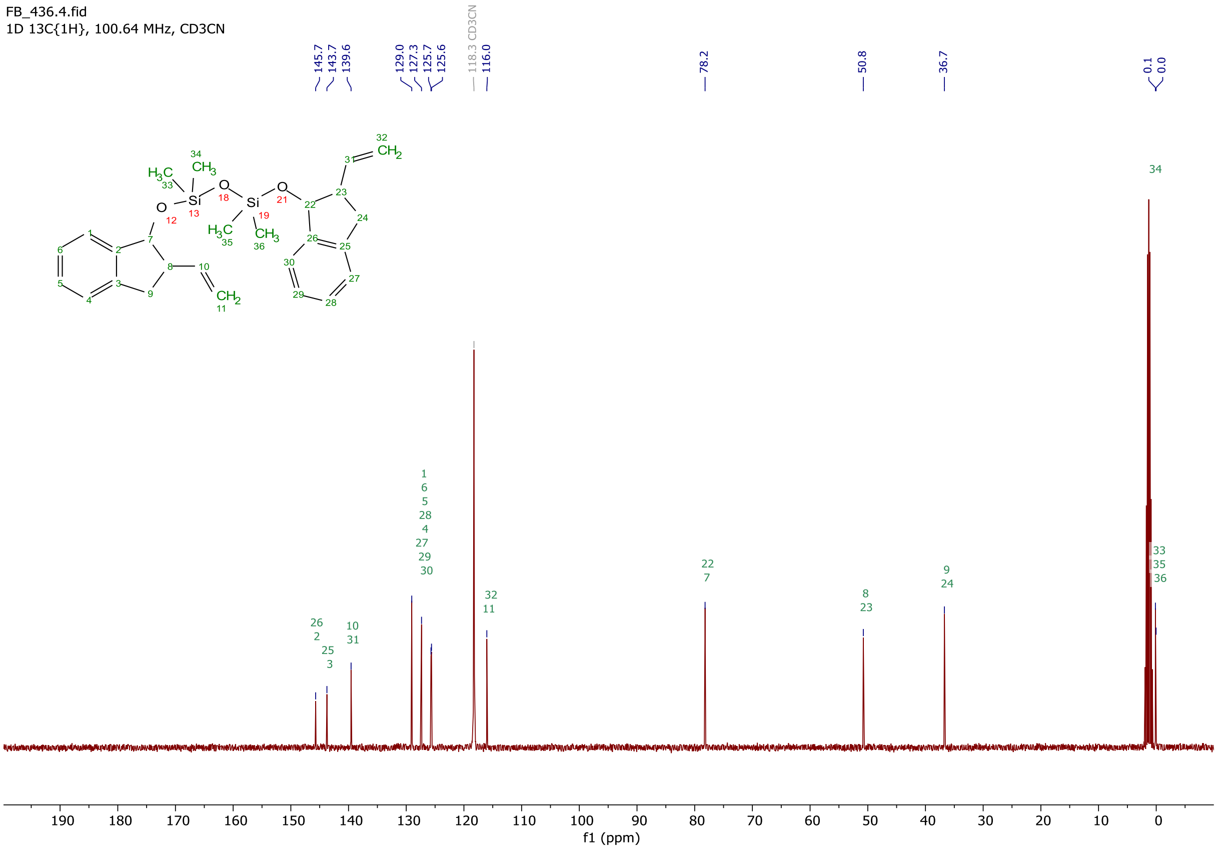
FB_436.4.fid
1D 13C{1H}, 100.64 MHz, CD3CN
145.7
143.7
139.6
129.0
127.3
125.7
125.6
118.3 CD3CN
116.0
78.2
50.8
36.7
0.1
0.0
f1 (ppm)

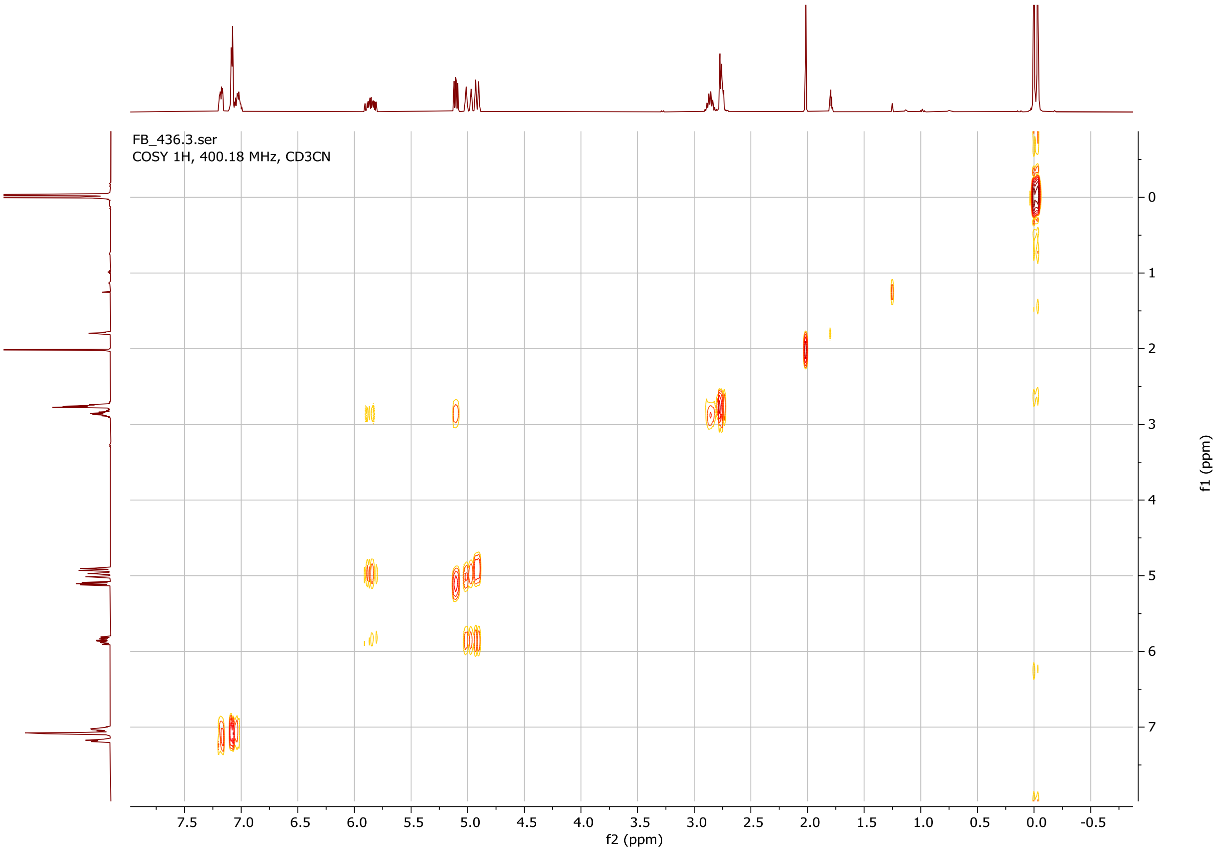
FB_436.3.ser
COSY 1H, 400.18 MHz, CD3CN
f2 (ppm)
f1 (ppm)

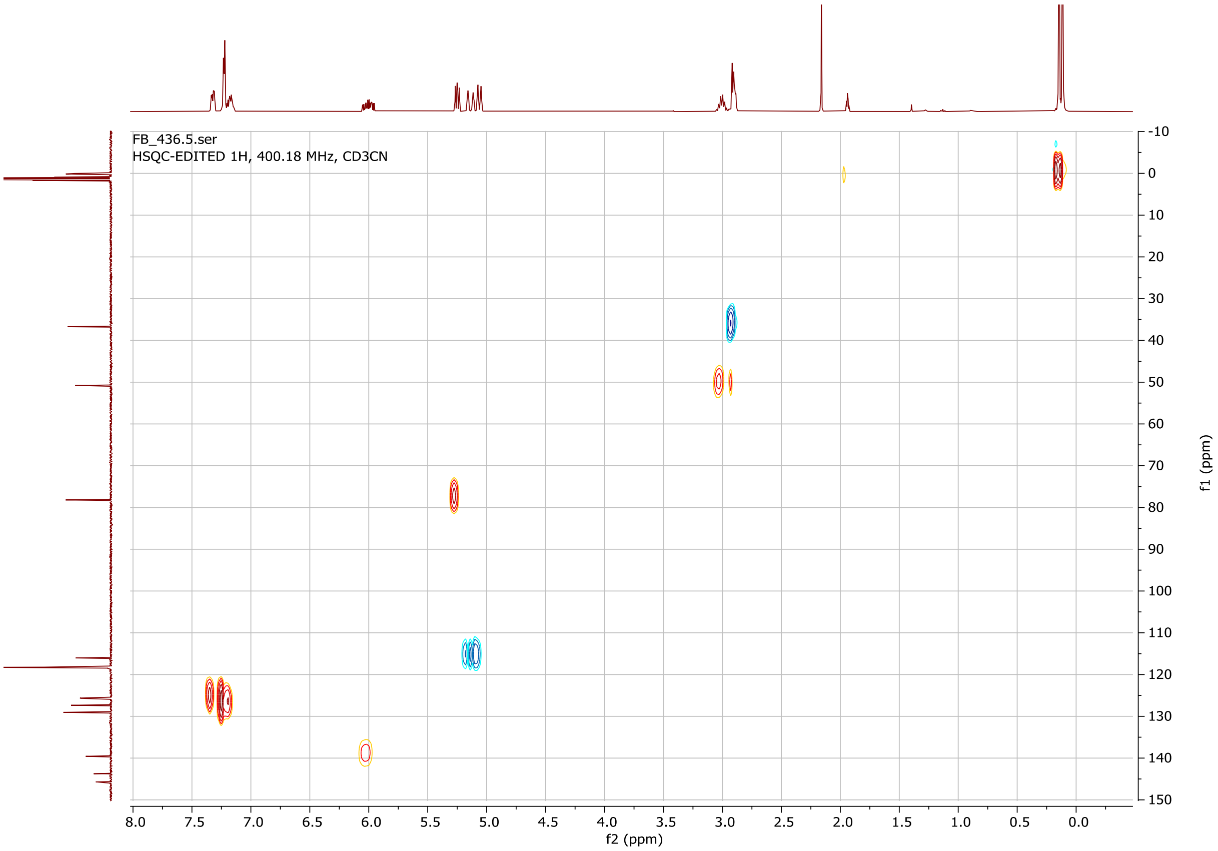
FB_436.5.ser
HSQC-EDITED 1H, 400.18 MHz, CD3CN
f2 (ppm)
f1 (ppm)

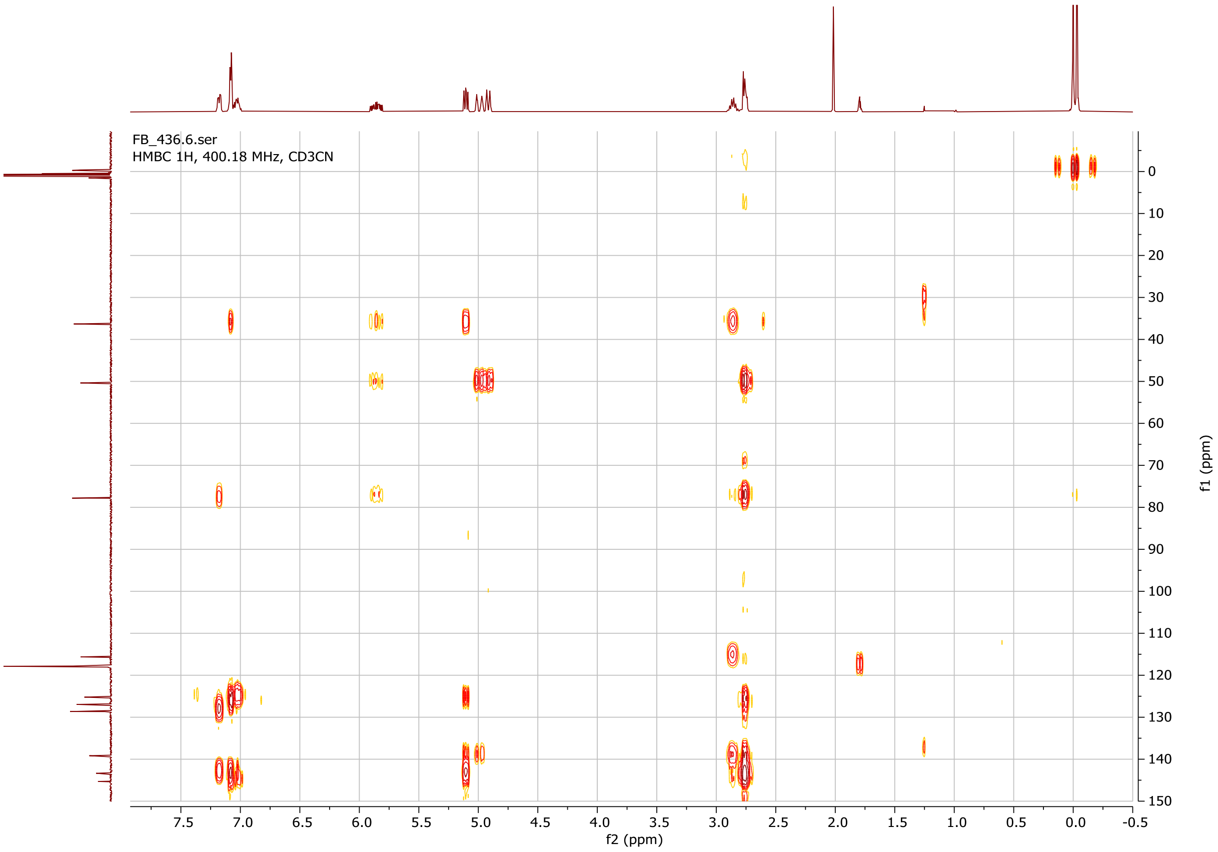
FB_436.6.ser
HMBC 1H, 400.18 MHz, CD3CN
f2 (ppm)
f1 (ppm)
